# Thermodynamic Modulation of Dihydrogen Activation Through Rational Ligand Design in Ge^II^–Ni^0^ Complexes

**DOI:** 10.1021/jacs.4c08297

**Published:** 2024-08-06

**Authors:** Philip M. Keil, Sophia Ezendu, Annika Schulz, Malte Kubisz, Tibor Szilvási, Terrance J. Hadlington

**Affiliations:** † Fakultät für Chemie, Technische Universität München, Lichtenbergstraße 4, 85748 Garching bei München, Germany; ‡ Department of Chemical and Biological Engineering, University of Alabama, Tuscaloosa, Alabama 35487, United States

## Abstract

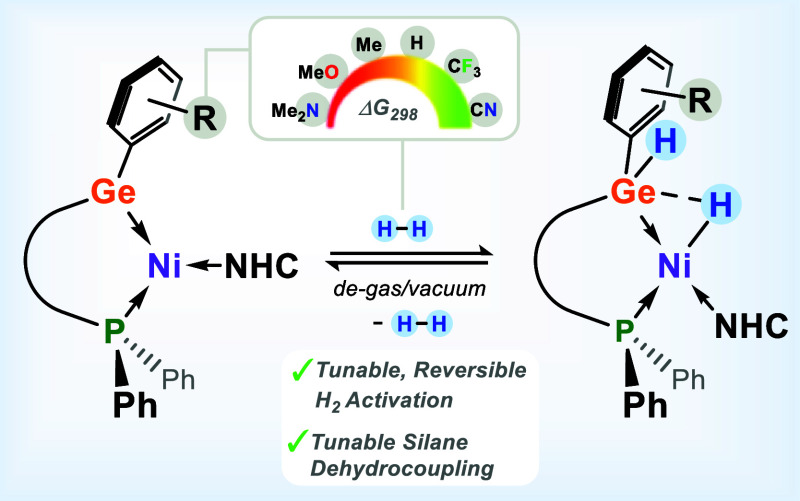

A family of chelating aryl-functionalized germylene ligands has been developed and employed in the synthesis of their corresponding 16-electron Ni^0^ complexes (^PhiP^DippGeAr·Ni·IPr; ^PhiP^Dipp = {[Ph_2_PCH_2_Si(^i^Pr)_2_](Dipp)N}^−^; IPr = [{(H)CN(Dipp)}_2_C:]; Dipp = 2,6-^i^Pr_2_C_6_H_3_). These complexes demonstrate the ability to cooperatively and reversibly activate dihydrogen at the germylene-nickel interface under mild conditions (1.5 atm H_2_, 298 K). We show that the thermodynamics of the dihydrogen activation process can be modulated by tuning the electronic nature of the germylene ligands, with an increase in the electron-withdrawing character displaying more exergonic Δ*G*_298_ values, as ascertained through NMR spectroscopic Van’t Hoff analyses for all systems. This is also shown to correlate with experimental ^31^P NMR and UV/vis absorption data as well as with computationally derived parameters such as Ge–Ni bond order and Ni/Ge NPA charge, giving a thorough understanding of the modulating effect of ligand design on this reversible, cooperative bond activation reaction. Finally, the utility of this modulation was demonstrated in the catalytic dehydrocoupling of phenylsilane, whereby systems that disfavor dihydrogen activation are more efficient catalysts, aligning with H_2_-elimination being the rate-limiting step. A density functional theory analysis supports cooperative activation of the Si–H moiety in PhSiH_3_.

## Introduction

The binding, activation, and utilization of dihydrogen has been and remains a central research focus across numerous facets of chemistry,^1^ centered perhaps most prominently in hydrogenation and dehydrogenation catalysis.^2^ Understanding such processes is increasingly more important, in discovering sustainable, efficient catalysts, and more broadly in realizing the effective use of H_2_ as an energy carrier.^3^ Classical heterogeneous systems (e.g., Raney nickel,^4^ Pd/C^5^) remain useful tools for achieving organic reduction chemistry. Still, molecular systems benefit in allowing in situ monitoring and well-defined structure–reactivity relationships,^6^ leading to fewer challenges in understanding and tuning, relative to heterogeneous systems. Molecular homogeneous catalysts for hydrogen activation are thus intensely investigated and historically dominated by precious heavier transition metals (TMs).^2^ Due to a necessary shift toward utilizing Earth-abundant metals for catalysis,^7^ homogeneous catalytic systems featuring 3*d* TMs are becoming increasingly more explored.^7,8^ In order to tame adverse processes associated with these metals (e.g., single electron mechanisms), and indeed to open new reactive pathways for heavier TMs, noninnocent ligands have played a pivotal role (i.e., metal–ligand cooperativity, MLC).^9^ For 3*d* metals, MLC has allowed for the facile activation of a number of catalytically relevant small molecules at the ligand–metal interface, including H_2_, showing particular promise in (de)hydrogenation catalysis employing these Earth-abundant metals.^9d,10^ Among these, well-defined examples of energetically tunable and reversible dihydrogen activation are rare, which would otherwise allow for a greater depth of understanding of ligand effects on this fundamentally significant process.

**Figure 1:**
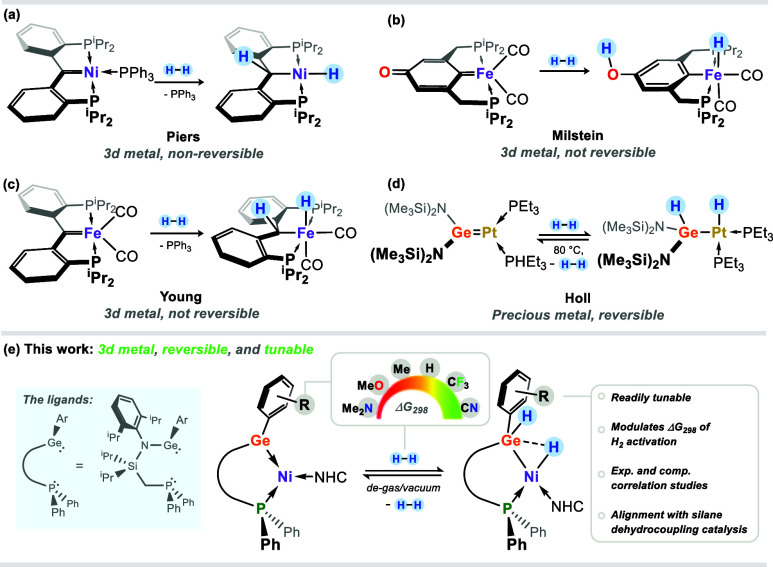
(a–c) Known examples of cooperative H_2_ activation with 3*d* metal carbene complexes; (d) reversible H_2_ activation in a Ge–Pt complex; (e) this work.

Carbenes, which have gained broad attention as both key reactive intermediates and spectator ligands,^11^ have also been explored as noninnocent ligands.^12^ Typically incorporated into a chelating ligand scaffold, noninnocent carbene ligands have allowed for the reversible activation of ammonia,^13^ as well as the stoichiometric activation of H_2_,^13,14^ in a small number of 3*d* TM complexes (Figure 1a–c). We note that H_2_ activation via this mechanism is known for a considerably greater number of heavier, precious TM systems.^12b,15^ The utilization of heavier *p*-block element centers in assisting H_2_ activation has gained significant attention in recent years, particularly in Lewis acidic group 13 moieties.^10c,16^ The heavier tetrylenes have also garnered some degree of attention here: a handful of silylene complexes of 3*d* TMs have been shown to activate H_2_ across the Si-TM bond,^17^ while one example of a Ge^II^–Pt^0^ system even demonstrates the reversible scission of H_2_ (Figure 1d),^18^ albeit with a precious TM. Such systems remain relatively unexplored. Still, tetrylenes are promising candidates as noninnocent ligands for several reasons: they are readily modifiable, perhaps even more so than classic carbenes, for example, through simple salt metathesis reactions.^19^ Through this, their electronic nature is readily tunable, as is their steric profile.^20^ The lessened propensity for *sp*-mixing on descending group 14 further allows tuning of their ambiphilic character, i.e., the HOMO–LUMO gap for a given system increases down the group.^20^ Our own work has demonstrated this, whereby tetrylenes can behave as Lewis acidic binding sites while coordinating Ni^0^ through their lone electron pair.^21^ In those systems, no MLC is observed due to the saturated 18-electron Ni center. We thus hypothesized that cooperative chemistry may be accessible on moving to a 16-electron Ni^0^ system and further that the energetics of this cooperativity may be modulated through ready modification of the germylene ligand. Herein, we describe efforts in this regard toward a deep understanding of factors affecting the energetics of dihydrogen activation in 16-electron Ni^0^ complexes bearing noninnocent germylene ligands. We find that electronic modulation of these ligands allows for the fine-tuning of the thermodynamics of H_2_ activation, while also allowing for complete inhibition of the H_2_ activation process. Experimental kinetic studies show a strong correlation with key NMR and UV/vis spectral data as well as with DFT-derived charge and bonding parameters, giving a clear understanding of ligand effects on H_2_ activation. Further, the described 16-electron nickel(0) complexes are effective in catalytic phenylsilane dehydrocoupling, with the catalytic rate also correlating with the catalyst’s ability to eliminate H_2_.

## Results and Discussion

### Ligand and Complex Development

**Figure 2:**
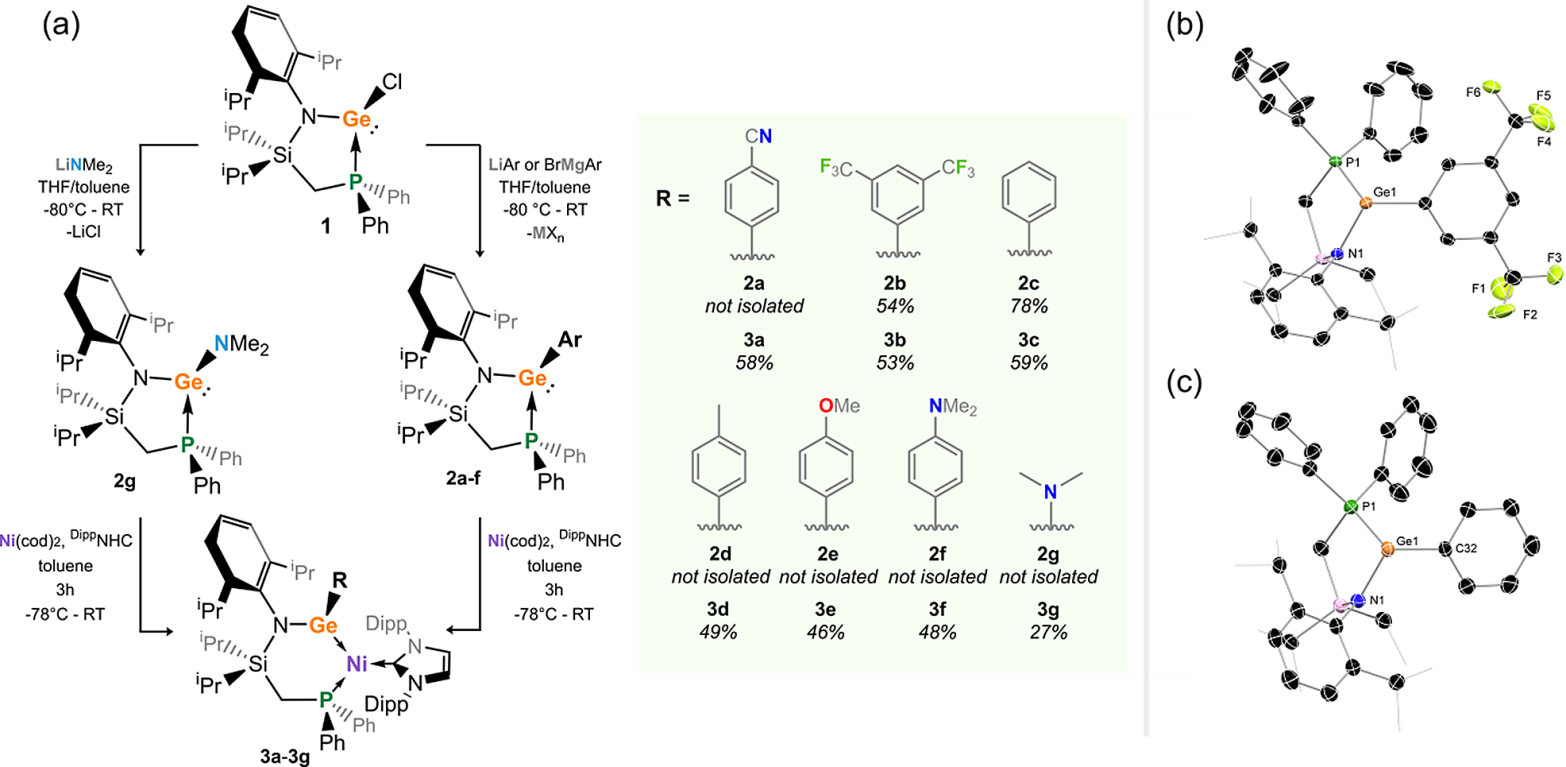
(a) Synthesis of germylene ligands 2a–g, and subsequent synthesis of 16-electron Ni^0^ complexes **3a**–**3g**. Inset graphic indicates yields of ligands and subsequent complexes**;** and molecular structureas of (b) **2b** and (c) **2c**, with hydrogen atoms omitted, and probability ellipsoids set at 30%. Selected distances and (Å) angles (deg) for **2b**: P1–Ge1 2.464(1), N1–Ge1 1.940(2), C32–Ge1 2.026(3), P1–Ge1–N1 88.38(6), P1–Ge1–C32 91.76(8), N1–Ge1–C32 105.4(1); for **2c**: P1–Ge1 2.516(2), N1–Ge1 1.944(4), C32–Ge1 2.039(5), P1–Ge1–N1 87.7(1), P1–Ge1–C32 91.9(1), and N1–Ge1–C32 103.9(2).

The starting point for this study was our reported (amido)(chloro)germylene ligand **1** (^PhiP^DippGeCl; ^PhiP^Dipp = {[Ph_2_PCH_2_Si(^i^Pr)_2_](Dipp)N}; Dipp = 2,6-^i^Pr_2_C_6_H_3_), which is readily accessible on a gram scale (Figure 2a).^21a^ We sought to diversify this system through metathesis of the Ge–Cl moiety. For aryl substitution, this was readily achieved with either aryl lithium or Grignard reagents, while the [Me_2_N] system could be generated with Me_2_NLi. This gave access to a family of six electronically distinct (amido)(aryl)germylene ligands (vis. **2a**–**f**), featuring strongly electron-withdrawing substituents (e.g., p-NC-Ph, *m*-(CF_2_)_2_-Ph) and electron-donating substituents (e.g., *p*-Me-, *p*-MeO-, and *p*-Me_2_N-Ph), as well as the “standard” Ph system. In addition, the [Me_2_N] ligand **2g** stands as a highly electron-rich germylene, expected to have a considerably stabilized LUMO through N → Ge π-donation. All systems are formed essentially quantitatively as ascertained by ^31^P NMR spectroscopy but are highly soluble in aliphatic solvents, including pentane. This made the isolation of all systems somewhat challenging, although large-scale synthesis of **2b** and **2c** allowed for their isolation as analytically pure solids in 54 and 78% yields, respectively, and for the growth of X-ray quality crystals (Figure 2b,c).^22^ A high-field shift for the signal relating to the ligand’s [Ph_2_P] moiety is observed in the ^31^P{^1^H} NMR spectra on moving from **2a** to **2g**, in line with increased electron density on moving from the *p*-NC-Ph moiety (δ = 5.9 ppm) to the strongly π-donating [Me_2_N] moiety (δ = −24.4 ppm). This already indicates that the electronic nature at Ge^II^ can be easily tuned through such ligand modifications. Reminiscent of our earlier reports,^21,23^ addition of ligands **2a**–**2g** to 1:1 mixtures of Ni(cod)_2_ and IPr (IPr = [(H)CN(Dipp)]_2_C:) in toluene led to immediate formation of deep purple (**2a**), red (**2b**–**f**), or green (**2g**) solutions, indicative of complex formation (**3a**–**3g**, Figure 2a).

**Figure 3:**
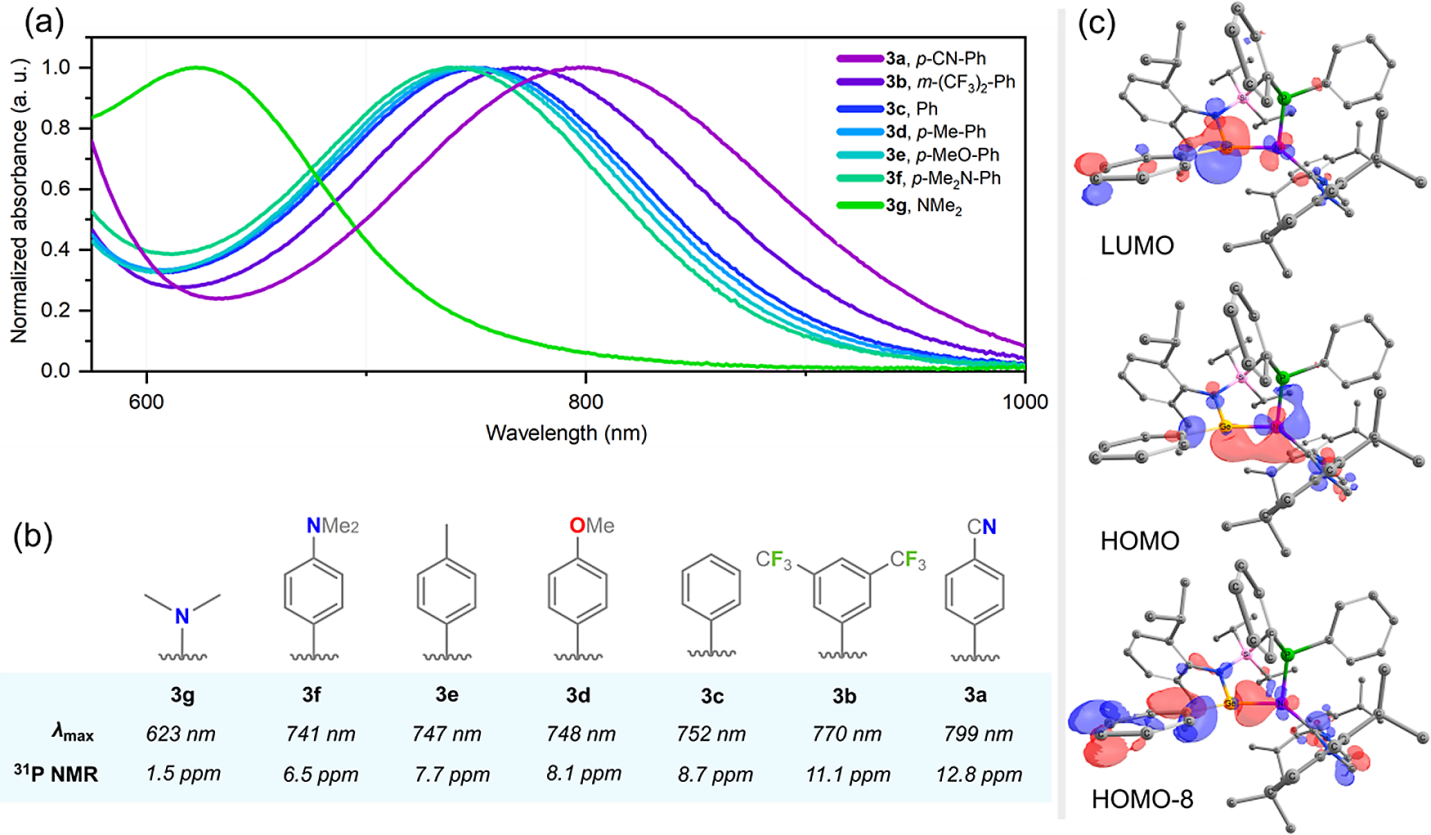
(a) Normalized UV/vis absorption spectra for **3a**–**3g** in the region 580–1000 nm; (b) a summary of key UV/vis absorption and ^31^P{^1^H} NMR spectral data; (c) selected frontier orbitals for **3c**.

**Figure 4:**
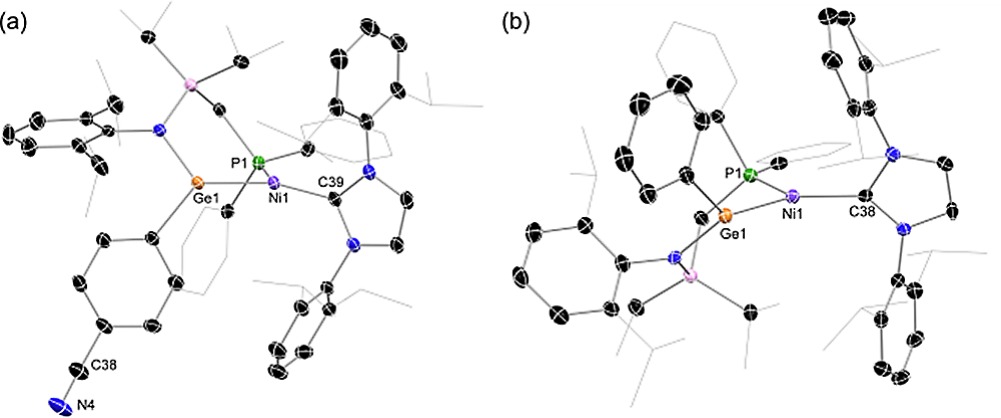
Molecular structure of (a) **3a** and (b) **3c**, with hydrogen atoms omitted and thermal ellipsoids at 30% probability. Selected distances (Å) and angles (deg) for **3a**: Ni1–Ge1 2.219(1), Ge1–N1 1.898(8), Ni1–C38 1.929(8), Ge1–Ni1–C38 133.5(2), 38–Ni1-P1 130.1(2), P1–Ni1–Ge1 96.33(7); for **3c**: Ni1–Ge1 2219(1), Ge1–N1 1.898(8), Ni1–C38 1.929(8), Ge1–Ni1–C38 133.5(2), C38–Ni1–P1 130.1(2), and P1–Ni1–Ge1 96.33(7).

In all cases, ^31^P NMR spectroscopic analysis of crude reaction mixtures suggested the formation of single reaction products, with resonances low-field shifted relative to the free ligands (Figure 3b). All complexes could be isolated as intensely colored crystalline solids in moderate yields of between 46 and 59%. Single-crystal X-ray diffraction analysis revealed all seven complexes to be the target 16-electron Ni^0^ systems, featuring acyclic two-coordinate (aryl)(amido)- or bis(amido)-germylene ligands through the insertion of the nickel center into the Ge–P bond of the “free” ligands (Figures 4, and S129–S133 in SI).^24^ The Ni centers in these complexes bear a trigonal planar geometry, in all cases supported by an agostic C–H···Ni interaction with one ligand Si-^*i*^*Pr* group, leading to a boat-conformation of the 6-membered core of these complexes (Figure S145 in Supporting Information). Indeed, such an interaction is maintained in solution, with one C(H)–C*H*_3_ doublet shifted upfield,^25^ with an increased shift aligning with increased Ge-*Ar* electron-withdrawing nature (Figures S58–S59 in SI), indicative of increased Ni → Ge back-donation. Through this, the Ge centers in all complexes deviate slightly from trigonal planarity, i.e. through slight pyramidalization. Again, this pyramidalization becomes more prominent with the electron-withdrawing nature of the Ge-*Ar* substituent (Table 1), and it is most likely caused by an increased Ni → Ge back-donation. This is further borne out by the ^31^P NMR spectra of **3a**–**3g**, in which a deshielding of the ligand’s P-center is observed along the same series (Figures 3b and S60 in SI), indicative of increased P → Ni donation. These points seem to have little effect on the experimental Ge–Ni bond lengths in these systems, which show no correlation with their aryl substituents. As one might expect, however, these are shorter than those in related N-heterocyclic germylene-Ni^0^ complexes,^26^ but longer than observed for the strongly electron-withdrawing [(F_5_C_6_)_2_Ge] ligand.^27^ The modulation of the electronic nature of this bond across this series is nevertheless clearly observed upon comparison of their UV/vis spectra (Figure 3a). Here, a gradual but significant red shift is observed on moving from the most electron-rich ligand system (**3g**: λ = 623 nm) to the most electron-deficient system (**2a**: λ = 799 nm). This absorption relates to the HOMO–LUMO gap of these complexes,^28^ late the frontier orbitals in **3a–3g**, having a distinct effect on reactivity (vide infra).

**Table 1:** Selected Experimental and Calculated Metrical Data for Compounds **3a**–**3g**

complex	*d*_GeNi_ (Å)	∠_NGeNi_ (deg)	λ_max_ (nm // eV)^a^	Δ*E*_HOMO/LUMO_ (eV)^b^
**3a**	2.2216(8)	117.1(1)	799 // 1.55	1.43
**3b**	2.209(1)	116.3(1)	770 // 1.61	1.55
**3c**	2.219(1)	116.8(2)	752 // 1.65	1.62
**3d**	2.231(1)	116.6(1)	748 // 1.66	1.62
**3e**	2.2203(8)	117.7(1)	747 // 1.66	1.64
**3f**	2.2176(9)	117.5(1)	741 // 1.67	1.64
**3g**	2.2162(9)	118.16(8)	623 // 1.99	1.80

a Determined with UV/vis spectroscopy.

b Calculated using the B97-D3 functional at the def2-SVP(def2-TZVP: Ge,Ni), with SMD: benzene solvent model.

Finally, assessing the electronic structure of the studied systems using Density Functional Theory (DFT) further bolsters the experimentally observed trends. First, the HOMO of all complexes is a distorted π-bond, showing significant Ge–Ni bonding character (e.g., Figure 3c). For all aryl systems, the LUMO represents the antibonding part of this orbital, that is, a distorted π*-orbital, with delocalization across the Ge-*Ar* substituent becoming more prominent with increasing electron-withdrawing character. This is not the case for bis(amido)germylene complex **3g**, where the LUMO is largely ligand-centered, and the LUMO + 1 represents the above-described Ge–Ni antibonding orbital. The calculated HOMO–LUMO gaps correlate well with those found experimentally, i.e., using UV/vis spectroscopy (Table 1), although generally underestimating this value. An NBO analysis of the Ge–Ni bonding interactions in **3a**–**3g** (Tables S22–S28) shows only one σ-type bond between the Ge and Ni atoms, in addition to empty *p*-orbitals on the Ge centers with considerable partial occupation (∼0.5e). This NBO-derived bonding picture is consistent with calculated Mayer Bond Orders (MBOs) which also indicate a single bond with additional secondary interactions (MBO ∼ 1.1). Natural population analysis (NPA) shows a partial negative charge on the Ni center (−0.512 to −0.546) and a positive charge on the Ge center (+1.116 to +1.160). Throughout the series of ligands, we see a clear trend in the MBO of the Ni–Ge bond with the partial charge of the Ge and Ni atoms, which also correlates with measured UV/vis and H_2_ activation parameters (vide infra). Among the series of **3a**–**3g**, **3g** provides a considerable difference in the Mayer bond order for the Ni–Ge bond (1.23) and partial charges (Ni: −0.59; Ge: + 1.31), indicating significant tunability of the Ge–Ni interaction through the described ligand design.

### Reversible Dihydrogen Activation

**Scheme 1:**
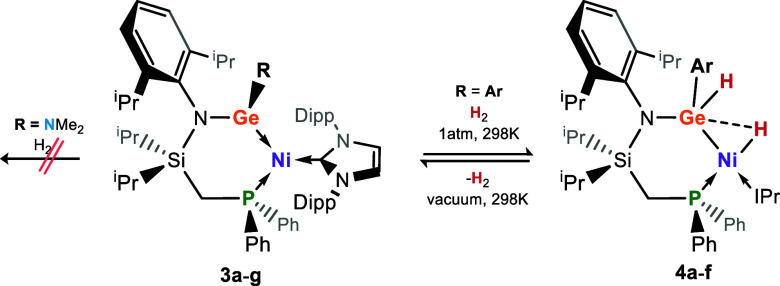
Reversible Activation of Dihydrogen by Complexes **3a–f**

**Figure 5:**
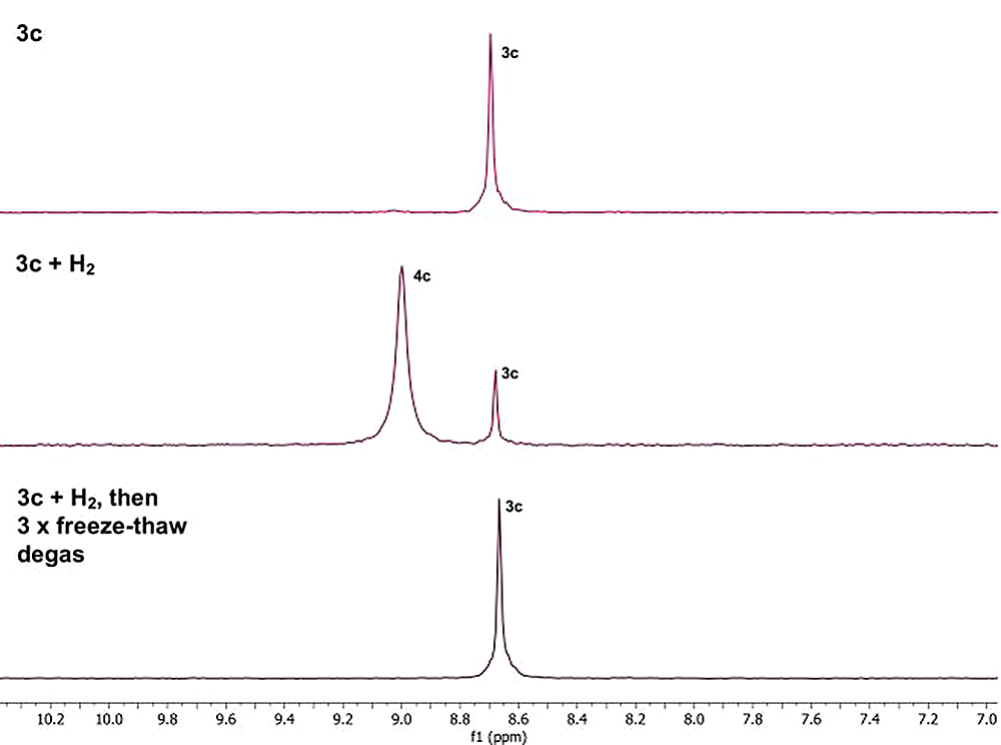
Stack-plot of ^31^P{^1^H} NMR spectra showing the reversible activation of H_2_ by **3c**, forming **4c**.

With complexes **3a**–**g** in hand, we aimed to investigate the effect of the described electronic modulation on the reactivity of these systems. The (amido)(aryl)germylene complexes **3a**–**3f** readily activate dihydrogen under just 1.5 atm of pressure at ambient temperature, each leading to a single activation product as ascertained by ^1^H and ^31^P{^1^H} NMR spectroscopy (**4a**–**4f**, Scheme 1).^29^ Evidence for cooperativity in these reactions came from variable-temperature (VT) NMR spectroscopic studies. Between −40 and −60 °C, a clear resonance for the Ge-*H* can be seen for all complexes between δ = 5.22 (**3f**) and 6.03 (**3a**) ppm. The corresponding Ni-*H* is observed as a broad doublet between δ = −9.76 (**3f**) and −10.83 (**3a**) ppm. Both resonances integrate to 1H, and couple with each other as shown via 2D-COSY NMR experiments for **3b** (Figure S72 in SI). Though these peaks are too broad to ascertain ^3^J_HH_ coupling constants, the signals observed for the Ni-*H* at low temperatures give ^2^J_PH_ coupling constants between 105 and 109 Hz, in keeping with known *trans-phosphine* nickel hydride complexes.^30^ As a whole, this demonstrates the activation of H_2_ across the Ge–Ni bond, forming novel germyl-nickel species with a [HGeNiH] core. Confirming this, these signals are not observed on the addition of D_2_ to **3c**, while the remainder of the spectrum is unchanged. At ambient temperature no clear signals are observed for the described hydride ligands in the H_2_ activation products, while at 60 °C all species exhibit a resonance at approximately δ = −2 ppm, integrating to 2H. These observations would suggest that a dynamic hydride exchange is in play in these complexes, as has been observed previously in the activation of silanes by Ni^0^ complexes.^31^ This dynamic exchange is further demonstrated by the addition of D_2_ to in situ generated hydride complex **4c**, leading to the formation of H–D (Figure S83 in SI). Notably, degassing reaction mixtures of in situ generated H_2_ activation complexes **4a**–**4f** leads to quantitative regeneration of **3a**–**3f** (e.g., Figure 5), indicative of complete reversibility in this H_2_ activation process. Importantly, bis(amido)germylene complex **3g** does not activate dihydrogen under the given conditions, or when heated under an atmosphere of H_2_, already indicating that electronic modulation of these complexes allows for tailored bond-activation capacities.^32^

**Figure 6:**
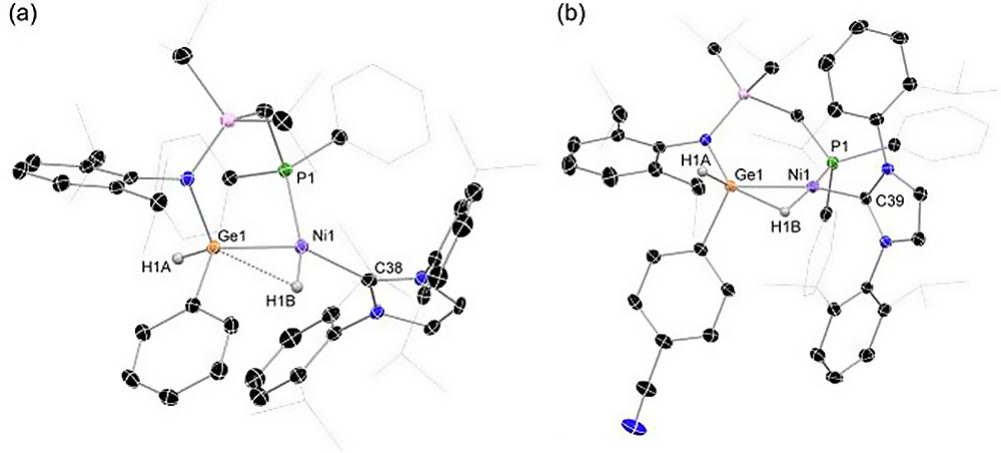
Molecular structures of (a) **4c** and (b) **4a**, with hydrogen atoms omitted, aside from Ge–H and Ni–H moieties, and probability ellipsoids set at 30%. Selected bond distances (Å) and angles (deg) for **4c**: Ni1–Ge1 2.312(1), Ge1–N1 1.931(4), Ni1–C38 1.920(5), Ni1–P1 2.174(1), C38–Ni1–P1 111.3(1), P1–Ni1–Ge1 92.16(4); for **4a**: Ni1–Ge1 2.323(1), Ge1–N1 1.923(3), Ni1–C39 1.918(3), Ni1–P1 2.201 (1), C39–Ni1–P1 114.71(9), P1–Ni1–Ge1 100.16(4).

The structure of the described complexes was confirmed through single-crystal X-ray diffraction analysis of **4a** and **4c** (Figure 6), which confirms the proposed 1,2-dihydride structure observed in low-temperature ^1^H NMR spectra.^33^ Given the aforementioned reversibility of this H_2_ activation reaction, crystals were grown under an atmosphere of H_2_. Attaining X-ray quality crystals of all species was thus not possible, but microcrystalline solids could be isolated for all systems except **3b**, through precipitation of these complexes from their concentrated solutions at ambient temperature. This allowed the collection of ATR-IR spectra for these H_2_ activation products, with Ge–H and Ni–H stretching frequencies observed between 1934 and 1949, and 1878–1893 cm^–1^, respectively. Both **4a** and **4c** bear a distorted square planar geometry at Ni (sum of angles at Ni: **4a** = 360.97°; **4c** = 360.46°), with a bridging Ni–H···Ge interaction. The Ge centers hold a tetrahedral geometry, with Ge–Ni bonds elongated relative to those in **3a** and **3c** (*d*_GeNi_: **3a** = 2.2216(8) Å; **4a** = 2.323(1) Å; **3c** = 2.219(1) Å; **4c** = 2.312(1) Å), presumably due to the absence of π-back-donation from Ni to Ge in accordance with now coordinately saturated Ge centers. We note that although both the Ge and Ni centers in complexes **3a** and **3c** are in fact chiral, no selectivity for differing diastereomers is observed given that both species crystallize in centrosymmetric space groups (P*2*_*1/n*_ and P*-1*, respectively).

### Thermodynamic Analysis of H_2_ Activation

**Figure 7:**
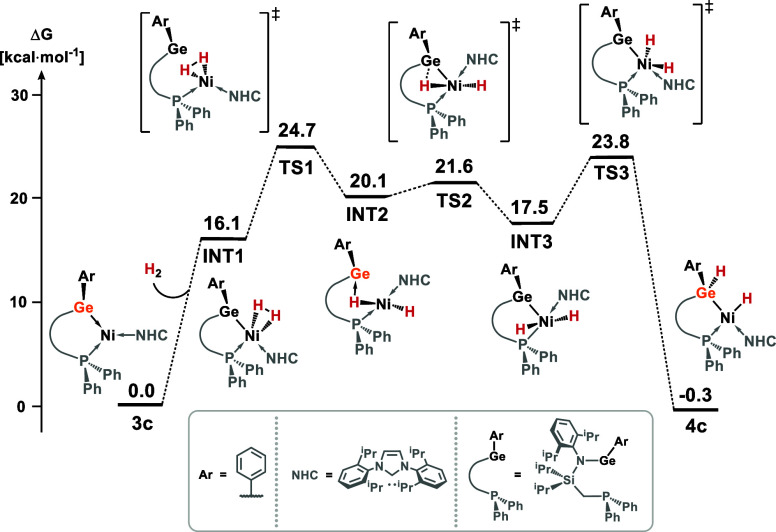
DFT-derived mechanism for the cooperative activation of H_2_ by **3c**.

To gain further insights into the H_2_ activation reaction, we used DFT to calculate the thermodynamics and reaction profile for complexes **3a–3g**. The calculated reaction profile for **3c** can be found in Figure 7, whereas the energetics of the same reaction steps for all synthesized complexes can be found in Table S29. We find that the initial coordination of an H_2_ molecule at Ni is a barrierless process, forming an η^2^-complex (INT1, 16.1 kcal·mol^–1^). The activation of the H_2_ bond occurs with the concurrent coordination of one of the H atoms to the Ge center (TS1, 24.7 kcal·mol^–1^) forming a square-pyramidal Ni-dihydride intermediate, the germylene ligand at the apex (INT3, 17.5 kcal·mol^–1^). The reaction concludes with the insertion of the germylene into one Ni–H bond (TS3, 23.8 kcal·mol^–1^) forming near thermoneutral product **4c** (−0.3 kcal·mol^–1^). The activation barrier for a number of the reaction steps in this mechanism is rather similar for all ligands, i.e., within a few kcal·mol^–1^. In addition to these differences being within the expected accuracy of DFT methods (∼5 kcal/mol), the formal rate-determining step may well change from ligand to ligand. Nevertheless, that calculated Δ*G* values are essentially thermoneutral for all arylgermylene systems is in line with the experimentally observed reversibility for this reaction.

**Figure 8:**
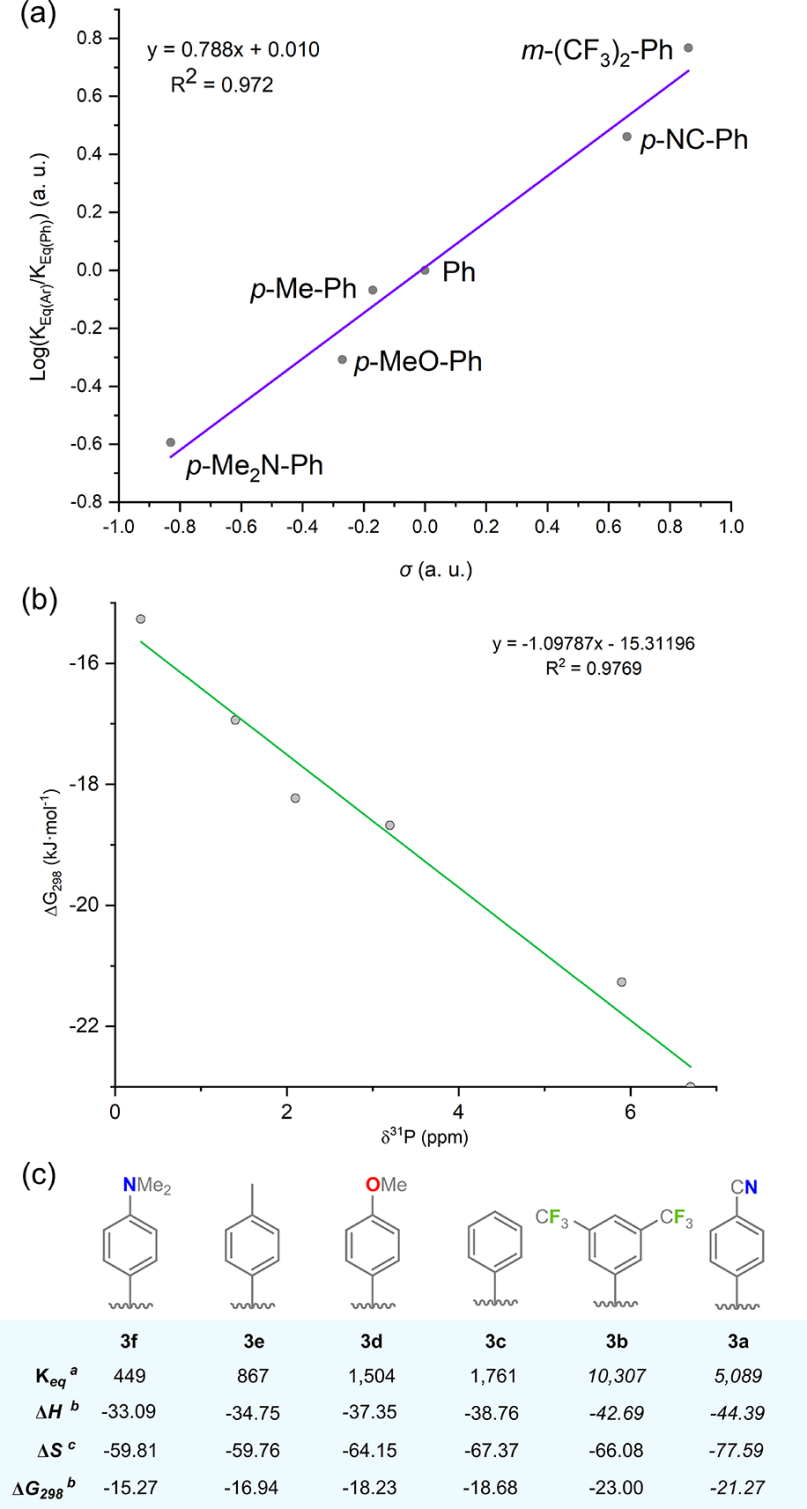
Analysis of the equilibrium for the H_2_ activation in **3a**–**f**; (a) Hammet plot of Log(*K*_eq(Ar)_/*K*_eq(Ph)_) vs. Hammet parameters (σ); (b) Plot of experimental Δ*G*_298_ values vs. ^31^P NMR shift for the “free” germylene ligands **2a–2f**; (c) A summary of experimental thermodynamic parameters for the H_2_ activation reaction in **3a–3f**; ^*a*^ determined by NMR spectroscopy, see Supporting Information for details; ^*b*^ in kJ·mol^–1^; ^*c*^ in J·K^–1^·mol^–1^.

A closer inspection of ^31^P{^1^H} NMR spectra for reactions of complexes **3a–3f** with H_2_ indicates that hydride complexes **4a–4f** are not quantitatively generated under the given conditions (i.e., 1.5 atm of H_2_, 298 K). Further, the ratio of complexes **3** to complexes **4** varies on the basis of the Ge–Ar substituent. Generating a Hammett plot for these equilibria clearly demonstrates that the reaction becomes more favorable for increasingly electron-withdrawing substituents (e.g., *K*_eq_ for **4b**: 10,307 L·mol^–1^; for **4f**: 449 L·mol^–1^; Figure 8a). A *K*_eq_ value of 2129 L·mol^–1^ was found for the activation of D_2_ by **4c** (for H_2_: 1761 L·mol^–1^), giving an inverse kinetic isotope effect (KIE) of 0.83, as is known for the activation of H_2_/D_2_ in TM systems.^34^ The thermodynamics of H_2_ activation by **3a–3f** could be further probed using VT ^31^P{^1^H} NMR spectroscopy (Figure 8a,b). Integration of relative concentrations of complexes **3** and **4** between 293 and 305 K in 2K intervals allowed for the extrapolation of thermodynamic data for all complexes through Van’t Hoff analyses (Tables S2–S9 in SI). Reaction enthalpies become more favorable with a decreasing HOMO–LUMO gap, i.e., correlating with a red shift in experimentally observed UV/vis absorptions. That is, **4a** shows the most exothermic reaction (λ = 799 nm; Δ*H* = −44.39 ± 1.14 kJ·mol^–1^), and **4e** shows the least exothermic reaction (λ = 741 nm; Δ*H* = −33.09 ± 1.20 kJ·mol^–1^). We note that, although **4a** has the largest Δ*H* value, this does not result in the largest *K*_eq_ at 298 K, due to the much higher Δ*S* value of −77.6 J·mol^–1^·K^–1^ for this system, leading to a greater influence of the temperature on *K*_eq_. We hypothesize that this entropic value may be due to the coordinative nature of the *p*-NC-Ph group at Ge in **3a**/**4a**, which could allow for the formation of higher-order complexes for product **4a** in solution, further lowering its entropy. Nevertheless, Δ*G*_298_ values also follow the trend described for enthalpic values.^35^

**Figure 9:**
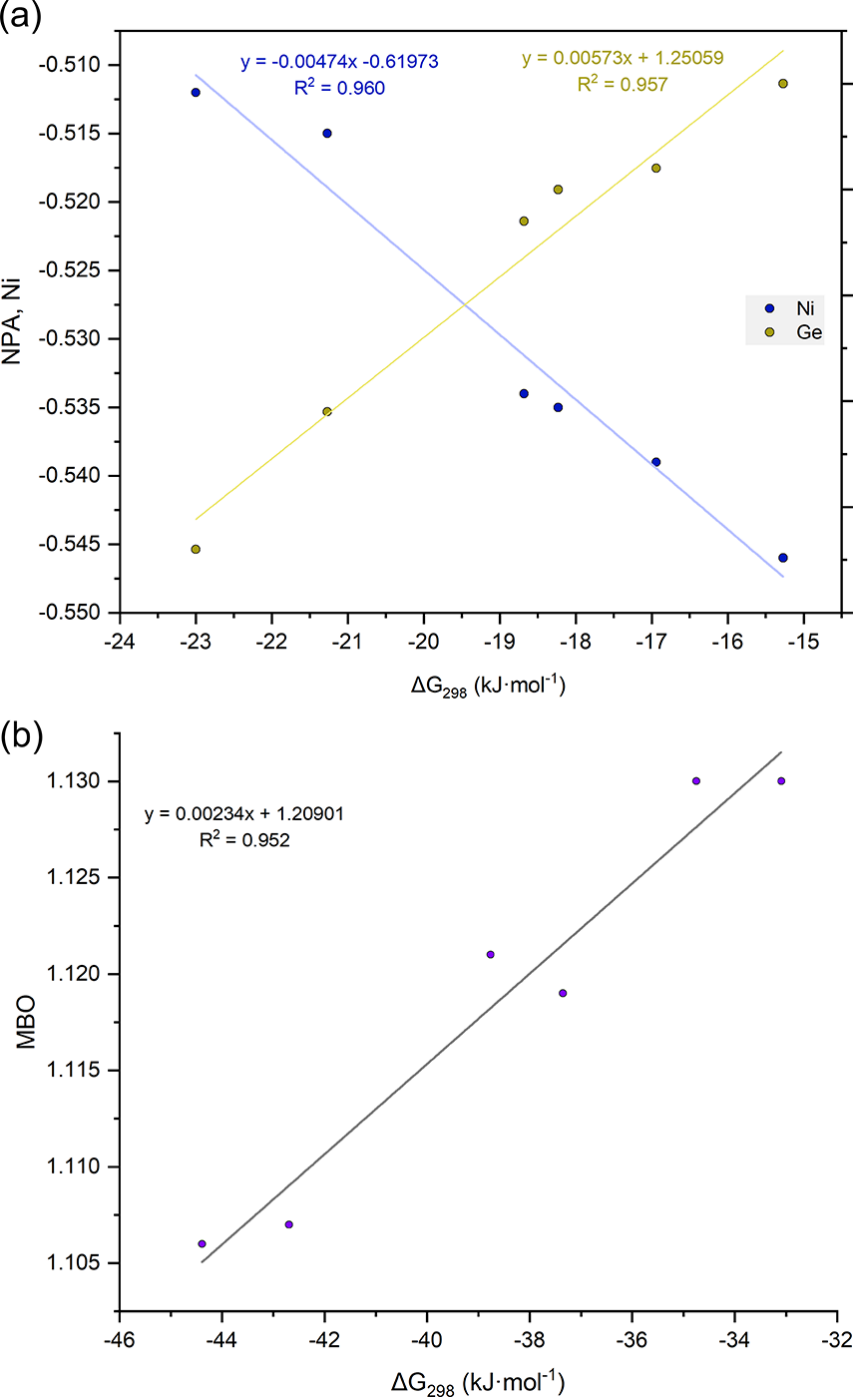
Plots of experimentally determined Δ*G*_298_ values for **4a**–**f** vs calculated (a) Ni/Ge NPA charges, and (b) Ni–Ge Mayer Bond Order (MBO).

Incorporating DFT calculations, plots of Δ*G*_298_ for H_2_ activation by **3a–3f** against related Ge and Ni NPA charges (Figure 9a), as well as against Mayer bond orders for Ge–Ni interactions (Figure 9b) all show a strong correlation. Here, a decrease in negativity at Ni, an increase in negativity at Ge, and a lower MBO all favor H_2_ activation. These data can be used to estimate the Δ*G*_298_ value for bis(amido)germylene complex **3g**, which does not activate H_2_ under the given conditions and would therefore be expected to have an endergonic value for this process. Using the linear regression equation in combination with the calculated NBO for **3g**, a Δ*G*_298_ value of 9.0 kJ·mol^–1^ is found, in line with experimental observations. Although this is lower than the corresponding calculated value for this system (i.e., 21.6 kJ·mol^–1^), the difference between the former Δ*G*_298_ value (i.e., 9.0 kJ·mol^–1^) and that found experimentally for **3c** (−18.7 kJ·mol^–1^; *d*_Δ*G*exp_ = 27.7 kJ·mol^–1^) is similar to the difference in the related *calculated* values for the same systems (*d*_Δ*G*calc_ = 22.8 kJ·mol^–1^). This observation further reinforces the utility of DFT calculations in predictive ligand design. As a whole, these results demonstrate that electronic modulation of easily accessible germylene ligands can be utilized in tuning the thermodynamics of the reversible, cooperative H_2_ activation reaction, employing an abundant 3*d* transition metal. Given the correlation between both experimental and calculated descriptors with thermodynamic values, this opens the door for utilizing high-throughput modeling methodologies (e.g., machine learning) to further define and discover such cooperative bond activation processes, rooted in computational ligand screening.

### Silane Dehydrocoupling

Having a handle on tuning the thermodynamics of a fundamentally important process such as H_2_ activation may have profound implications in catalysis, with ligand design being a cornerstone of catalyst development.^36^ As such, we sought to couple the observed reversible H_2_ activation systems to a dehydrocoupling process. For this, we focused our efforts on the dehydrocoupling of phenyl silane, given that this relies on H_2_ elimination, and is known for low-valent nickel systems.

**Scheme 2:**
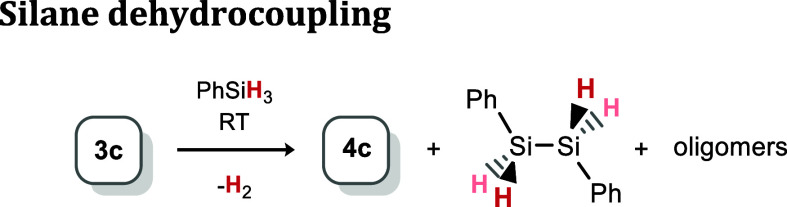
Formation of **4c** through the Dehydrocoupling of PhSiH_3_ by **3c**

In an initial reaction, a slight excess of PhSiH_3_ was added to **3c**, which was seen to generate the dihydride complex **4c**, [Ph(H)_2_Si]_2_, and H_2_ (Scheme 2). From these reaction mixtures, analytically pure **4c** precipitates from the solution and is readily isolated in up to 77% yield.

**Figure 10:**
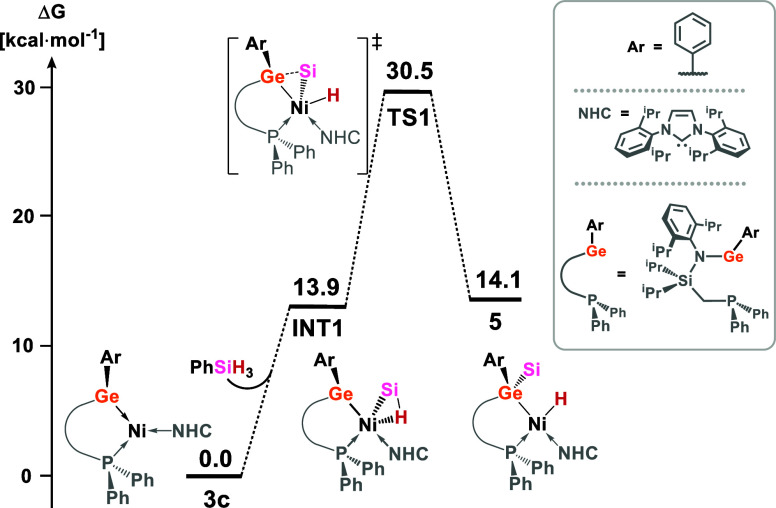
DFT-derived mechanism for the cooperative activation of PhSiH_3_ by **3c**.

We then sought to ascertain whether the initial activation of phenyl silane proceeds via a similar mechanism to dihydrogen activation, that is, involving both the Ni and Ge centers. The most feasible calculated mechanism for this process is given in Figure 10. We find that the initial coordination of the Si–H bond in PhSiH_3_ occurs at the Ni center in a barrierless step (INT1, 13.9 kcal·mol^–1^). The scission of the Si–H linkage occurs with the help of the Ge center, through a Ge···Si bridge (TS1, 30.5 kcal·mol^–1^), resulting in **5** with a [Ge(Si)-Ni(H)] core (14.1 kcal·mol^–1^), which is favored over the corresponding Ge-*H* intermediated (17.5 kcal·mol^–1^; Figure S153 in SI). This latter point is interesting, given that the Ge center is more electropositive than Ni and thus may be expected to be a stronger electrophile in this case.

Extending the described stoichiometric activation of phenyl silane, we found that (aryl)germylene-Ni^0^ complexes **3a**–**3f** are capable of catalyzing the dehydrocoupling of PhSiH_3_, akin to earlier reported Ni complexes (Table 2).^37^ Using a 2.5 mol % catalytic loading of **3c,** full consumption of PhSiH_3_ is observed after 48 h, with the visible generation of H_2_ in reaction mixtures. Here, oligo-silanes can be observed in ^1^H NMR spectra of reaction mixtures.^37^ GPC analysis of polymers isolated from these reactions yielded Mw values of between 795 and 1052 g·mol^–1^, with average PDIs of 1.67 (Figures S129–S131 in SI). A key point here is the observation of **4c** in stoichiometric reaction mixtures, which would suggest that the loss of H_2_ to regenerate **3c** is rate-determining. It follows that more endergonic H_2_ activation values should favor the dehydrocoupling reaction. That is, **3f** should be the most active catalyst, and **3a** the least. Comparing these systems demonstrated that this is indeed the case: dehydrocoupling of PhSiH_3_ with 1 mol % of complexes **3a**–**f** at ambient temperature for 3 h led to 48% conversion for **3b** (Δ*G*_298_ = −23.00 kJ·mol^–1^), in contrast to 64% for **3f** (Δ*G*_298_ = −15.27 kJ·mol^–1^). Although being a small data set, this gives early evidence that the electronic modulation in these complexes may allow for catalyst fine-tuning in further key synthetic processes. In this regard, we continue to investigate the stoichiometric silane activation using these and related complexes, to further elucidate key mechanistic aspects and allow for further catalyst improvements.

**Table 2:**
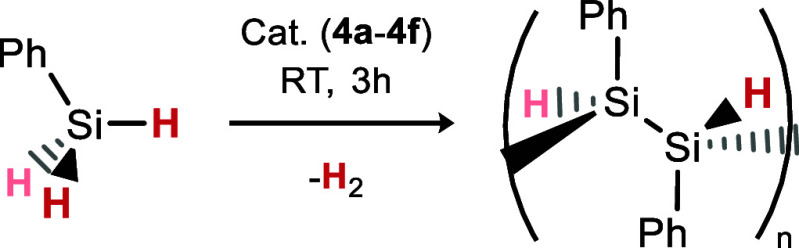
Catalytic Phenylsilane Dehydrocoupling Catalyzed by Complexes **3a**–**3g**

Ni^0^ complex	catalyst loading (mol %)	consumption of PhSiH_3_, %(yield, %)^a^^,^^b^	*M*_w_^b^	PDI^b^
4c^c^	2.5	>99 (93)	948	1.67
4a	1	48		
4b	1	57		
4c	1	62		
4d	1	60		
4e	1	62		
4f	1	64		

a Determined by relative integration of Si-*H* peaks vs an internal standard (see the SI for details).

b Values given are averaged from three runs.

c This experiment was run for 48 h to ensure full consumption of PhSiH_3_.

## Conclusions

In summary, we present a novel family of (amido)(aryl)germylene ligands **2**, which are employed in the synthesis of 16-electron Ni^0^ complexes **3**. All systems are capable of reversible activation of dihydrogen, leading to 1,2-dihydride complexes (**4**) featuring HGe-NiH cores. Van’t Hoff analyses of equilibria formed upon addition of H_2_ to **3** allow for the accurate extrapolation of thermodynamic data for this activation process, showing a strong correlation with the electronic nature of the germylene ligands: generally, more electron-withdrawing systems favor the H_2_ activation reaction. This is further supported by computational investigations, allowing for the identification of calculable descriptors that correlate with experimental data in linear regression analyses. These observations could then be applied in a catalytic context: all Ge^II^–Ni^0^ complexes demonstrate the ability to catalyze the dehydrocoupling of phenyl silane. As the rate-limiting step in this process is the loss of H_2_, we have shown that catalysts that disfavor H_2_ activation lead to a more rapid dehydrocoupling reaction. Taken as a whole, this study further establishes tetrylenes as readily tunable noninnocent ligands, giving key insights into the effects of ligand modifications on modulating both stoichiometric and catalytic processes. Ligand modification has a predicable effect on catalyst performance, having exciting implications for the broader use of low-valent *p*-block ligands in catalytic systems. Taking this forward, we now aim to develop models for computationally exploring effective tetrylene-TM complexes as catalysts for hydrogen-centric processes.

## Supporting Information Available

Synthetic and characterization data for all new compounds, including spectra; details of kinetic and thermodynamic studies; and full details and references for the crystallographic and computational data (PDF)

## References

[ref1] aKubas G. J. Molecular hydrogen complexes: coordination of a σ-bond to transition metals. Acc. Chem. Res. 1988, 21, 120–128. 10.1021/ar00147a005.

[ref2] aNichimura S., Ed. Handbook of Heterogeneous Catalytic Hydrogenation for Organic Synthesis; Wiley-Interscience, 2001;.

[ref3] aPreuster P.; Papp C.; Wasserscheid P. Liquid Organic Hydrogen Carriers (LOHCs): Toward a Hydrogen-free Hydrogen Economy. Acc. Chem. Res. 2017, 50, 74–85. 10.1021/acs.accounts.6b00474. 28004916

[ref4] aRayhan U.; Kowser Z.; Islam N.; Redshaw C.; Yamato T. Top. Catal. 2018, 61, 560–574. 10.1007/s11244-018-0994-2.

[ref5] aLiu X.; Astruc D. Development of the Applications of Palladium on Charcoal in Organic Synthesis. Adv. Synth. Catal. 2018, 360, 3426–3459. 10.1002/adsc.201800343.

[ref6] Kurosawa H.; Yamamoto A., Eds. Fundamentals of Molecular Catalysis; Elsevier Science, 2003.

[ref7] aLudwig J. R.; Schindler C. S. Catalyst: Sustainable Catalysis. Chem. 2017, 2, 313–316. 10.1016/j.chempr.2017.02.014.

[ref8] aFilonenko G. A.; Putten R. V.; Hensen E. J. M.; Pidko E. A. Catalytic (de)hydrogenation promoted by non-precious metals – Co, Fe and Mn: recent advances in an emerging field. Chem. Soc. Rev. 2018, 47, 1459–1483. 10.1039/C7CS00334J. 29334388

[ref9] aLyaskovskyy V.; de Bruin B. Redox Non-Innocent Ligands: Versatile New Tools to Control Catalytic Reactions. ACS Catal. 2012, 2, 270–279. 10.1021/cs200660v.

[ref10] For some examples, see: aHarman W. H.; Peters J. C. Reversible H2 Addition across a Nickel–Borane Unit as a Promising Strategy for Catalysis. 10.1021/ja211419t 22380492

[ref11] aDötz K. H. Metal Carbenes in Organic Synthesis; Springer, 2004;.

[ref12] aDzik W. I.; Zhang X. P.; de Bruin B. Redox Noninnocence of Carbene Ligands: Carbene Radicals in (Catalytic) C-C Bond Formation. Inorg. Chem. 2011, 50, 9896–9903. 10.1021/ic200043a. 21520926

[ref13] aGutsulyak D. V.; Piers W. E.; Borau-Garcia J.; Parvez M. Activation of Water, Ammonia, and Other Small Molecules by PCcarbeneP Nickel Pincer Complexes. J. Am. Chem. Soc. 2013, 135, 11776–11779. 10.1021/ja406742n. 23906261

[ref14] aDauth A.; Gellrich U.; Diskin-Posner Y.; Ben-David Y.; Milstein D. The Ferraquinone–Ferrahydroquinone Couple: Combining Quinonic and Metal-Based Reactivity. J. Am. Chem. Soc. 2017, 139, 2799–2807. 10.1021/jacs.6b13050. 28141925 PMC5330656

[ref15] aSmith J. D.; Durrant G.; Ess D. H.; Gelfand B. S.; Piers W. E. Chem. Sci. 2020, 11, 10705–10717. 10.1039/D0SC02694H. 34094323 PMC8162389

[ref16] aCammarota R. C.; Xie J.; Burgess S. A.; Vollmer M. V.; Vogiatzis K. D.; Ye J.; Linehan J. C.; Appel A. M.; Hoffmann C.; Wang X.; Young V. G. Jr.; Lu C. C. Thermodynamic and kinetic studies of H_2_ and N_2_ binding to bimetallic nickel-group 13 complexes and neutron structure of a Ni(η_2_-H_2_) adduct. Chem. Sci. 2019, 10, 7029–7042. 10.1039/C9SC02018G. 31588270 PMC6676469

[ref17] aHadlington T. J.; Szilvási T.; Driess M. Silylene–Nickel Promoted Cleavage of B-O Bonds: From Catechol Borane to the Hydroborylene Ligand. Angew. Chem., Int. Ed. 2017, 56, 7470–7474. 10.1002/anie.201702772. 28481013

[ref18] Litz K. E.; Henderson K.; Gourley R. W.; Holl M. M. B. Reversible Insertion Reactions of a Platinum Germylene Complex. Organometallics 1995, 14, 5008–5010. 10.1021/om00011a015.

[ref19] aPu L.; Olmstead M. M.; Power P. P.; Schiemenz B. Synthesis and Characterization of the Monomeric Terphenyl–Metal Halides Ge(Cl){C_6_H_3_-2,6-Trip_2_} (Trip = C_6_H_2_-2,4,6-*i*-Pr_3_) and Sn(I){C_6_H_3_-2,6-Trip_2_} and the Terphenyl–Metal Amide Sn{N(SiMe_3_)_2_}{C_6_H_3_-2,6-Trip_2_}. Organometallics 1998, 17 (26), 5602–5606. 10.1021/om980697l.

[ref20] aProtchenko A. V.; Bates J. I.; Saleh L. M. A.; Blake M. P.; Schwarz A. D.; Kolychev E. L.; Thompson A. L.; Jones C.; Mountford P.; Aldridge S. Enabling and Probing Oxidative Addition and Reductive Eliminationat a Group 14 Metal Center: Cleavage and Functionalization of E–H Bonds by a Bis(boryl)stannylene. J. Am. Chem. Soc. 2016, 138, 4555–4564. 10.1021/jacs.6b00710. 26981766

[ref21] aKeil P. M.; Szilvási T.; Hadlington T. J. Reversible metathesis of ammonia in an acyclic germylene–Ni^0^ complex. Chem. Sci. 2021, 12, 5582–5590. 10.1039/D1SC00450F. 34168794 PMC8179610

[ref22] We have also extended this ligand class to P-modifications, specifically using Cy- and ^i^Pr-substituents at P, and the Ph-substituent at Ge. These ligands do not react with Ni^0^ fragments described here, and as such have not been included in this study. The synthesis and analytical data for the Cy-derivative are included in the ESI, as ^**CyiP**^**DippGePh**.

[ref23] Schulz A.; Kalkuhl T. L.; Keil P. M.; Hadlington T. J. T-shaped Ni^0^ Systems Featuring Cationic Tetrylenes: Direct Observation of L/Z-type Ligand Duality, and Alkene Hydrogenation Catalysis. Angew. Chem., Int. Ed. 2023, 62, e202305996 10.1002/anie.202305996. 37195749

[ref24] We regard these complexes as Ge^II^-Ni^0^ complexes, largely based on literature formalisms. Given the significant degree of donor-acceptor interaction, we are presently working to better establish the ‘true’ oxidation state of these centres through XPS and EXAFS methods.

[ref25] Brookhart M.; Green M. L. H.; Parkin G. Agostic interactions in transition metal compounds. Proc. Natl. Acad. Sci. U.S.A. 2007, 104, 6908–6914. 10.1073/pnas.0610747104. 17442749 PMC1855361

[ref26] aHerrmann W. A.; Denk M.; Behm J.; Scherer W.; Klingan F.-R.; Bock H.; Solouki B.; Wagner M. Stable Cyclic Germanediyls (“Cyclogermylenes”): Synthesis, Structure, Metal Complexes, and Thermolyses. Angew. Chem., Int. Ed. Engl. 1992, 31, 1485–1488. 10.1002/anie.199214851.

[ref27] Bender J. E.; Shusterman A. J.; Banaszak Holl M. M.; Kampf J. W. X-ray Crystallographic and Theoretical Comparison of Ge[2,4,6-(CF_3_)_3_C_6_H_2_]_2_ and Ge[N(SiMe_3_)_2_]_2_as Ligands in (Ph_3_P)_2_NiGeX_2_ Complexes. Organometallics 1999, 18, 1547–1552. 10.1021/om9900521.

[ref28] Lai T. Y.; Guo J.-D.; Fettinger J. C.; Nagase S.; Power P. P. Facile insertion of ethylene into a group 14 element-carbon bond: effects of the HOMO–LUMO energy gap on reactivity. Chem. Commun. 2019, 55, 405–407. 10.1039/C8CC08488B. 30542688

[ref29] We note that our previously reported chloro-germylene complex [^PhiP^Dipp(Cl)Ge·Ni·IPr] (see ref. (23)) also reversibly activates H_2_ and D_2_ (Figs. S106-S111 in ESI). However, two species are formed, which we believe to be due to H/Cl exchange at Ni, leading to a dihydrogermyl complex (*i.e.* [{^PhiP^Dipp(H)_2_GeNi(Cl)}·IPr]). This has been observed, albeit not reversibly, by Kato et al. in a related chloro-silylene complex (see ref. (17a)). From these mixtures, only the dimeric Ni^I^ hydride complex [{^PhiP^Dipp(Cl)Ge·Ni(H)}_2_] could be identified crystallographically (Fig. S137 in ESI).

[ref30] For examples, see: Verhoeven D. G. A.; Orsino A. F.; Bienenmann R. L. M.; Lutz M.; Moret M.-E. Cooperative Si–H Addition to Side-On Ni(0)-Imine Complexes Forms Reactive Hydrosilazane Complexes. Organometallics 2020, 39, 623–629. 10.1021/acs.organomet.0c00059.

[ref31] Schmidt D.; Zell T.; Schaub T.; Radius U. Si–H bond activation at {(NHC)_2_Ni^0^ } leading to hydrido silyl and bis(silyl) complexes: a versatile tool for catalytic Si–H/D exchange, acceptorless dehydrogenative coupling of hydrosilanes, and hydrogenation of disilanes to hydrosilanes. Dalton Trans. 2014, 43, 10816–10827. 10.1039/c4dt01250j. 24894607

[ref32] We would like to add two notes here: (i) addition of *N*-bases to solutions of **3a–f** does not prevent the activation of H_2_ by these systems, which might suggest that such bases do not bind either Ge or Ni centres in these complexes. And (ii) the addition of H_2_ to **3g** does not lead to any changes in the ^1^H or ^31^P NMR spectra, suggesting that no H_2_ complex is formed.

[ref33] Storage of **3b** under an atmosphere of H_2_ for 3 weeks led to the isolation of a few crystals of [^PhiP^Dipp{(CF_3_)_2_Ph}_2_Ge]Ni(H)IPr, presumably through rearrangement of 1,2-dihydride **4b**. This compound was crystallographically characterized (Fig. S138 in ESI), but no further data could be collected due to the very low yield of this complex.

[ref34] See the following, and references therein: Bender B. R.; Kubas G. J.; Jones L. H.; Swanson B. I.; Eckert J.; Capps K. B.; Hoff C. D. Why Does D_2_ Bind Better Than H_2_? A Theoretical and Experimental Study of the Equilibrium Isotope Effect on H_2_ Binding in a M(η^2^-H_2_) Complex. Normal Coordinate Analysis of W(CO)_3_(PCy_3_)_2_(η^2^-H_2_). J. Am. Chem. Soc. 1997, 119, 9179–9190. 10.1021/ja971009c.

[ref35] We note that, although calculated ΔG values are higher than those observed experimentally, these are well within the expected error for DFT calculations of ∼5 cal·mol^–1^.

[ref36] Stradiotto M.; Lundgren R. J. Ligand Design in Metal Chemistry: Reactivity and Catalysis; John Wiley & Sons, Ltd., 2016.

[ref37] aFontaine F.-G.; Zargarian D. Dehydrogenative Oligomerization of PhSiH_3_ Catalyzed by (1-Me-Indenyl)Ni(PR_3_)(Me). Organometallics 2002, 21, 401–408. 10.1021/om010757e.

