# Supplementary material for: Thermodynamic Modulation of Dihydrogen Activation Through Rational Ligand Design in GeII–Ni0 Complexes
Source: J Am Chem Soc. 2024 Aug 6;146(33):23606–15. doi: 10.1021/jacs.4c08297 (PMC11345810; doi:10.1021/jacs.4c08297)
Supplement: Supplementary file 1 — ja4c08297_si_001.pdf [file ja4c08297_si_001.pdf]

Supporting Information

Thermodynamic modulation of dihydrogen activation through rational ligand design in Ge^{II}-Ni^{0} complexes

Philip M. Keil,[a] Sophia Ezendu,[b] Annika Schulz,[a] Malte Kubisz,[a] Tibor Szilvási,[b,*] and Terrance J. Hadlington[a,*]

[a] Lehrstuhl für anorganische Chemie mit Schwerpunkt neue Materialien, Technische Universität München, Lichtenbergstraße 4, 85747 Garching, Germany

[b] Department of Chemical and Biological Engineering, University of Alabama, Tuscaloosa, AL 35487, United States.

1. **Experimental methods and data……………………..S2**
   - General Considerations……………………………………….S2
   - Synthetic details and data……………………………..…S2
   - NMR, MS, UV/vis, and IR spectra……………………….....S13
   - Kinetic and Thermodynamic Analyses…………………..…S69
   - Details of dehydrocoupling catalyses………………………S78
2. **X-ray crystallographic details…………………….….S90**
3. **Computational methods and details………………..…S100**
4. **References…………………………………………….....S388**

1. Experimental methods and data

General considerations. All experiments and manipulations were carried out under dry oxygen free argon atmosphere using standard Schlenk techniques or in a MBraun inert atmosphere glovebox containing an atmosphere of high purity argon. C_6D_6 was dried, degassed and stored over a potassium mirror. THF and Et_2O were dried over Na/Benzephenone, distilled and stored over 4Å molecular sieves. All other solvents were dried over activated 4Å molecular sieves and thoroughly degassed before use. **1** ^{PhiP}DippGeCl,[1] 4-Me-C_6H_4-Li,[2] 4-OMe-C_6H_4-Li,[3] 4-NMe_2-C_6H_4-Li,[4] [^{PhiP}Dipp(Cl)Ge·Ni·IPr][5], $Ni(COD)_2$,[6] and IPr[7] were synthesized using reported procedures. All other reagents were used as received. NMR spectra were recorded on a Bruker AV 400 Spectrometer. ^{1}H and $^{13}C\{^{1}H\}$ NMR spectra were referenced to the residual solvent signals as internal standards. $^{29}Si\{^{1}H\}$ NMR spectra were externally calibrated with $SiMe_4$. $^{31}P\{^{1}H\}$ NMR spectra were externally calibrated with H_3PO_4. Liquid Injection Field Desorption Ionization Mass Spectrometry (LIFDI-MS) was measured directly from an inert atmosphere glovebox with a Thermo Fisher Scientific Exactive Plus Orbitrap equipped with an ion source from Linden CMS.[8] Commercial hydrogen gas with a purity of ≥99.999% was used in all hydrogenation experiments. Elemental analyses (C, H, N) were performed with a combustion analyzer (elementar vario EL, Bruker). Infrared spectra were measured with the Alpha FT-IR from Bruker containing a platinum diamond ATR device. Absorption spectra (UV/vis) were recorded on an Agilent Cary 60 UV/vis spectrophotometer. GPC was carried out on a Varian PL GPC-50 equipped with two PL Polargel columns. As eluent, THF stabilized with 250 ppm BHT was used. Absolute molecular weights have been determined by two angle laser light scattering analysis using a concentration and viscosity detector, coupled with GPC.

Synthetic details and data

General Method for preparation of ^{PhiP}DippGe(R), 2.

A solution of freshly prepared Grignard or Lithium reagent was added dropwise to a stirred solution of **1** in toluene at -78 °C. The mixture was then allowed to warm to ambient temperature and stirred for 30 min. All volatiles were subsequently removed *in vacuo* and the residue was extracted with toluene. All volatiles were again removed *in vacuo* and the residue dissolved in pentane, and stored at -32 °C overnight. The resulting suspension was filtered and the solid washed with pentane and dried *in vacuo*, yielding the substituted germylene **2**. If no solid formed after storing the pentane solution at -32 °C it was used without further purification in subsequent steps.

^{PhiP}Dipp[*m*-$(CF_3)_2$-Ph]Ge, 2b. The germylene was synthesized according to the general method using **1** (1.80 g, 3.0 mmol) and a freshly prepared Et_2O solution of *m*-$(CF_3)_2$-PhMgBr using *m*-$(CF_3)_2$-PhBr (1.15 g, 3.9 mmol), Mg (0.14 g, 5.9 mmol), and a crystal of iodide. An off-white powder of **2b** (1.26 g, mmol, 54 %) was isolated after precipitation from a pentane solution at -32°C. Colorless crystals

suitable for X-ray diffraction analysis were obtained from a concentrated pentane solution, which was stored at ambient temperature for 14 days.

^{1}H NMR (C_6D_6, 400 MHz, 298 K): δ = 0.73 (d, 3H, $^3J_{HH}$ = 6.8 Hz, Si-^{i}PrC*H₃*), 0.82 (m, 1H, Si-^{i}PrC*H*), 0.92 (m, 3H, $^3J_{HH}$ = 7.1 Hz, Si-^{i}PrCH_3), 1.00 (d, 3H, $^3J_{HH}$ = 6.7 Hz, Dipp-^{i}PrCH_3), 1.05 (d, 3H, $^3J_{HH}$ = 6.7 Hz, Dipp-^{i}PrCH_3), 1.26 (m, 6H, Dipp-^{i}PrCH_3/Si-^{i}PrCH_3), 1.44 (d, 3H, $^3J_{HH}$ = 7.4 Hz, Si-^{i}PrCH_3), 1.48 (d, 3H, $^3J_{HH}$ = 6.9 Hz, Dipp-^{i}PrCH_3), 1.70 (m, 3H, CH_2-PPh$_2$/Si-^{i}PrC*H*), 3.07 (sept, 1H, $^3J_{HH}$ = 6.7 Hz, Dipp-^{i}PrC*H*), 4.54 (sept, 1H, $^3J_{HH}$ = 6.7 Hz, Dipp-^{i}PrC*H*), 6.61 (m, 2H, Ar-C*H*), 6.75 (m, 2H, Ar-C*H*), 6.88 (m, 1H, Ar-C*H*), 7.12 (m, 7H, Ar-C*H*), 7.80 (m, 4H, Ar-C*H*), 7.65 (s, 1H, Ge-Ar$_{CF3}$-H_{para}), 7.71 (m, 2H, Ar-C*H*), 8.11 (s, 2H, Ge-Ar$_{CF3}$-H_{ortho}).

^{31}P{^{1}H} NMR (C_6D_6, 162 MHz, 298 K): δ = 6.7 (s, *P*Ph$_2$).

^{13}C{^{1}H} NMR (C_6D_6, 101 MHz, 298 K): δ = 7.7 (*C*H$_2$-PPh$_2$), 17.4 (d, Si-^{i}Pr*C*H), 19.1 and 19.9 (Si-^{i}Pr*C*H$_3$), 20.3 (d, Si-^{i}Pr*C*H), 20.7 and 20.9 (Si-^{i}Pr*C*H$_3$), 22.3 and 23.2 (Dipp-^{i}PrCH_3), 27.7 (Dipp-^{i}PrC*H*), 28.3 (Dipp-^{i}PrCH_3), 28.7 (Dipp-^{i}PrC*H*), 28.8 (Dipp-^{i}PrCH_3), 120.0, 123.4, 124.7, 124.8, 126.1, 128.8, 129.1, 129.2, 129.5, 129.6, 129.8, 130.0, 130.3, 130.5, 130.6, 131.9, 131.9, 134.1, 134.2, 145.3, 145.4, 146.9, 146.9, 148.4, 148.4 and 163.0 (Ar-*C*).

^{29}Si{^{1}H} NMR (C_6D_6, 99 MHz, 298 K): δ = 12.6 (d, $^2J_{SiP}$ = 13.7 Hz, CH$_2$-*Si*-^{i}Pr$_2$).

MS/LIFDI-HRMS found (calcd.) m/z: 775.2257 (775.2253) for $[M]^+$.

Anal. calcd. for $C_{39}H_{46}F_6GeNPSi$: C, 60.48%; H, 5.99%; N, 1.81%; found: C, 58.30%; H, 5.66%; N, 1.81%.

^{PhiP}Dipp(Ph)Ge, 2c. The germylene was synthesized according to the general method using **1** (2.00 g, 3.4 mmol), and a freshly prepared Et_2O solution of PhMgBr using PhBr (0.68 g, 4.4 mmol), Mg (0.16 g, 6.5 mmol), and a crystal of iodide. An off-white powder of **2c** (1.35 g, 2.1 mmol, 63%) was isolated after precipitation from a pentane solution at -32°C.

^{1}H NMR (C_6D_6, 400 MHz, 298 K): δ = 0.81 (m, 3H, Si-^{i}PrCH_3), 0.87 (m, 1H, Si-^{i}PrC*H*), 1.04 (m, 9H, Si-^{i}PrCH_3/Dipp-^{i}PrCH_3), 1.28 (d, 3H, $^3J_{HH}$ = 7.6 Hz, Si-^{i}PrCH_3), 1.37 (d, 3H, $^3J_{HH}$ = 6.4 Hz, Dipp-^{i}PrCH_3), 1.42 (d, 3H, $^3J_{HH}$ = 7.3 Hz, Si-^{i}PrCH_3), 1.47 (d, 3H, $^3J_{HH}$ = 6.7 Hz, Dipp-^{i}PrCH_3), 1.88 (m, 3H, CH_2-PPh$_2$/Si-^{i}PrC*H*), 3.25 (sept, 1H, $^3J_{HH}$ = 6.5 Hz, Dipp-^{i}PrC*H*), 4.51 (sept, 1H, $^3J_{HH}$ = 6.5 Hz, Dipp-^{i}PrC*H*), 6.78 (m, 5H, Ar-C*H*), 7.13 (m, 9H, Ar-C*H*), 7.80 (m, 4H, Ar-C*H*).

^{31}P{^{1}H} NMR (C_6D_6, 162 MHz, 298 K): δ = 3.2 (s, *P*Ph$_2$).

^{13}C{^{1}H} NMR (C_6D_6, 101 MHz, 298 K): δ = 8.3 (d, $^1J_{CP}$ = 7.6 Hz, *C*H$_2$-PPh$_2$), 17.6 (d, Si-^{i}Pr*C*H), 19.6 and 20.3 (Si-^{i}Pr*C*H$_3$), 20.6 (d, Si-^{i}Pr*C*H), 21.1 and 21.2 (Si-^{i}Pr*C*H$_3$), 22.6 and 23.2 (Dipp-^{i}PrCH_3), 28.2 (Dipp-^{i}PrC*H*), 28.3 (Dipp-^{i}PrCH_3), 28.6 (Dipp-^{i}PrC*H*), 28.8 (Dipp-^{i}PrCH_3), 123.2, 124.3, 124.5, 127.6, 129.0, 131.4, 131.5, 131.5, 134.2, 134.3, 134.4, 134.5 and 148.7 (Ar-*C*).

^{29}Si{^{1}H} NMR (C_6D_6, 99 MHz, 298 K): δ = 9.5 (d, $^{2}J_{SiP}$ = 13.8 Hz, CH_2-*Si*-$^{i}Pr_2$).

MS/LIFDI-HRMS found (calcd.) m/z: 639.2507 (639.2505) for $[M]^+$.

Anal. calcd. for $C_{37}H_{48}GeNPSi$: C, 69.60%; H, 7.58%; N, 2.19%; found: C, 66.48%; H, 7.48%; N, 2.21%.

^{PhiP}Dipp(*p*-NC-Ph)Ge, 2a. The germylene was synthesized according to the general method using **1** (1.0 g, 1.68 mmol) and a freshly prepared THF solution of *p*-NC-PhLi using 4-CN-PhBr (350 mg, 1.92 mmol) and n-BuLi (2.5 M in hexane, 1.54 mL, 1.92 mmol). Because no precipitation of **2c** was observed after storing the crude pentane solution at -32°C overnight the solution was used without further purification assuming full conversion due to the ^{31}P NMR.

^{31}P{^{1}H} NMR (toluene, 162 MHz, 298 K): δ = 5.9 (s, *P*Ph_2).

^{PhiP}Dipp(*p*-Me-Ph)Ge, 2d. The germylene was synthesized according to the general method using **1** (250 mg, 0.84 mmol) and a freshly prepared Et_2O solution of 4-Me-PhLi (131 mg, 1.34 mmol). Because no precipitation of **2d** was observed after storing the crude pentane solution at -32°C overnight the solution was used without further purification assuming full conversion due to the ^{31}P NMR.

^{31}P{^{1}H} NMR (toluene, 162 MHz, 298 K): δ = 2.1 (s, *P*Ph_2).

^{PhiP}Dipp(*p*-OMe-Ph)Ge, 2e. The germylene was synthesized according to the general method using **1** (500 mg, 0.84 mmol) and a freshly prepared Et_2O solution of 4-OMe-PhLi (153 mg, 1.34 mmol). Because no precipitation of **2e** was observed after storing the crude pentane solution at -32°C overnight the solution was used without further purification assuming full conversion due to the ^{31}P NMR.

^{31}P{^{1}H} NMR (toluene, 162 MHz, 298 K): δ = 1.4 (s, *P*Ph_2).

^{PhiP}Dipp(*p*-Me_2N-Ph)Ge, 2f. The germylene was synthesized according to the general method using ^{PhiP}DippGeCl (250 mg, 0.42 mmol) and a freshly prepared Et_2O solution of 4-NMe_2-PhLi (85 mg, 0.67 mmol). Because no precipitation of **2f** was observed after storing the crude pentane solution at -32°C overnight the solution was used without further purification assuming full conversion due to the ^{31}P NMR.

^{31}P{^{1}H} NMR (C_6D_6, 162 MHz, 298 K): δ = 0.3 (s, *P*Ph_2).

^{PhiP}Dipp(Me$_2$N)Ge, 2g. The germylene was synthesized according to the general method using **1** (250 mg, 0.42 mmol) and a freshly prepared solution of NMe$_2$Li using NHMe2 (1M in THF, 0.42 mL, 0.42 mmol) and n-BuLi (2.5M in hexane, 0.17 ml, 0.42 mmol) in Et$_2$O. Because no precipitation of **2g** was observed after storing the crude pentane solution at -32°C overnight the solution was used without further purification assuming full conversion due to the ^{31}P NMR.

^{31}P{^{1}H} NMR (toluene, 162 MHz, 298 K): δ = -24.4 (s, *P*Ph$_2$).

^{CyiP}Dipp(Ph)Ge. The germylene was synthesized according to the general method, using ^{CyiP}DippGeCl (3.0 g, 4.9 mmol), and a freshly prepared Et$_2$O solution of PhMgBr using PhBr (1.0 g, 6.4 mmol), Mg (0.24 g, 6.5 mmol), and a crystal of iodide. The target compound was isolated by extraction of the crude mixture with toluene, filtration, and washing with pentane (2 x 5 mL), leading to an analytically pure off-white solid (2.2 g, 72%). Crystals suitable for SCXRD analysis were obtained by dissolving the solid in diethyl ether and storing the concentrated solution at RT overnight.

^{1}H NMR (C$_6$D$_6$, 400 MHz, 298 K): δ = 0.64-0.80 (4H, Cy-C*H*), 0.84 (d, 3H, $^3J_{HH}$ = 6.6 Hz, Dipp-^{i}Pr-C*H*$_3$), 0.89 (d, 3H, $^3J_{HH}$ = 7.5 Hz, Dipp-^{i}Pr-C*H*$_3$), 1.00-1.06 (m, 1H, Cy-*H*), 1.10 (d, 3H, $^3J_{HH}$ = 7.6 Hz, Si-^{i}Pr-C*H*$_3$), 1.14-1.20 (m, 3H, Cy-C*H*), 1.23-1.32 (m, 6H, Cy-C*H*, Si-^{i}Pr-C*H*, C*H*$_2$-PCy$_2$), 1.33-1.38 (m, 10H, Cy-C*H*), 1.41 (d, 3H, $^3J_{HH}$ = 7.0 Hz,), 1.49 (d, 3H, $^3J_{HH}$ = 6.8 Hz Dipp-^{i}Pr-C*H*$_3$, Dipp-^{i}Pr-C*H*$_3$), 1.55-1.59 (m, 2H, Cy-C*H*), 1.65 (d, 3H, $^3J_{HH}$ = 6.6 Hz, Dipp-^{i}Pr-C*H*$_3$), 1.69-1.83 (m, 4H, Cy-C*H*, Si-^{i}Pr-C*H*), 1.94-2.02 (1H, Cy-C*H*), 2.50-2.55 (1H, Cy-C*H*), 3.71 (hept, 1H, $^3J_{HH}$ = 6.9 Hz, Dipp-^{i}Pr-C*H*$_3$), 4.31 (hept, 1H, $^3J_{HH}$ = 6.8 Hz, Dipp-^{i}Pr-C*H*$_3$), 7.15 (s, 2H, Ar-*H*), 7.18-7.24 (m, 2H, Ar-*H*), 7.32 (t, 2H, $^3J_{HH}$ = 7.4 Hz, Ar-*H*), 8.05-8.07 (m, 2H, Ar-*H*).

^{13}C{^{1}H} NMR (C$_6$D$_6$, 101 MHz, 298 K): δ = 2.23 (d, $^1J_{CP}$ = 5.9 Hz, Cy$_2$-P-*C*H$_2$), 18.6 (d, $^1J_{CP}$ = 3.7 Hz, Cy-*C*H), 19.8 (Si-^{i}Pr-*C*H$_3$), 20.7 (Si-^{i}Pr-*C*H$_3$), 20.8 (d, $^2J_{CP}$ = 2.9 Hz, Cy-*C*H), 21.2 (d, $^2J_{CP}$ = 2.9 Hz Cy-*C*H), 22.7 (Dipp-^{i}Pr-*C*H$_3$), 23.9 (Dipp-^{i}Pr-*C*H$_3$), 26.1 (Si-^{i}Pr-*C*H), 26.5 (Si-^{i}Pr-*C*H), 27.4 (Dipp-^{i}Pr-*C*H), 27.5 (d, $^3J_{CP}$ = 2.9 Hz, Cy-*C*H), 27.7 (Cy-*C*H), 27.9 (Dipp-^{i}Pr-*C*H$_3$), 28.0 (Cy-*C*H), 28.2 (Cy-*C*H), 28.3 (Dipp-^{i}Pr-*C*H), 28.5 (Dipp-^{i}Pr-*C*H$_3$), 28.9 (Si-^{i}Pr-*C*H$_3$), 29.0 (Si-^{i}Pr-*C*H$_3$), 29.1 (Cy-*C*H), 29.2 (Cy-*C*H), 29.3 (d, $^2J_{CP}$ = 3.3 Hz, Cy-*C*H), 30.9 (d, $^3J_{CP}$ = 2.6 Hz, Cy-*C*H), 35.4 (d, $^1J_{CP}$ = 5.5 Hz, Cy-*C*H), 35.8 (Ar-*C*), 36.0 (Ar-*C*), 123.5 (Ar-*C*), 124.0 (Ar-*C*), 124.3 (Ar-*C*), 127.0 (d, $^3J_{CP}$ = 3.3 Hz, Ar-*C*), 127.6 (d, $^3J_{CP}$ = 2.6 Hz, Ar-*C*), 134.6 (d, $^2J_{CP}$ = 5.9 Hz, Ar-*C*), 146.5 (Ar-*C*), 146.6 (Ar-*C*), 149.1 (d, $^4J_{CP}$ = 2.2 Hz, Ar-*C*), 158.4 (d, $^4J_{CP}$ = 2.2 Hz, Ar-*C*).

^{31}P{^{1}H} NMR (C$_6$D$_6$, 162 MHz, 298 K): δ = 13.3 (Cy$_2$-*P*-CH$_2$).

^{29}Si{^{1}H} NMR (C$_6$D$_6$, 99 MHz, 298 K): δ = 9.1 (d, $^2J_{SiP}$ = 11.2 Hz *Si*-iPr$_2$).

MS/LIFDI-HRMS found (calcd.) m/z: 651.3460 (651.3144) for $C_{37}H_{60}$GeNPSi.

Anal. calcd. $C_{37}H_{60}$GeNPSi: C, 68.31%; H, 9.30%; N, 2.15%; found: C, 66.94%; H, 9.42%; N, 2.00%.

General Method for preparation of [^{PhiP}Dipp(R)Ge·Ni·IPr], 3.

Toluene was added to a solid mixture of Ni(COD)$_2$ and IPr at ambient temperature, and stirred for 2 h. The resulting orange solution was cooled to -78°C and a toluene solution of **2** was added dropwise. The mixture was subsequently allowed to warm to ambient temperature and stirred for 3 h. The mixture was filtered, and all volatiles removed *in vacuo*. The residue was extracted with pentane and the solution stored at -32°C for 16 h resulting in the formation of a crystalline solid of **3**.

[{^{PhiP}Dipp(*p*-NC-Ph)Ge}·Ni·IPr], 3a. The complex was synthesized according to the general method using Ni(COD)$_2$ (461 mg, 1.68 mmol), IPr (651 mg, 1.68 mmol) and freshly prepared solution of **2a** from **1** (1.0 g, 1.68 mmol). After filtering and concentrating the resulting deep purple reaction solution to 5 mL, purple crystalline solid started to precipitate. After addition of 10 mL pentane the mixture was stored at -32°C for 16 h yielding purple crystalline solid of **3a** (1.08 g, 0.97 mmol, 58%). Deep purple crystals suitable for X-Ray diffraction analysis were grown from a concentrated Et$_2$O solution, which was stored at ambient temperature for two days.

^{1}H NMR (THF-d$_8$, 400 MHz, 298 K): δ = -0.97 (d, 3H, $^{3}J_{HH}$ = 5.3 Hz, Dipp-^{i}PrC*H$_3$*), 0.73 (m, 16H, Si-^{i}PrC*H*/Si-^{i}PrC*H$_3$*/Dipp-^{i}PrC*H$_3$*), 1.06 (m, 27H, Si-^{i}PrC*H*/Si-^{i}PrC*H$_3$*/Dipp-^{i}PrC*H$_3$*), 1.24 (d, 3H, $^{3}J_{HH}$ = 5.3 Hz, Dipp-^{i}PrC*H$_3$*), 1.65 (m, 1H, C*H$_2$*-PPh$_2$), 1.92 (m, 2H, Dipp-^{i}PrC*H$_3$*/C*H$_2$*-PPh$_2$), 2.70 (m, 1H, Si-^{i}PrC*H*), 3.01 (m, 2H, Dipp-^{i}PrC*H*), 3.26 (m, 2H, Dipp-^{i}PrC*H*), 3.94 (m, 1H, Dipp-^{i}PrC*H*), 6.29 (d, $^{3}J_{HH}$ = 8.0 Hz, Ge-Ph*H*-CN), 6.60 (d, 1H, $^{3}J_{HH}$ = 6.1 Hz, N-C*H*=C*H*-N), 6.86 (m, 3H, Ge-Ph*H*-CN/ N-C*H*=C*H*-N), 6.98 (m, 4H, Ar-C*H*), 7.19 (m, 3H, Ar-C*H*), 7.42 (m, 8H, Ar-C*H*), 7.63 (m, 2H, Ar-C*H*/N-C*H*=C*H*-N), 7.80 (m, 3H, Ar-C*H*).

^{31}P{^{1}H} NMR (THF-d$_8$, 162 MHz, 298 K): δ = 12.8 (s, *P*Ph$_2$).

^{13}C{^{1}H} NMR (THF-d$_8$, 101 MHz, 298 K): δ = 10.3 (d, $^{1}J_{CP}$ = 11.5 Hz *C*H$_2$-PPh$_2$), 15.4 (Si-^{i}Pr*C*H), 19.2 (Si-^{i}Pr-*C*H$_3$), 20.6 (Si-^{i}Pr*C*H), 21.6, 22.7 and 22.9 (Si-^{i}Pr-*C*H$_3$), 23.3, 23.5, 26. and 26.6 (Dipp-^{i}Pr-*C*H$_3$), 28.1, 28.5, 29.4 and 29.6 (Dipp-^{i}Pr*C*H), 110.6 (Ge-Ph-*C*N), 120.4 and 123.4 (Ar-*C*), 124.4 (N-*C*H=*C*H-N), 125.0, 125.6, 126.0, 128.3, 128.4, 129.1, 129.2, 129.3, 129.7, 130.3, 132.6, 132.8, 133.8, 133.9, 135.9, 139.0, 145.0, 145.6, 147.3, 164.9 and 165.0 (Ar-*C*), 201.8 (d, $^{2}J_{CP}$ = 18.5 Hz, ^{Carbene}*C*-Ni-P).

^{29}Si{^{1}H} NMR (THF-d$_8$, 99 MHz, 298 K): δ = 3.9 (d, $^{2}J_{SiP}$ = 10.2 Hz, CH$_2$-*Si*-^{i}Pr$_2$).

MS/LIFDI-HRMS found (calcd.) m/z: 1110.4683 (1110.4690) for [M]$^+$.

λ_{max}, nm (*ε*, Lcm^{-1}mol^{-1}): 799 (5030), 511 (11080), 396 (15110), 327 (16850).

Anal. calcd. for $C_{65}H_{83}GeN_4NiPSi$: C, 70.28%; H, 7.53%; N, 5.04%; found: C, 70.21%; H, 7.41%; N, 4.77%.

[{^{PhiP}Dipp(*m*-(CF$_3$)$_2$-Ph)Ge}·Ni·IPr], 3b. The complex was synthesized according to the general method using **2b** (564 mg, 0.73 mmol), Ni(COD)$_2$ (200 mg, 0.73 mmol) and IPr (283 mg, 0.73 mmol). The resulting deep red pentane solution was stored at -32°C for 16 h yielding green crystals of **3b** (471 mg, 0.39 mmol, 53%) suitable for X-Ray diffraction analysis.

^{1}H NMR (C_6D_6, 400 MHz, 298 K): δ = -0.63 (m, 3H, Dipp-^{i}PrC*H$_3$*), 0.97 (m, 37H, Si-^{i}PrC*H*/Si-^{i}PrC*H$_3$*/Dipp-^{i}PrC*H$_3$*), 1.14 (m, 3H, Dipp-^{i}PrC*H$_3$*), 1.48 (m, 6H, Dipp-^{i}PrC*H$_3$*), 1.64 (m, 1H, C*H$_2$*-PPh$_2$), 1.91 (m, 1H, C*H$_2$*-PPh$_2$), 2.07 (m, 1H, Dipp-^{i}PrC*H$_3$*), 2.95 (m, 1H, Si-^{i}PrC*H*), 3.16 (m, 4H, Dipp-^{i}PrC*H*), 4.12 (m, 1H, Dipp-^{i}PrC*H*), 6.64 (m, 3H, Ar-C*H*/N-C*H*=C*H*-N), 7.05 (m, 7H, Ar-C*H*), 7.21 (m, 7H, Ar-C*H*), 7.36 (m, 2H, Ar-C*H*), 7.47 (m, 2H, Ar-C*H*), 7.69 (m, 3H, Ar-C*H*).

^{31}P{^{1}H} NMR (C_6D_6, 162 MHz, 298 K): δ = 11.1 (s, *P*Ph$_2$).

^{13}C{^{1}H} NMR (C_6D_6, 101 MHz, 298 K): δ = 10.1 (d, $^1J_{CP}$ = 10.9 Hz *C*H$_2$-PPh$_2$), 14.6 (Si-^{i}Pr*C*H), 19.0 (Si-^{i}Pr-*C*H$_3$), 20.1 (Si-^{i}Pr*C*H), 20.3, 20.7 and 21.3 (Si-^{i}Pr-*C*H$_3$)22.5, 22.9, 23.3, 25.4, 26.1 and 26.2 (Dipp-^{i}Pr-*C*H$_3$), 27.3, 28.1, 28.5 and 28.8 (Dipp-^{i}Pr*C*H), 120.4, 123.3 and 123.5 (Ar-*C*), 124.6 (N-*C*H=*C*H-N), 124.9, 125.0, 126.0, 128.9, 129.2, 130.3, 132.1, 132.2, 132.5, 132.6, 133.4, 137.8, 141.8, 144.1, 144.4, 145.7, 146.6, 146.9, 160.6 and 160.7 (Ar-*C*), 201.2 (d, $^2J_{CP}$ = 17.8 Hz, ^{Carbene}*C*-Ni-P).

^{29}Si{^{1}H} NMR (C_6D_6, 99 MHz, 298 K): δ = 5.3 (d, $^2J_{SiP}$ = 10.2 Hz, CH$_2$-*Si*-^{i}Pr$_2$).

MS/LIFDI-HRMS found (calcd.) m/z: 1221.4416 (1221.4485) for $[M]^+$.

λ_{max}, nm (*ε*, Lcm^{-1}mol^{-1}): 770 (2630), 485 (5980), 390 (8470), 326 (9320).

Anal. calcd. for $C_{66}H_{82}F_6GeN_3NiPSi$: C, 64.88%; H, 6.77%; N, 3.44%; found: C, 62.43%; H, 6.49%; N, 3.35%.

[{^{PhiP}Dipp(Ph)Ge}·Ni·IPr], 3c. The complex was synthesized according to the general method using **2a** (507 mg, 0.79 mmol), Ni(COD)$_2$ (218 mg, 0.79 mmol) and IPr (309 mg, 0.79 mmol). The resulting deep red pentane solution was stored at -32°C for 16 h yielding green crystals of **3c** (510 mg, 0.47mmol, 59%) suitable for X-Ray diffraction analysis.

^{1}H NMR (C_6D_6, 400 MHz, 298 K): δ = -0.54 (m, 3H, Dipp-^{i}PrC*H$_3$*), 1.01 (m, 37H, Si-^{i}PrC*H*/Si-^{i}PrC*H$_3$*/Dipp-^{i}PrC*H$_3$*), 1.22 (m, 3H, Dipp-^{i}PrC*H$_3$*), 1.47 (m, 6H, Dipp-^{i}PrC*H$_3$*), 1.89 (m, 2H, C*H$_2$*-PPh$_2$), 2.22 (m, 1H, Dipp-^{i}PrC*H$_3$*), 2.78 (m, 1H, Si-^{i}PrC*H*), 3.15 (m, 2H, Dipp-^{i}PrC*H*), 3.38 (m, 2H, Dipp-^{i}PrC*H*), 4.23 (m, 1H, Dipp-^{i}PrC*H*), 6.54 (d, 2H, $^3J_{HH}$ = 6.9 Hz, Ar-C*H*), 6.68 (s, 2H, N-C*H*=C*H*-N), 6.89 (m, 6H, Ar-C*H*), 7.09 (m, 6H, Ar-C*H*), 7.23 (m, 4H, Ar-C*H*), 7.42 (m, 4H, Ar-C*H*), 7.89 (m, 2H, Ar-C*H*).

^{31}P{^{1}H} NMR (C_6D_6, 162 MHz, 298 K): δ = 8.7 (s, *P*Ph$_2$).

^{13}C{^{1}H} NMR (C_6D_6, 101 MHz, 298 K): δ = 10.3 (d, $^1J_{CP}$ = 5.5 Hz *C*H$_2$-PPh$_2$), 14.8 (Si-^{i}Pr*C*H), 19.0 (Si-^{i}Pr-*C*H$_3$), 20.1 (Si-^{i}Pr*C*H), 20.4, 20.6 and 21.3 (Si-^{i}Pr-*C*H$_3$), 22.3, 22.6, 23.1, 23.2, 25.1 and 26.3 (Dipp-

^{i}Pr-CH_3), 27.9, 28.2, 28.6 and 28.9 (Dipp-^{i}Pr*C*H), 123.0 and 124.1 (Ar-*C*), 124.2 (N-*C*H=*C*H-N), 124.7, 124.8, 125.3, 126.7, 127.3, 127.6, 127.7, 129.4, 131.9, 132.1, 133.4, 133.6, 134.9, 138.6, 141.6, 142.0, 142.8, 144.4, 145.1, 146.9, 147.0, 147.1, 156.6 and 156.7 (Ar-*C*), 203.6 (d, $^2J_{CP}$ = 18.3 Hz, $^{Carbene}C$-Ni-P).

^{29}Si{^{1}H} NMR (C_6D_6, 99 MHz, 298 K): δ = 3.7 (d, $^2J_{SiP}$ = 10.8 Hz, CH_2-*Si*-^{i}Pr_2).

MS/LIFDI-HRMS found (calcd.) m/z: 1085.4713 (1085.4737) for $[M]^+$.

λ_{max}, nm (ε, $Lcm^{-1}mol^{-1}$): 752 (2710), 485 (6030), 388 (8600).

Anal. calcd. for $C_{64}H_{84}GeN_3NiPSi$: C, 70.80%; H, 7.80%; N, 3.87%; found: C, 70.58%; H, 7.93%; N, 4.03%.

[{^{PhiP}Dipp(*p*-Me-Ph)Ge}·Ni·IPr, 3d. The complex was synthesized according to the general method using $Ni(COD)_2$ (115 mg, 0.42 mmol), IPr (163 mg, 0.42 mmol) and freshly prepared solution of **2d** from **1** (250 mg, 0.42 mmol). The resulting deep red pentane solution was stored at -32°C for 16 h yielding a red crystalline solid of **3d** (227 mg, 0.21 mmol, 49%). Green crystals suitable for X-Ray diffraction analysis were grown from a concentrated Et_2O solution, which was stored at -32°C temperature for two days.

^{1}H NMR (C_6D_6, 400 MHz, 298 K): δ = -0.48 (d, 3H, $^3J_{HH}$ = 5.8 Hz, Dipp-^{i}PrC*H*$_3$), 1.06 (m, 40H, Si-^{i}PrC*H*/Si-^{i}PrC*H*$_3$/Dipp-^{i}PrC*H*$_3$), 1.48 (d, 6H, $^3J_{HH}$ = 6.0 Hz, Dipp-^{i}PrC*H*$_3$), 1.88 (m, 2H, C*H*$_2$-PPh_2), 2.02 (s, 3H, Ge-Ph-C*H*$_3$), 2.26 (m, 1H, Dipp-^{i}PrC*H*$_3$), 2.78 (m, 1H, Si-^{i}PrC*H*), 3.17 (m, 2H, Dipp-^{i}PrC*H*), 3.40 (m, 2H, Dipp-^{i}PrC*H*), 4.23 (m, 1H, Dipp-^{i}PrC*H*), 6.47 (d, 2H, $^3J_{HH}$ = 7.7 Hz, Ge-Ph*H*-CH_3), 6.69 (s, 2H, N-C*H*=C*H*-N), 6.77 (d, 2H, $^3J_{HH}$ = 7.7 Hz, Ge-Ph*H*-CH_3), 6.91 (m, 4H, Ar-C*H*), 7.11 (m, 4H, Ar-C*H*), 7.23 (m, 5H, Ar-C*H*), 7.44 (m, 4H, Ar-C*H*), 7.90 (m, 2H, Ar-C*H*).

^{31}P{^{1}H} NMR (C_6D_6, 162 MHz, 298 K): δ = 8.1 (s, *P*Ph_2).

^{13}C{^{1}H} NMR (C_6D_6, 101 MHz, 298 K): δ = 10.6 (d, $^1J_{CP}$ = 9.7 Hz *C*H_2-PPh_2), 14.86 (Si-^{i}Pr*C*H), 19.0 (Si-^{i}Pr-*C*H_3), 20.2 (Si-^{i}Pr*C*H), 20.4, 20.6 and 21.4 (Si-^{i}Pr*C*H_3), 21.7 (Ge-Ph-*C*H_3), 22.4, 22.6, 23.1, 23.3, 25.0 and 26.3 (Dipp^{i}Pr-*C*H_3), 27.9, 28.2, 28.6 and 28.9 (Dipp-^{i}Pr*C*H), 123.0 and 124.0 (Ar-*C*), 124.2 (N-*C*H=*C*H-N), 124.7, 124.9, 125.4, 127.5, 127.6, 127.6, 129.4, 131.9 132.1, 133.5, 133.7, 135.1, 136.6, 138.4, 144.4, 145.2, 147.0, 147.2, 147.3, 153.4 and 153.5 (Ar-*C*), 203.9 (d, $^2J_{CP}$ = 18.1 Hz, $^{Carbene}C$-Ni-P).

^{29}Si{^{1}H} NMR (C_6D_6, 99 MHz, 298 K): δ = 3.6 (d, $^2J_{SiP}$ = 10.7 Hz, CH_2-*Si*-^{i}Pr_2).

MS/LIFDI-HRMS found (calcd.) m/z: 1099.4945 (1099.4894) for $[M]^+$.

λ_{max}, nm (ε, $Lcm^{-1}mol^{-1}$): 748 (2760), 487 (6170), 387 (8590).

Anal. calcd. for $C_{65}H_{86}GeN_3NiPSi$: C, 70.99%; H, 7.88%; N, 3.82%; found: C, 71.77%; H, 8.16%; N, 3.55%.

[{^{PhiP}Dipp(*p*-OMe-Ph)Ge}·Ni·IPr], 3e. The complex was synthesized according to the general method using $Ni(COD)_2$ (230 mg, 0.84 mmol), IPr (307 mg, 0.84 mmol) and freshly prepared solution of **2e** from **1** (500 mg, 0.84 mmol). The resulting deep red pentane solution was stored at -32°C for 16 h yielding a red crystalline solid of **3e** (433 mg, 0.39 mmol, 46%). Green crystals suitable for X-Ray diffraction analysis were grown from a concentrated Et_2O solution, which was stored at -32°C temperature for two days.

^{1}H NMR (C_6D_6, 400 MHz, 298 K): δ = -0.44 (d, 3H, $^3J_{HH}$ = 6.2 Hz, Dipp-^{i}PrC*H₃*), 0.91 (m, 12H, Si-^{i}PrC*H₃*/Dipp-^{i}PrC*H₃*), 1.07 (m, 25H, Si-^{i}PrC*H*/Si-^{i}PrC*H₃*/Dipp-^{i}PrC*H₃*), 1.23 (m, 3H, Si-^{i}PrC*H₃*), 1.48 (d, 6H, $^3J_{HH}$ = 5.8 Hz, Dipp-^{i}PrC*H₃*), 1.90 (m, 2H, C*H₂*-PPh_2), 2.29 (sept, 1H, $^3J_{HH}$ = 6.3 Hz, Dipp-^{i}PrC*H₃*), 2.75 (sept, 1H, $^3J_{HH}$ = 6.9 Hz, Si-^{i}PrC*H*), 3.14 (sept, 2H, $^3J_{HH}$ = 6.4 Hz, Dipp-^{i}PrC*H*), 3.22 (s, 3H, Ge-Ph-OC*H₃*), 3.42 (m, 2H, Dipp-^{i}PrC*H*), 4.25 (sept, 1H, $^3J_{HH}$ = 6.7 Hz, Dipp-^{i}PrC*H*), 6.48 (d, 2H, $^3J_{HH}$ = 8.5 Hz, Ge-Ph*H*-OCH_3), 6.57 (d, 2H, $^3J_{HH}$ = 8.5 Hz, Ge-Ph*H*-OCH_3), 6.68 (s, 2H, N-C*H*=C*H*-N), 6.91 (m, 4H, Ar-C*H*), 7.10 (m, 4H, Ar-C*H*), 7.24 (m, 5H, Ar-C*H*), 7.39 (m, 2H, Ar-C*H*), 7.48 (m, 2H, Ar-C*H*), 7.91 (m, 2H, Ar-C*H*).

^{31}P{^{1}H} NMR (C_6D_6, 162 MHz, 298 K): δ = 7.7 (s, *P*Ph_2).

^{13}C{^{1}H} NMR (C_6D_6, 101 MHz, 298 K): δ = 10.7 (d, $^1J_{CP}$ = 9.5 Hz *C*H_2-PPh_2), 14.9 (Si-^{i}Pr*C*H), 19.0 (Si^{i}Pr-*C*H_3), 20.1 (Si-^{i}Pr*C*H), 20.4, 20.6 and 21.4 (Si^{i}Pr*C*H_3), 22.5, 22.5, 23.2, 23.5, 24.9 and 26.2 (Dipp^{i}Pr-*C*H_3), 28.0, 28.1, 28.6 and 28.8 (Dipp-^{i}Pr*C*H), 54.3 (Ge-Ph-O*C*H_3), 112.4, 123.0 and 124.0 (Ar-*C*), 124.1 (N-*C*H=*C*H-N), 124.7, 124.8, 125.4, 127.5, 127.6, 129.4, 131.9, 132.1, 133.5, 133.7, 136.6, 138.5, 144.5, 145.3, 146.9, 147.3, 148.2, 148.3 and 159.6 (Ar-*C*), 204.0 (d, $^2J_{CP}$ = 18.3 Hz, ^{Carbene}*C*-Ni-P).

^{29}Si{^{1}H} NMR (C_6D_6, 99 MHz, 298 K): δ = 3.5 (d, $^2J_{SiP}$ = 10.5 Hz, CH_2-*Si*-^{i}Pr_2).

MS/LIFDI-HRMS found (calcd.) m/z: 1115.4914 (1115.4843) for $[M]^+$.

λ_{max}, nm (*ε*, $Lcm^{-1}mol^{-1}$): 747 (2840), 483 (6370), 388 (8630).

Anal. calcd. for $C_{65}H_{86}GeN_3NiOPSi$: C, 69.97%; H, 7.77%; N, 3.77%; found: C, 69.39%; H, 8.17%; N, 3.53%.

[{^{PhiP}Dipp(*p*-Me_2N-Ph)Ge}·Ni·IPr], 3f. The complex was synthesized according to the general method using $Ni(COD)_2$ (115 mg, 0.42 mmol), IPr (163 mg, 0.42 mmol) and freshly prepared solution of **2f** from **1** (250 mg, 0.42 mmol). The resulting deep red pentane solution was stored at -32°C for 16 h yielding a red crystalline solid of **3f** (226 mg, 0.20 mmol, 48%). Green crystals suitable for X-Ray

diffraction analysis were grown from a concentrated Et_2O solution, which was stored at -32°C temperature for two days.

^{1}H NMR (C_6D_6, 400 MHz, 298 K): δ = -0.33 (d, 3H, $^3J_{HH}$ = 6.8 Hz, Dipp-^{i}PrC*H₃*), 1.09 (m, 40H, Si-^{i}PrC*H*/Si-^{i}PrC*H₃*/Dipp-^{i}PrC*H₃*), 1.47 (m, 6H, Dipp-^{i}PrC*H₃*), 1.93 (m, 2H, C*H₂*-PPh₂), 2.38 (m, 1H, Dipp-^{i}PrC*H₃*), 2.48 (s, 6H, Ge-Ph-N(C*H₃*)₂), 2.72 (m, 1H, Si-^{i}PrC*H*), 3.23 (m, 2H, Dipp-^{i}PrC*H*), 3.48 (m, 2H, Dipp-^{i}PrC*H*), 4.26 (m, 1H, Dipp-^{i}PrC*H*), 6.33 (d, 2H, $^3J_{HH}$ = 8.4 Hz, Ge-Ph*H*-N(CH₃)₂), 6.46 (d, 2H, $^3J_{HH}$ = 8.4 Hz, Ge-Ph*H*-N(CH₃)₂), 6.71 (s, 2H, N-C*H*=C*H*-N), 6.93 (m, 4H, Ar-C*H*), 7.08 (m, 4H, Ar-C*H*), 7.27 (m, 5H, Ar-C*H*), 7.49 (m, 4H, Ar-C*H*), 7.93 (m, 2H, Ar-C*H*).

^{31}P{^{1}H} NMR (C_6D_6, 162 MHz, 298 K): δ = 6.5 (s, *P*Ph₂).

^{13}C{^{1}H} NMR (C_6D_6, 101 MHz, 298 K): δ = 10.3 (d, $^1J_{CP}$ = 8.7 Hz *C*H₂-PPh₂), 14.9 (Si-^{i}Pr*C*H), 19.0 (Si--^{i}Pr-*C*H₃), 20.2 (Si-^{i}Pr*C*H), 20.4, 20.7 and 21.4 (Si-^{i}Pr*C*H₃), 22.5, 22.6, 23.3, 23.7, 24.9 and 26.2 (Dipp-^{i}Pr-*C*H₃), 28.2, 28.6 and 28.8 (Dipp-^{i}Pr*C*H), 39.8 (Ge-Ph-N(*C*H₃)₂), 110.9, 123.0 and 123.9 (Ar-*C*), 124.1 (N-*C*H=*C*H-N), 124.6, 124.8, 125.3, 127.5, 127.6, 127.6, 129.2, 131.9, 132.1, 133.7, 133.9, 136.3, 138.7, 142.4, 142.7, 143.6, 144.0, 144.1, 144.6, 145.3, 147.1, 147.6 and 150.1 (Ar-*C*), 204.7 (d, $^2J_{CP}$ = 18.5 Hz, ^{Carbene}*C*-Ni-P).

^{29}Si{^{1}H} NMR (C_6D_6, 99 MHz, 298 K): δ = 3.2 (d, $^2J_{SiP}$ = 10.7 Hz, CH₂-*Si*-^{i}Pr₂).

MS/LIFDI-HRMS found (calcd.) m/z: 1128.5175 (1128.5159) for $[M]^+$.

λ_{max}, nm (*ε*, $Lcm^{-1}mol^{-1}$): 741 (2500), 484 (5990), 337 (15680).

Anal. calcd. for $C_{66}H_{89}GeN_4NiPSi$: C, 70.22%; H, 7.95%; N, 4.96%; found: C, 68.40%; H, 8.02%; N, 4.68%.

[{^{PhiP}Dipp(Me₂N)Ge}·Ni·IPr], 3g. The complex was synthesized according to the general method using Ni(COD)₂ (115 mg, 0.42 mmol), IPr (163 mg, 0.42 mmol) and freshly prepared solution of **2g** from **1** (250 mg, 0.42 mmol). The resulting deep green pentane solution was stored at -32°C for 16 h yielding a green crystalline solid of **3g** (117 mg, 0.11 mmol, 27%). Red crystals suitable for X-Ray diffraction analysis were grown from a concentrated Et_2O solution, which was stored at -32°C temperature for two days.

^{1}H NMR (C_6D_6, 400 MHz, 298 K): δ = 0.32 (m, 3H, Dipp-^{i}PrC*H₃*), 0.81 (m, 6H, Si-^{i}PrC*H₃*/Dipp-^{i}PrC*H₃*), 1.09 (m, 34H, Si-^{i}PrC*H*/Si-^{i}PrC*H₃*/Dipp-^{i}PrC*H₃*), 1.37 (m, 6H, Dipp-^{i}PrC*H₃*), 1.87 (m, 1H, C*H₂*-PPh₂), 2.01 (overlapping singlets, 6H, Ge-N(C*H₃*)₃), 2.13 (m, 2H, C*H₂*-PPh₂/Dipp-^{i}PrC*H₃*), 2.75 (m, 1H, Si-^{i}PrC*H*), 3.15 (m, 2H, Dipp-^{i}PrC*H*), 3.52 (m, 2H, Dipp-^{i}PrC*H*), 4.06 (m, 1H, Dipp-^{i}PrC*H*), 6.65 (s, 2H, N-C*H*=C*H*-N), 7.04 (m, 13H, Ar-C*H*), 7.27 (m, 2H, Ar-C*H*), 7.53 (m, 2H, Ar-C*H*), 7.92 (m, 2H, Ar-C*H*).

^{31}P{^{1}H} NMR (C_6D_6, 162 MHz, 298 K): δ = 1.5 (s, *P*Ph₂).

$^{13}C\{^1H\}$ NMR (C_6D_6, 101 MHz, 298 K): δ = 13.6 (d, $^1J_{CP}$ = 6.5 Hz *C*H_2-PPh_2), 14.6 and 18.9 (Si--^{i}Pr-*C*H), 20.1, 20.2, 20.5 and 21.4 (Si-^{i}Pr*C*H_3), 22.5, 23.1, 23.6, 24.0, 24.2 and 26.0 (Dipp-^{i}Pr-*C*H_3), 28.0 and 28.6 (Dipp-^{i}Pr*C*H), 43.5 (Ge-N(*C*H_3)$_2$), 123.3 and 123.8 (Ar-*C*), 124.1 (N-*C*H=*C*H-N), 124.5, 124.6, 124.7, 124.8, 124.9, 129.2, 131.8, 131.9, 134.2, 134.4, 139.1, 144.8, 145.6 and 146.8 (Ar-*C*), 205.4 (d, $^2J_{CP}$ = 22.8 Hz, ^{Carbene}*C*-Ni-P).

$^{29}Si\{^1H\}$ NMR (C_6D_6, 99 MHz, 298 K): δ = 3.7 (d, $^2J_{SiP}$ = 12.0 Hz, CH_2-*Si*-^{i}Pr_2).

MS/LIFDI-HRMS found (calcd.) m/z: 1052.4789 (1052.4846) for $[M]^+$.

λ_{max}, nm (*ε*, $Lcm^{-1}mol^{-1}$): 623 (2830), 395 (10290).

Anal. calcd. for $C_{60}H_{85}GeN_4NiPSi$: C, 68.45%; H, 8.14%; N, 5.32%; found: C, 67.14%; H, 8.14%; N, 4.97%.

[{^{PhiP}Dipp(Ph)Ge(H)}Ni(H)·IPr], 4c.

Method A: A deep red Et_2O solution of **3c** (100 mg, 0.092 mmol) was subjected to 1.5 bar H_2 leading to slight decolorization and was stored at ambient temperature for 24 h, resulting in yellow crystals of **4c** (89 mg, 0.082 mmol, 89%) suitable for X-Ray diffraction analysis. NMR data of the equilibrium between **3c** and **4c** were obtained by subjecting **3c** to an ambient pressure of H_2 in a J-Young NMR tube.

Method B: A deep red Et_2O (5 mL) solution of **3c** (100 mg, 0.092 mmol) was cooled to -40 °C, and neat $PhSiH_3$ added (12.4 µL, 1.1 equiv.). The mixture was briefly stirred, and the reaction warmed to room temperature. After standing for 1 hr, a significant amount of yellow precipitate had formed, which was isolated by filtration, and found to be analytically pure **4c** (76 mg, 77%).

^1H NMR (C_6D_6, 400 MHz, 333 K): δ = -1.98 (b, 2H, Ge-*H*/Ni-*H*), 0.65 (d, 6H, $^3J_{HH}$ = 5.6 Hz, Si-^{i}Pr-C*H_3*), 0.88 (m, 12H, ^{i}Pr-C*H_3*), 0.89 (m, 18H, ^{i}Pr-C*H_3*), 1.07 (d, 6H, $^3J_{HH}$ = 6.7 Hz, Dipp-^{i}Pr-C*H_3*) 1.19 (d, 6H, $^3J_{HH}$ = 6.7 Hz, -Dipp-^{i}Pr-C*H_3*), 1.29 (m, 2H, Si-^{i}Pr-C*H*), 1.85 (d, 2H, $^2J_{HP}$ = 12.0 Hz, C*H_2*-PPh_2), 3.13 (m, 4H, Dipp-^{i}PrC*H*), 3.31 (m, 2H, Dipp-^{i}PrC*H*), 6.72 (s, 2H, N-C*H*=C*H*-N), 7.00 (m, 12H, Ar-C*H*), 7.12 (m, 4H, Ar-C*H*), 7.19 (m, 2H, Ar-C*H*), 7.29 (m, 2H, Ar-C*H*), 7.65 (m, 4H, Ar-C*H*).

^1H NMR (D_8-Toluene, 400 MHz, 213 K): δ = -10.02 (br d, 1H, $^2J_{PH}$ = 95 Hz, Ni-*H*), 5.23 (v br, 1H, Ge-*H*).

$^{31}P\{^1H\}$ NMR (C_6D_6, 162 MHz, 298 K): δ = 9.0 (s, *P*Ph_2).

IR, *v*/cm^{-1} (ATR): 1949 (*Ge-H*) and 1893 (*Ni-H*).

[{^{PhiP}Dipp(Ph)Ge(D)}Ni(D)·IPr], 4c-D_2. A deep red Et_2O solution of **3c** (100 mg, 0.092 mmol) was subjected to 1.5 bar D_2 leading to slight decolorization and was stored at ambient temperature for 24

h, resulting in a yellow crystalline solid of **4c-D$_2$** (86 mg, 0.082 mmol, 86%). NMR data of the equilibrium between **3c** and **4c-D$_2$** were obtained by subjecting **3c** to an ambient pressure of H_2 in a J-Young NMR tube.

^{1}H NMR (toluene-d$_8$, 400 MHz, 333 K): δ = 0.58 (d, 6H, $^3J_{HH}$ = 5.6 Hz, Si-^{i}Pr-C*H$_3$*), 0.85 (m, 12H, ^{i}Pr-C*H$_3$*), 0.93 (m, 18H, ^{i}Pr-C*H$_3$*), 1.05 (d, 6H, $^3J_{HH}$ = 6.7 Hz, Dipp-^{i}Pr-C*H$_3$*) 1.12 (d, 6H, $^3J_{HH}$ = 6.7 Hz, -Dipp-^{i}Pr-C*H$_3$*), 1.24 (m, 2H, Si-^{i}Pr-C*H*), 1.80 (d, 2H, $^2J_{HP}$ = 12.0 Hz, C*H$_2$*-PPh$_2$), 3.09 (m, 4H, Dipp-^{i}PrC*H*), 3.24 (m, 2H, Dipp-^{i}PrC*H*), 6.72 (s, 2H, N-C*H*=C*H*-N), 6.95 (m, 12H, Ar-C*H*), 7.09 (D, 6H, $^3J_{HH}$ = 7.5 Hz; Ar-C*H*), 7.27 (m, 2H, Ar-C*H*), 7.61(m, 4H, Ar-C*H*).

^{31}P{^{1}H} NMR (C_6D_6, 162 MHz, 298 K): δ = 9.1 (s, *P*Ph$_2$).

[[^{PhiP}Dipp{*m*-(CF$_3$)$_2$-Ph}Ge(H)]Ni(H)·IPr], 4b. A deep red pentane solution of **3b** (163 mg, 0.42 mmol) was subjected to 1.5 bar H_2 leading to a complete light-yellow solution and was stored for a prolonged time but did not result in any precipitation of **4b**. NMR data of the equilibrium between **3b** and **4b** were obtained by subjecting **3b** to an ambient pressure of H_2 in a J-Young NMR tube.

^{1}H NMR (toluene-d$_8$, 400 MHz, 333 K): δ = -2.58 (b, 2H, Ge-*H*/Ni-*H*), 0.57 (m, 6H, Si-^{i}Pr-C*H$_3$*), 0.75 (m, 15H, ^{i}Pr-C*H$_3$*), 0.86 (m, 20H, Si-^{i}Pr-C*H*/^{i}Pr-C*H$_3$*), 1.08 (d, 6H, $^3J_{HH}$ = 6.6 Hz, Dipp-^{i}Pr-C*H$_3$*), 1.73 (d, 2H, $^2J_{HP}$ = 13.2 Hz, C*H$_2$*-PPh$_2$), 2.92 (sept, 4H, $^3J_{HH}$ = 6.7 Hz, Dipp-^{i}PrC*H*), 3.21 (m, 2H, Dipp-^{i}PrC*H*), 6.67 (s, 2H, N-C*H*=C*H*-N), 6.85 (m, 2H, Ar-C*H*), 7.00 (m, 10H, Ar-C*H*), 7.23 (m, 3H, Ar-C*H*), 7.56 (m, 4H, Ar-C*H*), 7.70 (s, 1H, 3,5-(CF$_3$)$_2$-PhH_p-Ge), 7.80 (s, 2H, 3,5-(CF$_3$)$_2$-Ph(H_o)$_2$-Ge).

^{1}H NMR (D$_8$-Toluene, 400 MHz, 213 K): δ = -10.72 (br d, 1H, $^2J_{PH}$ = 107 Hz, Ni-*H*), 5.59 (v br, 1H, Ge-H).

^{31}P{^{1}H} NMR (C_6D_6, 162 MHz, 298 K): δ = 6.7 (s, *P*Ph$_2$).

[{^{PhiP}DippGe(4-CN-C$_6$H$_4$)(H)}Ni(H)·IPr], 4a. A deep purple Et$_2$O solution of **3a** (150 mg, 0.14 mmol) was subjected to 1.5 bar H_2 leading to a red solution and was stored at ambient temperature for 24 h, resulting in red crystals of **4a** (132 mg, 0.12 mmol, 88%) suitable for X-Ray diffraction analysis. NMR data of the equilibrium between **3a** and **4a** were obtained by subjecting **3a** to an ambient pressure of H_2 in a J-Young NMR tube.

^{1}H NMR (C_6D_6, 400 MHz, 333 K): δ = -2.33 (b, 2H, Ge-*H*/Ni-*H*), 0.58 (m, 6H, Si-^{i}Pr-C*H$_3$*), 0.88 (m, 32H, Si-^{i}Pr-C*H*/^{i}Pr-C*H$_3$*), 1.02 (d, 6H, $^3J_{HH}$ = 6.2 Hz, Dipp-^{i}Pr-C*H$_3$*), 1.13 (d, 6H, $^3J_{HH}$ = 6.7 Hz, Dipp-^{i}Pr-C*H$_3$*) 1.79 (d, 2H, $^2J_{HP}$ = 11.9 Hz, C*H$_2$*-PPh$_2$), 3.01 (m, 4H, Dipp-^{i}PrC*H*), 3.17 (m, 2H, Dipp-^{i}PrC*H*), 6.69 (s, 2H, N-C*H*=C*H*-N), 7.00 (m, 17H, Ar-C*H*), 7.25 (m, 2H, Ar-C*H*), 7.58 (m, 4H, Ar-C*H*).

^{1}H NMR (D$_8$-THF, 400 MHz, 233 K): δ = -10.86 (br d, 1H, $^2J_{PH}$ = 105 Hz, Ni-*H*), 6.03 (v br, 1H, Ge-*H*).

^{31}P{^{1}H} NMR (C_6D_6, 162 MHz, 298 K): δ = 8.0 (s, *P*Ph$_2$).

IR, ν/cm^{-1} (ATR): 2227 (Ph-*C≡N*), 1934 (*Ge-H*) and 1885 (*Ni-H*).

[{^{PhiP}DippGe(4-Me-C_6H_4)(H)}Ni(H)·IPr], 4d. A deep red Et_2O solution of **3d** (150 mg, 0.14 mmol) was subjected to 1.5 bar H_2 leading to a red solution and was stored at ambient temperature for 24 h, resulting in yellow crystalline solid of **4d** (121 mg, 0.11 mmol, 80%). NMR data of the equilibrium between **3d** and **4d** were obtained by subjecting **3d** to an ambient pressure of H_2 in a J-Young NMR tube.

^{1}H NMR (C_6D_6, 400 MHz, 333 K): δ = -1.97 (b, 2H, Ge-*H*/Ni-*H*), 0.68 (d, 6H, $^3J_{HH}$ = 5.3 Hz, Si-^{i}Pr-C*H$_3$*), 0.89 (m, 6H, $^3J_{HH}$ = 7.5 Hz, Dipp-^{i}Pr-C*H$_3$*), 0.98 (m, 24, ^{i}Pr-C*H$_3$*), 1.07 (d, 6H, $^3J_{HH}$ = 6.7 Hz, Dipp-^{i}Pr-C*H$_3$*), 1.20 (d, 6H, $^3J_{HH}$ = 6.8 Hz, Dipp-^{i}Pr-C*H$_3$*), 1.30 (m, 2H, Si-^{i}PrC*H*), 1.85 (d, 2H, $^2J_{HP}$ = 12.0 Hz, C*H$_2$*-PPh$_2$), 2.17 (s, 3H, Ge-Ph-C*H$_3$*), 3.15 (m, 4H, Dipp-^{i}PrC*H*), 3.33 (m, 2H, Dipp-^{i}PrC*H*), 6.73 (s, 2H, N-C*H*=C*H*-N), 7.06 (m, 15H, Ar-C*H*), 7.29 (m, 4H, Ar-C*H*), 7.66 (m, 4H, Ar-C*H*).

^{1}H NMR (D_8-Toluene, 400 MHz, 213 K): δ = -9.95 (br d, 1H, $^2J_{PH}$ = 108 Hz, Ni-*H*), 5.30 (v br, 1H, Ge-*H*).

^{31}P{^{1}H} NMR (C_6D_6, 162 MHz, 298 K): δ = 9.0 (s, *P*Ph$_2$).

IR, ν/cm^{-1} (ATR): 1940 (*Ge-H*) and 1879 (*Ni-H*).

[{^{PhiP}DippGe(4-OMe-C_6H_4)(H)}Ni(H)·IPr], 4e. A deep red Et_2O solution of **3e** (100 mg, 0.089 mmol) was subjected to 1.5 bar H_2 leading to a red solution and was stored at -32°C for 24 h, resulting in yellow crystalline solid of **4e** (23 mg, 0.021 mmol, 23%). NMR data of the equilibrium between **3e** and **4e** were obtained by subjecting **3e** to an ambient pressure of H_2 in a J-Young NMR tube.

^{1}H NMR (toluene-d$_8$, 400 MHz, 333 K): δ = -2.02 (b, 2H, Ge-*H*/Ni-*H*), 0.62 (m, 6H, Si-^{i}Pr-C*H$_3$*), 0.84 (m, 6H, $^3J_{HH}$ = 7.5 Hz, Dipp-^{i}Pr-C*H$_3$*), 0.95 (m, 24, ^{i}Pr-C*H$_3$*), 1.06 (d, 6H, $^3J_{HH}$ = 6.6 Hz, Dipp-^{i}Pr-C*H$_3$*), 1.14 (d, 6H, $^3J_{HH}$ = 6.7 Hz, Dipp-^{i}Pr-C*H$_3$*), 1.26 (m, 2H, Si-^{i}PrC*H*), 1.80 (d, 2H, $^2J_{HP}$ = 12.0 Hz, C*H$_2$*-PPh$_2$), 3.11 (m, 4H, Dipp-^{i}PrC*H*), 3.25 (m, 2H, Dipp-^{i}PrC*H*), 3.38 (s, 3H, Ge-Ph-OC*H$_3$*), 6.54 (d, $^3J_{HH}$ = 8.0 Hz, Ge-Ph*H*-OCH$_3$), 6.73 (s, 2H, N-C*H*=C*H*-N), 6.97 (m, 11H, Ar-C*H*), 7.10 (m, 4H, Ar-C*H*), 7.22 (d, $^3J_{HH}$ = 7.7 Hz, Ge-Ph*H*-OCH$_3$), 7.63 (m, 4H, Ar-C*H*).

^{1}H NMR (D_8-Toluene, 400 MHz, 213 K): δ = -9.92 (br d, 1H, $^2J_{PH}$ = 109 Hz, Ni-*H*), 5.29 (v br, 1H, Ge-*H*).

^{31}P{^{1}H} NMR (C_6D_6, 162 MHz, 298 K): δ = 9.2 (s, *P*Ph$_2$).

IR, ν/cm^{-1} (ATR): 1936 (*Ge-H*) and 1881 (*Ni-H*).

[{^{PhiP}DippGe(4-NMe$_2$-C_6H_4)(H)}Ni(H)·IPr], 4f. A deep red Et_2O solution of **3f** (100 mg, 0.089 mmol) was subjected to 1.5 bar H_2 leading to a red solution and was stored at -32°C for 24 h, resulting in

yellow crystalline solid of **4f** (23 mg, 0.021 mmol, 23%). NMR data of the equilibrium between **3f** and **4f** were obtained by subjecting **3f** to an ambient pressure of H_2 in a J-Young NMR tube.

^{1}H NMR (tol-d8, 400 MHz, 333.15 K): δ = -1.94 (b, 2H, Ge-*H*/Ni-*H*), 0.65 (d, 6H, $^3J_{HH}$ = 5.6 Hz, C*H$_3$*), 1.82 (d, 2H, $^2J_{HP}$ = 12.0 Hz, C*H$_2$*-PPh$_2$), 2.50 (s, 6H, Ge-N(C*H$_3$*)$_2$), 3.13 (m, 4H, Dipp-^{i}PrCH), 3.31 (m, 2H, Dipp-^{i}PrCH), 6.22 (d, $^3J_{HH}$ = 8.4 Hz, Ge-Ph*H*-NMe$_2$), 6.36 (d, $^3J_{HH}$ = 8.6 Hz, Ge-Ph*H*-NMe$_2$), 6.73 (s, 2H, N-C*H*=C*H*-N), 6.94 (m, 5H, Ar-C*H*), 7.12 (m, 4H, Ar-C*H*), 7.26 (m, 4H, Ar-C*H*), 7.42 (m, 2H, Ar-C*H*), 7.64 (m, 4H, Ar-C*H*).

^{1}H NMR (D$_8$-Toluene, 400 MHz, 213 K): δ = -9.75 (br d, 1H, $^2J_{PH}$ = 105 Hz, Ni-*H*), 5.22 (v br, 1H, Ge-*H*).

N. B. In the region between 1.3 and 0.7 ppm, CH_3 and CH signals of **3f** and **4f** overlap and therefore cannot be distinguished.

^{31}P{^{1}H} NMR (C_6D_6, 162 MHz, 298 K): δ = 9.3 (s, *P*Ph$_2$).

IR, *v*/cm^{-1} (ATR): 1940 (*Ge-H*) and 1883 (*Ni-H*).

NMR, MS, UV/vis, and IR spectra

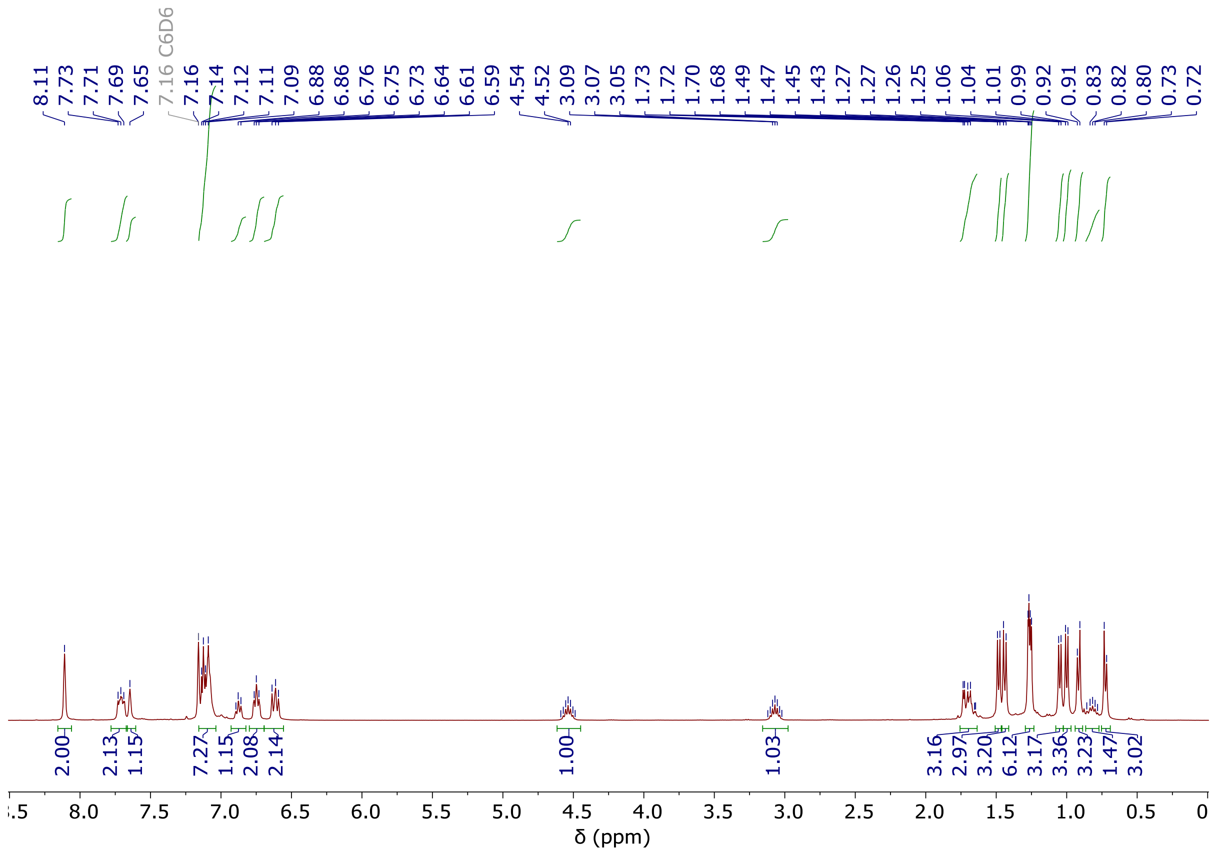

Figure S1. ^{1}H NMR spectrum of **2b** as a solution in C_6D_6 at ambient temperature.

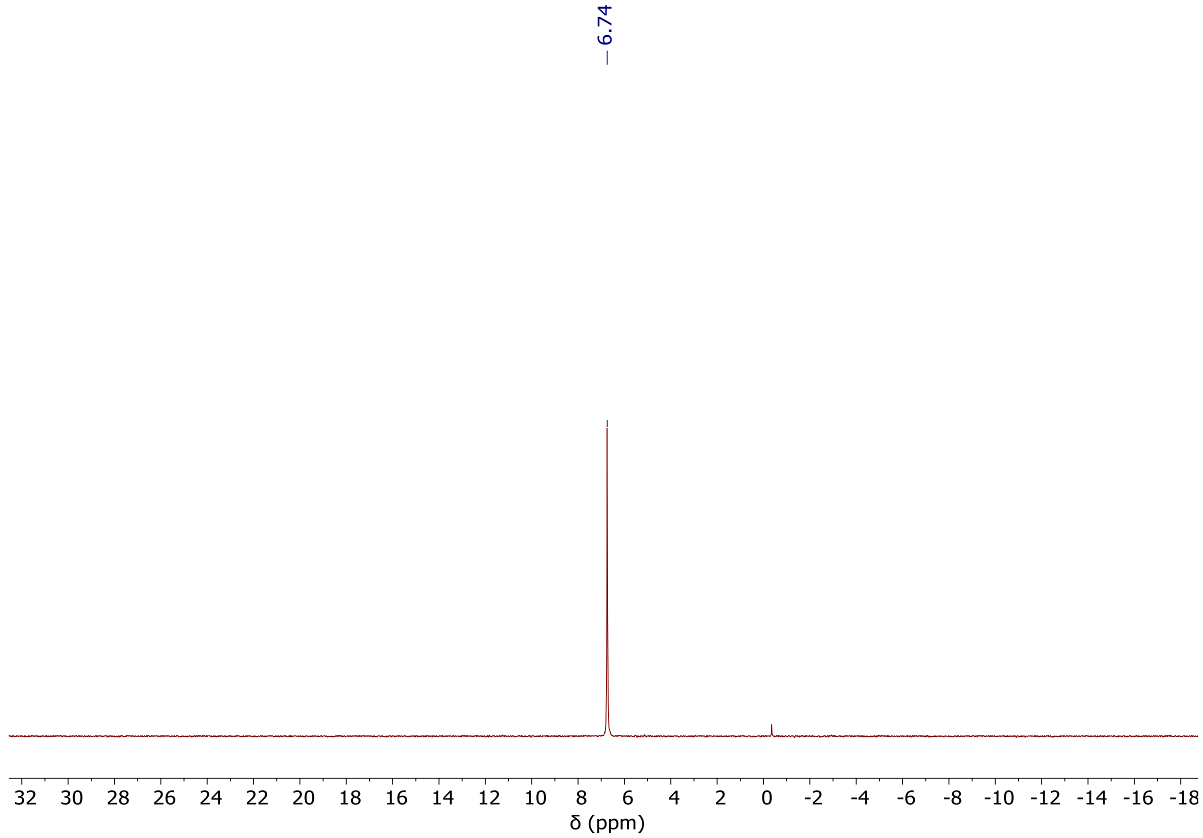

Figure S2. $^{31}P\{^{1}H\}$ NMR spectrum of **2b** as a solution in C_6D_6 at ambient temperature.

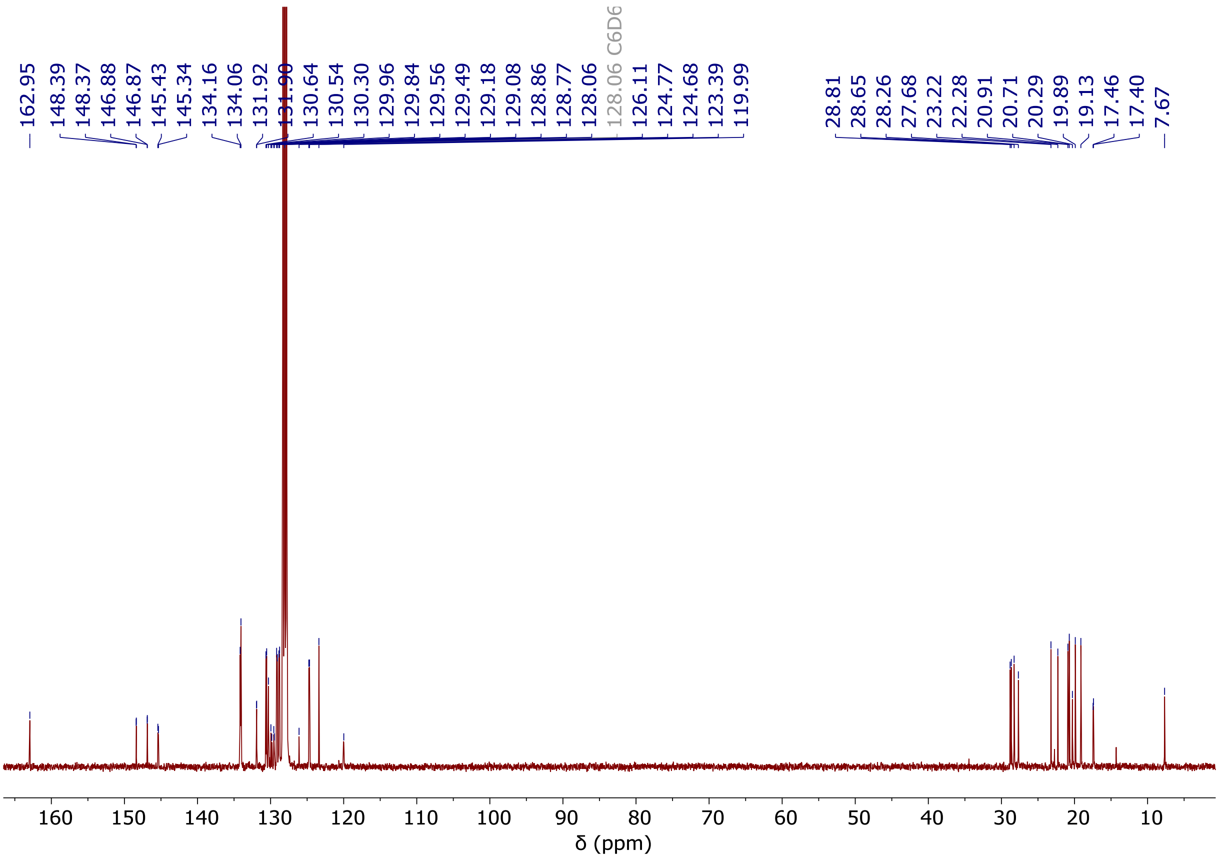

Figure S3. $^{13}C\{^1H\}$ NMR spectrum of **2b** as a solution in C_6D_6 at ambient temperature.

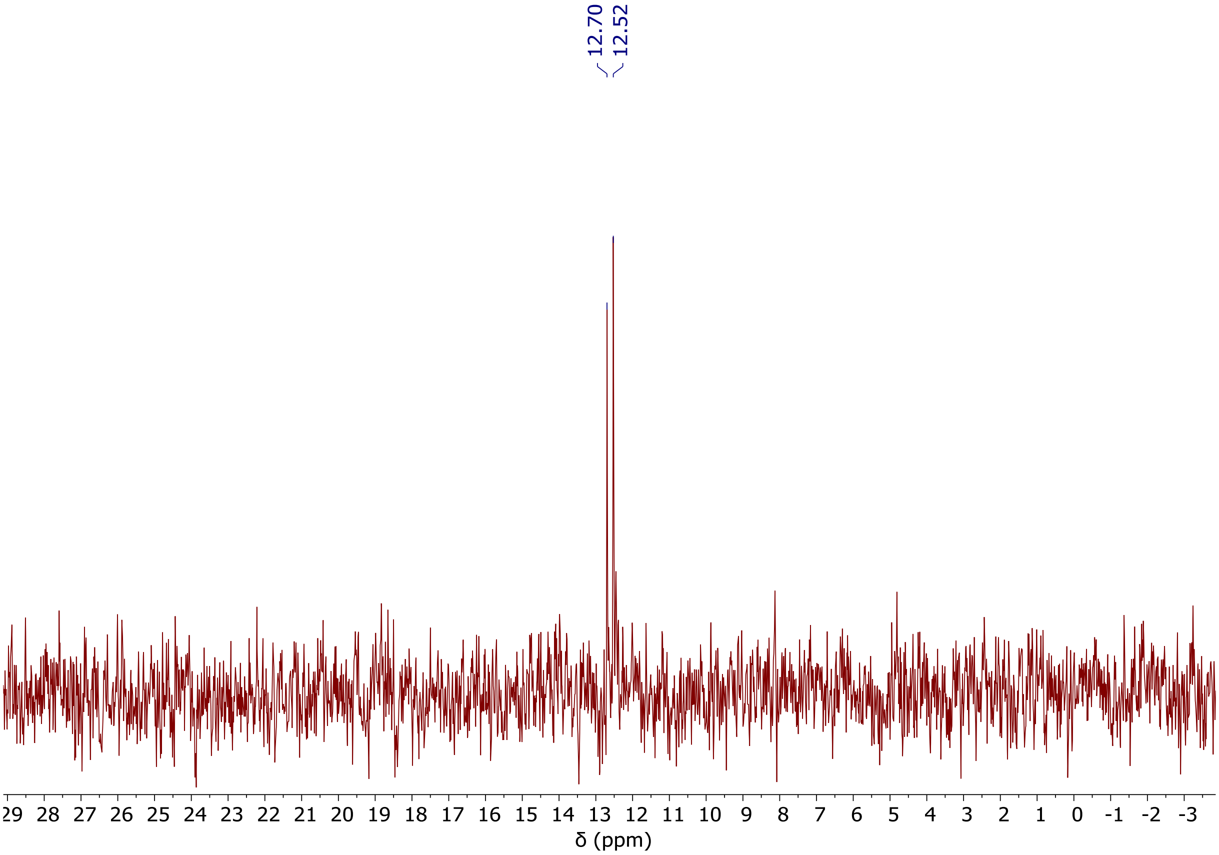

Figure S4. $^{29}Si\{^1H\}$ NMR spectrum of **2b** as a solution in C_6D_6 at ambient temperature.

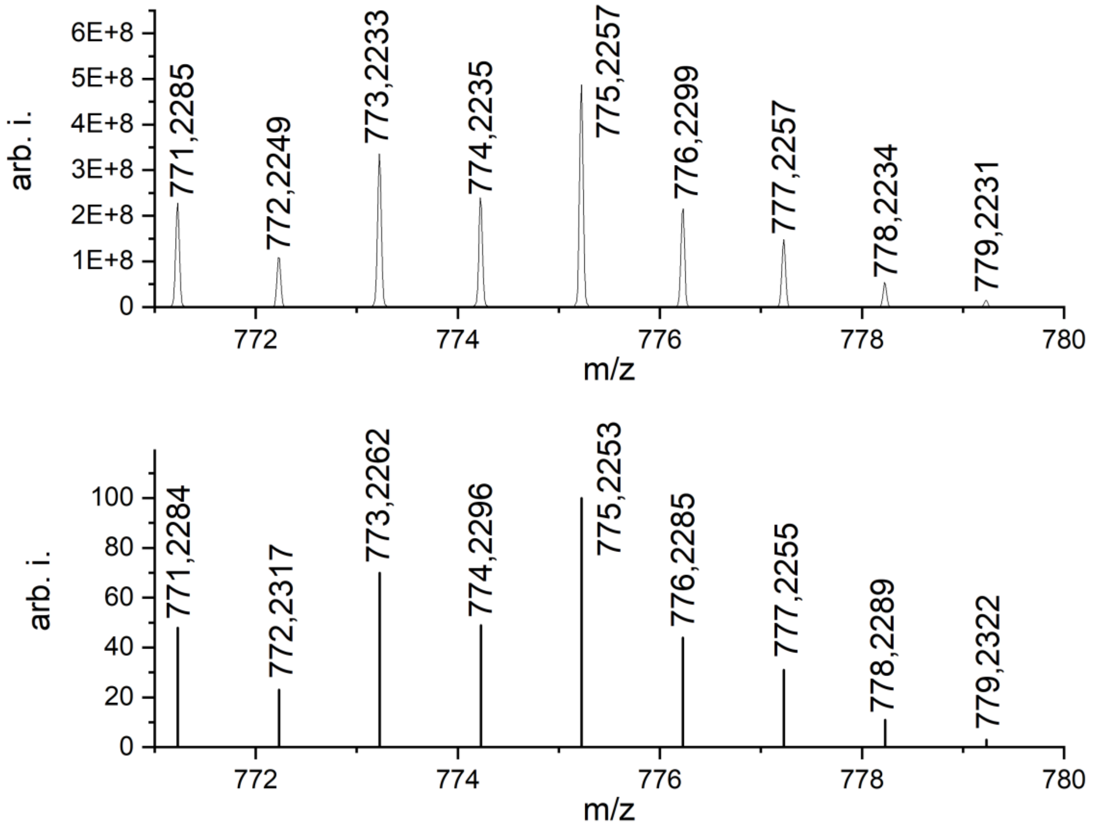

Figure S5. *Top*: Cutout from LIFDI/MS of **2b**; *Bottom*: Calculated MS spectrum of [**2b**]$^+$.

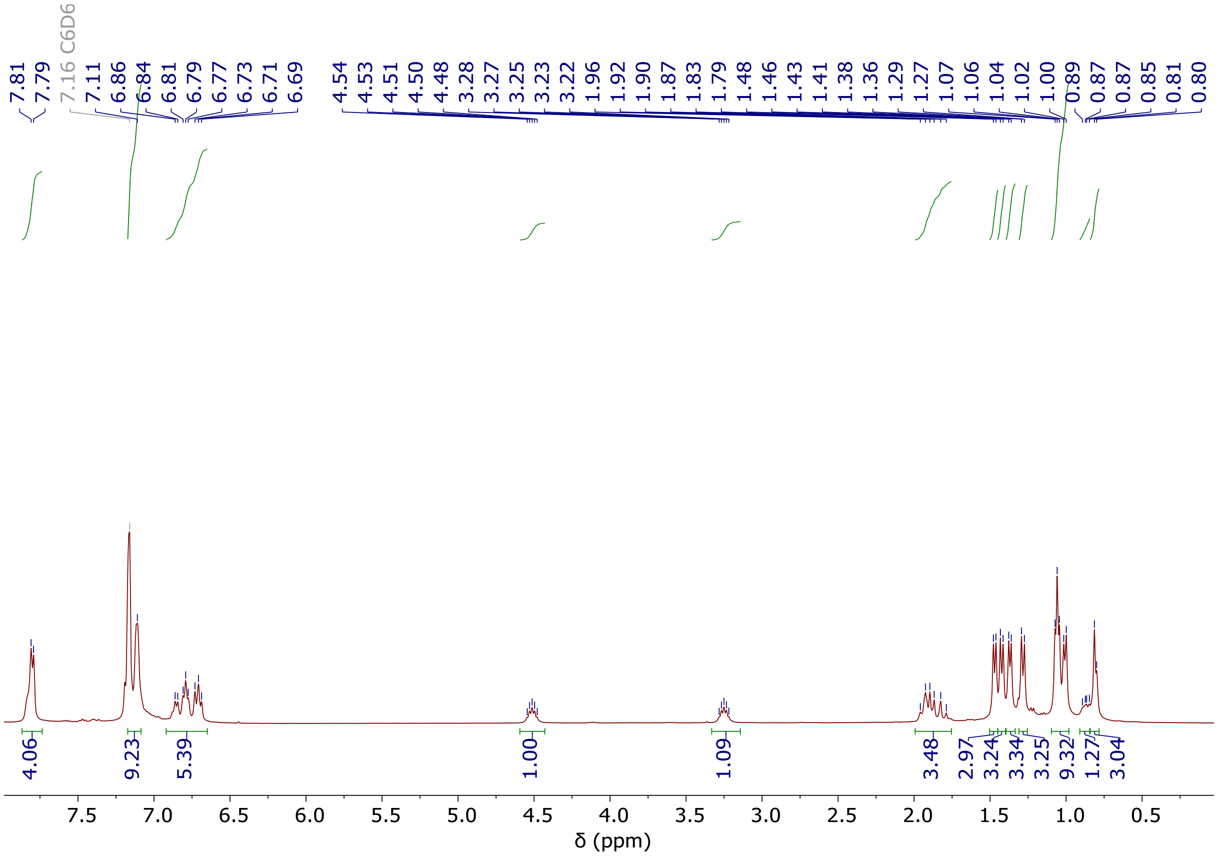

Figure S6. ^{1}H NMR spectrum of **2c** as a solution in C_6D_6 at ambient temperature.

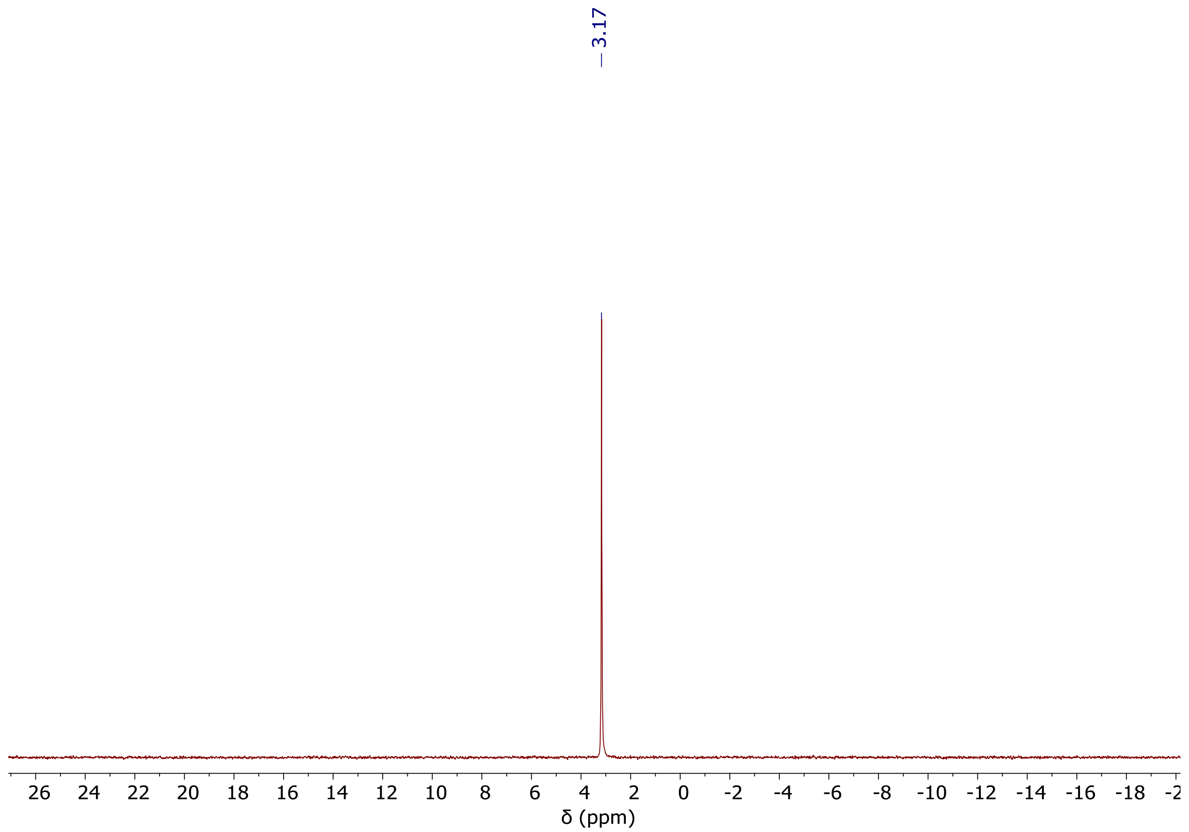

Figure S7. $^{31}P\{^{1}H\}$ NMR spectrum of **2c** as a solution in C_6D_6 at ambient temperature.

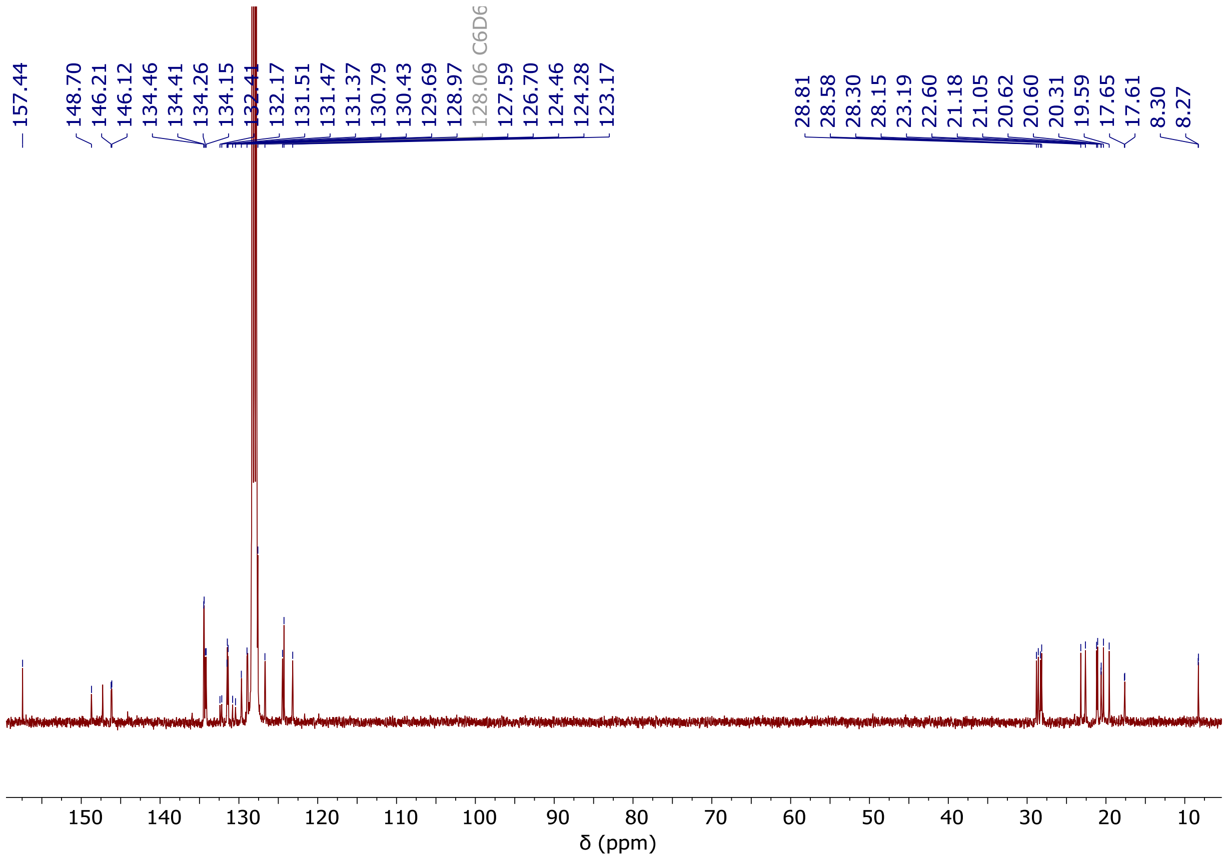

Figure S8. $^{13}C\{^{1}H\}$ NMR spectrum of **2c** as a solution in C_6D_6 at ambient temperature.

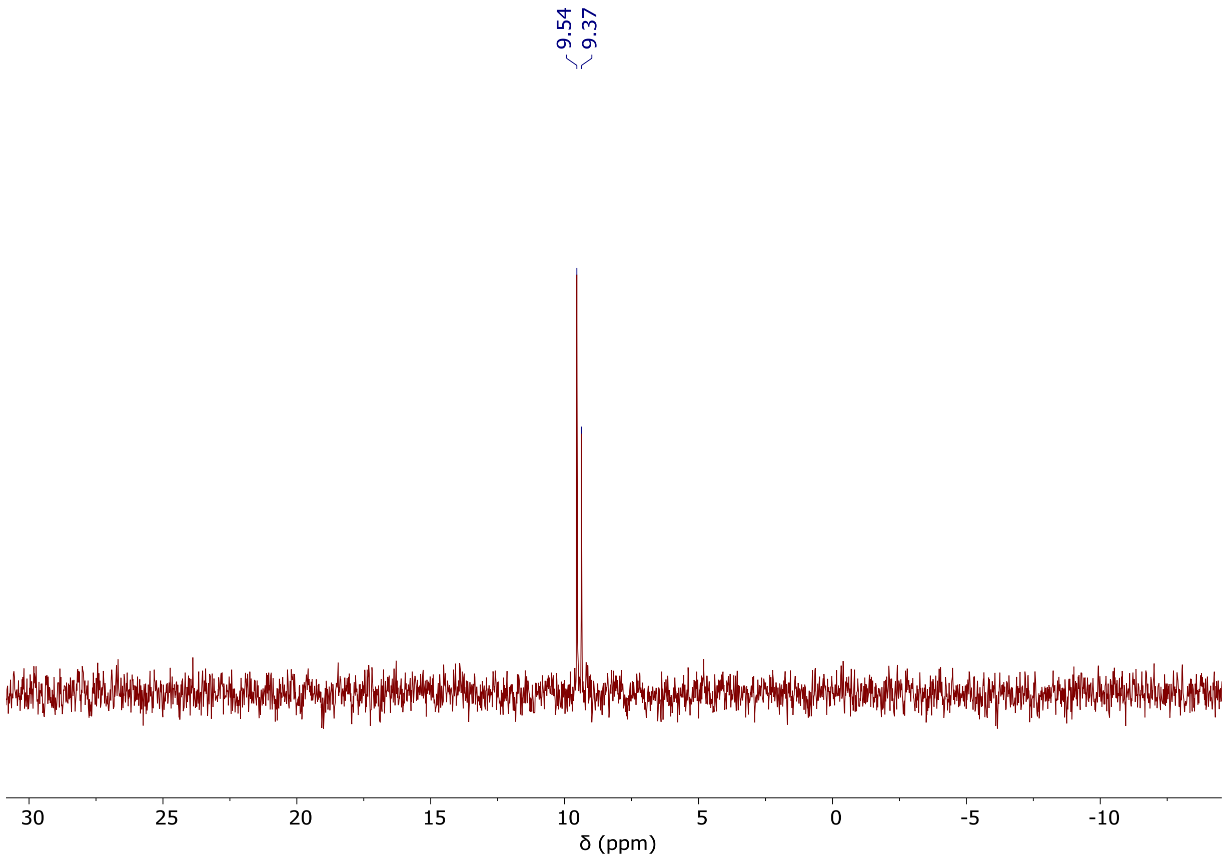

Figure S9. $^{29}Si\{^1H\}$ NMR spectrum of **2c** as a solution in C_6D_6 at ambient temperature.

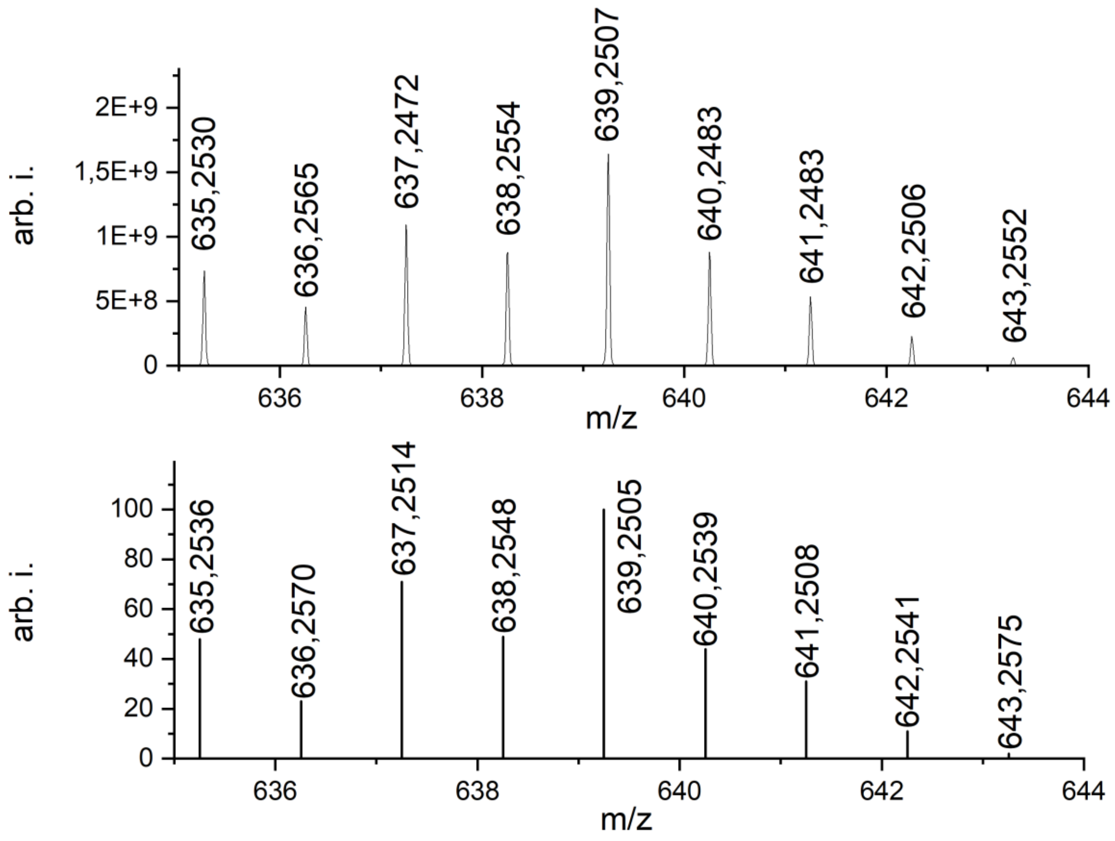

Figure S10. *Top*: Cutout from LIFDI/MS of **2c**; *Bottom*: Calculated MS spectrum of [**2c**]$^+$.

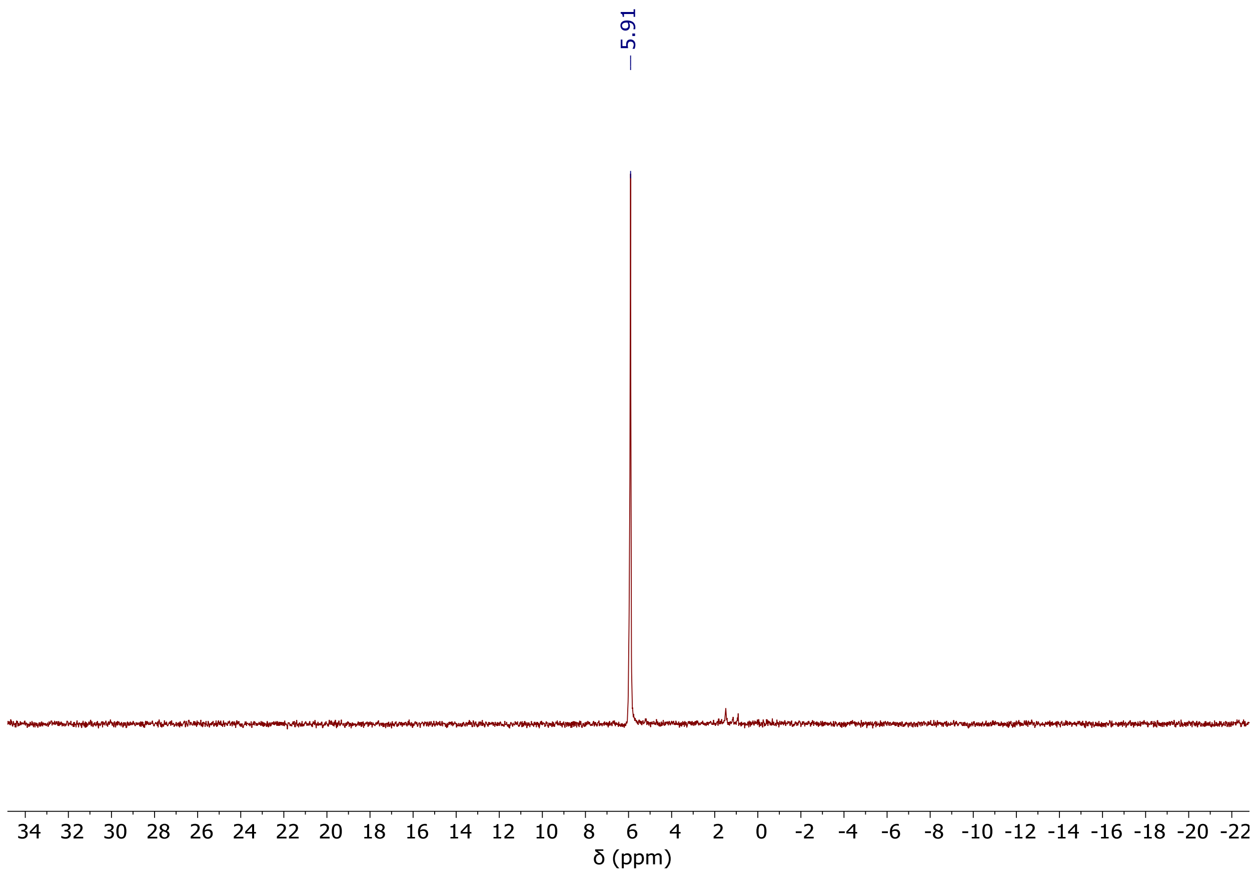

Figure S11. Crude $^{31}P\{^{1}H\}$ NMR spectrum of **2a** from the reaction mixture at ambient temperature.

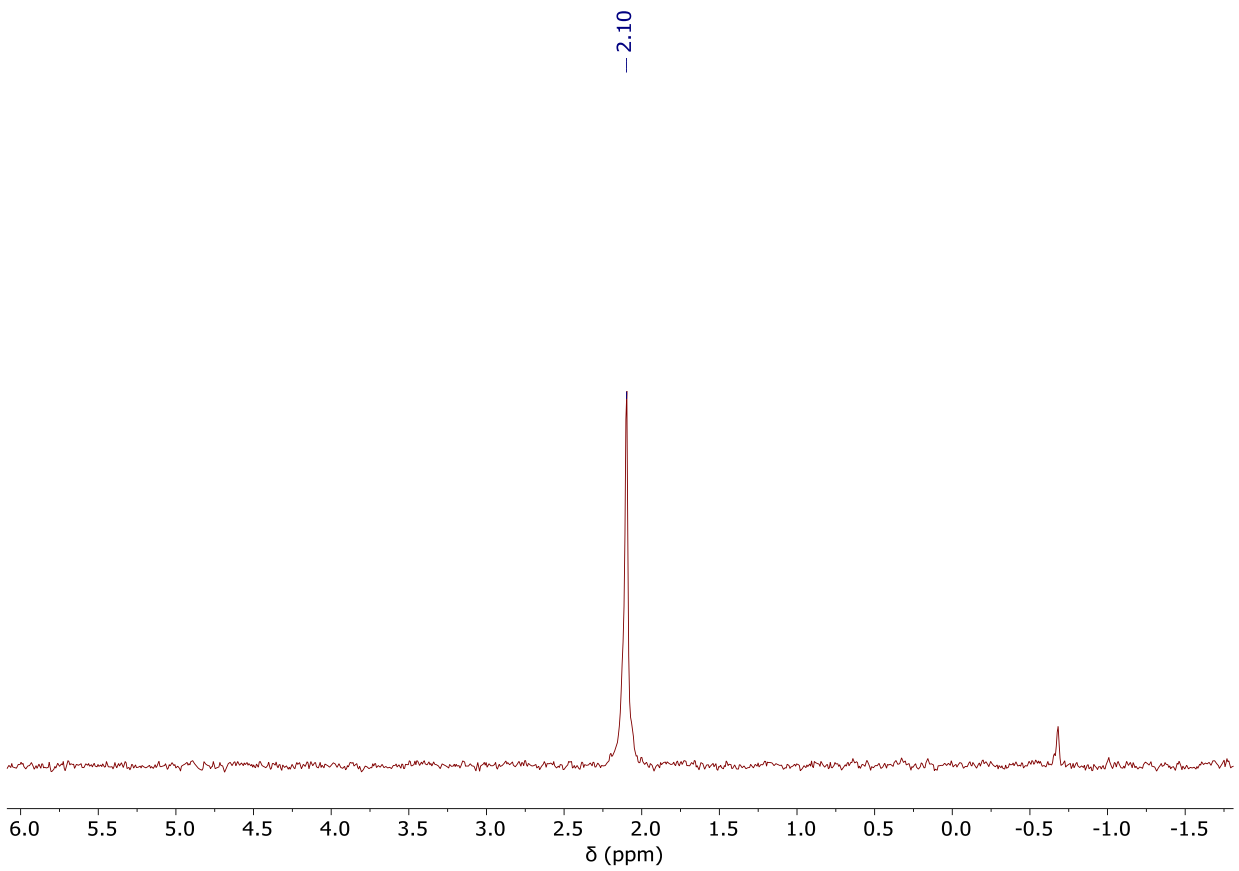

Figure S12. Crude $^{31}P\{^{1}H\}$ NMR spectrum of **2d** from the reaction mixture at ambient temperature.

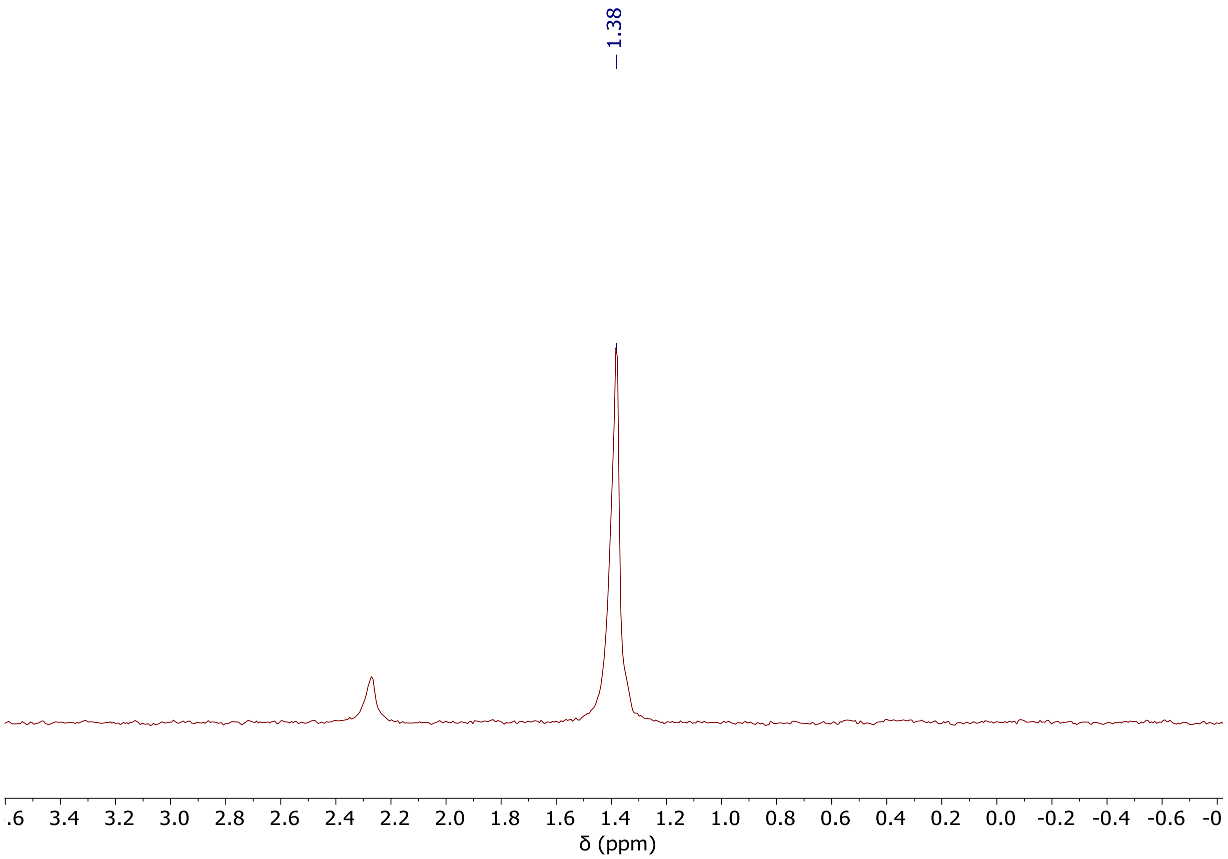

Figure S13. Crude $^{31}P\{^{1}H\}$ NMR spectrum of **2e** from the reaction mixture at ambient temperature.

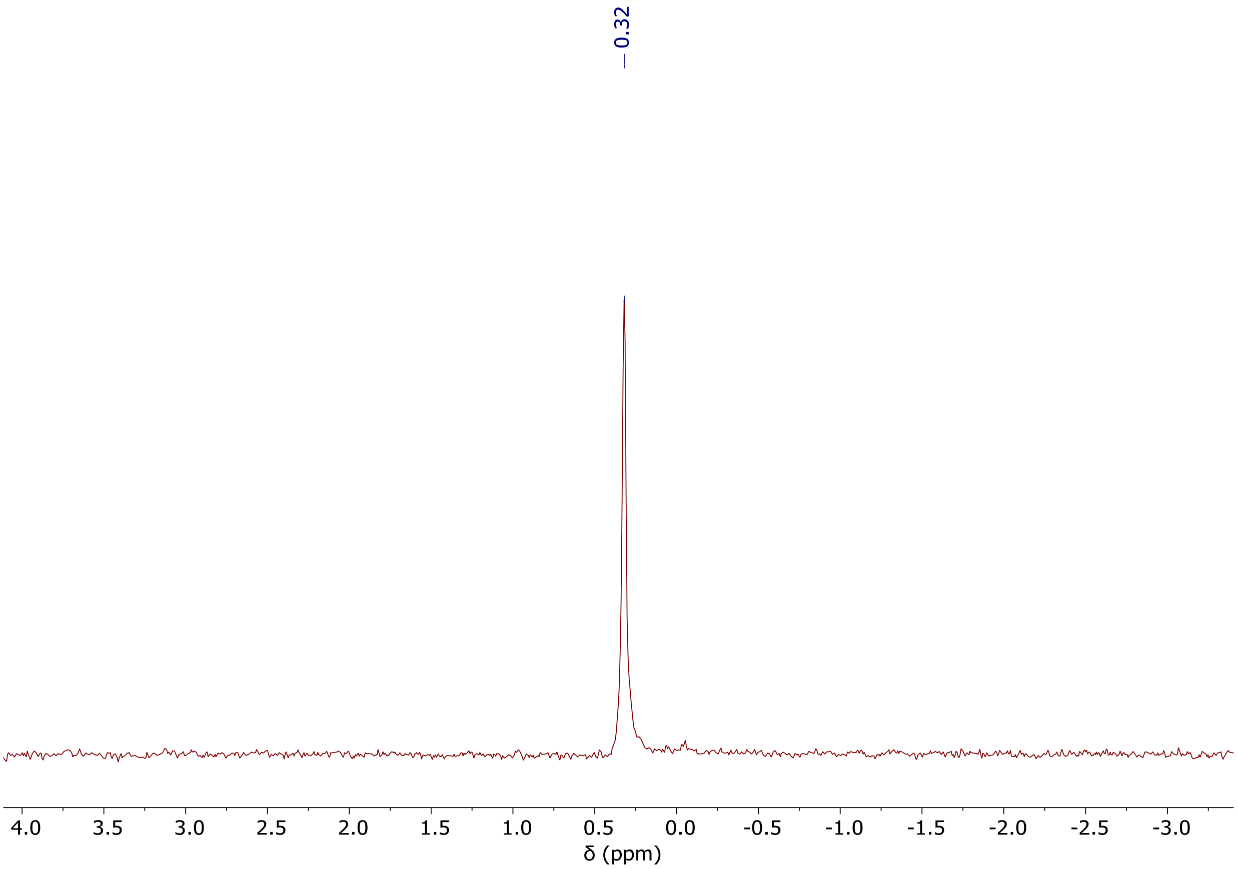

Figure S14. Crude $^{31}P\{^{1}H\}$ NMR spectrum of **2f** from the reaction mixture at ambient temperature.

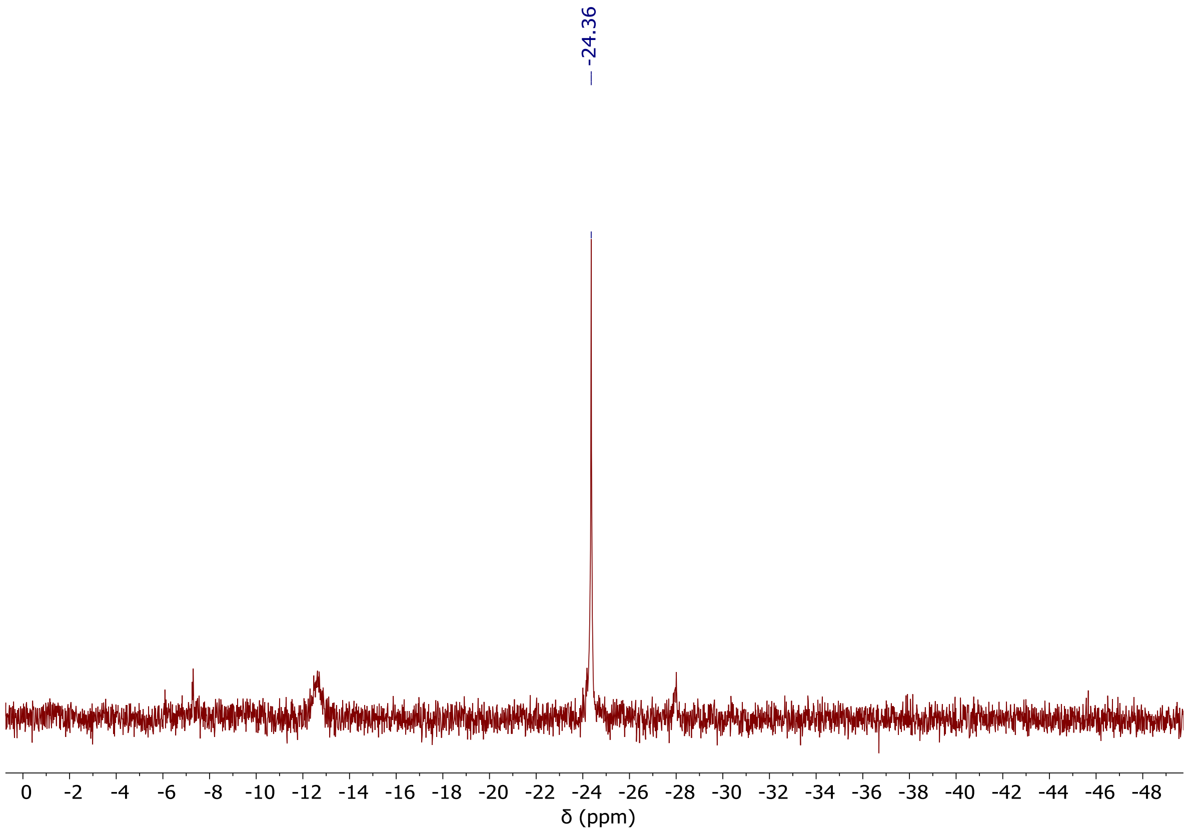

Figure S15. Crude $^{31}P\{^{1}H\}$ NMR spectrum of **2g** from the reaction mixture at ambient temperature.

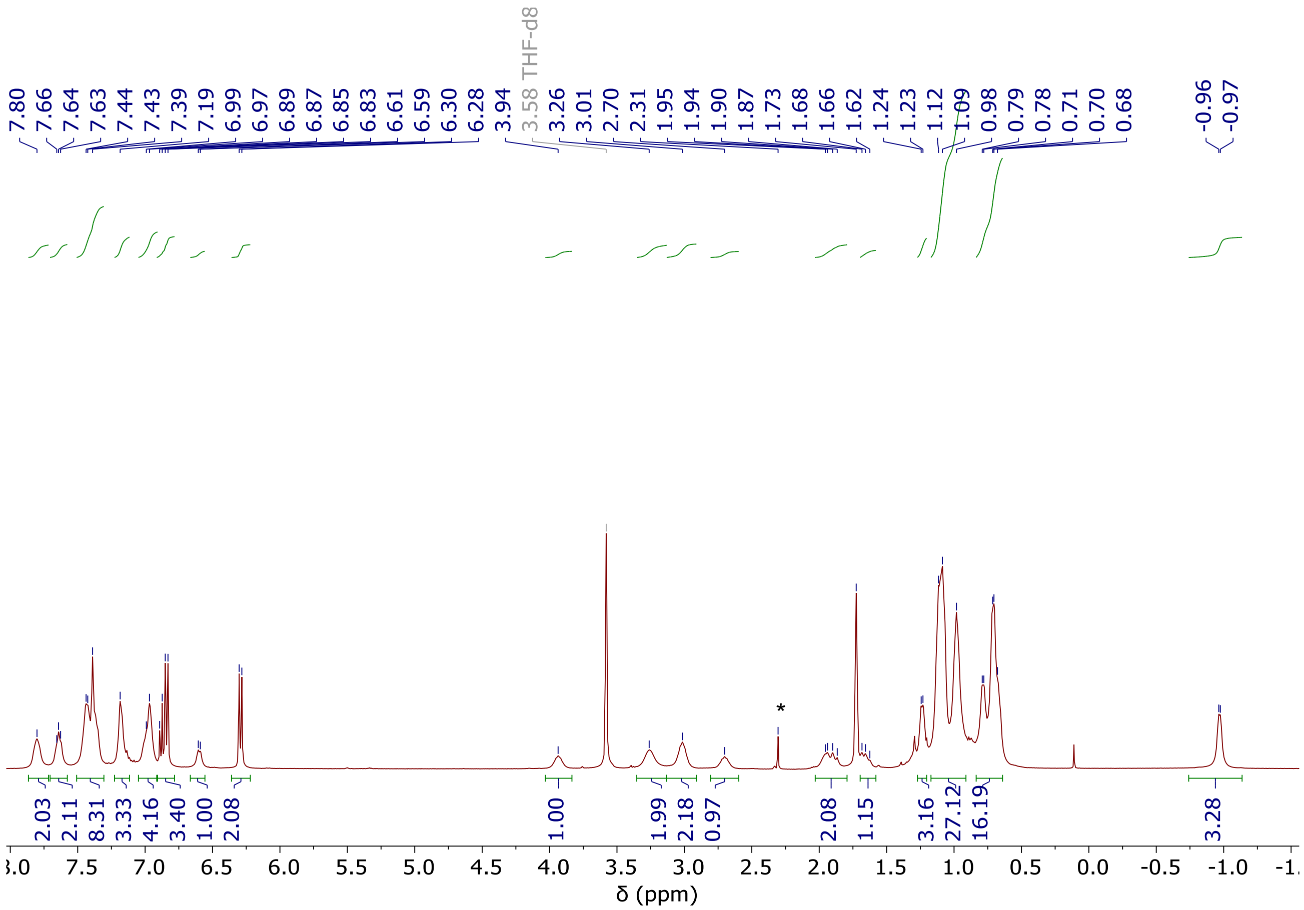

Figure S16. ^{1}H NMR spectrum of **3a** as a solution in C_6D_6 at ambient temperature;* indicates minor amounts of toluene.

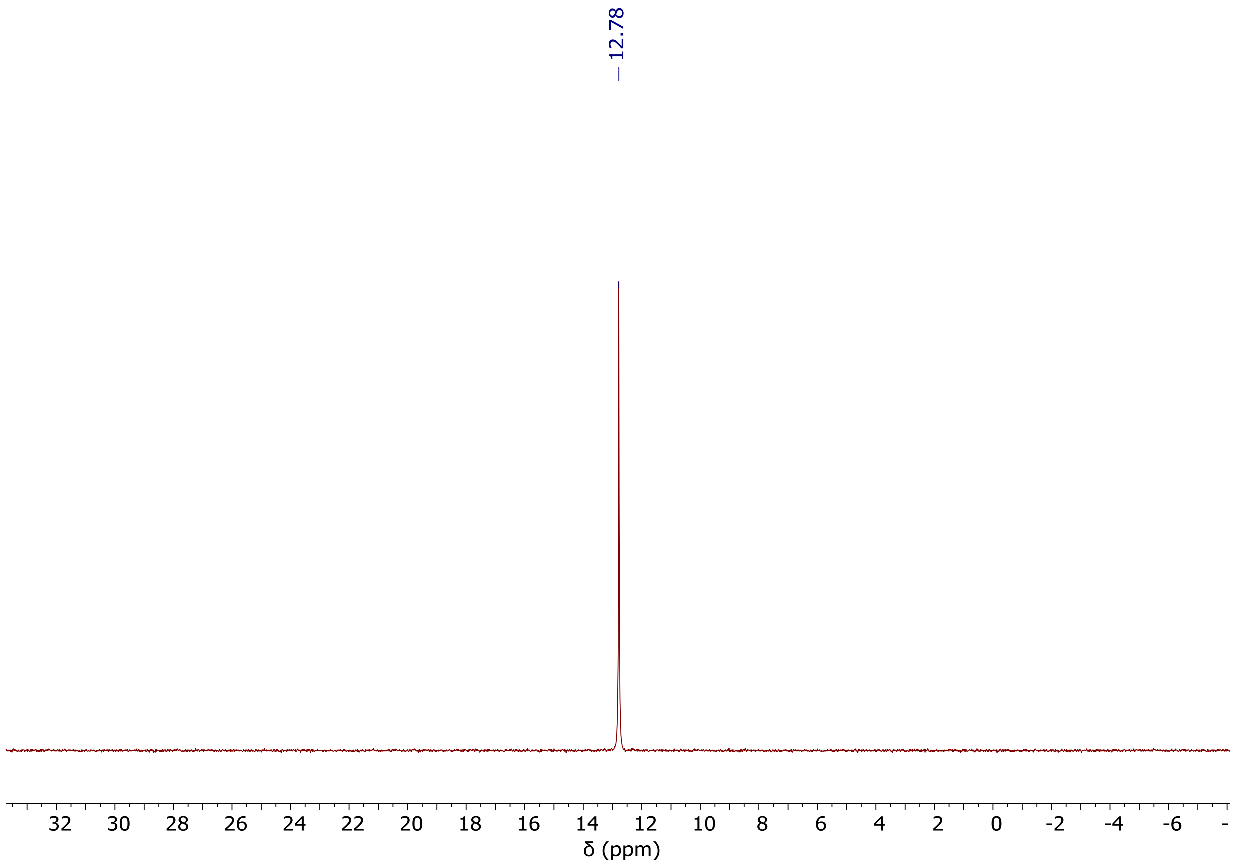

Figure S17. $^{31}P\{^1H\}$ NMR spectrum of **3a** as a solution in C_6D_6 at ambient temperature.

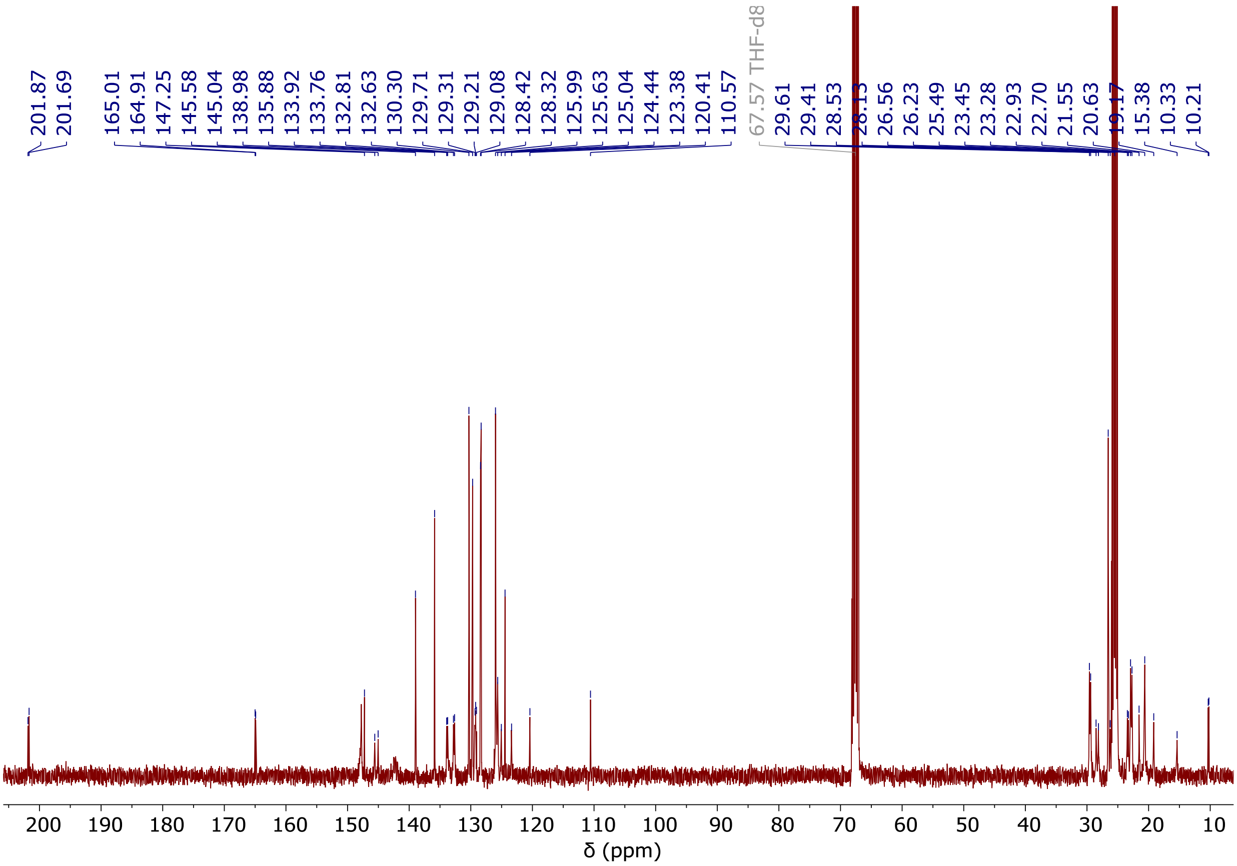

Figure S18. $^{13}C\{^1H\}$ NMR spectrum of **3a** as a solution in C_6D_6 at ambient temperature.

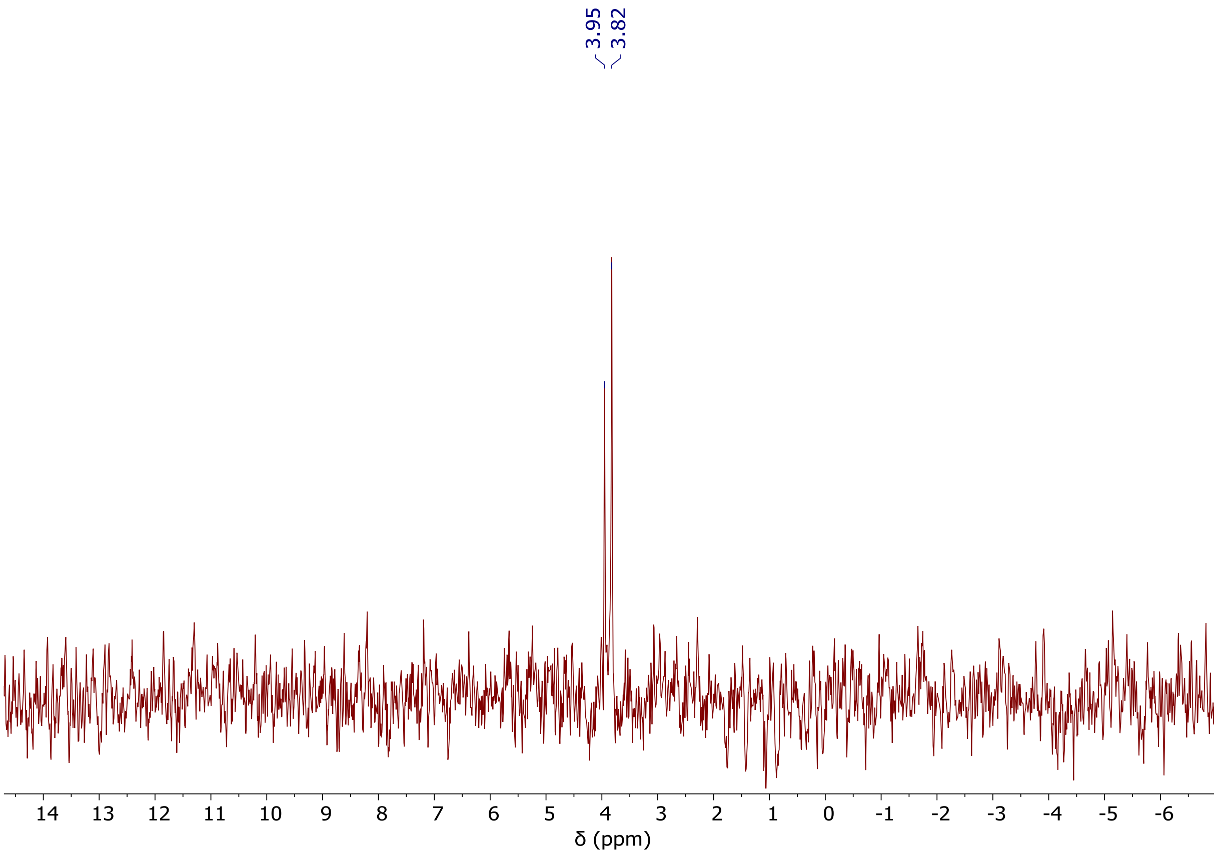

Figure S19. $^{29}Si\{^{1}H\}$ NMR spectrum of **3a** as a solution in C_6D_6 at ambient temperature.

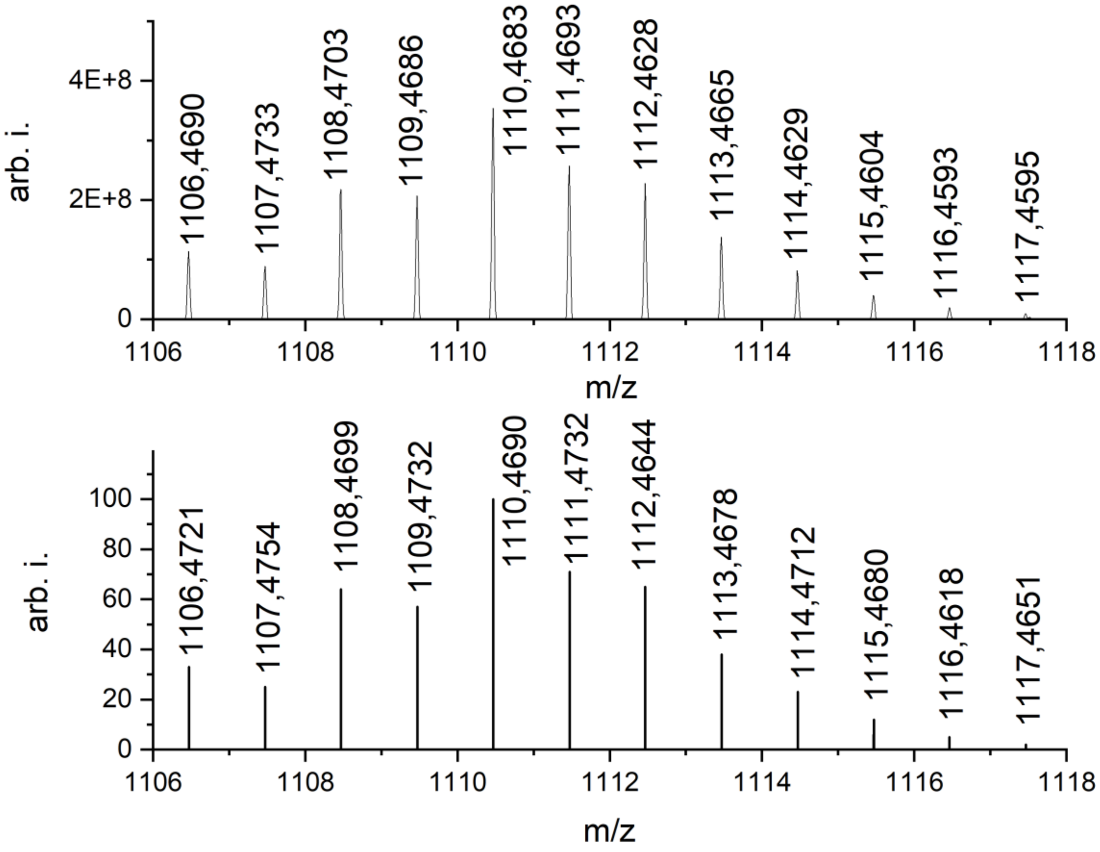

Figure S20. *Top*: Cutout from LIFDI/MS of **3a**; *Bottom*: Calculated MS spectrum of [**3a**]$^+$.

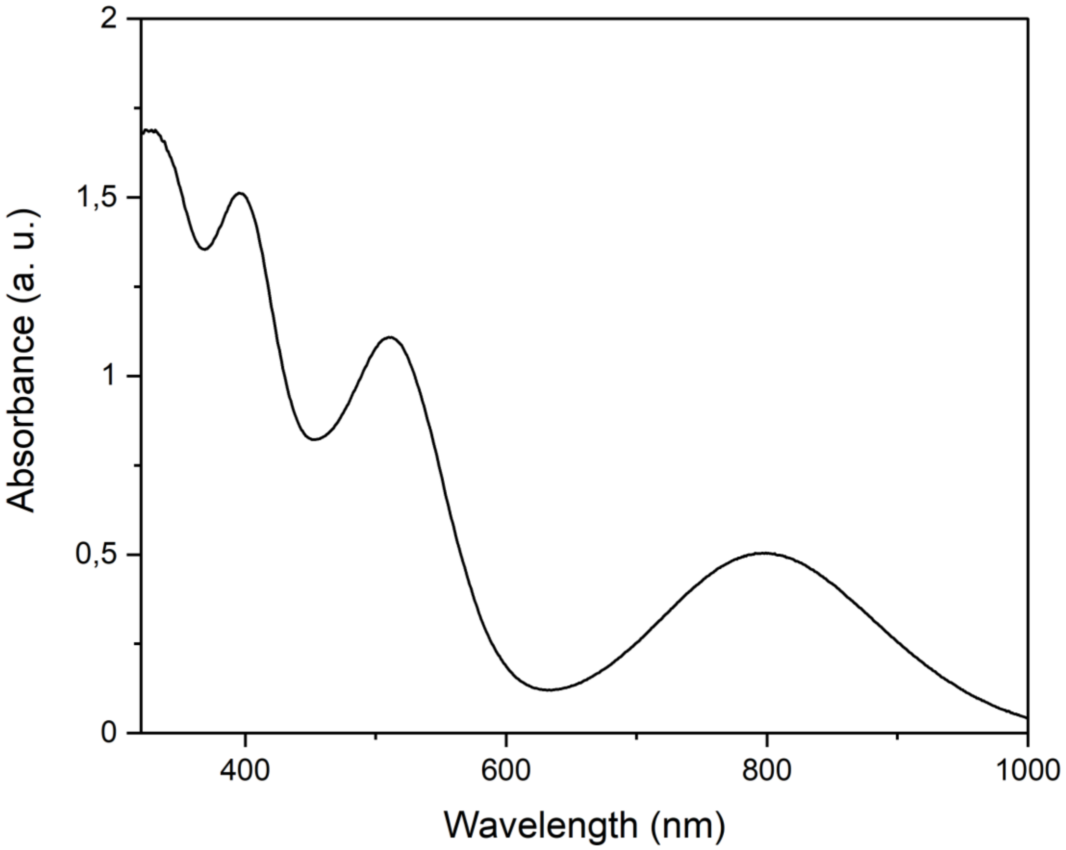

Figure S21. UV/vis spectrum of a 1.0x10^{-4} M solution of **3a** in toluene at ambient temperature.

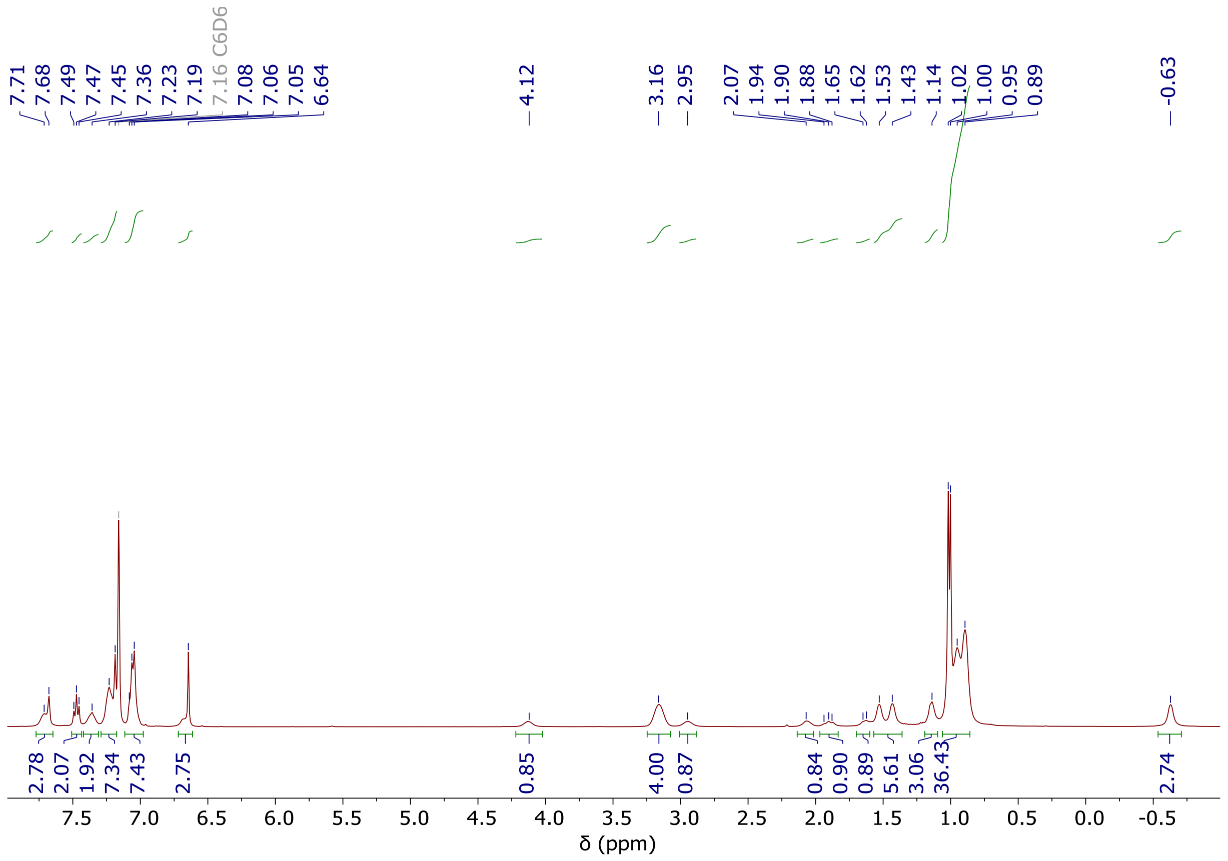

Figure S22. ^{1}H NMR spectrum of **3b** as a solution in C_6D_6 at ambient temperature.

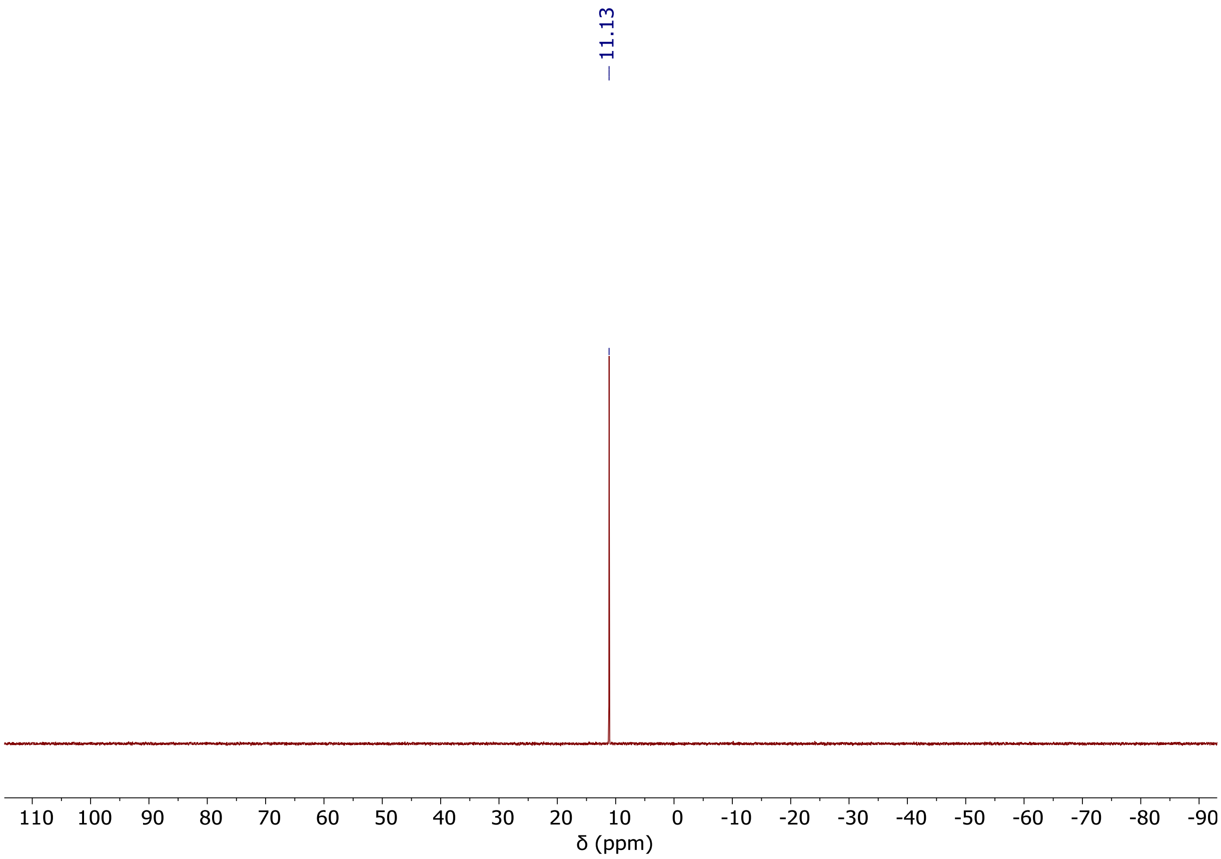

Figure S23. $^{31}P\{^1H\}$ NMR spectrum of **3b** as a solution in C_6D_6 at ambient temperature.

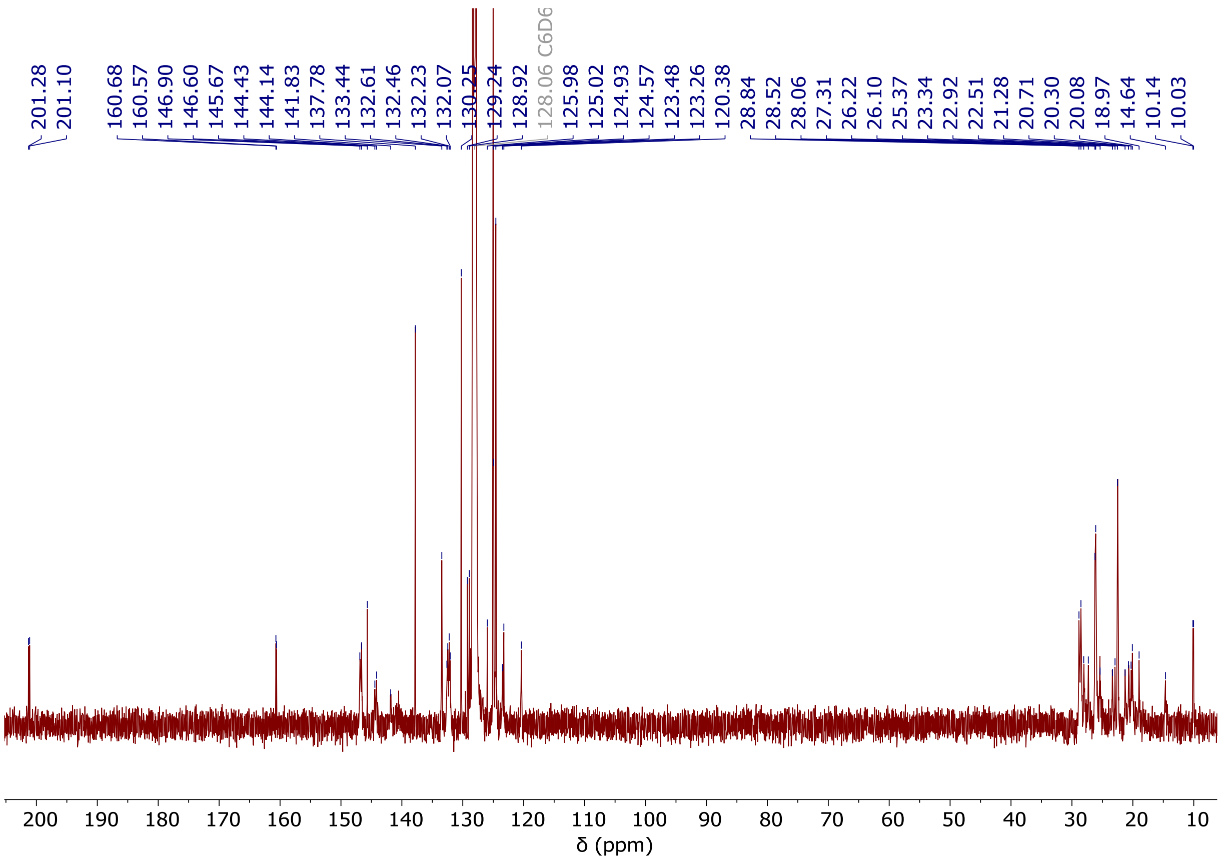

Figure S24. $^{13}C\{^1H\}$ NMR spectrum of **3b** as a solution in C_6D_6 at ambient temperature.

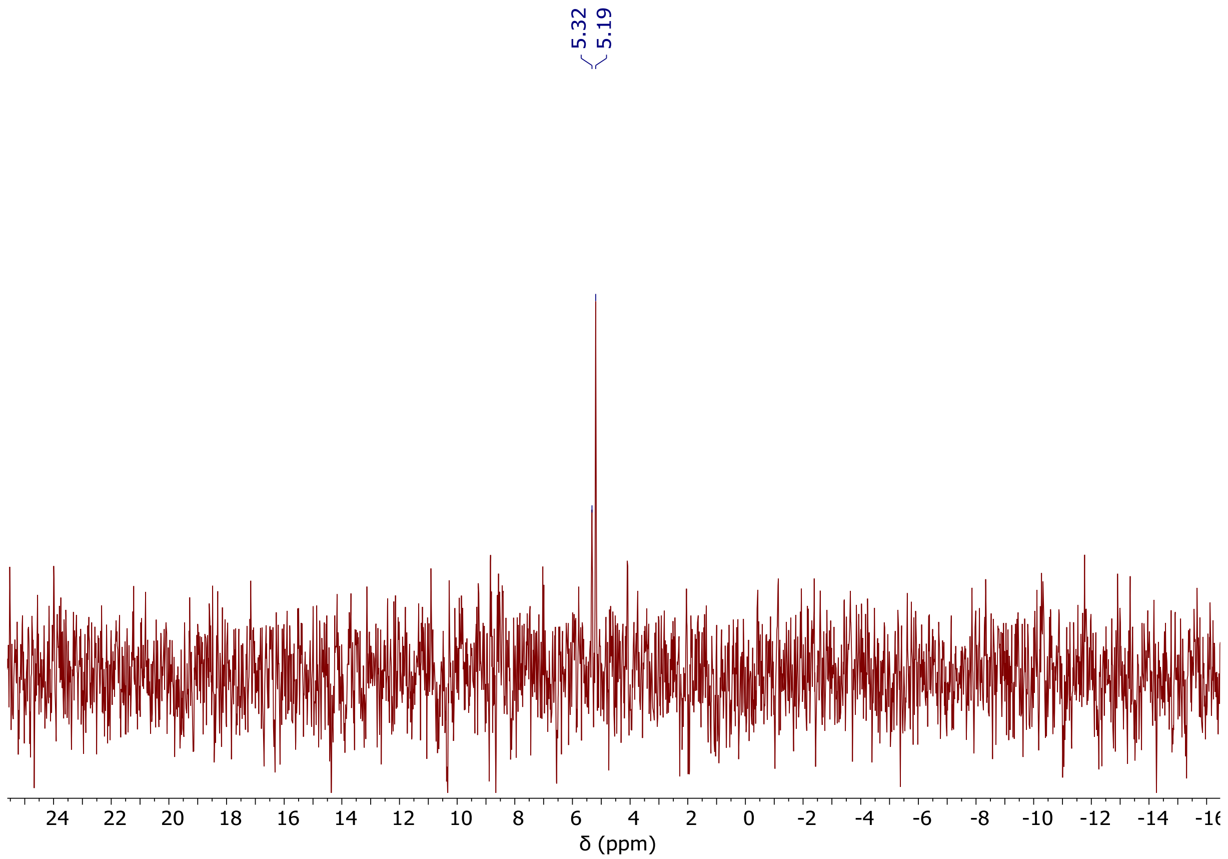

Figure S25. $^{29}Si\{^{1}H\}$ NMR spectrum of **3b** as a solution in C_6D_6 at ambient temperature.

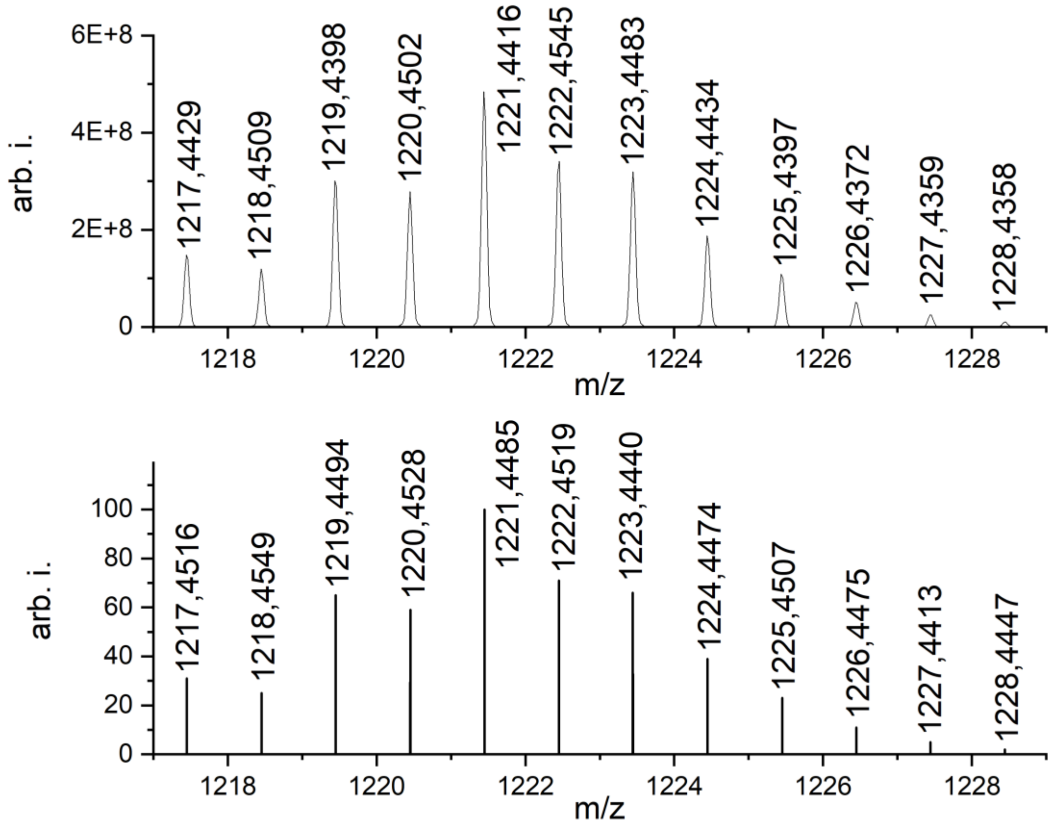

Figure S26. *Top*: Cutout from LIFDI/MS of **3b**; *Bottom*: Calculated MS spectrum of [**3b**]$^{+}$.

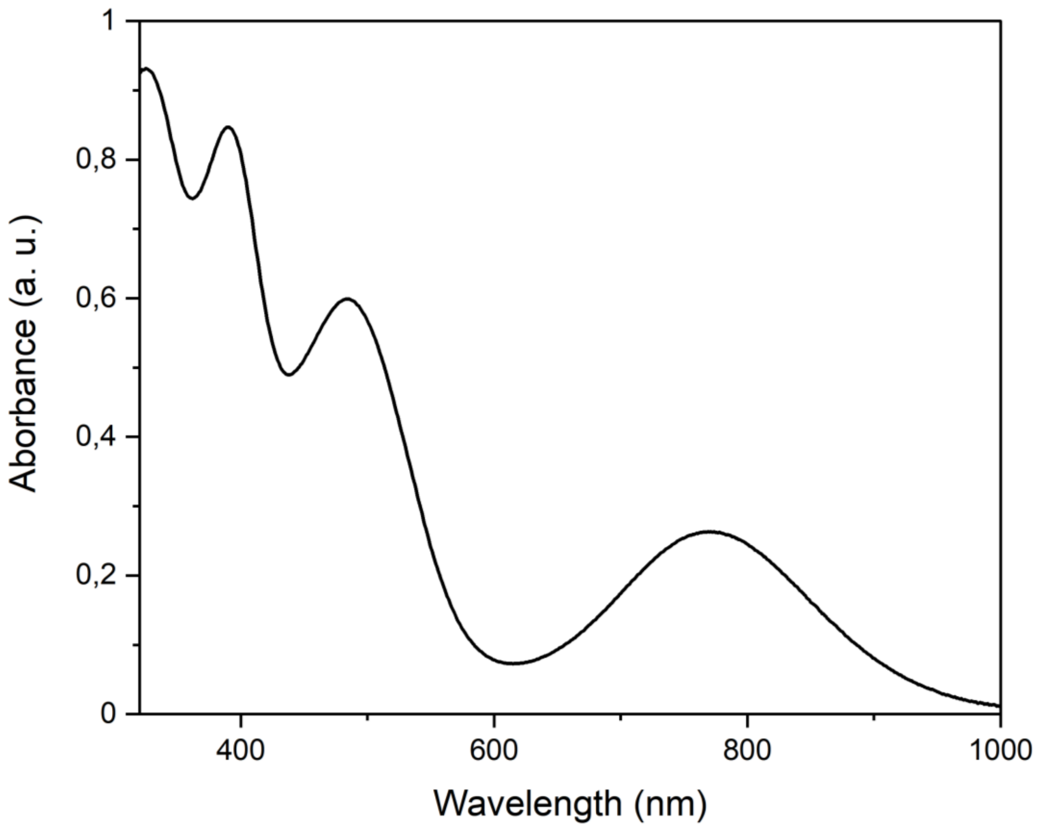

Figure S27. UV/vis spectrum of a 1.0x10^{-4} M solution of **3b** in toluene at ambient temperature.

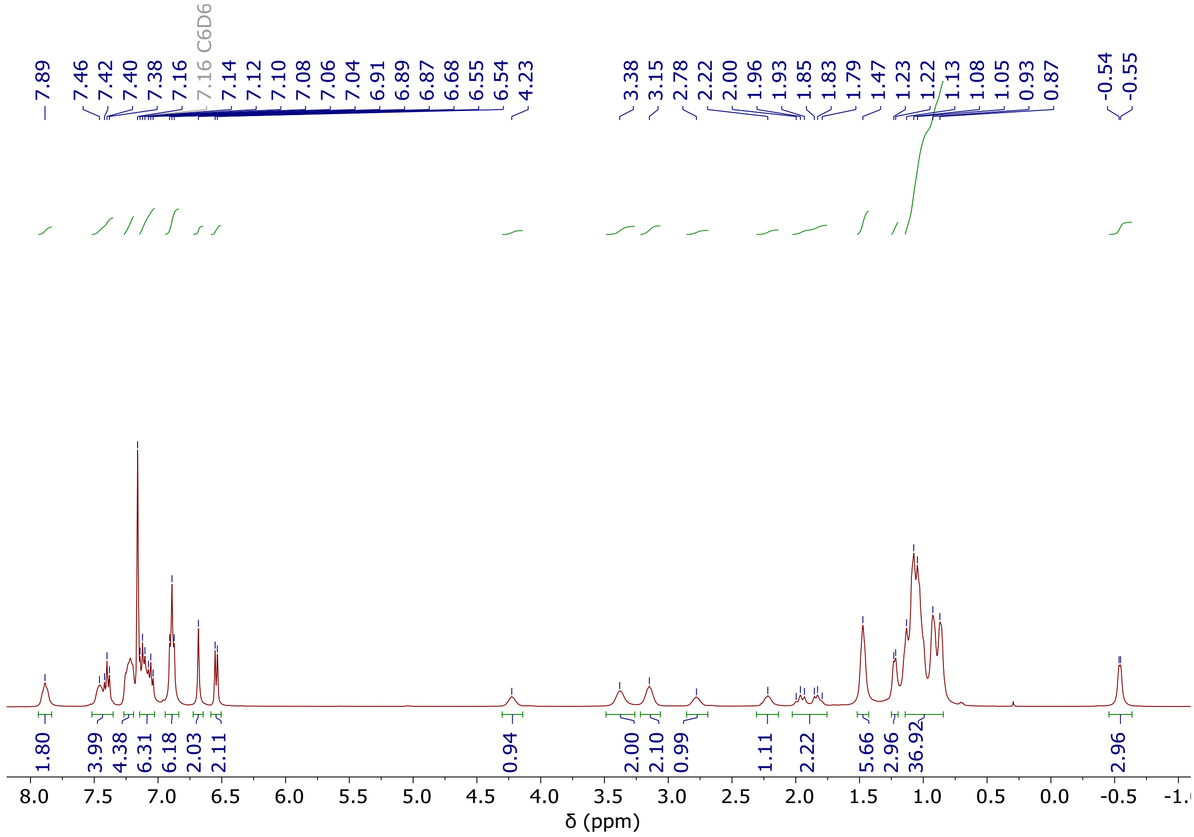

Figure S28. ^{1}H NMR spectrum of **3c** as a solution in C_6D_6 at ambient temperature.

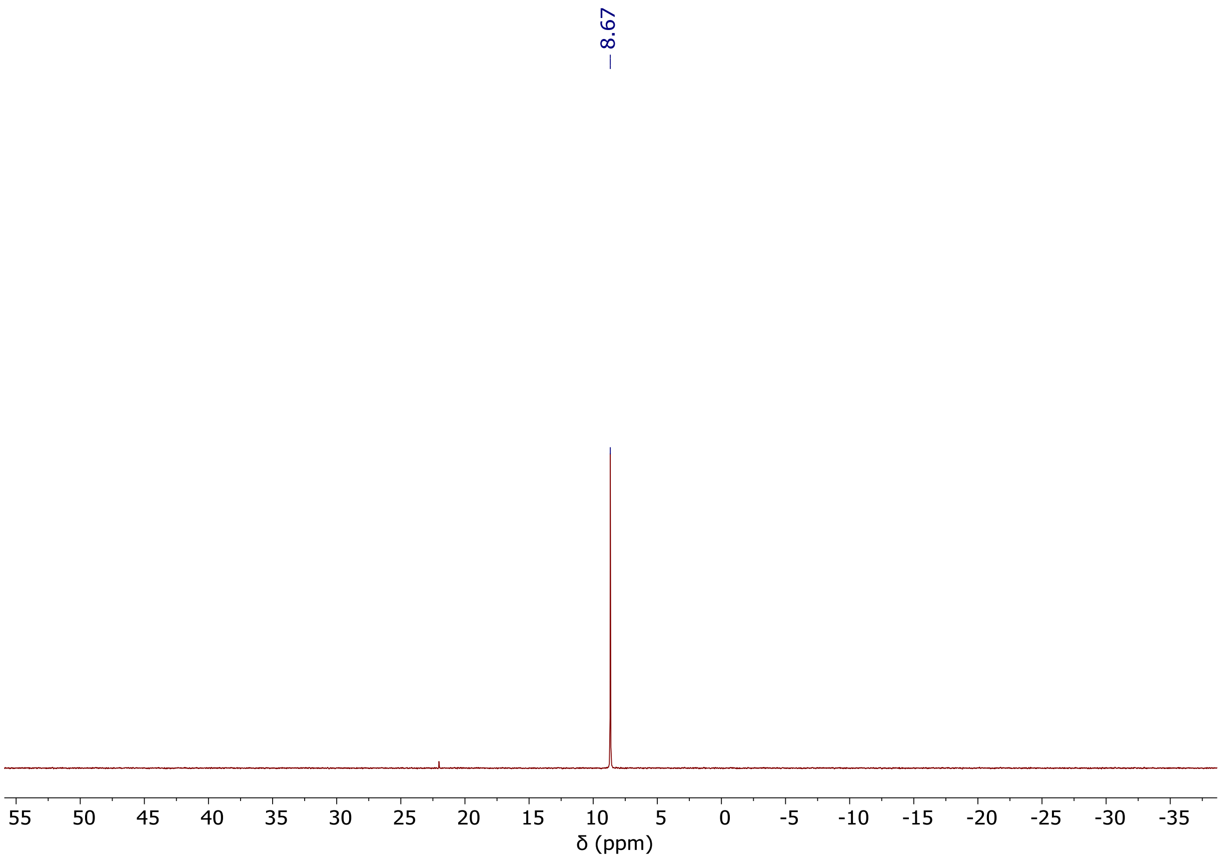

Figure S29. $^{31}P\{^1H\}$ NMR spectrum of **3c** as a solution in C_6D_6 at ambient temperature.

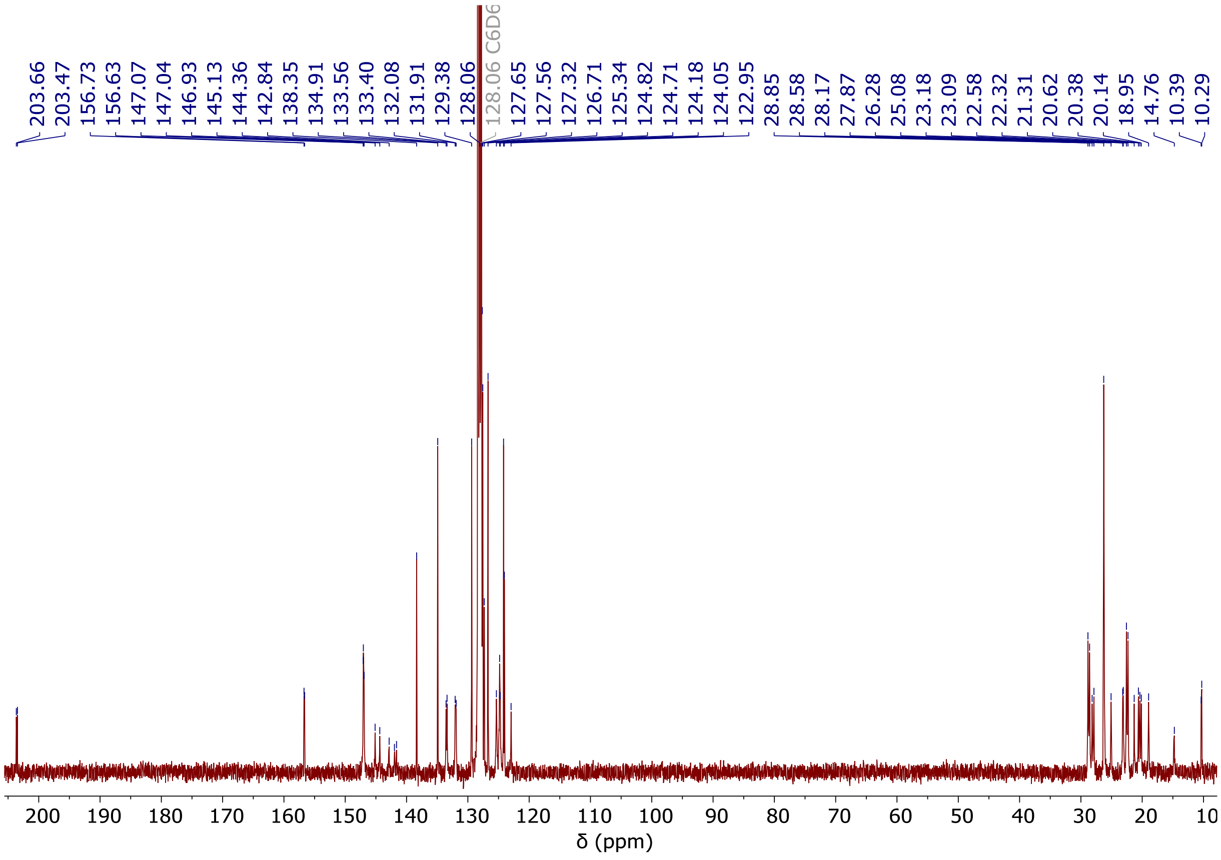

Figure S30. $^{13}C\{^1H\}$ NMR spectrum of **3c** as a solution in C_6D_6 at ambient temperature.

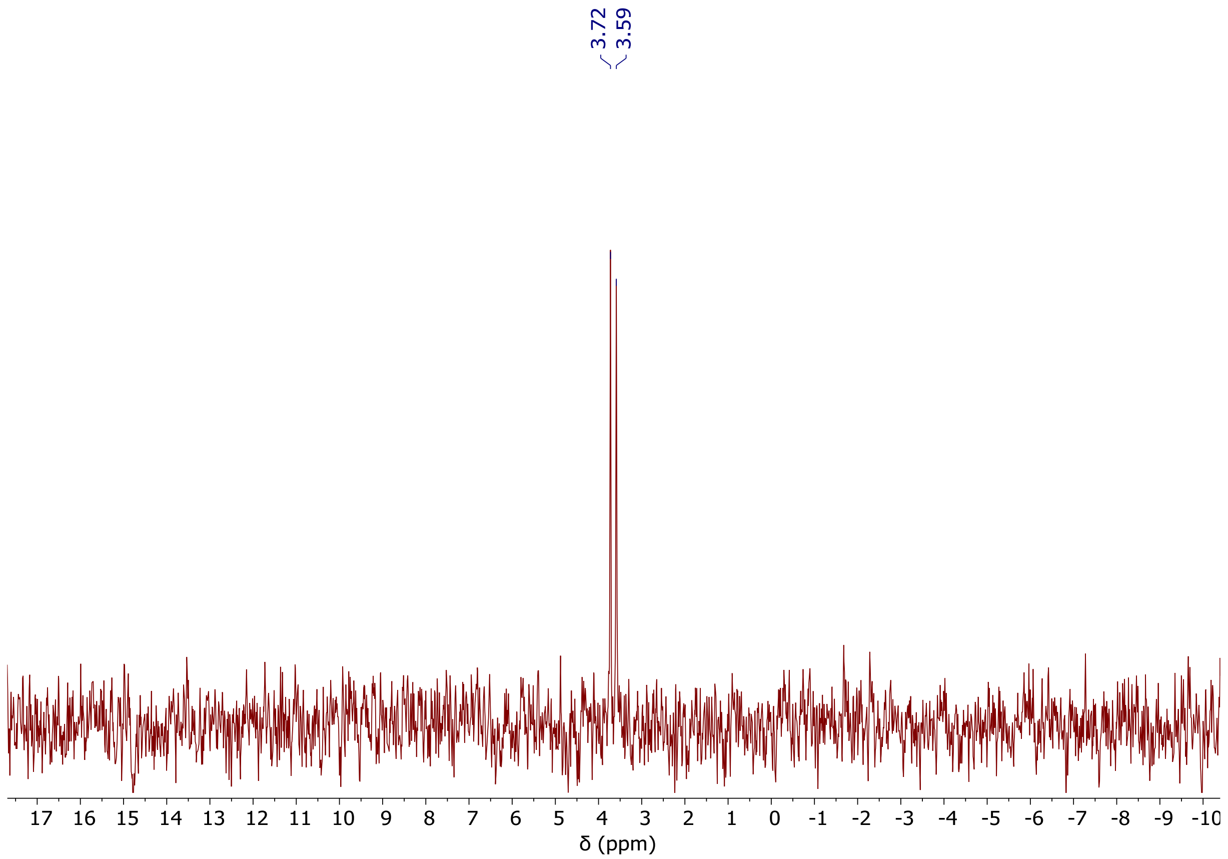

Figure S31. $^{29}Si\{^{1}H\}$ NMR spectrum of **3c** as a solution in C_6D_6 at ambient temperature.

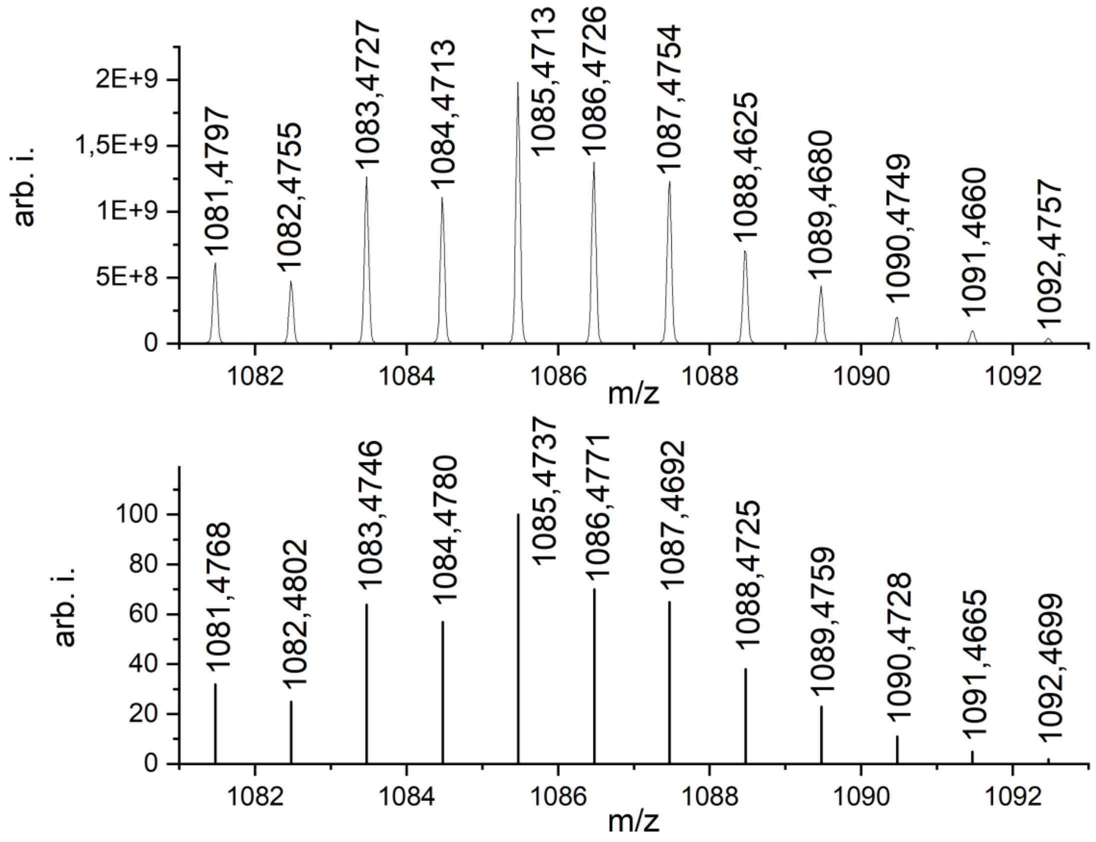

Figure S32. *Top*: Cutout from LIFDI/MS of **3c**; *Bottom*: Calculated MS spectrum of [**3c**]$^{+}$.

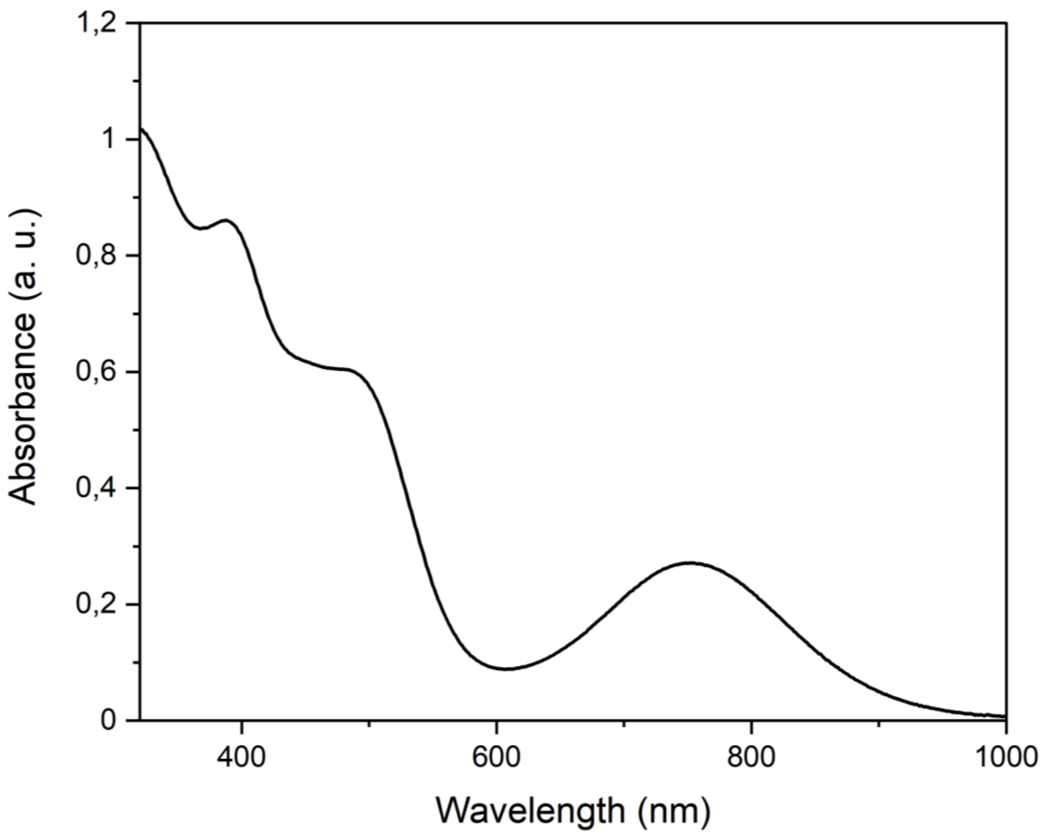

Figure S33. UV/vis spectrum of a $1.0x10^{-4}$ M solution of **3c** in toluene at ambient temperature.

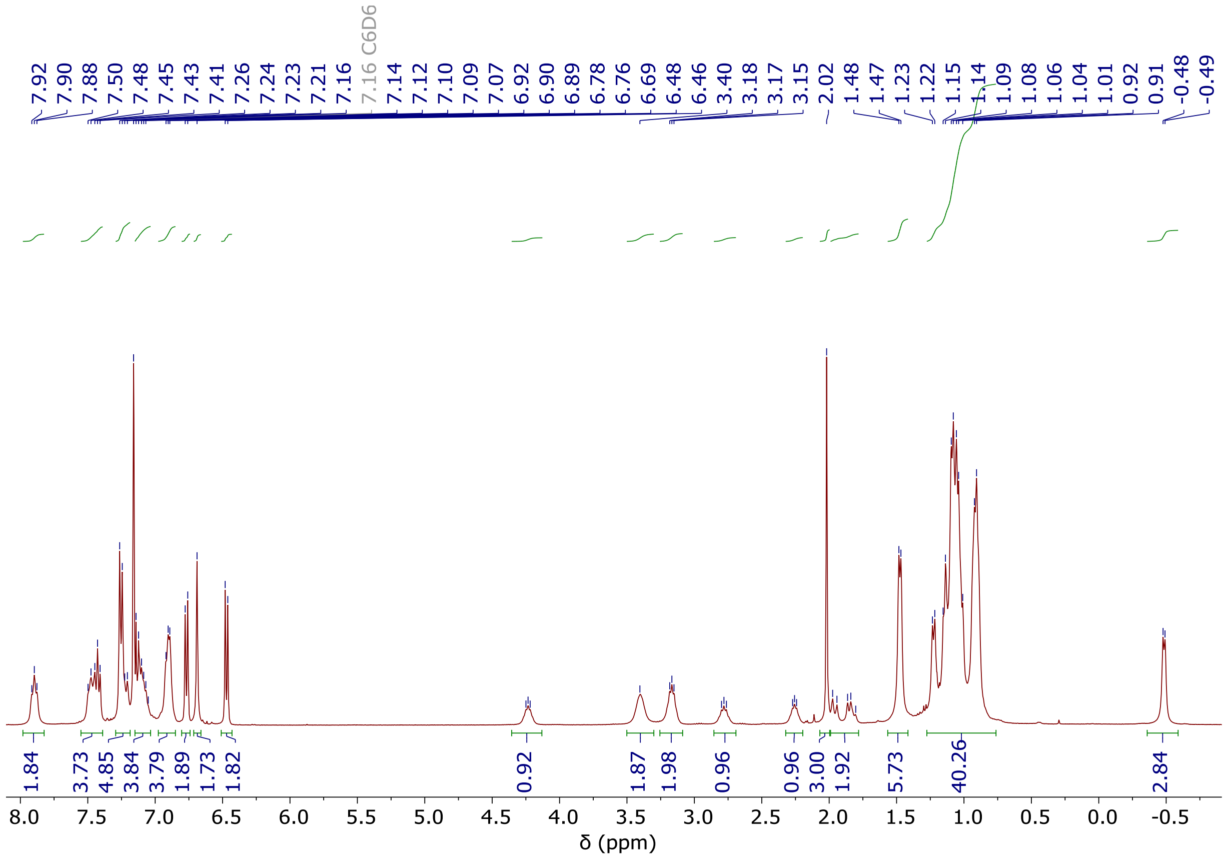

Figure S34. ^1H NMR spectrum of **3d** as a solution in C_6D_6 at ambient temperature.

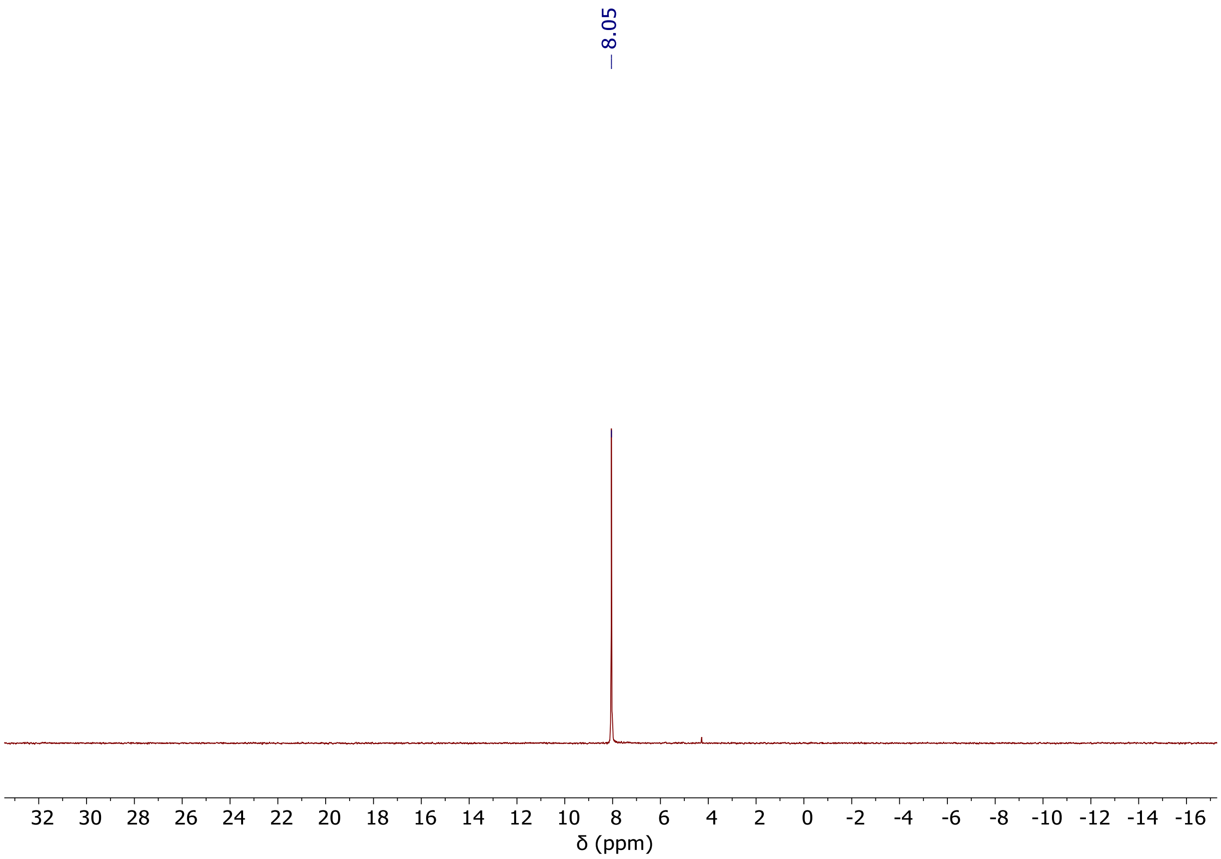

Figure S35. $^{31}P\{^1H\}$ NMR spectrum of **3d** as a solution in C_6D_6 at ambient temperature.

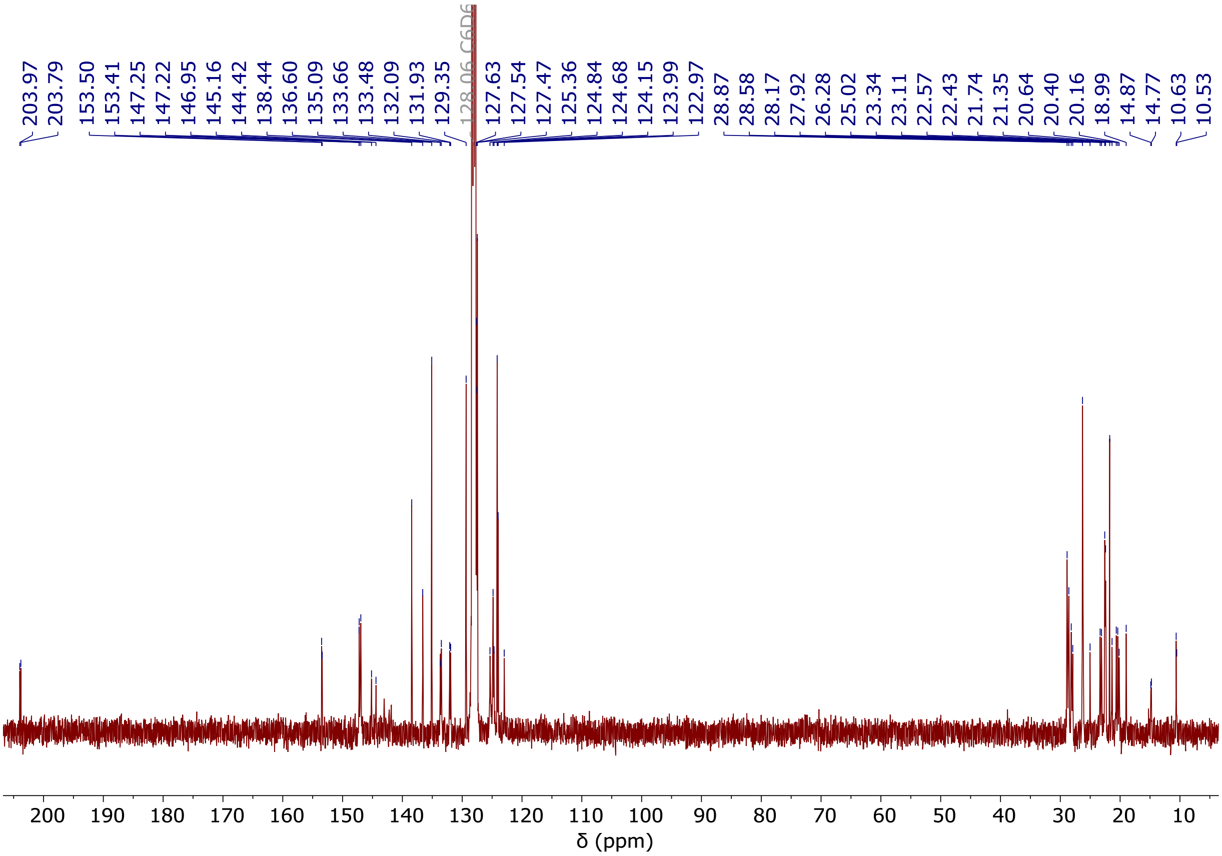

Figure S36. $^{13}C\{^1H\}$ NMR spectrum of **3d** as a solution in C_6D_6 at ambient temperature.

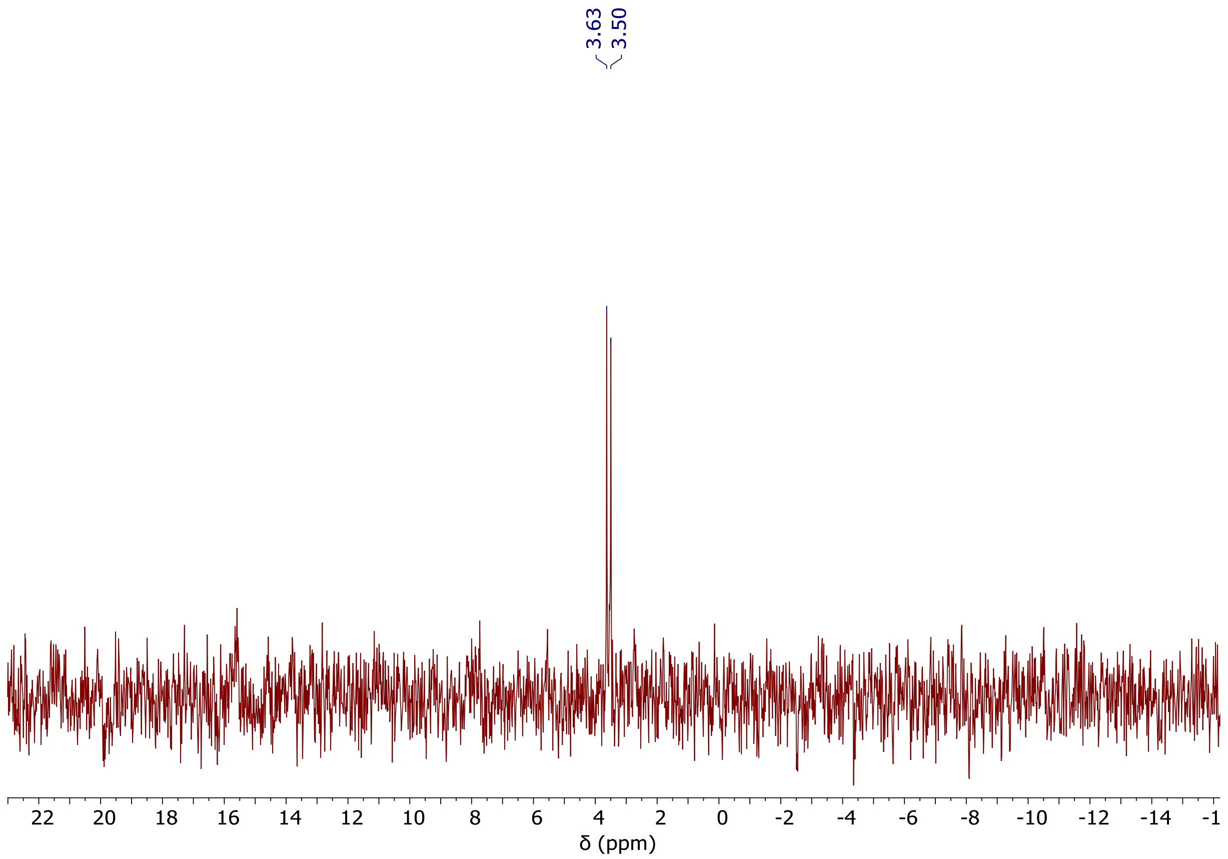

Figure S37. $^{29}Si\{^{1}H\}$ NMR spectrum of **3d** as a solution in C_6D_6 at ambient temperature.

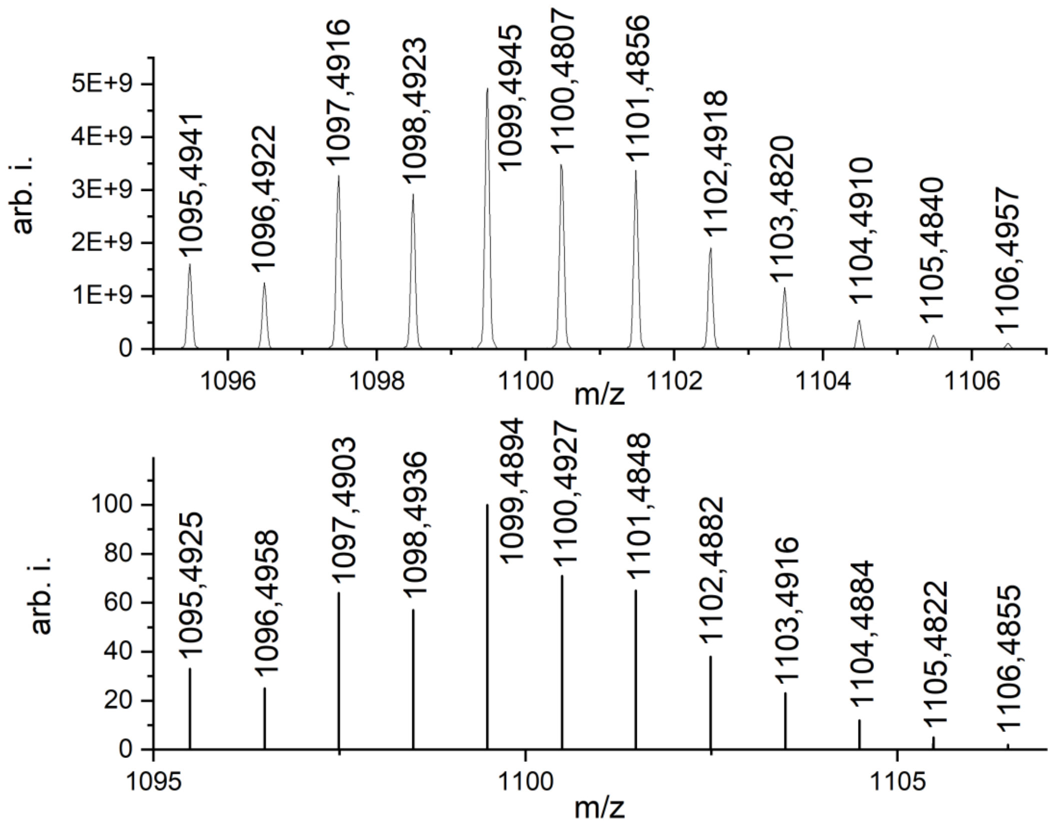

Figure S38. *Top*: Cutout from LIFDI/MS of **3d**; *Bottom*: Calculated MS spectrum of [**3d**]$^+$.

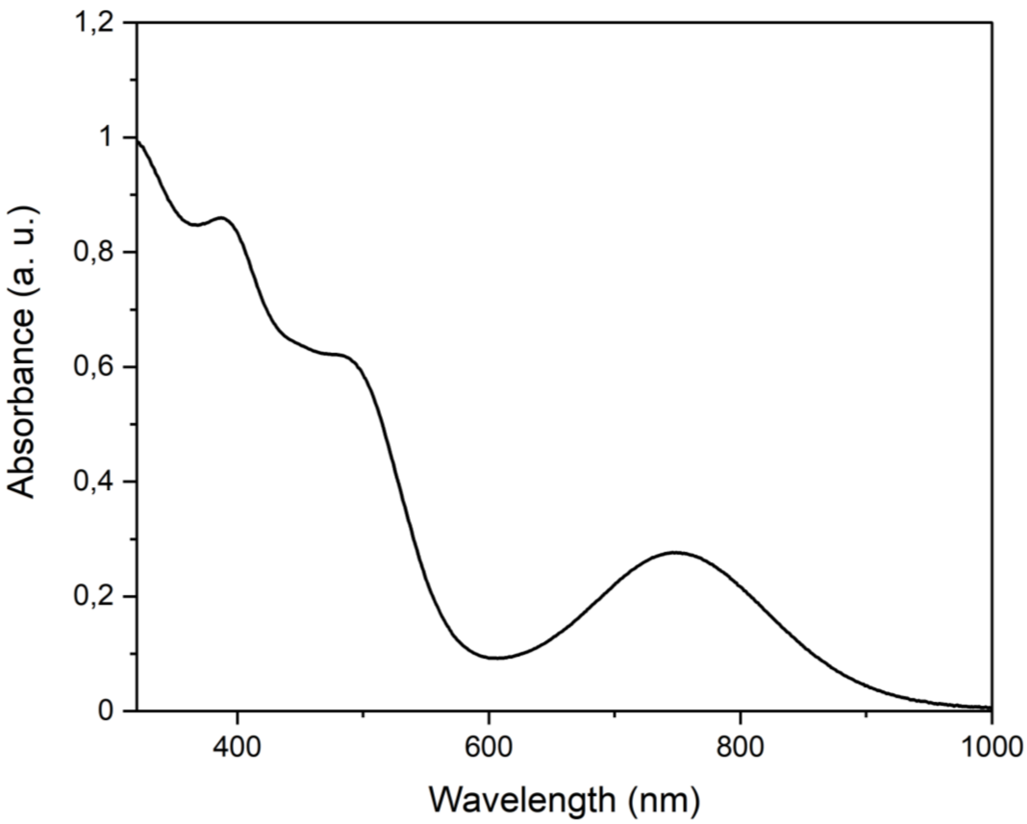

Figure S39. UV/vis spectrum of a 1.0x10^{-4} M solution of **3d** in toluene at ambient temperature.

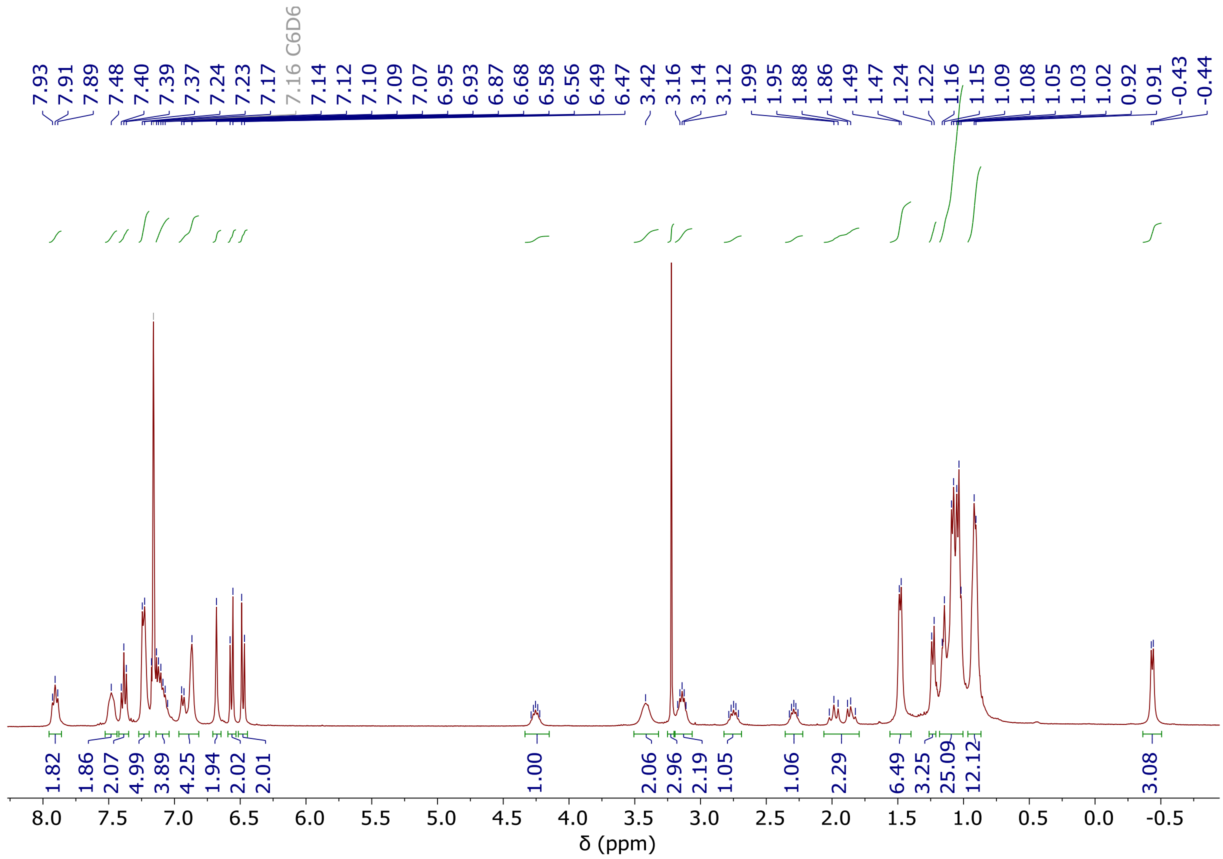

Figure S40. ^{1}H NMR spectrum of **3e** as a solution in C_6D_6 at ambient temperature.

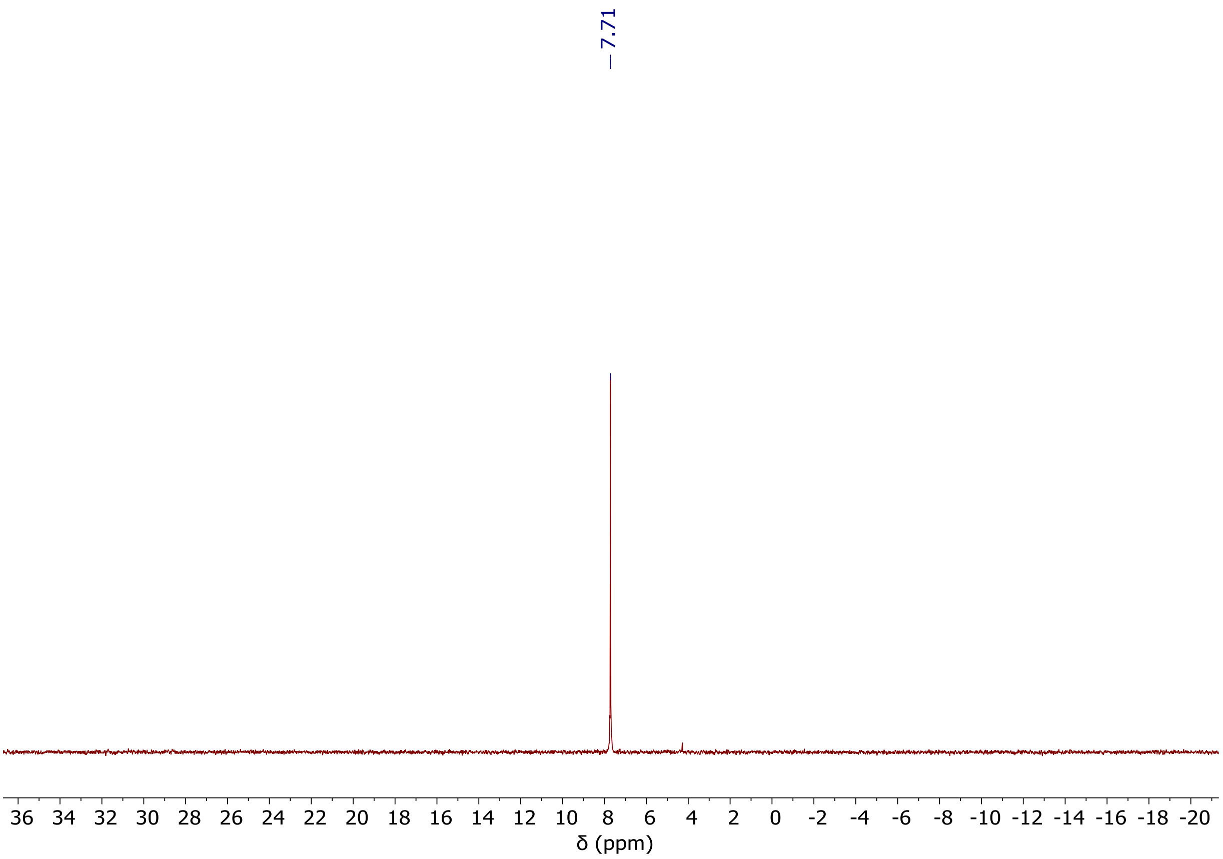

Figure S41. $^{31}P\{^1H\}$ NMR spectrum of **3e** as a solution in C_6D_6 at ambient temperature.

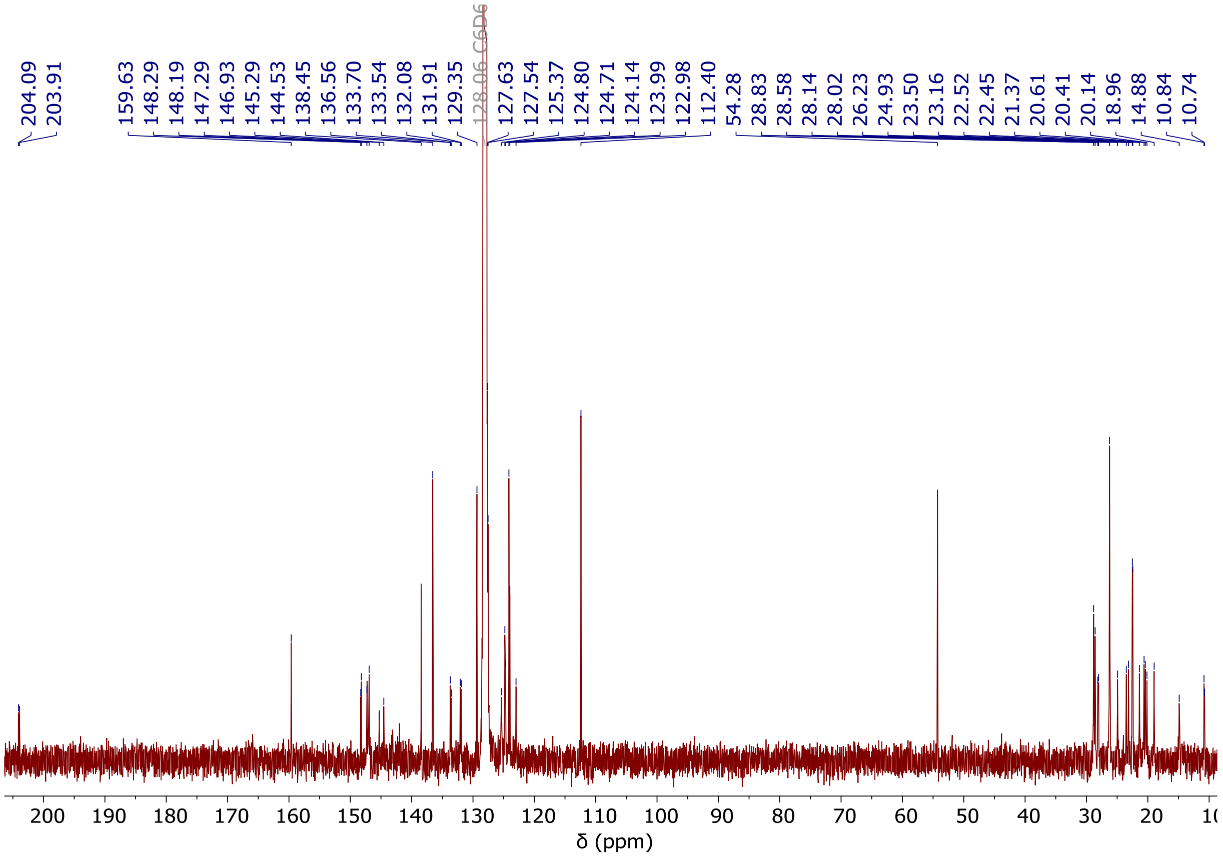

Figure S42. $^{13}C\{^1H\}$ NMR spectrum of **3e** as a solution in C_6D_6 at ambient temperature.

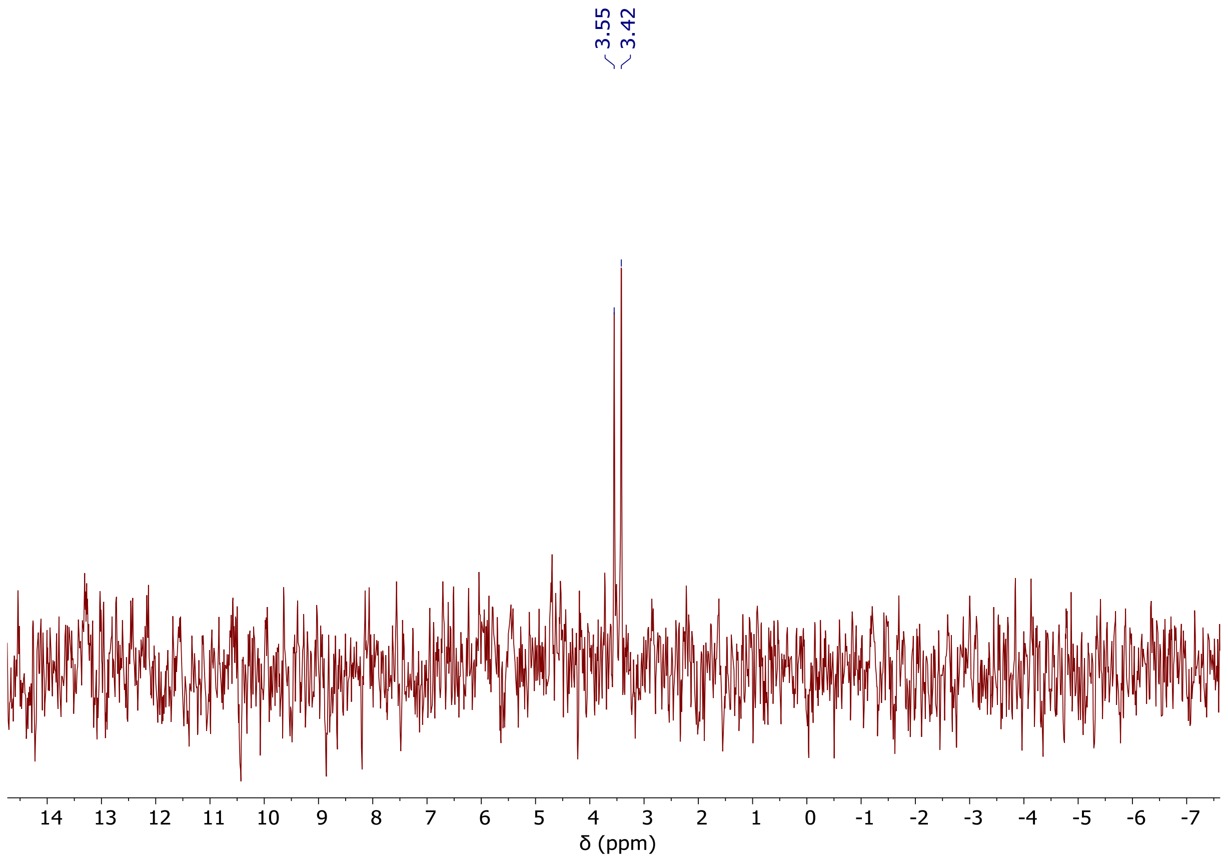

Figure S43. $^{29}Si\{^1H\}$ NMR spectrum of **3e** as a solution in C_6D_6 at ambient temperature.

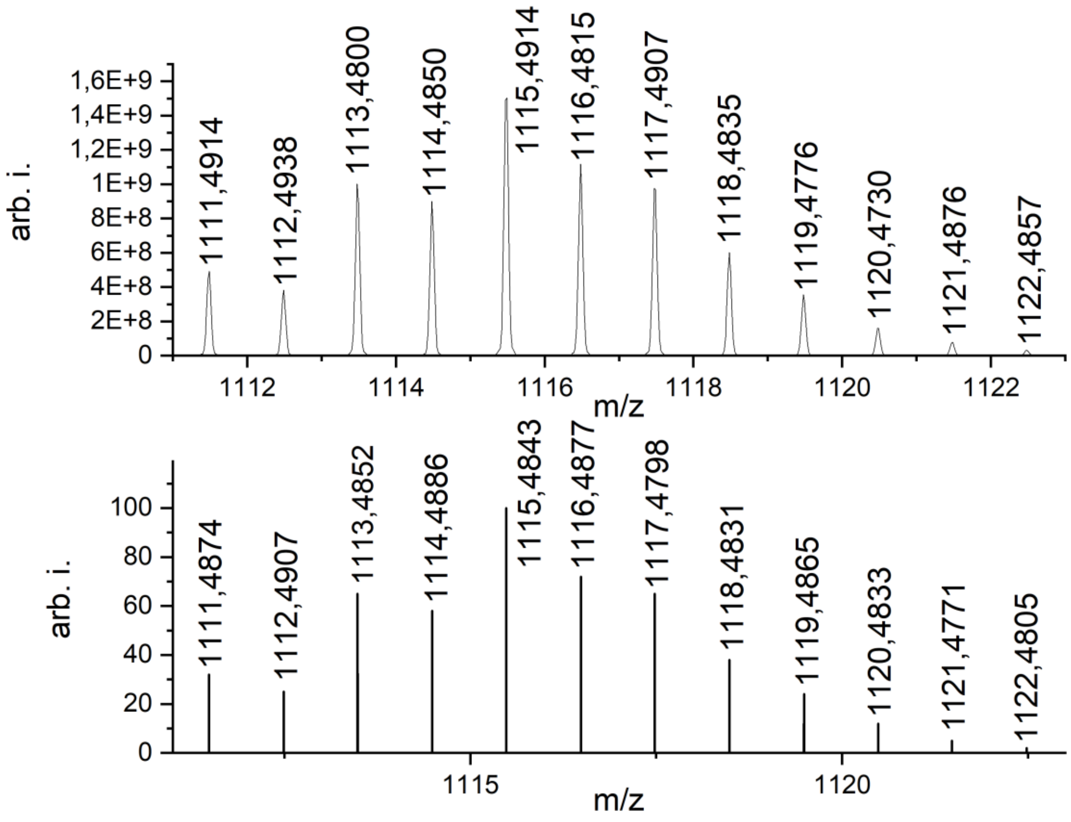

Figure S44. *Top*: Cutout from LIFDI/MS of **3e**; *Bottom*: Calculated MS spectrum of [**3e**]$^+$.

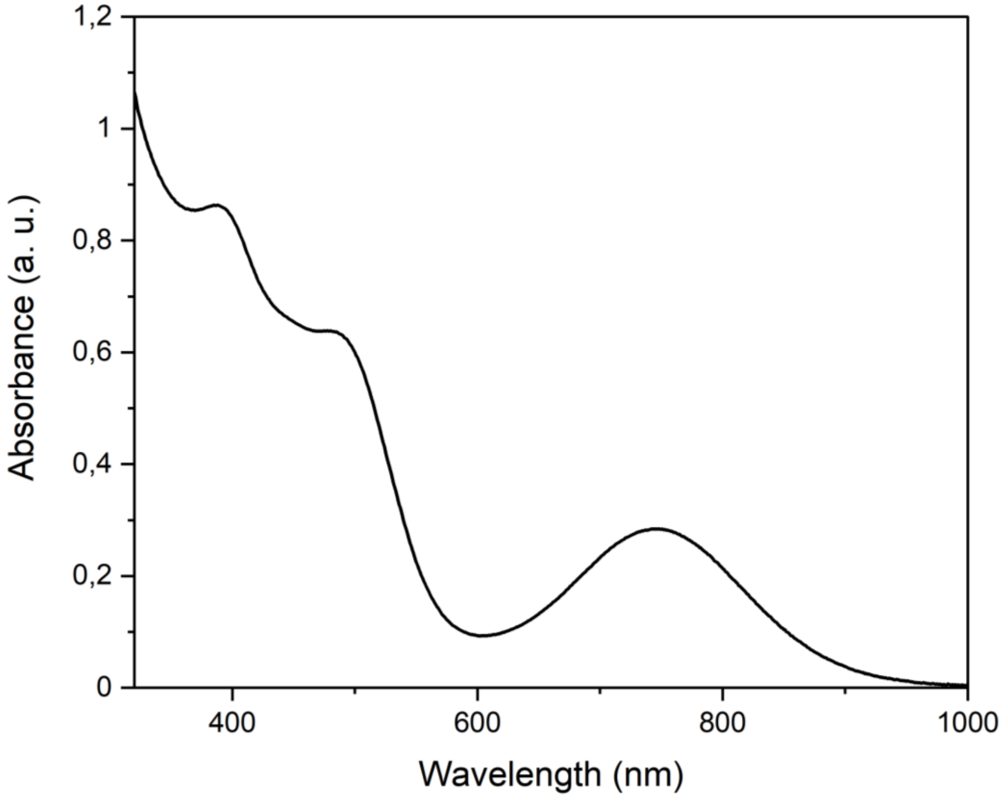

Figure S45. UV/vis spectrum of a 1.0x10^{-4} M solution of **3e** in toluene at ambient temperature.

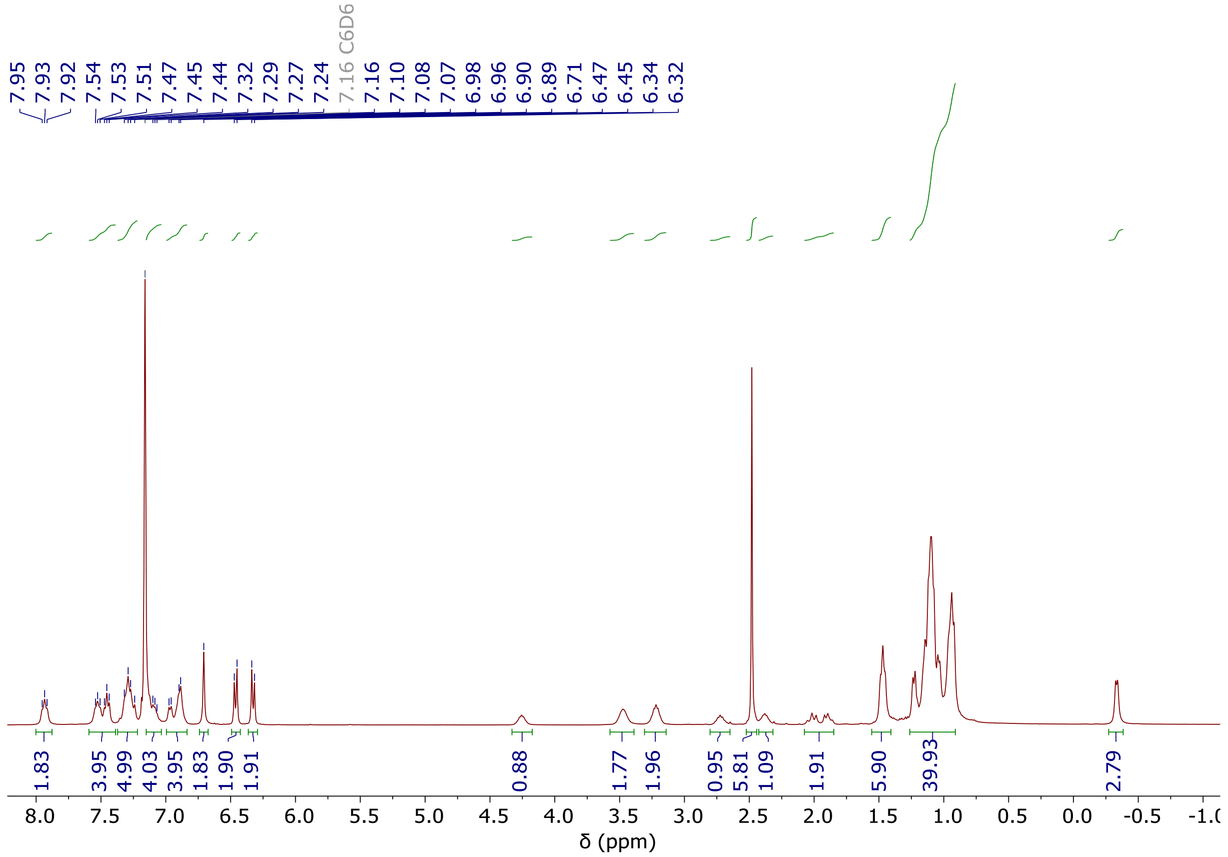

Figure S46. ^{1}H NMR spectrum of **3f** as a solution in C_6D_6 at ambient temperature.

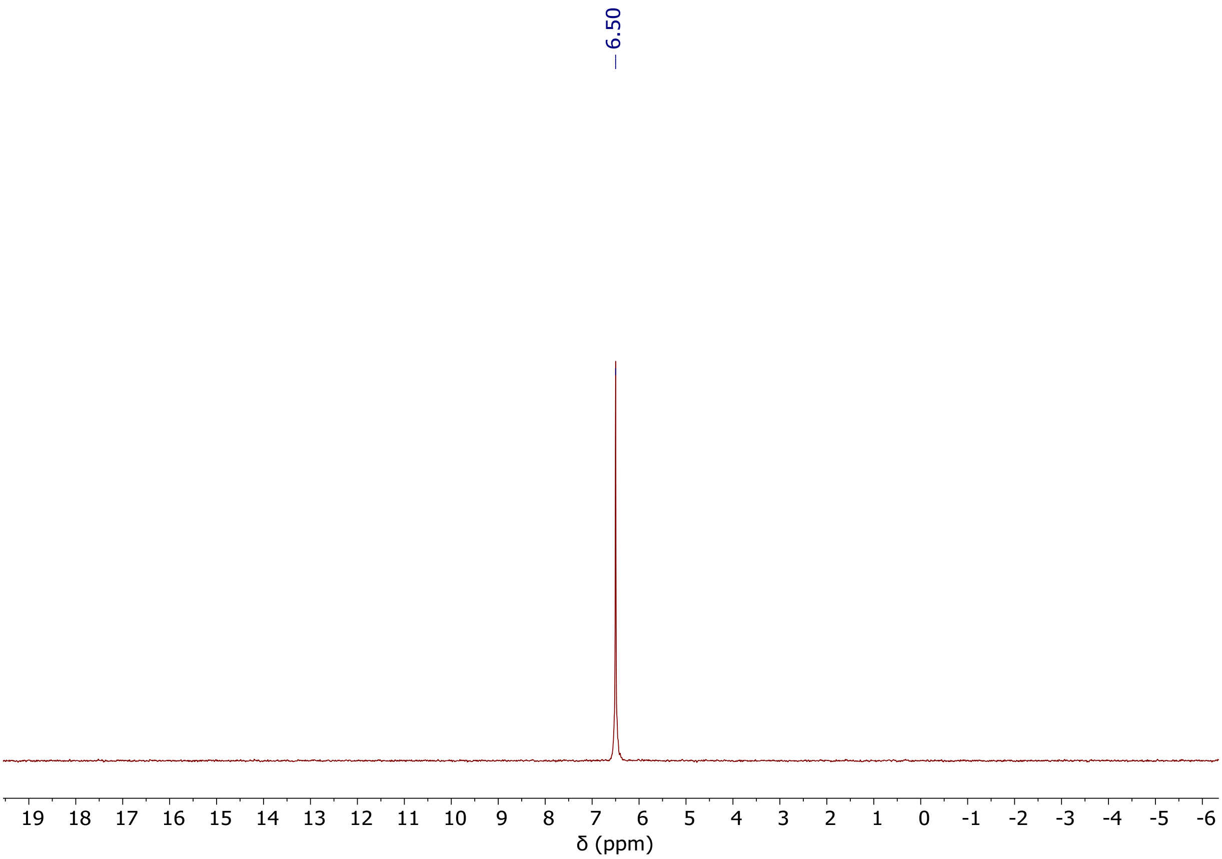

Figure S47. $^{31}P\{^{1}H\}$ NMR spectrum of **3f** as a solution in C_6D_6 at ambient temperature.

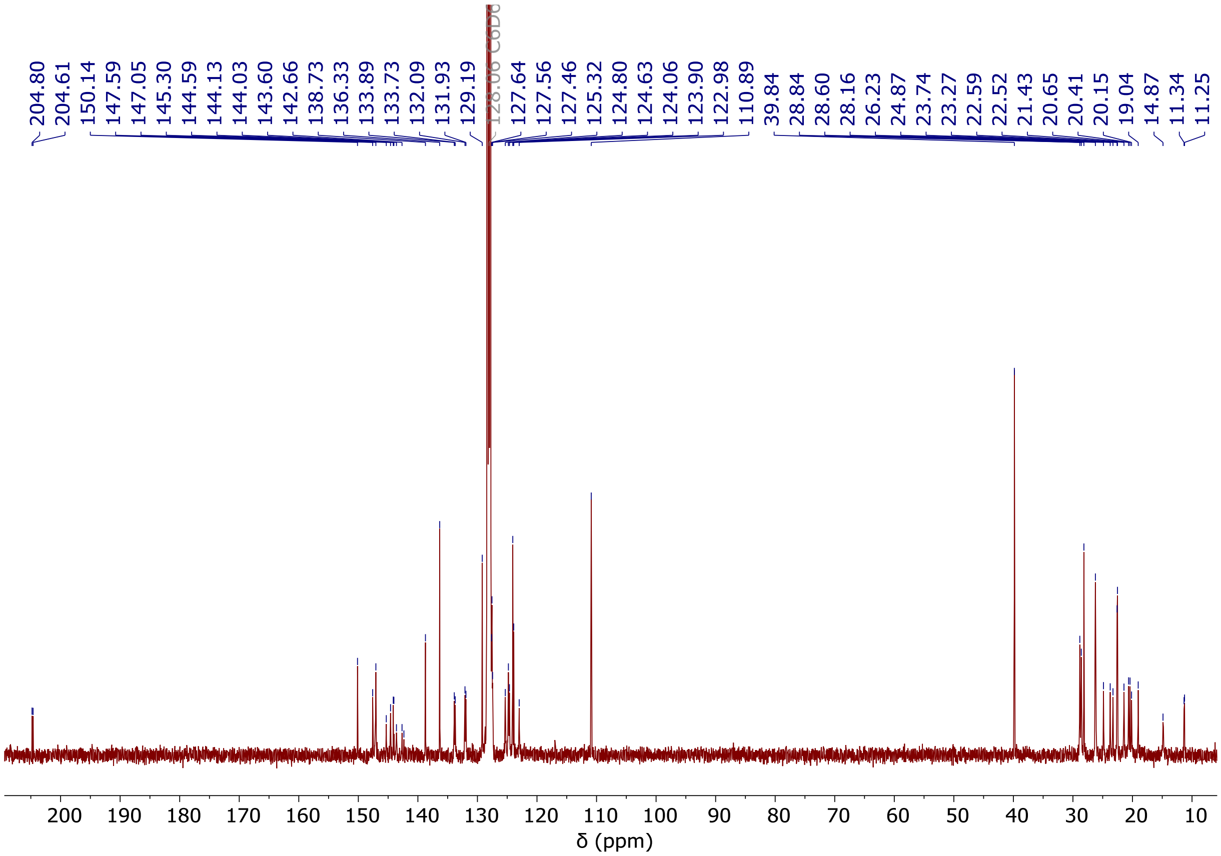

Figure S48. $^{13}C\{^{1}H\}$ NMR spectrum of **3f** as a solution in C_6D_6 at ambient temperature.

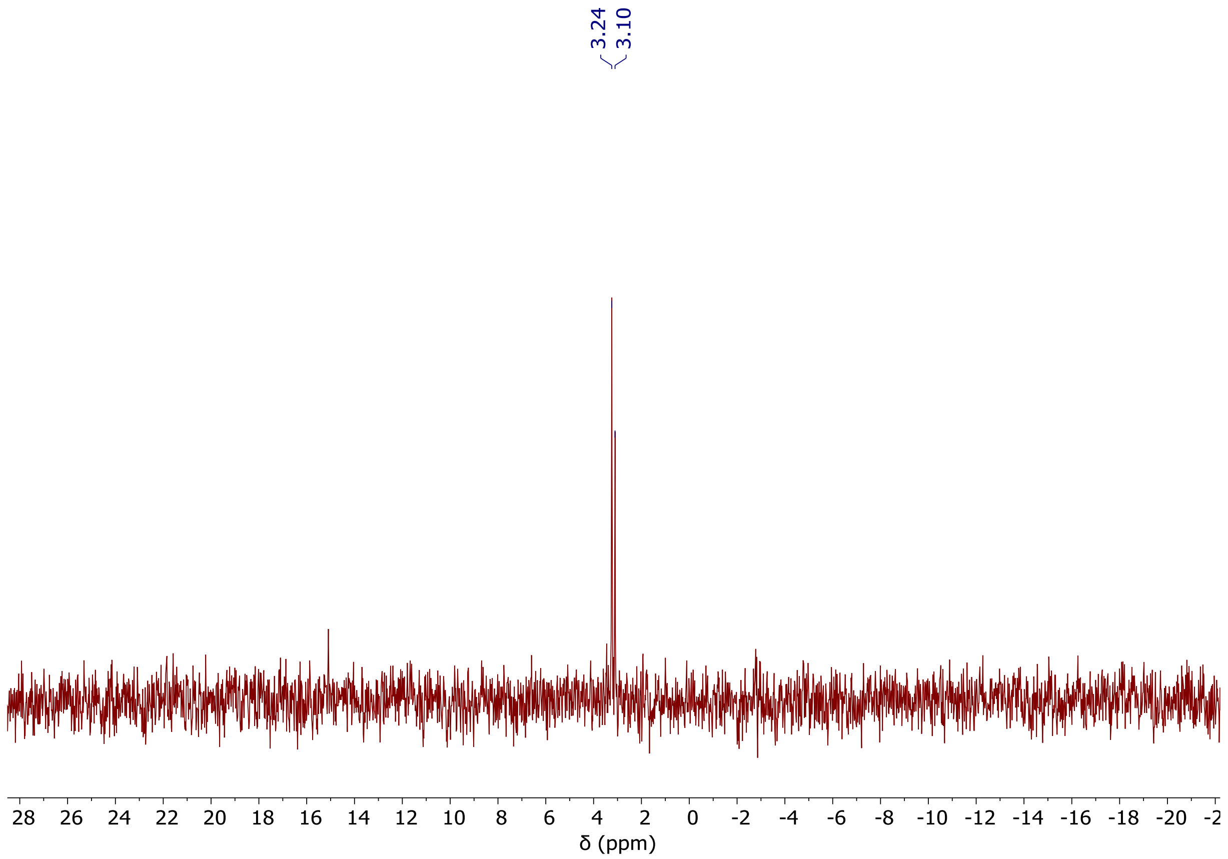

Figure S49. $^{29}Si\{^{1}H\}$ NMR spectrum of **3f** as a solution in C_6D_6 at ambient temperature.

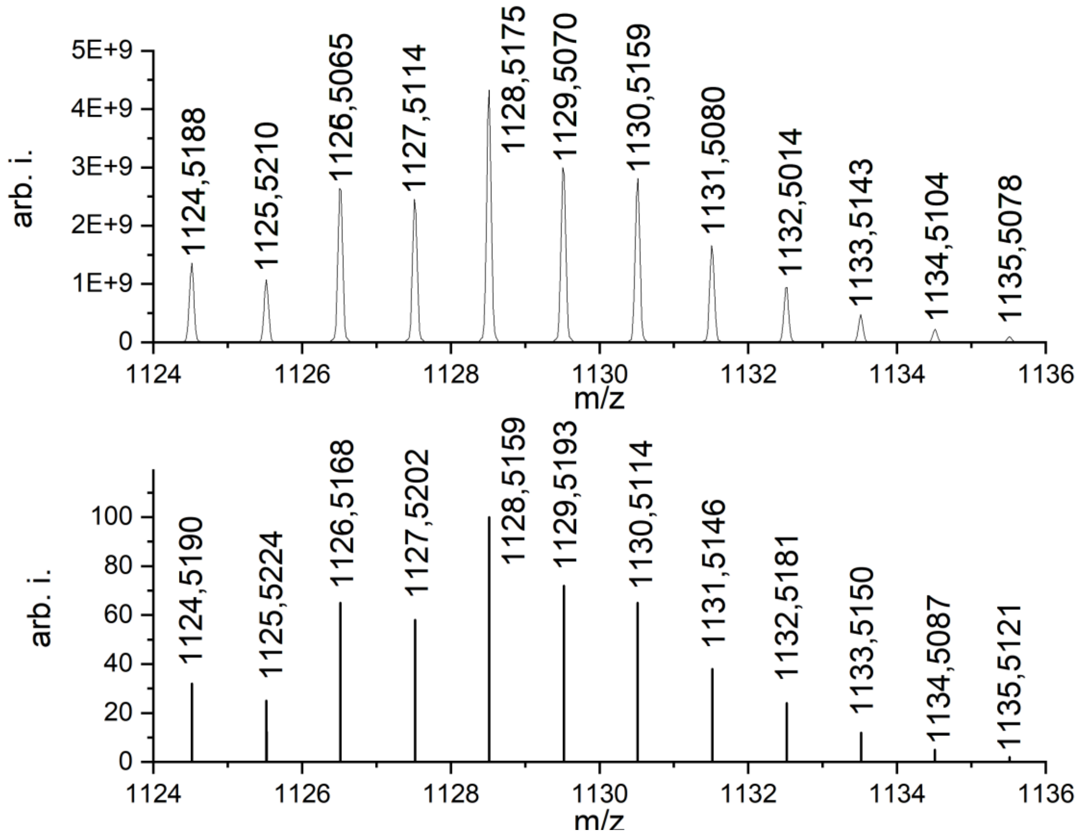

Figure S50. *Top*: Cutout from LIFDI/MS of **3f**; *Bottom*: Calculated MS spectrum of [**3f**]$^{+}$.

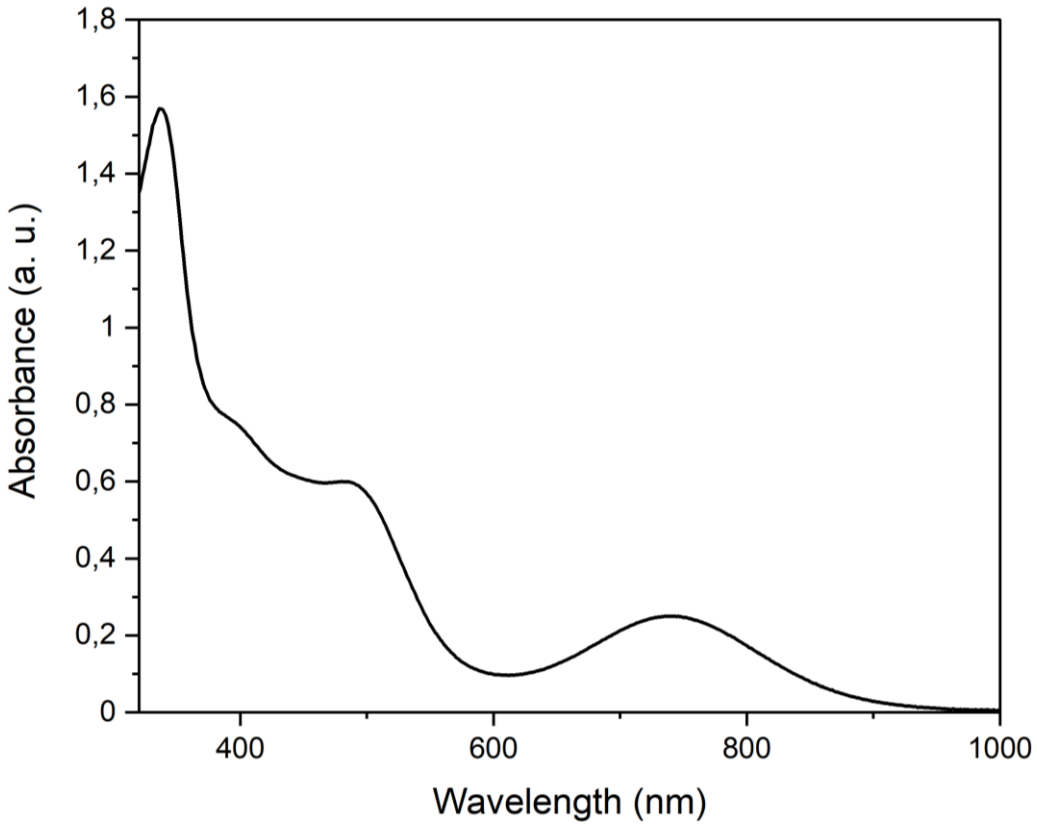

Figure S51. UV/vis spectrum of a 1.0x10^{-4} M solution of **3f** in toluene at ambient temperature.

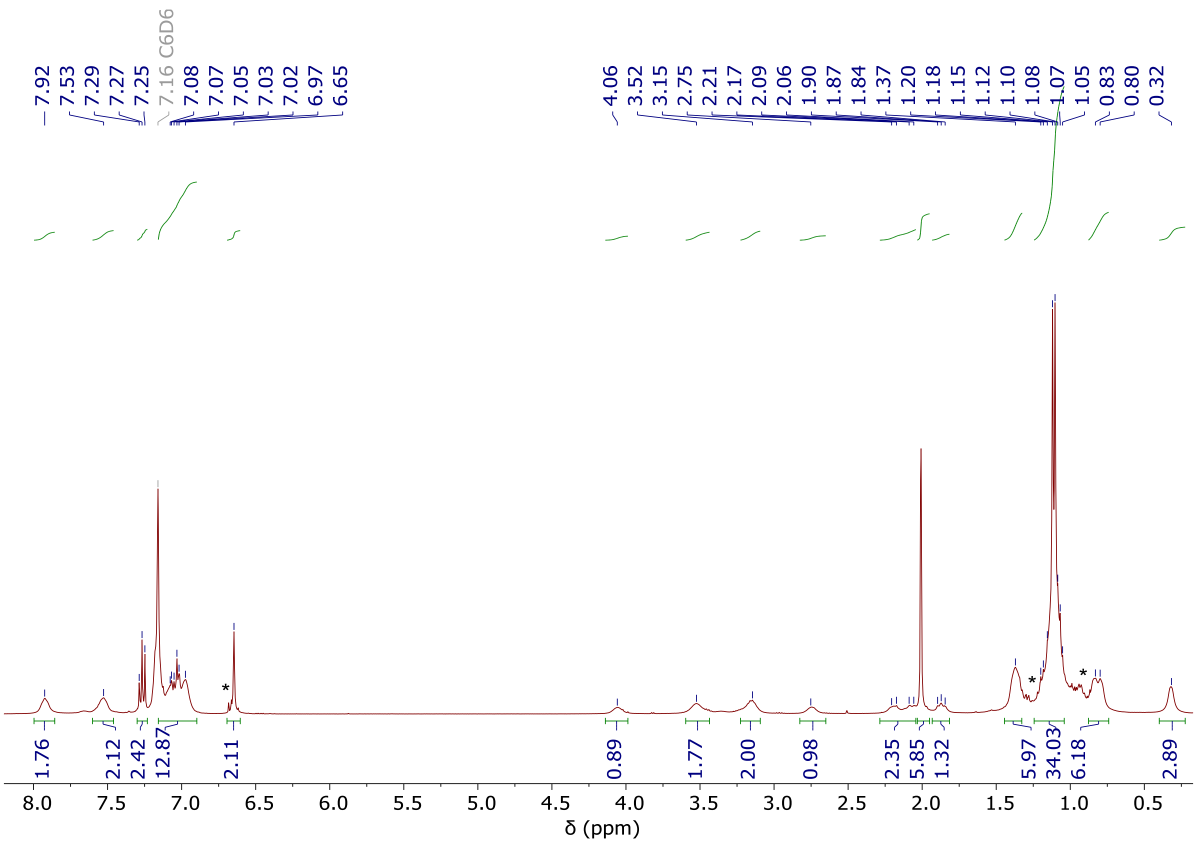

Figure S52. ^{1}H NMR spectrum of **3g** as a solution in C_6D_6 at ambient temperature;* indicates minor amount of unknown impurity.

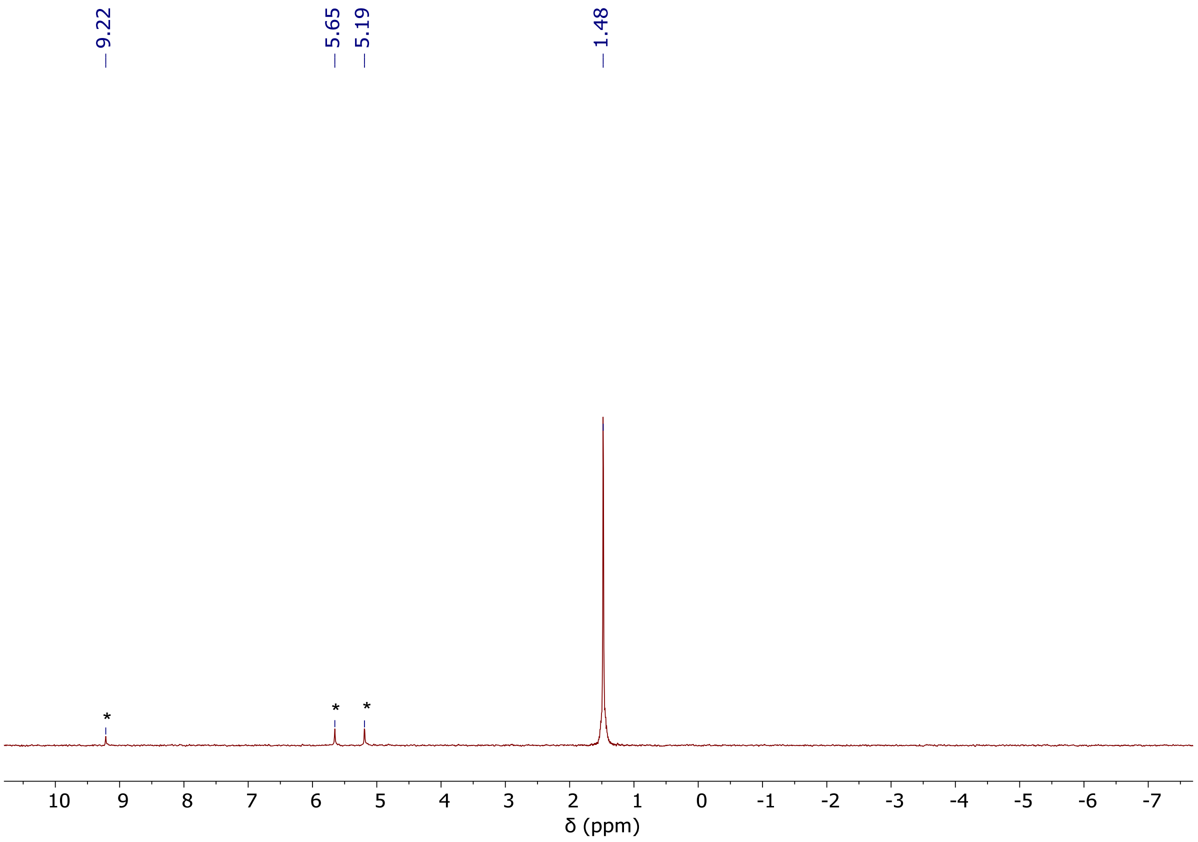

Figure S53. $^{31}P\{^1H\}$ NMR spectrum of **3g** as a solution in C_6D_6 at ambient temperature;* indicates minor amount of unknown impurity.

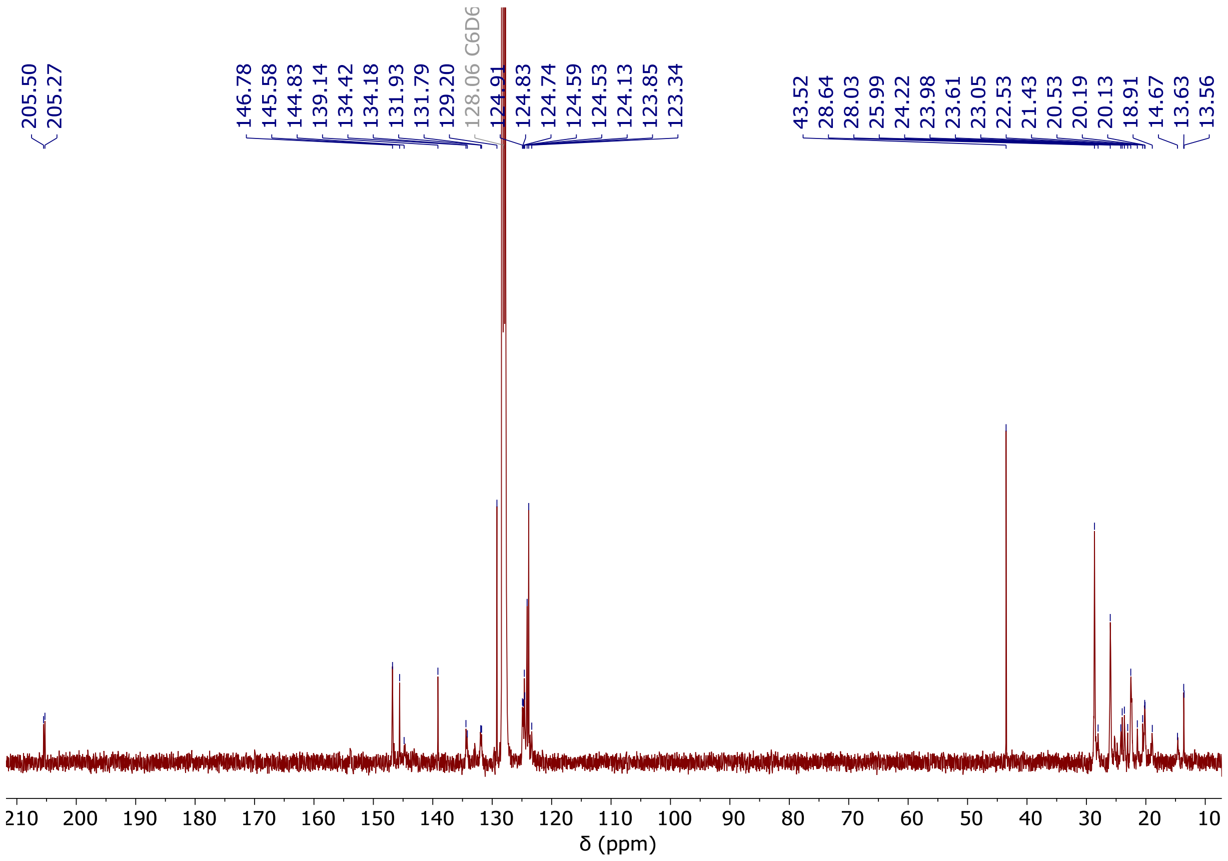

Figure S54. $^{13}C\{^1H\}$ NMR spectrum of **3g** as a solution in C_6D_6 at ambient temperature.

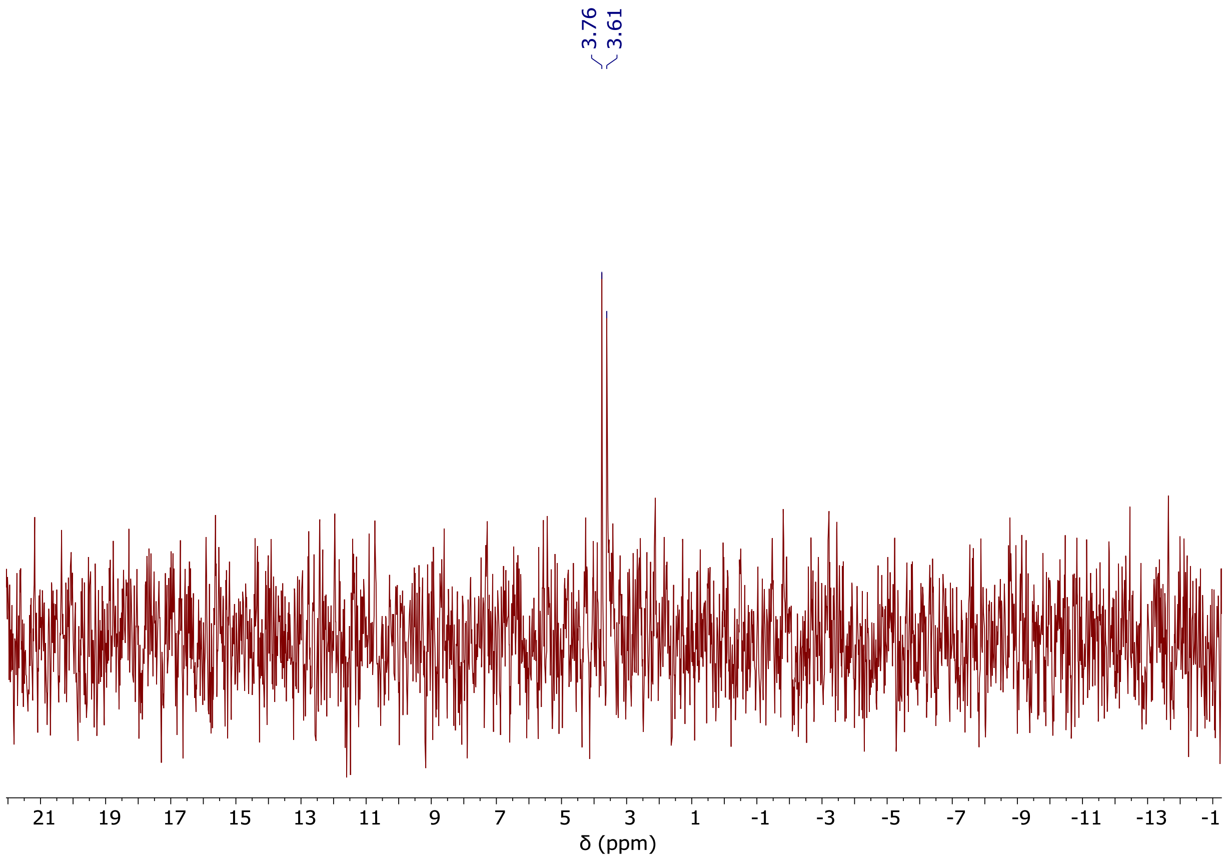

Figure S55. $^{29}Si\{^{1}H\}$ NMR spectrum of **3g** as a solution in C_6D_6 at ambient temperature.

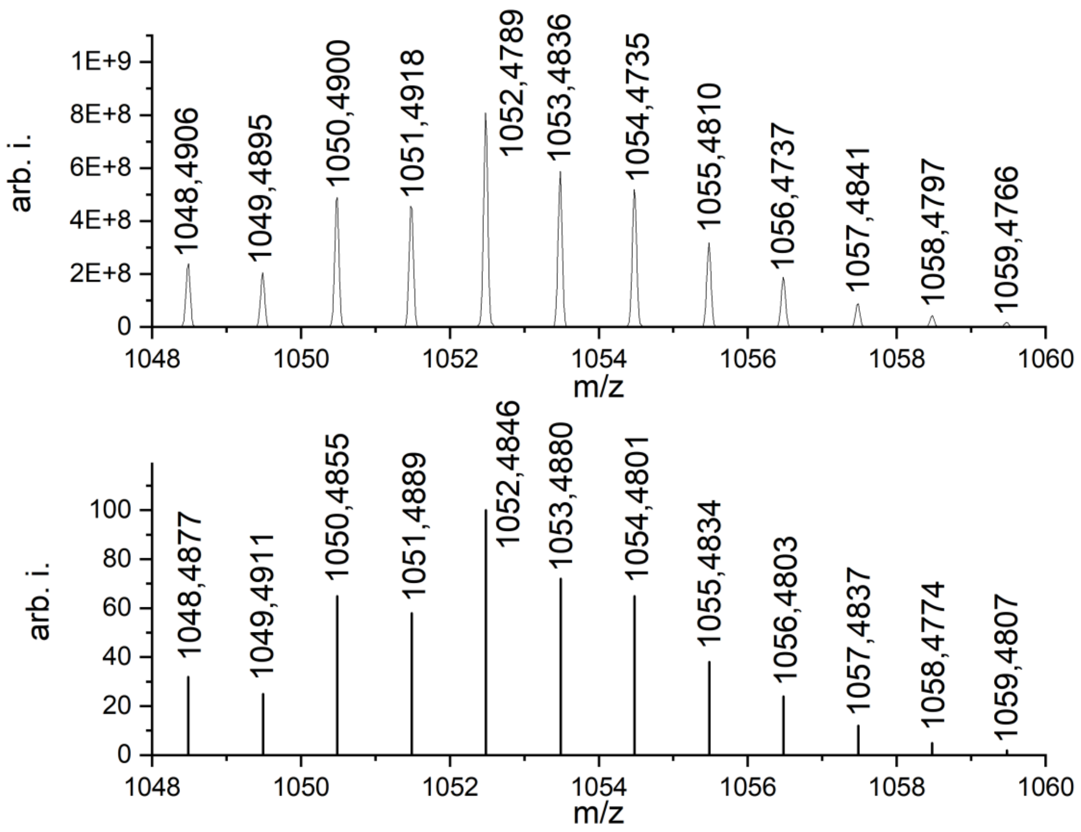

Figure S56. *Top*: Cutout from LIFDI/MS of **3g**; *Bottom*: Calculated MS spectrum of [**3g**]$^{+}$.

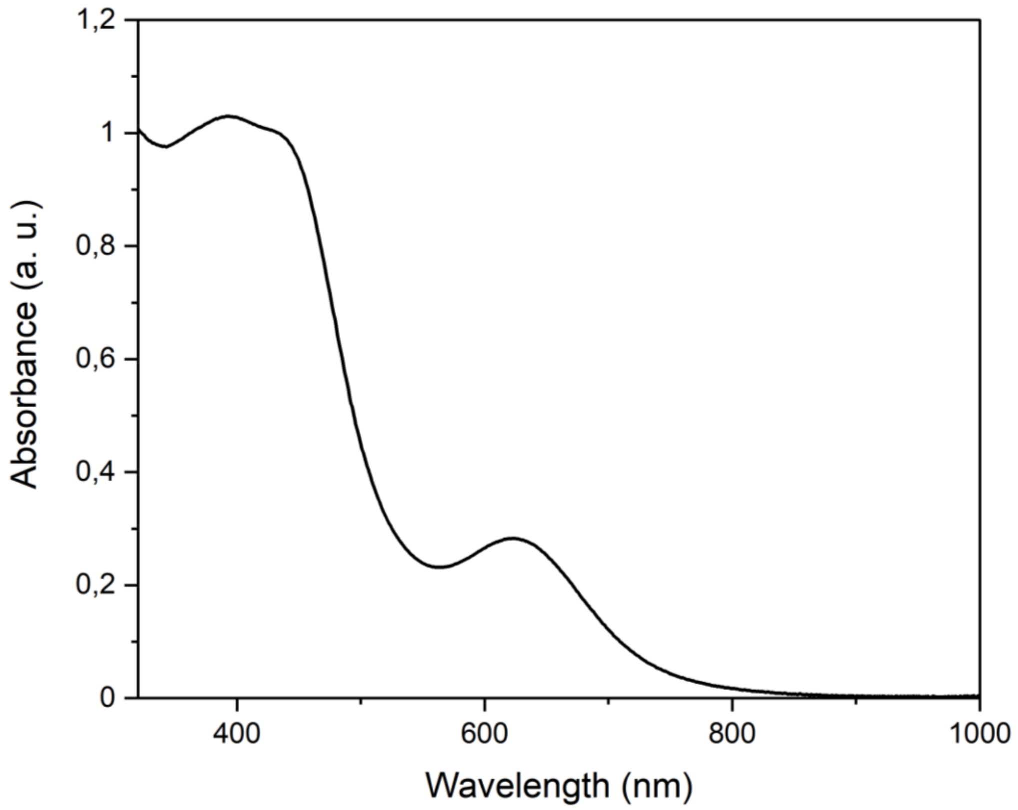

Figure S57. UV/vis spectrum of a 1.0x10^{-4} M solution of **3g** in toluene at ambient temperature.

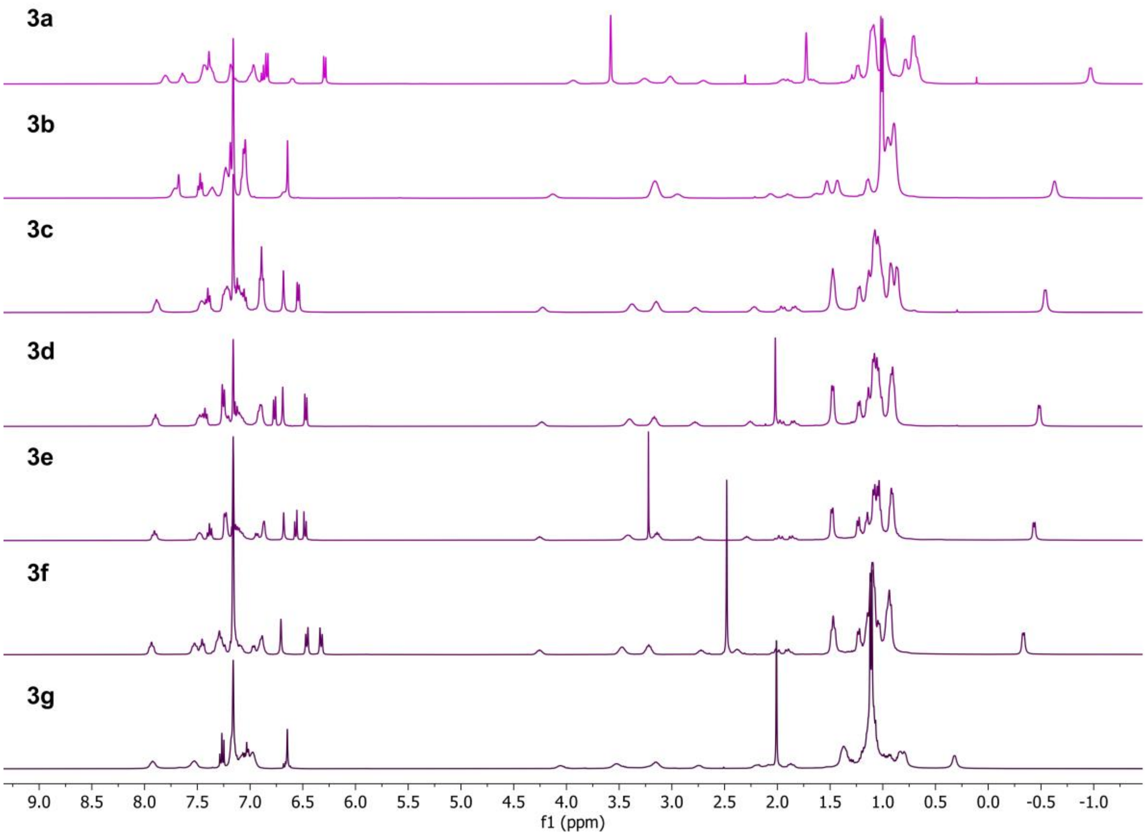

Figure S58. Stack-plot of ^{1}H NMR spectra of **3a**-**3g**.

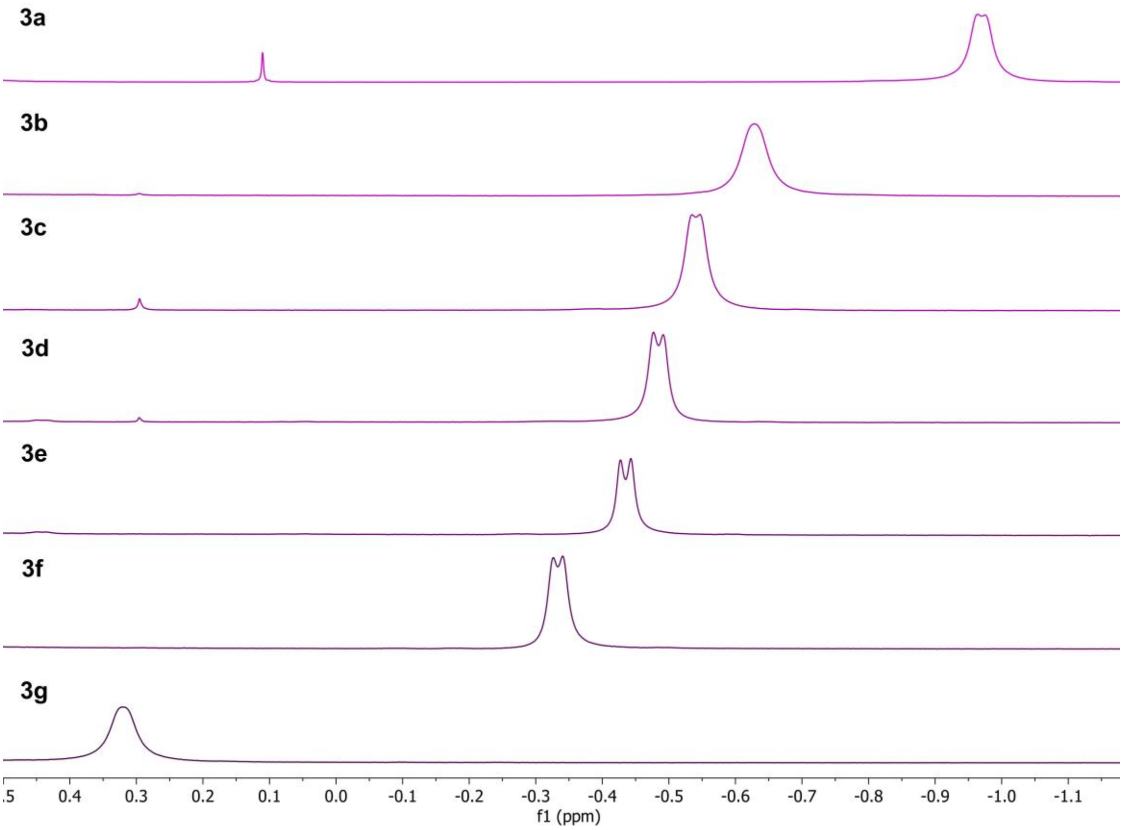

Figure S59. Stack-plot of ^{1}H NMR spectra of **3a**-**3g**, between 0.5 and -1.2 ppm, showing the upfield shift of one ^{i}Pr-CH_3 doublet

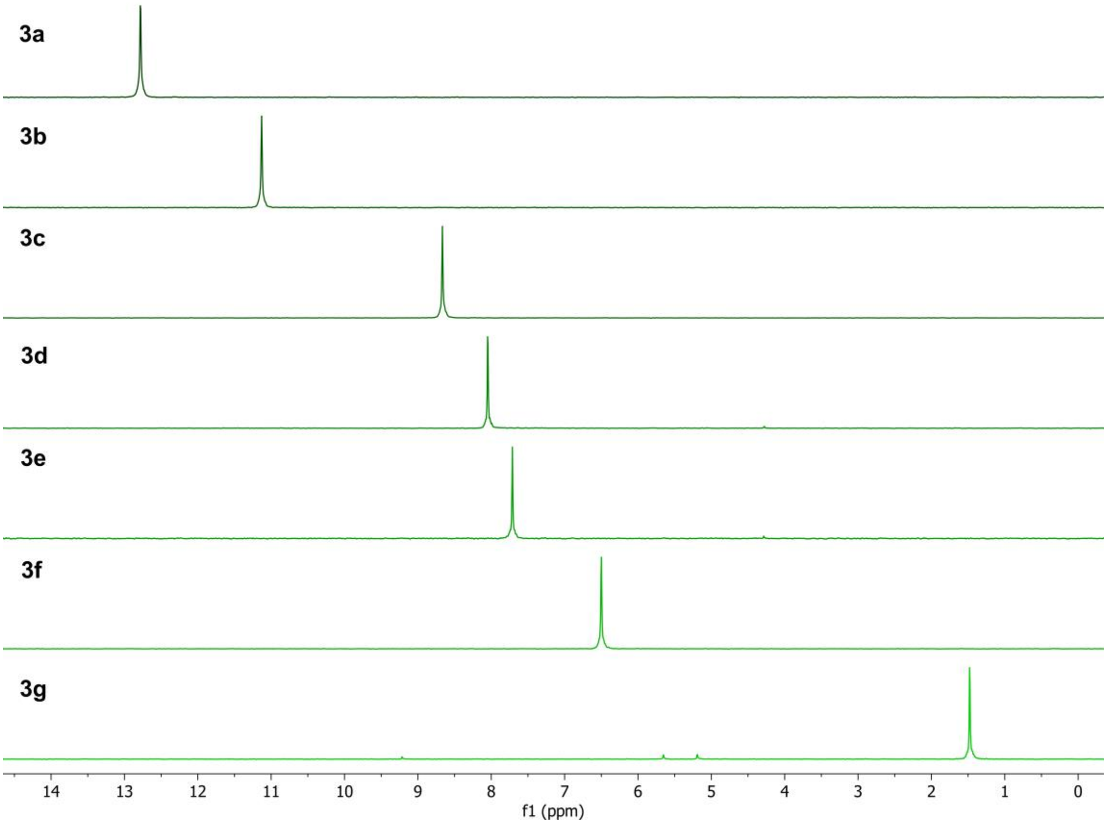

Figure S60. Stack-plot of $\{^{1}H\}^{31}P$ NMR spectra of **3a**-**3g**, showing the gradual shift of the P-centres in these complexes.

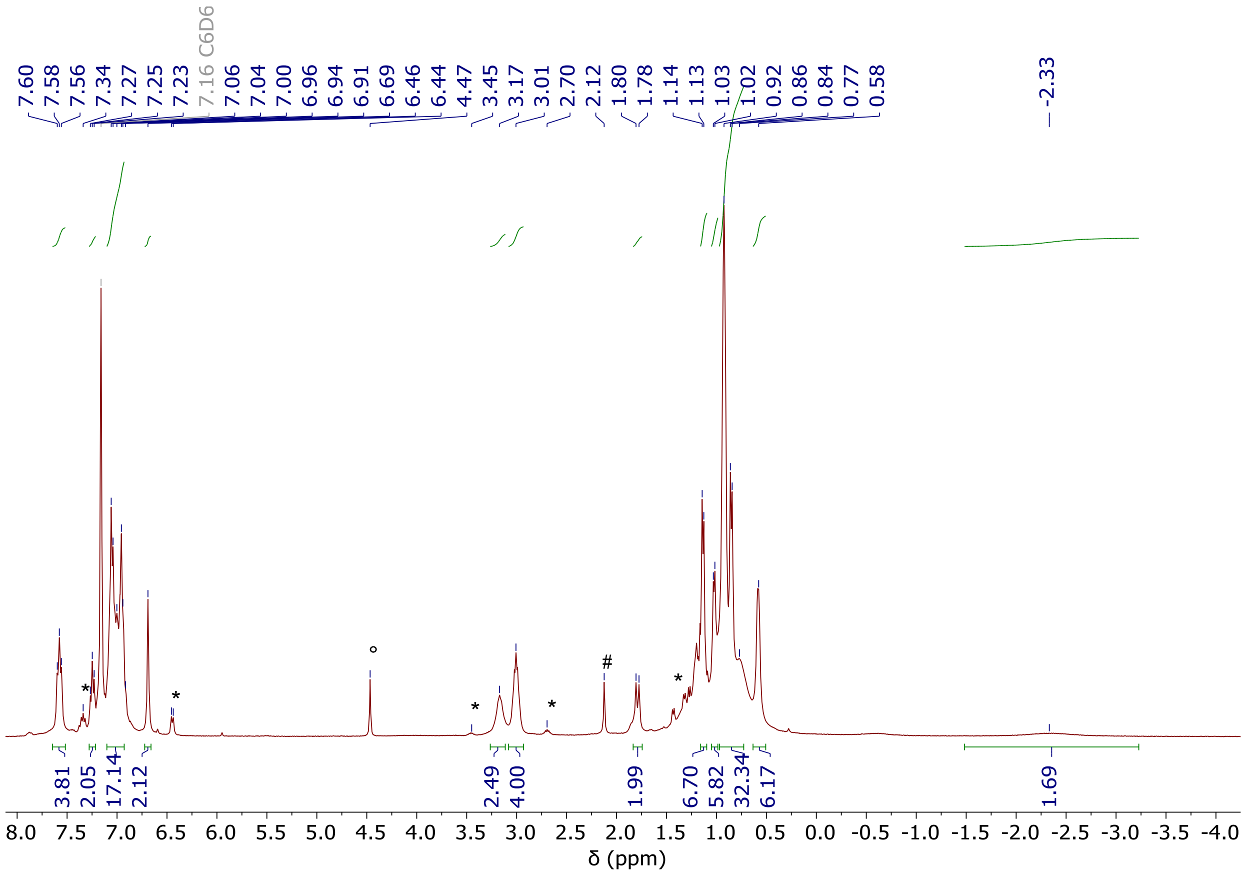

Figure S61. ^{1}H NMR spectrum of **3a** as a solution in C_6D_6 at 60°C under 1 atm H_2 exhibiting an equilibrium with **4a**; *indicates **3a**; °indicates H_2, #indicates minor amounts of toluene.

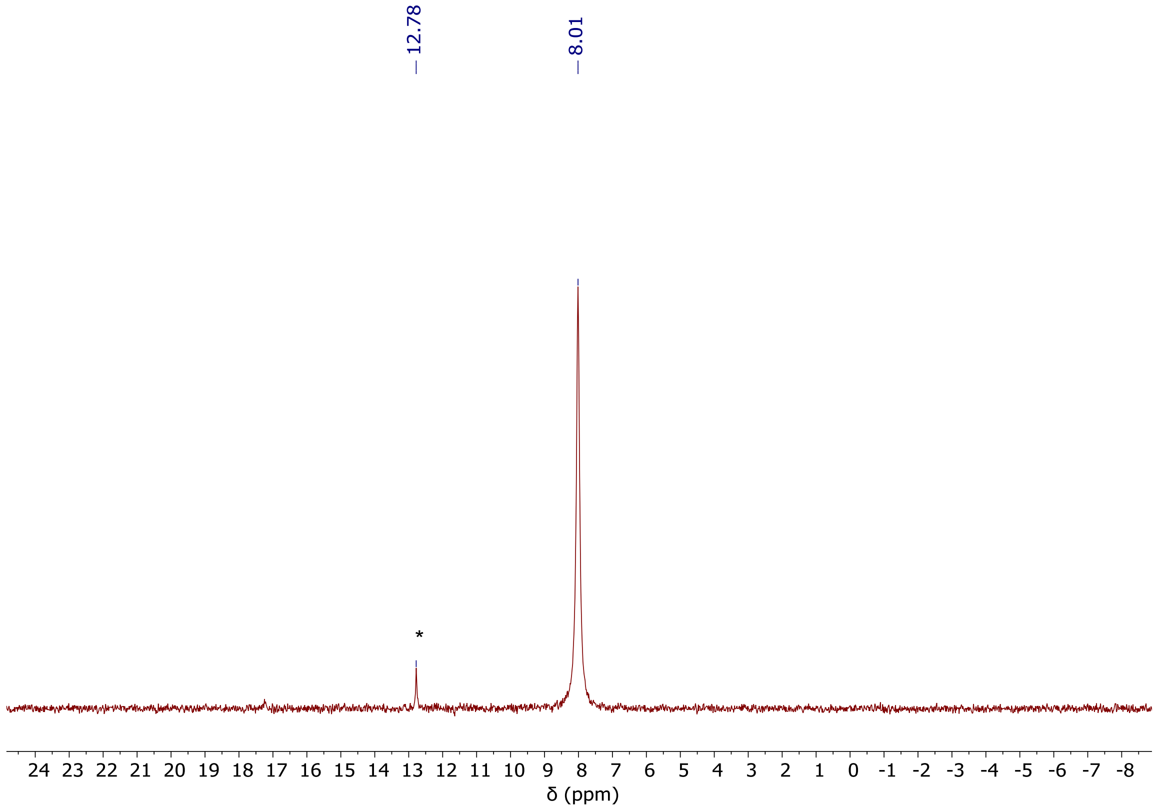

Figure S62. ^{31}P{^{1}H} NMR spectrum of **3a** as a solution in C_6D_6 at ambient temperature under 1 atm H_2 exhibiting an equilibrium with **4a**; *indicates **3a**.

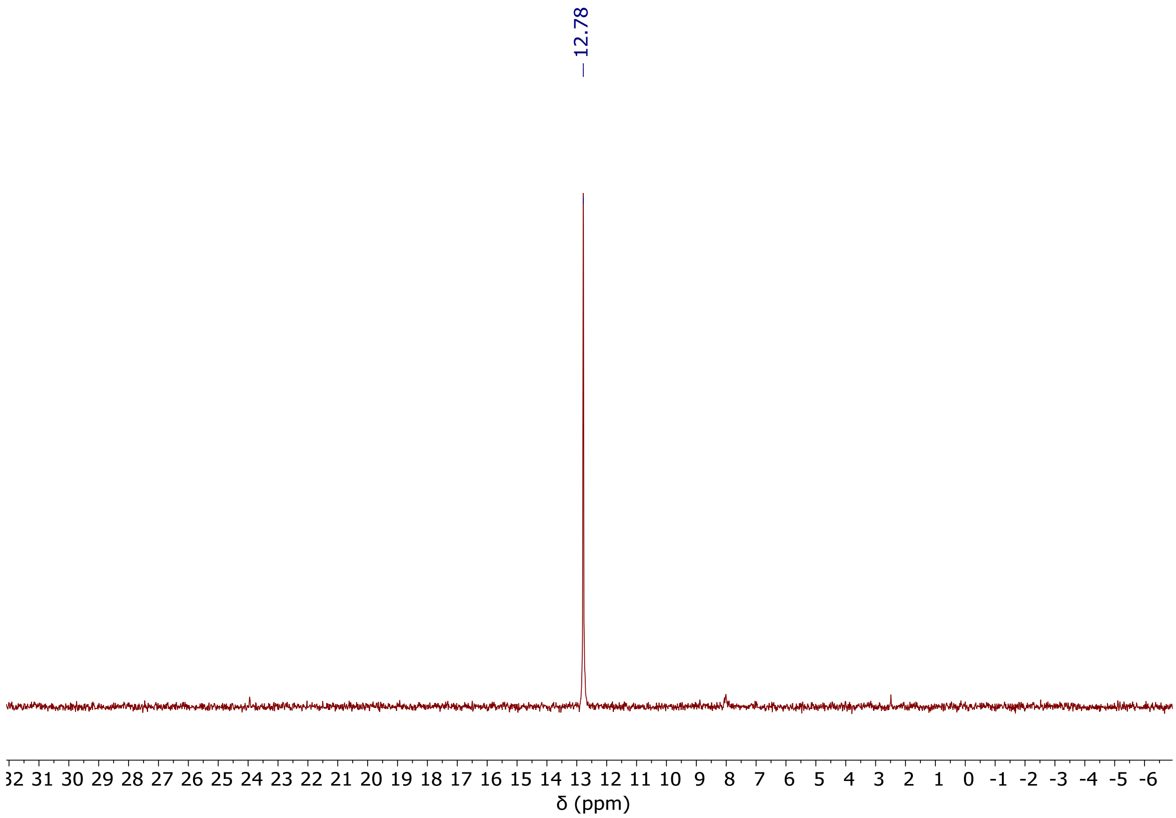

Figure S63. $^{31}P\{^1H\}$ NMR spectrum after removing all volatiles of the reaction of **3a** with H_2 *in vacuo* and redissolving the residue in C_6D_6 showing full regeneration of **3a**.

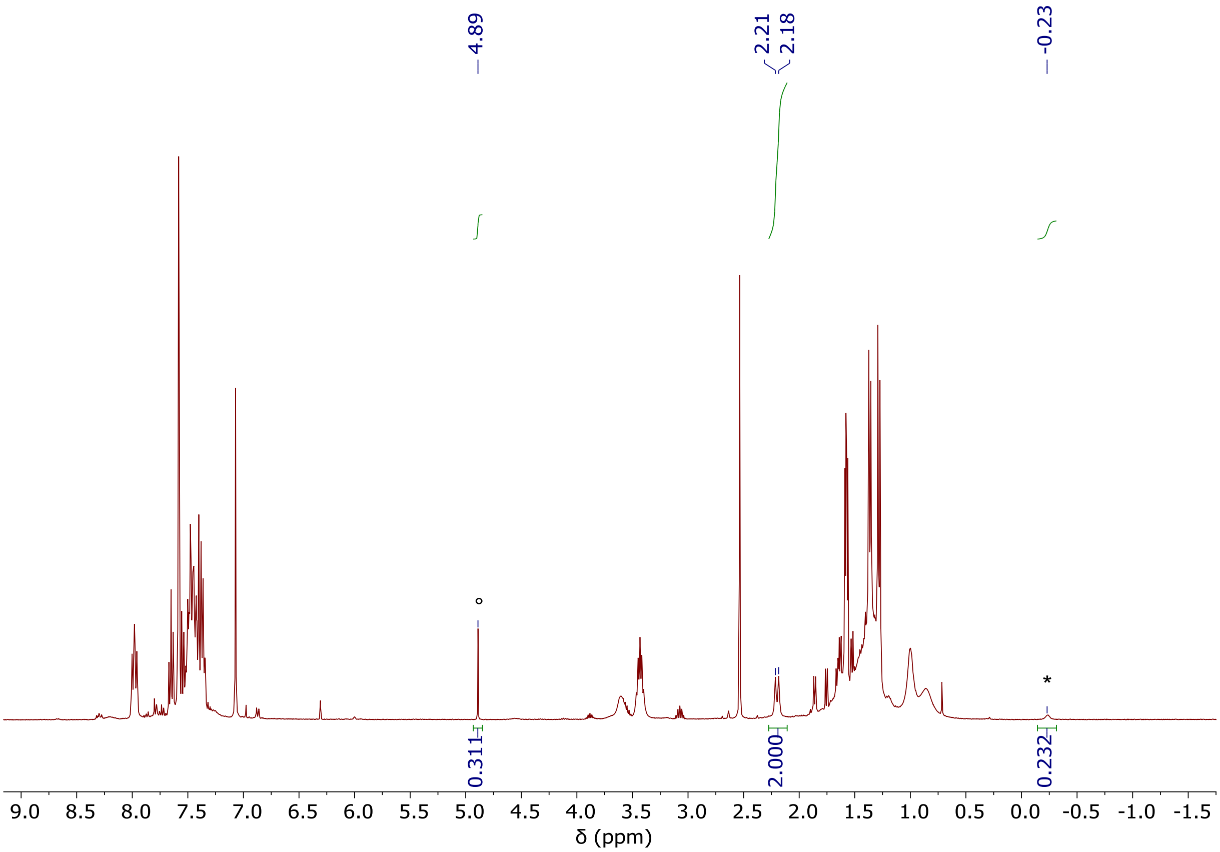

Figure S64. ^1H NMR spectrum of 9 mg of **3a** under 1 atm H_2 as a solution in 0.45 mL C_6D_6 at 26°C exhibiting an equilibrium with **4a**; *indicates **3a**; °indicates H_2.

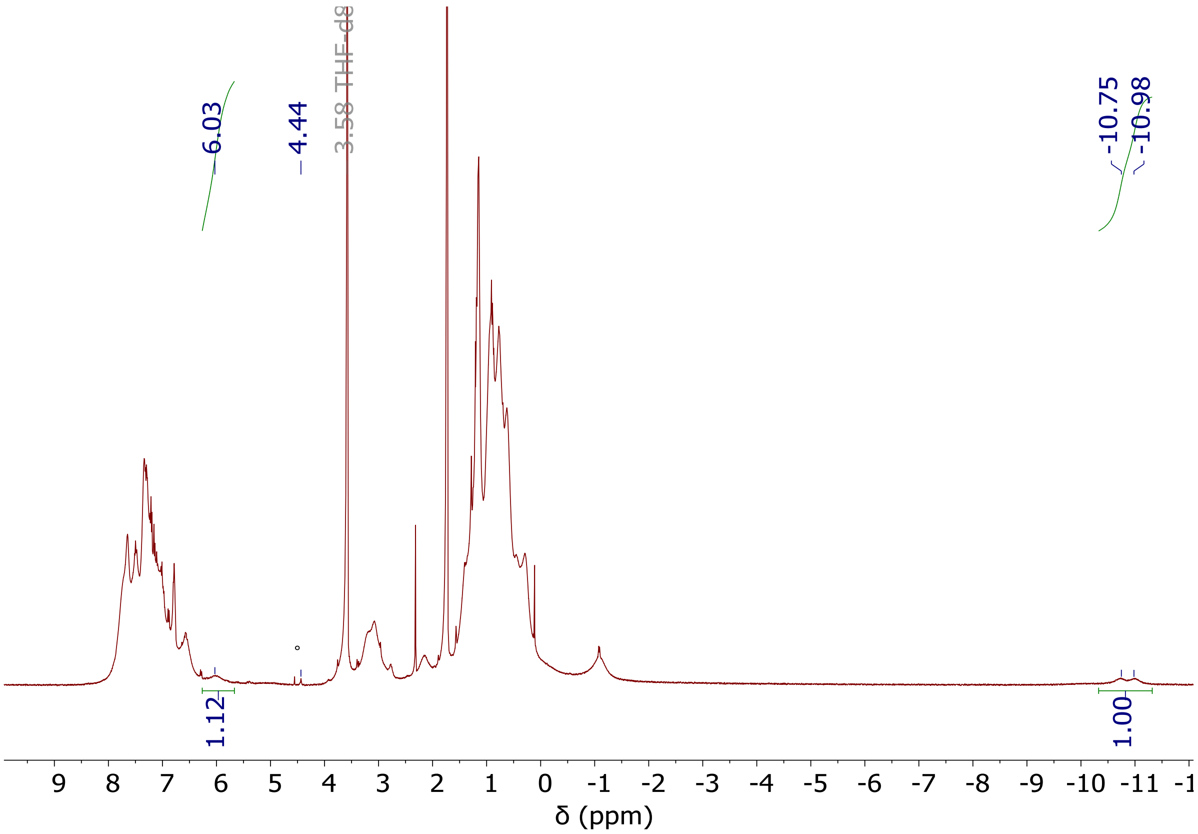

Figure S65. ^{1}H NMR spectrum of **3a** as a solution in THF-d_8 at -40°C under 1 atm H_2 showing the Ge-*H* and Ni-*H* hydride peaks. °indicates H_2.

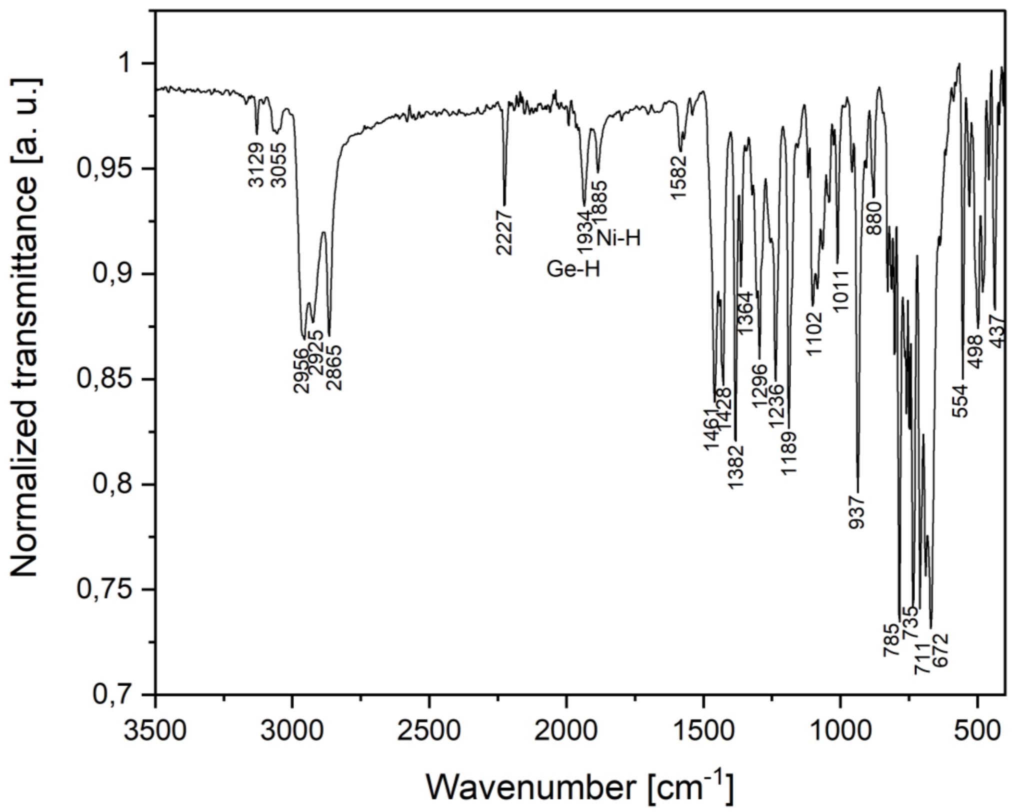

Figure S66. ATR-IR spectrum of solid crystalline **4a** at ambient temperature.

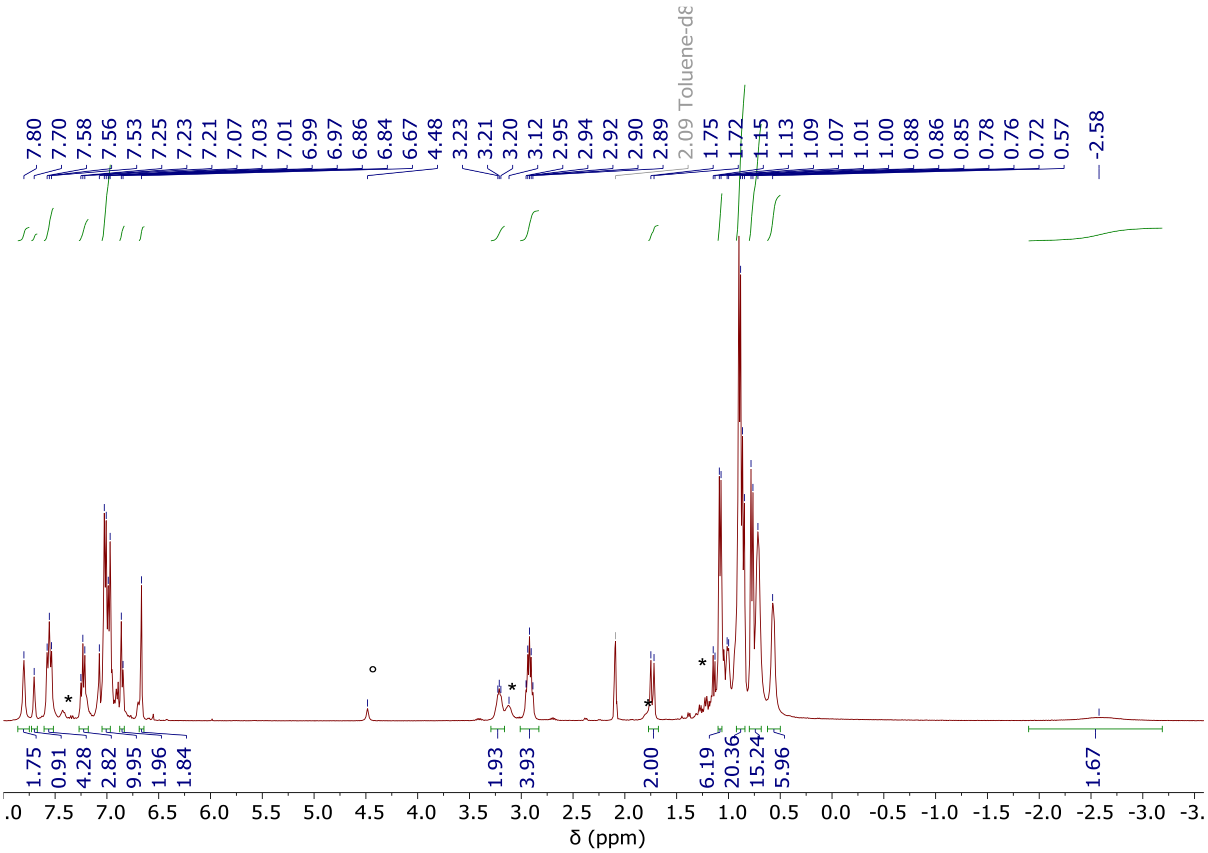

Figure S67. ^{1}H NMR spectrum of **3b** as a solution in toluene-d_8 at 60°C under 1 atm H_2, exhibiting an equilibrium with **4b**; *indicates **3b**; °indicates H_2.

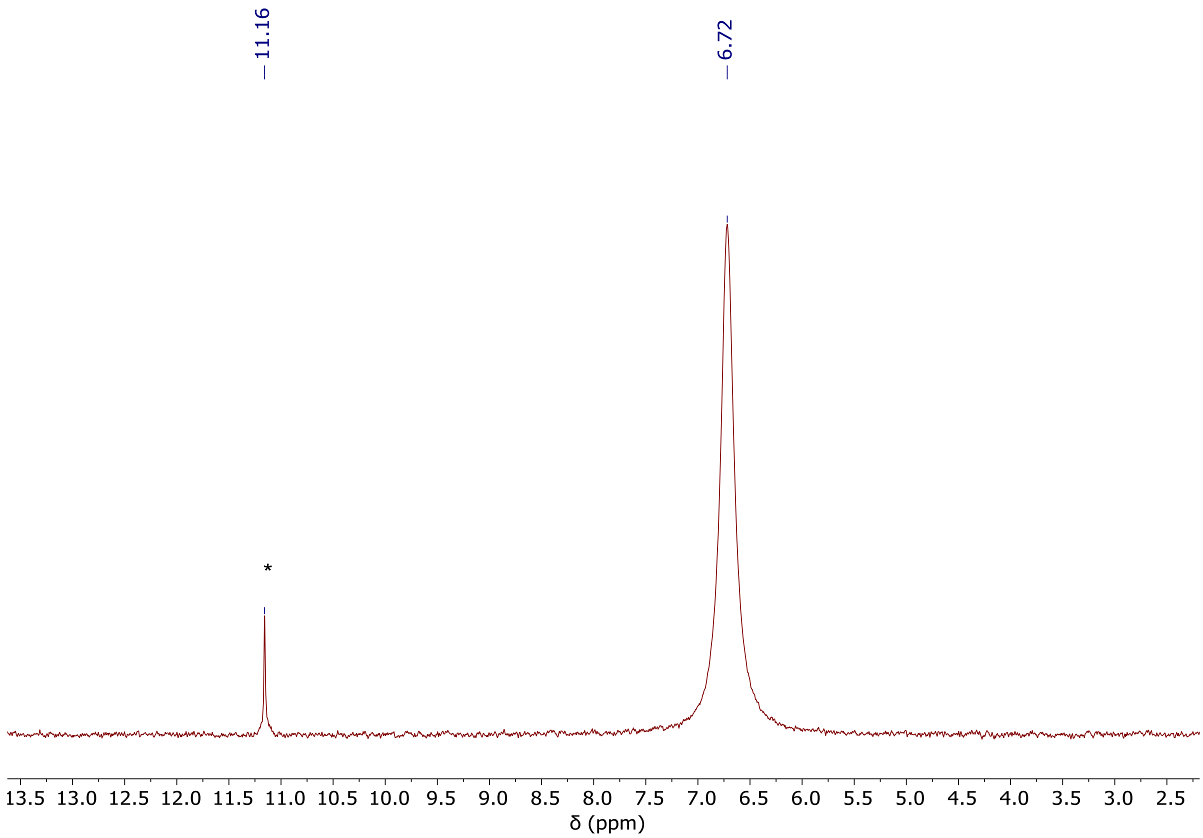

Figure S68. $^{31}P\{^{1}H\}$ NMR spectrum of **3b** as a solution in C_6D_6 at ambient temperature under 1 atm H_2 exhibiting an equilibrium with **4b**; *indicates **3b**.

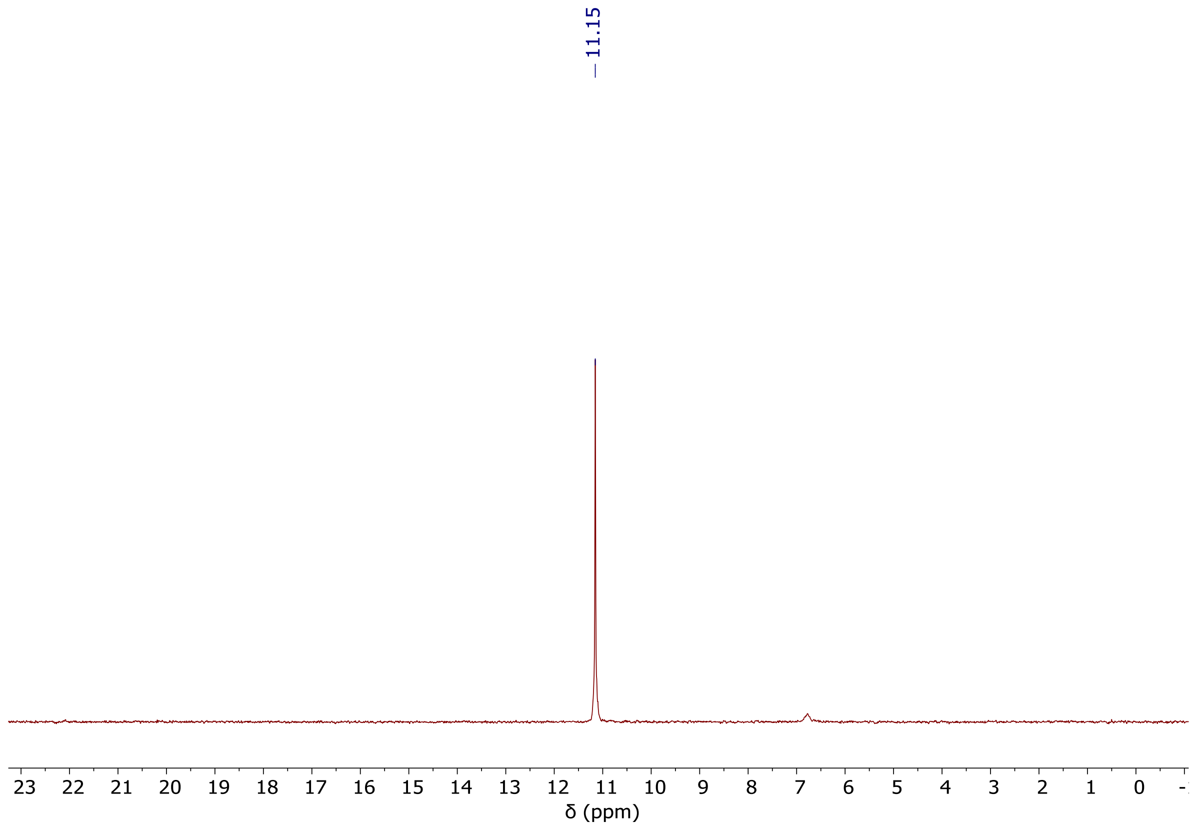

Figure S69. $^{31}P\{^{1}H\}$ NMR spectrum after removing all volatiles of the reaction of **3b** with H_2 *in vacuo*, and redissolving the residue in C_6D_6 showing full regeneration of **3b**.

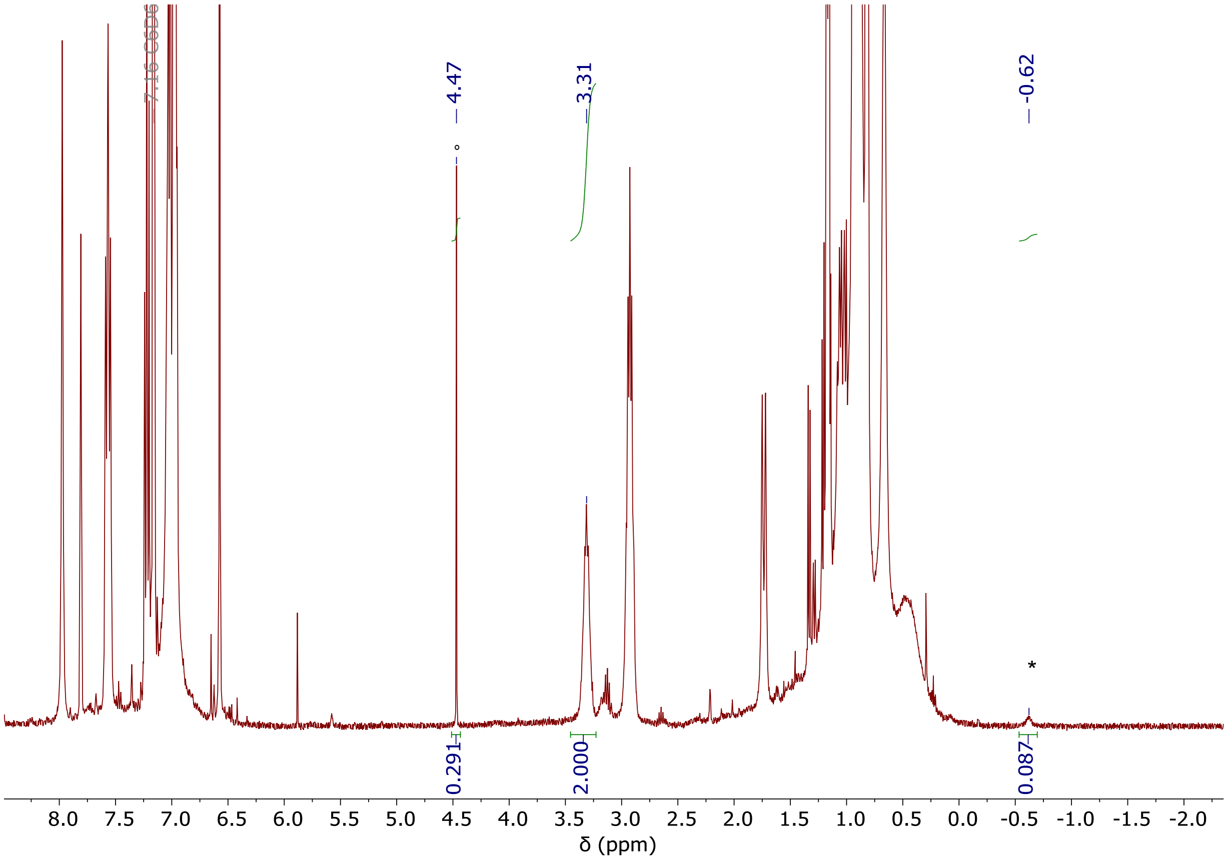

Figure S70. ^{1}H NMR spectrum of 13 mg of **3b** under 1 atm H_2 as a solution in 0.45 mL C_6D_6 at 26°C exhibiting an equilibrium with **4b**; *indicates **3b**; °indicates H_2.

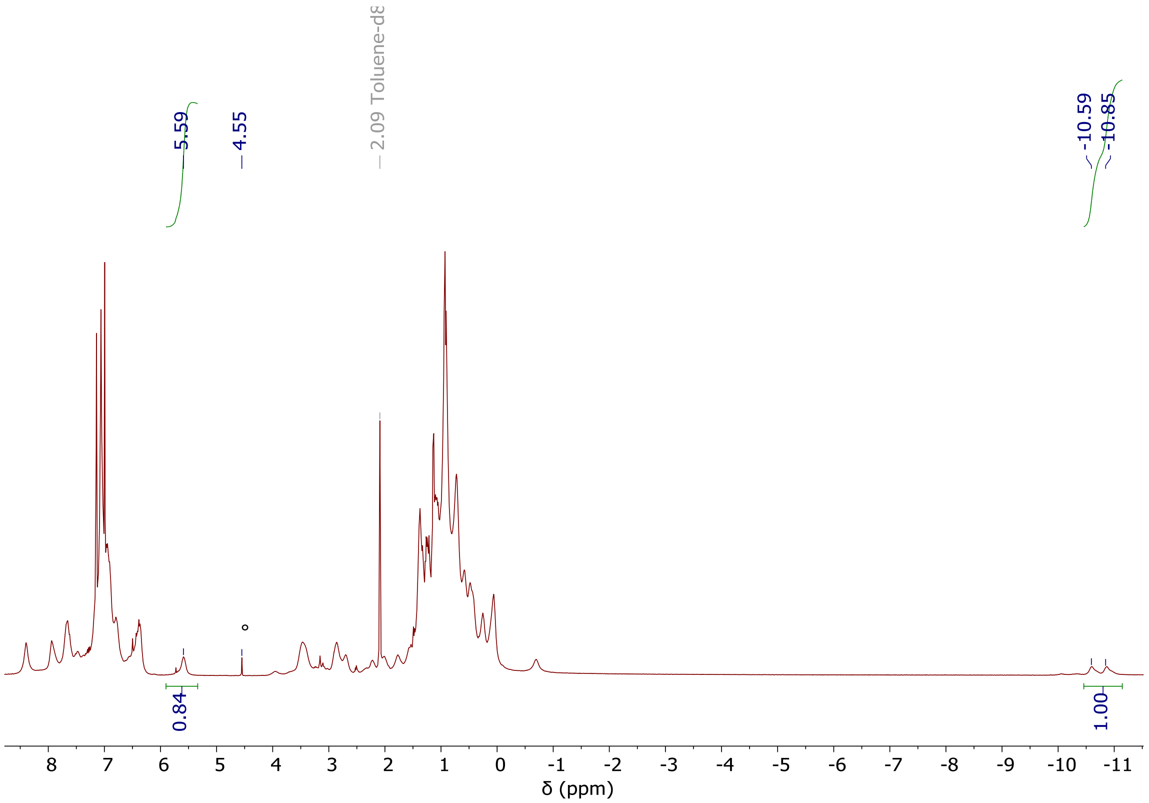

Figure S71. ^{1}H NMR spectrum of **4b** as a solution in toluene-d$_8$ at -60°C under 1 atm H_2 showing the Ge-*H* and Ni-*H* hydride signals. °indicates H_2.

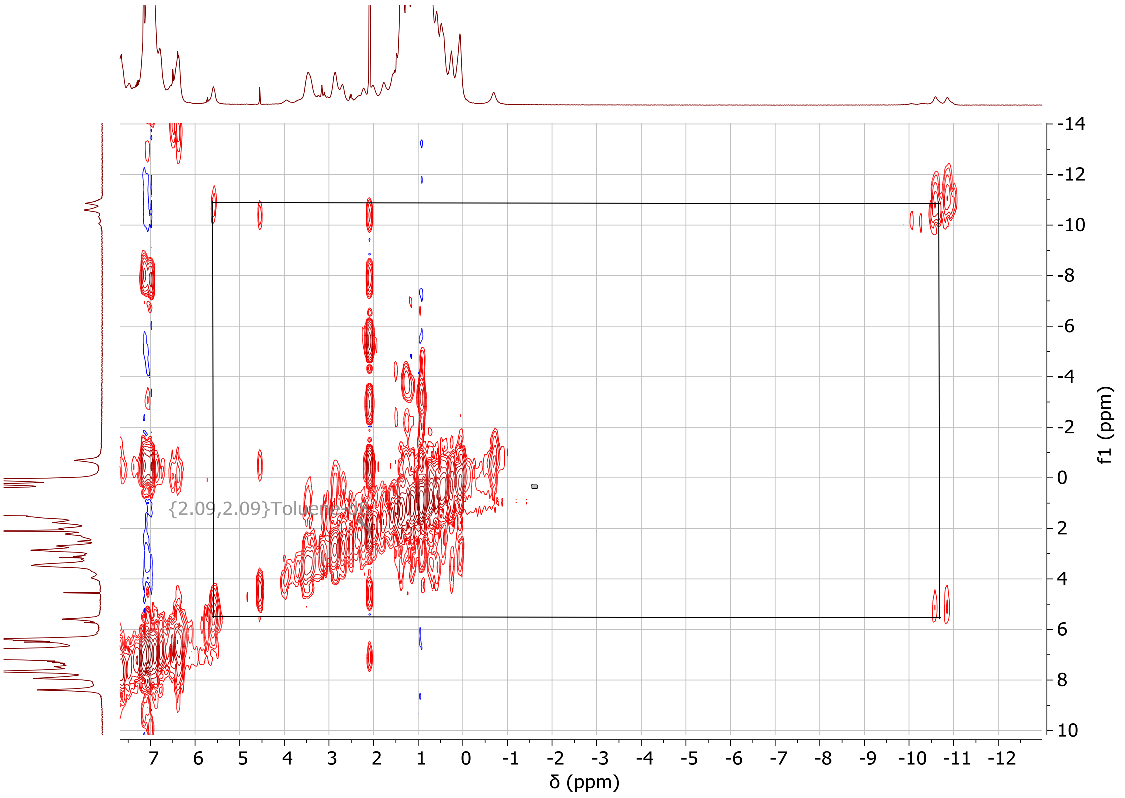

Figure S72. 2D H-H COSY NMR spectrum of the reaction of **3b** under 1 atm H_2 as a solution in toluene-d$_8$ at -60°C showing the correlation of the assumed Ge-*H* and Ni-*H* hydride peaks.

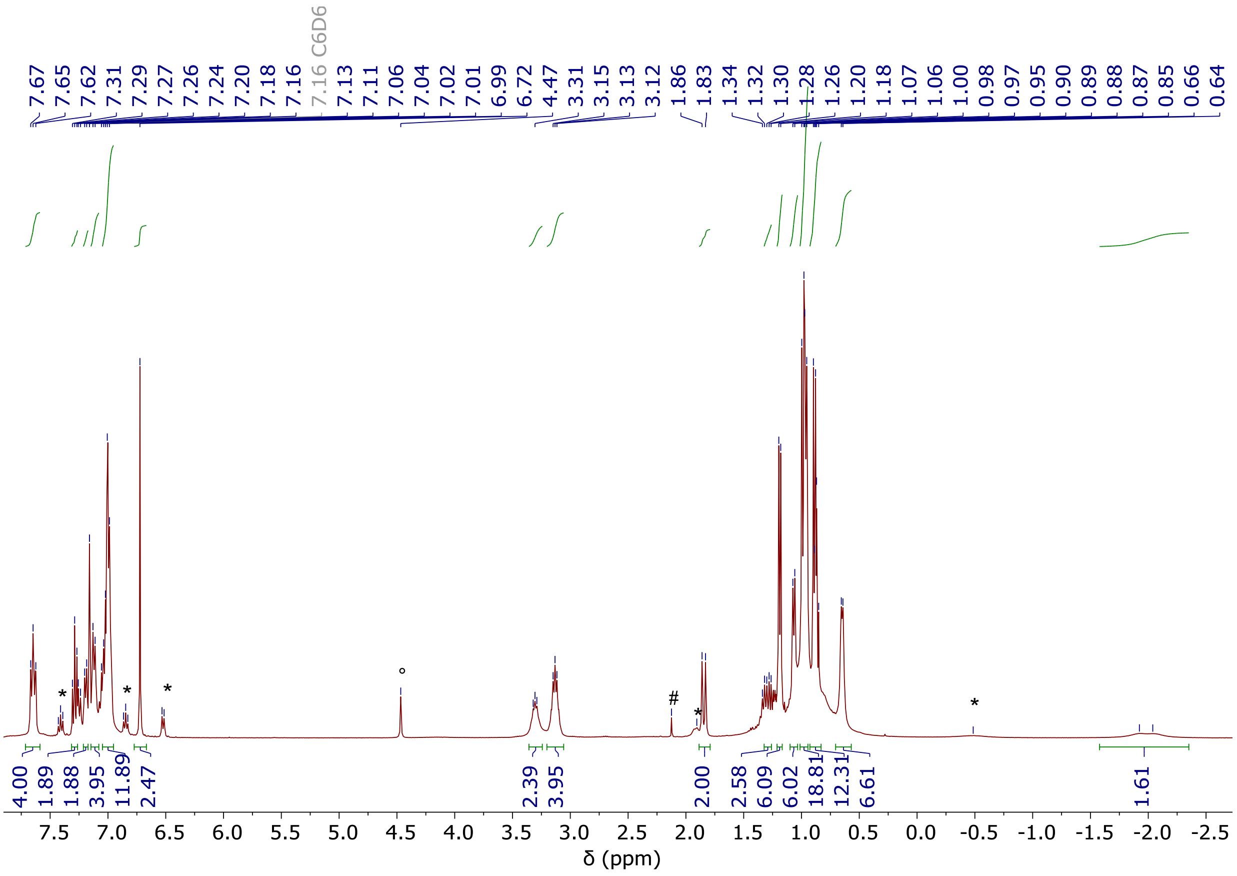

Figure S73. ^{1}H NMR spectrum of **3c** as a solution in C_6D_6 at 60°C under 1 atm H_2, exhibiting an equilibrium with **4c**; *indicates **3c**; °indicates H_2, #indicates minor amounts of toluene.

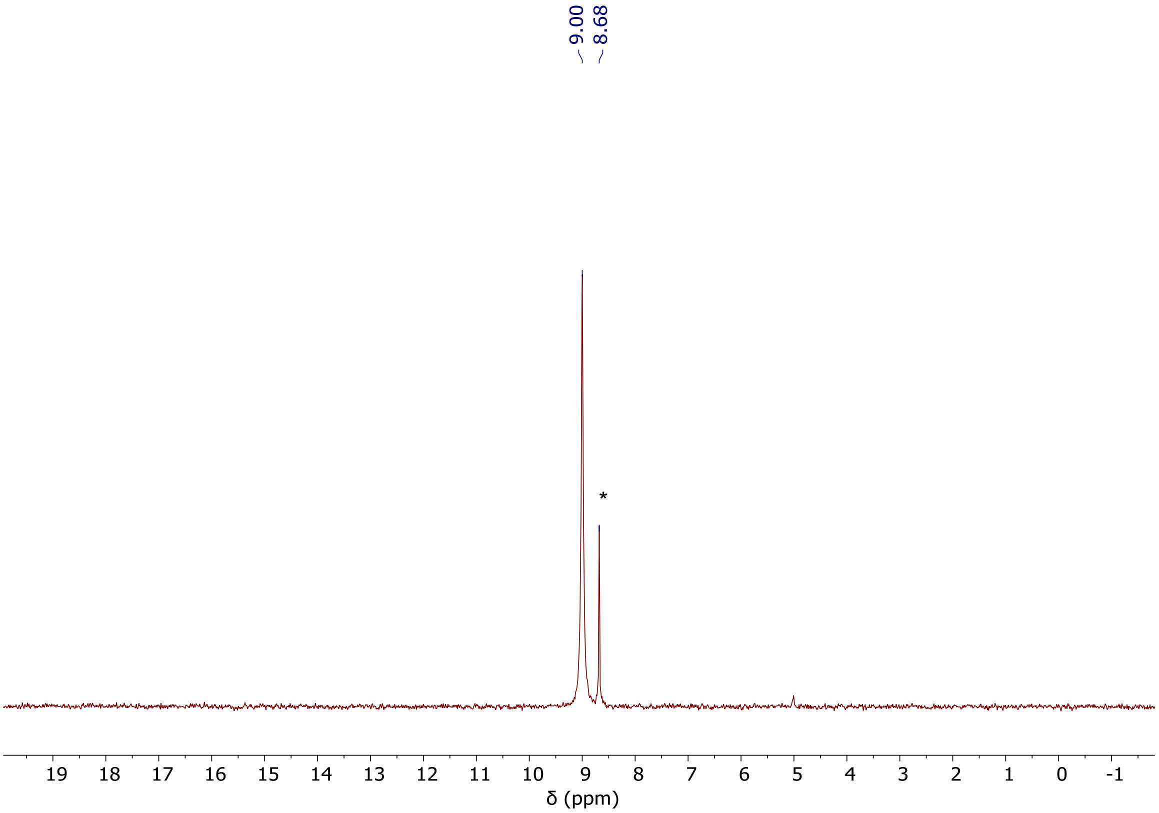

Figure S74. $^{31}P\{^{1}H\}$ NMR spectrum of **3c** as a solution in C_6D_6 at ambient temperature under 1 atm H_2, exhibiting an equilibrium with **4c**; *indicates **3c**.

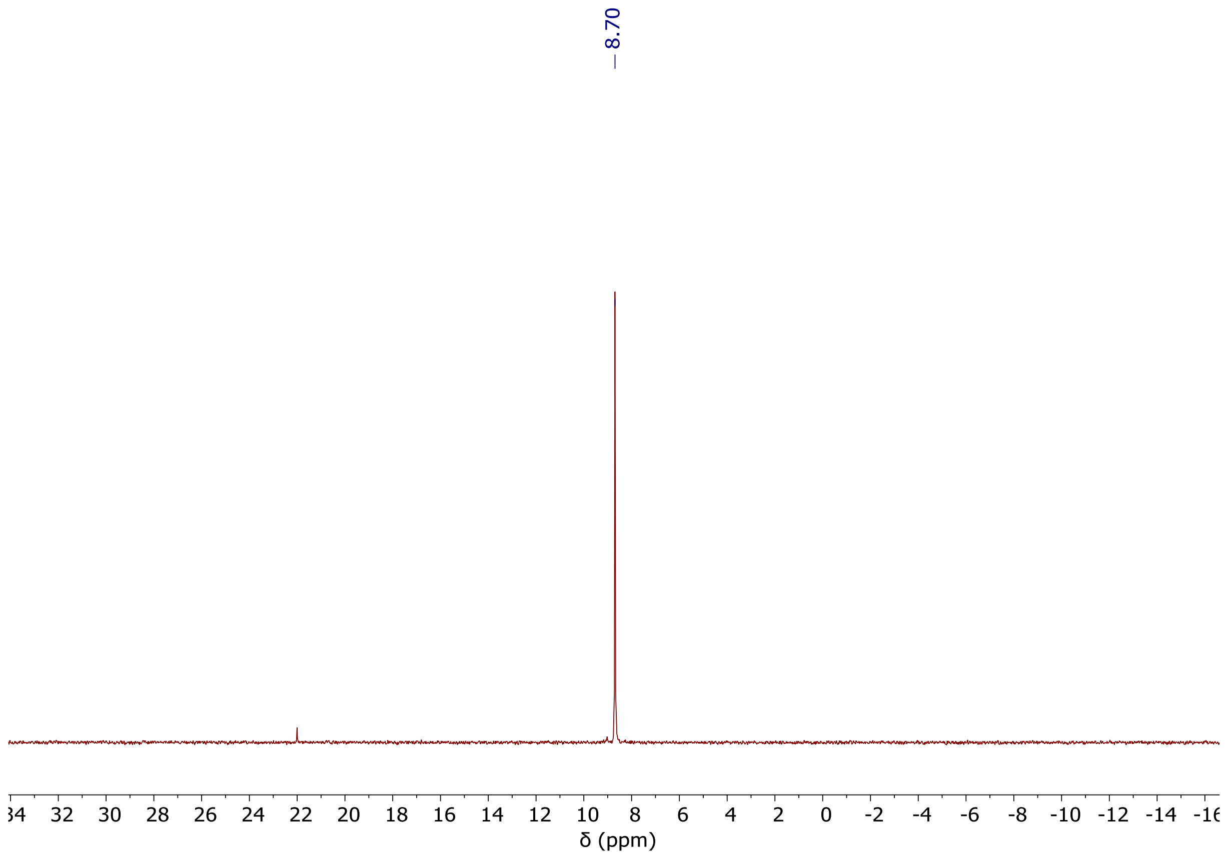

Figure S75. $^{31}P\{^1H\}$ NMR spectrum after removing all volatiles of the reaction of **3c** with H_2 *in vacuo*, and redissolving the residue in C_6D_6 showing full regeneration of **3c**.

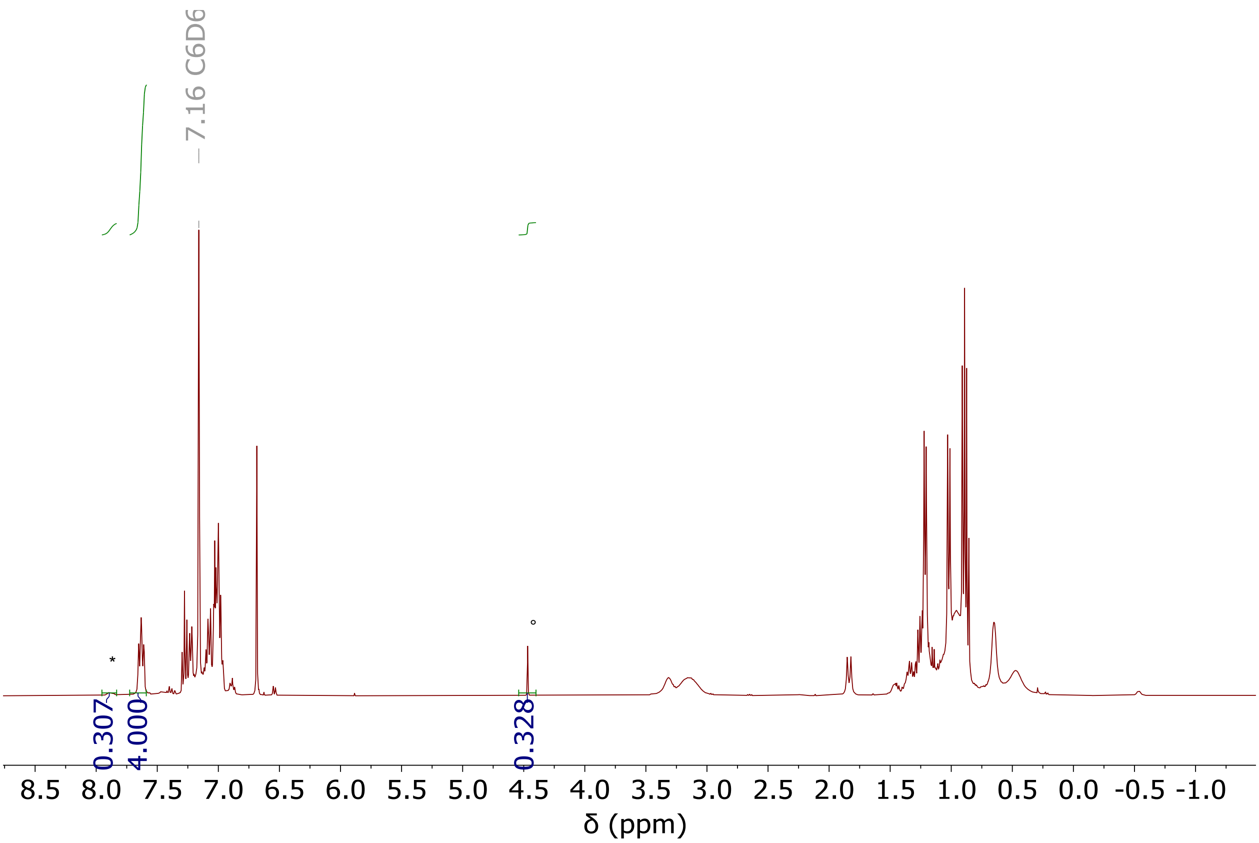

Figure S76. ^1H NMR spectrum of 13 mg of **3c** under 1 atm H_2 as a solution in 0.45 mL C_6D_6 at 26°C exhibiting an equilibrium with **4c**; *indicates **3c**; °indicates H_2.

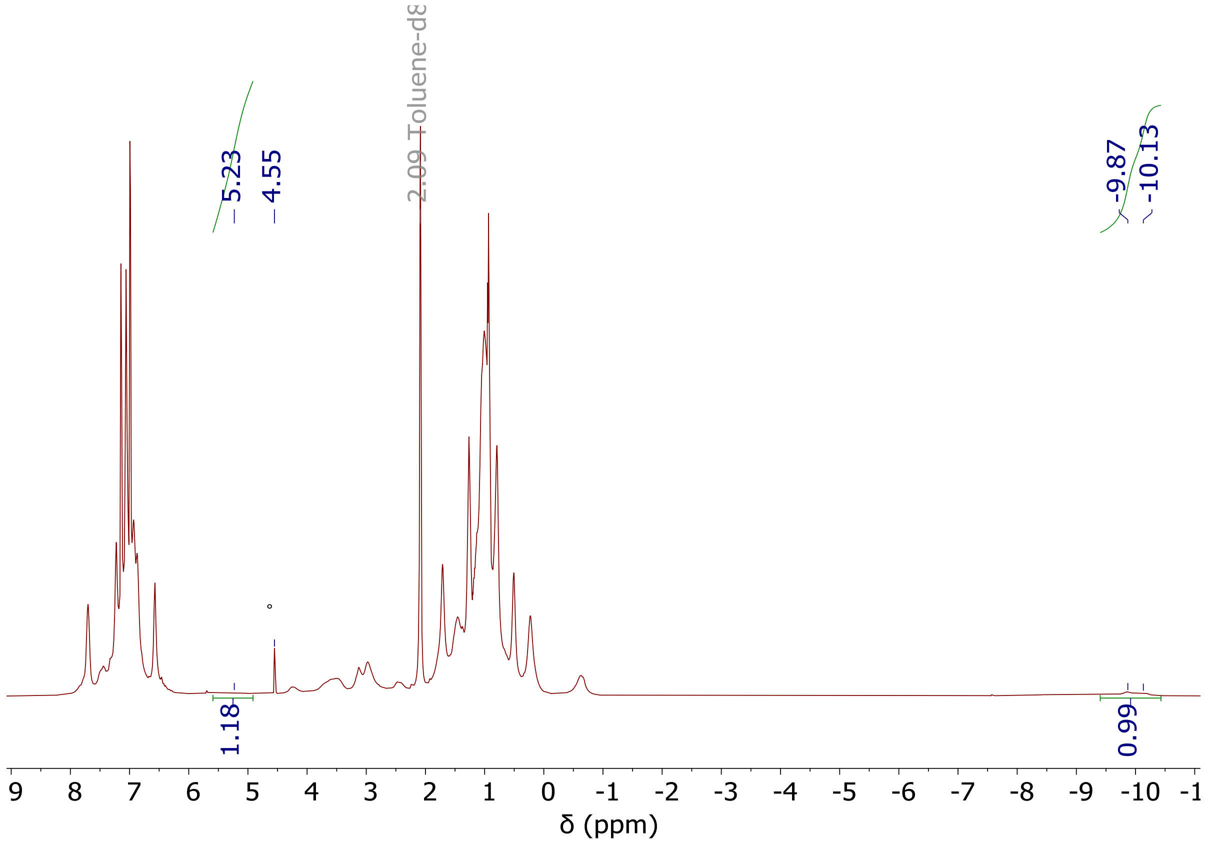

Figure S77. ^1H NMR spectrum of **3c** as a solution in toluene-d_8 at -80°C under 1 atm H_2, showing the Ge-*H* and Ni-*H* hydride signals. °indicates H_2.

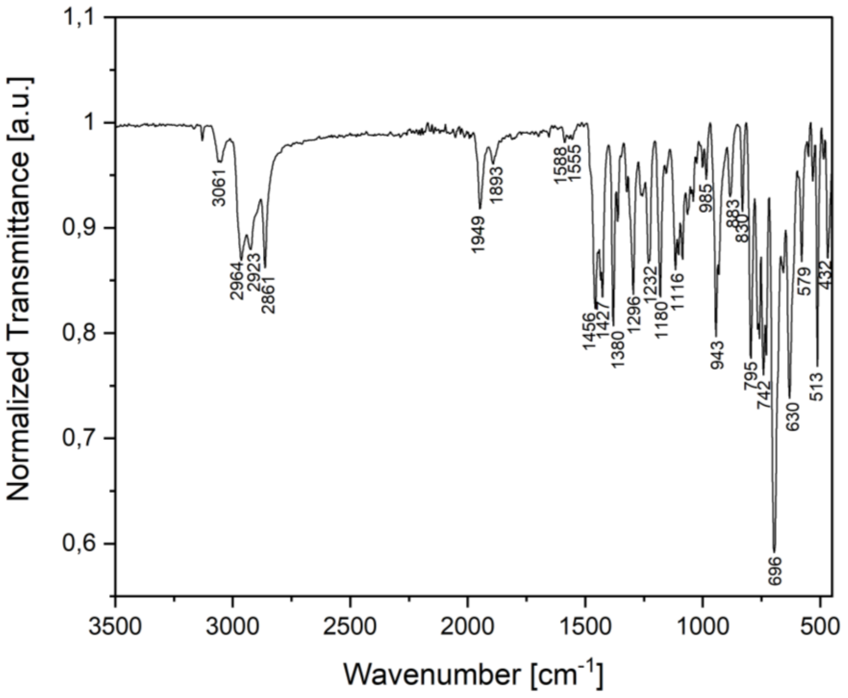

Figure S78. ATR-IR spectrum of solid crystalline **4c** at ambient temperature.

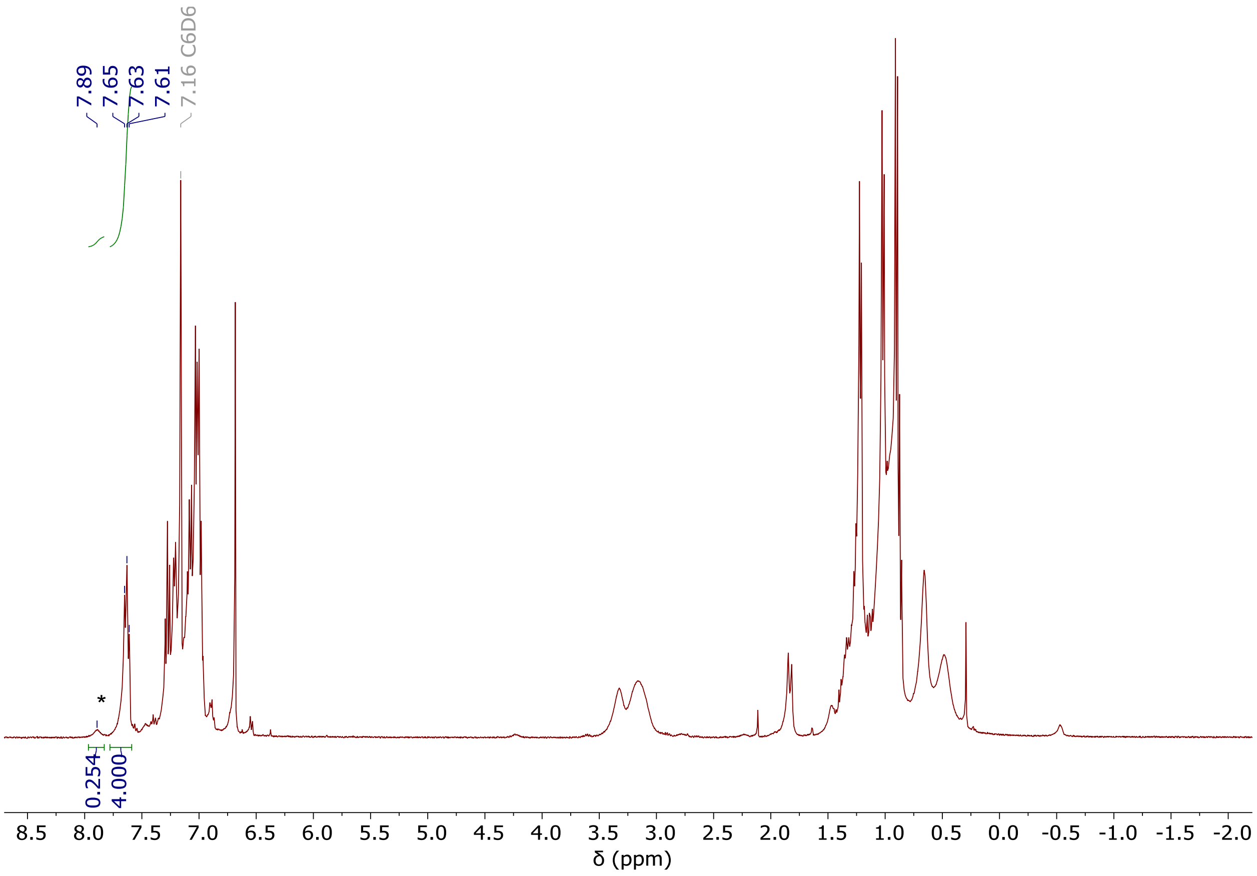

Figure S79. ^{1}H NMR spectrum of 13 mg of **3c** under 1 atm D_2 as a solution in 0.45 mL C_6D_6 at 26°C exhibiting an equilibrium with **4c-D$_2$**; *indicates **3c**.

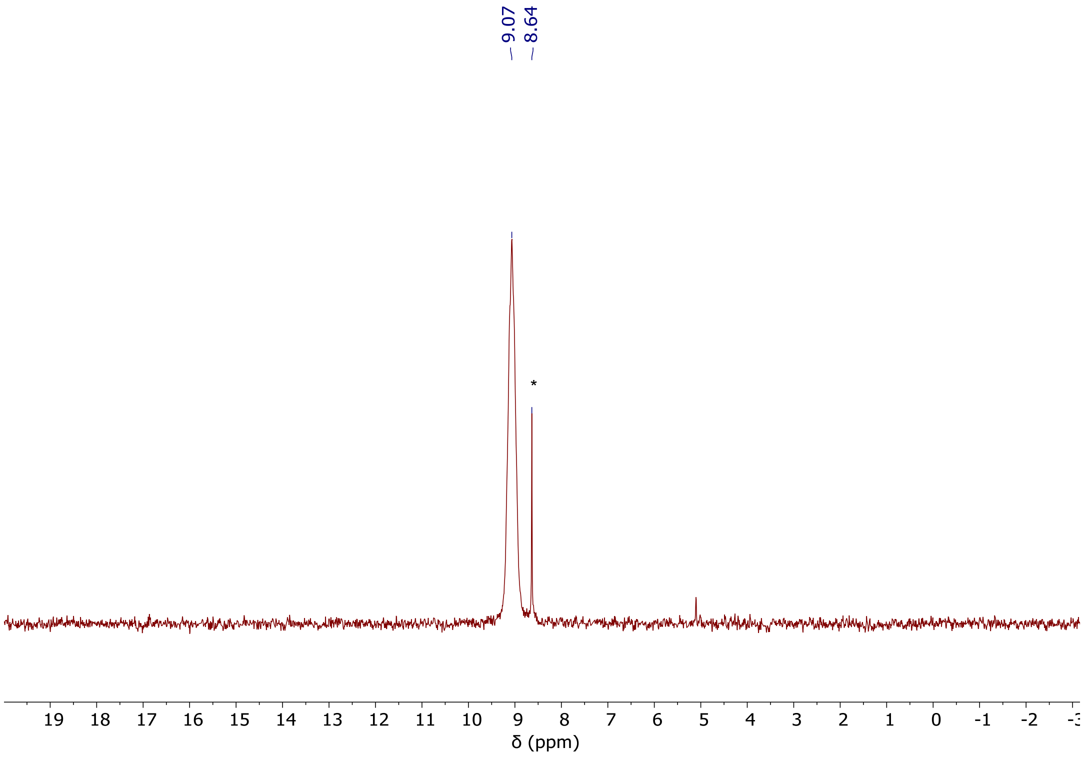

Figure S80. Crude $^{31}P\{^{1}H\}$ NMR spectrum of the reaction of **3c** with D_2 as a solution in toluene-d_8 at ambient temperature showing an equilibrium with **4c-D$_2$**; *indicates **3c.**

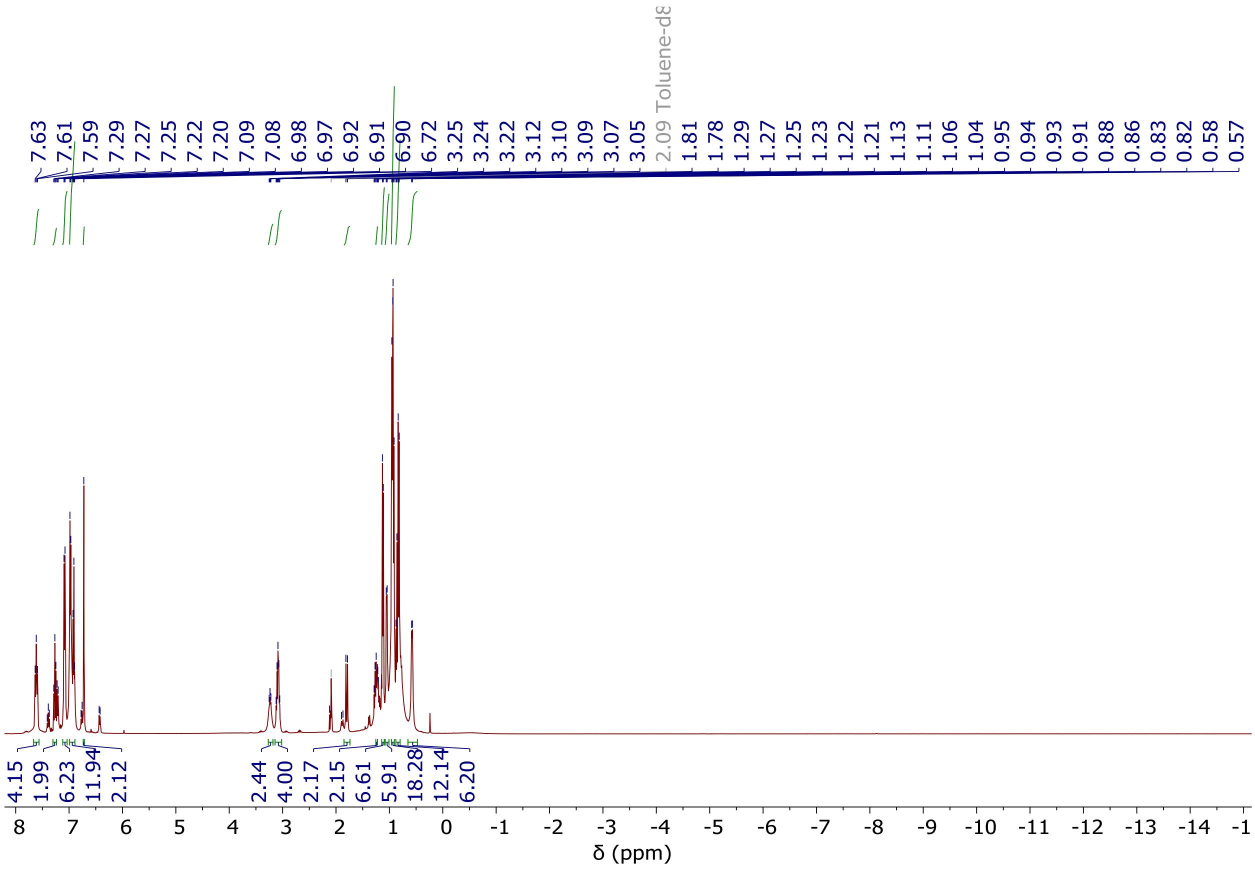

Figure S81. ^{1}H NMR spectrum of **3c** under 1 atm D_2 as a solution in toluene-d_8 at 60°C.

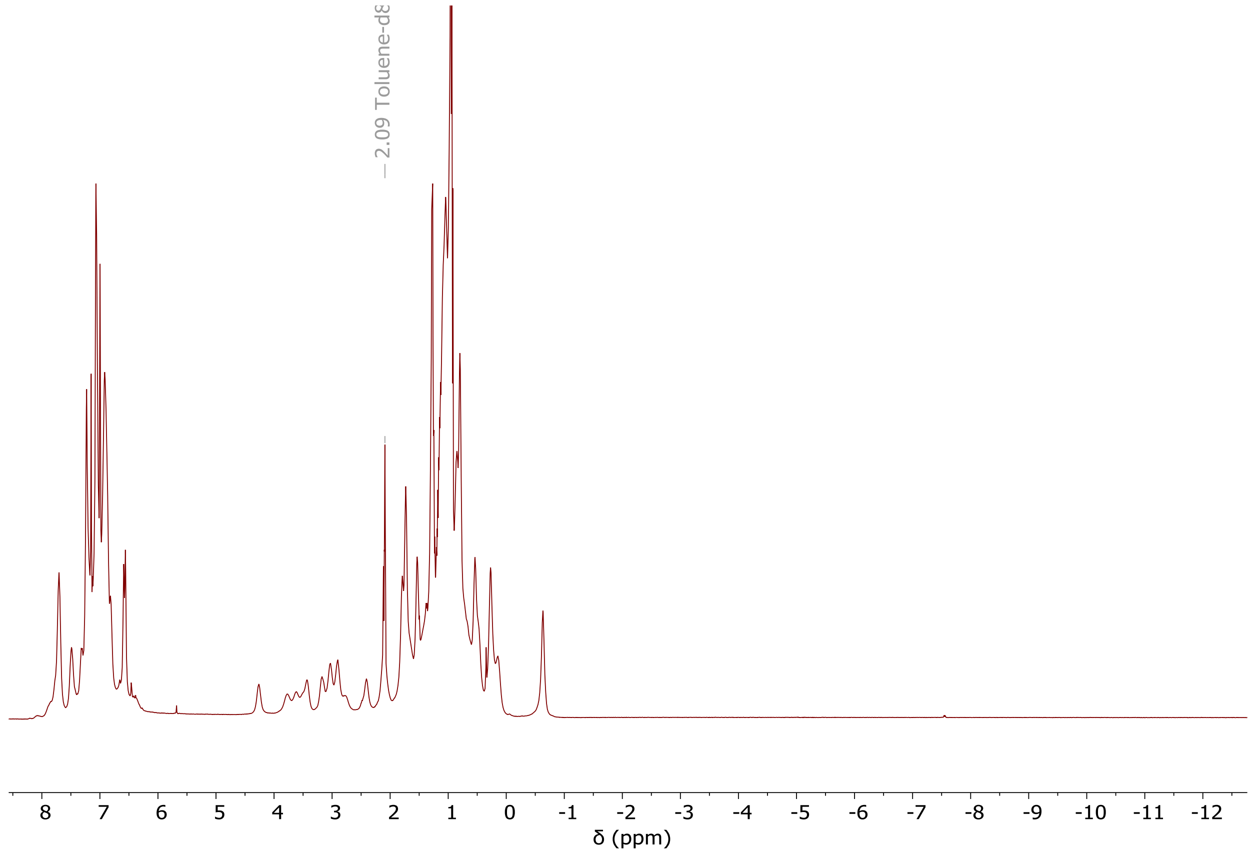

Figure S82. ^{1}H NMR spectrum of **3c** under 1 atm D_2 as a solution in toluene-d_8 at -80°C.

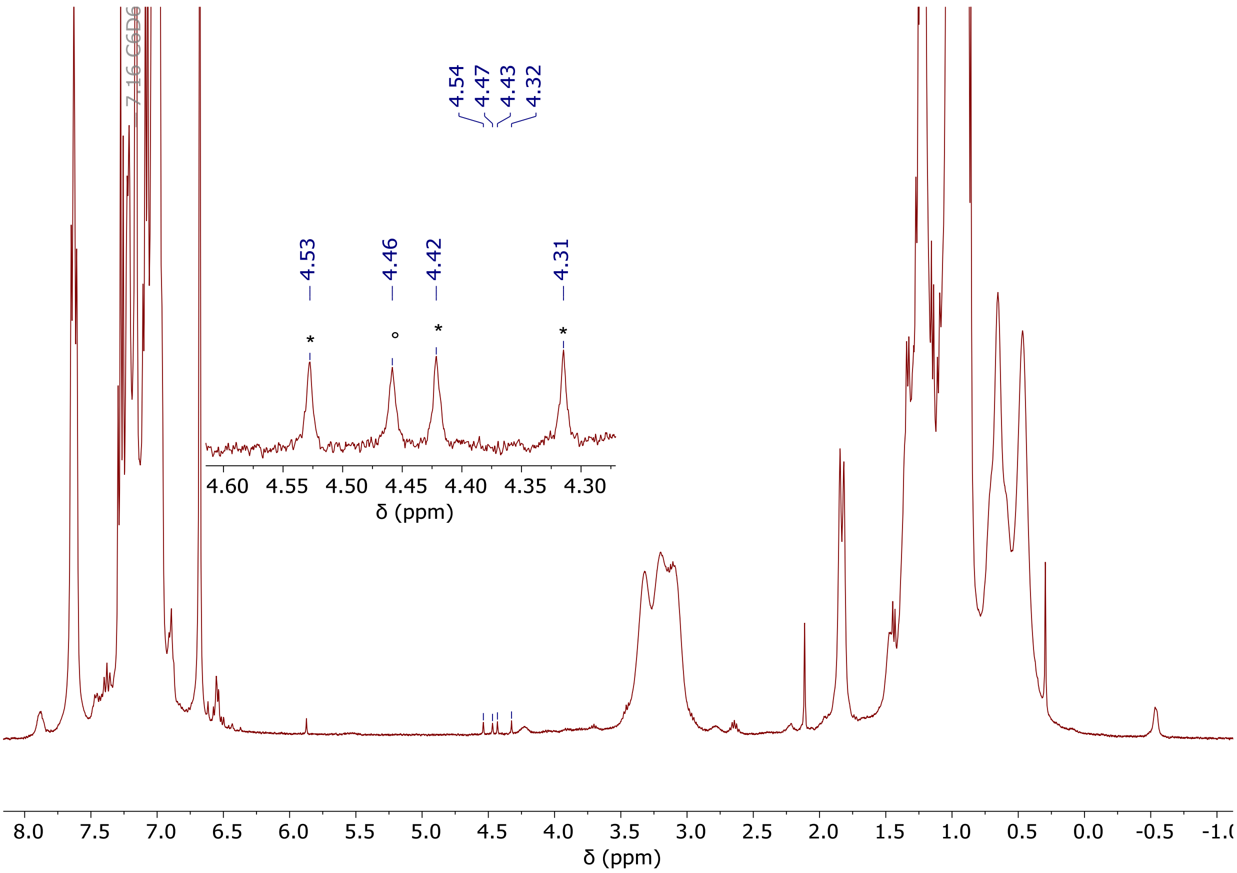

Figure S83. ^{1}H NMR spectrum of **3c** under H_2/D_2 as a solution in C_6D_6 at ambient temperature resulting in the formation of H-D gas; * indicates H-D; °indicates H_2.

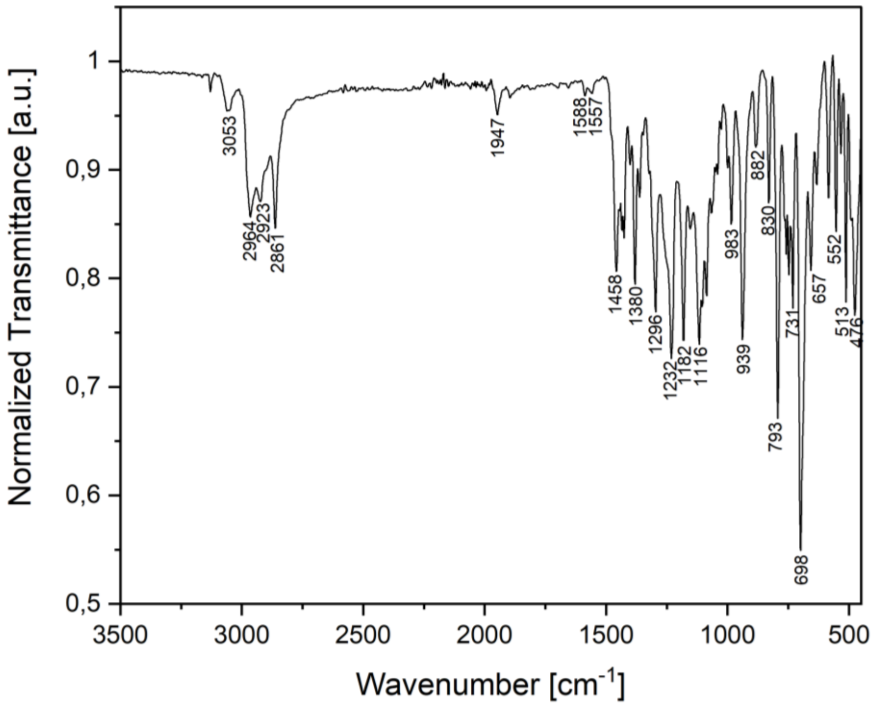

Figure S84. ATR-IR spectrum of solid crystalline **4c-D₂** at ambient temperature.

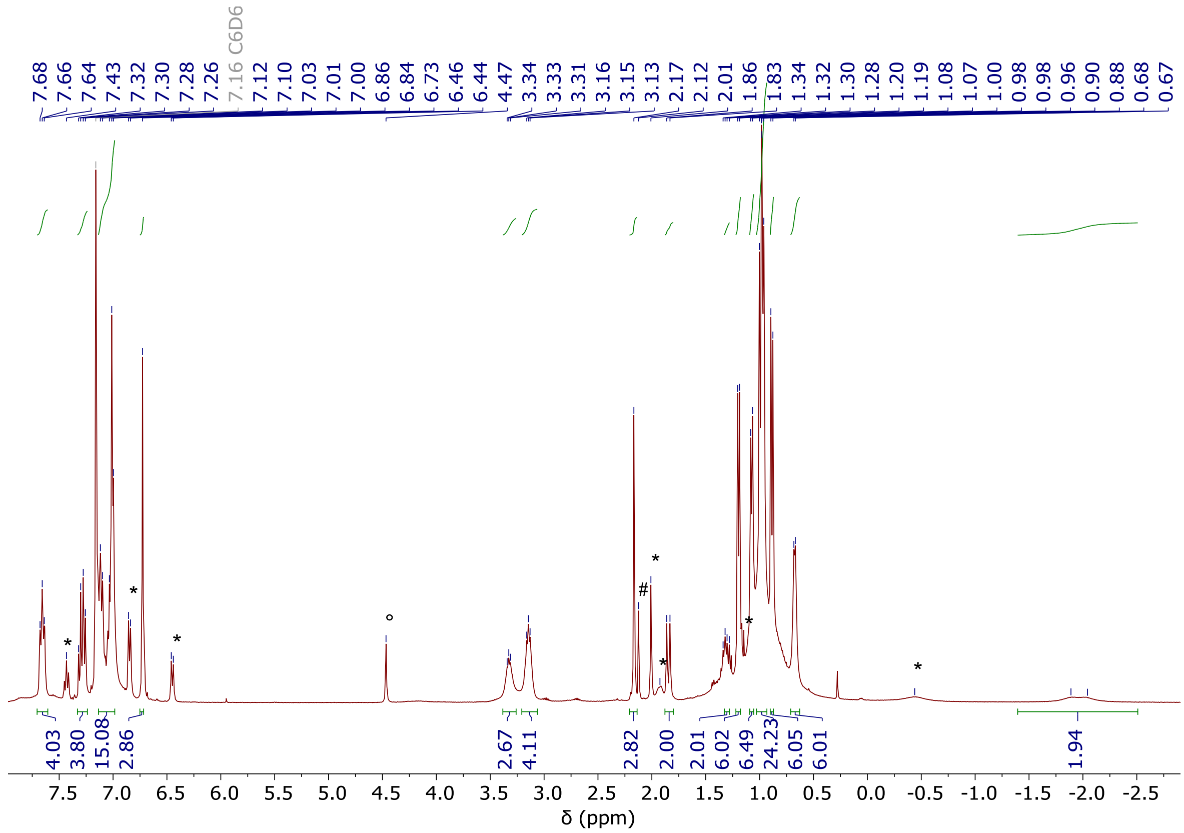

Figure S85. ^{1}H NMR spectrum of **3d** as a solution in C_6D_6 at 60°C under 1 atm H_2 exhibiting an equilibrium with **4d**; *indicates **3d**; °indicates H_2, #indicates minor amounts of toluene.

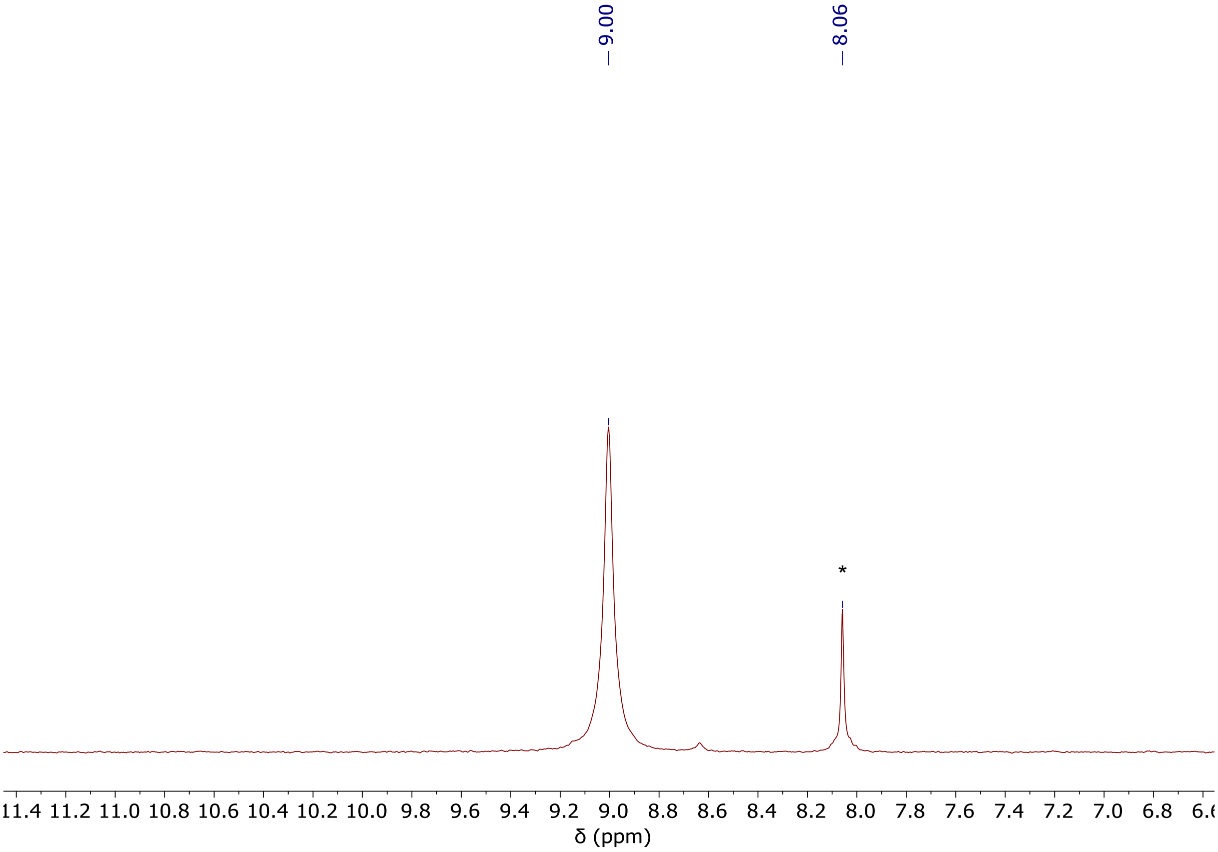

Figure S86. $^{31}P\{^{1}H\}$ NMR spectrum of **3d** as a solution in C_6D_6 at ambient temperature under 1 atm H_2 exhibiting an equilibrium with **4d**; *indicates **3d**.

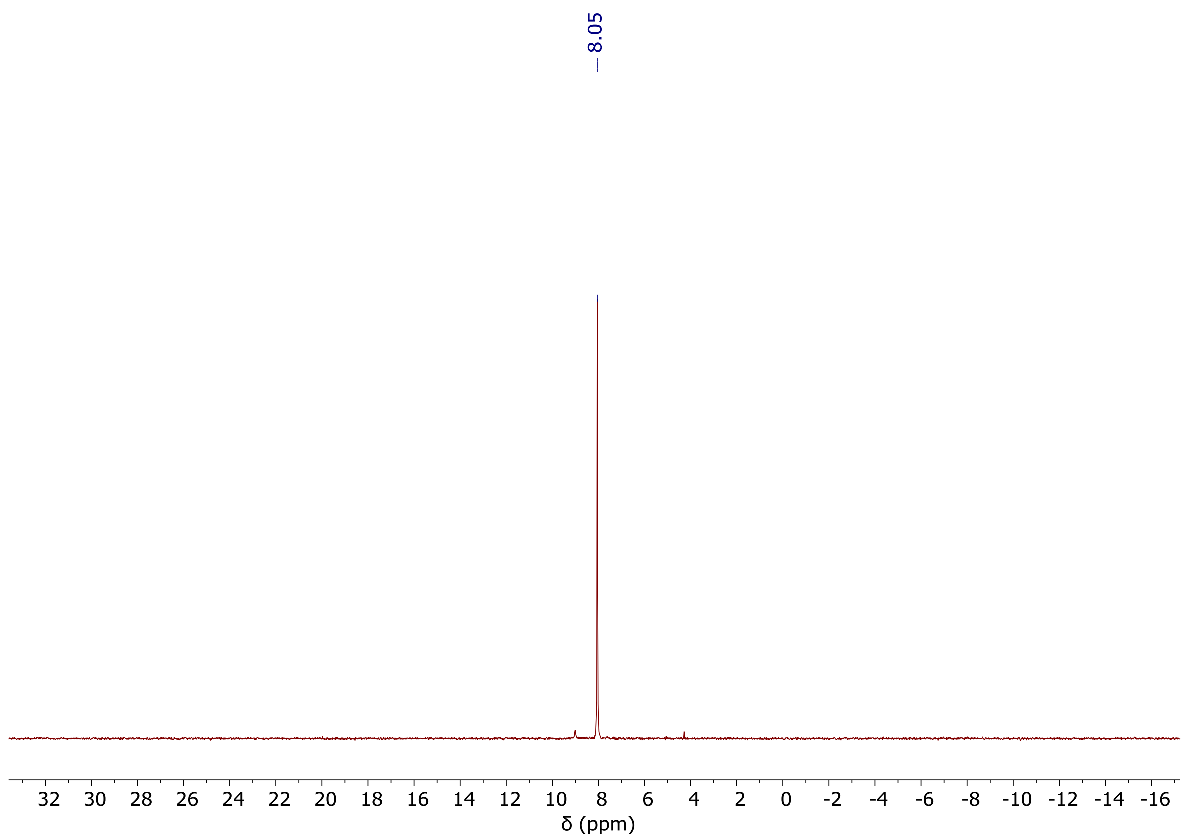

Figure S87. $^{31}P\{^{1}H\}$ NMR spectrum after removing all volatiles of the reaction of **4d** with H_2 *in vacuo* and redissolving the residue in C_6D_6 showing full regeneration of **4d**.

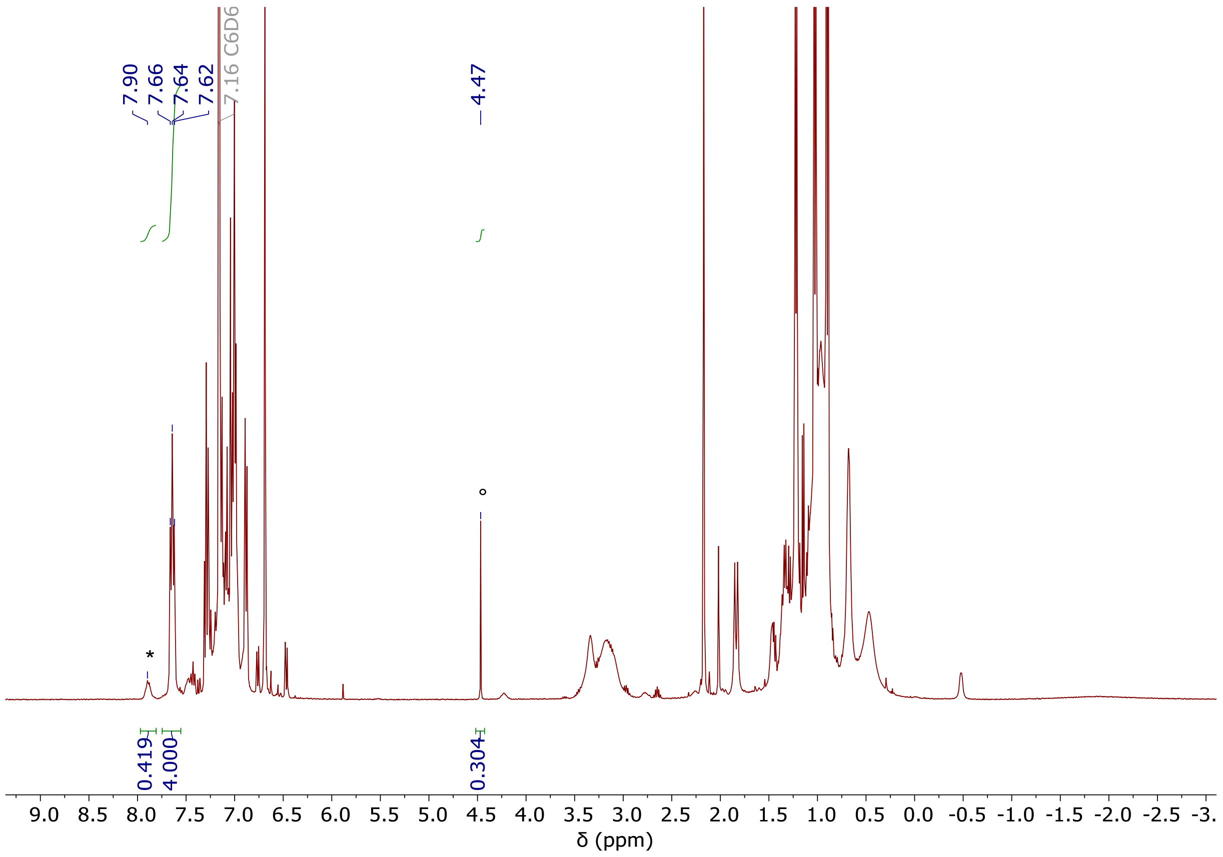

Figure S88. ^{1}H NMR spectrum of 13 mg of **3d** under 1 atm H_2 as a solution in 0.45 mL C_6D_6 at 26°C exhibiting an equilibrium with **4d**; *indicates **3d**; °indicates H_2.

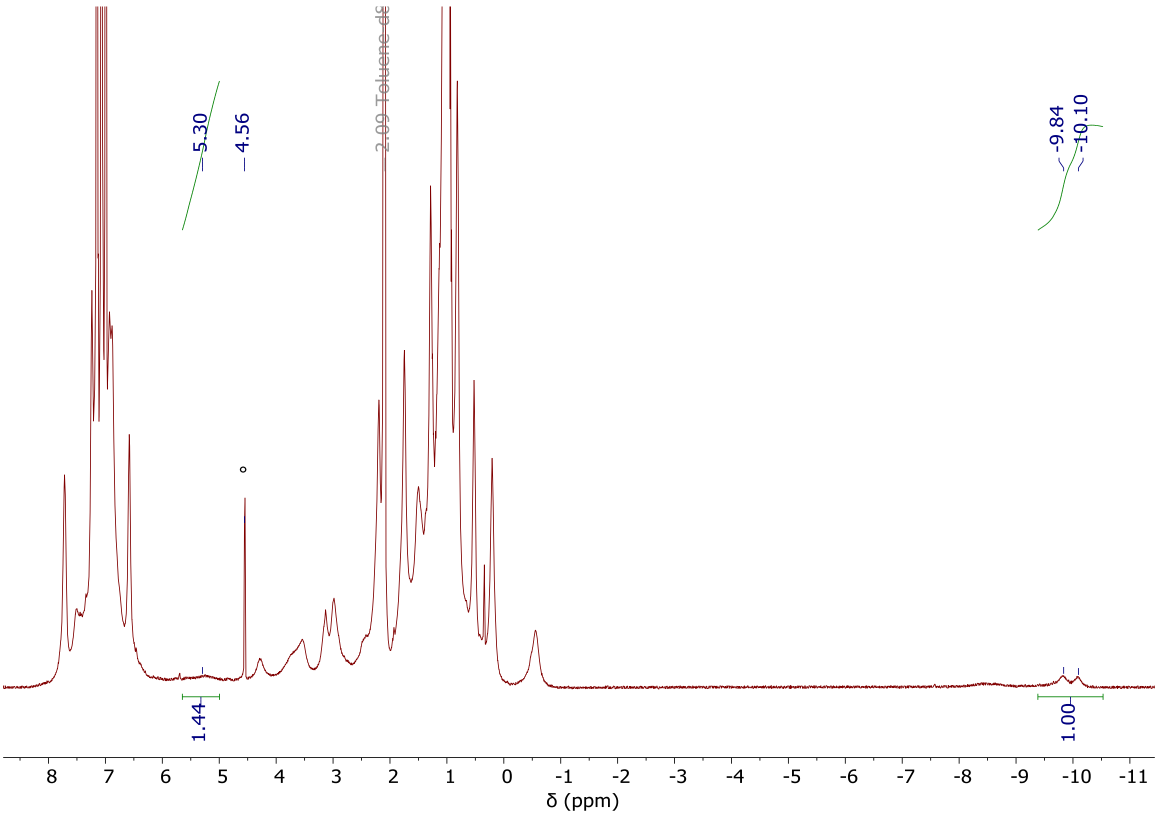

Figure S89. ^{1}H NMR spectrum of **3d** as a solution in toluene-d_8 at -80°C under 1 atm H_2 showing the Ge-*H* and Ni-*H* hydride peaks. °indicates H_2.

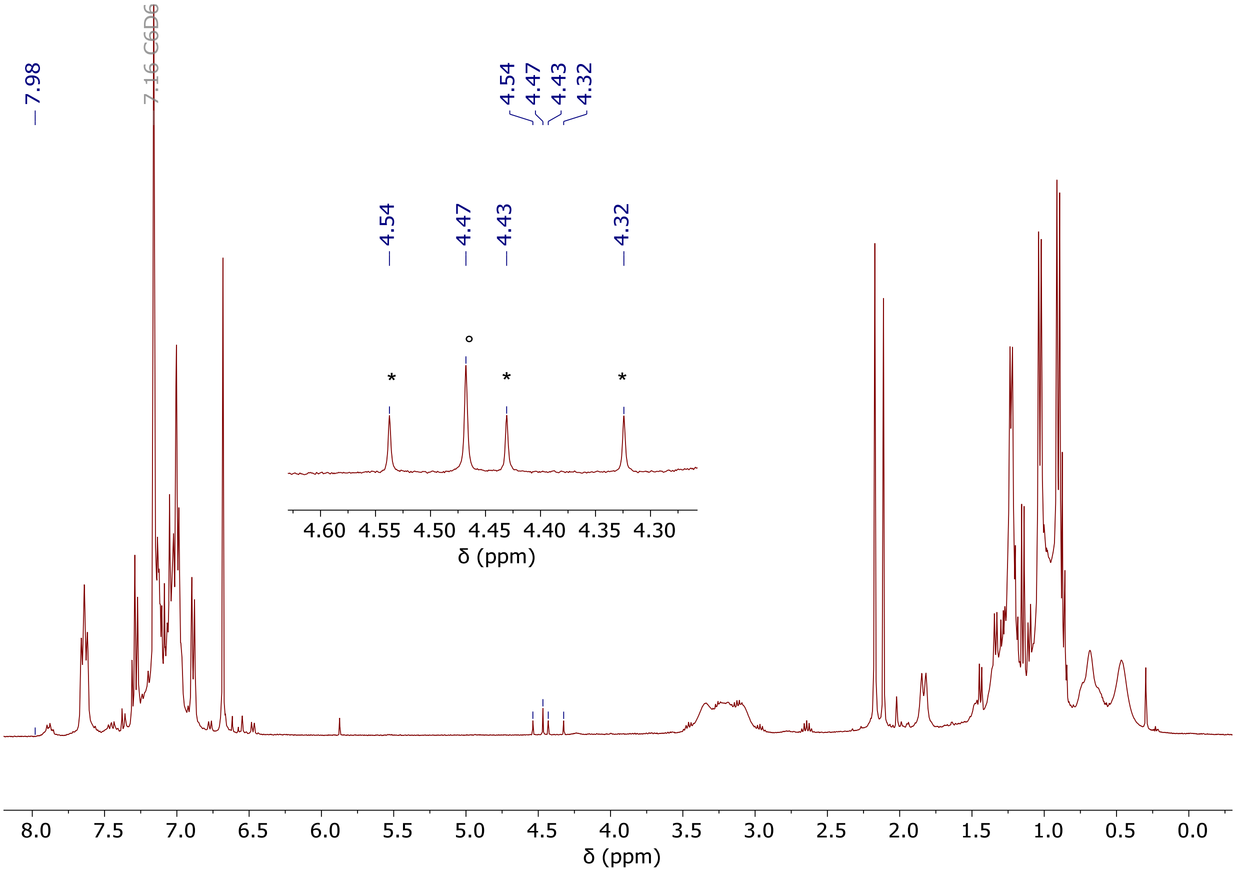

Figure S90. ^{1}H NMR spectrum of **3d** under H_2/D_2 as a solution in C_6D_6 at ambient temperature resulting in the formation of H-D gas; * indicates H-D; °indicates H_2.

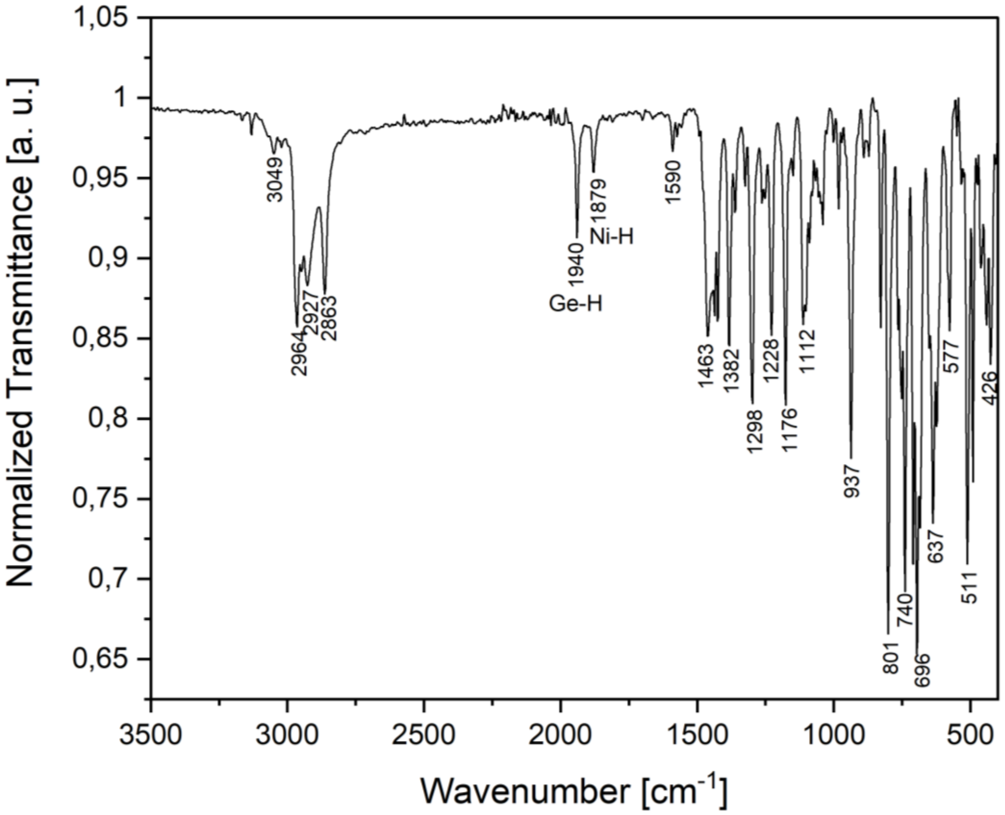

Figure S91. ATR-IR spectrum of solid crystalline **4d** at ambient temperature.

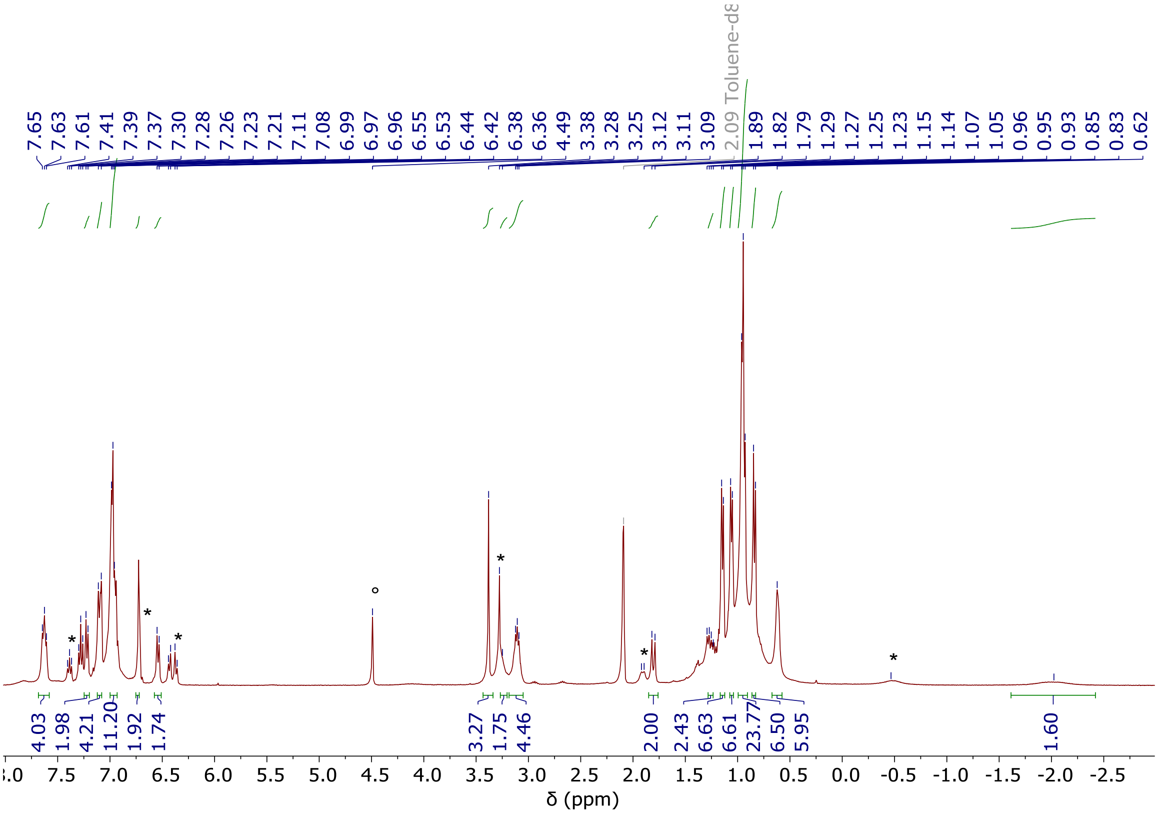

Figure S92. ^{1}H NMR spectrum of **3e** as a solution in toluene-d$_8$ at 60°C under 1 atm H_2 exhibiting an equilibrium with **4e**; *indicates **3e**; °indicates H_2.

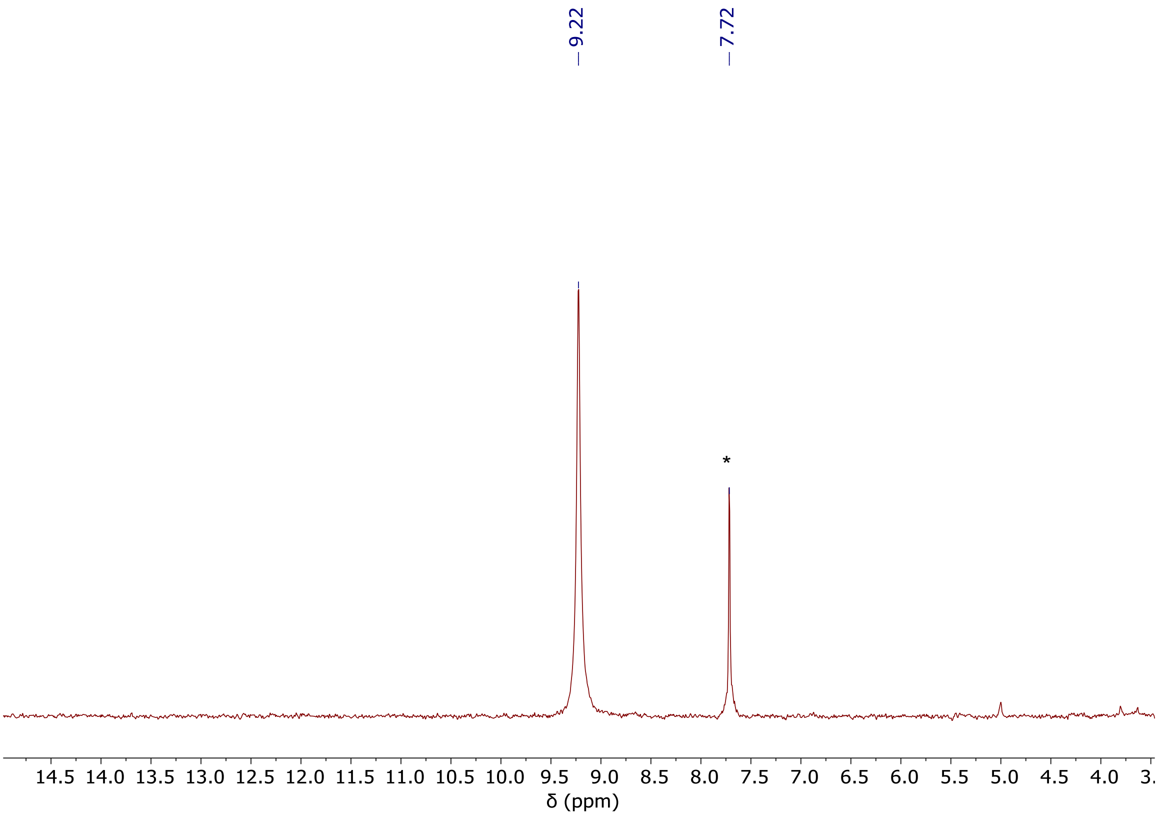

Figure S93. $^{31}P\{^1H\}$ NMR spectrum of **3e** as a solution in C_6D_6 at ambient temperature under 1 atm H_2 exhibiting an equilibrium with **4e**; *indicates **3e**.

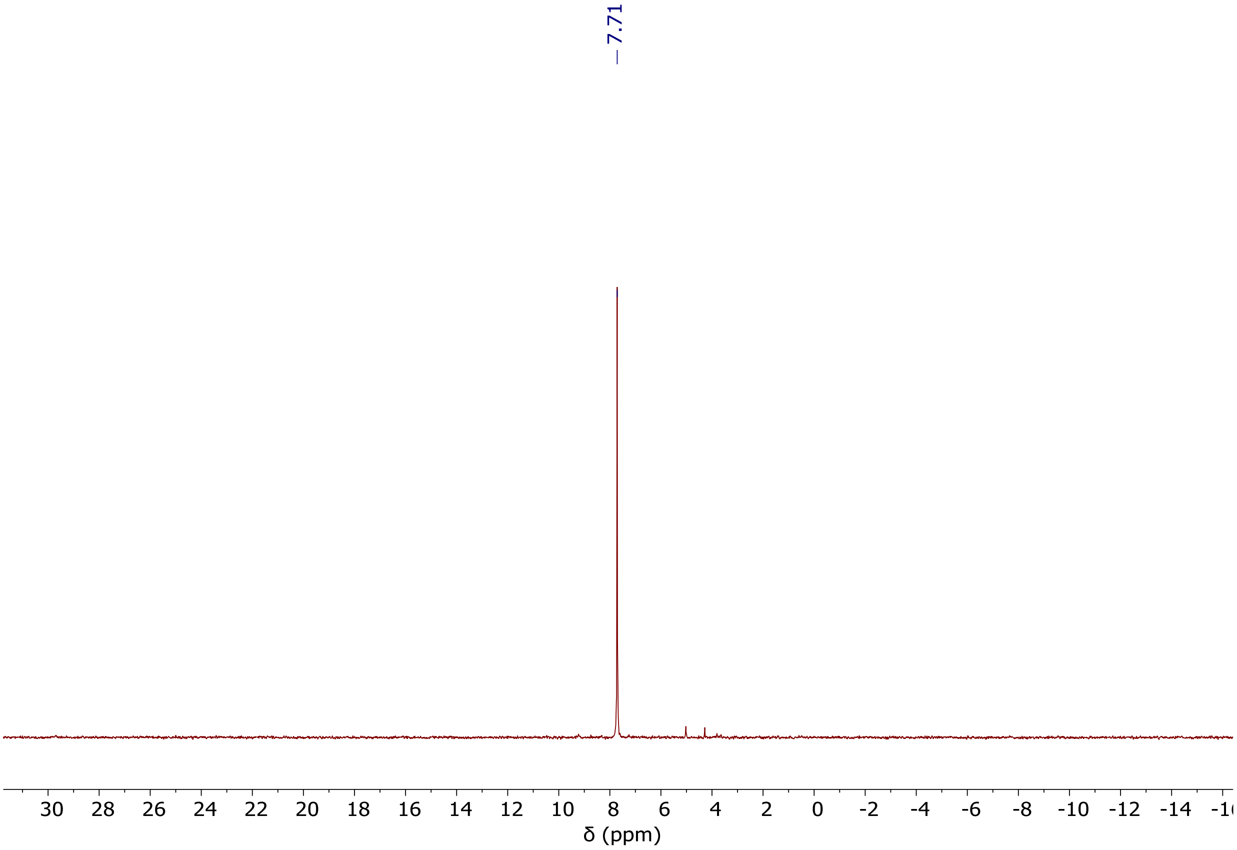

Figure S94. $^{31}P\{^1H\}$ NMR spectrum after removing all volatiles of the reaction of **3e** with H_2 *in vacuo* and redissolving the residue in C_6D_6 showing full regeneration of **3e**.

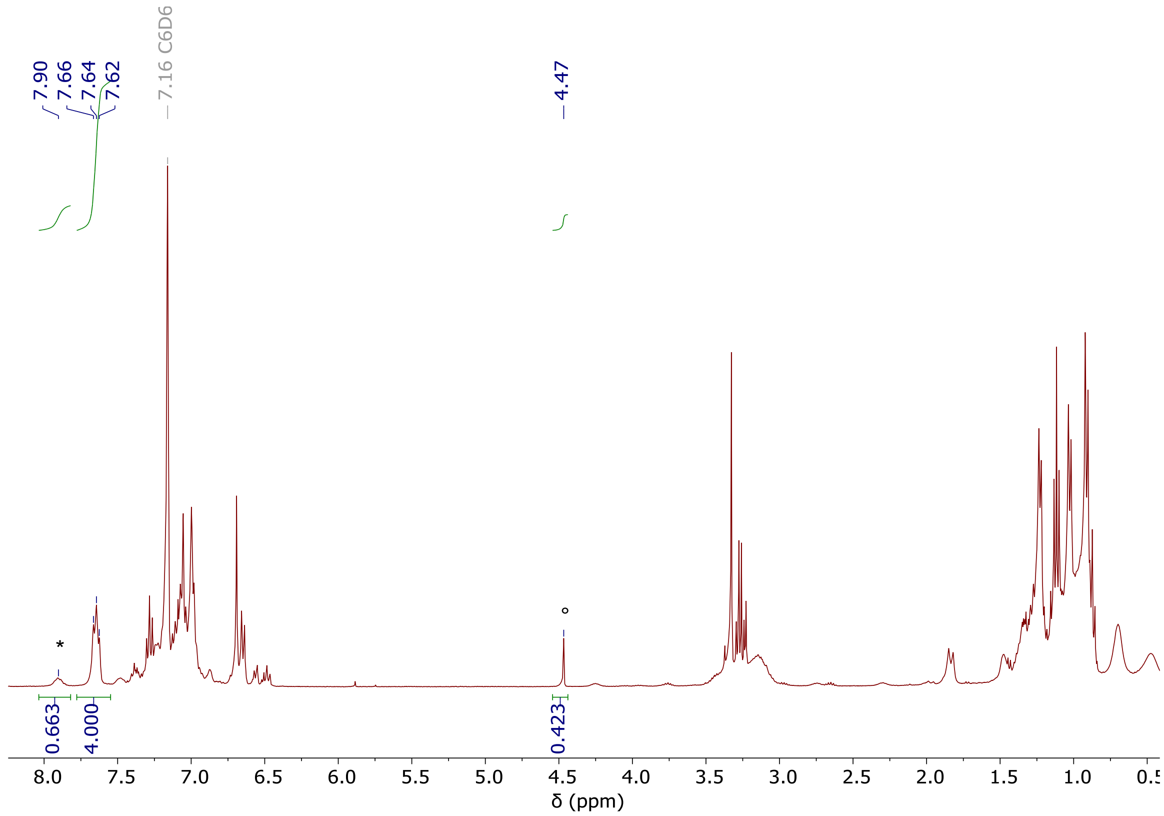

Figure S95. ^{1}H NMR spectrum of 11 mg of **3e** under 1 atm H_2 as a solution in 0.45 mL C_6D_6 at 26°C exhibiting an equilibrium with **4e**; *indicates **3e**; °indicates H_2.

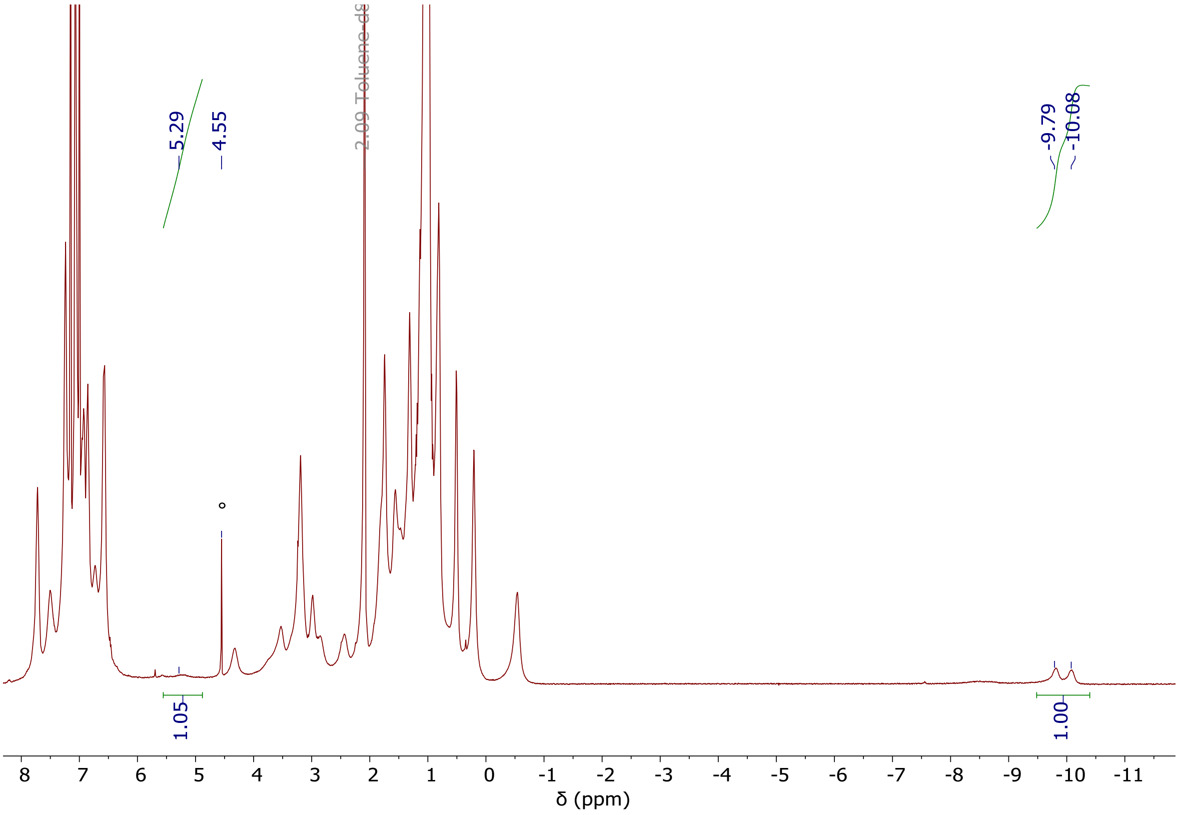

Figure S96. ^{1}H NMR spectrum of **3e** as a solution in THF-d_8 at -40°C under 1 atm H_2 showing the Ge-*H* and Ni-*H* hydride peaks. °indicates H_2.

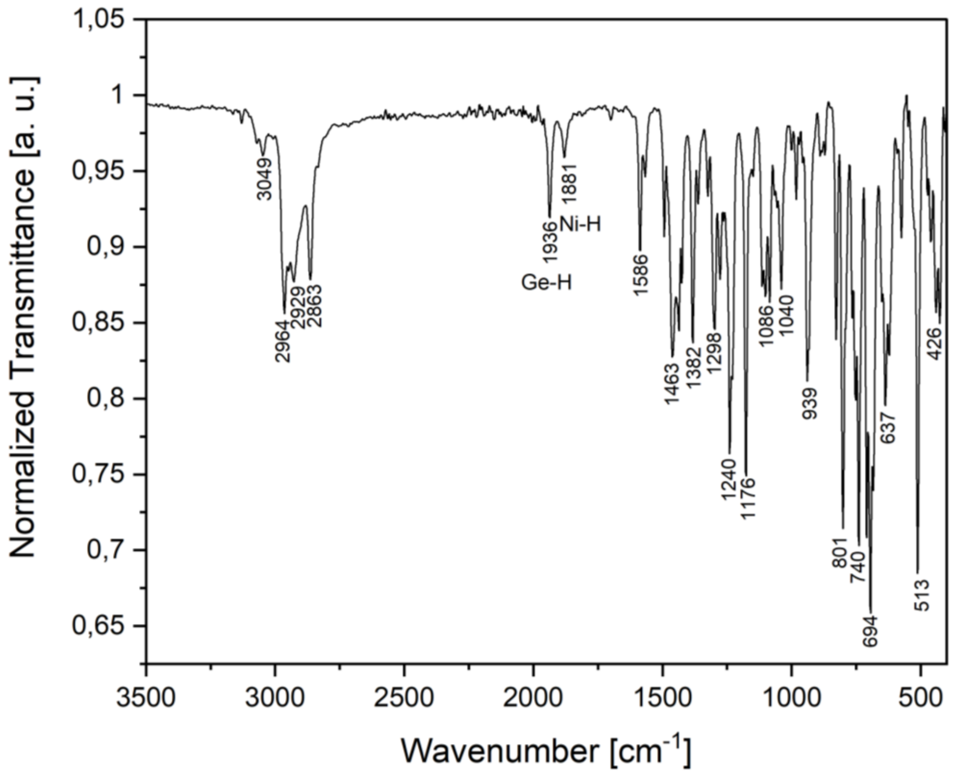

Figure S97. ATR-IR spectrum of solid crystalline **4e** at ambient temperature.

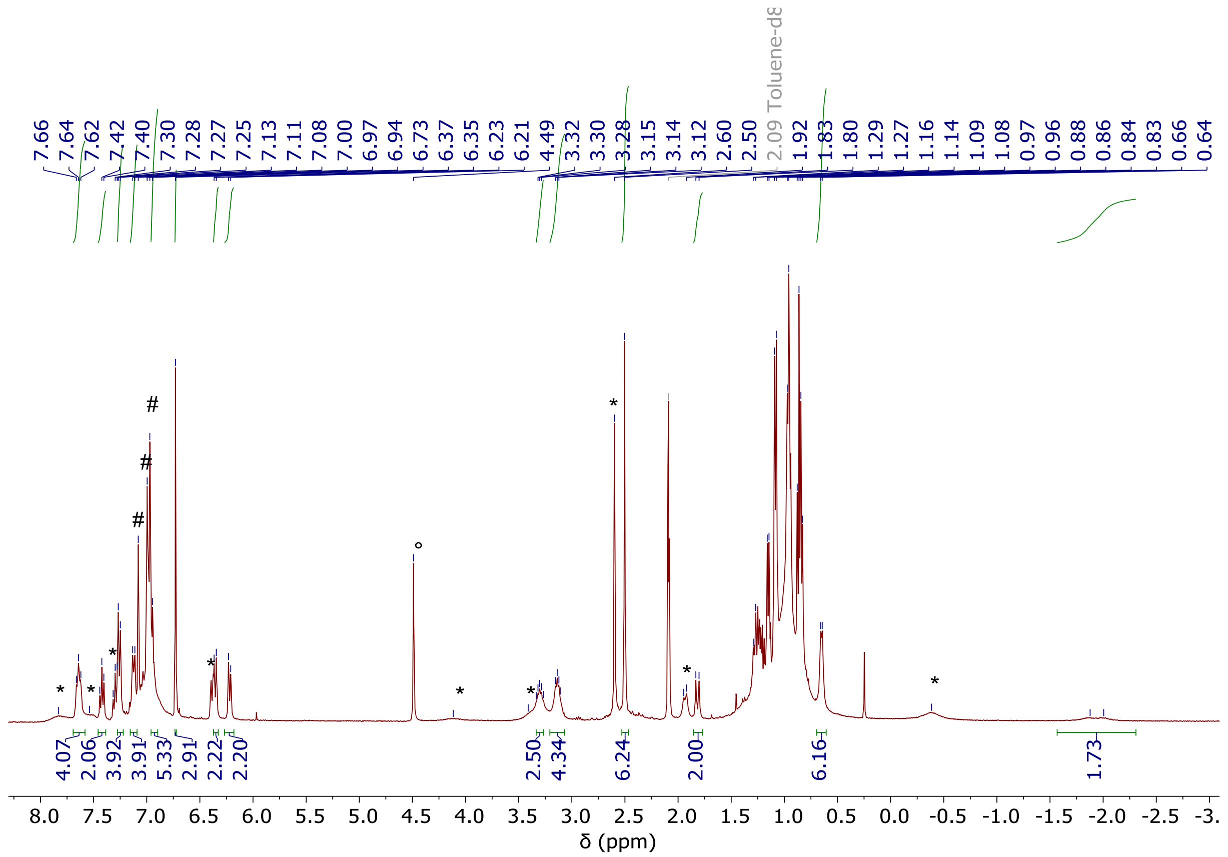

Figure S98. ^{1}H NMR spectrum of **3f** as a solution in toluene-d$_8$ at 60°C under 1 atm H_2 exhibiting an equilibrium with **4f**; *indicates **3f**; °indicates H_2; indicates toluene-d$_8$.

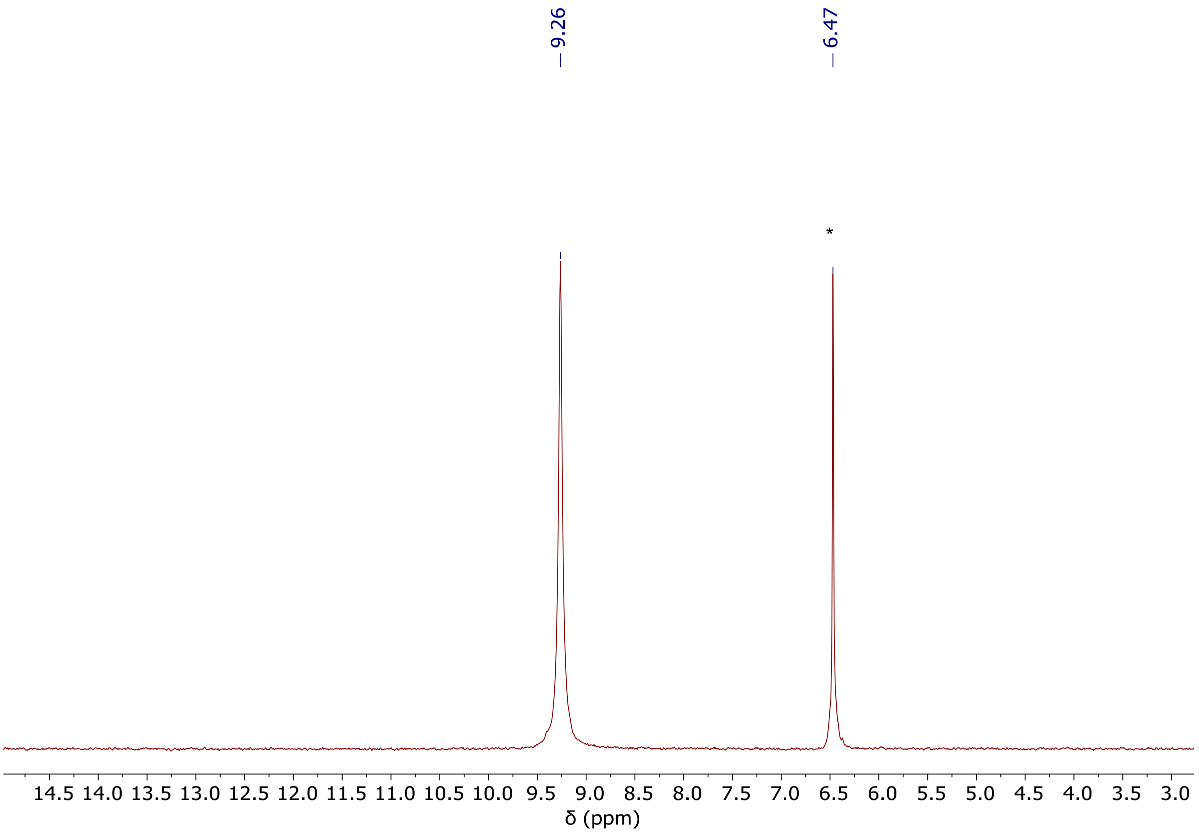

Figure S99. $^{31}P\{^1H\}$ NMR spectrum of **3f** as a solution in C_6D_6 at ambient temperature under 1 atm H_2 exhibiting an equilibrium with **4f**; *indicates **3f**.

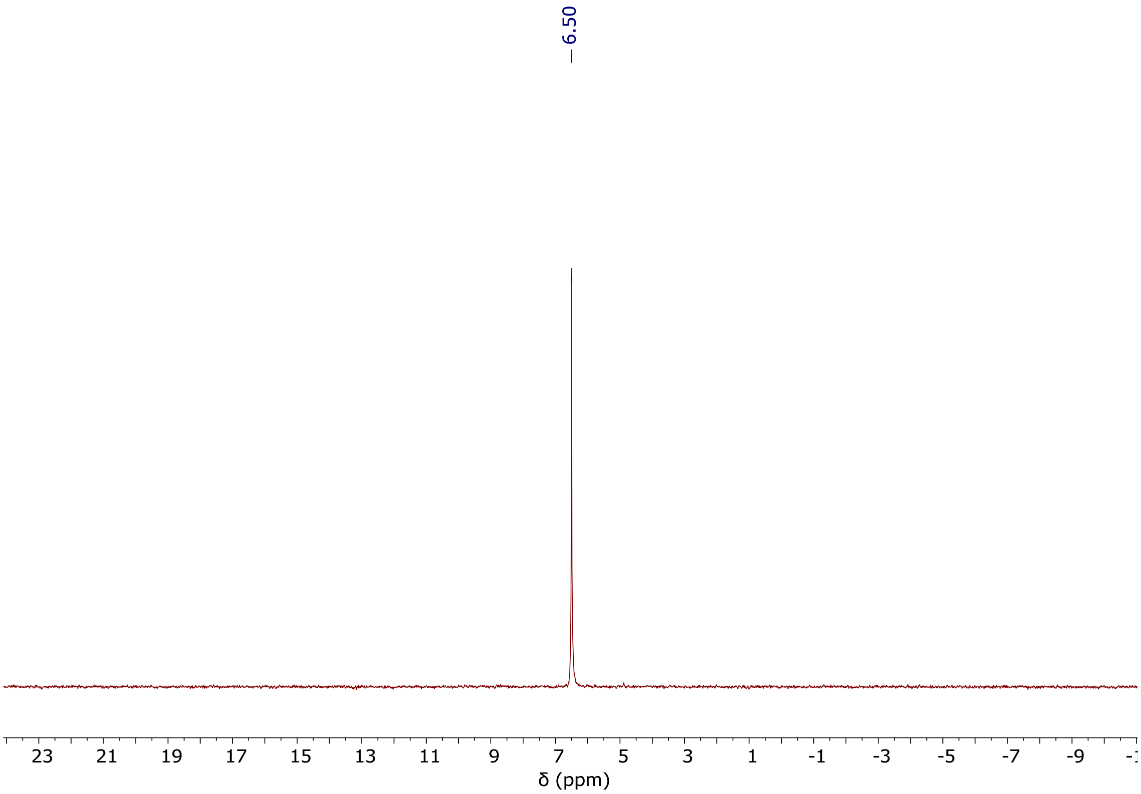

Figure S100. $^{31}P\{^1H\}$ NMR spectrum after removing all volatiles of the reaction of **3f** with H_2 *in vacuo* and redissolving the residue in C_6D_6 showing full regeneration of **3f**.

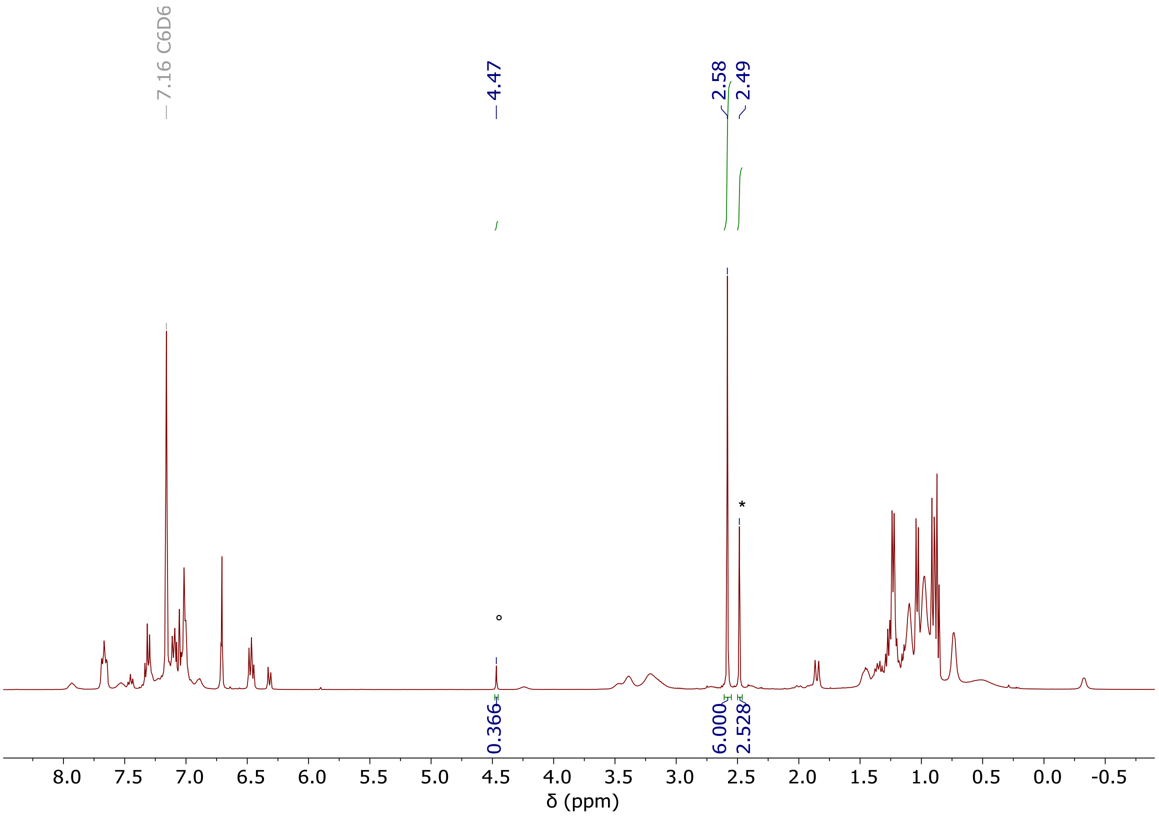

Figure S101. ^{1}H NMR spectrum of 27 mg of **3f** under 1 atm H_2 as a solution in 0.45 mL C_6D_6 at 26°C exhibiting an equilibrium with **4f**; *indicates **3f**; °indicates H_2.

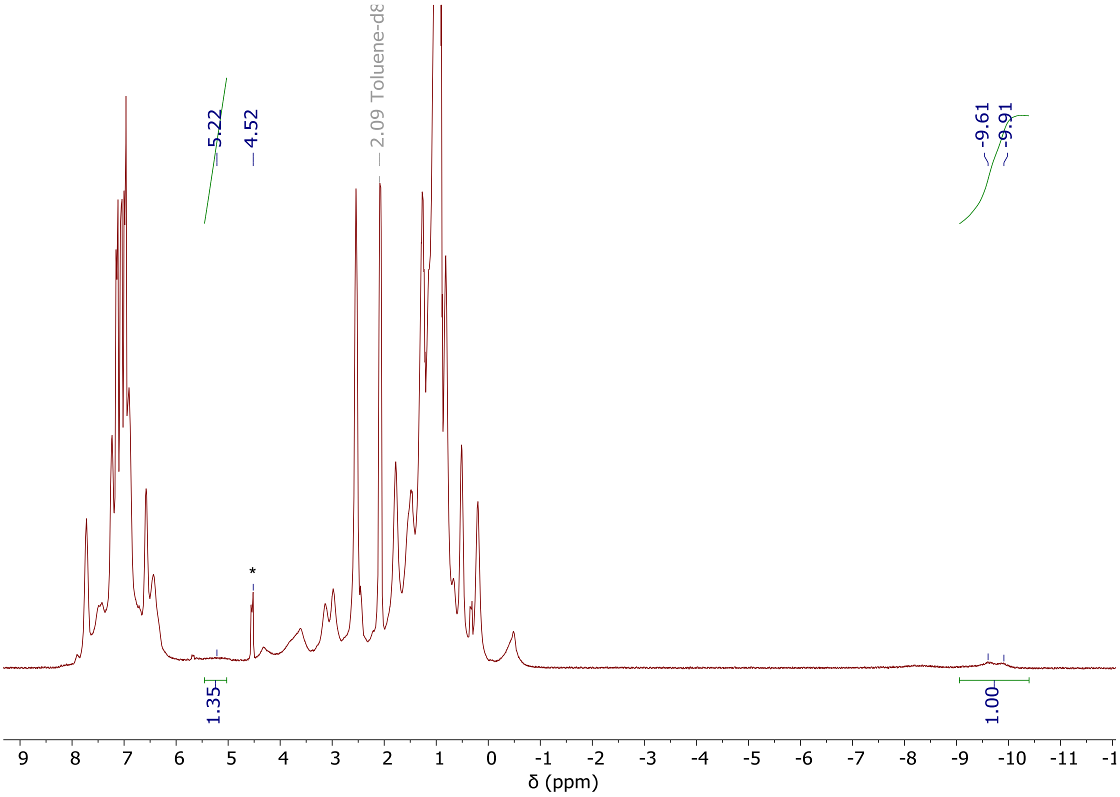

Figure S102. ^{1}H NMR spectrum of **3f** as a solution in toluene-d_8 at -80°C under 1 atm H_2 showing the Ge-*H* and Ni-*H* hydride peaks. °indicates H_2.

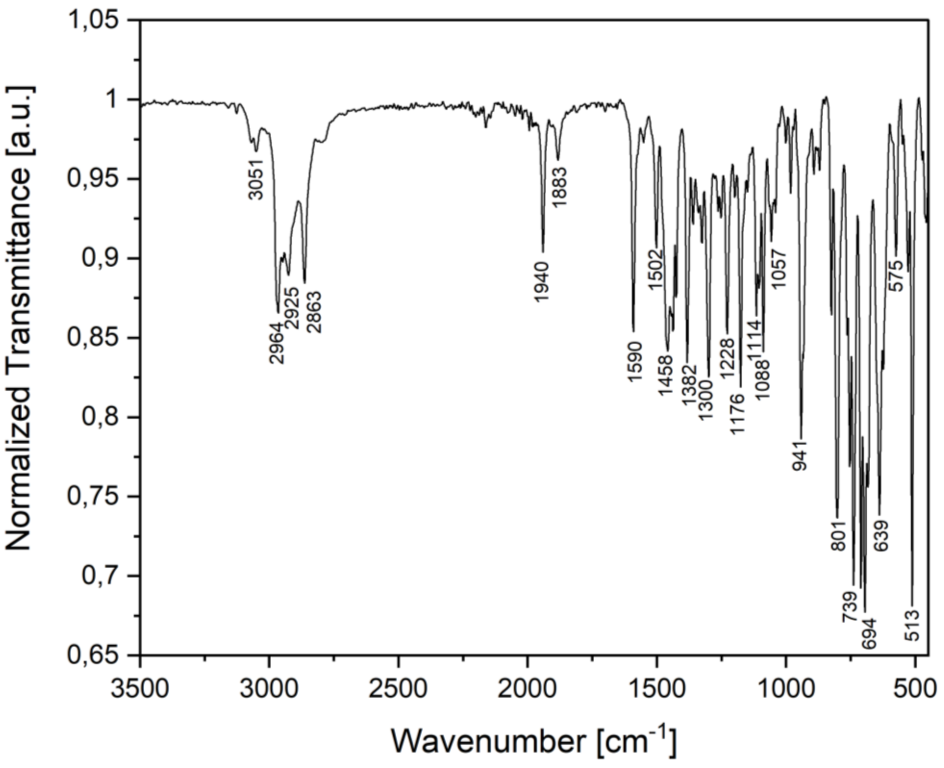

Figure S103. ATR-IR spectrum of solid crystalline **4f** at ambient temperature.

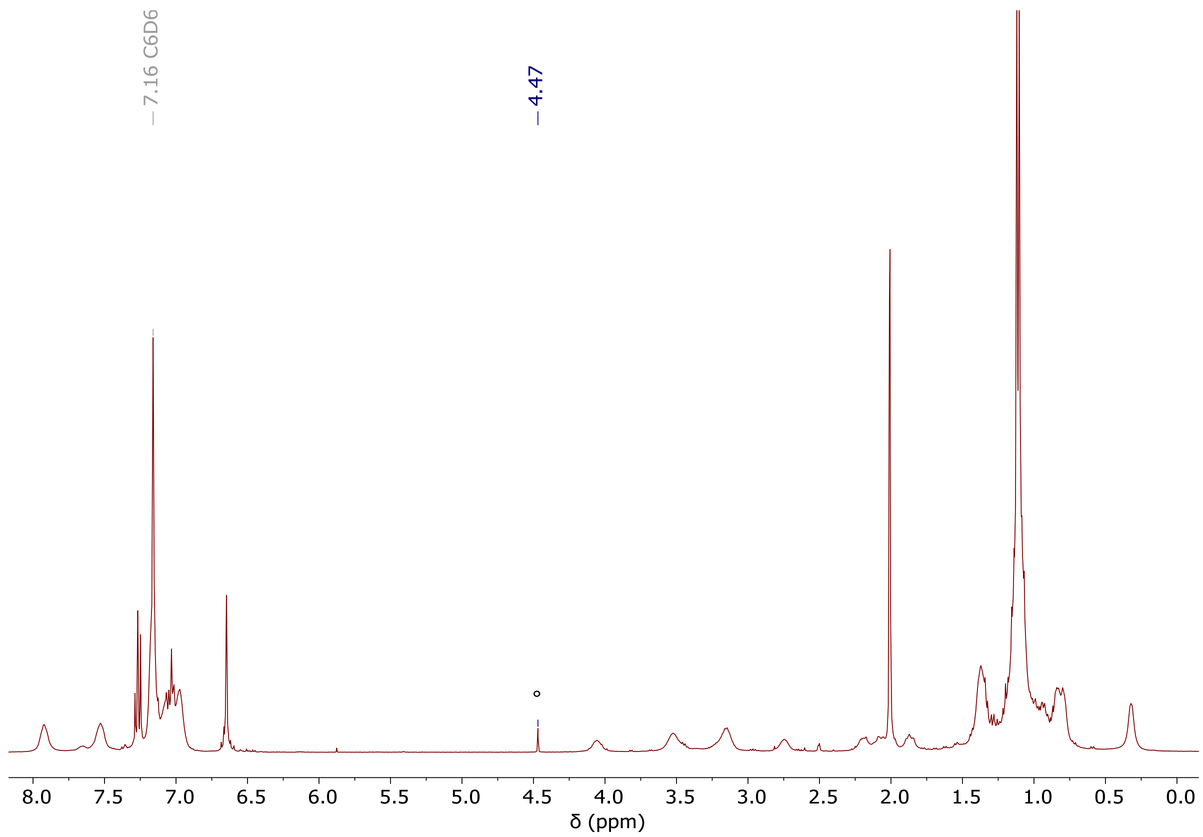

Figure S104. ^{1}H NMR spectrum of **3g** as a solution in C_6D_6 at 60°C under 1 atm H_2 showing no reaction; °indicates H_2.

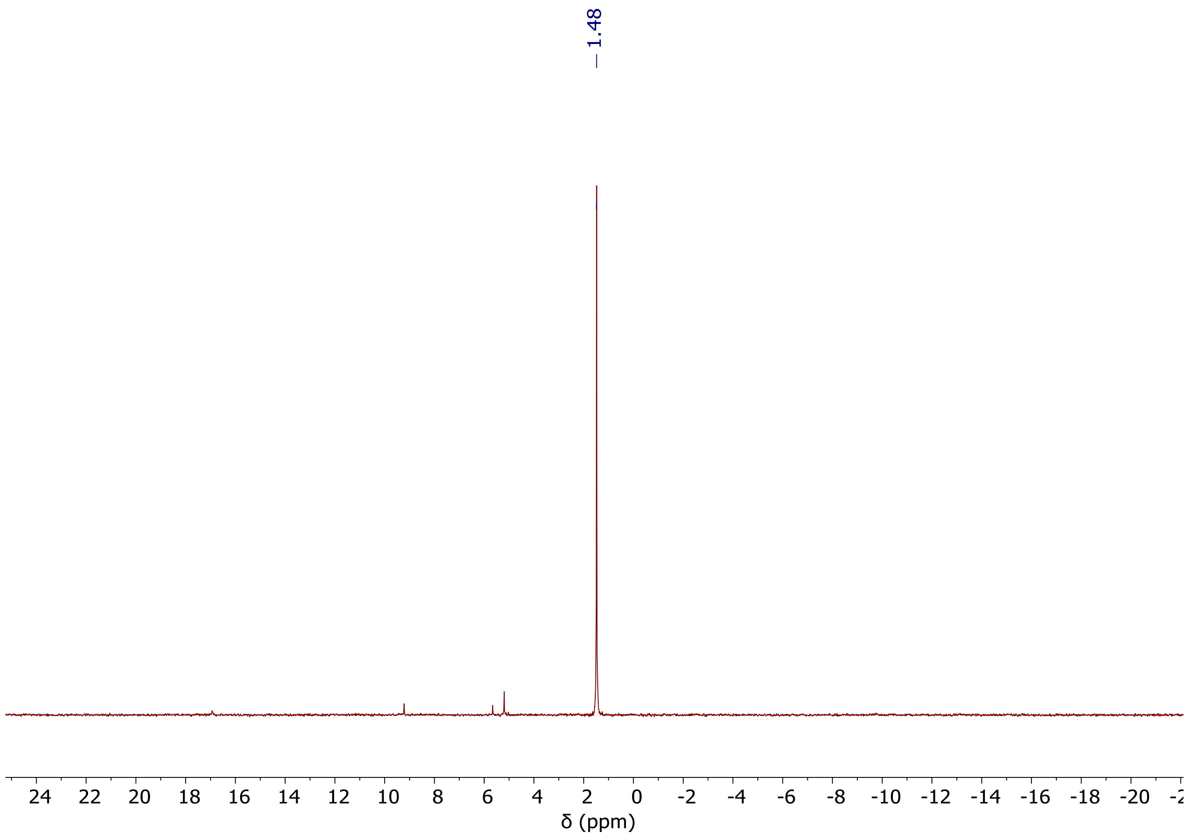

Figure S105. $^{31}P\{^{1}H\}$ NMR spectrum of **3g** as a solution in C_6D_6 at 60°C under 1 atm H_2 showing no reaction; °indicates H_2.

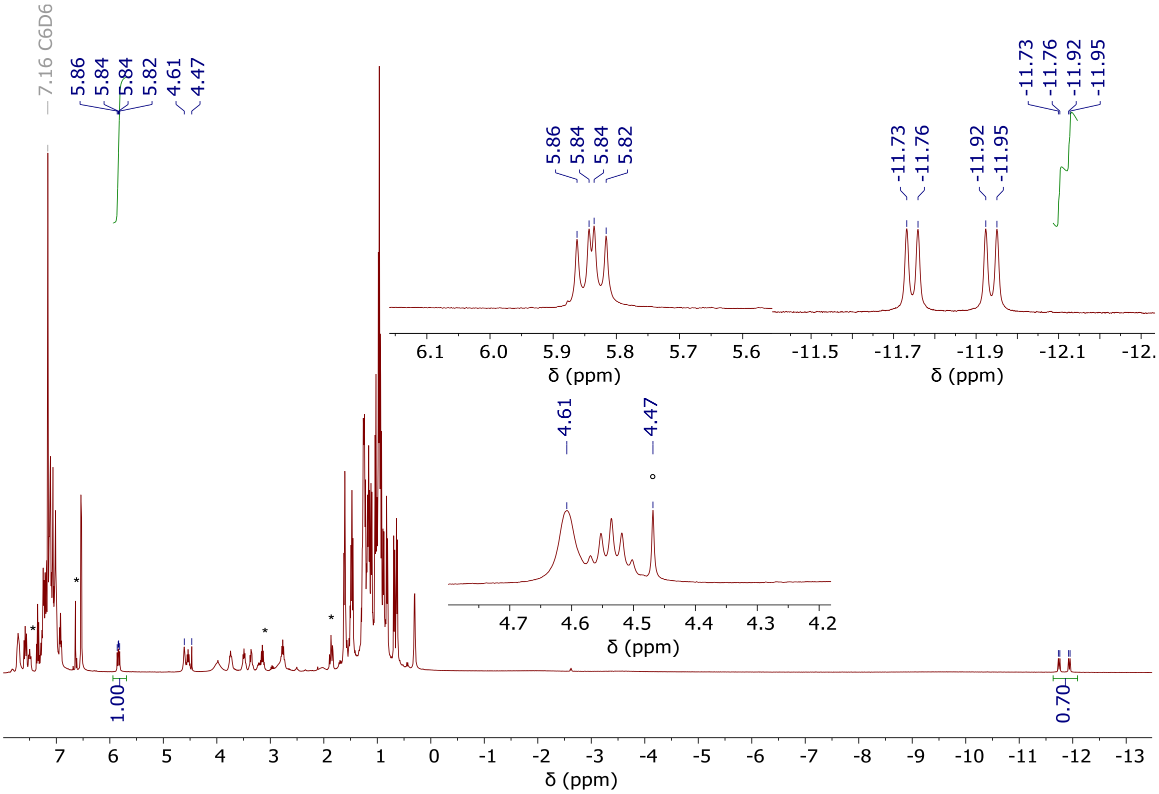

Figure S106. ^{1}H NMR spectrum of [^{PhiP}Dipp(Cl)Ge·Ni·^{Dipp}NHC] as a solution in C_6D_6 at ambient temperature under 1 atm H_2 exhibiting an equilibrium with two species which we tentatively assign as [{^{PhiP}Dipp(H)(Cl)GeNi(H)}·^{Dipp}NHC] (δ = -11.8 and 5.8 ppm) and [{^{PhiP}Dipp(H)$_2$GeNi(Cl)}·^{Dipp}NHC] (δ = 4.6 ppm); *indicating presence of [^{PhiP}Dipp(Cl)Ge·Ni·^{Dipp}NHC]; °indicating presence of H_2.

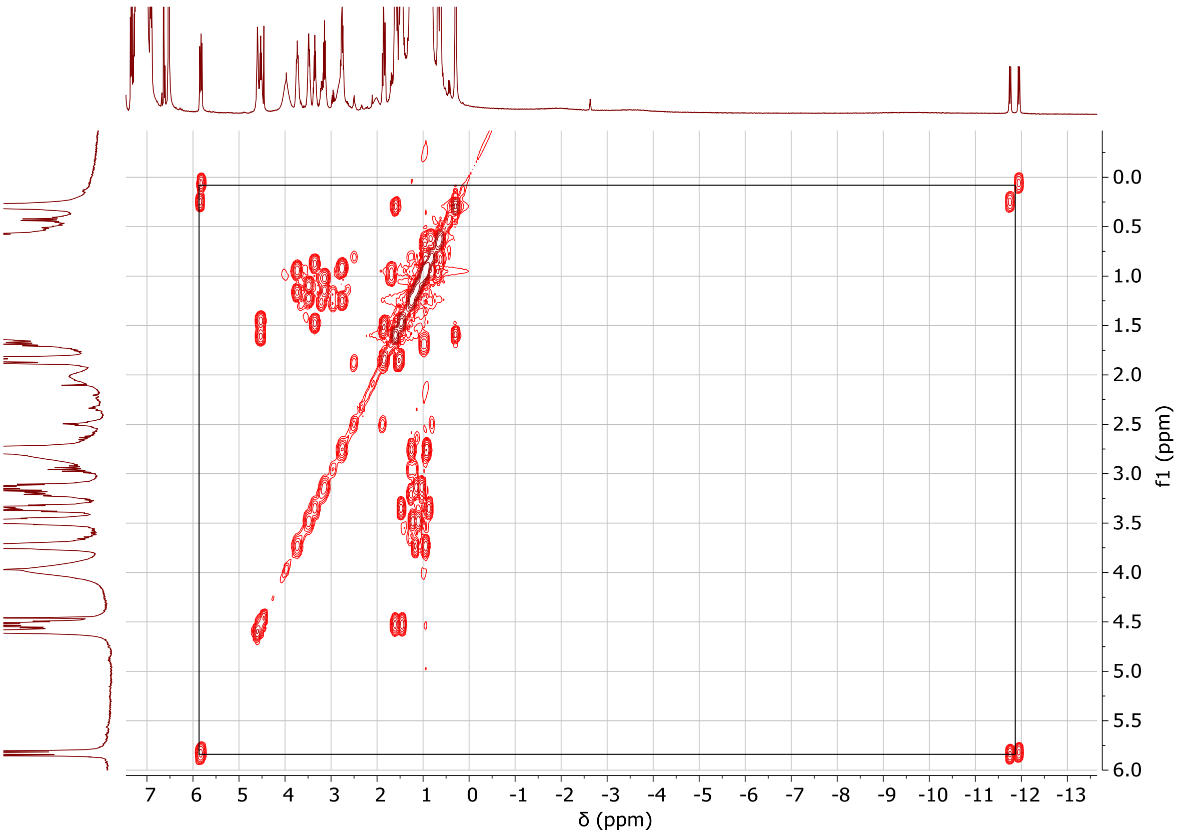

Figure S107. 2D H-H COSY NMR spectrum of the reaction of [^{PhiP}Dipp(Cl)Ge·Ni·^{Dipp}NHC] under 1 atm H_2 as a solution in C_6D_6 at ambient temperature showing the correlation of the assumed Ge-H and Ni-H hydride peaks.

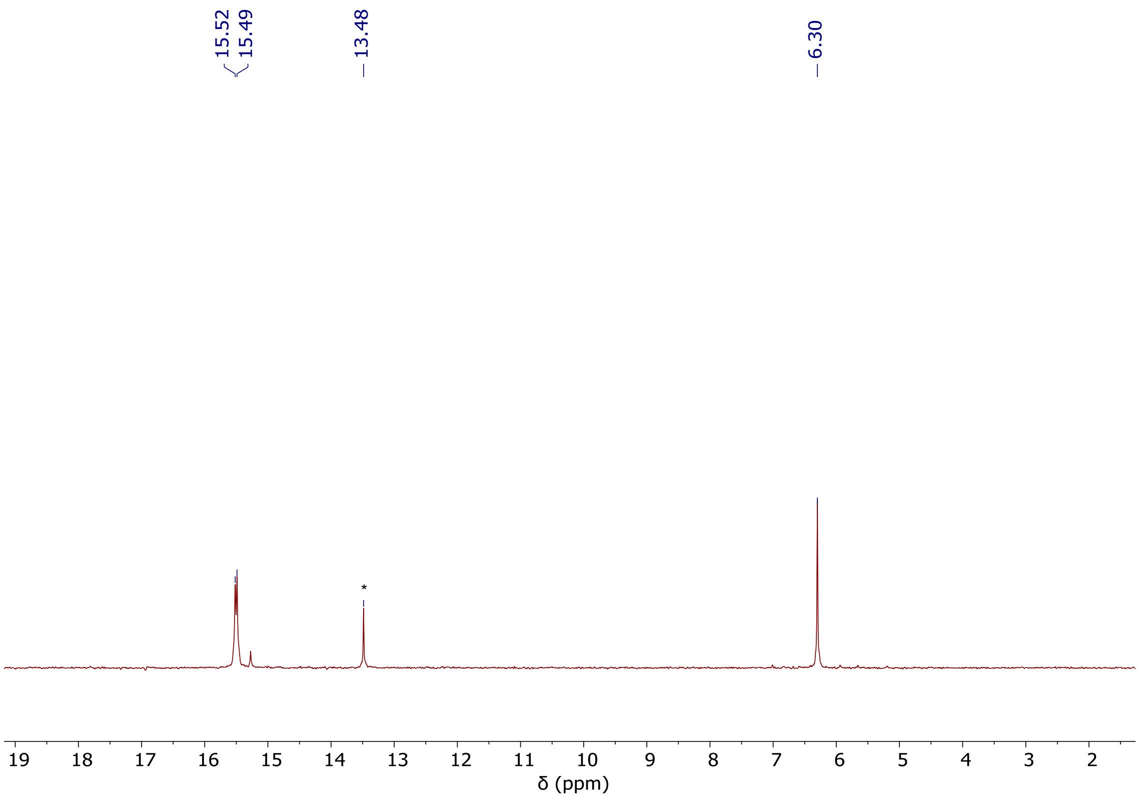

Figure S108. $^{31}P\{^1H\}$ NMR spectrum of [^{PhiP}Dipp(Cl)Ge·Ni·^{Dipp}NHC] as a solution in C_6D_6 at 60°C under 1 atm H_2 exhibiting an equilibrium with two unknown species; *indicating presence of [^{PhiP}Dipp(Cl)Ge·Ni·^{Dipp}NHC].

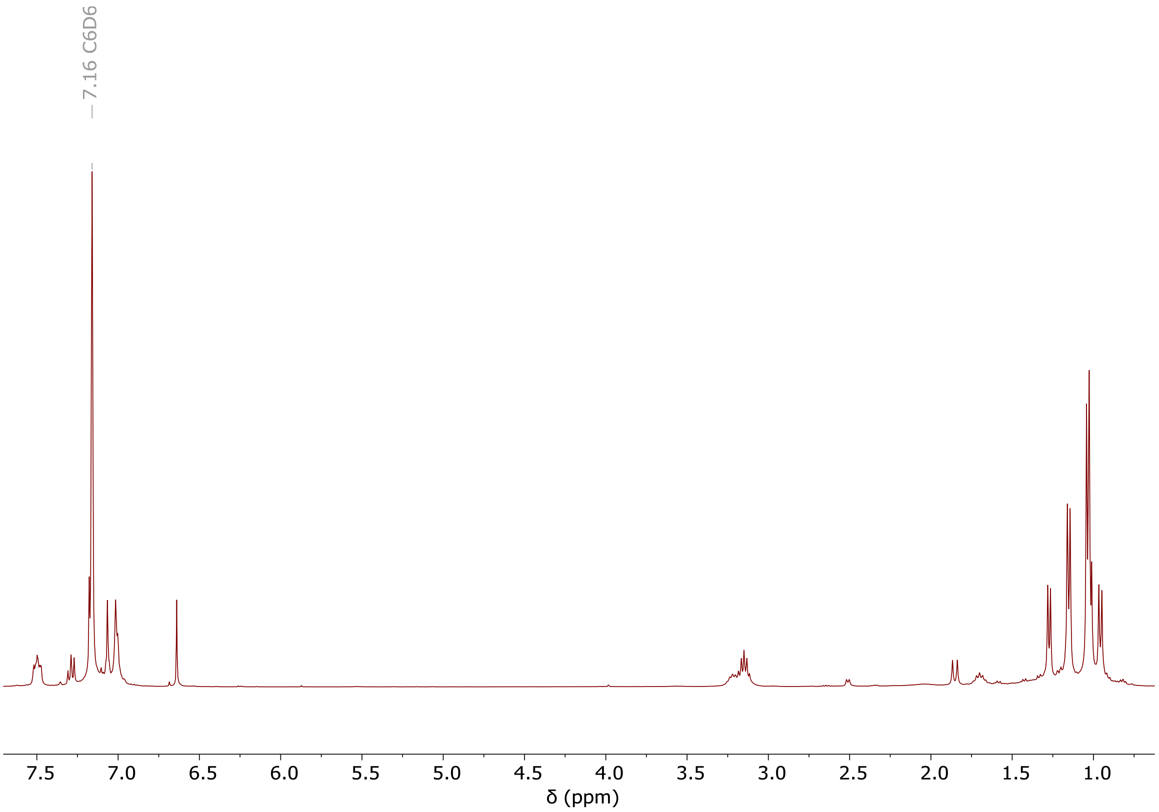

Figure S109. ^{1}H NMR spectrum after removing all volatiles of the reaction of [^{PhiP}Dipp(Cl)Ge·Ni·^{Dipp}NHC] with H_2 *in vacuo* and redissolving the residue in C_6D_6 showing full regeneration of [^{PhiP}Dipp(Cl)Ge·Ni·^{Dipp}NHC].

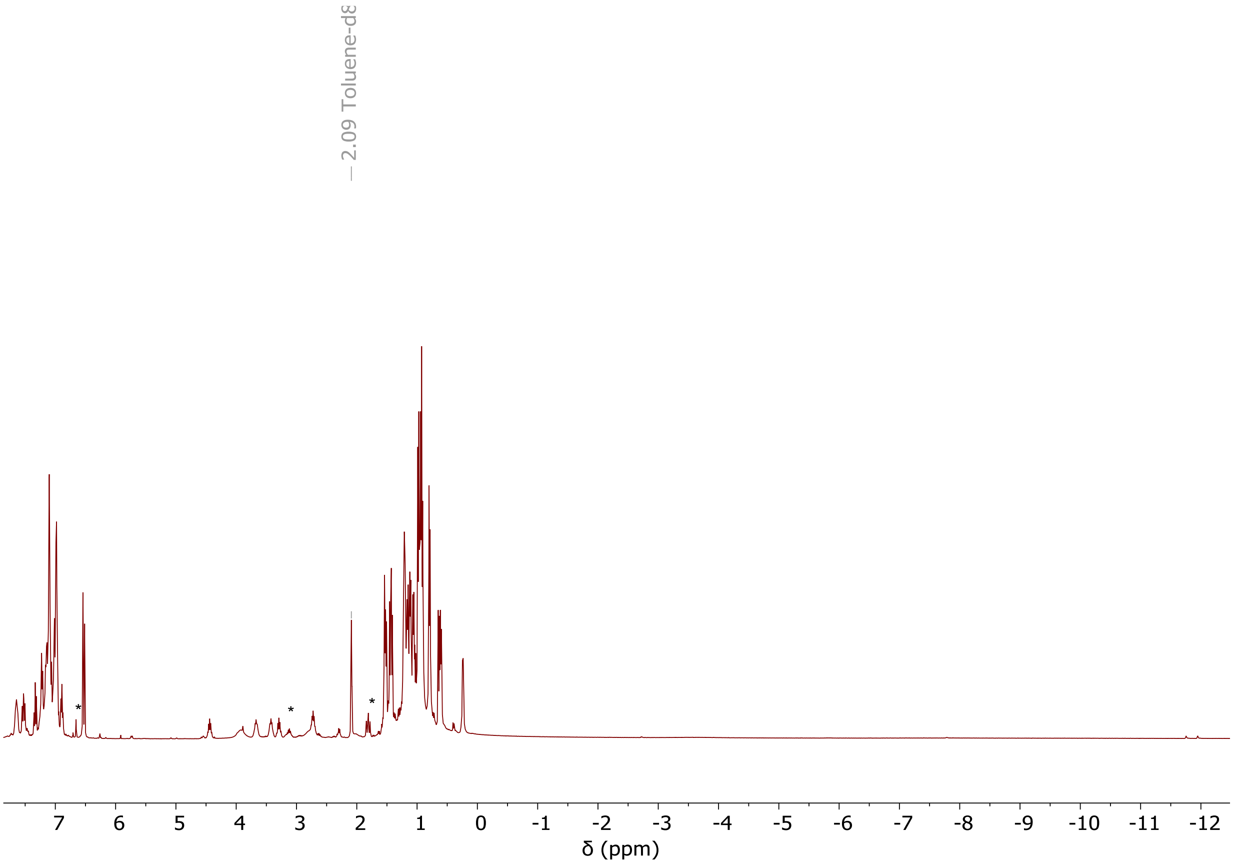

Figure S110. ^{1}H NMR spectrum of [^{PhiP}Dipp(Cl)Ge·Ni·^{Dipp}NHC] as a solution in toluene-d_8 at ambient temperature under 1 atm D_2 exhibiting an equilibrium with two unknown species; * indicating presence of [^{PhiP}Dipp(Cl)Ge·Ni·^{Dipp}NHC].

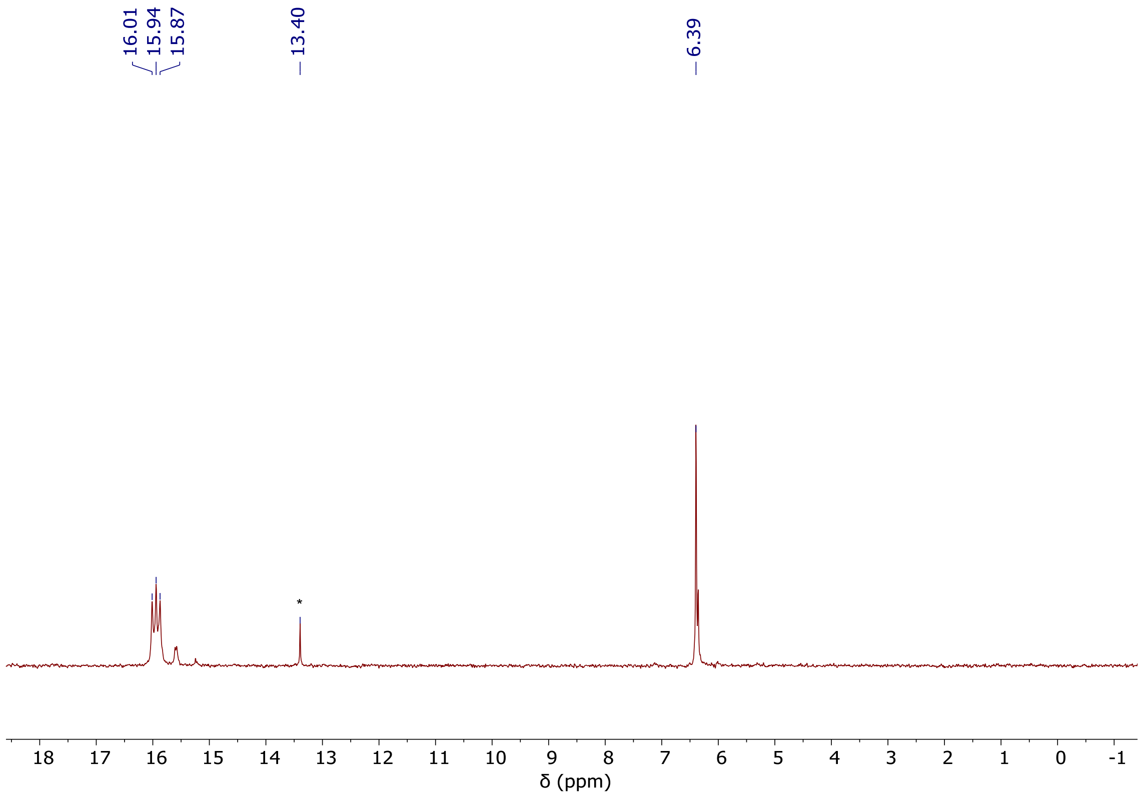

Figure S111. $^{31}P\{^{1}H\}$ NMR spectrum of [^{PhiP}Dipp(Cl)Ge·Ni·^{Dipp}NHC] as a solution in toluene-d_8 at ambient temperature under 1 atm D_2 exhibiting an equilibrium with two unknown species; * indicates presence of [^{PhiP}Dipp(Cl)Ge·Ni·^{Dipp}NHC].

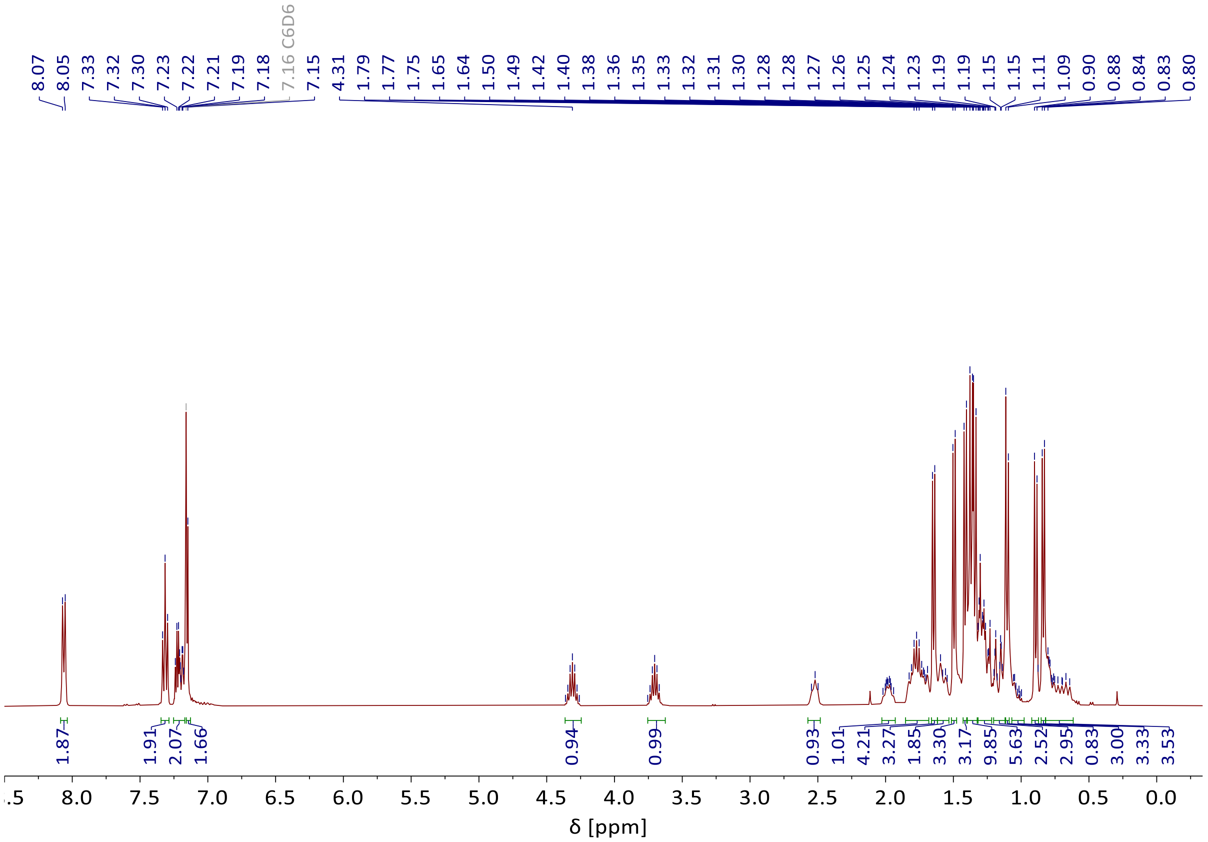

Figure S112. ^{1}H NMR spectrum of **^{CyiP}DippGePh** as a solution in C_6D_6 at ambient temperature.

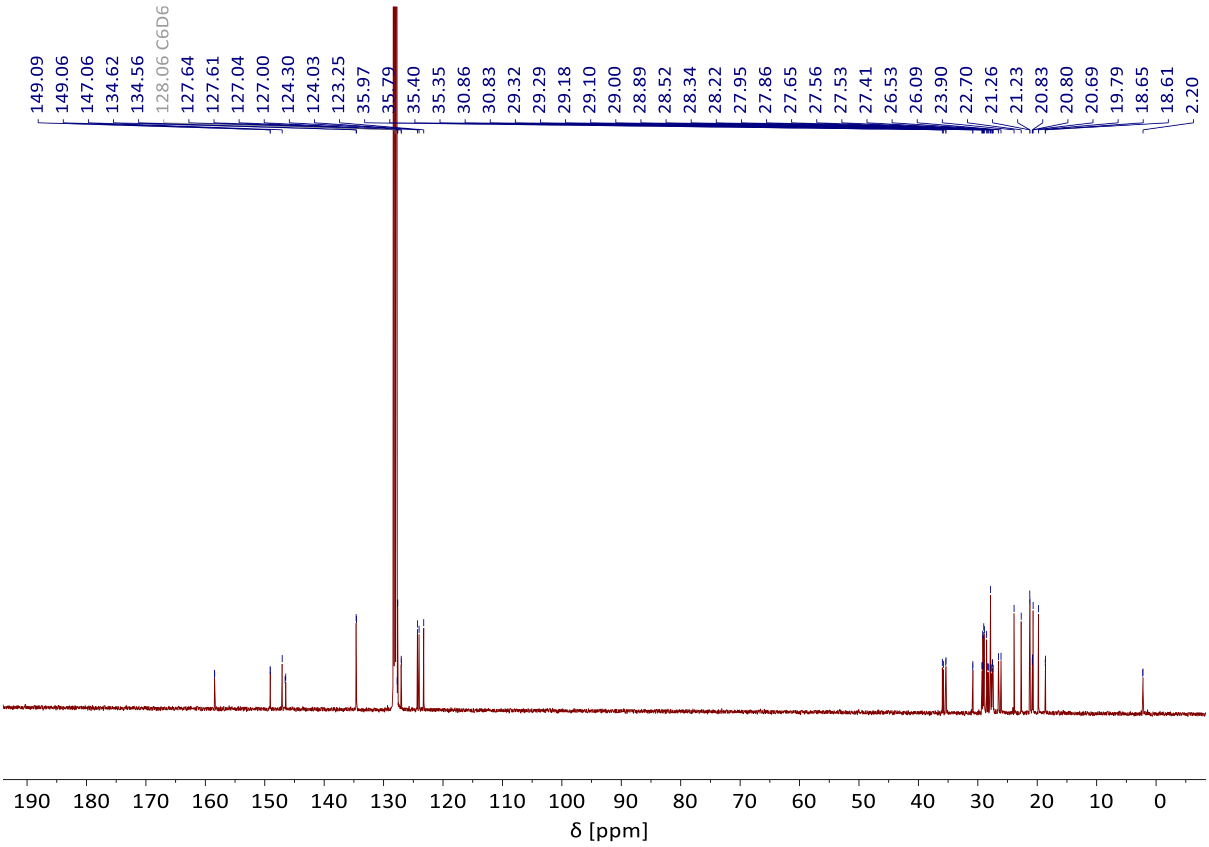

Figure S113. $^{13}C\{^{1}H\}$ NMR spectrum of **^{CyiP}DippGePh** as a solution in C_6D_6 at ambient temperature.

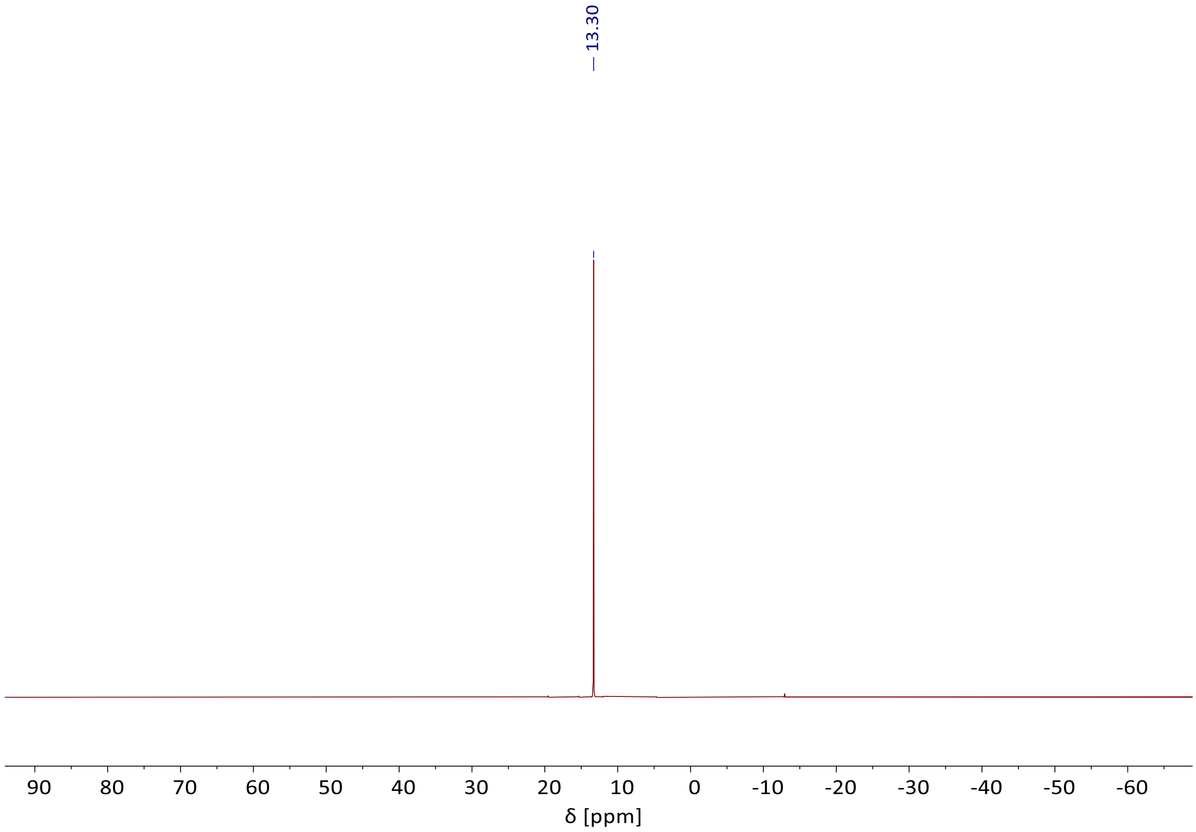

Figure S114. $^{31}P\{^{1}H\}$ NMR spectrum of **^{CyiP}DippGePh** as a solution in C_6D_6 at ambient temperature.

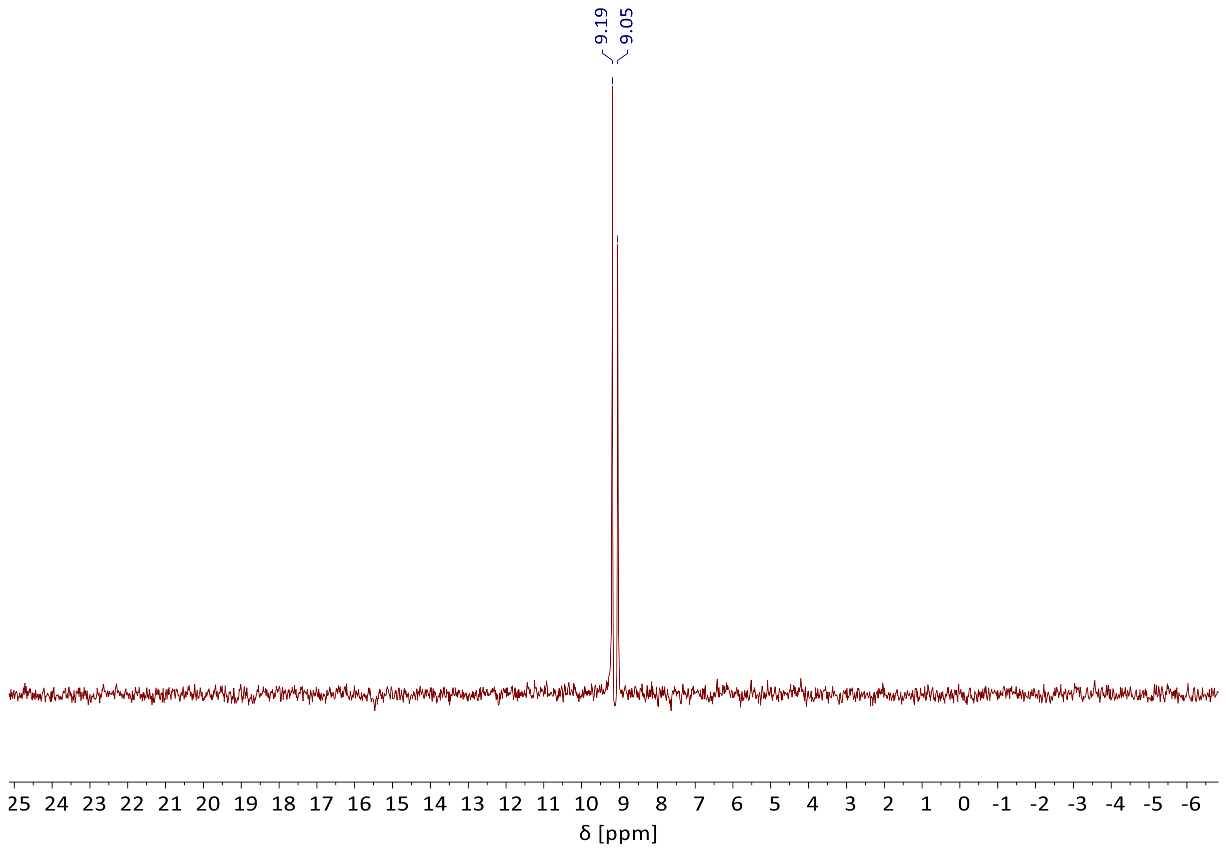

Figure S115. $^{29}Si\{^{1}H\}$ NMR spectrum of **^{CyiP}DippGePh** as a solution in C_6D_6 at ambient temperature.

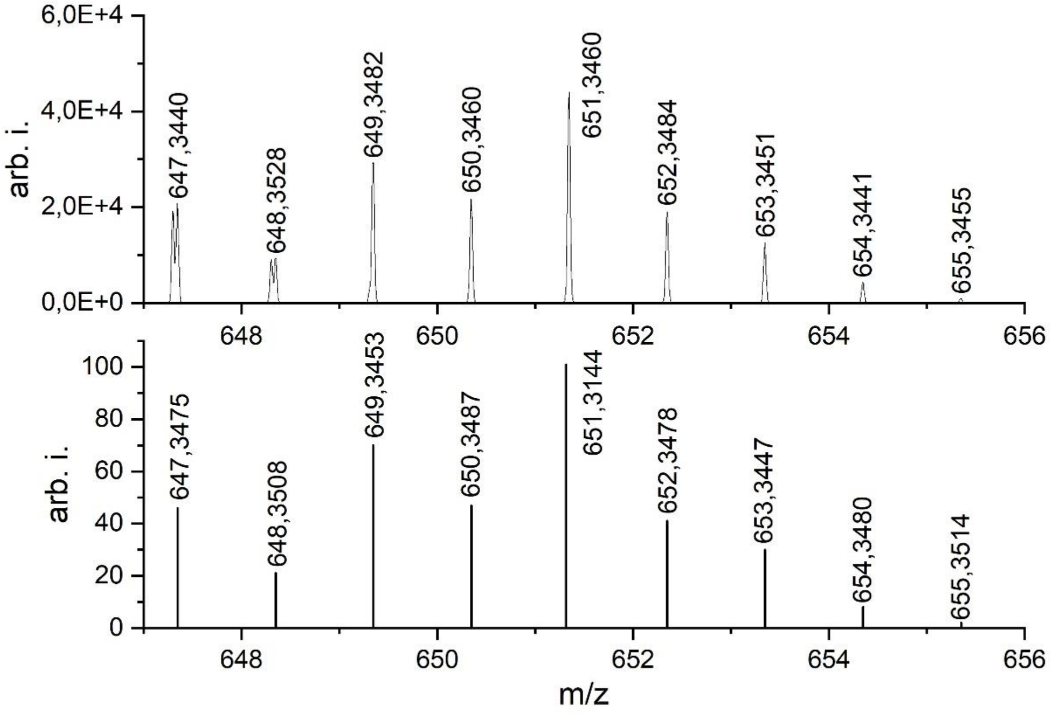

Figure S116. *Top*: Cutout from LIFDI/MS of **^{CyiP}DippGePh**; *Bottom*: Calculated MS spectrum of **^{CyiP}DippGePh**.

Kinetic and Thermodynamic Analyses

Generation of Hammet plot

The equilibrium constant was calculated for the H_2 activation with **4a-f** at 26°C via 1H NMR integrating each a signal of **4a-f** and **5a-f** determining the ratio. The H_2 concentration in solution was calculated by integrating the H_2 signal and taking the absolute concentration of **4a-f** and **5a-f** into account. Log($K_{eq(R)}$/$K_{eq\mathbf{(Ph)}}$) was plotted against the respective Hammet parameters of the aryl substituents.[9]

Table S1. Details for the Hammet plot of complexes **3a-f**.

	K**$_{eq}$ **at 299.15 K [Lmol^{-1}]	**Log(K_{eq4}/K_{eq4a})**	***Hammet parameter σ***
3a (4-CN-C_6H_4)	5089	0.461	0.66
3b (3,5 (CF_3)$_2$-C_6H_3)	10307	0.767	0.86
3c (C_6H_5)	1761	0	0
3d (4-Me-C_6H_4)	1504	-0.069	-0.17
3e (4-OMe-C_6H_4)	867	-0.308	-0.27
3f (4-NMe$_2$-C_6H_4)	449	-0.594	-0.83

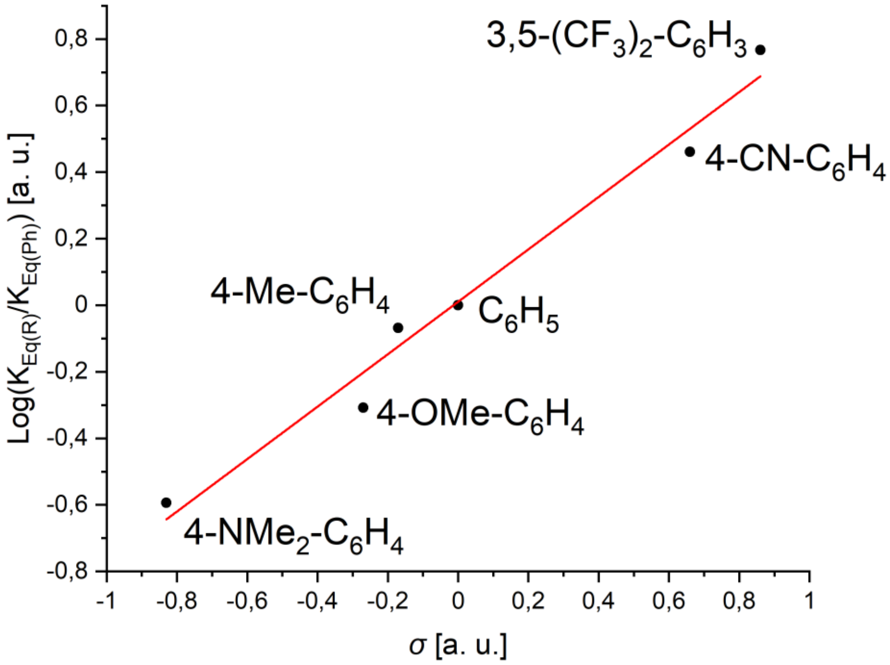

Figure S 117. Hammet plot for the reversible H_2 activation of **3a-f** plotting Log($K_{eq(R)}$/$K_{eq\mathbf{(Ph)}}$) vs the Hammet parameters σ of the residues at the phenyl group.

Thermodynamic assessment of the H_2 activation reaction through Van't Hoff analyses

The equilibrium constant was calculated for the H_2 activation with **3a**-**f** between 20 and 32°C via ^{1}H NMR integrating each a signal of **3a**-**f** and **4a-f** determining the ratio. The H_2 concentration in solution was calculated by integrating the H_2 signal and taking the absolute concentration of **3a-f** and **4a-f** into account. For each compound Ln(K_{eq}) was plotted against T^{-1}. From the slope ΔH_R can be calculated, while ΔS_R can be determined via the x-intercept. The same was also done for the D_2 activation of **3a**. Comparing the K_{eq} values to the H_2 activation could be used to obtain the kinetic isotope effect (KIE).

$$\boldsymbol{K_{eq} = \frac{c(4a-f)}{c(H_2) \times c(3a-f)}}$$

$$\boldsymbol{K_{eq} = e^{-\frac{\Delta G}{RT}}}$$

$$\boldsymbol{ln(K_{eq}) = -\frac{\Delta G}{RT}}$$

$$\boldsymbol{\Delta G = \Delta H - \Delta S \times T}$$

$$\boldsymbol{\ln(K_{eq}) = -\frac{\Delta H}{R} \times \frac{1}{T} + \frac{\Delta S}{R}}$$

$$\boldsymbol{\Delta H = -Slope \times R;\ \Delta S = xIntercept \times R}$$

For 3c, including determination of KIE:

Table S2. Values derived from an Van't Hoff analysis of the equilibrium formed on addition of H_2 to **3c** [a]

T [K]	T^{-1}	ln(K_{eq})
295.15	0.00339	7.697
297.15	0.00337	7.591
299.15	0.00334	7.474
301.15	0.00332	7.371
303.15	0.00330	7.243
305.15	0.00328	7.203

- Linear fitting resulting in: y = 4661.57(±240.10)-8.10(±0.80); R^2 = 0.987

- ΔH_R = -38.76±2.00 kJmol^{-1}; ΔS_R = -67.37±6.65 Jmol^{-1}K^{-1}

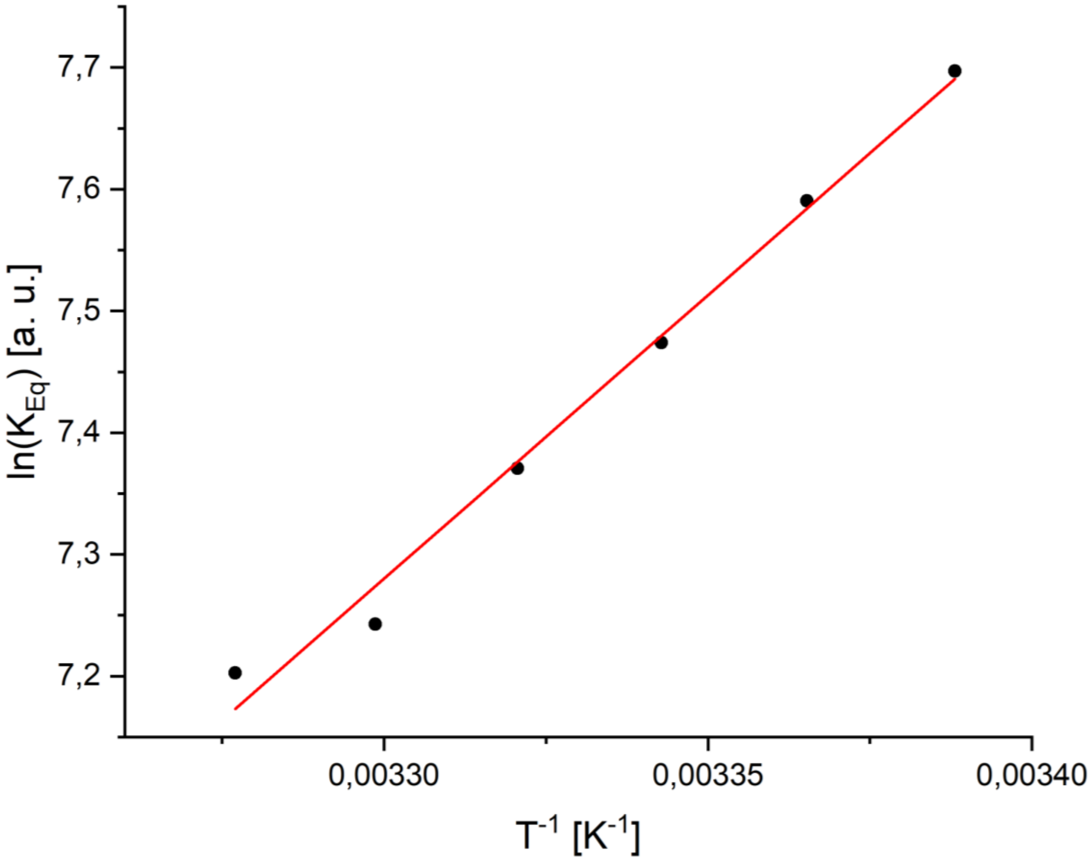

Figure S118. Plot of ln(K_{eq}) vs T^{-1} for **3c** with H_2.

Table S3. Values derived from an Van't Hoff analysis of the equilibrium formed on addition of D_2 to **3c.**

T [K]	T^{-1}	ln(K_{eq})
295.15	0.00339	7.910
297.15	0.00337	7.761
299.15	0.00334	7.663
301.15	0.00332	7.562
303.15	0.00330	7.454
305.15	0.00328	7.370

- Linear fitting resulting in: y = 4799.06(±173.72)-8.37(±0.58); R^2 = 0.993
- ΔH_R = -39.90±1.44 kJmol^{-1}; ΔS_R = -69.60±4.81 Jmol^{-1}K^{-1}

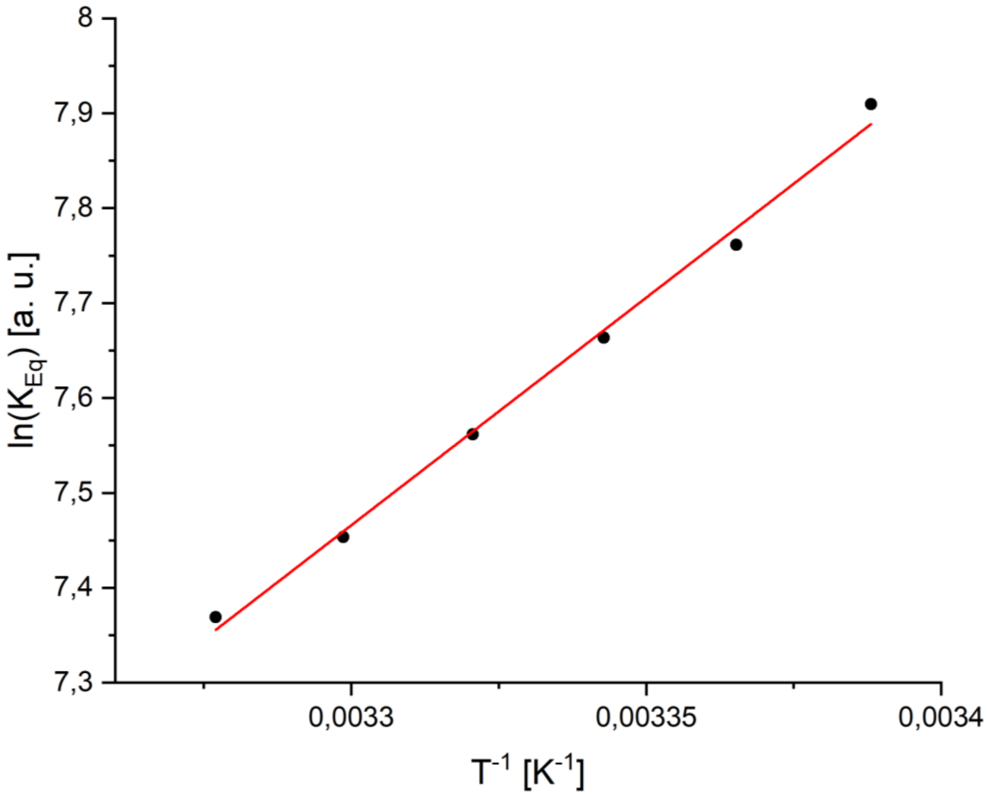

Figure S119. Plot of ln(K_{eq}) vs T^{-1} for **3c** with D_2.

Table S4. Determination of the Kinetic Isotope Effect (KIE) for H_2/D_2 activation by **3c**.

T [K]	*K_{eq} with H_2*	*K_{eq} with D_2*	*KIE*
295.15	2202	2723	0.81
297.15	1979	2348	0.84
299.15	1761	2129	0.83
301.15	1589	1923	0.83
303.15	1398	1726	0.81
305.15	1343	1586	0.85

Table S5. Values derived from an Van't Hoff analysis of the equilibrium formed on addition of D_2 to **3a.**

T [K]	*T^{-1}*	ln(K_{eq})
295.15	0.00339	8.754
297.15	0.00337	8.632
299.15	0.00334	8.535
301.15	0.00332	8.382
303.15	0.00330	8.285
305.15	0.00328	8.163

- Linear fitting resulting in: y = 5339.12(±136.85)-9.33(±0.46); R^2 = 0.997
- ΔH_R = -44.39±1.14 kJmol^{-1}; ΔS_R = -77.59±3.79 Jmol^{-1}K^{-1}

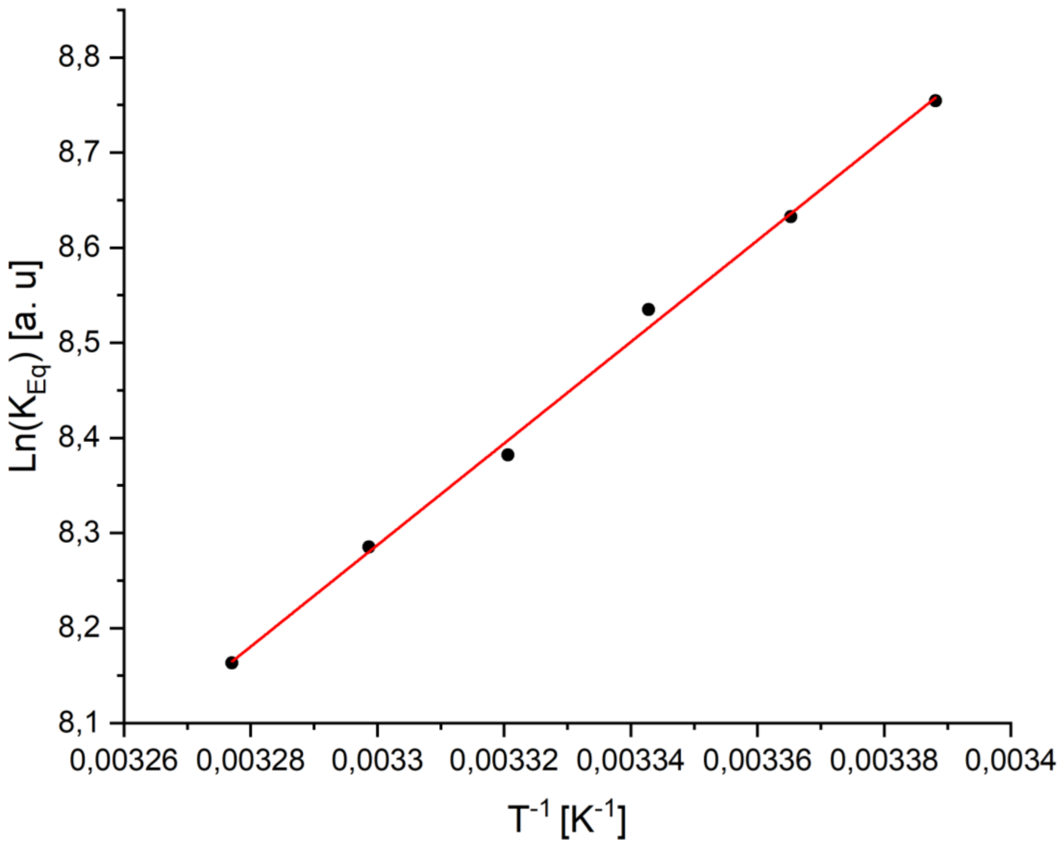

Figure S120. Plot of ln(K_{eq}) vs T^{-1} for **3a** with H_2.

Table S6. Values derived from an Van't Hoff analysis of the equilibrium formed on addition of D_2 to **3b.**

T [K]	T^{-1}	$\ln(K_{eq})$
295.15	0.00339	9.431
297.15	0.00337	9.343
299.15	0.00334	9.241
301.15	0.00332	9.104
303.15	0.00330	8.979
305.15	0.00328	8.878

- Linear fitting resulting in: y = 5134.97(±182.60)-7.95(±0.61); $R^2 = 0.994$
- ΔH_R = -42.69±1.52 kJmol^{-1}; ΔS_R = -66.08±5.06 Jmol^{-1}K^{-1}

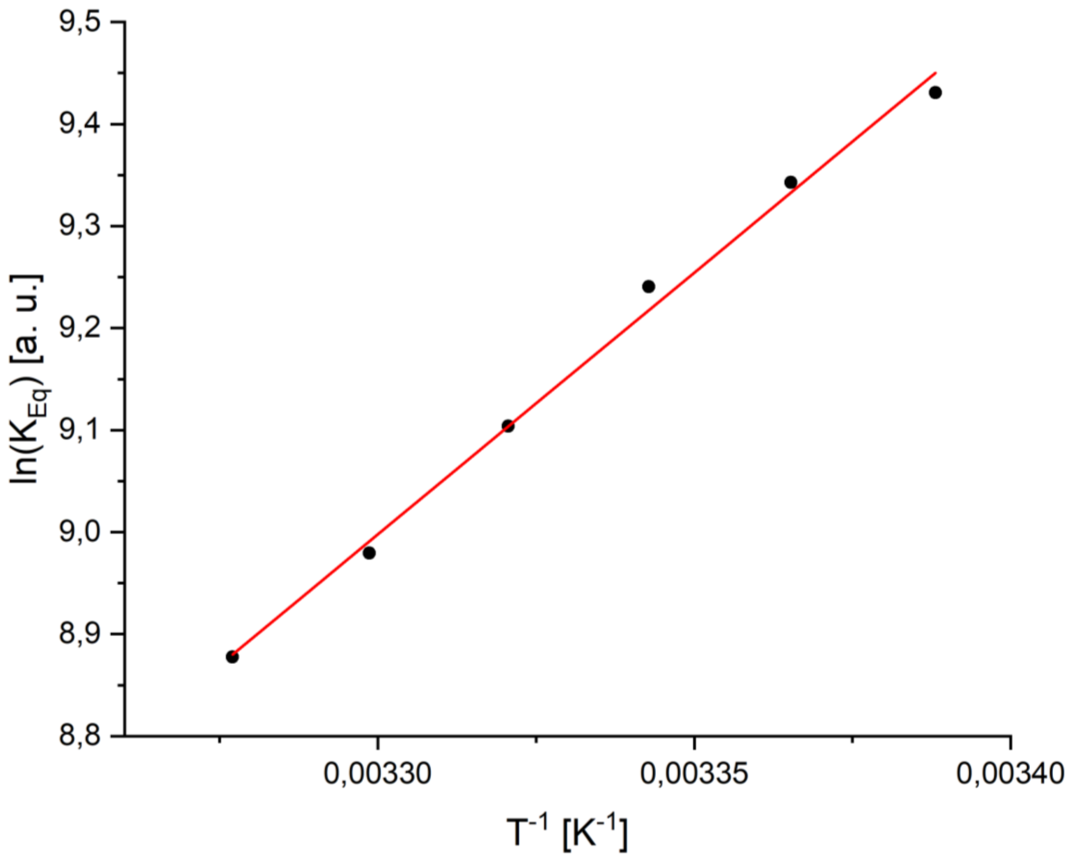

Figure S121. Plot of $\ln(K_{eq})$ vs T^{-1} for **3b** with H_2.

Table S7. Values derived from an Van't Hoff analysis of the equilibrium formed on addition of D_2 to **3d.**

T [K]	*T^{-1}*	**ln(K_{eq})**
293.15	0.00341	7.615
295.15	0.00339	7.491
297.15	0.00337	7.400
299.15	0.00334	7.316
301.15	0.00332	7.200
303.15	0.00330	7.098

- Linear fitting resulting in: y = 4492.09(±114.82)-7.72(±0.39); R^2 = 0.997
- ΔH_R = -37.35±0.95 kJmol^{-1}; ΔS_R = -64.15±3.20 Jmol^{-1}K^{-1}

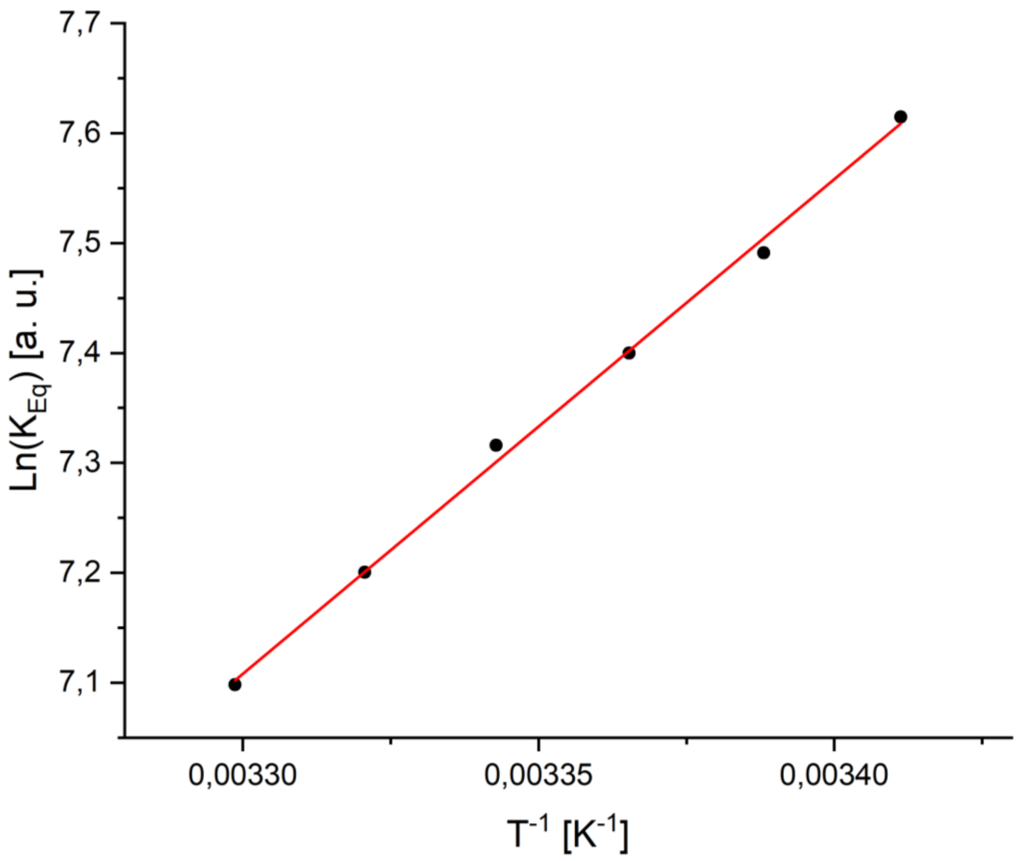

Figure S122. Plot of ln(K_{eq}) vs T^{-1} for **3d** with H_2.

Table S8. Values derived from an Van't Hoff analysis of the equilibrium formed on addition of D_2 to **3e.**

T [K]	T^{-1}	$\ln(K_{eq})$
295.15	0.00339	7.000
297.15	0.00337	6.854
299.15	0.00334	6.765
301.15	0.00332	6.703
303.15	0.00330	6.626
305.15	0.00328	6.500

- Linear fitting resulting in: y = 4180.50(±268.02)-7.19(±0.89); R^2 = 0.980
- ΔH_R = -34.76±2.23 kJmol^{-1}; ΔS_R = -59.76±7.42 Jmol^{-1}K^{-1}

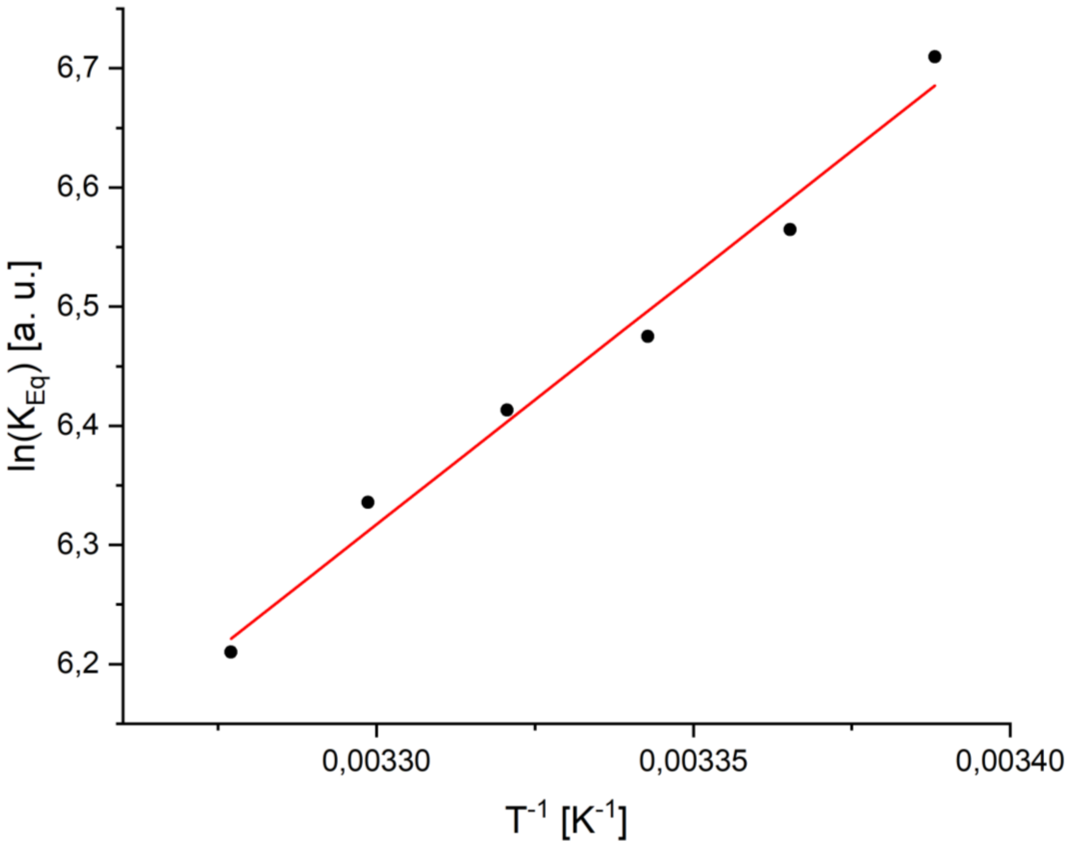

Figure S123. Plot of $\ln(K_{eq})$ vs T^{-1} for **3e** with H_2.

Table S9. Values derived from an Van't Hoff analysis of the equilibrium formed on addition of D_2 to **3f.**

T [K]	T^{-1}	ln(K_{eq})
295.15	0.00339	6.309
297.15	0.00337	6.185
299.15	0.00334	6.108
301.15	0.00332	6.021
303.15	0.00330	5.949
305.15	0.00328	5.849

- Linear fitting resulting in: y = 3980.53(±144.10)-7.19(±0.48); R^2 = 0.993
- ΔH_R = -33.10±1.20 kJmol^{-1}; ΔS_R = -59.81±3.99 Jmol^{-1}K^{-1}

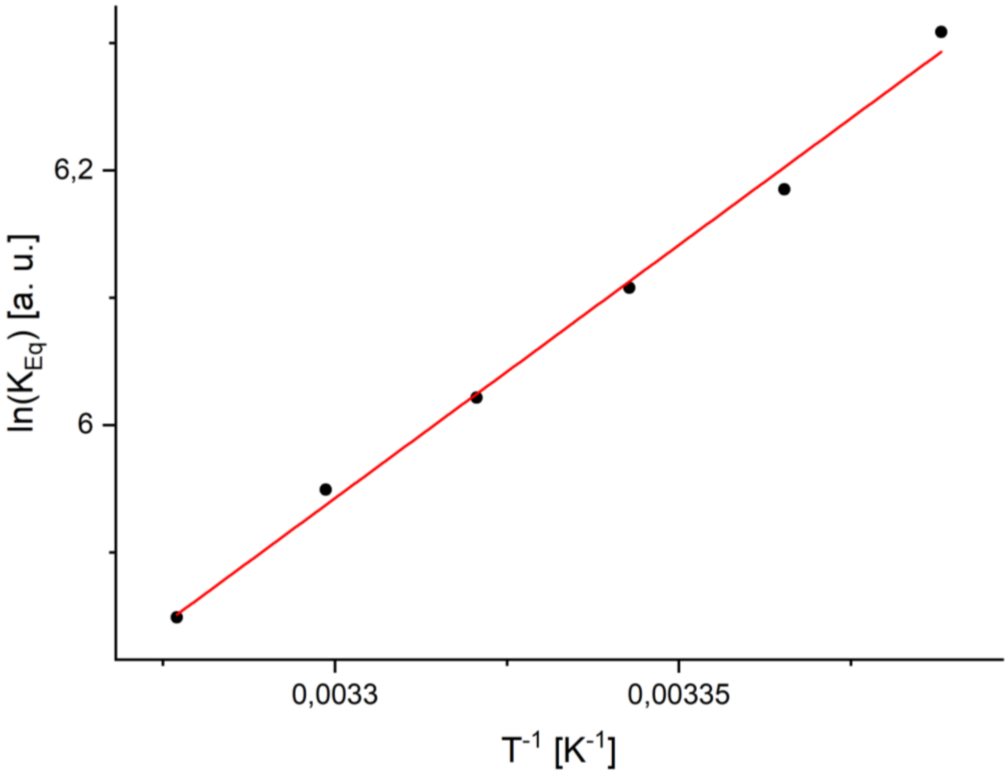

Figure S124. Plot of ln(K_{eq}) vs T^{-1} for **3f** with H_2.

Details of dehydrocoupling catalyses

General Method for catalytic dehydrocoupling of $PhSiH_3$ with [^{PhiP}DippGeX·Ni·^{Dipp}NHC] complexes

0.5 mL of a C_6D_6 with **4a-f** (1.62 mM) was filled in a 15 mL Schlenk flask. Afterwards $PhSiH_3$ (20 µL, 0.16 mM) was added and the flask stirred at 7000 rpm for 3 h while being open to the Schlenk line with a slight overpressure of 0.05 bar. Then mesitylene (11.2 µL, 0.08 mmol) was added as an internal standard. The consumption of $PhSiH_3$ was ascertained by integration of the residual $PhSiH_3$ signal against the internal standard in the ^{1}H NMR spectra of reaction mixtures after the allotted reaction. All measurements were performed in triplicate. This gave the data provided in the main text. Due to the small scale of these reactions, oligomers/polymers were not isolated or further characterized.

General Method for polyphenysilane *via* dehydrocoupling of $PhSiH_3$ with 4a

4a (10 mg, 0.0092 mmol) was dissolved in 1.5 mL C6D6 in a 50 mL Schlenk flask. Subsequently $PhSiH_3$ (45 μL, 0.3687 mmol) was added to the reaction mixture, and the reaction stirred for 48 h while open to an inert gas manifold with a slight overpressure of 0.05 bar Ar. The crude solution was filtered over silica and basic Al_2O_3 with toluene as eluent under air. All volatiles were then removed *in vacuo* leading to a colorless oily solid. This process was repeated in triplicate resulting in a mean isolated yield of 36 mg (92%, 0.34 mmol). These solids were analyzed by GPC, using THF stabilized with 250 ppm BHT as eluent. Absolute molecular weights have been determined by two angle laser light scattering analysis using a concentration and viscosity detector, coupled with GPC, as shown in Figs. S129-131.

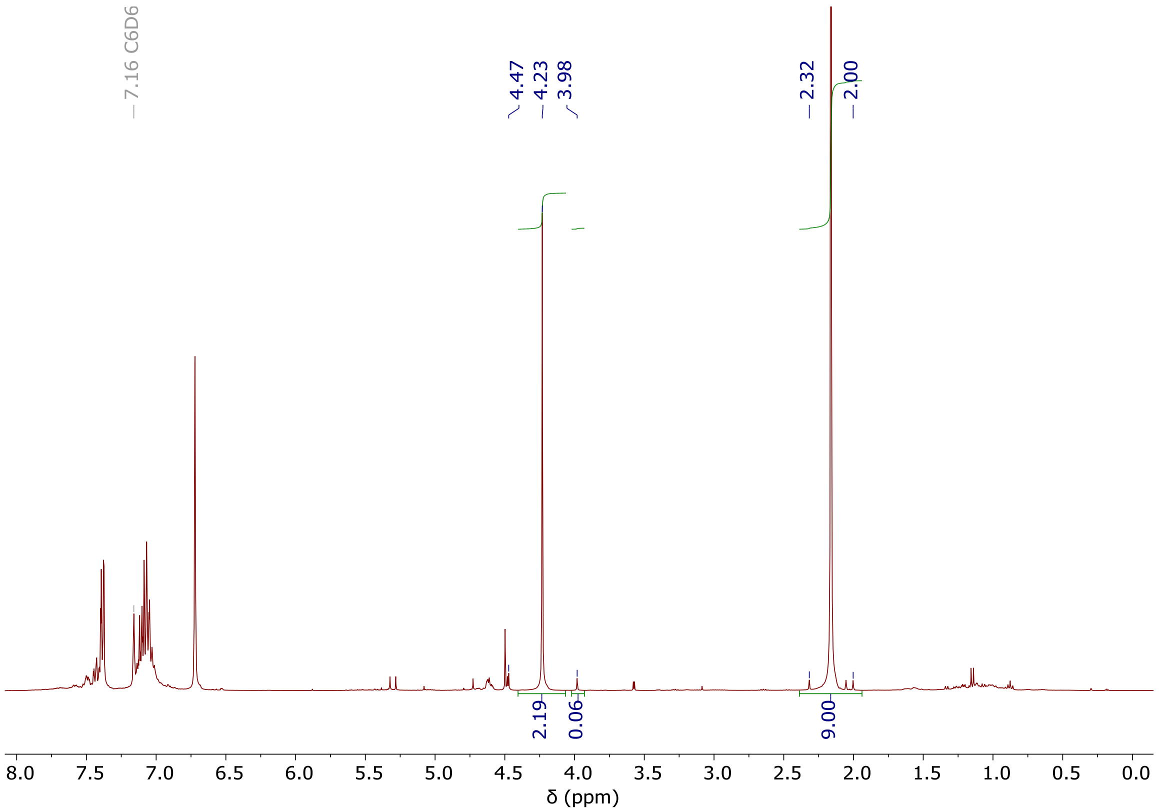

Figure S 125. In-situ ^{1}H NMR of dehydrogenative coupling of $PhSiH_3$ with 1 mol% **4a** after 3 hours with mesitylene as standard to determine consumption.

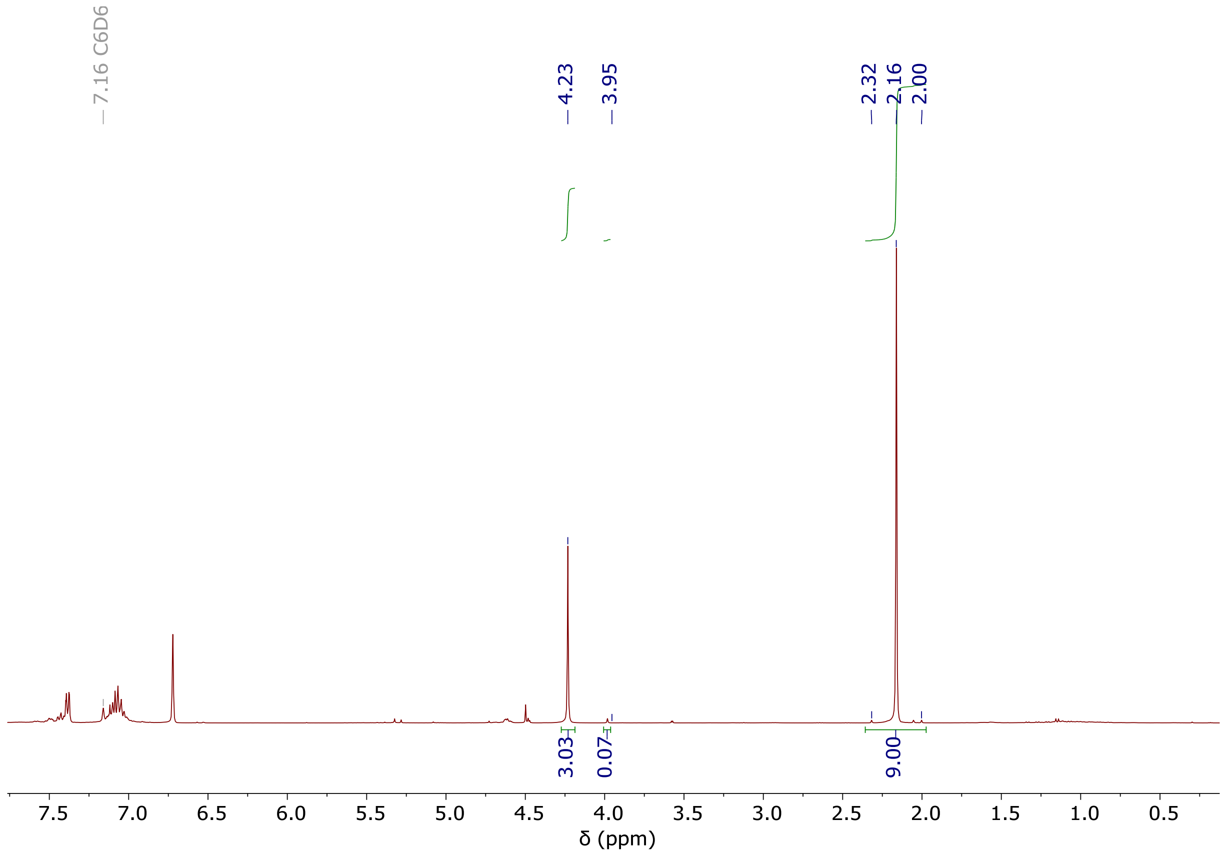

Figure S 126. In-situ ^{1}H NMR of dehydrogenative coupling of $PhSiH_3$ with 1 mol% **4b** after 3 hours with mesitylene as standard to determine consumption.

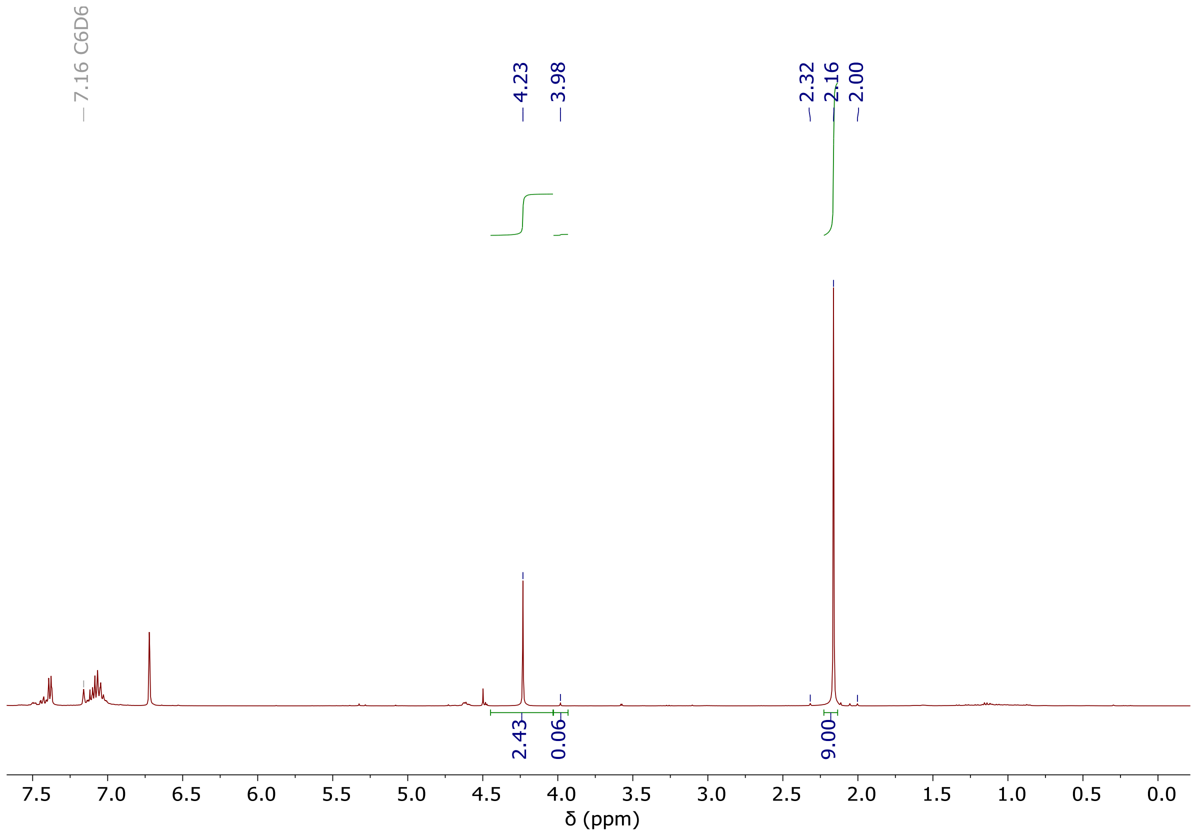

Figure S 127. In-situ ^{1}H NMR of dehydrogenative coupling of $PhSiH_3$ with 1 mol% **4c** after 3 hours with mesitylene as standard to determine consumption.

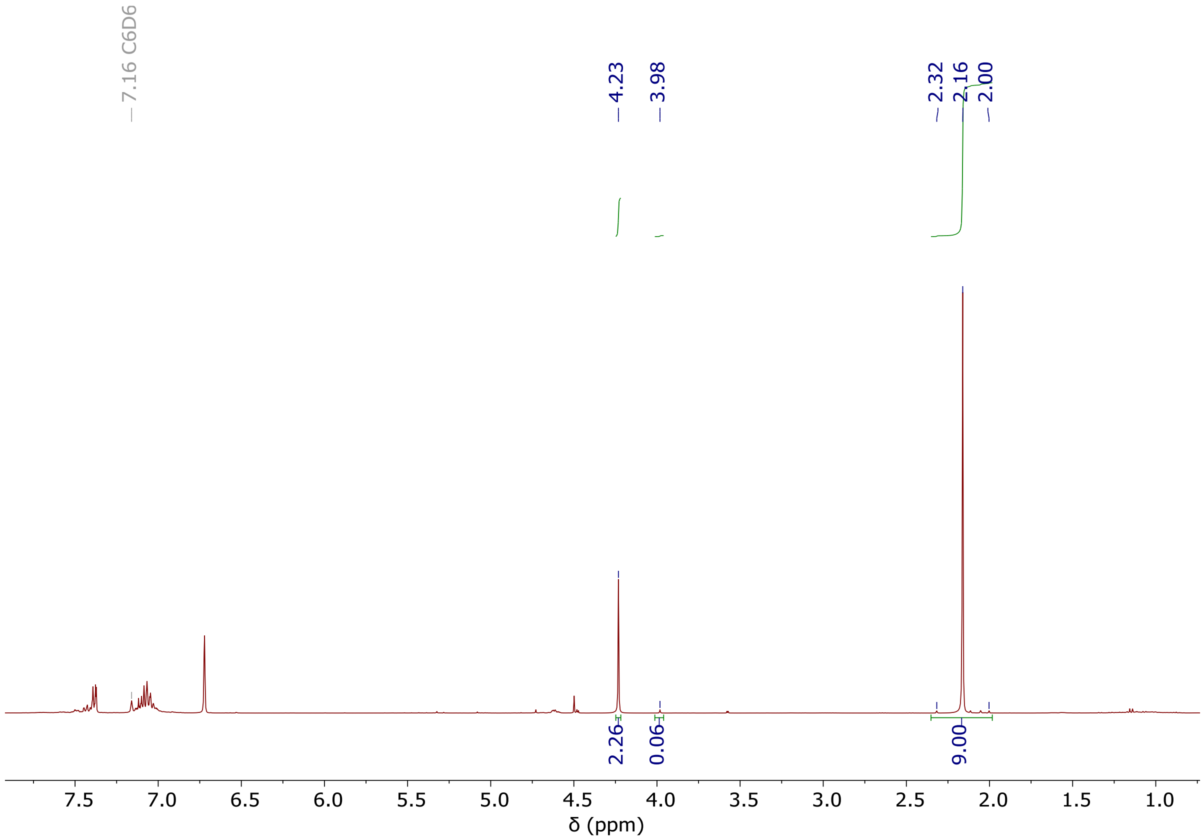

Figure S 128. In-situ ^{1}H NMR of dehydrogenative coupling of $PhSiH_3$ with 1 mol% **4d** after 3 hours with mesitylene as standard to determine consumption.

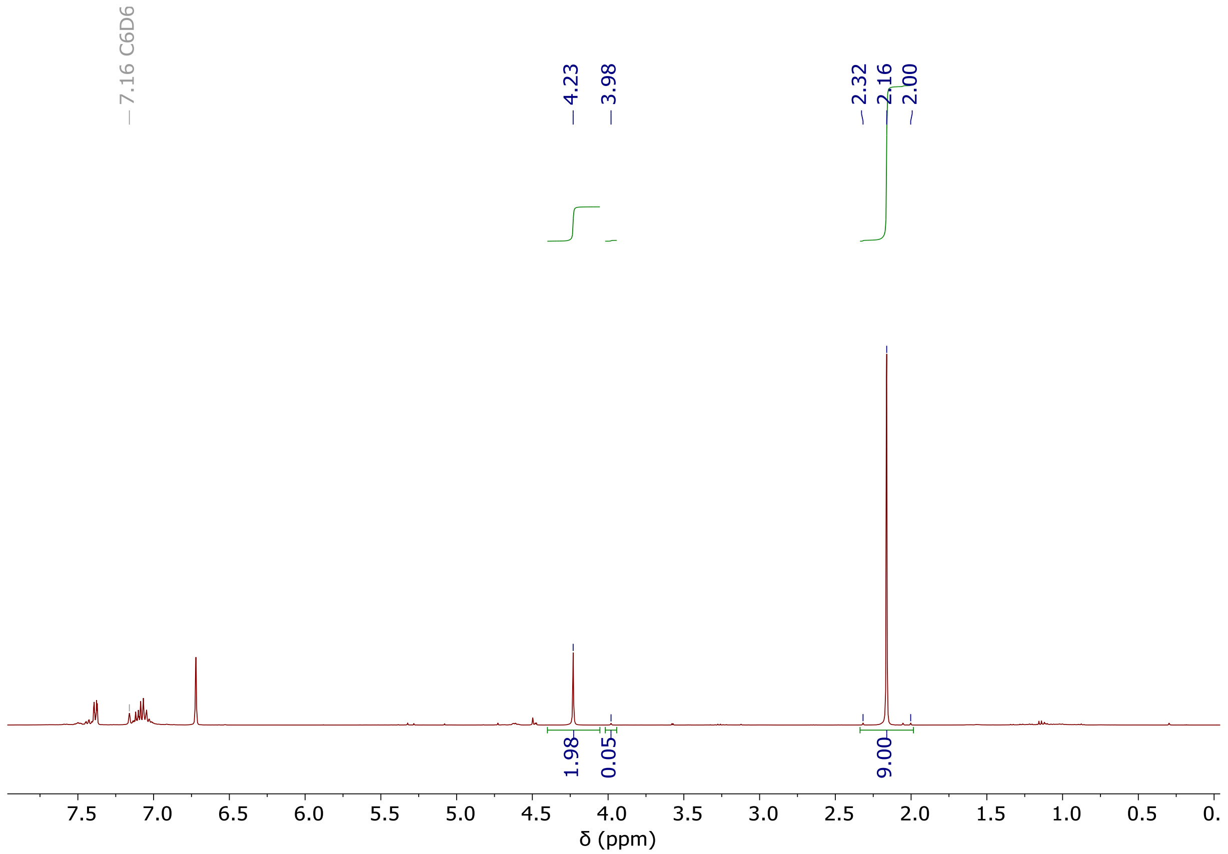

Figure S129. In-situ ^{1}H NMR of dehydrogenative coupling of $PhSiH_3$ with 1 mol% **4e** after 3 hours with mesitylene as standard to determine consumption.

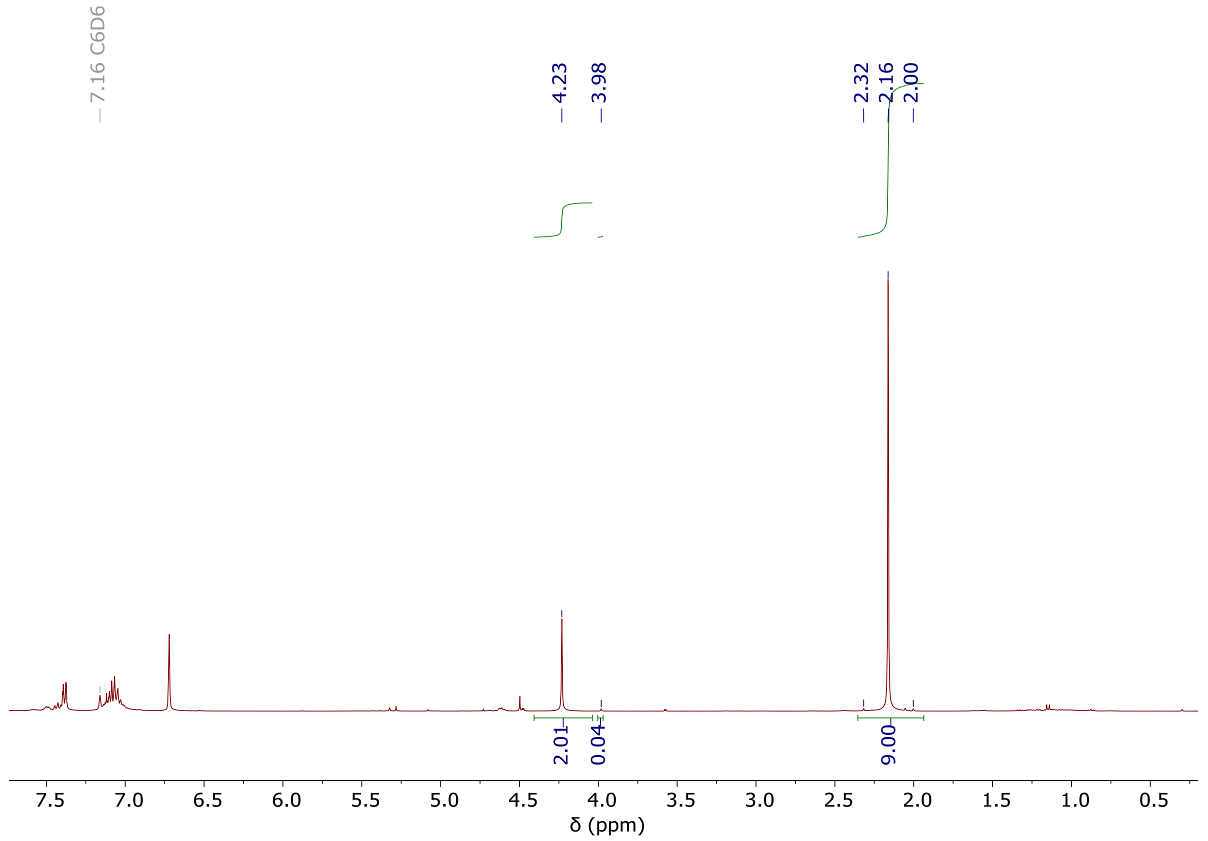

Figure S130. In-situ ^{1}H NMR of dehydrogenative coupling of $PhSiH_3$ with 1 mol% **4f** after 3 hours with mesitylene as standard to determine consumption.

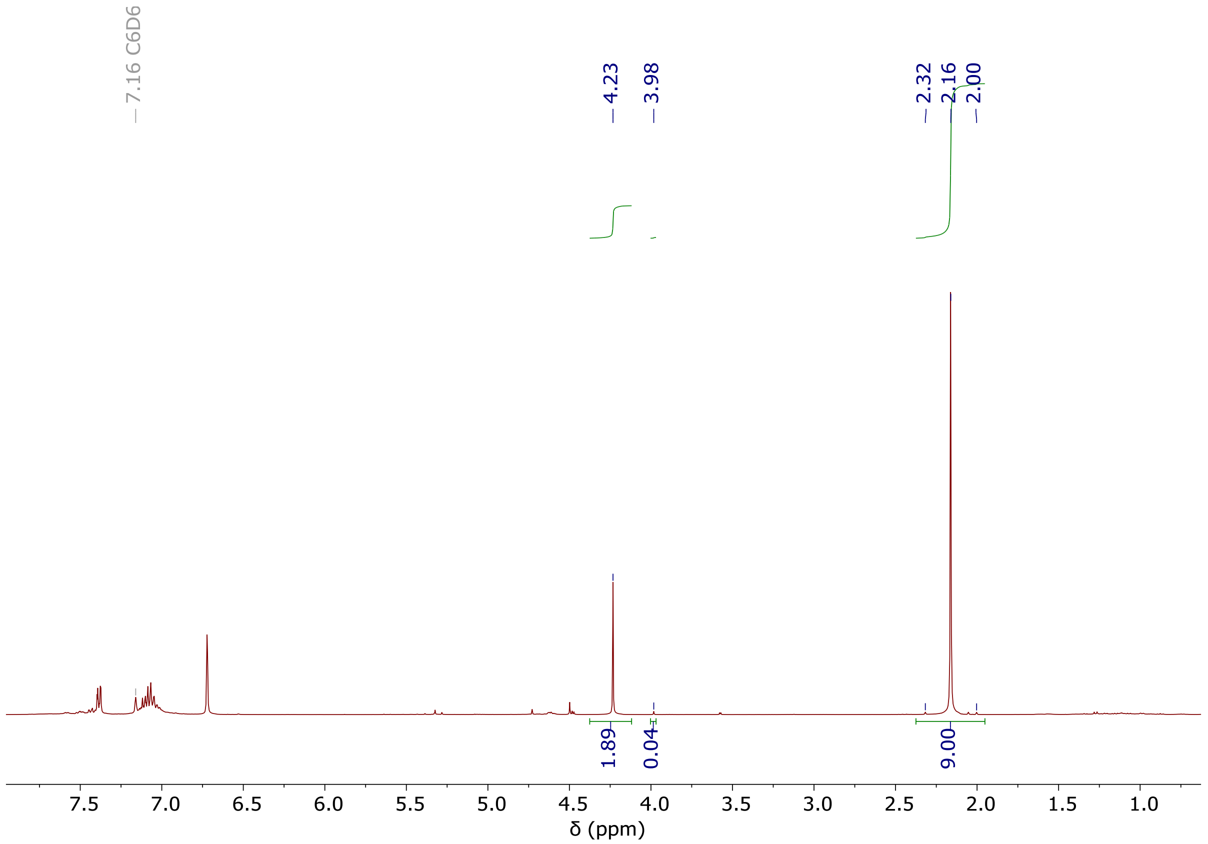

Figure S131. In-situ ^1H NMR of dehydrogenative coupling of $PhSiH_3$ with 1 mol% **4g** after 3 hours with mesitylene as standard to determine consumption.

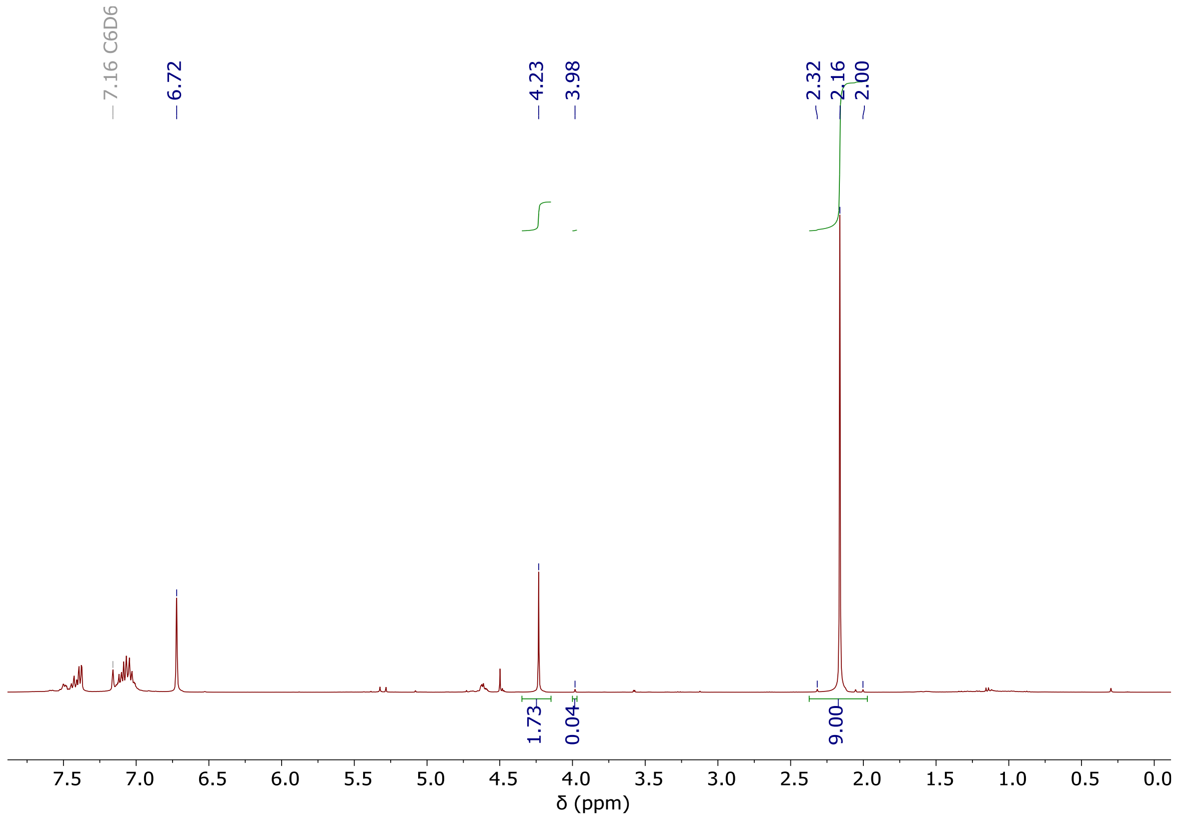

Figure S132. In-situ ^1H NMR of dehydrogenative coupling of $PhSiH_3$ with 1 mol% **3** after 3 hours with mesitylene as standard to determine consumption.

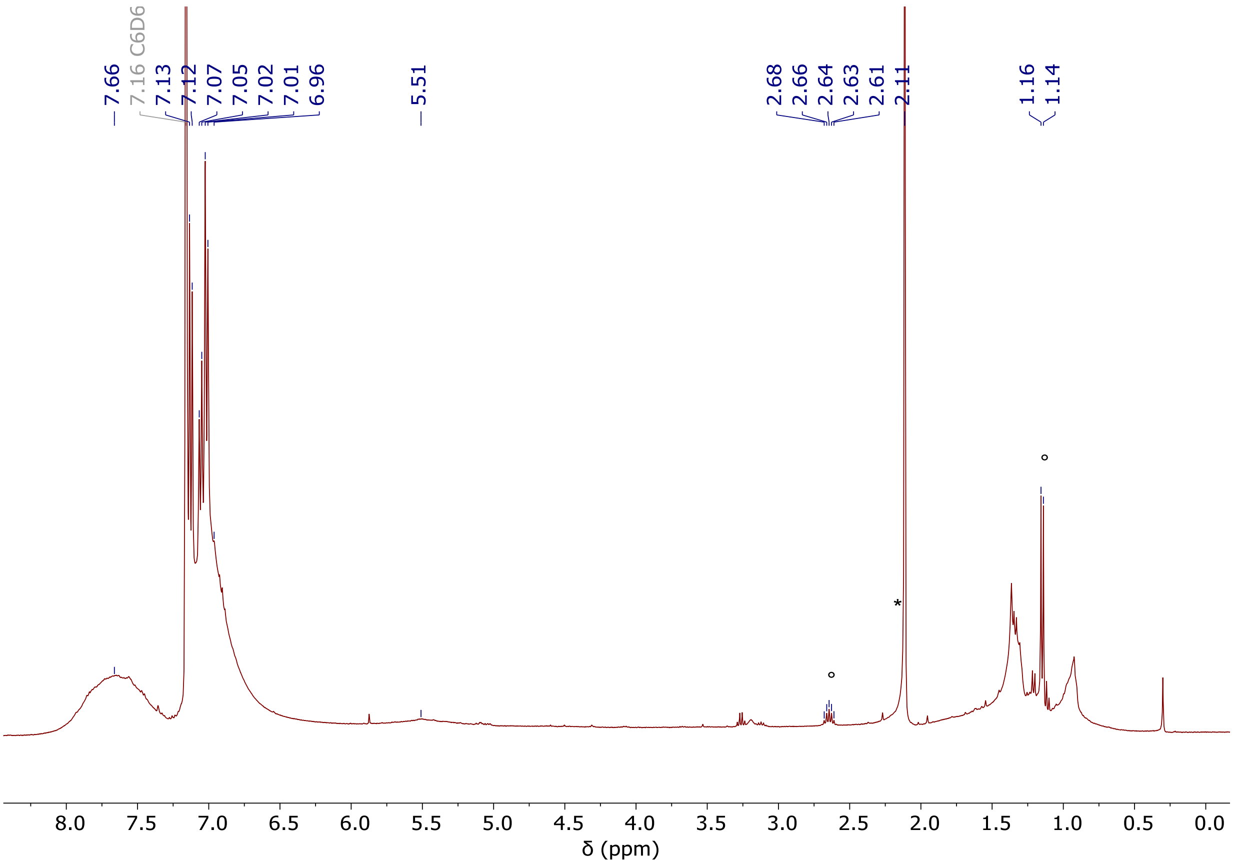

Figure S133. ^{1}H NMR of polyphenysilane from dehydrogenative coupling of $PhSiH_3$ with 2.5 mol% **3c**; * indicates presence of toluene; ° indicates presence of minor amounts of $DippNH_2$ originating from catalyst decomposition.

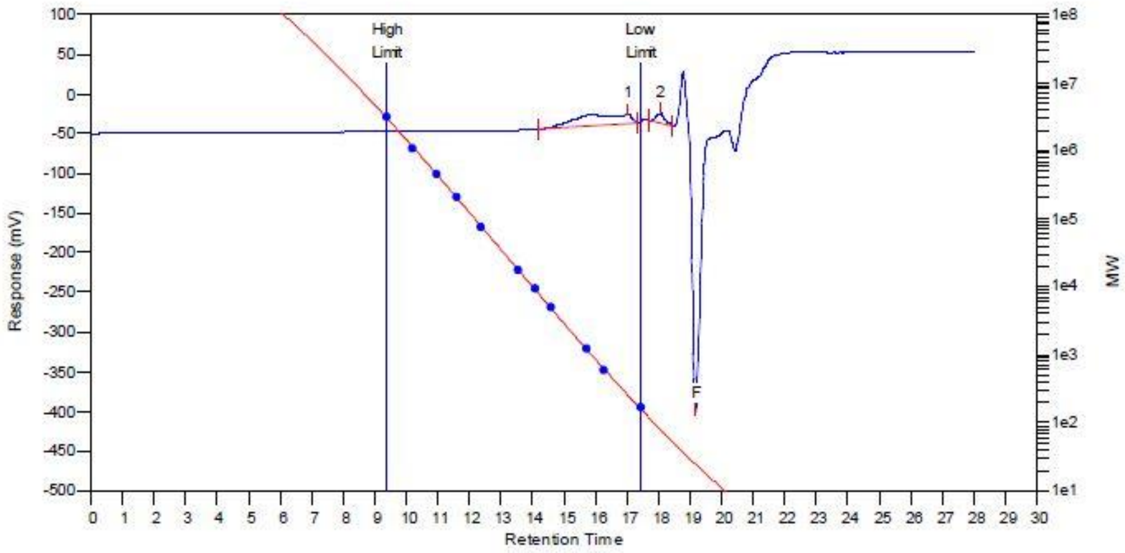

MW Averages

Peak No	Mp	Mn	Mw	Mz	Mz+1	Mv	PD
1	1080	586	1052	1817	2673	961	1.79522
2	78	78	79	81	82	79	1.01282
3	0	0	0	0	0	0	0

Figure S134. GPC-analysis of a THF solution of polyphenylsilane (3mg/mL) prepared from PhSiH3 with 2.5 mol% **3c** as catalyst.

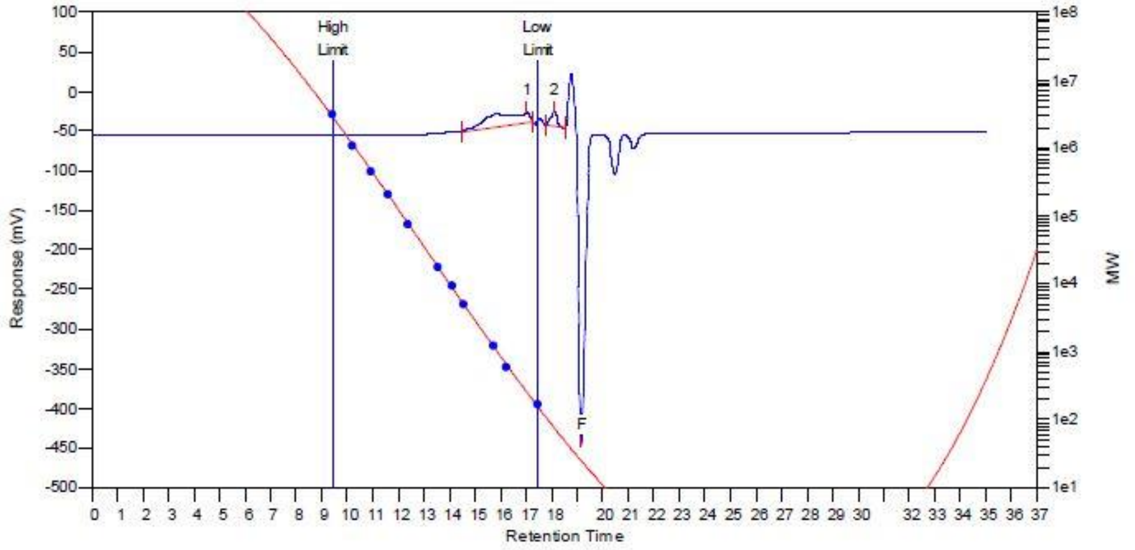

MW Averages

Peak No	Mp	Mn	Mw	Mz	Mz+1	Mv	PD
1	1084	590	996	1592	2212	921	1.68814
2	75	76	77	78	80	77	1.01316
3	0	0	0	0	0	0	0

Figure S135. GPC-analysis of a THF solution of polyphenylsilane (3mg/mL) prepared from PhSiH3 with 2.5 mol% **3c** as catalyst.

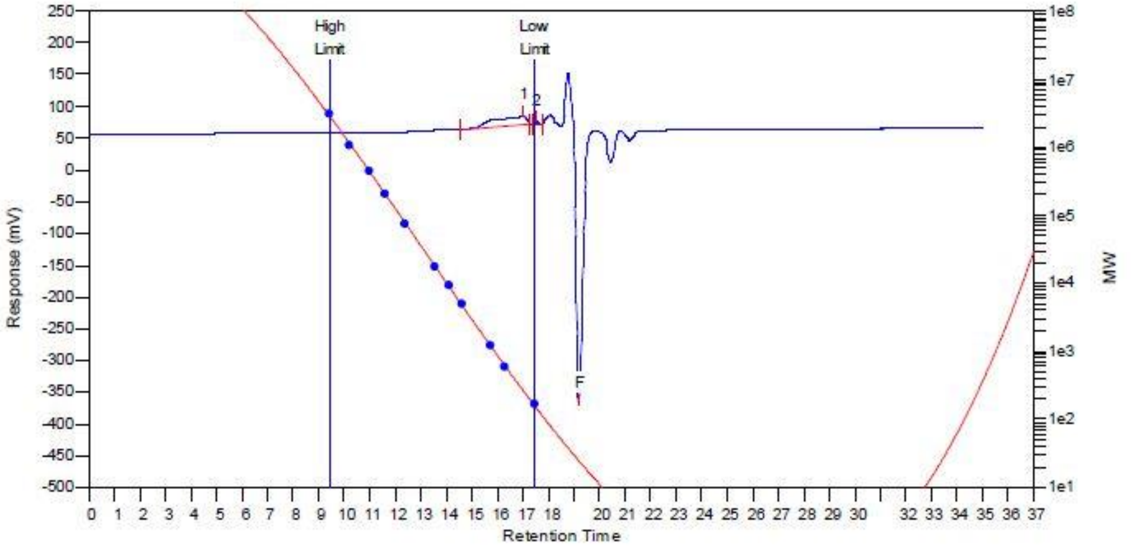

MW Averages

Peak No	Mp	Mn	Mw	Mz	Mz+1	Mv	PD
1	265	522	795	1175	1559	745	1.52299
2	144	141	142	142	143	142	1.00709
3	0	0	0	0	0	0	0

Figure S136. GPC-analysis of a THF solution of polyphenylsilane (3mg/mL) prepared from PhSiH3 with 2.5 mol% **3c** as catalyst.

2. X-ray crystallographic details

Single crystals of **2b**, **3a**-**g**, **4a**, **4c**, [^{PhiP}Dipp{(CF_3)$_2$Ph}$_2$Ge]Ni(H)·^{Dipp}NHC, and [^{PhiP}Dipp(Cl)Ge·Ni(H)]$_2$ suitable for X-ray structural analysis were mounted in perfluoroalkyl ether oil on a nylon loop and positioned in a 150 K cold N_2 gas stream. Data collection was performed with a STOE StadiVari diffractometer (MoKα radiation) equipped with a DECTRIS PILATUS 300K detector. Structures were solved by Direct Methods (SHELXS-97) and refined by full-matrix least-squares calculations against F^2 (SHELXL-2018).[1] The positions of the hydrogen atoms were calculated and refined using a riding model, aside from Ge-*H* and Ni-*H* moieties in **4a**, **4c**, [^{PhiP}Dipp{(CF_3)$_2$Ph}$_2$Ge]Ni(H)·^{Dipp}NHC, and [{^{PhiP}Dipp(Cl)Ge·Ni(H)}$_2$] which were located and freely refined. All non-hydrogen atoms were treated with anisotropic displacement parameters. Crystal data, details of data collections, and refinements for all structures can be found in their CIF files, which are available free of charge *via* www.ccdc.cam.ac.uk/data_request/cif. Details of crystallographic details are summarized in Tables S11-13.

Responses to CheckCIF B Alerts:

Compound **2b**:

PLAT910_ALERT_3_B Missing # of FCF Reflection(s) Below Theta(Min).

This relates to a small number of low-angle reflections, which does not effect the overall quality and completeness of the data (i.e. R = 4.16%; 99.9%).

Compound **3a**:

PLAT919_ALERT_3_B Reflection # Likely Affected by the Beamstop ... 1

This relates to a single low-angle reflection, and does not effect the overall quality of the data.

PLAT934_ALERT_3_B Number of (Iobs-Icalc)/Sigma(W) > 10 Outliers .. 4 Check

0 4 0, -1 2 1, 0 2 2, -1 0 3

As above, relating to 4 low-angle reflections.

PLAT990_ALERT_1_B Deprecated .res/.hkl Input Style SQUEEZE Job ... ! Note

This relates to the SQUEEZE function used to remove highly disordered solvent during refinement of this structure. All details of number of solvent molecules removed can be found in the CIF.

Compound **3b**:

PLAT213_ALERT_2_B Atom F6 has ADP max/min Ratio ..... 4.6 prolat

This is due to rotational disorder in the CF_3 groups of the Ge-Ar fragment, which was not improved by modelling.

PLAT213_ALERT_2_B Atom C46 has ADP max/min Ratio ..... 4.2 prolat

As above.

PLAT220_ALERT_2_B NonSolvent Resd 1 C Ueq(max)/Ueq(min) Range 9.0 Ratio

As above.

PLAT919_ALERT_3_B Reflection # Likely Affected by the Beamstop ... 1 Check 1 1 2,

This relates to a single low-angle reflection, and does not effect the overall quality of the data.

PLAT934_ALERT_3_B Number of (Iobs-Icalc)/Sigma(W) > 10 Outliers .. 4 Check -1 1 2, 1 1 2, 0 0 4, -4 5 8

As above, relating to 4 low-angle reflections.

PLAT990_ALERT_1_B Deprecated .res/.hkl Input Style SQUEEZE Job ... ! Note

This relates to the SQUEEZE function used to remove highly disordered solvent during refinement of this structure. All details of number of solvent molecules removed can be found in the CIF.

Compound **3c**:

PLAT990_ALERT_1_B Deprecated .res/.hkl Input Style SQUEEZE Job ... ! Note

This relates to the SQUEEZE function used to remove highly disordered solvent during refinement of this structure. All details of number of solvent molecules removed can be found in the CIF.

Compound **3d**:

PLAT990_ALERT_1_B Deprecated .res/.hkl Input Style SQUEEZE Job ... ! Note

This relates to the SQUEEZE function used to remove highly disordered solvent during refinement of this structure. All details of number of solvent molecules removed can be found in the CIF.

Compound **3e**:

PLAT910_ALERT_3_B Missing # of FCF Reflection(s) Below Theta(Min). 11 Note

1 1 0, 0 2 0, 1 2 0, -1 0 1, 1 0 1, -1 1 1, 0 1 1, 1 1 1, 0 2 1, 0 0 2, 0 1 2

This relates to a small number of low-angle reflections, which does not effect the overall quality and completeness of the data (i.e. R = 6.24%; 99.7%).

PLAT990_ALERT_1_B Deprecated .res/.hkl Input Style SQUEEZE Job ... ! Note

This relates to the SQUEEZE function used to remove highly disordered solvent during refinement of this structure. All details of number of solvent molecules removed can be found in the CIF.

Compound **3f**:

PLAT990_ALERT_1_B Deprecated .res/.hkl Input Style SQUEEZE Job ... ! Note

This relates to the SQUEEZE function used to remove highly disordered solvent during refinement of this structure. All details of number of solvent molecules removed can be found in the CIF.

Compound **3g**:

PLAT910_ALERT_3_B Missing # of FCF Reflection(s) Below Theta(Min). 11 Note

1 0 0, 0 1 0, 1 1 0, -1 -1 1, 0 -1 1, -1 0 1,0 0 1, 1 0 1, 0 1 1, 1 1 1, 0 0 2,

This relates to a small number of low-angle reflections, which does not effect the overall quality and completeness of the data (i.e. R = 4.76%; 99.9%).

Compound **4a**:

PLAT990_ALERT_1_B Deprecated .res/.hkl Input Style SQUEEZE Job ... ! Note

This relates to the SQUEEZE function used to remove highly disordered solvent during refinement of this structure. All details of number of solvent molecules removed can be found in the CIF.

Compound **4c**:

PLAT910_ALERT_3_B Missing # of FCF Reflection(s) Below Theta(Min). 11 Note

1 0 0, 0 1 0, 1 1 0, -1 -1 1, 0 -1 1, -1 0 1,0 0 1, 1 0 1, 0 1 1, 1 1 1, 0 0 2,

This relates to a small number of low-angle reflections, which does not effect the overall quality and completeness of the data (i.e. R = 5.48%; 99.9%).

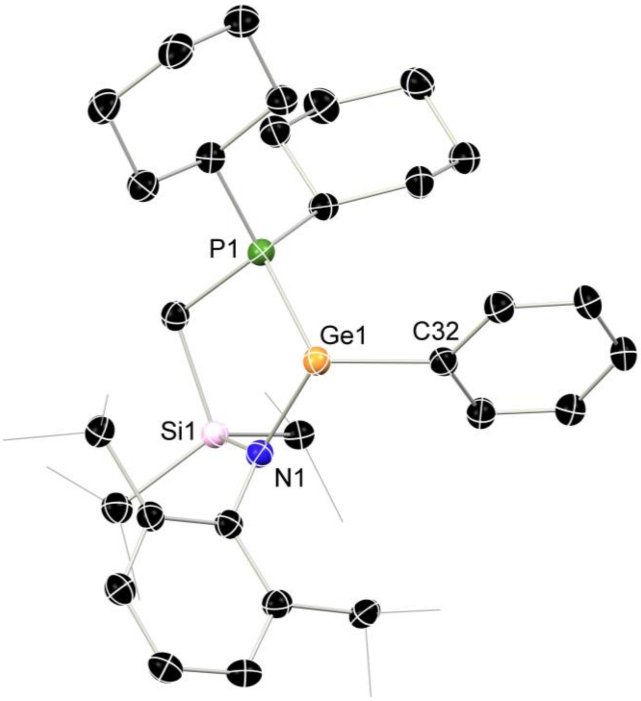

Figure S137. The molecular structure of **CyiPDippGePh,** with hydrogen atoms omitted and thermal ellipsoids at 25% probability. Selected distances and (Å) and angles (°): P1-Ge1 2.503(1), N1-Ge1 1.962(3), C32-Ge1 2.028(4), P1-Ge1-N1 88.57(9), P1-Ge1-C32 97.0(1), N1-Ge1-C32 104.9(1).

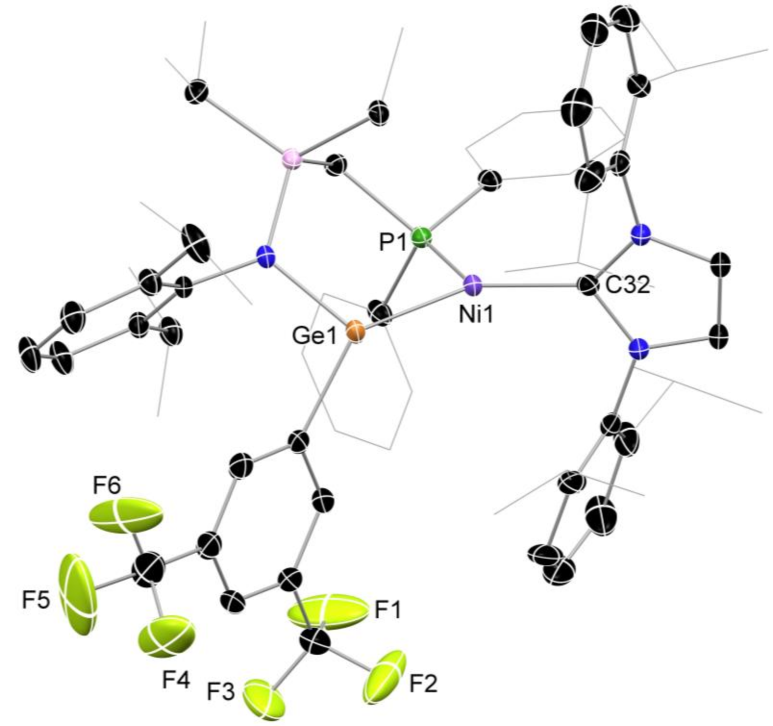

Figure S138. The molecular structure of **3b** with hydrogen atoms omitted and thermal ellipsoids at 25% probability. Selected distances and (Å) and angles (°) for **3b**: Ni1-Ge1 2.2086(9), Ge1-N1 1.901(3), Ni1-C32 1.936(4), P1-Ni1 2.143(1), Ge1-Ni1-C32 138.8(1), C32-Ni1-P1 125.3(1), P1-Ni1-Ge1 95.79(3), N1-Ge1-C59 102.5(1).

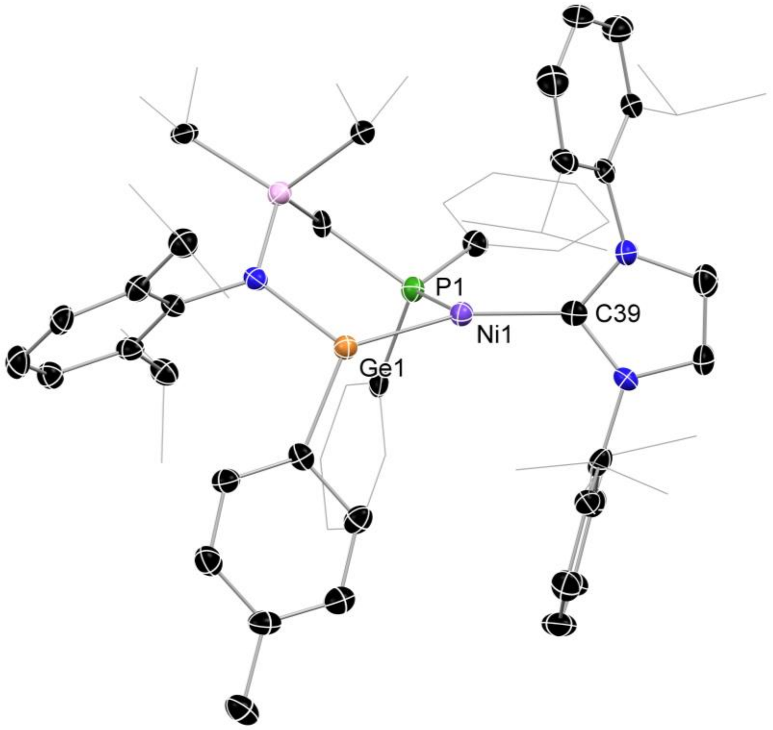

Figure S139. The molecular structure of **3d** with hydrogen atoms omitted and thermal ellipsoids at 25% probability. Selected distances and (Å) and angles (°) for **3d**: Ni1-Ge1 2.231(1), Ge1-N1 1.897(4), Ni1-C39 1.907(6),Ge1-Ni1-C39 138.1(2), C39-Ni1-P1 124.3(2), P1-Ni1-Ge1 97.35(75).

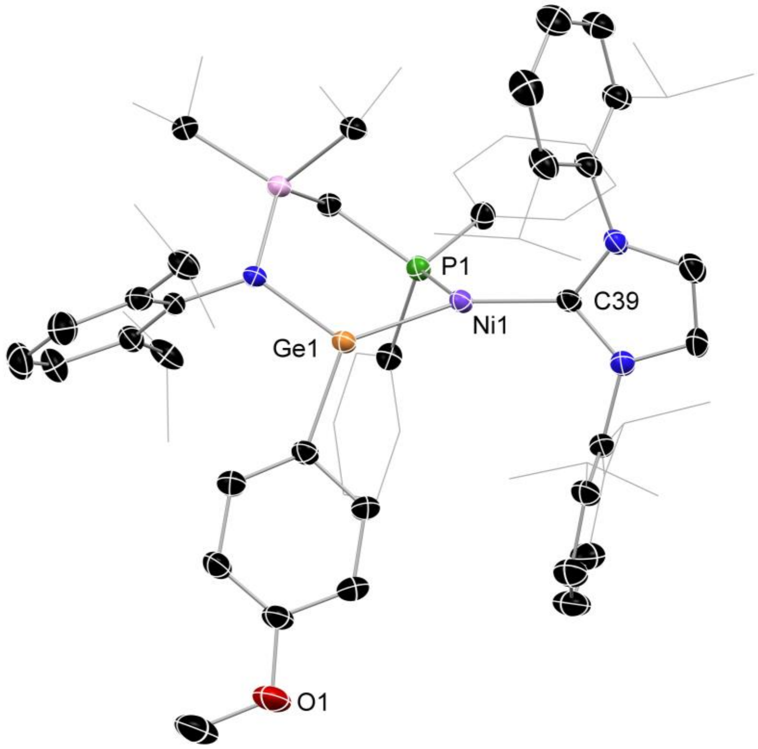

Figure S140. The molecular structure of **3e** with hydrogen atoms omitted and thermal ellipsoids at 25% probability. Selected distances and (Å) and angles (°) for **3e**: Ni1-Ge1 2.2203(8), Ge1-N1 1.885(3), Ni1-C39 1.904(5),Ge1-Ni1-C39 137.1(1), C39-Ni1-P1 126.8(1), P1-Ni1-Ge1 95.89(4).

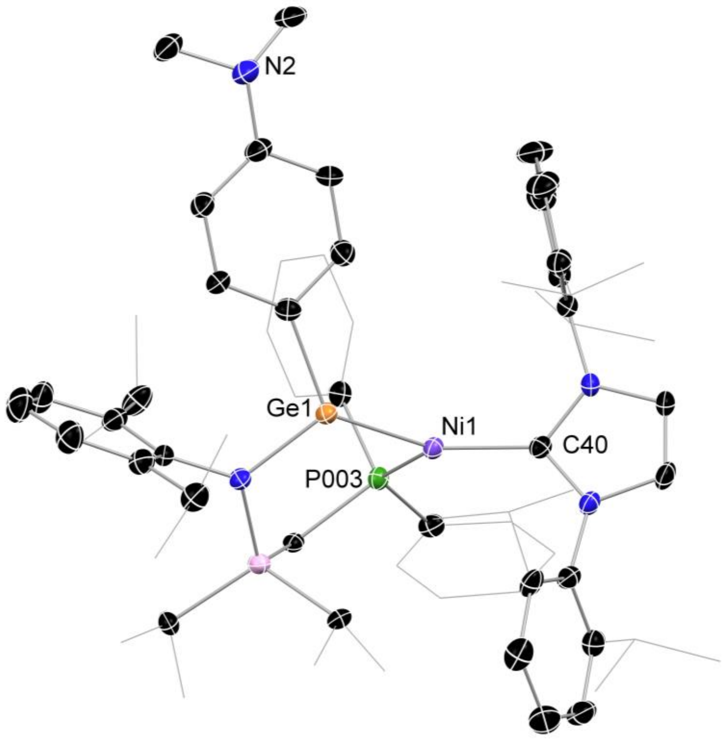

Figure S141. The molecular structure of **3f** with hydrogen atoms omitted and thermal ellipsoids at 25% probability. Selected distances and (Å) and angles (°) for **3f**: Ni1-Ge1 2.2176(9), Ge1-N1 1.893(4), Ni1-C40 1.901(5),Ge1-Ni1-C40 136.6(1), C40-Ni1-P003 127.3(1), P003-Ni1-Ge1 95.89(5).

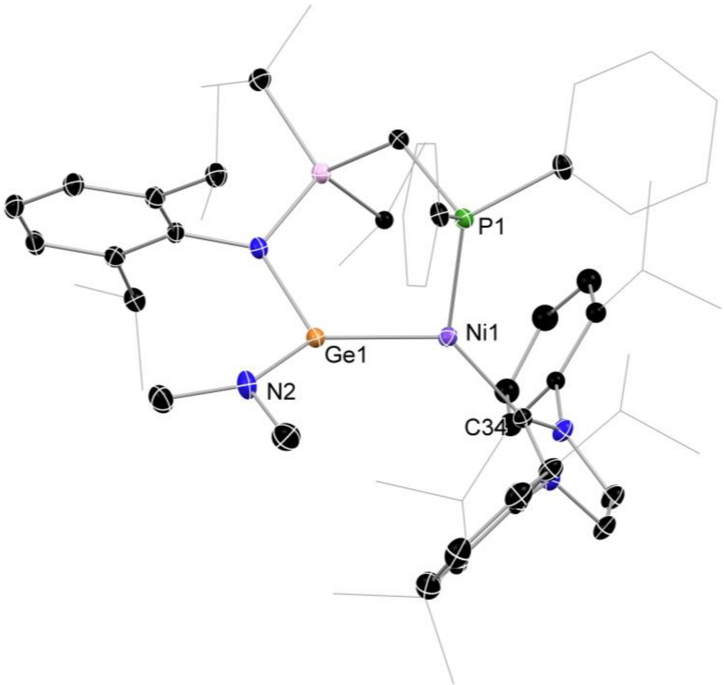

Figure S142. The molecular structure of **3g** with hydrogen atoms omitted and thermal ellipsoids at 25% probability. Selected distances and (Å) and angles (°) for **3g**: Ni1-Ge1 2.2162(9), Ge1-N1 1.892(2), Ge1-N2 1.848(4), Ni1-C34 1.914(3), Ge1-Ni1-C34 131.6(1), C34-Ni1-P1 132.4(1), P1-Ni1-Ge1 95.93(3).

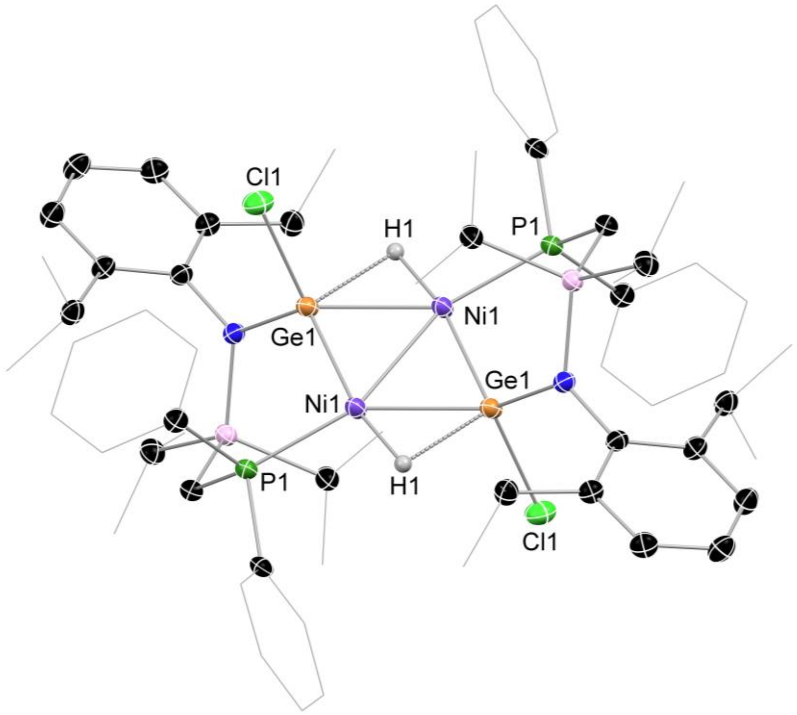

Figure S143. The molecular structure of [^{PhiP}Dipp(Cl)Ge·Ni(H)]$_2$ with hydrogen atoms omitted and thermal ellipsoids at 25% probability. Selected distances and (Å) and angles (°) for [^{PhiP}Dipp(Cl)Ge·Ni(H)]$_2$: Ni1-Ge1 2.2176(9), Ge1-N1 1.893(4), Ni1-C40 1.901(5),Ge1-Ni1-C40 136.6(1), C40-Ni1-P003 127.3(1), P003-Ni1-Ge1 95.89(5).

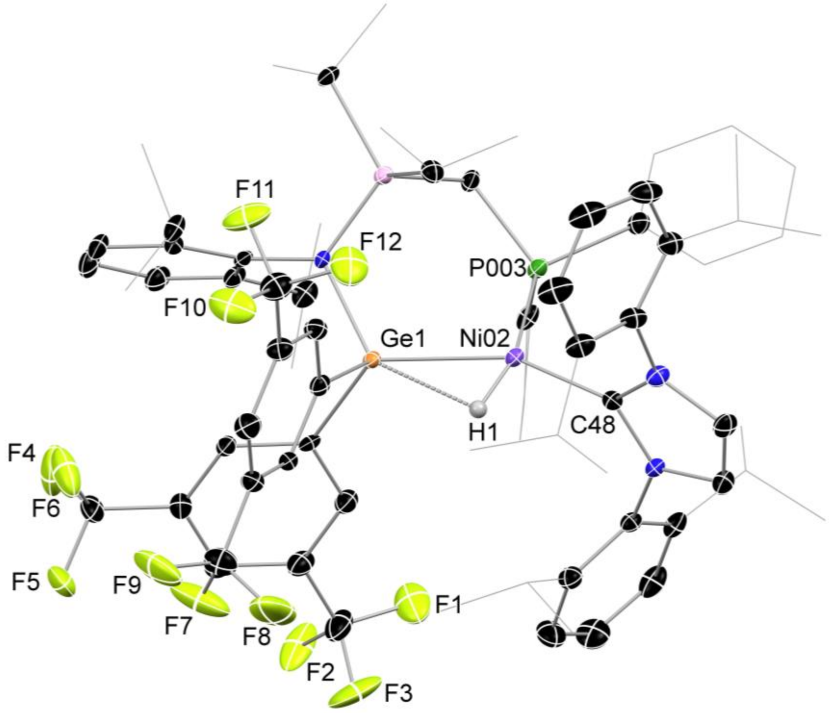

Figure S144. The molecular structure of [^{PhiP}Dipp{$(CF_3)_2$Ph}$_2$Ge]Ni(H)·^{Dipp}NHC with hydrogen atoms omitted and thermal ellipsoids at 25% probability. Selected distances and (Å) and angles (°) for [^{PhiP}Dipp{$(CF_3)_2$Ph}$_2$Ge]Ni(H)·^{Dipp}NHC: Ni02-Ge1 2.375(1), Ni02-C48 1.961(6), Ni02-P003 2.237(2), Ni02-H1 1.21(7), Ge1⋯Ni02 2.07(7), Ge1-Ni02-C48 147.5(2), C48-Ni02-P003 113.8(2), Ge1-Ni02-H1 61(3), H1-Ni02-C48 87(3), Ge1-Ni02-C48 147.5(2).

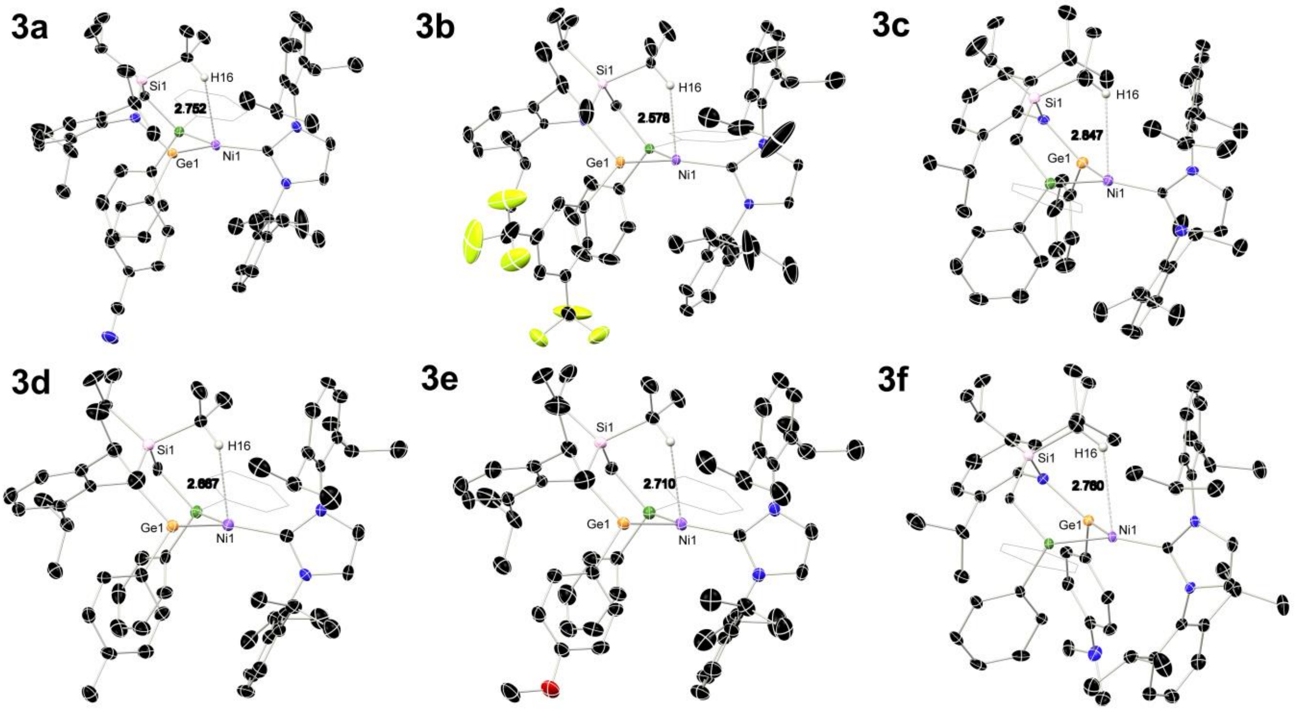

Figure S145. Representation of the agostic interactions observed in complexes **3a**-**3f**.

Table S11. Crystallographic details for **2b**, **2c**, **3a**, **3b**, and **3c**.

	**2b**	**2c**	**3a**	**3b**	**3c**
empirical form.	$C_{39}H_{46}F_6GeNPSi$	$C_{37}H_{48}GeNPSi$	$C_{65}H_{83}GeN_4NiPSi$	$C_{66}H_{82}F_6GeN_3NiPSi$	$C_{64}H_{84}GeN_3NiPSi$
formula wt	774.42	638.41	1190.85	1221.70	1085.70
crystal syst.	triclinic	orthorhombic	monoclinic	monoclinic	triclinic
space group	*P-1*	$P2_12_12_1$	$P2_1/n$	$P2_1/c$	*P-1*
a (Å)	12.310(3)	12.044(2)	13.882(3)	14.330(3)	12.650(3)
b (Å)	14.320(3)	14.955(3)	22.527(5)	20.120(4)	13.610(3)
c (Å)	22.750(5)	38.305(8)	21.112(4)	23.510(5)	19.970(4)
α (deg.)	97.70(3)	90	90	90	79.10(3)
β (deg.)	95.30(3)	90	99.59(3)	97.60(3)	74.20(3)
γ (deg.)	96.20(3)	90	90	90	71.00(3)
vol ($Å^3$)	3927.6(14)	6899(2)	6510(2)	6719(2)	3108.9(13)
Z	4	8	4	4	2
ρ(calc) ($g.cm^{-3}$)	1.310	1.133	1.133	1.208	1.160
μ (mm^{-1})	0.907	0.994	0.832	0.824	0.870
F(000)	1608	2704	2360	2568	1156
T (K)	150(2)	150(2)	150(2)	150(2)	150(2)
reflns collect.	53284	45574	89512	40322	38461
unique reflns	15417	13336	12785	13162	12208
R_{int}	0.0395	0.0549	0.0944	0.0430	0.1565
R1 [$I>2\sigma(I)$]	0.0416	0.0447	0.0490	0.0570	0.0862
*w*R2 (all data)	0.0933	0.1114	0.1161	0.1514	0.2239
CCDC No.	2312484	2312485	2312486	2312487	2312488

Table S12. Crystallographic details for **3d**-**3g**, and **4a**.

	**3d**	**3e**	**3f**	**3g**	**4a**
empirical form.	$C_{65}H_{86}GeN_3NiPSi$	$C_{65}H_{86}GeN_3NiOPSi$	$C_{66}H_{89}GeN_4NiPSi$	$C_{60}H_{85}GeN_4NiPSi$	$C_{62}H_{85}GeN_4NiPSi\cdot0.5(C_6H_{14})$
formula wt	1099.72	1115.72	1128.77	1052.67	1155.81
crystal syst.	monoclinic	monoclinic	monoclinic	triclinic	monoclinic
space group	*P2₁/n*	*P2₁/n*	*P2₁/n*	*P-1*	*P2₁/n*
a (Å)	13.880(3)	14.060(3)	14.108(3)	12.632(3)	13.953(3)
b (Å)	22.720(5)	22.580(5)	22.317(5)	13.335(3)	22.828(5)
c (Å)	20.900(4)	21.370(4)	21.590(4)	19.285(4)	20.809(4)
α (deg.)	90	90	90	84.11(3)	90
β (deg.)	100.50(3)	101.00(3)	101.55(3)	89.52(3)	100.55(3)
γ (deg.)	90	90	90	61.95(3)	90
vol (Å^3)	6481(2)	6660(2)	6660(2)	2848.9(12)	6516(2)
Z	4	4	4	2	4
ρ(calc) (g.cm^{-3})	1.127	1.113	1.126	1.227	1.178
μ (mm^{-1})	0.835	0.815	0.815	0.947	0.834
F(000)	2344	2376	2408	1124	2468
T (K)	150(2)	150(2)	150(2)	150(2)	150(2)
reflns collect.	56743	45595	46377	11210	47185
unique reflns	12727	12976	12961	11210	12764
R_{int}	0.1475	0.0741	0.1015	0.0550	0.0398
R1 [I>2σ(I)]	0.0726	0.0624	0.0696	0.0476	0.0457
*w*R2 (all data)	0.1638	0.1434	0.1757	0.1178	0.1138
CCDC No.	2312489	2312490	2312491	2312492	2312493

Table S13. Crystallographic details for **4c**, [^{PhiP}Dipp{(CF_3)$_2$Ph}$_2$Ge]Ni(H)·^{Dipp}NHC, and [^{PhiP}Dipp(Cl)Ge·Ni(H)]$_2$.

	**4c**	**[^{PhiP}Dipp{(CF_3)$_2$Ph}$_2$Ge] Ni(H)·^{Dipp}NHC**	**[^{PhiP}Dipp(Cl)Ge·Ni(H)]$_2$**	**^{CyiP}DippGePh**
empirical form.	$C_{64}H_{86}GeN_3Ni$ PSi·Et_2O	$C_{74}H_{86}F_{12}GeN_3NiPSi$	$C_{62}H_{88}Cl_2Ge_2N_2Ni_2P_2Si_2$	$C_{37}H_{60}GeNPSi$
formula wt	1161.83	1435.81	1312.96	650.51
crystal syst.	triclinic	monoclinic	monoclinic	triclinic
space group	*P-1*	*P2$_1$/n*	*C2/c*	*P-1*
a (Å)	12.740(3)	13.960(3)	28.730(6)	9.020(2)
b (Å)	13.890(3)	22.100(4)	13.500(3)	10.160(2)
c (Å)	19.630(4)	23.960(5)	20.873(4)	21.620(4)
α (deg.)	81.90(3)	90	90	84.70(3)
β (deg.)	84.30(3)	96.00(3)	128.76(3)	80.40(3)
γ (deg.)	66.30(3)	90	90	67.00(3)
vol (Å^3)	3145.5(13)	7352(3)	6313(3)	1797.5(7)
Z	2	4	4	2
ρ(calc) (g.cm^{-3})	1.227	1.297	1.381	1.202
μ (mm^{-1})	0.865	0.775	1.743	0.955
F(000)	1244	2992	2744	700
T (K)	150(2)	150(2)	150(2)	150(2)
reflns collect.	42397	51199	23486	16302
unique reflns	12355	14419	6195	7064
R_{int}	0.0766	0.0781	0.0371	0.0313
R1 [$I>2\sigma(I)$]	0.0548	0.0815	0.0368	0.0570
*w*R2 (all data)	0.1313	0.2130	0.0843	0.0621
CCDC No.	2312494	2312482	2312483	2363825

3. Computational methods

DFT calculations for the reaction mechanism discovery were performed at the B97-D3(SMD=benzene)/def2-TZVPP//B97-D3/def2-SVP level of theory.[10-12] To identify stationary points on the potential energy surface (PES), harmonic vibrational frequency calculations were conducted. Transition states, distinguished by a singular imaginary frequency, were further validated using intrinsic reaction coordinate (IRC) calculations to confirm their status as intermediates. All calculations were executed using the GAUSSIAN 16 software suite[13]. All computed energetics were corrected from gas phase standard state to liquid phase standard state by adding a correction term 1.89 kcal mol^{-1} at 298 K.[14]

We performed Natural Bond Orbital (NBO) analysis with the NBO program (version 3.1)[15] as implemented in GAUSSIAN 16 at the B97-D3/def2-TZVPP level of theory using the optimized geometry gotten from the B97-D3/def2-SVP level of theory. To produce the molecular orbitals, the optimized geometries were taken from the checkpoint file produced during NBO analysis at B97-D3/def2-TZVPP level of theory. By using the formchk utility program4 a formatted checkpoint file is generated, from this file the molecular orbitals were pre-generated and visualized using the Avogadro program (version 1.20).[16,17]

Optimized catalyst structures

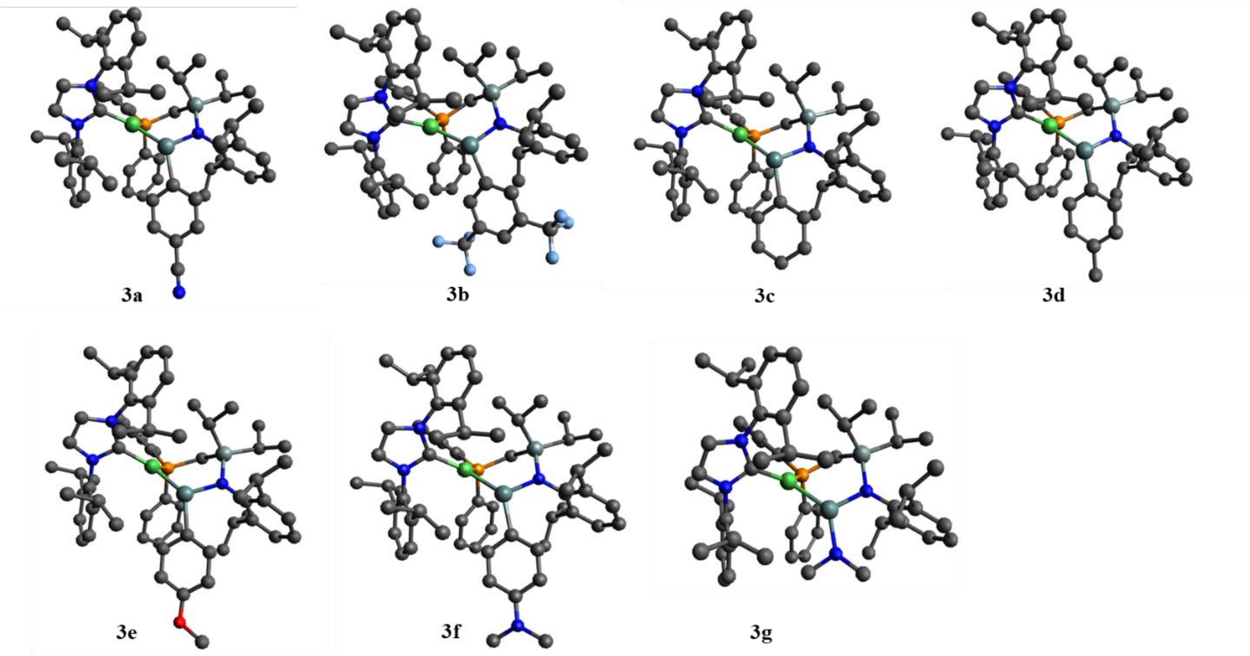

Figure S146. Optimized geometries for catalyst with different substituents on Ge (**3a-3g**). Hydrogen atoms are omitted for clarity. Color code: C – gray, Ge – teal, N – blue, Ni – green, P – orange, Si – darker-teal.

Electronic structure analysis

Table S14. Calculated bond length [Å], NPA, Wiberg Bond Index (WBI), and Mayer Bond Order (MBO) in complex **3a**.

**Property**	**3a**	
**Bond length [Å]**	Ni-Ge	2.240
**NPA charge**	Ni/Ge	-0.515/+1.129
**Wiberg Bond Index**	Ni-Ge	0.947
**Mayer Bond Order**	Ni-Ge	1.106

Table S15. Calculated bond length [Å], NPA, Wiberg Bond Index (WBI), and Mayer Bond Order (MBO) in complex **3b**.

**Property**	**3b**	
**Bond length [Å]**	Ni-Ge	2.243
**NPA charge**	Ni/Ge	-0.512/+1.116
**Wiberg Bond Index**	Ni-Ge	0.955
**Mayer Bond Order**	Ni-Ge	1.107

Table S16. Calculated bond length [Å], NPA, Wiberg Bond Index (WBI), and Mayer Bond Order (MBO) in complex **3c**.

**Property**	**3c**	
**Bond length [Å]**	Ni-Ge	2.241
**NPA charge**	Ni/Ge	-0.534/+1.147
**Wiberg Bond Index**	Ni-Ge	0.946
**Mayer Bond Order**	Ni-Ge	1.121

Table S17. Calculated bond length [Å], NPA, Wiberg Bond Index (WBI), and Mayer Bond Order (MBO) in complex **3d**.

**Property**	**3d**	
**Bond length [Å]**	Ni-Ge	2.242
**NPA charge**	Ni/Ge	-0.535/+1.150
**Wiberg Bond Index**	Ni-Ge	0.945
**Mayer Bond Order**	Ni-Ge	1.119

Table S18. Calculated bond length [Å], NPA, Wiberg Bond Index (WBI), and Mayer Bond Order (MBO) in complex **3e**.

**Property**	**3e**	
**Bond length [Å]**	Ni-Ge	2.242
**NPA charge**	Ni/Ge	-0.539/+1.152
**Wiberg Bond Index**	Ni-Ge	0.943
**Mayer Bond Order**	Ni-Ge	1.130

Table S19. Calculated bond length [Å], NPA, Wiberg Bond Index (WBI), and Mayer Bond Order (MBO) in complex **3f**.

**Property**	**3f**	
**Bond length [Å]**	Ni-Ge	2.243
**NPA charge**	Ni/Ge	-0.546/+1.160
**Wiberg Bond Index**	Ni-Ge	0.936
**Mayer Bond Order**	Ni-Ge	1.130

Table S20. Calculated bond length [Å], NPA, Wiberg Bond Index (WBI), and Mayer Bond Order (MBO) in complex **3g**.

**Property**	**3g**	
**Bond length [Å]**	Ni-Ge	2.252
**NPA charge**	Ni/Ge	-0.586/+1.313
**Wiberg Bond Index**	Ni-Ge	0.873
**Mayer Bond Order**	Ni-Ge	1.230

Correlation between DFT calculations and experimental data

Table S21. Summary of calculated Ni-Ge bond length [Å], NPA, Wiberg Bond Index (WBI), Mayer Bond Order (MBO), experimentally measured Gibbs free energy (ΔG), enthalpy (ΔH), and entropy (ΔS) for H_2 activation.

	**Bond Length [Å]**	**NPA charges**		**Wiberg Bond Index**	**Mayer Bond Order**	**Experimental Data [kJ/mol]**		
**Catalyst**	**Ni-Ge**	**Ni**	**Ge**	**Ni-Ge**	**Ni-Ge**	**ΔG**	**ΔH**	**ΔS**
**3a**	2.240	-0.515	1.129	0.947	1.106	-21.96	-43.81	-0.073
**3b**	2.243	-0.512	1.116	0.955	1.107	-22.99	-42.69	-0.066
**3c**	2.241	-0.534	1.147	0.946	1.121	-18.67	-38.76	-0.067
**3d**	2.242	-0.535	1.150	0.945	1.119	-18.22	-37.35	-0.064
**3e**	2.242	-0.539	1.152	0.943	1.130	-16.93	-34.75	-0.060
**3f**	2.243	-0.546	1.160	0.936	1.130	-15.26	-33.09	-0.060

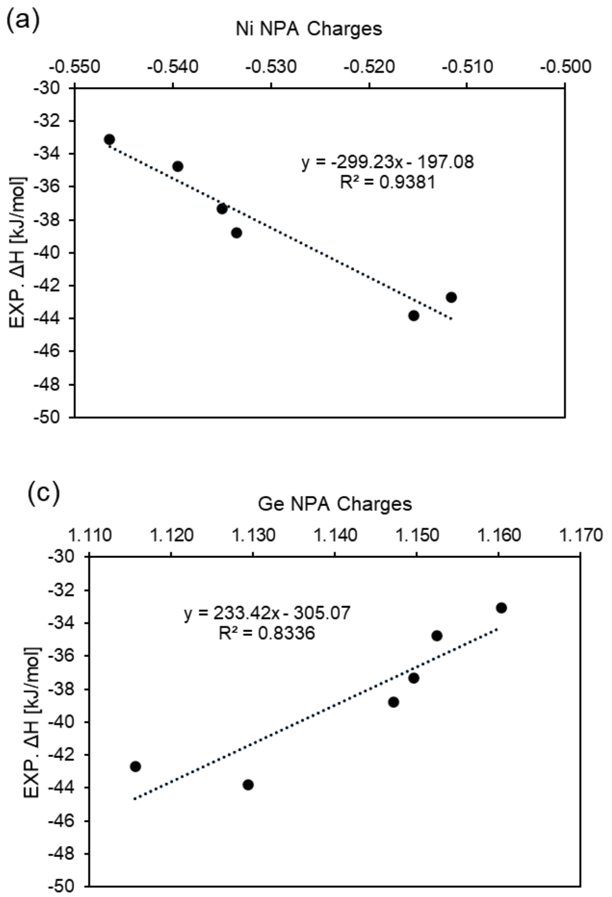

Figure S147. Correlation between experimentally measured ΔH and calculated a) Ni NPA charges, b) Ni-Ge Mayer Bond Order (MB), and c) Ge NPA charges for **3a-3f**.

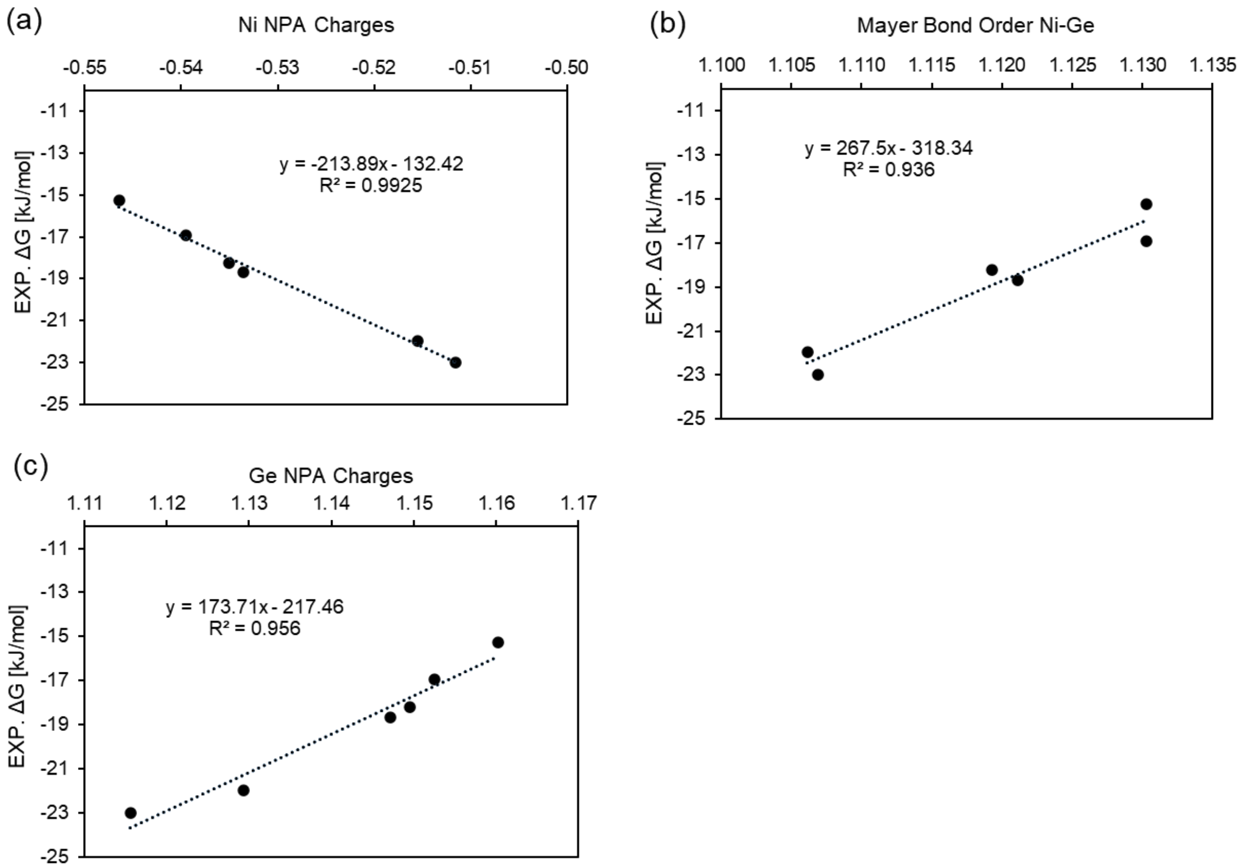

Figure S148. Correlation between experimentally measured ΔG and calculated a) Ni NPA charges, b) Ni-Ge Mayer Bond Order (MBO), and c) Ge NPA charges for **3a-3f**.

HOMO-LUMO analysis

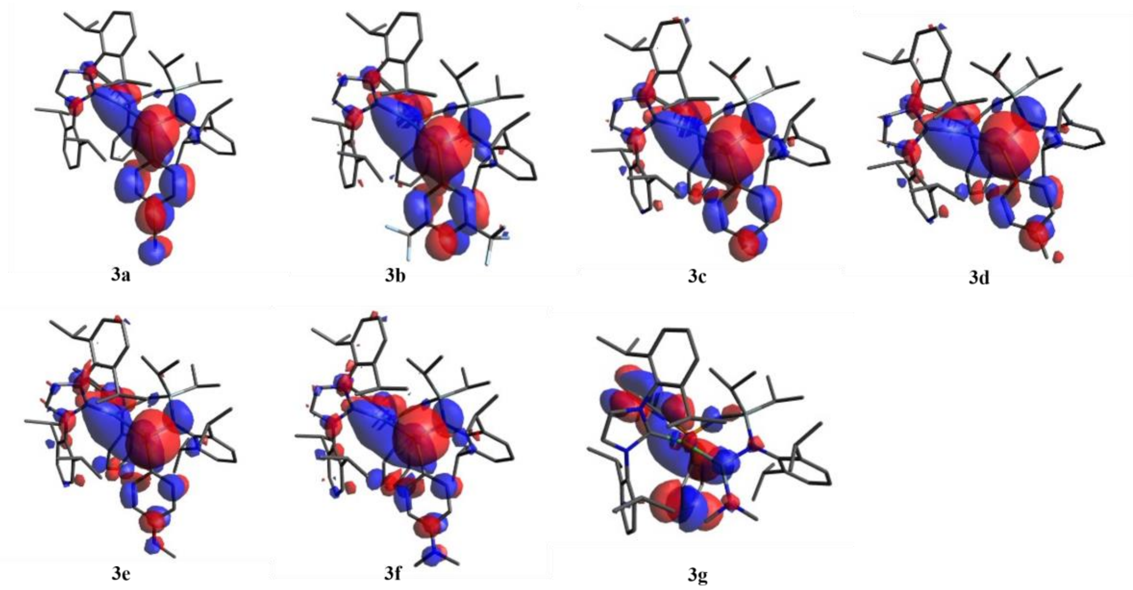

Figure S149. Lowest unoccupied molecular orbital (LUMO) of **3a-3g**. Orbital energies: **3a**: -2.35 eV, **3b**: -2.21 eV, **3c**: -1.93 eV, **3d**: -1.90 eV, **3e**: **-**1.85 eV, **3f**: -1.75 eV, and **3g**: -1.64 eV.

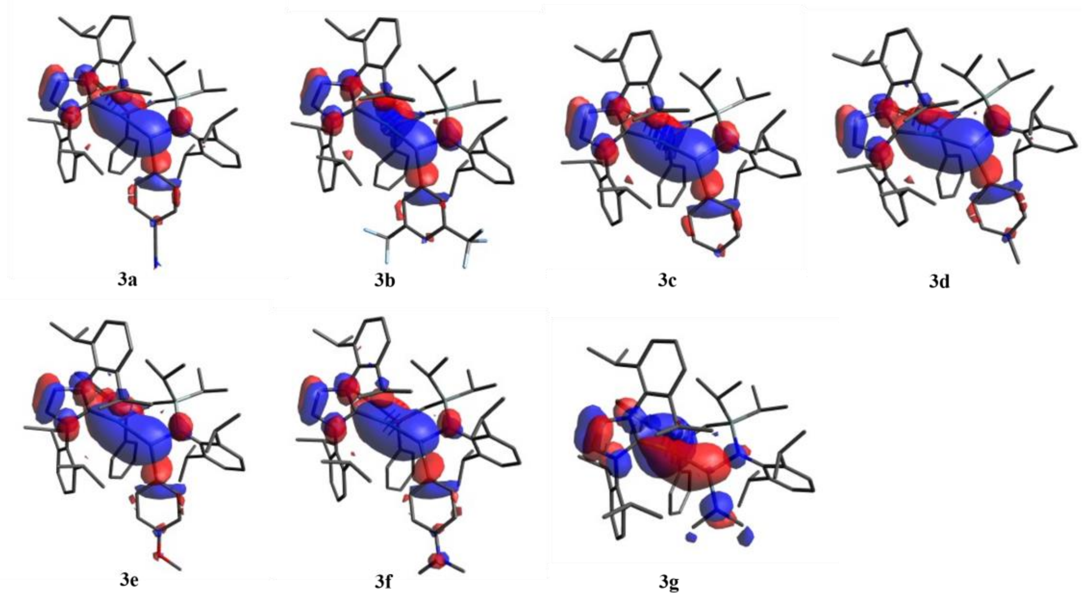

Figure S150. Highest occupied molecular orbital (HOMO) of **3a-3g**. Orbital energies: **3a**: -3.78 eV, **3b**: -3.76 eV, **3c**: -3.55 eV, **3d**: -3.52 eV, **3e**: **-**3.49 eV, **3f**: -3.39 eV, and **3g**: -3.44 eV.

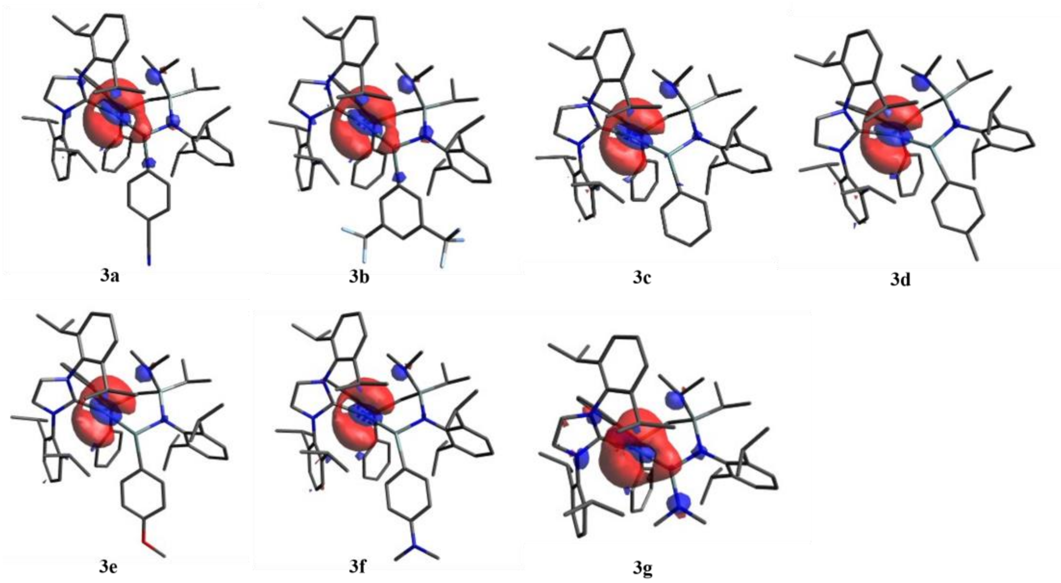

Figure S151. Second highest occupied molecular orbital (HOMO-1) of **3a-3g**. Orbital energies: **3a**: -3.84 eV, **3b**: -3.81 eV, **3c**: -3.61 eV, **3d**: -3.59 eV, **3e**: -3.56 eV, **3f**: -3.48 eV, and **3g**: -3.51 eV.

NBO analysis

Table S22. NBO analysis of the Ni-Ge moiety of **3a**.

Alpha orbitals	Occupation	Atom	Polarization	s-character	p-character	d-character
Bond	1.82	Ni	22.39%	34.09%	65.22%	0.69%
		Ge	77.61%	59.04%	40.73%	0.23%
Bond	1.81	Ni	16.98%	35.14%	61.38%	3.47%
		P	83.02%	41.40%	58.50%	0.10%
Bond	1.95	Ge	27.25%	34.27%	65.55%	0.18%
		C	72.75%	28.92%	71.00%	0.07%
Lone Pair	1.98	Ni	-	0.85%	0.04%	99.12%
Lone Pair	1.96	Ni	-	0.04%	0.22%	99.74%
Lone Pair	1.94	Ni	-	0.01%	0.18%	99.80%
Lone Pair	1.86	Ni	-	0.22%	0.91%	98.87%
Lone Pair	1.68	Ni	-	0.14%	3.18%	96.68%
Empty orbital	0.30	Ni	-	29.50%	68.06%	2.42%
Empty orbital	0.04	Ni	-	0.07%	99.26%	0.67%
Empty orbital	0.49	Ge	-	1.04%	98.86%	0.08%
Empty orbital	0.32	Ge	-	6.38%	93.41%	0.21%

Table S23. NBO analysis of the Ni-Ge moiety of **3b**.

Alpha orbitals	Occupation	Atom	Polarization	s-character	p-character	d-character
Bond	1.83	Ni	22.06%	34.14%	65.17%	0.69%
		Ge	77.94%	59.66%	40.12%	0.22%
Bond	1.80	Ni	16.96%	35.17%	61.30%	3.53%
		P	83.04%	41.18%	58.71%	0.10%
Bond	1.95	Ge	26.94%	33.63%	66.18%	0.18%
		C	73.06%	28.89%	71.05%	0.06%
Lone Pair	1.98	Ni	-	0.85%	0.04%	99.11%
Lone Pair	1.96	Ni	-	0.03%	0.21%	99.76%
Lone Pair	1.94	Ni	-	0.01%	0.18%	99.80%
Lone Pair	1.86	Ni	-	0.22%	0.87%	98.91%
Lone Pair	1.68	Ni	-	0.14%	3.25%	96.61%
Empty orbital	0.30	Ni	-	29.45%	68.14%	2.39%
Empty orbital	0.04	Ni	-	0.06%	99.28%	0.66%
Empty orbital	0.50	Ge	-	1.00%	98.91%	0.08%
Empty orbital	0.32	Ge	-	6.42%	93.37%	0.20%

Table S24. NBO analysis of the Ni-Ge moiety of **3c**.

Alpha orbitals	Occupation	Atom	Polarization	s-character	p-character	d-character
Bond	1.82	Ni	22.97%	34.44%	64.85%	0.71%
		Ge	77.03%	58.43%	41.34%	0.22%
Bond	1.81	Ni	83.05%	41.85%	58.05%	0.09%
		P	16.95%	34.53%	62.17%	3.29%
Bond	1.95	Ge	27.92%	35.09%	64.73%	0.17%
		C	72.08%	28.37%	71.55%	0.08%
Lone Pair	1.98	Ni	-	0.85%	0.04%	99.11%
Lone Pair	1.96	Ni	-	0.05%	0.22%	99.72%
Lone Pair	1.94	Ni	-	0.01%	0.18%	99.80%
Lone Pair	1.86	Ni	-	0.21%	0.94%	98.84%
Lone Pair	1.70	Ni	-	0.10%	3.08%	96.82%
Empty orbital	0.30	Ni	-	29.81%	67.69%	2.48%
Empty orbital	0.04	Ni	-	0.07%	99.24%	0.68%
Empty orbital	0.48	Ge	-	1.07%	98.84%	0.07%
Empty orbital	0.32	Ge	-	6.14%	93.65%	0.21%

Table S25. NBO analysis of the Ni-Ge moiety of **3d**.

Alpha orbitals	Occupation	Atom	Polarization	s-character	p-character	d-character
Bond	1.82	Ni	23.02%	34.44%	64.84%	0.71%
		Ge	76.98%	58.32%	41.45%	0.23%
Bond	1.81	Ni	16.94%	34.52%	62.20%	3.27%
		P	83.06%	41.95%	57.96%	0.09%
Bond	1.95	Ge	27.93%	35.24%	64.58%	0.17%
		C	72.07%	28.49%	71.43%	0.07%
Lone Pair	1.98	Ni	-	0.85%	0.04%	99.11%
Lone Pair	1.96	Ni	-	0.06%	0.22%	99.72%
Lone Pair	1.94	Ni	-	0.01%	0.19%	99.80%
Lone Pair	1.86	Ni	-	0.21%	0.95%	98.84%
Lone Pair	1.70	Ni	-	0.09%	3.07%	96.83%
Empty orbital	0.30	Ni	-	29.82%	67.68%	2.48%
Empty orbital	0.04	Ni	-	0.07%	99.25%	0.68%
Empty orbital	0.47	Ge	-	1.08%	98.83%	0.07%
Empty orbital	0.32	Ge	-	6.09%	93.69%	0.21%

Table S26. NBO analysis of the Ni-Ge moiety of **3e**.

Alpha orbitals	Occupation	Atom	Polarization	s-character	p-character	d-character
Bond	1.82	Ni	23.10%	34.45%	64.84%	0.71%
		Ge	76.90%	58.31%	41.46%	0.22%
Bond	1.82	Ni	16.94%	34.41%	62.37%	3.22%
		P	83.06%	42.05%	57.85%	0.09%
Bond	1.95	Ge	27.89%	35.29%	64.53%	0.17%
		C	72.11%	28.92%	71.00%	0.08%
Lone Pair	1.97	Ni	-	0.85%	0.04%	99.11%
Lone Pair	1.96	Ni	-	0.06%	0.23%	99.71%
Lone Pair	1.94	Ni	-	0.01%	0.18%	99.80%
Lone Pair	1.85	Ni	-	0.21%	0.97%	98.82%
Lone Pair	1.70	Ni	-	0.08%	3.03%	96.89%
Empty orbital	0.30	Ni	-	29.92%	67.55%	2.51%
Empty orbital	0.04	Ni	-	0.07%	99.23%	0.69%
Empty orbital	0.47	Ge	-	1.07%	98.85%	0.07%
Empty orbital	0.32	Ge	-	6.05%	93.73%	0.21%

Table S27. NBO analysis of the Ni-Ge moiety of **3f**.

Alpha orbitals	Occupation	Atom	Polarization	s-character	p-character	d-character
Bond	1.82	Ni	23.37%	34.54%	64.73%	0.73%
		Ge	76.63%	57.96%	41.81%	0.22%
Bond	1.82	Ni	16.93%	34.21%	62.64%	3.14%
		P	83.07%	42.25%	57.65%	0.09%
Bond	1.95	Ge	28.04%	35.74%	64.09%	0.17%
		C	71.96%	29.03%	70.89%	0.08%
Lone Pair	1.97	Ni	-	0.85%	0.04%	99.11%
Lone Pair	1.96	Ni	-	0.07%	0.24%	99.70%
Lone Pair	1.94	Ni	-	0.01%	0.19%	99.80%
Lone Pair	1.85	Ni	-	0.21%	0.99%	98.79%
Lone Pair	1.71	Ni	-	0.07%	2.98%	96.96%
Empty orbital	0.30	Ni	-	30.04%	67.41%	2.53%
Empty orbital	0.04	Ni	-	0.07%	99.23%	0.70%
Empty orbital	0.47	Ge	-	1.10%	98.82%	0.06%
Empty orbital	0.31	Ge	-	5.93%	93.86%	0.21%

Table S28. NBO analysis of the Ni-Ge moiety of **3g**.

Alpha orbitals	Occupation	Atom	Polarization	s-character	p-character	d-character
Bond	1.89	Ni	21.19%	32.58%	66.80%	0.61%
		Ge	78.81%	82.18%	17.69%	0.13%
Bond	1.84	Ni	16.76%	35.20%	61.99%	2.80%
		P	83.24%	42.83%	57.08%	0.08%
Lone Pair	1.97	Ni	-	0.86%	0.03%	99.11%
Lone Pair	1.95	Ni	-	0.03%	0.22%	99.75%
Lone Pair	1.94	Ni	-	0.00%	0.24%	99.76%
Lone Pair	1.85	Ni	-	0.50%	1.28%	98.21%
Lone Pair	1.75	Ni	-	0.07%	2.41%	97.52%
Empty orbital	0.31	Ni	-	30.77%	66.34%	2.87%
Empty orbital	0.04	Ni	-	0.05%	99.21%	0.75%
Empty orbital	0.49	Ge	-	0.02%	99.82%	0.13%
Empty orbital	0.40	Ge		18.81%	81.08%	0.11%
Empty orbital	0.27	Ge	-	0.04%	99.64%	0.30%

Pathway for H_2 activation

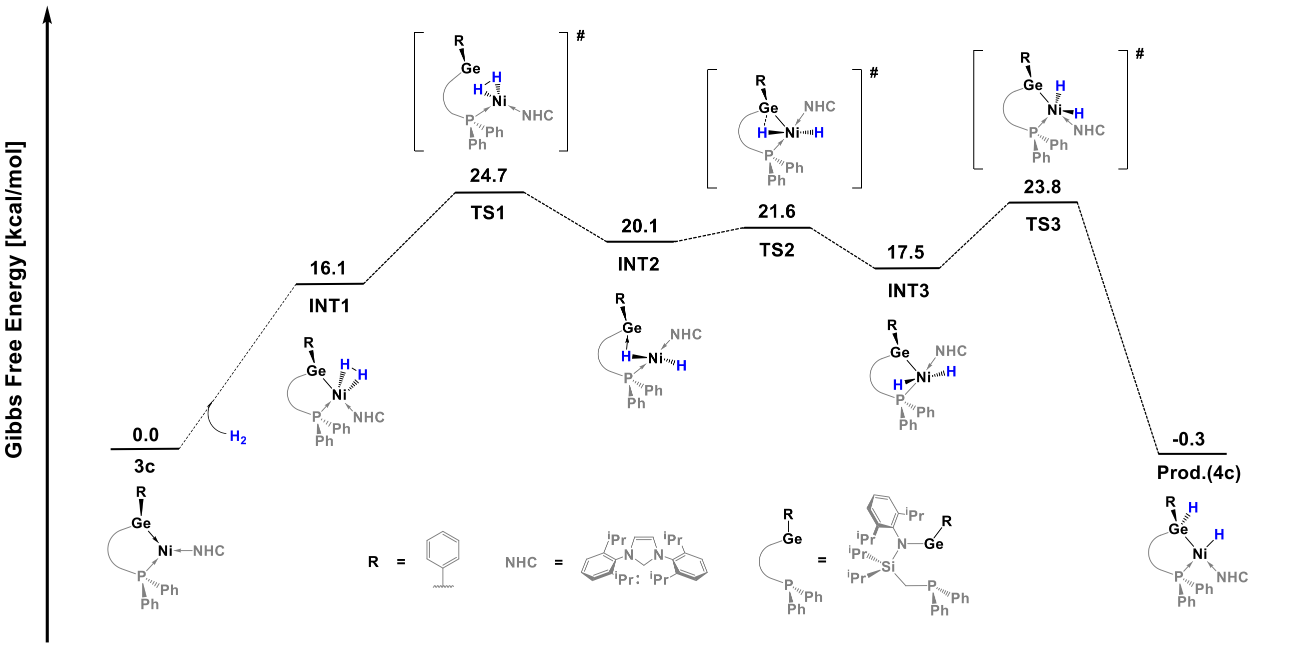

Figure S152. Gibbs free energy profile for the activation of H_2 by complex **3c** at B97-D3/def2-SVP level of theory. TS, INT, and Prod. denote transition state, intermediate and product **4c**.

Table S29. Gibbs free energies for the activation of H_2 by **3a-3g** computed at B97-D3/def2-SVP level of theory. Transition state (TS), intermediate (INT), and product (Prod.) **4a-4g** notations are based on Figure S145.

**ΔG‡ of Geometries [kcal mol⁻¹]**							
**Structures**	**3a**	**3b**	**3c**	**3d**	**3e**	**3f**	**3g**
**INT1**	16.2	16.3	16.1	15.5	15.9	16.8	17.6
**TS1**	23.9	23.2	24.7	24.6	25.3	25.5	23.2
**INT2**	18.9	20.3	20.1	20.9	21.4	22.0	23.4
**TS2**	20.8	22.3	21.6	21.4	21.7	22.2	24.6
**INT3**	17.1	16.1	17.5	17.8	17.7	17.7	18.3
**TS3**	24.6	23.3	23.8	23.5	23.9	23.9	27.6
**Prod.**	0.0	0.8	-0.3	0.3	0.6	1.0	5.2

Pathways related to catalytic dehydrocoupling

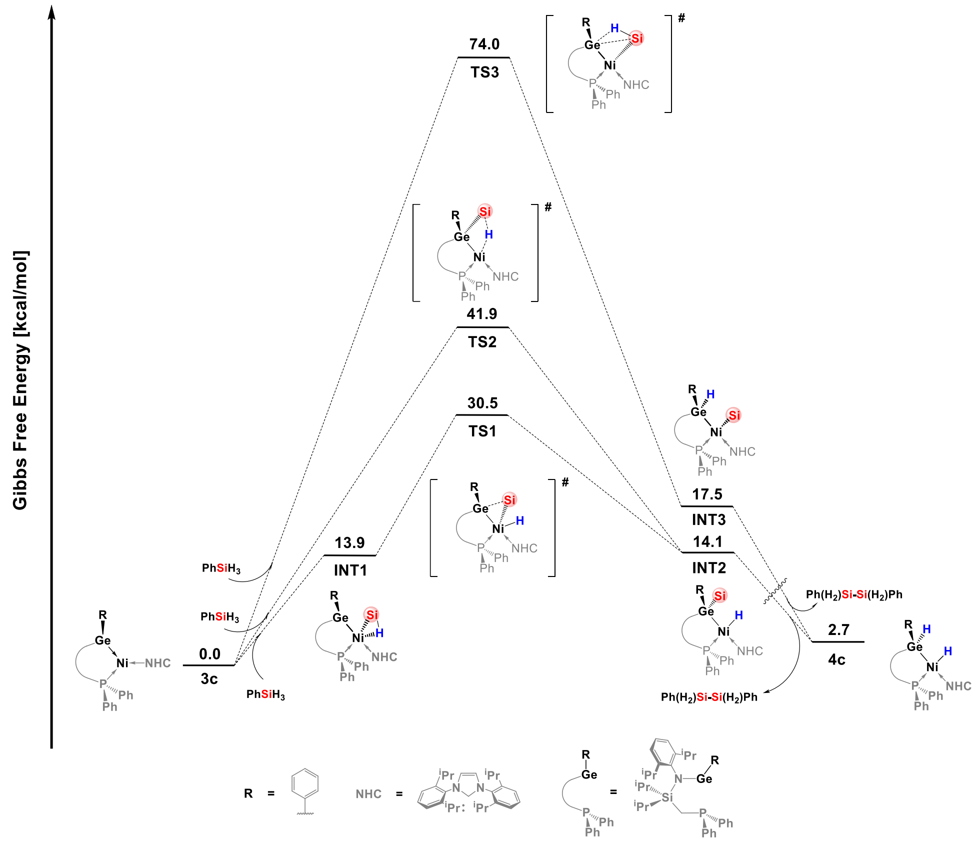

Figure S153. Gibbs free energy profile related to the catalytic dehydrocoupling of $PhSiH_3$ by complex **3c** at B97-D3/def2-SVP level of theory. TS and INT denote transition states and intermediates.

Table S30. Gibbs free energies for the catalytic dehydrocoupling of $PhSiH_3$ by **3a-3g** computed at B97-D3/def2-SVP level of theory. Transition state (TS) and intermediate (INT) notations are based on Figure S146.

ΔG‡ of Geometries [kcal·mol^{-1}]						
**Structures**	**3a**	**3b**	**3c**	**3d**	**3e**	**3f**
**INT1**	14.7	12.3	13.9	13.0	13.5	13.7
**TS1**	31.4	27.2	30.5	30.1	31.0	30.8
**TS2**	42.8	44.6	41.9	41.8	41.6	41.4
**TS3**	74.4	78.1	74.0	74.3	74.4	75.0
**INT2**	14.1	16.6	14.1	14.2	14.3	14.1
**INT3**	17.0	27.1	17.5	17.8	18.0	19.1
**4**	3.0	3.8	2.7	3.4	3.6	4.0

Table S31. Cartesian geometry of 3a in Figure S145 in Angstrom [Å].

Atomtype	X Coordinates	Y Coordinates	Z Coordinates
C	4.204093	-2.560824	-0.642253
C	3.627743	-1.677113	0.326765
C	4.478488	-0.938648	1.206139
C	5.870669	-1.146237	1.136653
C	6.436758	-2.025812	0.206124
C	5.602068	-2.714398	-0.685427
N	2.210818	-1.502566	0.351979
Si	1.142220	-2.364063	1.496609
C	2.132758	-3.562870	2.628400
C	1.205875	-4.218549	3.679277
C	3.938882	0.114443	2.176255
C	4.567314	1.504689	1.933251
C	3.334052	-3.288071	-1.668663
C	3.667886	-4.786211	-1.811211
Ge	1.351311	-0.186990	-0.770325
C	2.851235	1.111675	-1.180866
C	2.485734	2.477634	-1.099390
C	3.393119	3.505043	-1.377697
C	4.712441	3.185052	-1.781498
C	5.087793	1.824449	-1.903417
C	4.168814	0.810224	-1.605056
Ni	-0.802437	0.140527	-0.247117
C	-2.500934	0.349738	-1.086864
N	-3.517473	-0.509110	-1.485491
C	-4.570654	0.186288	-2.096087
C	-4.226634	1.505465	-2.097437
N	-2.972714	1.588777	-1.493728
C	-3.514640	-1.951446	-1.432036
C	-4.512918	-2.607738	-0.666494
C	-4.607379	-4.011492	-0.767339
C	-3.722891	-4.741086	-1.566690
C	-2.716824	-4.075776	-2.281576
C	-2.593461	-2.674575	-2.243774
C	-2.190916	2.796877	-1.414409
C	-2.484955	3.742107	-0.401570
C	-1.731656	4.934258	-0.381648
C	-0.709115	5.156264	-1.311470
C	-0.429029	4.198705	-2.299258
C	-1.170994	3.005911	-2.384961
C	-5.472676	-1.868887	0.268084
C	-6.927938	-1.934936	-0.244652
C	-1.549718	-1.957011	-3.095179
C	-0.311372	-2.803034	-3.427279
C	-3.584673	3.500306	0.628980
C	-4.862278	4.285693	0.258789
C	-0.955943	2.009837	-3.525066
C	-1.972490	2.279317	-4.658969
P	-0.500175	0.345851	1.872351
C	0.362875	-1.087236	2.712251
C	0.451541	1.853978	2.405329
C	1.215956	1.959132	3.586148
C	1.867903	3.160475	3.910169
C	1.765230	4.275182	3.059691
C	1.005622	4.180483	1.882063
C	0.359139	2.978140	1.558949
C	-2.041154	0.545989	2.923572
C	-3.292417	0.405919	2.297689
C	-4.481665	0.609321	3.016909

C	-4.431137	0.945119	4.378900
C	-3.183822	1.069003	5.019111
C	-1.996547	0.871856	4.296554
C	-5.379699	-2.412766	1.710568
C	-2.185091	-1.382524	-4.379350
C	-3.141477	3.825345	2.068702
C	0.472068	1.990697	-4.094915
C	-0.301013	-3.210422	0.549673
C	-1.624690	-3.211142	1.342080
C	0.028048	-4.629386	0.042997
C	4.135898	-0.293159	3.651666
C	3.401293	-2.586762	-3.043496
C	3.005985	-4.632766	1.942075
H	-0.464999	-2.557363	-0.334521
H	-5.447644	-0.328995	-2.484629
H	-4.743412	2.383384	-2.483147
H	-3.311159	0.154706	1.230755
H	-1.147868	1.006943	-3.107132
H	-0.389101	-1.598679	3.338221
H	1.156839	-0.730983	3.391496
H	5.210161	-0.412855	3.888191
H	3.634735	-1.248068	3.888663
H	3.729326	0.485214	4.324331
H	0.112312	-5.344644	0.882877
H	0.973113	-4.674657	-0.526692
H	-0.779792	-4.996709	-0.617968
H	-0.122510	6.083269	-1.269547
H	6.046991	-3.384569	-1.431818
H	2.856376	0.212174	1.990478
H	2.823398	-2.886535	3.171318
H	-1.183208	-1.108403	-2.489838
H	-0.231542	2.900039	0.639090
H	7.524882	-2.164712	0.165865
H	1.319376	1.102832	4.262741
H	6.527241	-0.587623	1.815965
H	4.504678	-0.227760	-1.686305
H	-5.378863	-4.539422	-0.194444
H	-1.031616	0.992013	4.803523
H	-1.935512	5.685084	0.390103
H	2.291770	-3.210067	-1.318231
H	6.107484	1.571045	-2.217092
H	-3.829827	2.425053	0.600981
H	-5.175627	-0.805842	0.295924
H	1.465397	2.752973	-0.811164
H	0.376563	4.386556	-3.016638
H	-3.807500	-5.833623	-1.626562
H	-5.448372	0.515646	2.506880
H	-1.424992	-0.836022	-4.967702
H	-3.003183	-0.677708	-4.150300
H	-2.592942	-2.194043	-5.012229
H	-2.412880	-3.735123	0.773095
H	-1.988557	-2.183872	1.521371
H	-1.527512	-3.717967	2.321785
H	-2.023169	-4.659028	-2.895610
H	3.028774	-1.547361	-2.988625
H	2.786738	-3.126419	-3.788333
H	4.443010	-2.551068	-3.415616
H	-5.358136	1.112654	4.942464
H	-3.136790	1.330296	6.084481
H	4.064975	2.259126	2.564966

H	4.462915	1.815505	0.882332
H	5.644018	1.510668	2.188006
H	2.466602	3.221917	4.828108
H	0.707060	2.916261	-4.653965
H	0.574389	1.148186	-4.803608
H	1.228760	1.864711	-3.304169
H	2.282359	5.209784	3.311628
H	0.916412	5.037699	1.203520
H	-5.686082	-3.473679	1.762032
H	-6.046876	-1.841270	2.380738
H	-4.352355	-2.329529	2.102318
C	5.656128	4.229585	-2.066223
H	-0.532318	-3.609445	-4.152599
H	0.121464	-3.259691	-2.520494
H	0.463162	-2.157431	-3.877959
H	-2.205609	3.303968	2.326614
H	-3.916498	3.494824	2.782970
H	-2.986288	4.910609	2.215628
H	-3.014748	2.207944	-4.304584
H	-1.842356	1.545213	-5.475103
H	-1.819016	3.291865	-5.078402
H	3.558478	-5.218817	2.704430
H	3.751048	-4.185746	1.264477
H	2.398957	-5.349066	1.360563
H	4.664260	-4.945584	-2.264962
H	2.926269	-5.278704	-2.468566
H	3.653636	-5.297561	-0.833378
H	3.090989	4.555280	-1.284688
H	0.477771	-4.903742	3.204888
H	0.632457	-3.478842	4.267797
H	1.798249	-4.822325	4.395870
H	-7.022681	-1.524840	-1.266430
H	-7.600404	-1.360131	0.419900
H	-7.290051	-2.979886	-0.269638
H	-4.666011	5.374860	0.265743
H	-5.668633	4.078616	0.987722
H	-5.230404	4.016224	-0.747825
N	6.425633	5.083097	-2.296765

Table S32. Cartesian geometry of 3b in Figure S145 in Angstrom [Å].

Atomtype	X Coordinates	Y Coordinates	Z Coordinates
C	3.375485	-3.310751	-0.180473
C	2.863138	-2.327896	0.727312
C	3.741352	-1.713788	1.673537
C	5.084646	-2.136282	1.726750
C	5.583360	-3.110941	0.855406
C	4.730648	-3.680161	-0.099505
N	1.495400	-1.928525	0.621592
Si	0.214106	-2.621559	1.657547
C	0.912953	-3.933181	2.880428
C	-0.191439	-4.425400	3.845934
C	3.297430	-0.564847	2.582102
C	4.206429	0.676277	2.434760
C	2.497191	-3.914119	-1.278263
C	2.615151	-5.447414	-1.387653
Ge	0.949740	-0.498874	-0.564982
C	2.707544	0.517707	-0.782829
C	2.684099	1.920125	-0.616156
C	3.852034	2.692716	-0.769210
C	5.068802	2.085206	-1.116109

C	5.099958	0.695593	-1.310437
C	3.938266	-0.076034	-1.144802
Ni	-1.188892	0.104752	-0.260143
C	-2.774857	0.513682	-1.241960
N	-3.845185	-0.216260	-1.743966
C	-4.743702	0.600583	-2.444601
C	-4.246637	1.868723	-2.396073
N	-3.056887	1.800926	-1.672919
C	-4.031251	-1.646916	-1.701563
C	-5.177531	-2.165640	-1.045081
C	-5.441119	-3.545981	-1.166790
C	-4.582835	-4.386878	-1.880631
C	-3.433615	-3.859814	-2.486555
C	-3.135083	-2.486082	-2.424276
C	-2.166270	2.919080	-1.489895
C	-2.483891	3.898745	-0.518197
C	-1.626666	5.013470	-0.408640
C	-0.480481	5.121341	-1.202676
C	-0.173677	4.124395	-2.141755
C	-1.016019	3.012694	-2.322692
C	-6.124700	-1.306020	-0.205241
C	-7.517190	-1.187958	-0.862876
C	-1.927446	-1.912565	-3.159953
C	-0.788748	-2.917532	-3.387404
C	-3.702582	3.770203	0.391404
C	-4.853250	4.679646	-0.092129
C	-0.755022	1.985620	-3.424796
C	-1.598733	2.325680	-4.675027
P	-1.063090	0.296432	1.876408
C	-0.479677	-1.227690	2.793091
C	0.028694	1.675915	2.476236
C	0.737458	1.680588	3.695111
C	1.527699	2.784836	4.057149
C	1.612348	3.903357	3.211218
C	0.901221	3.912750	1.999926
C	0.124063	2.804352	1.633368
C	-2.650224	0.705003	2.785756
C	-3.857942	0.672382	2.066390
C	-5.070632	1.016204	2.686289
C	-5.088428	1.387818	4.039839
C	-3.886491	1.409305	4.771731
C	-2.674742	1.071479	4.148911
C	-6.250229	-1.851787	1.234168
C	-2.351101	-1.251154	-4.488715
C	-3.367764	4.051305	1.869665
C	0.725852	1.837717	-3.811100
C	-1.240963	-3.273056	0.583109
C	-2.617734	-3.070146	1.248706
C	-1.076532	-4.735206	0.120737
C	3.236173	-0.979981	4.067401
C	2.785512	-3.254062	-2.645658
C	1.676938	-5.134365	2.287149
H	-1.227535	-2.622059	-0.317578
H	-5.632822	0.193663	-2.923387
H	-4.612959	2.802631	-2.820481
H	-3.822814	0.393714	1.006607
H	-1.082142	1.007922	-3.030182
H	-1.344633	-1.617315	3.357527
H	0.297703	-0.971865	3.533537
H	4.226182	-1.328589	4.417717

H	2.514557	-1.796701	4.242593
H	2.940594	-0.119583	4.696719
H	-1.175612	-5.436442	0.970652
H	-0.100247	-4.929523	-0.356817
H	-1.865118	-4.995655	-0.610347
H	0.192781	5.978898	-1.084904
H	5.130052	-4.419630	-0.804630
H	2.282546	-0.266517	2.268620
H	1.644840	-3.356096	3.480265
H	-1.510671	-1.122816	-2.508894
H	-0.411662	2.800639	0.676561
H	6.638957	-3.406416	0.901827
H	0.690916	0.819020	4.371026
H	5.763100	-1.666991	2.449985
H	4.019471	-1.154859	-1.290932
H	-6.327391	-3.967707	-0.678156
H	-1.741936	1.112831	4.724534
H	-1.847577	5.789481	0.332945
H	1.452318	-3.675978	-1.020258
C	6.419076	0.011752	-1.607597
H	-4.049235	2.724428	0.331033
H	-5.698963	-0.289411	-0.137580
H	1.750681	2.429913	-0.352592
H	0.737333	4.219254	-2.739778
H	-4.800942	-5.459639	-1.958076
H	-6.000618	1.006020	2.104923
H	-1.469447	-0.813714	-4.992484
H	-3.082055	-0.440114	-4.326913
H	-2.803635	-1.996044	-5.171249
H	-3.416844	-3.493258	0.614831
H	-2.847772	-1.998481	1.381950
H	-2.682458	-3.561579	2.238789
H	-2.763196	-4.530444	-3.033375
H	2.512951	-2.182609	-2.645263
H	2.202022	-3.744484	-3.447138
H	3.859315	-3.327461	-2.901546
H	-6.033329	1.664874	4.525004
H	-3.893005	1.700543	5.830324
H	3.742416	1.546630	2.932978
H	4.368484	0.935869	1.378240
H	5.197385	0.508025	2.897188
H	2.086249	2.764784	5.001924
H	1.102231	2.725550	-4.353806
H	0.848622	0.967731	-4.482339
H	1.365793	1.677805	-2.928048
H	2.243705	4.757469	3.485417
H	0.969682	4.769525	1.321012
H	-6.686454	-2.867441	1.243851
H	-6.911239	-1.201746	1.835421
H	-5.267669	-1.892267	1.732710
H	5.975392	2.685361	-1.239041
H	-1.045871	-3.683095	-4.144376
H	-0.510623	-3.433172	-2.451963
H	0.105342	-2.381713	-3.751667
H	-2.509482	3.446889	2.205731
H	-4.231299	3.791487	2.507203
H	-3.129275	5.117748	2.040202
H	-2.679220	2.337465	-4.451515
H	-1.426862	1.579005	-5.471763
H	-1.316559	3.320746	-5.068786

H	2.062850	-5.778660	3.103161
H	2.540044	-4.816789	1.680537
H	1.027000	-5.766069	1.656269
H	3.607904	-5.758421	-1.763874
H	1.859873	-5.835570	-2.097255
H	2.452955	-5.935650	-0.411457
C	3.824169	4.176597	-0.473282
H	-0.967381	-5.005283	3.310855
H	-0.699576	-3.599181	4.376371
H	0.238926	-5.096198	4.616507
H	-7.455467	-0.775303	-1.886056
H	-8.174169	-0.527868	-0.265495
H	-8.005513	-2.178157	-0.931445
H	-4.549418	5.743336	-0.058949
H	-5.741798	4.557582	0.556073
H	-5.150402	4.446402	-1.130701
F	6.253230	-1.139263	-2.303461
F	7.250296	0.807236	-2.328798
F	7.078361	-0.307616	-0.462181
F	4.834060	4.843343	-1.081998
F	2.659818	4.757471	-0.876693
F	3.936421	4.423204	0.860055

Table S33. Cartesian geometry of 3c in Figure S145 in Angstrom [Å].

Atomtype	X Coordinates	Y Coordinates	Z Coordinates
C	1.097627	2.747138	3.153701
C	0.328631	2.340762	2.042993
C	0.030206	3.290933	1.044955
C	0.475937	4.616602	1.153081
C	1.238304	5.012333	2.264264
C	1.548561	4.072905	3.263263
P	-0.373804	0.636192	1.785016
C	-1.873108	0.722052	2.911676
C	-3.100738	0.259067	2.405050
C	-4.275563	0.355563	3.168899
C	-4.232443	0.908279	4.458646
C	-3.005372	1.355987	4.982645
C	-1.833776	1.264309	4.214555
Ni	-0.724903	0.079495	-0.263476
C	-2.460128	-0.142570	-1.007408
N	-3.323842	-1.212905	-1.213446
C	-4.513068	-0.804934	-1.835221
C	-4.413176	0.539094	-2.039127
N	-3.168228	0.924421	-1.543670
C	-3.057709	-2.609878	-0.971424
C	-3.886646	-3.314772	-0.060417
C	-3.730232	-4.713557	0.031269
C	-2.763053	-5.384733	-0.722273
C	-1.925585	-4.663019	-1.584588
C	-2.056849	-3.270769	-1.741147
C	-2.612107	2.245855	-1.686442
C	-3.006402	3.260992	-0.780824
C	-2.476390	4.553065	-0.976003
C	-1.569018	4.810751	-2.010508
C	-1.184635	3.785136	-2.888656
C	-1.705642	2.484501	-2.757274
C	-4.921694	-2.628107	0.832840
C	-4.666208	-2.935932	2.324566
C	-1.198960	-2.511563	-2.749337
C	-1.986909	-2.238802	-4.048515

C	-3.980856	2.981753	0.360084
C	-3.534665	3.598094	1.699866
C	-1.380773	1.385329	-3.768953
C	-0.000781	1.517254	-4.434073
Ge	1.426065	0.084559	-0.894339
N	2.555687	-0.907327	0.322465
Si	1.716416	-1.769839	1.643761
C	0.412812	-2.985246	0.923030
C	0.973215	-4.383223	0.591248
C	2.647603	1.523362	-1.614554
C	2.049067	2.802623	-1.715767
C	2.755351	3.908814	-2.214999
C	4.081508	3.753228	-2.653289
C	4.685601	2.485405	-2.589503
C	3.979383	1.384486	-2.074840
C	3.978341	-0.844022	0.225749
C	4.722668	0.144656	0.940090
C	6.125583	0.163904	0.809415
C	6.797733	-0.734831	-0.027666
C	6.059932	-1.674065	-0.762068
C	4.659552	-1.749982	-0.649994
C	4.046555	1.222920	1.789372
C	4.385378	1.087775	3.288805
C	3.886500	-2.751136	-1.509713
C	3.766946	-2.250848	-2.966355
C	4.394409	2.646596	1.300802
C	4.471869	-4.176696	-1.476026
C	-6.362527	-3.018447	0.437048
C	0.152952	-3.172232	-3.057927
C	-5.405364	3.451099	-0.009537
C	-2.484340	1.319369	-4.850199
C	0.771748	-0.496283	2.739901
C	2.961481	-2.602400	2.853732
C	3.982323	-3.594820	2.261320
C	2.218495	-3.247187	4.047733
C	-0.852770	-3.104143	1.796975
H	0.094563	-2.504741	-0.027282
H	-5.299420	-1.514479	-2.087713
H	-5.097418	1.248713	-2.502638
H	-3.116628	-0.156521	1.390319
H	-1.376563	0.435318	-3.208453
H	0.153012	-1.043378	3.472451
H	1.514902	0.082051	3.316287
H	5.472962	1.198238	3.460892
H	4.082196	0.106762	3.695471
H	3.871911	1.875390	3.871717
H	1.222379	-4.943312	1.512417
H	1.885324	-4.345338	-0.030085
H	0.218312	-4.978077	0.042746
H	-1.153337	5.819182	-2.135523
H	6.583817	-2.361693	-1.438117
H	2.956835	1.100745	1.674659
H	3.542194	-1.743957	3.247904
H	-0.961278	-1.536746	-2.285593
H	-0.560517	2.975449	0.177416
H	7.891057	-0.695538	-0.118879
H	1.361995	2.031839	3.941397
H	6.701715	0.916992	1.362453
H	4.491297	0.419400	-2.017978
H	-4.368143	-5.280444	0.719717

H	-0.889238	1.636819	4.628484
H	-2.760725	5.359149	-0.290263
H	2.863539	-2.799875	-1.101722
H	5.720971	2.351733	-2.930584
H	-4.012000	1.888293	0.501572
H	-4.821719	-1.536765	0.698468
H	1.009083	2.944983	-1.401275
H	-0.467341	4.001373	-3.687128
H	-2.650685	-6.472666	-0.631412
H	-5.227590	0.008408	2.748689
H	-1.365780	-1.657150	-4.754571
H	-2.906491	-1.659858	-3.854634
H	-2.271800	-3.188304	-4.541450
H	-1.554779	-3.835692	1.359330
H	-1.390704	-2.141718	1.859903
H	-0.622266	-3.438480	2.827236
H	-1.164391	-5.198087	-2.161376
H	3.217481	-1.293624	-3.023760
H	3.228134	-2.988881	-3.589914
H	4.768454	-2.089645	-3.409101
H	-5.149922	0.993377	5.055403
H	-2.963785	1.788160	5.991196
H	3.795935	3.390245	1.857387
H	4.178831	2.766985	0.227848
H	5.464222	2.877448	1.465883
H	2.153675	4.370208	4.129552
H	0.044715	2.380305	-5.125308
H	0.207003	0.610742	-5.032350
H	0.804970	1.628439	-3.691019
H	1.598913	6.045499	2.349049
H	0.229816	5.332602	0.359387
H	-4.769511	-4.015934	2.536836
H	-5.396502	-2.401265	2.958439
H	-3.655195	-2.618444	2.629195
H	4.640029	4.613238	-3.046162
H	0.045191	-4.097530	-3.655974
H	0.703823	-3.417563	-2.133567
H	0.776941	-2.473040	-3.642325
H	-2.498786	3.314236	1.945409
H	-4.184477	3.232818	2.515012
H	-3.597767	4.702458	1.685397
H	-3.480089	1.133751	-4.413074
H	-2.272887	0.504290	-5.566542
H	-2.526793	2.271152	-5.413521
H	4.667083	-3.955434	3.055848
H	4.601087	-3.131638	1.476261
H	3.489990	-4.484617	1.830467
H	5.458741	-4.229420	-1.973623
H	3.798916	-4.875276	-2.008864
H	4.593241	-4.538871	-0.440634
H	2.266587	4.891657	-2.262406
H	1.602208	-4.107146	3.722948
H	1.551367	-2.539036	4.572797
H	2.942989	-3.632262	4.793489
H	-6.576840	-2.780781	-0.620683
H	-7.097393	-2.480577	1.065569
H	-6.529283	-4.103220	0.575764
H	-5.424691	4.545864	-0.171441
H	-6.114854	3.212765	0.805605
H	-5.768383	2.966966	-0.934180

Table S34. Cartesian geometry of 3d in Figure S145 in Angstrom [Å].

Atomtype	X Coordinates	Y Coordinates	Z Coordinates
C	4.387423	-2.264229	-0.630535
C	3.771100	-1.373466	0.306794
C	4.587102	-0.544589	1.137053
C	5.988081	-0.671121	1.055325
C	6.594333	-1.558062	0.157531
C	5.791329	-2.335763	-0.688740
N	2.347301	-1.285890	0.350977
Si	1.360960	-2.165248	1.553262
C	2.450860	-3.242016	2.718954
C	1.589254	-3.910812	3.816006
C	3.995966	0.519704	2.063895
C	4.520951	1.934166	1.731387
C	3.548884	-3.087862	-1.609112
C	3.971373	-4.568007	-1.691194
Ge	1.387283	-0.063512	-0.801762
C	2.788481	1.286649	-1.328145
C	2.347208	2.630592	-1.312124
C	3.198259	3.691519	-1.658302
C	4.526800	3.450105	-2.062113
C	4.963105	2.108834	-2.116368
C	4.117414	1.049025	-1.755121
Ni	-0.777648	0.118692	-0.247937
C	-2.490787	0.167687	-1.069382
N	-3.456873	-0.773834	-1.408358
C	-4.565368	-0.177004	-2.026715
C	-4.309762	1.160146	-2.094315
N	-3.053066	1.353680	-1.522200
C	-3.357007	-2.208404	-1.294210
C	-4.298749	-2.896114	-0.485325
C	-4.300460	-4.306067	-0.524023
C	-3.379193	-5.009983	-1.304683
C	-2.429533	-4.311210	-2.063394
C	-2.400399	-2.904571	-2.088798
C	-2.351647	2.612058	-1.512679
C	-2.679836	3.576206	-0.528338
C	-2.005836	4.813992	-0.575008
C	-1.024940	5.064044	-1.541947
C	-0.708520	4.087966	-2.500009
C	-1.372232	2.847182	-2.518166
C	-5.292228	-2.182458	0.433655
C	-6.747546	-2.367783	-0.048852
C	-1.418834	-2.157507	-2.987007
C	-0.135873	-2.936941	-3.312237
C	-3.733187	3.302800	0.541759
C	-5.075644	3.971456	0.171874
C	-1.116698	1.818349	-3.619720
C	-2.172247	1.970319	-4.739614
P	-0.458523	0.437445	1.855027
C	0.516249	-0.898130	2.734822
C	0.408236	2.023094	2.303822
C	1.170847	2.233361	3.471847
C	1.758128	3.483781	3.725927
C	1.592358	4.542893	2.816239
C	0.836947	4.342704	1.649263
C	0.255091	3.091526	1.396566
C	-1.987773	0.580936	2.934678
C	-3.235985	0.318760	2.342236

C	-4.425083	0.472981	3.073880
C	-4.377029	0.881358	4.416136
C	-3.131719	1.126893	5.024193
C	-1.945179	0.978572	4.288625
C	-5.141169	-2.654869	1.896246
C	-2.111330	-1.674269	-4.279276
C	-3.283116	3.727478	1.952915
C	0.297552	1.865692	-4.221460
C	-0.036178	-3.152206	0.675437
C	-1.343401	-3.207136	1.492736
C	0.381463	-4.567704	0.227836
C	4.248141	0.206116	3.553654
C	3.552828	-2.446395	-3.014454
C	3.385636	-4.280753	2.067009
H	-0.258534	-2.552409	-0.233456
H	-5.412905	-0.765748	-2.373989
H	-4.890915	1.983209	-2.508353
H	-3.253177	0.012829	1.289236
H	-1.234082	0.825219	-3.153861
H	-0.187884	-1.435465	3.393798
H	1.293967	-0.460999	3.385389
H	5.332201	0.175568	3.774083
H	3.821036	-0.768220	3.850036
H	3.797575	0.988171	4.193317
H	0.530681	-5.234796	1.097927
H	1.316721	-4.575310	-0.359390
H	-0.410835	-5.020936	-0.397856
H	-0.497848	6.027091	-1.550821
H	6.266155	-3.011885	-1.411096
H	2.905975	0.534233	1.898793
H	3.100711	-2.494325	3.217150
H	-1.095947	-1.264595	-2.421035
H	-0.330029	2.928645	0.484274
H	7.688382	-1.633802	0.106703
H	1.323651	1.421095	4.192140
H	6.618016	-0.042212	1.697673
H	4.518308	0.031869	-1.792860
H	-5.027113	-4.858308	0.083646
H	-0.983712	1.193810	4.769914
H	-2.236610	5.579498	0.174440
H	2.508988	-3.056808	-1.244541
H	5.992061	1.889892	-2.435138
H	-3.895684	2.212080	0.568437
H	-5.066998	-1.101861	0.410267
H	1.315686	2.864183	-1.025491
H	0.067510	4.296321	-3.243740
H	-3.390692	-6.107364	-1.315591
H	-5.390648	0.284274	2.588517
H	-1.397089	-1.105642	-4.903106
H	-2.967445	-1.012510	-4.061193
H	-2.478969	-2.533621	-4.872641
H	-2.103551	-3.807584	0.962326
H	-1.772815	-2.199593	1.634925
H	-1.195454	-3.662687	2.491172
H	-1.705998	-4.873519	-2.662307
H	3.116894	-1.430928	-2.999059
H	2.964183	-3.057370	-3.724456
H	4.585289	-2.362139	-3.404311
H	-5.304555	1.011021	4.988879
H	-3.086409	1.445324	6.074077

H	3.981412	2.685236	2.336244
H	4.371081	2.178440	0.668341
H	5.600527	2.025199	1.958304
H	2.356173	3.627507	4.635174
H	0.458055	2.777225	-4.828291
H	0.440317	0.999052	-4.893341
H	1.077400	1.827624	-3.444103
H	2.059125	5.516460	3.013762
H	0.702512	5.154066	0.923477
H	-5.374289	-3.730647	1.998662
H	-5.835207	-2.100374	2.553385
H	-4.115977	-2.486036	2.265277
C	5.468219	4.584847	-2.402730
H	-0.317773	-3.782218	-4.003583
H	0.340393	-3.331600	-2.398205
H	0.588726	-2.261439	-3.800523
H	-2.300688	3.297376	2.205665
H	-4.009674	3.366389	2.702484
H	-3.215682	4.827484	2.048925
H	-3.200037	1.847564	-4.358039
H	-2.011208	1.210110	-5.525935
H	-2.094538	2.970869	-5.206435
H	3.991493	-4.788796	2.844879
H	4.084878	-3.816953	1.353013
H	2.820493	-5.065578	1.533594
H	4.968906	-4.687144	-2.154936
H	3.251524	-5.133708	-2.312981
H	4.003142	-5.034615	-0.691599
H	2.822028	4.723730	-1.614933
H	0.901872	-4.664385	3.386530
H	0.976395	-3.186428	4.383333
H	2.233678	-4.440610	4.546339
H	-6.885987	-2.009182	-1.084921
H	-7.446976	-1.811231	0.603415
H	-7.038469	-3.434798	-0.023330
H	-4.962531	5.071462	0.123844
H	-5.845912	3.739285	0.931763
H	-5.446777	3.627598	-0.810605
H	6.091280	4.343982	-3.284680
H	4.921678	5.522780	-2.610655
H	6.163731	4.785553	-1.562714

Table S35. Cartesian geometry of 3e in Figure S145 in Angstrom [Å].

Atomtype	X Coordinates	Y Coordinates	Z Coordinates
C	4.044858	-2.731492	-0.568424
C	3.479946	-1.842352	0.402541
C	4.337210	-1.163591	1.322906
C	5.719557	-1.434825	1.290442
C	6.272711	-2.321369	0.358480
C	5.434587	-2.950938	-0.572830
N	2.073933	-1.602188	0.391483
Si	0.943056	-2.441436	1.491531
C	1.858957	-3.703853	2.620590
C	0.884083	-4.339133	3.639963
C	3.816683	-0.104167	2.296175
C	4.491444	1.267350	2.072359
C	3.173674	-3.391154	-1.638377
C	3.436627	-4.900728	-1.805677
Ge	1.306830	-0.206557	-0.709544
C	2.863514	1.002277	-1.110807

C	2.562346	2.388584	-1.071347
C	3.525861	3.367152	-1.328690
C	4.844572	2.987525	-1.665659
C	5.165209	1.615369	-1.746878
C	4.183553	0.647320	-1.469130
Ni	-0.849018	0.173978	-0.224905
C	-2.502487	0.460181	-1.114573
N	-3.545936	-0.344574	-1.560570
C	-4.550011	0.409962	-2.185165
C	-4.148201	1.711957	-2.149001
N	-2.909924	1.727904	-1.508549
C	-3.604817	-1.785481	-1.545863
C	-4.651344	-2.419217	-0.826782
C	-4.800627	-3.814776	-0.966915
C	-3.922825	-4.560837	-1.758545
C	-2.869742	-3.920269	-2.426847
C	-2.691128	-2.526613	-2.350126
C	-2.071611	2.893722	-1.390665
C	-2.314736	3.814680	-0.342413
C	-1.503035	4.965973	-0.282324
C	-0.476177	5.172555	-1.211631
C	-0.247676	4.239564	-2.235352
C	-1.044622	3.085905	-2.356400
C	-5.606522	-1.664023	0.099465
C	-7.047459	-1.654663	-0.456295
C	-1.597219	-1.831267	-3.155536
C	-0.384454	-2.717751	-3.476164
C	-3.423331	3.583281	0.680341
C	-4.689279	4.386371	0.308009
C	-0.873616	2.112721	-3.522957
C	-1.847399	2.481288	-4.666211
P	-0.594699	0.322884	1.906012
C	0.181043	-1.171974	2.726646
C	0.432301	1.760873	2.492801
C	1.134847	1.817203	3.714931
C	1.849517	2.971559	4.075087
C	1.876120	4.085524	3.217498
C	1.185751	4.036601	1.995441
C	0.473878	2.881776	1.638494
C	-2.146951	0.574908	2.932629
C	-3.385828	0.459212	2.276317
C	-4.587943	0.700632	2.961594
C	-4.564411	1.050160	4.321142
C	-3.331446	1.147695	4.992446
C	-2.131046	0.912416	4.303278
C	-5.579911	-2.248064	1.528895
C	-2.175918	-1.203787	-4.441926
C	-2.989809	3.892049	2.126044
C	0.561740	2.013140	-4.065914
C	-0.506392	-3.209675	0.490049
C	-1.850709	-3.175608	1.245570
C	-0.219875	-4.629177	-0.040096
C	3.975218	-0.531550	3.770361
C	3.320054	-2.663751	-2.993359
C	2.702091	-4.797957	1.935174
H	-0.618081	-2.529225	-0.381551
H	-5.437140	-0.057602	-2.609431
H	-4.614932	2.618882	-2.531399
H	-3.382322	0.199638	1.210560
H	-1.141065	1.113596	-3.140161

H	-0.611984	-1.672239	3.309732
H	0.964869	-0.870937	3.443911
H	5.041164	-0.680092	4.028113
H	3.445823	-1.476365	3.986791
H	3.573227	0.249804	4.442363
H	-0.186748	-5.365149	0.785528
H	0.737806	-4.700048	-0.585454
H	-1.023280	-4.949781	-0.730266
H	0.156671	6.066701	-1.138595
H	5.870176	-3.626201	-1.320332
H	2.741319	0.030868	2.094922
H	2.565066	-3.065661	3.189588
H	-1.214365	-1.011993	-2.519926
H	-0.064697	2.837134	0.685030
H	7.353881	-2.512053	0.348575
H	1.140676	0.957165	4.395033
H	6.379740	-0.921309	2.001454
H	4.484889	-0.403021	-1.519740
H	-5.609925	-4.324114	-0.430364
H	-1.178152	1.013741	4.835736
H	-1.663738	5.694985	0.519833
H	2.125963	-3.267410	-1.318583
H	6.175177	1.283864	-2.009193
H	-3.680662	2.511547	0.641522
H	-5.265789	-0.615690	0.163759
H	1.545240	2.720166	-0.833996
H	0.566175	4.410862	-2.947549
H	-4.049847	-5.647237	-1.848633
H	-5.543084	0.627223	2.427118
H	-1.380989	-0.671450	-4.996459
H	-2.974358	-0.475390	-4.217181
H	-2.595289	-1.984705	-5.105296
H	-2.643248	-3.652074	0.641801
H	-2.176363	-2.138681	1.441303
H	-1.801815	-3.710253	2.214148
H	-2.182264	-4.516414	-3.035540
H	3.003020	-1.607383	-2.923905
H	2.701461	-3.153919	-3.768591
H	4.373474	-2.676149	-3.333090
H	-5.501165	1.248715	4.858008
H	-3.305200	1.419228	6.056075
H	4.013124	2.029606	2.713211
H	4.397981	1.593448	1.024839
H	5.567596	1.232170	2.329222
H	2.397230	2.996017	5.026130
H	0.873780	2.939923	-4.583962
H	0.618714	1.192801	-4.805386
H	1.290304	1.803648	-3.266347
H	2.442888	4.982672	3.497764
H	1.202646	4.891559	1.308508
H	-5.928617	-3.297022	1.542519
H	-6.245233	-1.668431	2.194010
H	-4.562771	-2.215980	1.953407
O	5.721177	4.007695	-1.898594
H	-0.616124	-3.496425	-4.228190
H	0.004115	-3.213976	-2.570165
H	0.427368	-2.092396	-3.888224
H	-2.049156	3.378111	2.380878
H	-3.764842	3.542541	2.831281
H	-2.847555	4.977022	2.289445

H	-2.898057	2.478876	-4.329546
H	-1.757034	1.757425	-5.497102
H	-1.613716	3.489147	-5.059488
H	3.216919	-5.416068	2.698841
H	3.476394	-4.371930	1.277295
H	2.078008	-5.481688	1.332857
H	4.438906	-5.100312	-2.229921
H	2.694213	-5.340222	-2.498836
H	3.363352	-5.432602	-0.841493
H	3.284038	4.435860	-1.277312
H	0.138243	-4.984651	3.137913
H	0.329500	-3.586976	4.230571
H	1.435560	-4.980482	4.356875
H	-7.094299	-1.214507	-1.468819
H	-7.714932	-1.069051	0.203958
H	-7.451954	-2.682368	-0.520048
H	-4.478606	5.472936	0.321180
H	-5.501094	4.185677	1.032838
H	-5.057051	4.125751	-0.700779
C	7.064150	3.693051	-2.227059
H	7.588925	4.654632	-2.360521
H	7.562572	3.117189	-1.418762
H	7.133812	3.109265	-3.169342

Table S36. Cartesian geometry of 3f in Figure S145 in Angstrom [Å].

Atomtype	X Coordinates	Y Coordinates	Z Coordinates
C	3.632708	-3.207534	-0.624087
C	3.149777	-2.312479	0.384903
C	4.057113	-1.785993	1.355695
C	5.399478	-2.213577	1.333239
C	5.869577	-3.108357	0.364230
C	4.987712	-3.586055	-0.615624
N	1.780952	-1.912823	0.363610
Si	0.538777	-2.668597	1.400768
C	1.280947	-4.082593	2.477047
C	0.219007	-4.653408	3.446323
C	3.638802	-0.724003	2.374742
C	4.467855	0.571579	2.230869
C	2.718199	-3.703546	-1.744849
C	2.803765	-5.223797	-1.984844
Ge	1.207073	-0.379254	-0.673147
C	2.891574	0.652480	-0.993356
C	2.750537	2.057827	-0.897076
C	3.819685	2.941471	-1.075906
C	5.120524	2.454755	-1.396206
C	5.263714	1.042544	-1.534055
C	4.181576	0.177987	-1.334727
Ni	-0.905844	0.213531	-0.209641
C	-2.487131	0.729878	-1.119912
N	-3.607597	0.074701	-1.622323
C	-4.502192	0.969878	-2.227706
C	-3.951805	2.212406	-2.123195
N	-2.734462	2.053579	-1.462151
C	-3.833530	-1.348267	-1.677523
C	-4.962994	-1.887312	-1.008349
C	-5.269822	-3.248076	-1.217009
C	-4.466102	-4.055276	-2.027215
C	-3.330230	-3.512781	-2.644837
C	-2.992875	-2.154498	-2.498754
C	-1.769850	3.106284	-1.272609

C	-1.929746	3.999775	-0.185129
C	-0.992603	5.044974	-0.053938
C	0.072346	5.174335	-0.953807
C	0.215630	4.270175	-2.018049
C	-0.705069	3.223071	-2.209330
C	-5.844895	-1.067608	-0.064530
C	-7.261204	-0.862138	-0.644987
C	-1.805616	-1.556705	-3.248095
C	-0.698228	-2.564657	-3.590542
C	-3.083098	3.853736	0.803432
C	-4.232079	4.824069	0.450504
C	-0.617567	2.289496	-3.416734
C	-1.520360	2.813480	-4.557596
P	-0.686773	0.227186	1.931100
C	-0.102856	-1.384684	2.687773
C	0.485102	1.507534	2.607256
C	1.159936	1.425428	3.843509
C	1.995672	2.469922	4.271981
C	2.173602	3.610462	3.469109
C	1.512629	3.698232	2.233131
C	0.679143	2.652998	1.808254
C	-2.221894	0.606117	2.944715
C	-3.448204	0.678789	2.259367
C	-4.629875	1.026681	2.934288
C	-4.599486	1.295444	4.311991
C	-3.381582	1.204640	5.011310
C	-2.200871	0.862780	4.332691
C	-5.921042	-1.715374	1.335297
C	-2.274768	-0.804267	-4.511852
C	-2.651264	4.033054	2.271263
C	0.809038	2.055346	-3.941173
C	-0.967862	-3.217306	0.340023
C	-2.314957	-3.071752	1.077247
C	-0.829479	-4.631444	-0.259100
C	3.717895	-1.238134	3.827439
C	2.986798	-2.933106	-3.056740
C	2.009659	-5.229535	1.748139
H	-0.986377	-2.485720	-0.496419
H	-5.427910	0.629862	-2.689275
H	-4.300777	3.184293	-2.469940
H	-3.447062	0.479915	1.180491
H	-1.000849	1.310314	-3.083746
H	-0.960843	-1.820299	3.229377
H	0.692331	-1.210633	3.434016
H	4.755019	-1.517823	4.093948
H	3.082055	-2.126888	3.987488
H	3.391310	-0.450302	4.532097
H	-0.895893	-5.407088	0.527366
H	0.126003	-4.781280	-0.792179
H	-1.648442	-4.825294	-0.977700
H	0.801736	5.984987	-0.825560
H	5.361098	-4.266219	-1.391872
H	2.589867	-0.457806	2.164839
H	2.042286	-3.558584	3.089796
H	-1.344587	-0.819879	-2.564990
H	0.162348	2.715887	0.843898
H	6.921681	-3.422248	0.363232
H	1.049477	0.540631	4.481666
H	6.097097	-1.817198	2.082499
H	4.372468	-0.894748	-1.431840

H	-6.145004	-3.682822	-0.719613
H	-1.256769	0.816915	4.888137
H	-1.087541	5.749618	0.779899
H	1.684662	-3.470532	-1.440378
H	6.234020	0.602721	-1.783419
H	-3.467838	2.825084	0.702595
H	-5.384594	-0.071242	0.056037
H	1.770079	2.492573	-0.671194
H	1.060951	4.377719	-2.705672
H	-4.716034	-5.114239	-2.171038
H	-5.572758	1.099517	2.378246
H	-1.409038	-0.346102	-5.025097
H	-2.985488	0.003715	-4.265673
H	-2.768576	-1.496942	-5.220519
H	-3.143496	-3.424898	0.437976
H	-2.527160	-2.016345	1.323535
H	-2.344940	-3.657654	2.016581
H	-2.702084	-4.157195	-3.268298
H	2.796044	-1.850964	-2.939368
H	2.336014	-3.307443	-3.869398
H	4.040180	-3.055049	-3.374222
H	-5.519052	1.576786	4.841595
H	-3.350547	1.411710	6.089260
H	4.068262	1.349252	2.906317
H	4.428960	0.958861	1.200897
H	5.528772	0.400405	2.496736
H	2.519054	2.386490	5.233354
H	1.230805	2.963558	-4.412603
H	0.790166	1.266580	-4.716130
H	1.494074	1.731945	-3.141090
H	2.834314	4.420904	3.803061
H	1.645836	4.575130	1.587705
H	-6.393331	-2.714016	1.292211
H	-6.526767	-1.090579	2.016309
H	-4.917246	-1.826068	1.777868
N	6.195708	3.314656	-1.569661
H	-1.001130	-3.272852	-4.385767
H	-0.393479	-3.147049	-2.704075
H	0.191734	-2.020835	-3.953987
H	-1.791014	3.390030	2.516855
H	-3.482314	3.751762	2.942432
H	-2.375928	5.081314	2.493722
H	-2.572452	2.910028	-4.240061
H	-1.490022	2.122575	-5.420257
H	-1.170299	3.806674	-4.898536
H	2.436754	-5.939166	2.485988
H	2.839531	-4.860776	1.124384
H	1.325281	-5.807697	1.102162
H	3.786489	-5.521549	-2.397249
H	2.033458	-5.536300	-2.715500
H	2.641388	-5.789505	-1.051279
H	3.631548	4.013848	-0.963886
H	-0.585554	-5.180612	2.898895
H	-0.257657	-3.874350	4.069284
H	0.678147	-5.391300	4.135006
H	-7.232258	-0.372867	-1.635404
H	-7.870903	-0.233033	0.031013
H	-7.781700	-1.830818	-0.766105
H	-3.891364	5.874497	0.526545
H	-5.080986	4.687619	1.147260

H	-4.602070	4.662746	-0.578098
C	7.515481	2.778371	-1.854826
C	6.019098	4.742032	-1.371917
H	8.238294	3.604161	-1.957947
H	7.877838	2.105901	-1.047483
H	7.529922	2.197437	-2.800891
H	6.975912	5.262975	-1.539417
H	5.274895	5.169293	-2.077349
H	5.675115	4.982981	-0.342482

Table S37. Cartesian geometry of 3g in Figure S145 in Angstrom [Å].

Atomtype	X Coordinates	Y Coordinates	Z Coordinates
C	1.430433	-2.622232	3.825609
C	1.490579	-1.615929	2.838310
C	2.671285	-0.854199	2.721025
C	3.775683	-1.103652	3.550724
C	3.711696	-2.117041	4.521850
C	2.532500	-2.869960	4.661471
P	0.139291	-1.241954	1.585429
C	-0.538431	-2.945309	1.243635
C	-1.285188	-3.721422	2.155360
C	-1.664576	-5.035189	1.832359
C	-1.324370	-5.584320	0.583370
C	-0.612426	-4.807433	-0.345465
C	-0.224888	-3.499961	-0.014545
Ni	0.732046	-0.124735	-0.161650
C	2.450889	0.462100	-0.701996
N	3.194064	1.627822	-0.535171
C	4.430828	1.557300	-1.191328
C	4.492005	0.334311	-1.788223
N	3.298150	-0.319033	-1.479032
C	2.743595	2.863502	0.050988
C	3.376588	3.338189	1.227119
C	3.050323	4.635879	1.673339
C	2.106064	5.416335	0.998484
C	1.464336	4.910599	-0.141569
C	1.773628	3.634882	-0.647923
C	2.886220	-1.543192	-2.114157
C	3.010646	-2.772112	-1.413353
C	2.638319	-3.945677	-2.095279
C	2.168502	-3.896740	-3.418205
C	2.050279	-2.669680	-4.082917
C	2.396022	-1.462975	-3.441555
C	4.392599	2.505775	2.009917
C	4.008408	2.392639	3.500846
C	1.160588	3.134236	-1.953688
C	2.092153	3.462138	-3.141688
C	3.499070	-2.778050	0.033513
C	3.133232	-4.040211	0.830996
C	2.271481	-0.127277	-4.176036
C	0.852025	0.135070	-4.716680
Ge	-1.315976	-0.068976	-1.097668
N	-2.143568	-1.149132	-2.408685
N	-2.707653	0.492587	0.127258
Si	-2.129214	0.993696	1.747575
C	-0.886131	2.449322	1.570774
C	-1.566539	3.830819	1.492718
C	-4.098362	0.403698	-0.178138
C	-4.727485	1.449624	-0.927456
C	-6.091808	1.338412	-1.252733

C	-6.848052	0.229731	-0.849281
C	-6.228508	-0.794572	-0.122211
C	-4.863046	-0.736125	0.218503
C	-3.934841	2.655324	-1.427372
C	-3.665063	2.549963	-2.944465
C	-4.222258	-1.935447	0.915670
C	-4.981192	-2.385806	2.179860
C	-3.614726	1.358198	2.918084
C	-4.651456	2.407340	2.466723
C	-1.185343	-0.435716	2.637547
C	-3.119002	1.685076	4.346798
C	-4.057026	-3.112666	-0.070319
C	-4.598152	4.003274	-1.081398
C	5.819938	3.077204	1.860834
C	-0.261420	3.653366	-2.214936
C	5.019963	-2.528924	0.141736
C	3.324856	-0.026800	-5.300359
C	0.218981	2.450557	2.646576
H	-0.378042	2.253944	0.600944
H	5.134232	2.388589	-1.178046
H	5.259197	-0.128237	-2.408433
H	2.716497	-0.090930	1.932204
H	2.481889	0.676879	-3.453581
H	-0.677199	-0.008846	3.520163
H	-1.903777	-1.183137	3.020828
H	-5.970075	-2.816677	1.933320
H	-5.147357	-1.546834	2.880338
H	-4.404803	-3.169520	2.707493
H	-2.003222	4.113703	2.469440
H	-2.376628	3.869679	0.743882
H	-0.827020	4.609729	1.227730
H	1.886279	-4.825539	-3.931020
H	-6.571080	2.134761	-1.836537
H	-3.206702	-1.641434	1.222323
H	-4.137809	0.380292	2.960429
H	1.075186	2.035583	-1.866209
H	0.348721	-2.896624	-0.727249
H	-7.912570	0.160299	-1.108262
H	-1.583027	-3.306993	3.126519
H	-6.815426	-1.673951	0.172603
H	3.536128	5.033235	2.572393
H	0.532506	-3.239661	3.934743
H	2.704784	-4.911525	-1.583598
H	-2.957204	2.625288	-0.919374
H	2.976469	-1.928220	0.506509
H	4.392665	1.485239	1.585980
H	1.669294	-2.642004	-5.111089
H	1.859627	6.420823	1.365692
H	4.693142	-0.515758	3.426477
H	1.672233	3.059379	-4.081798
H	3.097184	3.025962	-3.003230
H	2.206210	4.557321	-3.256355
H	0.879948	3.325644	2.517112
H	0.856116	1.551892	2.569893
H	-0.194746	2.494091	3.672967
H	0.722232	5.528618	-0.657986
H	-3.063538	1.654752	-3.183694
H	-3.115158	3.438974	-3.306011
H	-4.614822	2.480698	-3.508412
H	4.577640	-2.323710	5.164161

H	2.474299	-3.664885	5.416788
H	-3.666894	-4.002840	0.451768
H	-3.341908	-2.848690	-0.865752
H	-5.025796	-3.376807	-0.535347
H	-2.241067	-5.627739	2.554645
H	0.532213	-0.642395	-5.434605
H	0.825544	1.106168	-5.245513
H	0.116383	0.173435	-3.895371
H	-1.627941	-6.608658	0.331805
H	-0.347912	-5.215669	-1.329120
H	4.009900	3.381407	3.995312
H	4.735109	1.756328	4.037550
H	3.007437	1.946638	3.623973
H	-0.279779	4.731952	-2.462753
H	-0.914133	3.488422	-1.341295
H	-0.700685	3.111540	-3.070790
H	2.059745	-4.278310	0.759970
H	3.370528	-3.877240	1.897197
H	3.710223	-4.920576	0.486989
H	4.347314	-0.172483	-4.905673
H	3.280681	0.966789	-5.785339
H	3.150168	-0.794730	-6.077904
H	-5.476243	2.468344	3.205830
H	-5.099210	2.159645	1.491271
H	-4.207885	3.416393	2.393930
H	-5.544864	4.150227	-1.634972
H	-3.924747	4.839108	-1.351047
H	-4.821313	4.075031	-0.002567
H	-2.570291	2.646141	4.371531
H	-2.449877	0.908218	4.759766
H	-3.977004	1.785224	5.041949
H	6.130566	3.131325	0.802050
H	6.550682	2.445516	2.400620
H	5.878491	4.099157	2.280870
H	5.582597	-3.357130	-0.330815
H	5.312755	-2.477583	1.207136
H	5.327890	-1.585169	-0.337283
C	-3.477976	-1.159800	-2.987623
C	-1.251591	-2.089831	-3.067922
H	-1.538673	-3.143693	-2.847766
H	-1.256019	-1.966218	-4.173594
H	-0.209527	-1.956636	-2.724920
H	-3.933323	-2.173824	-2.939411
H	-4.156918	-0.468963	-2.470489
H	-3.445004	-0.870590	-4.063906

Table S38. Cartesian geometry of H_2 in Figure S145 in Angstrom [Å].

Atomtype	X Coordinates	Y Coordinates	Z Coordinates
H	0.000000	0.000000	0.380841
H	0.000000	0.000000	-0.380841

Table S39. Cartesian geometry of 3a-INT1 (16.2 kcal/mol) in Figure S145 in Angstrom [Å].

Atomtype	X Coordinates	Y Coordinates	Z Coordinates
C	-1.498032	3.320681	-1.775300
C	-2.431103	2.909730	-0.778915
C	-2.722314	3.705478	0.358392
C	-2.021977	4.921107	0.507260
C	-1.088998	5.338366	-0.447816
C	-0.840197	4.549048	-1.580761
N	-3.168993	1.689552	-0.988558

C	-2.631173	0.410056	-0.938134
N	-3.694831	-0.367679	-1.394342
C	-4.825602	0.393574	-1.686768
C	-4.496815	1.692110	-1.429956
Ni	-0.943960	-0.271055	-0.275241
Ge	1.226003	0.034135	-0.761218
C	2.426025	1.666820	-0.980613
C	1.812337	2.881463	-0.594036
C	2.480457	4.108030	-0.662883
C	3.803751	4.157263	-1.162057
C	4.421795	2.959530	-1.597673
C	3.739353	1.739998	-1.506078
C	-3.580042	-1.761013	-1.741160
C	-3.988081	-2.748102	-0.811025
C	-3.817902	-4.098814	-1.171800
C	-3.246375	-4.451885	-2.402008
C	-2.850760	-3.455913	-3.304460
C	-3.014000	-2.090728	-2.997130
C	-4.607845	-2.363144	0.527186
C	-4.116514	-3.229068	1.700477
C	-2.570910	-1.024966	-3.996213
C	-3.340932	-1.142321	-5.327956
C	-3.807919	3.334577	1.369702
C	-3.313628	3.365547	2.829545
C	-1.255873	2.479398	-3.030752
C	0.086277	2.751128	-3.731778
P	-0.558922	-0.547144	1.836585
C	0.221671	0.890144	2.734134
C	0.915067	0.795652	3.958544
C	1.378237	1.955066	4.600806
C	1.148914	3.222208	4.032852
C	0.451707	3.323942	2.818816
C	-0.007951	2.162919	2.177647
C	0.506047	-2.014688	2.260337
Si	1.751443	-2.648176	0.924166
C	3.098894	-3.509551	1.997989
C	4.373397	-4.048540	1.314281
N	2.436746	-1.256023	0.021129
C	3.852547	-1.097224	-0.130901
C	4.630009	-0.421758	0.859673
C	6.010185	-0.246338	0.636127
C	6.632023	-0.710571	-0.528366
C	5.866526	-1.371849	-1.498274
C	4.485775	-1.580193	-1.321552
C	4.021829	0.156549	2.138035
C	4.707014	-0.380353	3.414843
C	3.686456	-2.271570	-2.427010
C	3.330787	-1.292684	-3.568274
C	0.857165	-3.906440	-0.248642
C	1.773876	-5.032527	-0.775623
C	-2.027830	-0.744140	2.978750
C	-2.013461	-1.487730	4.177509
C	-3.148998	-1.521522	5.004877
C	-4.306665	-0.805173	4.651472
C	-4.324610	-0.057120	3.461470
C	-3.193530	-0.034962	2.632022
C	4.397282	-3.518052	-2.993315
C	4.074805	1.700475	2.143489
C	-6.149875	-2.379707	0.441231
C	-1.044831	-1.057892	-4.216290

C	-5.038385	4.254193	1.194486
C	-2.412600	2.656346	-4.041967
C	-0.416057	-4.529594	0.359436
C	2.467954	-4.610626	2.884739
H	0.547187	-3.297549	-1.121008
H	-5.742455	-0.056014	-2.067206
H	-5.068050	2.613691	-1.532861
H	-3.196559	0.524893	1.689505
H	-1.236577	1.426416	-2.694373
H	-0.187457	-2.846696	2.477401
H	1.077227	-1.840397	3.190818
H	5.734218	0.017976	3.515045
H	4.779778	-1.482396	3.421044
H	4.141180	-0.061855	4.311026
H	2.039600	-5.745183	0.026614
H	2.713963	-4.658400	-1.210395
H	1.252674	-5.613215	-1.563306
H	-0.556189	6.289199	-0.317035
H	6.353116	-1.728628	-2.414208
H	2.958201	-0.137199	2.169166
H	3.411684	-2.689372	2.673629
H	-2.811552	-0.039481	-3.568133
H	-0.566540	2.220714	1.234494
H	7.707901	-0.556358	-0.681768
H	1.093042	-0.179877	4.427631
H	6.608983	0.279950	1.390103
H	4.260835	0.838164	-1.836184
H	-4.121037	-4.885752	-0.472156
H	-1.114549	-2.040544	4.476907
H	-2.223958	5.552732	1.380420
H	2.732890	-2.599139	-1.981765
H	5.443632	2.990861	-1.994260
H	-4.136379	2.303947	1.152287
H	-4.298543	-1.326838	0.739222
H	0.780699	2.878695	-0.232084
H	-0.105892	4.890527	-2.316368
H	-3.107499	-5.510356	-2.657445
H	-5.224043	0.501728	3.171567
H	-3.055545	-0.318299	-6.008831
H	-4.433633	-1.092381	-5.164604
H	-3.117996	-2.095701	-5.843102
H	-0.887342	-5.230081	-0.358025
H	-1.179381	-3.772859	0.605278
H	-0.191977	-5.100867	1.281215
H	-2.403188	-3.739412	-4.265057
H	2.717180	-0.445888	-3.208924
H	2.757047	-1.808761	-4.361880
H	4.247149	-0.869268	-4.022190
H	-5.191420	-0.833921	5.300379
H	-3.128920	-2.108051	5.932838
H	3.612654	2.094413	3.065586
H	3.537123	2.128273	1.285133
H	5.121791	2.055919	2.102739
H	1.925323	1.869385	5.548641
H	0.107301	3.748064	-4.211941
H	0.240180	2.002472	-4.530075
H	0.937065	2.683599	-3.034945
H	1.519258	4.124659	4.535731
H	0.259710	4.302698	2.361967
H	-4.479088	-4.271523	1.626696

H	-4.482615	-2.811420	2.654120
H	-3.014735	-3.246572	1.747272
C	4.504296	5.409081	-1.234529
H	-0.718544	-2.023346	-4.647629
H	-0.515739	-0.902457	-3.258991
H	-0.740077	-0.254708	-4.912729
H	-2.445356	2.703751	2.977216
H	-4.117631	3.027348	3.508283
H	-3.022187	4.386639	3.138385
H	-3.391458	2.391096	-3.609008
H	-2.248300	2.010869	-4.924854
H	-2.462538	3.705693	-4.390338
H	5.091755	-4.401377	2.082256
H	4.885299	-3.284691	0.707466
H	4.152755	-4.910865	0.660697
H	5.270411	-3.245942	-3.615570
H	3.705311	-4.095443	-3.634528
H	4.756150	-4.179966	-2.184431
H	1.981856	5.028694	-0.338089
H	2.118216	-5.468359	2.280101
H	1.606794	-4.248781	3.476399
H	3.216270	-5.005651	3.601030
H	-6.518471	-1.708246	-0.355436
H	-6.593080	-2.052149	1.400870
H	-6.518136	-3.400228	0.221832
H	-4.776114	5.305341	1.418490
H	-5.850515	3.951756	1.882518
H	-5.430668	4.221966	0.161639
H	-0.915425	-1.866114	-1.057525
H	-1.382739	-1.982781	-0.409175
N	5.070659	6.433542	-1.292697

Table S40. Cartesian geometry of 3b-INT1 (16.3 kcal/mol) in Figure S145 in Angstrom [Å].

Atomtype	X Coordinates	Y Coordinates	Z Coordinates
C	-0.834604	3.325326	-1.743163
C	-2.008702	3.192725	-0.944991
C	-2.289115	4.063447	0.137815
C	-1.345174	5.069247	0.434098
C	-0.171359	5.199650	-0.312342
C	0.077314	4.335460	-1.389693
N	-2.970269	2.180458	-1.300077
C	-2.749932	0.814310	-1.198623
N	-3.880443	0.290691	-1.820552
C	-4.754298	1.283953	-2.261538
C	-4.182002	2.478008	-1.932335
Ni	-1.361286	-0.202083	-0.314317
Ge	0.863222	-0.393238	-0.466702
C	2.529833	0.811931	-0.546265
C	2.422778	2.151349	-0.104121
C	3.525621	3.027030	-0.166660
C	4.749256	2.599866	-0.705870
C	4.856461	1.286568	-1.183736
C	3.764453	0.408209	-1.101287
C	-4.034905	-1.099977	-2.159503
C	-4.795238	-1.943079	-1.312847
C	-4.888771	-3.305463	-1.657762
C	-4.233670	-3.811068	-2.788992
C	-3.484391	-2.956082	-3.608211
C	-3.372983	-1.582658	-3.315378
C	-5.506369	-1.393246	-0.081381

C	-5.379282	-2.302998	1.153429
C	-2.548678	-0.671951	-4.222078
C	-3.123570	-0.629969	-5.653236
C	-3.581006	3.987386	0.952013
C	-3.324540	3.930160	2.471819
C	-0.604757	2.442937	-2.972613
C	0.855118	2.388384	-3.452726
P	-1.351254	-0.509124	1.823817
C	-0.342748	0.698494	2.822276
C	0.225791	0.427815	4.083647
C	0.891541	1.441025	4.793212
C	0.977777	2.740127	4.261629
C	0.403582	3.020054	3.010946
C	-0.239155	2.001138	2.291208
C	-0.761701	-2.189563	2.355391
Si	0.466975	-3.134796	1.201413
C	1.401949	-4.253065	2.462612
C	2.600435	-5.100040	1.985178
N	1.585700	-1.972917	0.403467
C	2.998759	-2.221310	0.373020
C	3.851374	-1.756812	1.422238
C	5.241409	-1.954891	1.306280
C	5.802863	-2.595850	0.197181
C	4.964533	-3.061464	-0.823231
C	3.568842	-2.890493	-0.758915
C	3.322730	-1.041352	2.666391
C	3.717873	-1.774129	3.969491
C	2.703762	-3.378273	-1.924271
C	2.661376	-2.362877	-3.089179
C	-0.537515	-4.200027	-0.073335
C	0.135404	-5.544010	-0.432499
C	-2.961216	-0.315769	2.755858
C	-3.270423	-0.993565	3.954091
C	-4.490364	-0.754696	4.609148
C	-5.409468	0.170841	4.082328
C	-5.103336	0.854400	2.893115
C	-3.889464	0.605043	2.235242
C	3.135604	-4.763067	-2.450883
C	3.810954	0.421565	2.744925
C	-6.988731	-1.093895	-0.395006
C	-1.058500	-1.069016	-4.219091
C	-4.511570	5.171149	0.603003
C	-1.515719	2.882148	-4.142739
C	-1.998893	-4.462315	0.344127
C	0.407563	-5.143645	3.247859
H	-0.561115	-3.584456	-0.994402
H	-5.683159	1.047902	-2.780234
H	-4.508272	3.503569	-2.099628
H	-3.642832	1.111904	1.295254
H	-0.885188	1.416919	-2.671556
H	-1.668155	-2.814400	2.443200
H	-0.314272	-2.159504	3.365677
H	4.801529	-1.672811	4.167914
H	3.492219	-2.853894	3.929384
H	3.181136	-1.334780	4.832071
H	0.084857	-6.257600	0.410023
H	1.195228	-5.439266	-0.711529
H	-0.387056	-6.017411	-1.288134
H	0.563382	5.971419	-0.059472
H	5.408706	-3.550689	-1.697977

H	2.220503	-1.019781	2.599496
H	1.792885	-3.503271	3.177931
H	-2.607774	0.350634	-3.817965
H	-0.675543	2.201526	1.303263
H	6.890046	-2.719300	0.120206
H	0.153160	-0.571787	4.528988
H	5.900691	-1.583855	2.100896
H	3.905952	-0.607239	-1.471089
H	-5.468514	-3.983758	-1.021628
H	-2.559215	-1.708607	4.385909
H	-1.536984	5.756707	1.266417
H	1.672371	-3.464176	-1.548435
C	6.186951	0.797634	-1.720299
H	-4.109351	3.060954	0.668636
H	-5.020905	-0.436819	0.172074
H	1.483168	2.522072	0.318282
H	1.004072	4.456419	-1.956320
H	-4.306013	-4.879139	-3.031657
H	-5.814898	1.574899	2.469072
H	-2.554495	0.088069	-6.273452
H	-4.184580	-0.317889	-5.649477
H	-3.062686	-1.619853	-6.143704
H	-2.516519	-5.076837	-0.418710
H	-2.582073	-3.532055	0.443679
H	-2.058472	-5.008725	1.305348
H	-2.972921	-3.359441	-4.490907
H	2.183287	-1.411189	-2.790720
H	2.076088	-2.768775	-3.936262
H	3.680772	-2.129052	-3.449840
H	-6.361648	0.354408	4.596553
H	-4.723988	-1.291573	5.537989
H	3.366242	0.926578	3.621543
H	3.531216	0.991653	1.848086
H	4.912163	0.463249	2.844635
H	1.343896	1.214116	5.767432
H	1.181378	3.351011	-3.890469
H	0.954521	1.620032	-4.241026
H	1.548923	2.127507	-2.637678
H	1.497633	3.529853	4.818913
H	0.462765	4.025391	2.581374
H	-5.926987	-3.255393	1.025355
H	-5.791809	-1.792276	2.040688
H	-4.322626	-2.535269	1.366999
H	5.604311	3.281270	-0.753288
H	-0.909045	-2.090474	-4.617541
H	-0.652399	-1.028091	-3.192718
H	-0.473992	-0.371264	-4.847205
H	-2.651622	3.098511	2.737044
H	-4.276869	3.782968	3.012954
H	-2.870989	4.868494	2.841459
H	-2.584853	2.857838	-3.873851
H	-1.371361	2.210673	-5.009798
H	-1.264268	3.911631	-4.462044
H	3.095569	-5.575724	2.856467
H	3.360601	-4.503264	1.456698
H	2.280663	-5.915869	1.313057
H	4.076097	-4.705357	-3.030196
H	2.360695	-5.174874	-3.124039
H	3.295733	-5.479409	-1.624851
C	3.419062	4.460031	0.308289

H	-0.039305	-5.923058	2.603002
H	-0.423398	-4.571535	3.700044
H	0.930570	-5.669517	4.071921
H	-7.088852	-0.387167	-1.238989
H	-7.488171	-0.650605	0.487448
H	-7.526814	-2.022742	-0.665086
H	-4.039591	6.133316	0.877392
H	-5.467278	5.090839	1.155052
H	-4.739001	5.208850	-0.478068
H	-1.564136	-1.784995	-1.103310
H	-2.140620	-1.774411	-0.537113
F	6.040727	-0.229778	-2.592501
F	6.861068	1.784621	-2.365294
F	6.997327	0.360479	-0.720797
F	4.602159	4.932264	0.769806
F	3.038453	5.300955	-0.700698
F	2.510689	4.610527	1.303332

Table S41. Cartesian geometry of 3c-INT1 (16.1 kcal/mol) in Figure S145 in Angstrom [Å].

Atomtype	X Coordinates	Y Coordinates	Z Coordinates
C	4.699367	0.682884	0.675863
C	4.063303	-0.272008	-0.175891
C	4.781798	-0.822665	-1.285647
C	6.105895	-0.408216	-1.520664
C	6.735644	0.521963	-0.682452
C	6.030872	1.054324	0.403057
N	2.699946	-0.657475	0.033414
Si	2.292036	-1.993355	1.160351
C	0.993833	-1.383236	2.455433
P	-0.338473	-0.220083	1.866776
Ni	-0.804101	-0.335652	-0.244137
Ge	1.266375	0.277201	-0.875795
C	2.152923	2.019434	-1.421609
C	1.346337	3.160304	-1.201056
C	1.798104	4.455552	-1.499825
C	3.072689	4.639592	-2.060226
C	3.877449	3.517479	-2.323892
C	3.425147	2.225193	-2.007835
C	4.124628	-1.817587	-2.243170
C	5.050345	-2.993520	-2.616124
C	3.978644	1.358647	1.843384
C	3.788861	2.871422	1.592448
C	-1.720151	-0.505827	3.097667
C	-1.546260	-1.048640	4.388187
C	-2.639826	-1.162394	5.263761
C	-3.916281	-0.725452	4.866332
C	-4.094784	-0.177987	3.583870
C	-3.003583	-0.077569	2.708166
C	0.175033	1.455581	2.510895
C	0.857816	1.673989	3.725588
C	1.113492	2.981128	4.169158
C	0.684049	4.085649	3.409438
C	-0.003064	3.875495	2.203831
C	-0.255350	2.567101	1.762106
C	-2.597305	-0.065195	-0.917317
N	-3.516988	-1.075842	-1.202932
C	-4.776302	-0.575574	-1.531555
C	-4.681935	0.783541	-1.473612
N	-3.362313	1.078857	-1.112943
C	-3.165383	-2.463625	-1.356609

C	-3.376291	-3.360614	-0.280740
C	-2.980545	-4.700795	-0.456673
C	-2.380766	-5.126641	-1.649798
C	-2.183185	-4.219533	-2.699175
C	-2.576527	-2.872151	-2.578632
C	-2.854573	2.427237	-1.117202
C	-3.255053	3.318571	-0.089249
C	-2.784719	4.647104	-0.149963
C	-1.968335	5.076092	-1.201553
C	-1.610249	4.184591	-2.223866
C	-2.039822	2.845091	-2.209892
C	-4.026319	-2.897257	1.017823
C	-5.541912	-3.194532	1.008740
C	-2.348456	-1.903834	-3.736307
C	-0.845145	-1.703252	-4.015396
C	-4.224507	2.915216	1.022521
C	-5.604963	3.572815	0.794192
C	-1.682098	1.883330	-3.345322
C	-2.883171	1.690777	-4.300680
C	-3.362622	-3.483047	2.276472
C	-3.109378	-2.346242	-5.003346
C	-3.697183	3.240159	2.434150
C	-0.439958	2.287805	-4.157915
C	1.601114	-3.550622	0.233264
C	0.467422	-4.271047	0.990988
C	3.799400	-2.445382	2.273546
C	3.391578	-3.476524	3.353919
C	2.680169	-4.582002	-0.164226
C	3.598691	-1.121533	-3.518117
C	4.704438	1.156891	3.192053
C	5.124768	-2.875414	1.609629
H	1.172620	-3.144267	-0.704223
H	-5.607344	-1.229544	-1.794536
H	-5.414564	1.565385	-1.669166
H	-3.127494	0.325538	1.696142
H	-1.452541	0.914965	-2.863052
H	0.477009	-2.278580	2.844632
H	1.552270	-0.954520	3.307945
H	5.668505	1.699491	3.212111
H	4.920968	0.093977	3.399769
H	4.083672	1.553718	4.017805
H	3.085697	-5.104340	0.721741
H	3.530239	-4.135288	-0.703137
H	2.241643	-5.358504	-0.823271
H	-1.611425	6.113790	-1.232156
H	6.655937	-0.817981	-2.376664
H	2.973933	0.909201	1.924311
H	3.993401	-1.487468	2.794640
H	-2.752862	-0.923489	-3.440592
H	-0.805044	2.380299	0.830796
H	7.770301	0.832437	-0.877974
H	1.187418	0.827904	4.341300
H	6.520676	1.792167	1.051221
H	4.090786	1.382410	-2.209872
H	-3.127768	-5.417484	0.359101
H	-0.555080	-1.380064	4.721676
H	-3.073927	5.354453	0.636372
H	3.244341	-2.231319	-1.724636
H	4.873201	3.646609	-2.768885
H	-4.369809	1.823116	0.966217

H	-3.902834	-1.803332	1.067543
H	0.339054	3.043005	-0.791467
H	-0.966091	4.535819	-3.035213
H	-2.064122	-6.171817	-1.760893
H	-5.087260	0.161118	3.259077
H	-2.988072	-1.593134	-5.804759
H	-4.190341	-2.463936	-4.800961
H	-2.729367	-3.310954	-5.389453
H	0.101498	-5.140840	0.410255
H	-0.404928	-3.619103	1.161982
H	0.809378	-4.648913	1.974183
H	-1.712217	-4.559246	-3.629931
H	2.838665	-0.353870	-3.283826
H	3.133487	-1.858000	-4.201379
H	4.422592	-0.618359	-4.059569
H	-4.767811	-0.815758	5.553037
H	-2.493944	-1.591639	6.263848
H	3.216058	3.323916	2.421125
H	3.245450	3.063266	0.656208
H	4.768479	3.382653	1.530024
H	1.653669	3.139604	5.111662
H	-0.627179	3.186748	-4.776264
H	-0.173201	1.467774	-4.849363
H	0.430813	2.484729	-3.512199
H	0.892129	5.105570	3.757599
H	-0.348484	4.722625	1.598573
H	-3.535472	-4.571884	2.367604
H	-3.775222	-2.998782	3.178264
H	-2.274707	-3.302984	2.271301
H	3.434749	5.648934	-2.297132
H	-0.359883	-2.649989	-4.320404
H	-0.336821	-1.320524	-3.112266
H	-0.700181	-0.969309	-4.829899
H	-2.721734	2.763426	2.620962
H	-4.407012	2.872206	3.197280
H	-3.579347	4.329680	2.582985
H	-3.779566	1.316991	-3.778418
H	-2.626231	0.962877	-5.092724
H	-3.143542	2.649269	-4.789373
H	5.914238	-2.983222	2.381373
H	5.483817	-2.144451	0.867864
H	5.032282	-3.853918	1.106308
H	5.866826	-2.675816	-3.291578
H	4.477818	-3.780239	-3.142298
H	5.513193	-3.441559	-1.718206
H	1.146801	5.316470	-1.298744
H	3.168092	-4.464404	2.908731
H	2.503651	-3.166498	3.935178
H	4.219823	-3.628885	4.075172
H	-6.042769	-2.720783	0.144997
H	-6.015148	-2.813048	1.933541
H	-5.725982	-4.284577	0.951599
H	-5.529096	4.674702	0.858533
H	-6.327614	3.235931	1.561559
H	-6.017297	3.325173	-0.201070
H	-0.506824	-2.001361	-0.781314
H	-0.948990	-2.096952	-0.111008

Table S42. Cartesian geometry of 3d-INT1 (15.5 kcal/mol) in Figure S145 in Angstrom [Å].

Atomtype	X Coordinates	Y Coordinates	Z Coordinates
C	-1.707579	3.205414	-1.869104
C	-2.603589	2.751147	-0.858008
C	-2.946825	3.549484	0.262684
C	-2.338711	4.817013	0.378792
C	-1.444180	5.278924	-0.592495
C	-1.141689	4.483224	-1.707533
N	-3.252079	1.476849	-1.034227
C	-2.626675	0.239596	-0.938272
N	-3.631432	-0.623422	-1.378953
C	-4.807525	0.049512	-1.708154
C	-4.571831	1.374505	-1.488284
Ni	-0.903963	-0.302027	-0.252868
Ge	1.229646	0.183289	-0.761659
C	2.300395	1.875714	-1.058997
C	1.619762	3.055405	-0.682343
C	2.214313	4.321851	-0.790220
C	3.515245	4.466561	-1.309675
C	4.185926	3.297858	-1.730434
C	3.596738	2.031100	-1.607327
C	-3.418367	-2.014583	-1.683810
C	-3.729336	-2.997507	-0.711770
C	-3.475045	-4.343711	-1.036632
C	-2.912939	-4.695007	-2.272282
C	-2.604276	-3.702885	-3.211817
C	-2.853143	-2.342756	-2.939887
C	-4.318042	-2.601895	0.637399
C	-3.812049	-3.461588	1.808369
C	-2.486137	-1.277139	-3.969564
C	-3.236406	-1.486374	-5.300887
C	-3.996673	3.119360	1.287883
C	-3.507198	3.236713	2.744926
C	-1.403388	2.350378	-3.101553
C	-0.086828	2.705806	-3.814244
P	-0.489320	-0.514635	1.859498
C	0.195251	0.994504	2.719783
C	0.898627	0.974716	3.941973
C	1.290259	2.176530	4.553128
C	0.977387	3.412057	3.955916
C	0.270298	3.439237	2.743682
C	-0.117029	2.235894	2.133468
C	0.693166	-1.886217	2.292071
Si	1.976287	-2.434725	0.953986
C	3.394921	-3.167278	2.034310
C	4.707291	-3.614274	1.355927
N	2.541916	-1.013410	0.016205
C	3.939756	-0.755571	-0.158302
C	4.676922	-0.000831	0.805797
C	6.039880	0.262102	0.564678
C	6.682682	-0.189161	-0.593561
C	5.955636	-0.923688	-1.540074
C	4.594003	-1.220892	-1.344594
C	4.038403	0.571359	2.072038
C	4.760931	0.115175	3.359458
C	3.832310	-1.989167	-2.425704
C	3.383056	-1.061024	-3.576541
C	1.179708	-3.787499	-0.185219
C	2.181109	-4.848209	-0.693457
C	-1.915331	-0.801298	3.038658

C	-1.834259	-1.551035	4.230765
C	-2.949402	-1.657983	5.079888
C	-4.154525	-1.010495	4.754351
C	-4.239980	-0.256045	3.570839
C	-3.128263	-0.159743	2.720988
C	4.626328	-3.187181	-2.985988
C	3.990147	2.115120	2.035984
C	-5.861668	-2.600950	0.591775
C	-0.959661	-1.206281	-4.180105
C	-5.300978	3.925527	1.089294
C	-2.570061	2.408550	-4.115262
C	-0.037607	-4.497058	0.441812
C	2.859704	-4.299514	2.944578
H	0.818861	-3.223523	-1.068223
H	-5.685697	-0.472343	-2.087545
H	-5.203661	2.250837	-1.626150
H	-3.182952	0.405930	1.783340
H	-1.303864	1.311802	-2.734826
H	0.069907	-2.769677	2.518632
H	1.252060	-1.658460	3.218497
H	5.762603	0.578135	3.440925
H	4.901059	-0.979669	3.397895
H	4.180654	0.425073	4.249471
H	2.502047	-5.523247	0.121126
H	3.089047	-4.409580	-1.135867
H	1.707061	-5.482269	-1.469840
H	-0.980585	6.268319	-0.486219
H	6.457581	-1.268331	-2.452427
H	2.996096	0.210793	2.116400
H	3.642578	-2.311915	2.693231
H	-2.799187	-0.299793	-3.570455
H	-0.678879	2.234829	1.190360
H	7.744656	0.032863	-0.761137
H	1.139105	0.023845	4.433458
H	6.606919	0.847743	1.299419
H	4.174005	1.162745	-1.934204
H	-3.699476	-5.127433	-0.304787
H	-0.898763	-2.052044	4.508574
H	-2.582161	5.452112	1.238870
H	2.913570	-2.379693	-1.958752
H	5.197177	3.379671	-2.153579
H	-4.236264	2.058455	1.100654
H	-3.990040	-1.568044	0.832976
H	0.597408	2.995913	-0.298719
H	-0.432581	4.857156	-2.451797
H	-2.708048	-5.749120	-2.500111
H	-5.176828	0.249790	3.302730
H	-3.007614	-0.661946	-6.002397
H	-4.330963	-1.512428	-5.144119
H	-2.940995	-2.433909	-5.789850
H	-0.450468	-5.252473	-0.255845
H	-0.859523	-3.797976	0.668178
H	0.232493	-5.023426	1.378003
H	-2.155552	-3.984381	-4.172528
H	2.712862	-0.257013	-3.221158
H	2.838084	-1.634978	-4.350612
H	4.256437	-0.576835	-4.053937
H	-5.023648	-1.097471	5.419070
H	-2.876250	-2.249087	6.002365
H	3.489632	2.500225	2.942068

H	3.439484	2.483742	1.158649
H	5.013037	2.536421	2.000280
H	1.845847	2.149227	5.499610
H	-0.148899	3.684194	-4.328512
H	0.126920	1.945329	-4.587476
H	0.766622	2.732258	-3.117910
H	1.291629	4.348455	4.434658
H	0.016336	4.392002	2.262719
H	-4.202443	-4.495874	1.762435
H	-4.140960	-3.019884	2.764531
H	-2.710042	-3.507252	1.823375
C	4.186318	5.819598	-1.400811
H	-0.562159	-2.158034	-4.581336
H	-0.451233	-0.986767	-3.224078
H	-0.707281	-0.402723	-4.896935
H	-2.580657	2.663658	2.908827
H	-4.276091	2.844738	3.435372
H	-3.312405	4.288926	3.024389
H	-3.525926	2.080248	-3.674471
H	-2.355100	1.753294	-4.980022
H	-2.700868	3.440758	-4.493125
H	5.452598	-3.896423	2.127691
H	5.155798	-2.822225	0.735474
H	4.555160	-4.502102	0.717072
H	5.462768	-2.859517	-3.631674
H	3.968683	-3.828299	-3.602396
H	5.052492	-3.804455	-2.174392
H	1.650767	5.209789	-0.471648
H	2.579214	-5.194265	2.357708
H	1.973901	-3.999182	3.534147
H	3.640625	-4.618691	3.664106
H	-6.244502	-1.928722	-0.196670
H	-6.273796	-2.262944	1.561599
H	-6.246569	-3.618842	0.388795
H	-5.127271	5.001715	1.278428
H	-6.083429	3.578109	1.790494
H	-5.691407	3.826783	0.060092
H	-0.745496	-1.901190	-1.006104
H	-1.213429	-2.044126	-0.361703
H	4.719610	5.943426	-2.362387
H	3.459176	6.645739	-1.299218
H	4.940955	5.940005	-0.597409

Table S43. Cartesian geometry of 3e-INT1 (15.9 kcal/mol) in Figure S145 in Angstrom [Å].

Atomtype	X Coordinates	Y Coordinates	Z Coordinates
C	-1.306937	3.470030	-1.646902
C	-2.269525	3.065793	-0.676374
C	-2.523349	3.821883	0.496351
C	-1.756168	4.986714	0.707408
C	-0.793752	5.394973	-0.222008
C	-0.582081	4.648165	-1.390544
N	-3.076279	1.903684	-0.949195
C	-2.617868	0.591156	-0.955615
N	-3.723873	-0.093991	-1.463330
C	-4.800807	0.750837	-1.729282
C	-4.395533	2.011395	-1.403716
Ni	-0.997753	-0.230774	-0.291722
Ge	1.199891	-0.014455	-0.725109
C	2.494203	1.526994	-0.892424
C	1.959639	2.776747	-0.489873

C	2.714697	3.951818	-0.500783
C	4.053604	3.924923	-0.948516
C	4.601769	2.703825	-1.396750
C	3.826910	1.531455	-1.362561
C	-3.696308	-1.473107	-1.875914
C	-4.181644	-2.474210	-0.998909
C	-4.100259	-3.814406	-1.423929
C	-3.539648	-4.146183	-2.665176
C	-3.064856	-3.137378	-3.513555
C	-3.138227	-1.780642	-3.140977
C	-4.784851	-2.111173	0.352951
C	-4.384122	-3.076216	1.482295
C	-2.606350	-0.701086	-4.080042
C	-3.345110	-0.710831	-5.434275
C	-3.636155	3.464834	1.482579
C	-3.151267	3.389026	2.943825
C	-1.098799	2.672787	-2.936563
C	0.263166	2.900821	-3.614997
P	-0.681872	-0.630505	1.811944
C	0.157022	0.723231	2.788045
C	0.762365	0.543800	4.049276
C	1.282610	1.644784	4.747789
C	1.199098	2.938298	4.198476
C	0.591081	3.124952	2.947437
C	0.072204	2.021660	2.251462
C	0.304456	-2.166048	2.192793
Si	1.525930	-2.809067	0.839493
C	2.801827	-3.806342	1.885759
C	4.043389	-4.404110	1.190564
N	2.310782	-1.423309	0.014274
C	3.736856	-1.343534	-0.077726
C	4.511354	-0.746907	0.964844
C	5.908231	-0.655223	0.805577
C	6.549826	-1.122961	-0.346958
C	5.786917	-1.701662	-1.370499
C	4.389791	-1.827128	-1.257323
C	3.881341	-0.158589	2.227968
C	4.454879	-0.773556	3.523877
C	3.594433	-2.432647	-2.414517
C	3.340261	-1.397990	-3.533335
C	0.572965	-3.955068	-0.400493
C	1.416357	-5.120106	-0.964218
C	-2.175176	-0.799257	2.928998
C	-2.232944	-1.612007	4.080545
C	-3.383405	-1.618373	4.888177
C	-4.484345	-0.806054	4.562571
C	-4.430536	0.011106	3.419672
C	-3.285557	0.005730	2.609558
C	4.249743	-3.702412	-2.995156
C	4.044328	1.376855	2.279474
C	-6.321640	-1.992323	0.255161
C	-1.079534	-0.818767	-4.262938
C	-4.808957	4.463626	1.351136
C	-2.233465	2.957865	-3.948210
C	-0.750241	-4.513725	0.161244
C	2.088186	-4.896842	2.721336
H	0.322491	-3.288757	-1.249372
H	-5.738154	0.379861	-2.143046
H	-4.906639	2.971149	-1.466286
H	-3.233407	0.619175	1.702332

H	-1.141574	1.606596	-2.645922
H	-0.429337	-2.971422	2.375758
H	0.877754	-2.051363	3.131417
H	5.508339	-0.471959	3.677661
H	4.426920	-1.877674	3.514595
H	3.877775	-0.417355	4.398386
H	1.622362	-5.881264	-0.189297
H	2.385220	-4.795947	-1.375461
H	0.865172	-5.631318	-1.779394
H	-0.205434	6.303970	-0.041296
H	6.289249	-2.060122	-2.277388
H	2.799456	-0.373928	2.195584
H	3.165164	-3.037995	2.596069
H	-2.798215	0.277178	-3.612687
H	-0.424934	2.146118	1.280961
H	7.639404	-1.035701	-0.449664
H	0.822592	-0.453476	4.502769
H	6.504993	-0.191807	1.601596
H	4.303382	0.608650	-1.702113
H	-4.464040	-4.611623	-0.766086
H	-1.378332	-2.240842	4.359211
H	-1.927366	5.585019	1.610225
H	2.606318	-2.714210	-2.015407
H	5.632277	2.644647	-1.761926
H	-4.023697	2.468663	1.209199
H	-4.391378	-1.117863	0.622839
H	0.919654	2.842503	-0.158282
H	0.180619	4.977916	-2.101996
H	-3.470384	-5.198187	-2.971011
H	-5.285426	0.645574	3.151100
H	-2.993278	0.125363	-6.067641
H	-4.437163	-0.603303	-5.295861
H	-3.164239	-1.650628	-5.989423
H	-1.259282	-5.144669	-0.594114
H	-1.460956	-3.716309	0.433703
H	-0.580485	-5.141272	1.057866
H	-2.624572	-3.404231	-4.482310
H	2.767005	-0.527404	-3.165730
H	2.766659	-1.853345	-4.363579
H	4.296669	-1.017444	-3.940520
H	-5.381041	-0.813978	5.195585
H	-3.419412	-2.259367	5.779011
H	3.542814	1.781751	3.175998
H	3.606283	1.861077	1.394891
H	5.114839	1.654041	2.327621
H	1.758904	1.493160	5.725273
H	0.340901	3.915036	-4.051851
H	0.383070	2.179731	-4.444127
H	1.102530	2.757400	-2.915709
H	1.614006	3.794731	4.745414
H	0.514289	4.125116	2.503571
H	-4.836529	-4.077385	1.352139
H	-4.722273	-2.677008	2.453952
H	-3.288853	-3.193843	1.534338
O	4.720852	5.115088	-0.920520
H	-0.800996	-1.784088	-4.727124
H	-0.570538	-0.736666	-3.285825
H	-0.706891	-0.007221	-4.915387
H	-2.326431	2.667974	3.059182
H	-3.979284	3.066368	3.601028

H	-2.798632	4.372478	3.306572
H	-3.230138	2.727512	-3.536490
H	-2.094660	2.345016	-4.858417
H	-2.222388	4.023160	-4.248861
H	4.725298	-4.841337	1.948486
H	4.615202	-3.651767	0.624442
H	3.771136	-5.218189	0.496039
H	5.165091	-3.466791	-3.570072
H	3.551988	-4.212531	-3.685438
H	4.529426	-4.412346	-2.195751
H	2.286147	4.906069	-0.172443
H	1.681458	-5.700003	2.078272
H	1.251241	-4.500253	3.325165
H	2.801406	-5.376007	3.422186
H	-6.623477	-1.249204	-0.504904
H	-6.746519	-1.681483	1.228600
H	-6.771708	-2.964693	-0.022978
H	-4.486812	5.484460	1.630747
H	-5.642402	4.174569	2.019304
H	-5.195334	4.508142	0.316444
H	-1.061428	-1.778546	-1.156170
H	-1.549961	-1.898585	-0.522271
C	6.074192	5.149720	-1.342534
H	6.414062	6.192903	-1.224031
H	6.715866	4.486761	-0.724551
H	6.183300	4.852586	-2.407092

Table S44. Cartesian geometry of 3f-INT1 (16.8 kcal/mol) in Figure S145 in Angstrom [Å].

Atomtype	X Coordinates	Y Coordinates	Z Coordinates
C	-0.907528	3.556935	-1.621361
C	-1.914837	3.265999	-0.655820
C	-2.073383	4.034400	0.525633
C	-1.167192	5.091829	0.750208
C	-0.161709	5.388231	-0.176297
C	-0.042824	4.633708	-1.352913
N	-2.863018	2.220567	-0.943551
C	-2.581550	0.858441	-0.952418
N	-3.763410	0.328208	-1.476291
C	-4.714810	1.309688	-1.751549
C	-4.150828	2.503960	-1.413212
Ni	-1.092007	-0.174616	-0.287962
Ge	1.118336	-0.235290	-0.715155
C	2.589709	1.122150	-0.863985
C	2.207913	2.421006	-0.455381
C	3.094987	3.500747	-0.421091
C	4.449059	3.341886	-0.833340
C	4.831722	2.048828	-1.298063
C	3.928402	0.980943	-1.305510
C	-3.912811	-1.040154	-1.898187
C	-4.516876	-1.977851	-1.024294
C	-4.611795	-3.313548	-1.459837
C	-4.105844	-3.704194	-2.707712
C	-3.506323	-2.759116	-3.550210
C	-3.400736	-1.407392	-3.166433
C	-5.046938	-1.548131	0.338612
C	-4.819361	-2.591353	1.446116
C	-2.727023	-0.399677	-4.094241
C	-3.453045	-0.299792	-5.451572
C	-3.225102	3.807136	1.505442
C	-2.760431	3.663332	2.968076

C	-0.794313	2.748727	-2.915928
C	0.590028	2.806481	-3.585067
P	-0.836090	-0.645723	1.808852
C	0.171821	0.572710	2.804658
C	0.744599	0.298948	4.064241
C	1.399966	1.313670	4.779621
C	1.486430	2.614726	4.248731
C	0.914118	2.895243	2.998399
C	0.259706	1.878093	2.285719
C	-0.056753	-2.302017	2.161351
Si	1.076976	-3.075080	0.799310
C	2.211061	-4.242319	1.834712
C	3.371469	-4.982518	1.136124
N	2.035384	-1.793536	-0.007464
C	3.459671	-1.894124	-0.098252
C	4.301718	-1.424039	0.956687
C	5.699130	-1.508621	0.798899
C	6.278497	-2.030427	-0.363400
C	5.450257	-2.483986	-1.399192
C	4.048378	-2.430909	-1.288538
C	3.749568	-0.785014	2.231744
C	4.250789	-1.483128	3.515471
C	3.184411	-2.899328	-2.459610
C	3.074222	-1.813103	-3.552778
C	-0.010623	-4.071932	-0.459103
C	0.687658	-5.319064	-1.044183
C	-2.334347	-0.639739	2.933428
C	-2.498587	-1.468750	4.062930
C	-3.637204	-1.343019	4.877706
C	-4.620096	-0.382043	4.581157
C	-4.459847	0.451786	3.460271
C	-3.327790	0.315780	2.643405
C	3.668805	-4.229871	-3.071006
C	4.091170	0.719987	2.303338
C	-6.539663	-1.160610	0.256051
C	-1.227995	-0.716981	-4.271718
C	-4.268634	4.940681	1.377354
C	-1.877294	3.181165	-3.931853
C	-1.392603	-4.475232	0.093637
C	1.362057	-5.246147	2.652009
H	-0.178425	-3.364663	-1.295394
H	-5.686681	1.068794	-2.181544
H	-4.529683	3.523126	-1.477048
H	-3.197714	0.941503	1.752371
H	-0.973329	1.694780	-2.632448
H	-0.890049	-3.008316	2.325860
H	0.520833	-2.280687	3.104094
H	5.331454	-1.304659	3.672814
H	4.098876	-2.576865	3.486599
H	3.717570	-1.079875	4.397451
H	0.798514	-6.113924	-0.283726
H	1.689812	-5.104805	-1.447450
H	0.082351	-5.744962	-1.869995
H	0.533646	6.216443	0.013754
H	5.904599	-2.883793	-2.314130
H	2.649686	-0.870741	2.202676
H	2.664696	-3.536131	2.557670
H	-2.790117	0.591329	-3.618618
H	-0.211197	2.078692	1.314959
H	7.370559	-2.081114	-0.464510

H	0.674130	-0.703913	4.503929
H	6.348476	-1.141438	1.603932
H	4.301993	0.015561	-1.655992
H	-5.068860	-4.063571	-0.804892
H	-1.736145	-2.214595	4.319115
H	-1.263283	5.696474	1.659939
H	2.166717	-3.057466	-2.066498
H	5.851811	1.861159	-1.646626
H	-3.728097	2.866823	1.222157
H	-4.483963	-0.645472	0.626331
H	1.175295	2.610064	-0.147593
H	0.756996	4.871846	-2.060208
H	-4.174978	-4.753746	-3.021967
H	-5.222827	1.202059	3.214219
H	-2.989805	0.487454	-6.075972
H	-4.521872	-0.049124	-5.316924
H	-3.395760	-1.250553	-6.014585
H	-1.973593	-5.026376	-0.672211
H	-2.000698	-3.601320	0.379996
H	-1.302630	-5.134124	0.979228
H	-3.106140	-3.073512	-4.522088
H	2.628622	-0.880289	-3.162043
H	2.442661	-2.164940	-4.391330
H	4.073996	-1.559086	-3.954430
H	-5.508417	-0.287397	5.218954
H	-3.756554	-1.998091	5.750894
H	3.654796	1.165032	3.214856
H	3.698106	1.266076	1.433603
H	5.187492	0.869193	2.335374
H	1.848575	1.088221	5.755994
H	0.797392	3.805091	-4.015878
H	0.622964	2.080392	-4.417909
H	1.399658	2.553888	-2.881538
H	2.005488	3.402736	4.809628
H	0.969668	3.902694	2.567735
H	-5.450388	-3.489316	1.305753
H	-5.068961	-2.153936	2.427984
H	-3.764424	-2.910870	1.482426
N	5.348851	4.396410	-0.792288
H	-1.077450	-1.705569	-4.746061
H	-0.717718	-0.714096	-3.291695
H	-0.747160	0.045081	-4.913093
H	-2.025743	2.850357	3.081253
H	-3.624100	3.434056	3.618553
H	-2.298164	4.596211	3.341352
H	-2.897749	3.076132	-3.527485
H	-1.811001	2.561082	-4.845321
H	-1.729545	4.238318	-4.225344
H	3.987640	-5.513145	1.890848
H	4.038055	-4.301017	0.584100
H	3.003406	-5.746288	0.428468
H	4.609128	-4.102480	-3.639883
H	2.912312	-4.627112	-3.773562
H	3.850677	-4.989858	-2.289517
H	2.717553	4.467840	-0.076503
H	0.862339	-5.983914	1.996190
H	0.577495	-4.756489	3.257910
H	2.005791	-5.819825	3.349236
H	-6.711106	-0.358260	-0.483630
H	-6.898639	-0.803511	1.240102

H	-7.153457	-2.033239	-0.039408
H	-3.826517	5.913439	1.664584
H	-5.133124	4.749983	2.041388
H	-4.643425	5.037402	0.342078
H	-1.343408	-1.693758	-1.170697
H	-1.848108	-1.755693	-0.540198
C	6.726177	4.193822	-1.207957
C	4.919569	5.693645	-0.301772
H	7.291367	5.133218	-1.093679
H	7.233485	3.415028	-0.599583
H	6.798303	3.882300	-2.271707
H	5.764116	6.401345	-0.329356
H	4.099394	6.121511	-0.917818
H	4.552767	5.641821	0.745948

Table S45. Cartesian geometry of 3g-INT1 (17.6 kcal/mol) in Figure S145 in Angstrom [Å].

Atomtype	X Coordinates	Y Coordinates	Z Coordinates
C	2.615493	0.149574	-3.639503
C	3.124210	-0.461873	-2.465407
C	3.415807	-1.847123	-2.390563
C	3.140218	-2.634011	-3.525761
C	2.647086	-2.051827	-4.703702
C	2.405741	-0.673109	-4.763559
N	3.458012	0.388723	-1.355930
C	2.586403	0.764432	-0.340243
N	3.331053	1.745036	0.318131
C	4.581829	1.951742	-0.266850
C	4.661770	1.097721	-1.325473
Ni	0.808694	0.184043	0.149182
Ge	-1.094096	0.242360	-1.064323
N	-1.616343	-0.290513	-2.805664
C	2.838389	2.616200	1.354438
C	2.830995	2.168076	2.700688
C	2.365489	3.065684	3.680191
C	1.905632	4.344197	3.332798
C	1.892893	4.749244	1.992929
C	2.360406	3.894773	0.973320
C	3.250273	0.739245	3.043772
C	2.711066	0.244650	4.395751
C	2.254948	4.309825	-0.495271
C	2.606479	5.789639	-0.742180
C	4.121020	-2.427635	-1.167024
C	3.677424	-3.852315	-0.792278
C	2.347203	1.653650	-3.709190
C	0.953203	2.001286	-4.267803
P	0.111976	-1.473881	1.369643
C	-0.348763	-3.025235	0.427175
C	-1.057709	-4.103731	0.996315
C	-1.265805	-5.283068	0.263267
C	-0.763883	-5.401558	-1.046513
C	-0.057313	-4.332473	-1.619665
C	0.148205	-3.153451	-0.884165
C	-1.418882	-1.076656	2.365784
Si	-2.617167	0.314701	1.759715
C	-4.289760	-0.227992	2.561271
C	-5.575815	0.565542	2.243884
N	-2.732073	0.321591	-0.031767
C	-4.012680	0.302184	-0.664586
C	-4.693731	-0.933183	-0.889973
C	-5.925957	-0.913502	-1.571238

C	-6.495956	0.283928	-2.021737
C	-5.829993	1.495104	-1.788701
C	-4.595926	1.530315	-1.112940
C	-4.102278	-2.276838	-0.462990
C	-5.116014	-3.176115	0.275916
C	-3.872186	2.863527	-0.923805
C	-3.077994	3.260153	-2.189214
C	-2.030994	2.029829	2.450400
C	-3.184144	3.007209	2.767341
C	1.200858	-2.325621	2.638237
C	0.765685	-2.806986	3.890519
C	1.660764	-3.466917	4.751745
C	2.998500	-3.665063	4.370028
C	3.439289	-3.193519	3.120038
C	2.547086	-2.524699	2.270172
C	-4.813646	4.009129	-0.500826
C	-3.505211	-3.031956	-1.668936
C	4.780869	0.544347	2.984907
C	0.848819	3.970193	-1.038380
C	5.653253	-2.382332	-1.374915
C	3.457954	2.358792	-4.518943
C	-1.092321	1.928702	3.669801
C	-4.132879	-0.372798	4.095181
H	-1.448705	2.472368	1.618221
H	5.285155	2.686691	0.124421
H	5.448945	0.931118	-2.059645
H	2.887170	-2.127946	1.307782
H	2.380776	2.046391	-2.679318
H	-1.059690	-0.757732	3.360653
H	-2.022718	-1.988199	2.534320
H	-5.887085	-3.570554	-0.412632
H	-5.639159	-2.634191	1.084772
H	-4.595645	-4.047327	0.717053
H	-3.749141	2.689425	3.662884
H	-3.904300	3.108238	1.939463
H	-2.781461	4.017836	2.982033
H	2.455455	-2.679127	-5.583897
H	-6.276838	2.430706	-2.146682
H	-3.264534	-2.072585	0.225375
H	-4.426920	-1.248485	2.152991
H	2.979204	3.708064	-1.072773
H	0.721878	-2.318963	-1.305990
H	-7.456355	0.274090	-2.553385
H	-1.431609	-4.039512	2.026339
H	-6.447469	-1.859957	-1.761754
H	2.337256	2.757224	4.729870
H	-0.276573	-2.676303	4.205634
H	3.341122	-3.711157	-3.498349
H	-3.132782	2.716923	-0.119169
H	3.883342	-1.761026	-0.319172
H	2.793769	0.106637	2.258003
H	2.025313	-0.229473	-5.691177
H	1.538831	5.022958	4.113684
H	4.483521	-3.336102	2.811940
H	2.649201	5.990098	-1.829089
H	3.585628	6.054310	-0.301468
H	1.845862	6.471341	-0.317467
H	-0.786813	2.937145	4.011797
H	-0.162832	1.381460	3.438701
H	-1.583715	1.423537	4.524129

H	1.505046	5.740693	1.732598
H	-2.322658	2.498967	-2.454935
H	-2.548648	4.219813	-2.032993
H	-3.756738	3.374691	-3.056009
H	3.694784	-4.179247	5.044918
H	1.308767	-3.830343	5.726358
H	-3.113220	-4.014040	-1.352768
H	-2.673467	-2.461630	-2.110105
H	-4.275760	-3.195639	-2.446509
H	-1.819206	-6.116031	0.716457
H	0.823890	1.646650	-5.307305
H	0.818737	3.098783	-4.276502
H	0.151324	1.565333	-3.648324
H	-0.928803	-6.325061	-1.616432
H	0.345179	-4.410695	-2.637670
H	3.183494	0.776036	5.244241
H	2.928021	-0.829807	4.512349
H	1.617478	0.376181	4.471364
H	0.068363	4.503957	-0.462756
H	0.650873	2.885950	-0.964584
H	0.758722	4.267171	-2.099881
H	2.585859	-3.918506	-0.658825
H	4.153357	-4.155359	0.157832
H	3.980135	-4.589527	-1.559645
H	4.457069	2.157113	-4.092058
H	3.300178	3.454243	-4.521803
H	3.459934	2.010644	-5.569626
H	-6.454478	0.043286	2.675083
H	-5.754113	0.676210	1.162676
H	-5.552774	1.575133	2.690232
H	-5.466130	4.333488	-1.333397
H	-4.225379	4.890534	-0.183473
H	-5.466719	3.707325	0.338191
H	-3.974805	0.608045	4.581701
H	-3.289800	-1.026473	4.385098
H	-5.052262	-0.806812	4.537702
H	5.195637	0.811204	1.998122
H	5.031017	-0.514638	3.184316
H	5.282310	1.166401	3.751352
H	5.945347	-3.018674	-2.232029
H	6.177074	-2.755567	-0.474333
H	6.007878	-1.356405	-1.575494
H	0.251796	1.679728	0.938643
H	0.657191	1.281005	1.516570
C	-2.820984	-0.106354	-3.601951
C	-0.602969	-1.037502	-3.532077
H	-0.895154	-2.104348	-3.667341
H	-0.420535	-0.611966	-4.541490
H	0.358080	-1.022419	-2.990724
H	-3.296805	-1.077865	-3.859054
H	-3.572810	0.502627	-3.085758
H	-2.570351	0.401053	-4.561918

Table S46. Cartesian geometry of 3a-TS1 (23.9 kcal/mol) in Figure S145 in Angstrom [Å].

Atomtype	X Coordinates	Y Coordinates	Z Coordinates
C	-1.979056	-1.299858	4.089871
C	-2.071690	-0.648121	2.840712
C	-3.322302	-0.141466	2.439369
C	-4.447227	-0.243112	3.269653
C	-4.344008	-0.883349	4.515785

C	-3.108766	-1.420378	4.917510
P	-0.616095	-0.304383	1.716849
C	0.655640	-1.534203	2.282876
Si	1.728671	-2.402868	0.933410
C	0.418622	-3.204009	-0.233296
C	-0.618337	-4.037691	0.552404
Ni	-1.016027	0.096461	-0.419482
C	-2.814049	0.312491	-0.920329
N	-3.735240	-0.608950	-1.375508
C	-4.935291	0.008899	-1.750467
C	-4.770597	1.349085	-1.547471
N	-3.477041	1.521894	-1.051524
C	-3.484012	-2.017527	-1.587398
C	-2.648041	-2.411720	-2.672094
C	-2.523522	-3.789920	-2.927605
C	-3.208033	-4.740120	-2.156000
C	-4.006398	-4.327712	-1.085245
C	-4.149425	-2.961170	-0.766768
C	-1.954280	-1.382110	-3.564537
C	-0.683182	-1.898190	-4.257942
C	-5.008167	-2.554632	0.428858
C	-6.506210	-2.809320	0.155348
C	-2.804819	2.792764	-0.914586
C	-1.977043	3.221954	-1.989971
C	-1.254410	4.416877	-1.819340
C	-1.382910	5.177847	-0.647069
C	-2.254095	4.765549	0.367022
C	-2.981170	3.562103	0.259064
C	-1.966498	2.485488	-3.328576
C	-3.029876	3.107023	-4.263360
C	-3.970825	3.167835	1.351874
C	-5.228327	4.063182	1.274221
C	-0.063600	1.311272	2.477130
C	0.068364	2.446872	1.653833
C	0.401017	3.694304	2.205938
C	0.593186	3.823994	3.589618
C	0.462514	2.695810	4.420302
C	0.137959	1.447697	3.867432
C	-0.591564	2.438151	-4.018093
C	-3.354800	3.195305	2.764168
C	-4.554251	-3.263956	1.721207
C	-2.941866	-0.801932	-4.601175
C	2.834203	-3.656732	1.904704
C	2.341817	-3.977560	3.332799
N	2.768557	-1.305613	0.022739
Ge	2.058072	-0.397183	-1.585746
C	2.984364	1.443150	-1.376850
C	4.393286	1.586648	-1.443349
C	5.011513	2.843463	-1.400122
C	4.226556	4.017589	-1.299985
C	2.815825	3.901397	-1.258656
C	2.218915	2.635556	-1.299562
C	4.847483	5.310870	-1.234138
N	5.346807	6.369983	-1.179268
C	3.102214	-4.971985	1.143148
C	4.145568	-1.066358	0.333842
C	4.534996	-0.104526	1.318376
C	5.902211	0.156558	1.531604
C	6.897227	-0.490193	0.790844
C	6.522250	-1.437847	-0.168845

C	5.169105	-1.750023	-0.405601
C	3.512104	0.635034	2.172809
C	3.698405	2.164558	2.164168
C	4.840683	-2.788384	-1.478184
C	5.684207	-4.074944	-1.346584
C	3.550525	0.098511	3.621101
C	4.995273	-2.198799	-2.897392
C	0.969722	-4.008299	-1.427290
H	-0.134038	-2.331144	-0.653817
H	-5.778963	-0.564855	-2.132116
H	-5.442269	2.191440	-1.710337
H	-3.397700	0.333043	1.456485
H	-2.265889	1.444535	-3.138615
H	0.114930	-2.367428	2.764405
H	1.283700	-1.080604	3.064994
H	4.516620	0.346874	4.100321
H	3.444759	-1.001346	3.650870
H	2.743706	0.550437	4.227543
H	1.492610	-4.924050	-1.100592
H	1.672396	-3.414668	-2.038957
H	0.138663	-4.323950	-2.087321
H	-0.809735	6.107110	-0.533516
H	7.299617	-1.949020	-0.750410
H	2.516646	0.429873	1.745960
H	3.802139	-3.120743	1.996984
H	-1.628184	-0.565256	-2.897728
H	-0.110924	2.353775	0.577138
H	7.957004	-0.260718	0.962094
H	0.024312	0.579339	4.527675
H	6.191985	0.894652	2.289843
H	5.037188	0.701181	-1.509332
H	-4.521924	-5.077468	-0.473667
H	-1.021887	-1.710111	4.434687
H	-2.364050	5.378246	1.268820
H	3.780868	-3.063887	-1.346959
H	6.104785	2.924170	-1.429211
H	-4.298567	2.134359	1.149491
H	-4.874822	-1.471379	0.590772
H	1.125045	2.591006	-1.253665
H	-0.594527	4.767945	-2.620515
H	-3.103091	-5.808418	-2.384187
H	-5.408995	0.166603	2.934295
H	-2.442253	-0.030407	-5.215686
H	-3.818481	-0.334343	-4.120355
H	-3.303896	-1.598874	-5.278516
H	-1.368432	-4.466181	-0.137400
H	-1.177649	-3.431625	1.287561
H	-0.145918	-4.878815	1.096736
H	-1.888454	-4.128454	-3.752350
H	4.342273	-1.318546	-3.047054
H	4.731180	-2.948710	-3.667850
H	6.039035	-1.874515	-3.073433
H	-5.222654	-0.973885	5.167230
H	-3.020273	-1.930628	5.885512
H	2.897703	2.641992	2.753625
H	3.655912	2.565158	1.138843
H	4.665789	2.462646	2.610302
H	0.614543	2.788861	5.503366
H	-0.258836	3.437663	-4.354168
H	-0.646613	1.794119	-4.914811

H	0.184152	2.025319	-3.350980
H	0.851990	4.798728	4.022398
H	0.505214	4.562648	1.545844
H	-4.678181	-4.360311	1.650170
H	-5.151838	-2.910290	2.579351
H	-3.495275	-3.045034	1.940230
H	-0.908685	-2.642792	-5.045281
H	0.016937	-2.346041	-3.533518
H	-0.161818	-1.053124	-4.742685
H	-2.443545	2.578132	2.817989
H	-4.078400	2.800746	3.500253
H	-3.088685	4.223745	3.070551
H	-4.033407	3.082660	-3.801077
H	-3.077917	2.549730	-5.217677
H	-2.783258	4.161736	-4.489276
H	3.874196	-5.574564	1.663124
H	3.454721	-4.803722	0.112952
H	2.188422	-5.594049	1.086507
H	6.736672	-3.906750	-1.643093
H	5.281183	-4.865939	-2.007195
H	5.684586	-4.455469	-0.310089
H	2.197437	4.804316	-1.180690
H	1.350711	-4.473026	3.320434
H	2.256974	-3.077438	3.967662
H	3.043504	-4.671598	3.838310
H	-6.852763	-2.273476	-0.747438
H	-7.119300	-2.472218	1.012538
H	-6.702101	-3.887301	0.001109
H	-4.968291	5.122407	1.459874
H	-5.971351	3.755666	2.034401
H	-5.705592	4.004250	0.278118
H	-0.586062	0.849986	-1.618639
H	0.361669	0.541574	-1.041610

Table S47. Cartesian geometry of 3b-TS1 (23.2 kcal/mol) in Figure S145 in Angstrom [Å].

Atomtype	X Coordinates	Y Coordinates	Z Coordinates
C	-3.014024	-1.659184	3.771430
C	-2.866271	-0.864409	2.613341
C	-3.985529	-0.151938	2.143874
C	-5.210665	-0.191647	2.824079
C	-5.345667	-0.975882	3.981273
C	-4.245805	-1.717906	4.445567
P	-1.250423	-0.599037	1.707472
C	-0.233798	-2.070539	2.218656
Si	0.772007	-2.979426	0.849169
C	-0.550257	-3.405185	-0.486722
C	-1.773171	-4.119804	0.130015
Ni	-1.317229	0.157394	-0.374132
C	-3.011905	0.688435	-1.015147
N	-3.992980	-0.018915	-1.682354
C	-5.060539	0.809287	-2.051834
C	-4.750424	2.068055	-1.624911
N	-3.504260	1.983323	-1.003530
C	-3.920815	-1.405468	-2.086254
C	-3.028319	-1.771557	-3.135062
C	-3.079553	-3.100461	-3.595376
C	-3.981815	-4.027401	-3.054019
C	-4.834467	-3.647140	-2.014122
C	-4.814963	-2.334544	-1.498184
C	-2.087943	-0.753192	-3.781493

C	-0.857123	-1.375428	-4.460577
C	-5.754892	-1.969564	-0.350541
C	-7.227161	-1.954629	-0.816203
C	-2.712652	3.121812	-0.600246
C	-1.728820	3.598292	-1.511353
C	-0.912718	4.662496	-1.089675
C	-1.087429	5.251878	0.171475
C	-2.101548	4.803458	1.023142
C	-2.936700	3.727279	0.658052
C	-1.639619	3.064551	-2.940670
C	-2.483808	3.961965	-3.874623
C	-4.079059	3.306130	1.578396
C	-5.209467	4.359290	1.537564
C	-0.568175	0.797308	2.748983
C	-0.180083	2.002070	2.127987
C	0.274889	3.086806	2.895780
C	0.339223	2.982517	4.293659
C	-0.049255	1.785847	4.923135
C	-0.499619	0.701091	4.155561
C	-0.204222	2.913366	-3.474859
C	-3.620128	3.039792	3.025327
C	-5.570388	-2.910088	0.858151
C	-2.853912	0.147218	-4.776888
C	1.572142	-4.506438	1.729698
C	0.975309	-4.827165	3.117956
N	2.049595	-1.978346	0.147872
Ge	1.606556	-0.793410	-1.363493
C	2.964818	0.737199	-0.990125
C	4.359553	0.495182	-1.030634
C	5.291683	1.534629	-0.879256
C	4.862186	2.861582	-0.712844
C	3.486147	3.125915	-0.694983
C	2.551032	2.077025	-0.819861
C	6.774449	1.228576	-0.820558
F	7.089229	0.077875	-1.460647
C	2.983195	4.543149	-0.548754
F	2.065160	4.653601	0.453602
C	1.600978	-5.790741	0.873615
C	3.412533	-2.027051	0.585656
C	3.884248	-1.207437	1.657901
C	5.254554	-1.213289	1.982248
C	6.171445	-2.000509	1.278643
C	5.711298	-2.818543	0.240726
C	4.349657	-2.855288	-0.117318
C	2.941353	-0.344314	2.489091
C	3.402822	1.120942	2.618243
C	3.923179	-3.739295	-1.288939
C	4.537381	-5.153764	-1.232820
C	2.759289	-0.970401	3.889986
C	4.249219	-3.070612	-2.642600
C	-0.042265	-4.158915	-1.731552
F	7.515896	2.220839	-1.380331
F	7.203918	1.101745	0.464141
F	3.969281	5.429225	-0.283797
F	2.357718	4.973961	-1.684054
H	-0.911609	-2.409163	-0.833891
H	-5.924209	0.421529	-2.590338
H	-5.288566	3.011837	-1.707384
H	-3.877523	0.435890	1.227935
H	-2.090904	2.061398	-2.951949

H	-0.923447	-2.841490	2.602955
H	0.415091	-1.788061	3.061581
H	3.709277	-0.931770	4.456294
H	2.463333	-2.033441	3.824513
H	1.992570	-0.421781	4.467351
H	0.285819	-5.184340	-1.486759
H	0.803411	-3.638006	-2.215538
H	-0.852549	-4.242484	-2.480709
H	-0.425762	6.068532	0.484148
H	6.430050	-3.431506	-0.316653
H	1.963620	-0.334192	1.978299
H	2.623489	-4.186286	1.885470
H	-1.704310	-0.118446	-2.962376
H	-0.249339	2.098518	1.038022
H	7.238978	-1.967085	1.528984
H	-0.810348	-0.221855	4.659897
H	5.613747	-0.570791	2.795174
H	4.745207	-0.519920	-1.167523
H	-5.525107	-4.381778	-1.583689
H	-2.166521	-2.233006	4.165897
H	-2.244346	5.285003	1.997237
H	2.827237	-3.844997	-1.232528
H	-4.500659	2.367244	1.182843
H	-5.499615	-0.951811	-0.010193
H	1.485540	2.326664	-0.757772
H	-0.128635	5.040994	-1.751768
H	-4.007489	-5.054799	-3.438941
H	-6.064397	0.380825	2.438590
H	-2.173349	0.903761	-5.209104
H	-3.689761	0.682735	-4.294881
H	-3.265672	-0.458447	-5.606759
H	-2.530095	-4.329142	-0.648220
H	-2.273315	-3.505673	0.900340
H	-1.496642	-5.085527	0.596381
H	-2.409020	-3.416835	-4.400353
H	3.718184	-2.107179	-2.759866
H	3.949824	-3.720859	-3.487167
H	5.333799	-2.866635	-2.727081
H	-6.304388	-1.018822	4.513825
H	-4.343358	-2.341521	5.343785
H	2.636045	1.710668	3.150235
H	3.562658	1.578283	1.628917
H	4.346552	1.203511	3.189445
H	-0.001052	1.696739	6.016175
H	0.319805	3.882457	-3.558345
H	-0.227674	2.461599	-4.483756
H	0.398979	2.256275	-2.825658
H	0.694840	3.829546	4.894347
H	0.581318	4.007884	2.389973
H	-5.823761	-3.955791	0.603746
H	-6.225962	-2.594928	1.688635
H	-4.530835	-2.881899	1.225581
H	5.587576	3.672969	-0.602712
H	-1.124123	-1.921271	-5.385907
H	-0.325928	-2.062862	-3.781256
H	-0.149550	-0.574696	-4.741745
H	-2.797760	2.306982	3.057968
H	-4.459266	2.637655	3.621392
H	-3.270350	3.966722	3.516147
H	-3.531229	4.028247	-3.527452

H	-2.487808	3.554759	-4.903273
H	-2.070362	4.987721	-3.908857
H	2.195039	-6.579997	1.377244
H	2.046381	-5.631378	-0.121360
H	0.582701	-6.197335	0.723219
H	5.619431	-5.141309	-1.462365
H	4.052857	-5.810797	-1.979818
H	4.409619	-5.610035	-0.235281
H	-0.091095	-5.120037	3.045927
H	1.040879	-3.975416	3.818408
H	1.512796	-5.677934	3.583550
H	-7.382031	-1.258161	-1.660628
H	-7.892022	-1.644620	0.012140
H	-7.546708	-2.960192	-1.149111
H	-4.851516	5.334949	1.916970
H	-6.061266	4.039556	2.167659
H	-5.579226	4.515439	0.506813
H	-0.635435	1.063437	-1.331882
H	0.197146	0.517469	-0.651507

Table S48. Cartesian geometry of 3c-TS1 (24.7 kcal/mol) in Figure S145 in Angstrom [Å].

Atomtype	X Coordinates	Y Coordinates	Z Coordinates
C	4.181838	2.163221	-1.805357
C	2.807750	1.848580	-1.661391
C	1.893526	2.933159	-1.679848
C	2.326839	4.263787	-1.813178
C	3.697172	4.549961	-1.936979
C	4.622191	3.492356	-1.936188
Ge	2.138133	-0.102712	-1.633788
N	3.014819	-0.743290	0.027980
C	4.345821	-0.280135	0.275033
C	4.605941	0.826207	1.142998
C	5.925182	1.294142	1.295549
C	6.994382	0.713988	0.604817
C	6.746515	-0.372475	-0.242613
C	5.447861	-0.889848	-0.414532
C	3.496818	1.506108	1.937468
C	3.463054	3.035274	1.750804
C	5.253010	-2.065560	-1.371642
C	6.281639	-3.196346	-1.155754
Si	2.166075	-1.879673	1.075027
C	0.969671	-2.984121	0.040888
C	0.094132	-3.885658	0.939799
C	0.997957	-1.045272	2.367394
P	-0.456149	-0.083911	1.725882
C	-1.826827	-0.529408	2.919355
C	-3.144166	-0.251769	2.507502
C	-4.230578	-0.439531	3.373158
C	-4.017185	-0.939502	4.668676
C	-2.710740	-1.250002	5.083822
C	-1.622275	-1.041852	4.218960
C	-0.143683	1.662819	2.316775
C	-0.184133	2.721096	1.387600
C	-0.033289	4.050645	1.813199
C	0.148723	4.339272	3.174017
C	0.190366	3.289203	4.109664
C	0.046408	1.960146	3.683421
Ni	-0.942138	0.051976	-0.425646
C	-2.755713	-0.059309	-0.903687
N	-3.596250	1.019790	-1.130165

C	-4.854192	0.608922	-1.574646
C	-4.817118	-0.754084	-1.647317
N	-3.533440	-1.147037	-1.247135
C	-3.121271	2.383501	-1.132209
C	-2.380745	2.824061	-2.265027
C	-1.840912	4.122378	-2.225934
C	-2.064599	4.964932	-1.126126
C	-2.851422	4.529263	-0.054533
C	-3.393224	3.227611	-0.030588
C	-3.074832	-2.515854	-1.331192
C	-3.578500	-3.462887	-0.406002
C	-3.233830	-4.817248	-0.595982
C	-2.395490	-5.208797	-1.643750
C	-1.872546	-4.247400	-2.520683
C	-2.201266	-2.884935	-2.394759
C	-2.275901	1.970175	-3.527449
C	-3.430834	2.334043	-4.489008
C	-4.297111	2.797964	1.121566
C	-5.678304	3.479645	0.993023
C	-4.471932	-3.072879	0.769545
C	-5.918329	-3.572398	0.564413
C	-1.680228	-1.855054	-3.397777
C	-0.359097	-2.246441	-4.079014
C	-0.917113	2.057849	-4.244291
C	-3.675328	3.057804	2.507401
C	-3.898328	-3.576842	2.109791
C	-2.758486	-1.526149	-4.454248
C	3.460961	-2.853538	2.133017
C	3.047567	-3.087415	3.602405
C	3.905373	-4.190253	1.502765
C	1.614531	-3.806368	-1.092069
C	3.626376	1.149152	3.434949
C	5.281279	-1.600638	-2.844300
H	0.277998	-2.252829	-0.438937
H	-5.568768	-1.481169	-1.952364
H	-5.646993	1.321936	-1.798955
H	-3.301355	0.112449	1.487757
H	-2.414705	0.920509	-3.230862
H	0.600227	-1.895621	2.948190
H	1.563752	-0.422701	3.077756
H	4.551793	1.584204	3.858441
H	3.679841	0.055853	3.589632
H	2.769050	1.548158	4.008396
H	2.286586	-4.590185	-0.701095
H	2.197029	-3.171409	-1.783303
H	0.830237	-4.313681	-1.686714
H	-1.631788	5.973660	-1.114150
H	7.582327	-0.830223	-0.786504
H	2.536475	1.115292	1.562695
H	4.341799	-2.177792	2.137070
H	-1.467008	-0.939524	-2.818422
H	-0.351429	2.501381	0.327714
H	8.013681	1.103266	0.726969
H	0.066078	1.151865	4.424533
H	6.115290	2.142690	1.964753
H	4.935877	1.366300	-1.798298
H	-3.621352	-5.571584	0.098871
H	-0.610086	-1.273050	4.573134
H	-3.038020	5.202103	0.790008
H	4.249483	-2.478393	-1.174935

H	5.696887	3.700719	-2.027254
H	-4.464649	1.711875	1.027790
H	-4.500586	-1.971199	0.824069
H	0.817147	2.753350	-1.574447
H	-1.247493	4.485670	-3.072226
H	-2.131923	-6.266470	-1.771328
H	-5.246879	-0.208408	3.027958
H	-2.387371	-0.752365	-5.151363
H	-3.687348	-1.147442	-3.993810
H	-3.008887	-2.428391	-5.044504
H	-0.598623	-4.487087	0.322569
H	-0.532768	-3.302982	1.638624
H	0.703965	-4.589528	1.539521
H	-1.202661	-4.566372	-3.325369
H	4.498021	-0.847289	-3.049841
H	5.117102	-2.453384	-3.531244
H	6.258404	-1.141176	-3.088917
H	-4.864139	-1.097536	5.348670
H	-2.534828	-1.650090	6.090958
H	2.605707	3.459836	2.300538
H	3.359724	3.303523	0.687270
H	4.381251	3.514420	2.140603
H	0.335593	3.506616	5.175786
H	-0.740952	3.057518	-4.683139
H	-0.886044	1.327333	-5.073426
H	-0.081108	1.832644	-3.560666
H	0.265502	5.378257	3.508075
H	-0.060699	4.856358	1.070982
H	-3.857261	-4.681130	2.146695
H	-4.529671	-3.232878	2.947480
H	-2.881139	-3.183274	2.276110
H	4.041699	5.588519	-2.030720
H	-0.486830	-3.088725	-4.785936
H	0.411770	-2.514932	-3.337996
H	0.023886	-1.385734	-4.656383
H	-2.677028	2.599166	2.592966
H	-4.317719	2.627925	3.297130
H	-3.571593	4.140044	2.708719
H	-4.414381	2.209244	-4.000493
H	-3.407172	1.684228	-5.383947
H	-3.345806	3.386006	-4.821674
H	4.763998	-4.618399	2.058868
H	4.215523	-4.083048	0.451042
H	3.090918	-4.939083	1.536551
H	7.289360	-2.906616	-1.508884
H	5.983732	-4.098309	-1.723555
H	6.365895	-3.469839	-0.089248
H	1.588919	5.078535	-1.808150
H	2.139726	-3.718808	3.675130
H	2.843074	-2.145875	4.143001
H	3.852686	-3.614901	4.153290
H	-6.354172	-3.184424	-0.374622
H	-6.563017	-3.248059	1.403217
H	-5.951864	-4.677412	0.518132
H	-5.581878	4.579296	1.068415
H	-6.355775	3.140042	1.799497
H	-6.153231	3.250119	0.020756
H	-0.635598	0.775222	-1.679332
H	0.365364	0.668743	-1.047583

Table S49. Cartesian geometry of 3d-TS1 (24.6 kcal/mol) in Figure S145 in Angstrom [Å].

Atomtype	X Coordinates	Y Coordinates	Z Coordinates
C	-1.839410	-1.272688	4.126542
C	-1.979035	-0.650597	2.866782
C	-3.259997	-0.224287	2.467697
C	-4.370560	-0.373961	3.309810
C	-4.220910	-0.983981	4.566538
C	-2.953819	-1.442310	4.966482
P	-0.557270	-0.247229	1.718605
C	0.793684	-1.379585	2.302557
Si	1.914614	-2.214487	0.968742
C	0.650987	-3.133142	-0.162399
C	-0.314586	-4.019405	0.656003
Ni	-0.997574	0.073255	-0.421006
C	-2.806681	0.162648	-0.915177
N	-3.672376	-0.824636	-1.341903
C	-4.909148	-0.291001	-1.726748
C	-4.825683	1.061650	-1.559383
N	-3.543494	1.325996	-1.074219
C	-3.334470	-2.219793	-1.516894
C	-2.481518	-2.591277	-2.596266
C	-2.269156	-3.965582	-2.811812
C	-2.884819	-4.934549	-2.006346
C	-3.700586	-4.542848	-0.940891
C	-3.929985	-3.179481	-0.662015
C	-1.860160	-1.545631	-3.522547
C	-0.564161	-2.000438	-4.212703
C	-4.802551	-2.792055	0.529789
C	-6.285020	-3.140526	0.275584
C	-2.946743	2.637547	-0.977691
C	-2.152519	3.084581	-2.070935
C	-1.497050	4.321975	-1.937489
C	-1.659491	5.104726	-0.784164
C	-2.498460	4.671222	0.247952
C	-3.156848	3.426236	0.177351
C	-2.107516	2.313526	-3.389014
C	-3.212469	2.844885	-4.331169
C	-4.112074	3.003856	1.289971
C	-5.422744	3.817997	1.200362
C	-0.101307	1.419793	2.432331
C	-0.018166	2.533893	1.574040
C	0.239924	3.814216	2.089406
C	0.406276	3.998170	3.470257
C	0.326096	2.891452	4.335555
C	0.075724	1.610817	3.819666
C	-0.736946	2.328580	-4.088463
C	-3.487083	3.108510	2.694701
C	-4.299330	-3.438776	1.836517
C	-2.889352	-1.058114	-4.566453
C	3.095705	-3.370886	1.974143
C	2.623612	-3.687975	3.409820
N	2.882864	-1.087732	0.020750
Ge	2.103584	-0.256709	-1.607629
C	2.917447	1.628744	-1.461186
C	4.313712	1.855987	-1.537068
C	4.850072	3.153938	-1.531584
C	4.018208	4.290072	-1.455854
C	2.625642	4.077488	-1.407249
C	2.090387	2.778635	-1.412733
C	4.605699	5.683432	-1.388087

C	3.450949	-4.684049	1.245572
C	4.242054	-0.759062	0.322678
C	4.575774	0.250067	1.279822
C	5.925984	0.590144	1.489177
C	6.957580	-0.022119	0.769708
C	6.637624	-1.012650	-0.166382
C	5.304292	-1.402987	-0.397996
C	3.511582	0.958769	2.109724
C	3.611764	2.495151	2.046934
C	5.034210	-2.478772	-1.449749
C	5.955851	-3.708955	-1.304663
C	3.577289	0.474728	3.575372
C	5.143092	-1.904770	-2.879618
C	1.247512	-3.924981	-1.342703
H	0.034820	-2.311614	-0.597543
H	-5.717641	-0.924555	-2.089588
H	-5.548082	1.856875	-1.741194
H	-3.369324	0.225946	1.476490
H	-2.343079	1.262467	-3.167855
H	0.308990	-2.234166	2.805965
H	1.395032	-0.868929	3.070465
H	4.525978	0.794691	4.047225
H	3.535900	-0.627951	3.641785
H	2.743510	0.898449	4.165734
H	1.840028	-4.791598	-1.000462
H	1.897958	-3.295460	-1.975832
H	0.435527	-4.317162	-1.985540
H	-1.136592	6.066117	-0.698681
H	7.443533	-1.495073	-0.733612
H	2.529965	0.683943	1.690167
H	4.026655	-2.770988	2.053330
H	-1.581492	-0.691699	-2.880874
H	-0.171840	2.397819	0.498258
H	8.003043	0.268001	0.937898
H	0.000029	0.758929	4.506314
H	6.172049	1.363685	2.227487
H	5.010863	1.010074	-1.584753
H	-4.161011	-5.305644	-0.302048
H	-0.857318	-1.620814	4.469623
H	-2.635308	5.299426	1.135323
H	3.993897	-2.815844	-1.306513
H	5.940676	3.286121	-1.574380
H	-4.377776	1.947110	1.119092
H	-4.730909	-1.698891	0.661611
H	0.999666	2.677016	-1.365535
H	-0.860061	4.685792	-2.751225
H	-2.711388	-6.000169	-2.203327
H	-5.356905	-0.026171	2.975704
H	-2.443265	-0.274473	-5.206334
H	-3.790448	-0.632848	-4.091580
H	-3.204957	-1.895107	-5.218378
H	-1.033829	-4.525729	-0.013896
H	-0.914682	-3.435830	1.377195
H	0.224454	-4.804423	1.221874
H	-1.618129	-4.286789	-3.631021
H	4.437117	-1.068522	-3.039106
H	4.920936	-2.682515	-3.635777
H	6.163896	-1.519651	-3.067783
H	-5.087795	-1.112640	5.227345
H	-2.828562	-1.928940	5.942622

H	2.788081	2.946636	2.625538
H	3.543267	2.855461	1.008040
H	4.563258	2.860164	2.478402
H	0.458865	3.026697	5.416795
H	-0.468693	3.335889	-4.457689
H	-0.758953	1.655003	-4.964737
H	0.067248	1.985844	-3.415582
H	0.605970	4.998831	3.874688
H	0.308660	4.663992	1.401036
H	-4.362081	-4.541961	1.796723
H	-4.908828	-3.095400	2.690429
H	-3.252478	-3.155190	2.039124
H	-0.748870	-2.777795	-4.978937
H	0.167425	-2.384712	-3.482898
H	-0.099265	-1.137465	-4.722639
H	-2.535750	2.555643	2.754107
H	-4.175831	2.685281	3.448132
H	-3.287954	4.160073	2.972402
H	-4.209757	2.774760	-3.860003
H	-3.234174	2.259189	-5.269372
H	-3.030046	3.905601	-4.588350
H	4.259458	-5.222394	1.780672
H	3.794458	-4.518003	0.212017
H	2.579980	-5.365896	1.202938
H	6.993708	-3.481883	-1.613637
H	5.596799	-4.534364	-1.948476
H	5.988374	-4.071088	-0.262035
H	1.946326	4.941132	-1.353031
H	1.665173	-4.244314	3.410868
H	2.482878	-2.779947	4.023215
H	3.366983	-4.324743	3.931304
H	-6.668535	-2.649054	-0.637472
H	-6.910953	-2.818010	1.129183
H	-6.418104	-4.231812	0.150096
H	-5.226487	4.896137	1.352981
H	-6.138908	3.487964	1.976819
H	-5.904186	3.700962	0.211403
H	-0.612115	0.821969	-1.635990
H	0.367062	0.580085	-1.016532
H	5.516269	5.771872	-2.009856
H	3.882317	6.449630	-1.723380
H	4.897909	5.939122	-0.349004

Table S50. Cartesian geometry of 3e-TS1 (25.3 kcal/mol) in Figure S145 in Angstrom [Å].

Atomtype	X Coordinates	Y Coordinates	Z Coordinates
C	-2.141944	-1.328339	4.051639
C	-2.186057	-0.637037	2.821284
C	-3.405880	-0.059937	2.419807
C	-4.545179	-0.129915	3.233420
C	-4.489570	-0.809738	4.461678
C	-3.287220	-1.417749	4.861903
P	-0.704258	-0.335766	1.718634
C	0.505675	-1.633819	2.268482
Si	1.555195	-2.521825	0.910737
C	0.219532	-3.241992	-0.280445
C	-0.853755	-4.052839	0.479600
Ni	-1.060977	0.136788	-0.408837
C	-2.838003	0.442553	-0.934666
N	-3.795822	-0.421627	-1.426316
C	-4.957854	0.262507	-1.805986

C	-4.731568	1.588133	-1.568876
N	-3.440194	1.686289	-1.047821
C	-3.606943	-1.834866	-1.666595
C	-2.773558	-2.243695	-2.747793
C	-2.706172	-3.620719	-3.029887
C	-3.442366	-4.554997	-2.286996
C	-4.236726	-4.129356	-1.218426
C	-4.323727	-2.764466	-0.873823
C	-2.020859	-1.227950	-3.607835
C	-0.767501	-1.788585	-4.298825
C	-5.177651	-2.343757	0.320349
C	-6.682271	-2.522221	0.023820
C	-2.706134	2.917887	-0.874805
C	-1.844360	3.327627	-1.930899
C	-1.059723	4.476470	-1.723355
C	-1.159035	5.213621	-0.533334
C	-2.062460	4.825048	0.461728
C	-2.852632	3.666176	0.316277
C	-1.857150	2.622306	-3.286035
C	-2.870346	3.325044	-4.219073
C	-3.873283	3.297767	1.389361
C	-5.085602	4.253699	1.316614
C	-0.084723	1.235436	2.522107
C	0.139550	2.373337	1.722229
C	0.523576	3.591462	2.305350
C	0.677169	3.689743	3.696388
C	0.457829	2.558262	4.503612
C	0.081078	1.339054	3.920052
C	-0.478189	2.510494	-3.959684
C	-3.274474	3.265807	2.808852
C	-4.772933	-3.100563	1.601967
C	-2.967031	-0.579487	-4.642724
C	2.587715	-3.846256	1.871514
C	2.063705	-4.173360	3.286630
N	2.657137	-1.463611	0.032361
Ge	1.998970	-0.471768	-1.561762
C	3.012536	1.300849	-1.323699
C	4.423494	1.377695	-1.360886
C	5.115704	2.601981	-1.295983
C	4.395250	3.809765	-1.195581
C	2.983702	3.766526	-1.178448
C	2.317278	2.537682	-1.244733
O	4.960032	5.052916	-1.109371
C	6.372567	5.156071	-1.083831
C	2.807741	-5.156224	1.086043
C	4.036854	-1.298098	0.367923
C	4.459389	-0.376132	1.376654
C	5.834408	-0.192301	1.618160
C	6.807434	-0.876689	0.881985
C	6.400209	-1.782774	-0.104660
C	5.037100	-2.017499	-0.370149
C	3.461789	0.402938	2.226005
C	3.721714	1.921545	2.230415
C	4.670542	-3.007512	-1.475354
C	5.452202	-4.335381	-1.379897
C	3.453577	-0.146649	3.669746
C	4.861833	-2.381513	-2.874361
C	0.748030	-4.038437	-1.489403
H	-0.293756	-2.337761	-0.683574
H	-5.821833	-0.260189	-2.214729

H	-5.358771	2.465677	-1.723174
H	-3.445258	0.443304	1.449010
H	-2.218297	1.596556	-3.123154
H	-0.077346	-2.452987	2.724246
H	1.142456	-1.225579	3.068578
H	4.422227	0.053372	4.166585
H	3.297480	-1.240907	3.688137
H	2.658120	0.336175	4.267594
H	1.235205	-4.979851	-1.180741
H	1.475727	-3.455670	-2.081826
H	-0.089543	-4.307985	-2.161766
H	-0.534579	6.104879	-0.389694
H	7.159907	-2.321692	-0.684746
H	2.463664	0.250024	1.783892
H	3.577229	-3.356047	1.986369
H	-1.664859	-0.442464	-2.918439
H	-0.001766	2.305980	0.638360
H	7.874843	-0.708600	1.076927
H	-0.101710	0.468503	4.561646
H	6.148443	0.514678	2.396468
H	5.026505	0.463396	-1.424774
H	-4.792403	-4.867819	-0.628596
H	-1.210227	-1.794690	4.394976
H	-2.147591	5.419417	1.378392
H	3.597964	-3.236463	-1.359431
H	6.210592	2.593414	-1.310082
H	-4.247540	2.285699	1.161044
H	-4.996236	-1.271496	0.507257
H	1.221432	2.558696	-1.218081
H	-0.369107	4.807494	-2.506833
H	-3.380579	-5.622216	-2.535335
H	-5.481705	0.335069	2.898485
H	-2.424070	0.181314	-5.233371
H	-3.827391	-0.082230	-4.162380
H	-3.356567	-1.343654	-5.342360
H	-1.615088	-4.437440	-0.223970
H	-1.394552	-3.442780	1.225377
H	-0.416916	-4.923009	1.007837
H	-2.074179	-3.970516	-3.852261
H	4.251300	-1.467819	-2.999641
H	4.569415	-3.094156	-3.669751
H	5.920657	-2.099627	-3.033426
H	-5.380288	-0.876331	5.099573
H	-3.236463	-1.959766	5.815325
H	2.946530	2.431951	2.826633
H	3.696310	2.330737	1.207830
H	4.703755	2.166849	2.677844
H	0.580362	2.626088	5.592388
H	-0.080647	3.496892	-4.262548
H	-0.560292	1.896301	-4.875183
H	0.261677	2.032936	-3.295339
H	0.975541	4.641915	4.153642
H	0.701577	4.460164	1.661642
H	-4.945813	-4.188368	1.505650
H	-5.364041	-2.738172	2.460972
H	-3.707779	-2.934952	1.837248
H	-1.019237	-2.502923	-5.106229
H	-0.098578	-2.285559	-3.577103
H	-0.199954	-0.958593	-4.756871
H	-2.391386	2.608649	2.858370

H	-4.023520	2.886001	3.527125
H	-2.967881	4.274336	3.142625
H	-3.879631	3.350593	-3.769301
H	-2.938757	2.791951	-5.185992
H	-2.559230	4.367987	-4.419064
H	3.545189	-5.803547	1.602786
H	3.181532	-4.982279	0.064296
H	1.868326	-5.735957	1.003909
H	6.513551	-4.207765	-1.665370
H	5.017349	-5.085791	-2.067282
H	5.427669	-4.747733	-0.355835
H	2.431185	4.711266	-1.098231
H	1.051336	-4.622692	3.252668
H	2.011608	-3.282883	3.938492
H	2.726743	-4.908643	3.786430
H	-6.991758	-1.950479	-0.870418
H	-7.290125	-2.176264	0.881293
H	-6.925735	-3.586244	-0.157636
H	-4.777026	5.295327	1.526363
H	-5.851509	3.967457	2.062397
H	-5.553394	4.237421	0.314392
H	-0.565946	0.913063	-1.566010
H	0.367532	0.537961	-0.930131
H	6.609164	6.230508	-0.994038
H	6.811387	4.615038	-0.218385
H	6.834938	4.761607	-2.013875

Table S51. Cartesian geometry of 3f-TS1 (25.5 kcal/mol) in Figure S145 in Angstrom [Å].

Atomtype	X Coordinates	Y Coordinates	Z Coordinates
C	-2.284828	-1.263767	4.042338
C	-2.270730	-0.546837	2.826295
C	-3.425438	0.168349	2.455497
C	-4.554373	0.205418	3.285423
C	-4.556521	-0.502613	4.499190
C	-3.422163	-1.245595	4.868606
P	-0.779790	-0.380949	1.706714
C	0.306517	-1.790284	2.234794
Si	1.281093	-2.747596	0.867064
C	-0.110779	-3.337427	-0.331731
C	-1.253044	-4.057705	0.419612
Ni	-1.122882	0.130089	-0.412413
C	-2.863796	0.617048	-0.907792
N	-3.902463	-0.149856	-1.396827
C	-4.993541	0.644224	-1.772941
C	-4.638803	1.941606	-1.536617
N	-3.342250	1.913592	-1.018905
C	-3.848990	-1.574586	-1.638198
C	-3.071857	-2.058732	-2.729862
C	-3.133136	-3.436531	-3.009346
C	-3.940888	-4.298944	-2.253991
C	-4.677575	-3.801686	-1.174908
C	-4.634699	-2.434310	-0.832126
C	-2.243010	-1.115778	-3.602246
C	-1.042799	-1.782429	-4.293421
C	-5.427761	-1.934124	0.373005
C	-6.945682	-1.953389	0.091352
C	-2.486661	3.065332	-0.855855
C	-1.599386	3.385834	-1.921622
C	-0.698823	4.448038	-1.723237
C	-0.710117	5.191427	-0.533158

C	-1.638475	4.897680	0.471470
C	-2.543577	3.824961	0.335656
C	-1.700265	2.688380	-3.277105
C	-2.645521	3.494845	-4.197591
C	-3.584962	3.561561	1.419691
C	-4.696002	4.634212	1.357475
C	-0.013482	1.122912	2.510910
C	0.322635	2.230759	1.708634
C	0.813223	3.410635	2.290403
C	0.960854	3.501676	3.682486
C	0.632027	2.398655	4.492172
C	0.150997	1.216140	3.909852
C	-0.348069	2.436030	-3.966885
C	-2.977486	3.471184	2.832925
C	-5.092293	-2.735225	1.647491
C	-3.138348	-0.397991	-4.636304
C	2.188229	-4.167174	1.818830
C	1.616584	-4.480388	3.218611
N	2.474474	-1.787736	0.000122
Ge	1.915324	-0.727930	-1.593352
C	3.028006	0.963945	-1.307370
C	4.444293	0.972336	-1.302483
C	5.191431	2.152216	-1.185060
C	4.554729	3.420621	-1.067695
C	3.130973	3.428168	-1.095226
C	2.407659	2.234557	-1.220310
N	5.287424	4.596372	-0.924740
C	4.594157	5.844080	-0.666929
C	2.317169	-5.473449	1.007816
C	3.862382	-1.739711	0.337423
C	4.357635	-0.876987	1.365925
C	5.742620	-0.809420	1.610239
C	6.658808	-1.551344	0.857211
C	6.181002	-2.398651	-0.149553
C	4.803636	-2.518601	-0.418213
C	3.426242	-0.038340	2.233659
C	3.810082	1.452944	2.274710
C	4.362875	-3.453698	-1.544155
C	5.025417	-4.846066	-1.464957
C	3.367633	-0.620078	3.663527
C	4.623620	-2.823078	-2.929842
C	0.347333	-4.167162	-1.547411
C	6.726810	4.527027	-0.744468
H	-0.541337	-2.387629	-0.726722
H	-5.905866	0.208348	-2.178477
H	-5.178392	2.875923	-1.689043
H	-3.420911	0.694995	1.496230
H	-2.164532	1.705368	-3.111747
H	-0.351577	-2.564186	2.666910
H	0.969494	-1.458614	3.049058
H	4.348531	-0.515546	4.165671
H	3.118442	-1.697036	3.654798
H	2.614723	-0.085851	4.273115
H	0.751759	-5.149788	-1.247213
H	1.122122	-3.644067	-2.135796
H	-0.510488	-4.358812	-2.221414
H	0.003919	6.014030	-0.396478
H	6.895461	-2.982441	-0.743641
H	2.420554	-0.096246	1.786865
H	3.211499	-3.759346	1.958732

H	-1.819162	-0.358580	-2.920616
H	0.187065	2.170552	0.623865
H	7.736147	-1.472737	1.054177
H	-0.117291	0.368901	4.552680
H	6.111450	-0.147331	2.403857
H	5.002556	0.030056	-1.371348
H	-5.289328	-4.485999	-0.575380
H	-1.403667	-1.833328	4.362594
H	-1.653502	5.497650	1.388279
H	3.273240	-3.590682	-1.441342
H	6.282566	2.071792	-1.165233
H	-4.061219	2.591923	1.196521
H	-5.132816	-0.887679	0.562019
H	1.314327	2.318934	-1.230203
H	0.015019	4.704732	-2.513643
H	-3.979874	-5.367700	-2.500370
H	-5.439039	0.776909	2.975057
H	-2.538485	0.313777	-5.233169
H	-3.952951	0.169581	-4.153826
H	-3.592415	-1.131009	-5.330393
H	-2.037946	-4.378772	-0.289839
H	-1.748782	-3.404741	1.160523
H	-0.893848	-4.961049	0.950796
H	-2.546340	-3.843050	-3.839184
H	4.102874	-1.853658	-3.040923
H	4.272238	-3.490394	-3.740587
H	5.705515	-2.639028	-3.076620
H	-5.440563	-0.485644	5.149527
H	-3.417635	-1.809515	5.810617
H	3.083215	2.008636	2.890602
H	3.812284	1.888505	1.262793
H	4.811667	1.605506	2.720213
H	0.750416	2.460260	5.581786
H	0.144811	3.376913	-4.274763
H	-0.503805	1.834567	-4.881227
H	0.347123	1.884995	-3.311366
H	1.340684	4.424734	4.139220
H	1.079643	4.254250	1.643925
H	-5.386302	-3.796851	1.552265
H	-5.628369	-2.312209	2.514759
H	-4.012317	-2.689845	1.869059
H	-1.354873	-2.485007	-5.089957
H	-0.410046	-2.321099	-3.568906
H	-0.414889	-1.005615	-4.765846
H	-2.164849	2.728242	2.875129
H	-3.753412	3.170561	3.560115
H	-2.566988	4.444081	3.160811
H	-3.641113	3.625315	-3.735683
H	-2.780844	2.973310	-5.163784
H	-2.229269	4.499832	-4.400974
H	2.998099	-6.185845	1.516549
H	2.713302	-5.305997	-0.006595
H	1.337272	-5.978096	0.904954
H	6.097610	-4.805405	-1.735241
H	4.536629	-5.543627	-2.171599
H	4.952100	-5.273180	-0.449302
H	2.570641	4.364433	-1.005039
H	0.571077	-4.843199	3.160633
H	1.628301	-3.603146	3.890154
H	2.208524	-5.278925	3.710746

H	-7.203161	-1.346394	-0.796066
H	-7.505950	-1.552388	0.957262
H	-7.299373	-2.985405	-0.093889
H	-4.282385	5.639782	1.562519
H	-5.479231	4.426750	2.111306
H	-5.173116	4.664205	0.359977
H	-0.589463	0.813671	-1.605218
H	0.319190	0.324709	-1.009159
H	7.138304	5.544778	-0.640815
H	7.016013	3.943626	0.158238
H	7.224113	4.054120	-1.615959
H	5.324411	6.665574	-0.579126
H	3.899749	6.103781	-1.492849
H	3.994423	5.815101	0.271597

Table S52. Cartesian geometry of 3g-TS1 (23.2 kcal/mol) in Figure S145 in Angstrom [Å].

Atomtype	X Coordinates	Y Coordinates	Z Coordinates
C	1.428844	0.740749	-4.270626
C	1.690946	0.667303	-2.884978
C	3.030993	0.617632	-2.457359
C	4.089084	0.683829	-3.374390
C	3.819883	0.770546	-4.750575
C	2.486505	0.786771	-5.195508
P	0.383001	0.710361	-1.547030
C	-1.156071	0.204542	-2.458271
Si	-2.409670	-0.928421	-1.526953
C	-1.333737	-2.478580	-1.083617
C	-0.505673	-2.976006	-2.291416
Ni	0.850637	-0.073195	0.448588
C	2.645505	-0.521042	0.794313
N	3.325697	-1.718111	0.675629
C	4.634291	-1.617934	1.166236
C	4.787889	-0.340832	1.623719
N	3.574754	0.313307	1.398682
C	2.754577	-2.967128	0.224800
C	1.844336	-3.658534	1.076522
C	1.397863	-4.925267	0.655617
C	1.840459	-5.494107	-0.547447
C	2.717258	-4.786405	-1.374226
C	3.180253	-3.503272	-1.015638
C	1.391897	-3.068746	2.413203
C	0.068172	-3.646129	2.940275
C	4.115133	-2.751698	-1.961065
C	5.515904	-3.400463	-1.994776
C	3.238681	1.618077	1.918153
C	2.516984	1.683607	3.142534
C	2.157159	2.956446	3.621850
C	2.525940	4.121294	2.933050
C	3.273132	4.031407	1.754048
C	3.642748	2.780541	1.218876
C	2.228682	0.435465	3.973438
C	3.335677	0.252249	5.035980
C	4.497541	2.722056	-0.043631
C	5.948752	3.150759	0.268594
C	0.216782	2.568122	-1.379346
C	0.314417	3.155084	-0.100599
C	0.295118	4.552281	0.044736
C	0.186891	5.379570	-1.083725
C	0.079372	4.802567	-2.362358
C	0.091511	3.406665	-2.507969

C	0.829685	0.427744	4.617286
C	3.906447	3.555092	-1.198441
C	3.523264	-2.644046	-3.381392
C	2.495131	-3.201062	3.486742
C	-3.808976	-1.290214	-2.810250
C	-3.406654	-1.083863	-4.286614
N	-3.132661	-0.225841	-0.065954
Ge	-2.285819	-0.733421	1.671973
N	-2.730017	0.750707	2.747107
C	-2.824683	2.121973	2.298431
C	-4.450119	-2.683526	-2.641813
C	-4.407089	0.434162	-0.043403
C	-4.578358	1.776324	-0.528592
C	-5.836738	2.401858	-0.430610
C	-6.938209	1.760489	0.143708
C	-6.787207	0.447408	0.599214
C	-5.559376	-0.235537	0.500549
C	-3.456324	2.564992	-1.202692
C	-3.300564	4.011526	-0.684022
C	-5.530658	-1.688299	0.980093
C	-6.645882	-2.544039	0.336837
C	-3.677927	2.609567	-2.731869
C	-5.620858	-1.785982	2.519439
C	-2.930300	0.571604	4.170683
C	-2.056405	-3.674009	-0.427883
H	-0.596401	-2.089778	-0.343185
H	5.317028	-2.466694	1.154189
H	5.635103	0.161593	2.089843
H	3.225548	0.519624	-1.384694
H	2.272800	-0.430262	3.297365
H	-0.838162	-0.414289	-3.315461
H	-1.637652	1.094435	-2.889800
H	-4.594174	3.183709	-2.966855
H	-3.806453	1.601826	-3.159860
H	-2.827386	3.105489	-3.238088
H	-2.707122	-4.200521	-1.147229
H	-2.677518	-3.377422	0.435697
H	-1.312051	-4.408393	-0.063565
H	2.235850	5.104749	3.325249
H	-7.650406	-0.069834	1.037065
H	-2.509666	2.037908	-0.997568
H	-4.584553	-0.536373	-2.559681
H	1.212296	-1.994115	2.227423
H	0.424652	2.513205	0.782422
H	-7.904998	2.274101	0.225863
H	0.026111	2.970575	-3.512147
H	-5.952939	3.424449	-0.810645
H	3.042954	-5.228618	-2.323083
H	0.396444	0.772167	-4.640417
H	3.567825	4.946700	1.228214
H	-4.563326	-2.118617	0.666698
H	4.536692	1.670313	-0.372845
H	4.229085	-1.723107	-1.578206
H	1.593640	3.038455	4.558172
H	1.484717	-6.488595	-0.845768
H	5.125212	0.650474	-3.012733
H	2.161188	-2.737424	4.433461
H	3.433505	-2.706174	3.184391
H	2.715422	-4.267426	3.686281
H	0.099987	-3.854818	-2.001395

H	0.204560	-2.216908	-2.663612
H	-1.152789	-3.281290	-3.137313
H	0.696706	-5.484635	1.282674
H	-4.831949	-1.189735	3.009546
H	-5.523767	-2.836903	2.853979
H	-6.596271	-1.401978	2.875099
H	4.643322	0.812493	-5.475127
H	2.266621	0.842760	-6.269752
H	-2.383742	4.459542	-1.103140
H	-3.231981	4.055942	0.415154
H	-4.152022	4.646859	-0.992463
H	-0.012255	5.442272	-3.249740
H	0.735431	1.184570	5.418708
H	0.635085	-0.559317	5.076652
H	0.041624	0.613601	3.866497
H	0.178971	6.471352	-0.970552
H	0.380349	4.987733	1.047383
H	3.395219	-3.638619	-3.847101
H	4.192679	-2.051571	-4.029018
H	2.543498	-2.136984	-3.363316
H	0.177934	-4.693812	3.281338
H	-0.726991	-3.600554	2.177636
H	-0.271768	-3.050417	3.806793
H	2.858388	3.277928	-1.398246
H	4.486065	3.387489	-2.124092
H	3.935970	4.637139	-0.972569
H	4.333738	0.183569	4.565274
H	3.165850	-0.674276	5.616101
H	3.348527	1.104882	5.741445
H	-5.389844	-2.757698	-3.225665
H	-4.691061	-2.917530	-1.592357
H	-3.773884	-3.476900	-3.013592
H	-7.641628	-2.284316	0.742871
H	-6.475360	-3.617247	0.547076
H	-6.685629	-2.406072	-0.757382
H	-2.600740	-1.781705	-4.588055
H	-3.051990	-0.059089	-4.497020
H	-4.269355	-1.277637	-4.956138
H	5.968249	-3.445881	-0.987114
H	6.194854	-2.823853	-2.651364
H	5.462429	-4.434356	-2.385494
H	5.982648	4.200142	0.617702
H	6.580436	3.071814	-0.636693
H	6.395148	2.518932	1.059117
H	0.515274	0.028958	1.878987
H	-0.488055	0.348684	1.108214
H	-3.970497	0.826051	4.483439
H	-2.750293	-0.482718	4.463248
H	-2.242528	1.209504	4.773993
H	-3.864797	2.517983	2.324916
H	-2.191900	2.791811	2.927623
H	-2.458797	2.202167	1.264339

Table S53. Cartesian geometry of 3a-INT2 (18.9 kcal/mol) in Figure S145 in Angstrom [Å].

Atomtype	X Coordinates	Y Coordinates	Z Coordinates
C	-3.395078	0.012005	3.317278
C	-2.168568	-0.651816	3.519286
C	-2.002112	-1.421918	4.691670
C	-3.035551	-1.517324	5.638285
C	-4.249443	-0.840185	5.431401

C	-4.425576	-0.075095	4.266611
P	-0.790090	-0.427445	2.282399
C	0.225047	-1.959036	2.459076
Si	0.996325	-2.746569	0.864383
C	1.956377	-4.238108	1.632301
C	2.873924	-5.035816	0.686148
C	0.191120	0.908390	3.125997
C	0.383620	2.132737	2.461290
C	1.071411	3.187211	3.082369
C	1.568203	3.028568	4.384958
C	1.378177	1.808280	5.059842
C	0.694666	0.753934	4.435550
Ni	-1.352502	0.136772	0.256780
Ge	1.268938	-0.234267	-1.251495
C	2.633504	1.273703	-0.872496
C	3.999206	1.165978	-1.239817
C	4.882959	2.247666	-1.131266
C	4.419208	3.498692	-0.656487
C	3.052990	3.637736	-0.311792
C	2.190396	2.542229	-0.426163
C	-2.253336	0.854479	-1.253749
N	-2.387851	2.183071	-1.594510
C	-3.350019	2.361793	-2.592440
C	-3.820750	1.122204	-2.907169
N	-3.138511	0.219240	-2.092580
C	-1.605820	3.285468	-1.087458
C	-0.653875	3.872024	-1.961365
C	0.057970	4.993132	-1.488680
C	-0.157734	5.492075	-0.200534
C	-1.101386	4.888956	0.645461
C	-1.859486	3.779853	0.222952
C	-3.378899	-1.200696	-2.163492
C	-4.453058	-1.738555	-1.415396
C	-4.764592	-3.098058	-1.613281
C	-4.024315	-3.883171	-2.506524
C	-2.947496	-3.328488	-3.213417
C	-2.597617	-1.972720	-3.061475
C	-0.415777	3.366288	-3.387415
C	1.072611	3.126150	-3.712674
C	-2.907213	3.126835	1.131498
C	-2.818666	3.552046	2.606396
C	-5.280411	-0.879369	-0.461243
C	-5.450335	-1.541704	0.919825
C	-1.471985	-1.343158	-3.882390
C	-2.011528	-0.808343	-5.228977
C	-1.032221	4.347294	-4.411669
C	-4.349346	3.346574	0.619568
C	-6.647907	-0.528936	-1.087143
C	-0.281110	-2.286903	-4.123284
C	-0.495923	-3.288178	-0.225545
C	-0.152395	-4.496727	-1.122737
N	2.031593	-1.661900	-0.040215
C	3.454714	-1.735341	-0.023129
C	4.213408	-1.174053	1.053968
C	5.618620	-1.267010	1.032729
C	6.298768	-1.859630	-0.037406
C	5.563067	-2.369159	-1.115290
C	4.155962	-2.323350	-1.133227
C	3.547107	-0.438498	2.216423
C	3.692240	-1.211879	3.544569

C	3.423835	-2.861409	-2.366876
C	3.581904	-1.903896	-3.570494
C	-1.798445	-3.555939	0.558103
C	4.086455	0.996549	2.396141
C	3.864148	-4.281843	-2.780941
C	1.025403	-5.202654	2.404011
H	-0.697755	-2.419089	-0.889320
H	-4.572506	0.793055	-3.622959
H	-3.600472	3.349731	-2.974011
H	-3.547906	0.583971	2.396137
H	-0.926183	2.392898	-3.496220
H	-0.454749	-2.734494	2.851518
H	1.010584	-1.797350	3.216594
H	4.755669	-1.280113	3.843185
H	3.306254	-2.243569	3.469094
H	3.146314	-0.693883	4.355239
H	-0.051000	-5.420874	-0.523322
H	0.791370	-4.361882	-1.680818
H	-0.956822	-4.673539	-1.861770
H	0.416032	6.358232	0.153445
H	6.095272	-2.814117	-1.965908
H	2.474653	-0.352151	1.974287
H	2.616683	-3.752299	2.379386
H	-1.080570	-0.486538	-3.302767
H	-0.024018	2.259351	1.454195
H	7.395249	-1.913645	-0.038042
H	0.549511	-0.187031	4.979092
H	6.193896	-0.845903	1.867318
H	4.402977	0.213696	-1.600556
H	-5.595711	-3.547036	-1.057015
H	-1.064051	-1.960206	4.873392
H	-1.247728	5.293791	1.651244
H	2.351250	-2.903344	-2.111735
H	5.939070	2.128615	-1.401726
H	-2.698734	2.038708	1.085434
H	-4.732480	0.065906	-0.300982
H	1.137543	2.693868	-0.172621
H	0.805631	5.466170	-2.134927
H	-4.280694	-4.941096	-2.646283
H	-5.373574	0.448751	4.087886
H	-1.198460	-0.322208	-5.800649
H	-2.816305	-0.066426	-5.086397
H	-2.414848	-1.637050	-5.841962
H	-2.594578	-3.901766	-0.126353
H	-2.177342	-2.640018	1.045789
H	-1.665791	-4.332367	1.335721
H	-2.373957	-3.958333	-3.901724
H	3.173975	-0.900485	-3.353501
H	3.047909	-2.299289	-4.455713
H	4.649501	-1.785325	-3.837933
H	-5.056444	-0.915456	6.171380
H	-2.890846	-2.125444	6.540592
H	3.511555	1.516489	3.182024
H	3.998940	1.577833	1.466129
H	5.149924	0.994525	2.701614
H	1.769658	1.675529	6.076461
H	1.660704	4.061146	-3.669288
H	1.166522	2.722256	-4.737950
H	1.530112	2.407781	-3.015068
H	2.111336	3.847625	4.873006

H	1.216431	4.128044	2.538085
H	-6.063726	-2.460141	0.862781
H	-5.956596	-0.848339	1.616212
H	-4.471196	-1.803038	1.355947
C	5.313827	4.614136	-0.527380
H	-0.543056	-3.122329	-4.799914
H	0.095312	-2.703479	-3.176447
H	0.547166	-1.725441	-4.589897
H	-1.808232	3.404112	3.021782
H	-3.518373	2.946655	3.209415
H	-3.102102	4.613648	2.739327
H	-2.106667	4.524938	-4.225205
H	-0.921244	3.953591	-5.439403
H	-0.520314	5.327005	-4.365539
H	3.403122	-5.833274	1.246850
H	3.638738	-4.393331	0.222615
H	2.303836	-5.530492	-0.120478
H	4.905158	-4.293744	-3.155713
H	3.218611	-4.653412	-3.599811
H	3.798253	-4.994579	-1.942752
H	2.676692	4.603565	0.044862
H	0.327237	-5.725207	1.723309
H	0.418673	-4.697462	3.177713
H	1.620327	-5.984992	2.917160
H	-6.525829	0.001745	-2.049212
H	-7.231674	0.120887	-0.407816
H	-7.239179	-1.444733	-1.277530
H	-4.605262	4.423257	0.632942
H	-5.065409	2.819403	1.278295
H	-4.499731	2.969157	-0.404718
H	0.237413	0.522450	-0.000388
H	-2.708712	-0.222658	0.651683
N	6.035531	5.532022	-0.421763

Table S54. Cartesian geometry of 3b-INT2 (20.3 kcal/mol) in Figure S145 in Angstrom [Å].

Atomtype	X Coordinates	Y Coordinates	Z Coordinates
C	4.120264	-0.556259	2.897216
C	3.017112	0.219751	3.302153
C	3.080561	0.884867	4.547316
C	4.213448	0.763140	5.367692
C	5.300948	-0.028643	4.959604
C	5.250889	-0.686191	3.720046
P	1.489866	0.297224	2.230934
C	0.807833	1.977349	2.570989
Si	0.058536	2.972221	1.089993
C	-0.532063	4.571377	2.005545
C	-1.353565	5.591318	1.194661
C	0.380563	-0.879975	3.147143
C	-0.021589	-2.073537	2.517053
C	-0.787662	-3.024137	3.211426
C	-1.173328	-2.783149	4.539133
C	-0.785392	-1.589381	5.173006
C	-0.007885	-0.646986	4.483729
Ni	1.724011	-0.250409	0.139866
Ge	-0.904158	0.602304	-1.026337
C	-2.586499	-0.552905	-0.592099
C	-3.858036	-0.013920	-0.902763
C	-5.035833	-0.768972	-0.787078
C	-4.980411	-2.110105	-0.385607
C	-3.728644	-2.678918	-0.102861

C	-2.547405	-1.910793	-0.195734
C	2.366875	-1.017273	-1.476290
N	2.274114	-2.337264	-1.855314
C	3.075554	-2.612236	-2.966136
C	3.679880	-1.440143	-3.309424
N	3.236387	-0.481951	-2.398215
C	1.428708	-3.348353	-1.268022
C	0.305771	-3.779035	-2.019090
C	-0.458804	-4.839014	-1.493132
C	-0.124239	-5.431812	-0.272390
C	0.983658	-4.976812	0.457154
C	1.794041	-3.929857	-0.021820
C	3.703706	0.880714	-2.454406
C	4.928752	1.194674	-1.818337
C	5.450380	2.487707	-2.018466
C	4.769429	3.424330	-2.806932
C	3.544195	3.092190	-3.402507
C	2.980130	1.811625	-3.241899
C	-0.058999	-3.186411	-3.383501
C	-1.525188	-2.715795	-3.465145
C	3.009039	-3.429196	0.766006
C	3.034815	-3.882834	2.234887
C	5.690312	0.164516	-0.986160
C	6.150651	0.732888	0.369866
C	1.682661	1.418683	-3.947381
C	1.976045	0.883100	-5.367676
C	0.242981	-4.205429	-4.506304
C	4.347961	-3.795695	0.086430
C	6.881878	-0.409784	-1.783320
C	0.647517	2.554858	-4.007997
C	1.514255	3.297409	-0.127059
C	1.317718	4.594489	-0.941555
N	-1.241825	2.117859	0.280298
C	-2.607301	2.499045	0.442604
C	-3.372241	2.044400	1.564578
C	-4.720463	2.432368	1.686564
C	-5.344595	3.227728	0.719648
C	-4.612809	3.642062	-0.399568
C	-3.257925	3.296230	-0.563030
C	-2.780039	1.116207	2.625064
C	-2.632813	1.826252	3.988033
C	-2.542109	3.739006	-1.843598
C	-2.985775	2.891165	-3.059142
C	2.908068	3.293005	0.536302
C	-3.604095	-0.176883	2.809902
C	-2.728362	5.235484	-2.174768
C	0.631108	5.293560	2.726669
H	1.494817	2.441363	-0.837004
H	4.381532	-1.192311	-4.104400
H	3.131398	-3.610510	-3.396365
H	4.098397	-1.044160	1.917735
H	0.571059	-2.295539	-3.554803
H	1.652113	2.585383	2.937354
H	0.066468	1.919611	3.385323
H	-3.623830	2.119814	4.383358
H	-2.024523	2.744802	3.915092
H	-2.159849	1.152802	4.726729
H	1.446222	5.487355	-0.301357
H	0.318085	4.665026	-1.405792
H	2.068533	4.661903	-1.751349

H	-0.745157	-6.242422	0.125827
H	-5.111052	4.237969	-1.174447
H	-1.778166	0.816975	2.273068
H	-1.207535	4.172822	2.789821
H	1.220408	0.603765	-3.359956
H	0.277673	-2.263443	1.479682
H	-6.402594	3.499088	0.822880
H	0.294658	0.271789	4.998594
H	-5.302238	2.080221	2.547749
H	-3.959119	1.021363	-1.235227
H	6.401131	2.764530	-1.548348
H	2.247040	1.512892	4.883769
H	1.215343	-5.446549	1.417787
H	-1.465592	3.557595	-1.687075
C	-6.384079	-0.123238	-1.030339
H	2.925556	-2.323787	0.762081
H	4.998600	-0.668841	-0.770311
H	-1.591242	-2.389790	0.042792
H	-1.338415	-5.193049	-2.038998
H	5.191292	4.427206	-2.950951
H	6.099144	-1.296166	3.383399
H	1.036610	0.567377	-5.859530
H	2.657796	0.014972	-5.348632
H	2.442442	1.670369	-5.990508
H	3.689988	3.526202	-0.209471
H	3.156660	2.303072	0.957604
H	2.985496	4.042346	1.347132
H	3.020956	3.838463	-4.009811
H	-2.689010	1.832756	-2.951476
H	-2.521955	3.272417	-3.988680
H	-4.084825	2.925318	-3.181597
H	6.185888	-0.124116	5.601676
H	4.246912	1.291425	6.329295
H	-3.058101	-0.879774	3.465197
H	-3.789667	-0.677399	1.848029
H	-4.584369	0.032052	3.278907
H	-1.089841	-1.389874	6.208388
H	-2.236676	-3.541236	-3.282017
H	-1.732370	-2.309487	-4.472719
H	-1.734036	-1.924391	-2.728732
H	-1.780867	-3.520661	5.078876
H	-1.085582	-3.946988	2.705925
H	6.917677	1.520020	0.247090
H	6.596304	-0.068338	0.987193
H	5.301047	1.158585	0.929962
H	-5.894965	-2.704361	-0.298912
H	0.972178	3.378910	-4.671121
H	0.448156	2.966032	-3.006412
H	-0.306880	2.165680	-4.402680
H	2.098760	-3.631091	2.761284
H	3.861998	-3.379838	2.765728
H	3.203756	-4.973264	2.319505
H	1.293793	-4.547346	-4.484006
H	0.046888	-3.757834	-5.498709
H	-0.400565	-5.099266	-4.402896
H	-1.656938	6.439466	1.841950
H	-2.271667	5.142408	0.785878
H	-0.772595	6.015257	0.356275
H	-3.768068	5.458724	-2.480277
H	-2.072981	5.518072	-3.020851

H	-2.480940	5.883552	-1.318561
C	-3.690593	-4.149641	0.240741
H	1.353239	5.720090	2.005221
H	1.196095	4.637517	3.413689
H	0.243361	6.138682	3.330561
H	6.547592	-0.873297	-2.729292
H	7.410124	-1.181026	-1.191124
H	7.607119	0.387802	-2.033205
H	4.479059	-4.894239	0.055845
H	5.191208	-3.368759	0.662229
H	4.420068	-3.411646	-0.944127
H	0.064382	-0.460325	0.057598
H	3.144553	-0.037845	0.372344
F	-7.278436	-1.005310	-1.551076
F	-6.928502	0.344003	0.124824
F	-6.306397	0.924397	-1.885950
F	-3.553224	-4.925595	-0.877941
F	-2.655487	-4.471855	1.059694
F	-4.824470	-4.567218	0.854091

Table S55. Cartesian geometry of 3c-INT2 (20.1 kcal/mol) in Figure S145 in Angstrom [Å].

Atomtype	X Coordinates	Y Coordinates	Z Coordinates
C	-0.029107	2.727850	1.955731
C	0.091435	1.649048	2.849674
C	0.630418	1.880758	4.133597
C	1.038781	3.169807	4.507665
C	0.915714	4.242401	3.604804
C	0.383806	4.017405	2.326190
P	-0.535400	-0.023294	2.329532
Ni	-1.255309	-0.025021	0.277478
C	-2.329340	0.140907	-1.279210
N	-2.801061	1.303505	-1.849819
C	-3.793490	1.043392	-2.799001
C	-3.939503	-0.310566	-2.850112
N	-3.037439	-0.842683	-1.929470
C	-2.313850	2.640386	-1.606746
C	-1.551034	3.257814	-2.631776
C	-1.138325	4.588923	-2.421967
C	-1.455160	5.262605	-1.238736
C	-2.203074	4.625068	-0.236913
C	-2.662985	3.303761	-0.397038
C	-2.912234	-2.263919	-1.722095
C	-3.805175	-2.892766	-0.822121
C	-3.765799	-4.298784	-0.745175
C	-2.863503	-5.036635	-1.522153
C	-1.971396	-4.383261	-2.384812
C	-1.973041	-2.980232	-2.508192
C	-1.206755	2.552273	-3.946969
C	-2.076395	3.109888	-5.097876
C	-3.494759	2.603431	0.683235
C	-4.955124	2.361203	0.237811
C	-4.809054	-2.093633	0.006613
C	-6.229882	-2.219431	-0.584936
C	-1.052380	-2.265042	-3.497093
C	0.331535	-2.921395	-3.639385
C	0.833519	-1.196598	2.722172
Si	1.755964	-2.061107	1.252145
C	0.434396	-3.156830	0.378201
C	-0.745868	-3.581739	1.277469
N	2.467433	-0.951323	0.102353

C	3.864089	-0.678741	0.044388
C	4.479080	0.241014	0.953217
C	5.865037	0.475905	0.868412
C	6.654535	-0.136139	-0.112065
C	6.048801	-0.998466	-1.035286
C	4.671798	-1.285843	-0.979967
C	3.668407	1.012151	1.994551
C	3.839357	2.540256	1.860957
C	4.072613	-2.201741	-2.052208
C	4.830608	-3.534955	-2.227515
Ge	1.353460	-0.006396	-1.306568
C	2.300827	1.827750	-1.300804
C	3.642460	1.977197	-1.732553
C	4.230450	3.245396	-1.885644
C	3.488212	4.409055	-1.621939
C	2.148636	4.288019	-1.215918
C	1.570687	3.016346	-1.062880
C	4.016560	0.577607	3.434299
C	3.982915	-1.479048	-3.416038
C	3.075110	-3.093651	2.220044
C	2.432462	-4.080658	3.222506
C	4.144875	-3.817666	1.380870
C	-1.793910	-0.332560	3.672879
C	-1.421122	-0.793907	4.954833
C	-2.382938	-0.954593	5.965975
C	-3.731929	-0.651994	5.714030
C	-4.113112	-0.197608	4.440675
C	-3.151917	-0.046169	3.428716
C	0.286586	2.646298	-4.321909
C	-3.487434	3.313943	2.046634
C	-4.785257	-2.493462	1.494683
C	-1.729206	-2.138847	-4.881420
C	1.062771	-4.393597	-0.299037
H	0.009752	-2.510105	-0.420734
H	-4.595008	-0.946654	-3.443042
H	-4.293878	1.846053	-3.337100
H	-3.457682	0.276361	2.427987
H	-1.439705	1.478891	-3.829934
H	0.375091	-2.024201	3.289808
H	1.562838	-0.705349	3.388354
H	5.067735	0.826696	3.675228
H	3.895986	-0.510157	3.581405
H	3.369840	1.101256	4.163245
H	1.401246	-5.130095	0.453893
H	1.935666	-4.140619	-0.926942
H	0.321501	-4.903312	-0.943670
H	-1.111500	6.294076	-1.087975
H	6.661175	-1.460435	-1.820555
H	2.604674	0.790250	1.806770
H	3.607197	-2.322724	2.814046
H	-0.875055	-1.246491	-3.104497
H	-0.460573	2.552008	0.966203
H	7.732163	0.067209	-0.165742
H	0.728305	1.058325	4.851857
H	6.332018	1.172420	1.576946
H	4.260850	1.096207	-1.937286
H	-4.448397	-4.820697	-0.064351
H	-0.374686	-1.038993	5.173320
H	-2.429886	5.169951	0.684316
H	3.043686	-2.439721	-1.732899

H	5.279149	3.320928	-2.204004
H	-3.019145	1.611271	0.822824
H	-4.515587	-1.030460	-0.046700
H	0.517970	2.960092	-0.770289
H	-0.539116	5.091699	-3.188853
H	-2.845787	-6.131461	-1.447319
H	-5.165733	0.029810	4.227518
H	-1.071292	-1.587004	-5.579212
H	-2.690605	-1.599539	-4.824130
H	-1.923529	-3.140225	-5.311432
H	-1.436363	-4.240771	0.719776
H	-1.339563	-2.711678	1.610149
H	-0.409054	-4.134605	2.175434
H	-1.266271	-4.974780	-2.978356
H	3.352596	-0.573846	-3.359986
H	3.545898	-2.147275	-4.182771
H	4.988380	-1.170559	-3.761626
H	-4.482888	-0.778079	6.504455
H	-2.075720	-1.320120	6.954428
H	3.163830	3.054782	2.566746
H	3.599724	2.882654	0.843150
H	4.875045	2.852487	2.095761
H	1.460804	3.337491	5.506837
H	0.595289	3.688784	-4.522511
H	0.472900	2.062482	-5.242668
H	0.934218	2.252886	-3.523344
H	1.245089	5.247373	3.897888
H	0.290812	4.837690	1.604551
H	-5.145518	-3.527716	1.648553
H	-5.441252	-1.823732	2.080597
H	-3.765661	-2.413841	1.908453
H	3.948826	5.399822	-1.733647
H	0.274897	-3.908311	-4.136799
H	0.815925	-3.045941	-2.658915
H	0.985539	-2.275075	-4.250551
H	-2.464069	3.499645	2.412299
H	-3.999492	2.684373	2.795755
H	-4.026852	4.279536	2.003186
H	-3.157097	3.034085	-4.881695
H	-1.873878	2.560474	-6.036443
H	-1.846159	4.178016	-5.272422
H	4.870832	-4.331146	2.044234
H	4.711340	-3.114961	0.750161
H	3.701961	-4.589399	0.726125
H	5.841527	-3.377681	-2.649261
H	4.282420	-4.191382	-2.930468
H	4.942638	-4.074112	-1.272715
H	1.546465	5.184583	-1.016008
H	1.871576	-4.880096	2.702581
H	1.736311	-3.592803	3.929290
H	3.215206	-4.579470	3.829295
H	-6.260440	-1.875072	-1.634979
H	-6.948928	-1.612043	-0.002940
H	-6.573123	-3.271267	-0.562882
H	-5.474717	3.324500	0.072784
H	-5.501938	1.811578	1.027514
H	-5.024824	1.768835	-0.688748
H	0.167170	0.697523	-0.158111
H	-2.464723	-0.639701	0.817047

Table S56. Cartesian geometry of 3d-INT2 (20.9 kcal/mol) in Figure S145 in Angstrom [Å].

Atomtype	X Coordinates	Y Coordinates	Z Coordinates
C	-3.324918	-0.152341	3.344803
C	-2.035763	-0.678467	3.561420
C	-1.785129	-1.378379	4.762464
C	-2.797898	-1.538832	5.722605
C	-4.075722	-0.997739	5.500623
C	-4.335685	-0.303910	4.307218
P	-0.695249	-0.367606	2.300163
C	0.467110	-1.782835	2.525705
Si	1.304935	-2.547104	0.952561
C	2.415186	-3.905293	1.769058
C	3.398589	-4.647652	0.844677
C	0.152588	1.090786	3.083317
C	0.217324	2.300944	2.370207
C	0.802329	3.441583	2.942341
C	1.322457	3.383936	4.244115
C	1.260212	2.178130	4.967338
C	0.679928	1.037897	4.391552
Ni	-1.327126	0.051140	0.262953
Ge	1.311311	-0.093690	-1.262823
C	2.519962	1.546639	-0.952372
C	3.882302	1.560193	-1.341427
C	4.657919	2.728993	-1.272552
C	4.108960	3.946929	-0.820861
C	2.746766	3.952267	-0.461219
C	1.975747	2.780484	-0.528256
C	-2.293670	0.615716	-1.271249
N	-2.552175	1.909494	-1.670591
C	-3.524952	1.952228	-2.673328
C	-3.875024	0.661115	-2.933137
N	-3.112571	-0.136635	-2.080783
C	-1.884694	3.103116	-1.208120
C	-0.990361	3.746033	-2.103115
C	-0.395538	4.949672	-1.674336
C	-0.666636	5.475437	-0.407834
C	-1.549398	4.815354	0.460697
C	-2.190837	3.620307	0.082118
C	-3.218653	-1.574549	-2.087985
C	-4.232586	-2.177563	-1.305986
C	-4.414068	-3.568053	-1.439184
C	-3.608014	-4.319254	-2.304333
C	-2.594372	-3.697339	-3.047516
C	-2.374084	-2.308939	-2.959757
C	-0.692275	3.208015	-3.505903
C	0.814116	3.135130	-3.830192
C	-3.168518	2.900260	1.017777
C	-3.123289	3.387274	2.475414
C	-5.133871	-1.357573	-0.384982
C	-5.241214	-1.972253	1.023929
C	-1.321723	-1.613361	-3.823367
C	-1.920053	-1.210852	-5.190987
C	-1.419638	4.060604	-4.571831
C	-4.624941	2.953116	0.502364
C	-6.528363	-1.161056	-1.018619
C	-0.041349	-2.441914	-4.025056
C	-0.132152	-3.282477	-0.099245
C	0.329360	-4.485190	-0.950054
N	2.219065	-1.400878	-0.000653

C	3.641610	-1.337387	0.003860
C	4.352103	-0.666441	1.050526
C	5.759682	-0.633517	1.021495
C	6.485824	-1.204752	-0.029987
C	5.793715	-1.818391	-1.082212
C	4.388353	-1.899616	-1.090086
C	3.627143	0.053814	2.187113
C	3.849779	-0.644236	3.545820
C	3.700408	-2.544665	-2.297510
C	3.767177	-1.621714	-3.535994
C	-1.395891	-3.651505	0.706495
C	4.032275	1.539155	2.297975
C	4.260021	-3.935769	-2.664231
C	1.593152	-4.923785	2.593202
H	-0.424930	-2.466387	-0.795824
H	-4.591195	0.231345	-3.631846
H	-3.870240	2.894205	-3.094659
H	-3.540158	0.360555	2.401637
H	-1.082471	2.176351	-3.565577
H	-0.130675	-2.606532	2.951878
H	1.236111	-1.515634	3.270229
H	4.916821	-0.602541	3.837012
H	3.559518	-1.709284	3.518980
H	3.263370	-0.143223	4.338810
H	0.526673	-5.369277	-0.315013
H	1.252371	-4.277099	-1.520060
H	-0.455385	-4.772816	-1.675542
H	-0.181506	6.406201	-0.086745
H	6.359595	-2.245177	-1.920504
H	2.550185	0.031374	1.950776
H	3.031369	-3.324749	2.485727
H	-1.016626	-0.691639	-3.294140
H	-0.204271	2.346084	1.361929
H	7.582895	-1.160779	-0.037212
H	0.634945	0.108538	4.971340
H	6.297983	-0.128907	1.834418
H	4.369771	0.642873	-1.690103
H	-5.194848	-4.068204	-0.854359
H	-0.796055	-1.810840	4.956401
H	-1.738560	5.241672	1.450338
H	2.636946	-2.669735	-2.031792
H	5.716389	2.690657	-1.567633
H	-2.851359	1.837586	1.012960
H	-4.674216	-0.360419	-0.268453
H	0.917048	2.847213	-0.261331
H	0.308479	5.465272	-2.336432
H	-3.762815	-5.402149	-2.393372
H	-5.333308	0.112695	4.116529
H	-1.164814	-0.673920	-5.795633
H	-2.797606	-0.550417	-5.079853
H	-2.237255	-2.108771	-5.755369
H	-2.155151	-4.105889	0.043744
H	-1.865878	-2.760842	1.160617
H	-1.179277	-4.377935	1.513233
H	-1.968451	-4.300189	-3.714090
H	3.275260	-0.650363	-3.350610
H	3.264565	-2.094517	-4.401583
H	4.818935	-1.420476	-3.816889
H	-4.866578	-1.123789	6.251140
H	-2.586712	-2.091227	6.647434

H	3.411835	2.039343	3.062273
H	3.891181	2.064771	1.341793
H	5.091964	1.646847	2.599508
H	1.671613	2.124204	5.983415
H	1.277402	4.138610	-3.858177
H	0.954429	2.677828	-4.827454
H	1.363290	2.532807	-3.090355
H	1.785786	4.271004	4.694485
H	0.851766	4.368306	2.358375
H	-5.770894	-2.943036	1.009735
H	-5.805456	-1.297146	1.693262
H	-4.241525	-2.126484	1.464403
C	4.963665	5.189986	-0.695888
H	-0.220454	-3.340963	-4.644860
H	0.385677	-2.755414	-3.060349
H	0.719288	-1.827595	-4.537995
H	-2.102889	3.355470	2.891938
H	-3.759403	2.738428	3.103224
H	-3.510675	4.419850	2.570135
H	-2.508389	4.116992	-4.393777
H	-1.257408	3.638143	-5.581279
H	-1.028513	5.095696	-4.569297
H	4.013661	-5.360703	1.431205
H	4.087204	-3.951649	0.340869
H	2.872774	-5.234105	0.070089
H	5.296955	-3.871573	-3.045317
H	3.645858	-4.390667	-3.465140
H	4.258900	-4.621290	-1.801123
H	2.277661	4.886913	-0.122470
H	0.942552	-5.540330	1.944673
H	0.947785	-4.448872	3.354863
H	2.267975	-5.622158	3.128601
H	-6.456147	-0.664438	-2.003641
H	-7.168198	-0.537604	-0.365457
H	-7.033927	-2.134431	-1.165739
H	-4.989084	3.998031	0.475277
H	-5.284570	2.381208	1.182438
H	-4.734853	2.523503	-0.506416
H	0.204570	0.600686	-0.030956
H	-2.636042	-0.442913	0.681850
H	4.351631	6.111044	-0.711918
H	5.529215	5.187895	0.258386
H	5.708664	5.255670	-1.511136

Table S57. Cartesian geometry of 3e-INT2 (21.4 kcal/mol) in Figure S145 in Angstrom [Å].

Atomtype	X Coordinates	Y Coordinates	Z Coordinates
C	-3.407070	0.200690	3.322195
C	-2.217163	-0.524501	3.531618
C	-2.089625	-1.285888	4.714438
C	-3.124236	-1.312411	5.664240
C	-4.300655	-0.574144	5.449925
C	-4.438468	0.182518	4.274480
P	-0.832342	-0.393983	2.286510
C	0.092242	-1.979414	2.479905
Si	0.811206	-2.828178	0.890577
C	1.681172	-4.366236	1.677059
C	2.549079	-5.227525	0.739948
C	0.225425	0.892725	3.115865
C	0.484461	2.097011	2.437729
C	1.232767	3.117344	3.045612

C	1.724113	2.944834	4.348378
C	1.468551	1.744101	5.036619
C	0.724837	0.723574	4.425197
Ni	-1.371618	0.178771	0.259714
Ge	1.224869	-0.352504	-1.257731
C	2.669430	1.068917	-0.900473
C	4.025276	0.888425	-1.282610
C	4.967664	1.919513	-1.188536
C	4.587238	3.192610	-0.711453
C	3.243957	3.412027	-0.346474
C	2.317303	2.357770	-0.448166
C	-2.221800	0.936692	-1.259449
N	-2.272394	2.267106	-1.617615
C	-3.218126	2.492509	-2.622054
C	-3.762261	1.280508	-2.925031
N	-3.140186	0.347132	-2.096732
C	-1.430392	3.327064	-1.116459
C	-0.439704	3.848716	-1.988679
C	0.331646	4.931914	-1.521513
C	0.135703	5.455759	-0.240355
C	-0.845791	4.915963	0.605099
C	-1.662558	3.847339	0.187890
C	-3.468698	-1.055763	-2.150521
C	-4.572624	-1.516952	-1.394306
C	-4.966650	-2.857534	-1.572406
C	-4.277387	-3.698701	-2.455434
C	-3.171246	-3.220068	-3.172250
C	-2.738890	-1.886117	-3.039791
C	-0.218513	3.313356	-3.406680
C	1.260148	3.013200	-3.727752
C	-2.748352	3.261005	1.097018
C	-2.639488	3.689408	2.569555
C	-5.345421	-0.595056	-0.452967
C	-5.562320	-1.228039	0.935170
C	-1.579641	-1.338560	-3.872416
C	-2.090189	-0.793200	-5.226093
C	-0.793929	4.302117	-4.447508
C	-4.174228	3.557818	0.578841
C	-6.685498	-0.164439	-1.088083
C	-0.448960	-2.356396	-4.101450
C	-0.715016	-3.294574	-0.187000
C	-0.442387	-4.528383	-1.073962
N	1.904032	-1.818793	-0.027161
C	3.319040	-1.975376	-0.012120
C	4.113522	-1.449739	1.056946
C	5.509821	-1.631130	1.036207
C	6.150227	-2.278054	-0.027062
C	5.382607	-2.750751	-1.099475
C	3.981393	-2.615921	-1.117237
C	3.495233	-0.658762	2.209368
C	3.598618	-1.417453	3.549661
C	3.214493	-3.116428	-2.345520
C	3.435787	-2.184352	-3.558925
C	-2.027007	-3.482838	0.603669
C	4.117557	0.745343	2.360962
C	3.556371	-4.568000	-2.743995
C	0.698393	-5.266198	2.462266
H	-0.869244	-2.420878	-0.857432
H	-4.530316	0.989886	-3.640184
H	-3.408964	3.489509	-3.014051

H	-3.531063	0.764556	2.391761
H	-0.766259	2.358676	-3.500550
H	-0.630364	-2.708496	2.884874
H	0.889174	-1.853867	3.232421
H	4.657132	-1.543621	3.847451
H	3.152372	-2.425907	3.492204
H	3.086591	-0.855216	4.353158
H	-0.391181	-5.451339	-0.466176
H	0.506334	-4.451253	-1.634592
H	-1.257157	-4.666822	-1.810097
H	0.755902	6.291082	0.110023
H	5.884692	-3.236448	-1.946307
H	2.429253	-0.513146	1.967034
H	2.370312	-3.910605	2.417228
H	-1.134273	-0.499032	-3.306753
H	0.083643	2.234729	1.429394
H	7.241232	-2.401237	-0.027600
H	0.530337	-0.202611	4.978546
H	6.111331	-1.236506	1.865429
H	4.375087	-0.084349	-1.646298
H	-5.822246	-3.247408	-1.008725
H	-1.181553	-1.871864	4.901096
H	-0.974433	5.337243	1.606570
H	2.141704	-3.081902	-2.090127
H	6.015501	1.758990	-1.470158
H	-2.601403	2.162443	1.058673
H	-4.737494	0.314211	-0.301710
H	1.279992	2.577172	-0.179079
H	1.111433	5.351726	-2.166250
H	-4.597438	-4.741184	-2.579364
H	-5.357801	0.753336	4.089581
H	-1.250885	-0.365865	-5.806847
H	-2.848537	-0.002003	-5.093954
H	-2.544388	-1.605550	-5.825414
H	-2.844020	-3.791251	-0.074340
H	-2.352894	-2.542671	1.083506
H	-1.933738	-4.257658	1.388714
H	-2.638884	-3.893088	-3.852731
H	3.097120	-1.153736	-3.351189
H	2.875611	-2.552153	-4.440040
H	4.508849	-2.140350	-3.827487
H	-5.108840	-0.595773	6.192217
H	-3.010257	-1.915004	6.574701
H	3.574607	1.313274	3.136823
H	4.062102	1.309795	1.418249
H	5.179753	0.685749	2.666425
H	1.856038	1.599604	6.053242
H	1.876148	3.931378	-3.724336
H	1.334735	2.568871	-4.737842
H	1.701113	2.310318	-3.004186
H	2.314202	3.736878	4.826827
H	1.429852	4.041113	2.488634
H	-6.237385	-2.102804	0.887664
H	-6.020875	-0.493397	1.622332
H	-4.603735	-1.550433	1.376008
O	5.578399	4.135759	-0.644103
H	-0.763122	-3.185008	-4.764307
H	-0.094497	-2.779156	-3.148994
H	0.410149	-1.853138	-4.578559
H	-1.639997	3.485724	2.988156

H	-3.374022	3.128454	3.174129
H	-2.862175	4.766301	2.696034
H	-1.861864	4.523131	-4.271496
H	-0.691495	3.890534	-5.469181
H	-0.245288	5.262381	-4.411105
H	3.032704	-6.047473	1.309657
H	3.348396	-4.635789	0.266979
H	1.949356	-5.698418	-0.059559
H	4.595078	-4.656038	-3.115355
H	2.888471	-4.902819	-3.560963
H	3.439285	-5.265711	-1.898721
H	2.900499	4.386535	0.014640
H	-0.027997	-5.759371	1.789033
H	0.120473	-4.716949	3.228099
H	1.248821	-6.073123	2.987304
H	-6.526450	0.344633	-2.056389
H	-7.227607	0.530802	-0.419150
H	-7.334604	-1.042284	-1.268967
H	-4.370437	4.647156	0.585606
H	-4.920689	3.074305	1.237366
H	-4.341438	3.183735	-0.444099
H	0.228645	0.483789	-0.015354
H	-2.747102	-0.114960	0.656330
C	5.253868	5.421995	-0.155116
H	6.185960	6.013242	-0.172185
H	4.494449	5.930114	-0.788998
H	4.868422	5.385707	0.886933

Table S58. Cartesian geometry of 3f-INT2 (22 kcal/mol) in Figure S145 in Angstrom [Å].

Atomtype	X Coordinates	Y Coordinates	Z Coordinates
C	-3.481299	0.629099	3.288494
C	-2.418555	-0.271834	3.499753
C	-2.426812	-1.062631	4.670094
C	-3.467959	-0.946197	5.606083
C	-4.514746	-0.033775	5.390498
C	-4.517727	0.753550	4.227119
P	-1.012705	-0.332194	2.272645
C	-0.333708	-2.038075	2.457230
Si	0.283067	-2.964033	0.868422
C	0.916178	-4.616625	1.650194
C	1.662457	-5.585711	0.713856
C	0.210766	0.776159	3.131552
C	0.668437	1.926704	2.465373
C	1.550735	2.818552	3.095323
C	1.977744	2.570901	4.408598
C	1.524413	1.422738	5.084801
C	0.647595	0.529519	4.450969
Ni	-1.439258	0.333143	0.247676
Ge	1.077653	-0.548911	-1.247127
C	2.700311	0.642865	-0.857946
C	4.023254	0.276502	-1.206346
C	5.110452	1.151309	-1.073113
C	4.934228	2.477813	-0.587312
C	3.605423	2.865929	-0.258270
C	2.538272	1.969056	-0.398643
C	-2.153201	1.221601	-1.270181
N	-2.007330	2.549109	-1.612349
C	-2.900961	2.920241	-2.621035
C	-3.611797	1.803143	-2.943382
N	-3.138829	0.780101	-2.122336

C	-1.021861	3.467712	-1.093945
C	0.043112	3.843723	-1.953254
C	0.963154	4.795483	-1.469536
C	0.837155	5.329582	-0.183877
C	-0.219965	4.932782	0.649446
C	-1.183694	4.002528	0.214909
C	-3.667046	-0.559667	-2.194067
C	-4.837406	-0.860327	-1.457309
C	-5.420069	-2.127803	-1.652842
C	-4.847212	-3.054625	-2.533184
C	-3.671374	-2.738162	-3.228904
C	-3.051417	-1.482356	-3.078906
C	0.195912	3.290412	-3.373228
C	1.617290	2.778024	-3.684263
C	-2.351788	3.574849	1.110358
C	-2.190322	3.964156	2.588936
C	-5.480084	0.156406	-0.515873
C	-5.796924	-0.448898	0.865423
C	-1.809542	-1.103984	-3.886057
C	-2.208352	-0.458464	-5.233139
C	-0.218862	4.358918	-4.411629
C	-3.712782	4.089295	0.588168
C	-6.739503	0.777320	-1.158334
C	-0.845067	-2.278219	-4.125234
C	-1.277971	-3.202217	-0.235859
C	-1.172707	-4.460365	-1.124243
N	1.522180	-2.113318	-0.022701
C	2.899098	-2.470754	0.008496
C	3.746564	-2.075439	1.093143
C	5.102491	-2.455419	1.087137
C	5.658369	-3.176668	0.024048
C	4.845693	-3.522621	-1.063518
C	3.478789	-3.187696	-1.096426
C	3.233206	-1.215095	2.247305
C	3.207961	-1.993206	3.580204
C	2.665500	-3.557560	-2.340991
C	3.038338	-2.653827	-3.538637
C	-2.616779	-3.198939	0.531833
C	4.048455	0.083525	2.421379
C	2.797293	-5.038985	-2.753988
C	-0.193276	-5.372011	2.418544
H	-1.292351	-2.314377	-0.905049
H	-4.407002	1.634823	-3.668066
H	-2.941322	3.938614	-3.002339
H	-3.504153	1.219434	2.366601
H	-0.485163	2.426949	-3.477501
H	-1.163252	-2.658597	2.837175
H	0.457575	-2.041285	3.226126
H	4.233227	-2.272197	3.890532
H	2.623120	-2.926930	3.505767
H	2.769553	-1.370894	4.382992
H	-1.265330	-5.383031	-0.520835
H	-0.214237	-4.519548	-1.670425
H	-1.987552	-4.477508	-1.873204
H	1.573413	6.057838	0.180330
H	5.284511	-4.066270	-1.910349
H	2.202819	-0.915018	1.994152
H	1.655780	-4.268538	2.399988
H	-1.248414	-0.356093	-3.295204
H	0.318359	2.125183	1.448535

H	6.720152	-3.456334	0.035764
H	0.299582	-0.356832	4.994280
H	5.742484	-2.159837	1.928821
H	4.240350	-0.733046	-1.573672
H	-6.331636	-2.392387	-1.104289
H	-1.621691	-1.783317	4.858118
H	-0.291597	5.356854	1.655418
H	1.605588	-3.369281	-2.099431
H	6.105331	0.781447	-1.339741
H	-2.371564	2.467068	1.058738
H	-4.750332	0.968813	-0.352669
H	1.539835	2.341088	-0.150049
H	1.804049	5.097920	-2.102965
H	-5.313679	-4.038535	-2.670772
H	-5.336490	1.460739	4.040741
H	-1.303912	-0.152783	-5.792496
H	-2.838528	0.436798	-5.092884
H	-2.771399	-1.178948	-5.857156
H	-3.457816	-3.385929	-0.161045
H	-2.811541	-2.221202	1.008016
H	-2.650646	-3.979357	1.316280
H	-3.231425	-3.477931	-3.906062
H	2.855712	-1.586879	-3.319065
H	2.439882	-2.922383	-4.430429
H	4.109081	-2.767783	-3.796053
H	-5.328053	0.056272	6.121998
H	-3.461019	-1.573739	6.506777
H	3.587180	0.712454	3.202863
H	4.079920	0.662693	1.486389
H	5.089138	-0.131666	2.731738
H	1.860598	1.219080	6.109576
H	2.361306	3.595704	-3.667631
H	1.635035	2.334576	-4.697436
H	1.943254	2.012961	-2.962612
H	2.671215	3.262039	4.904573
H	1.903500	3.699788	2.546413
H	-6.587429	-1.220033	0.805030
H	-6.154077	0.338217	1.554711
H	-4.897062	-0.905540	1.311440
N	6.008053	3.353638	-0.440001
H	-1.273453	-3.035979	-4.808641
H	-0.571531	-2.768373	-3.178466
H	0.086719	-1.902652	-4.583230
H	-1.236569	3.605561	3.010117
H	-3.006153	3.514604	3.182265
H	-2.247222	5.060862	2.728173
H	-1.244183	4.733674	-4.242355
H	-0.169226	3.942688	-5.435405
H	0.464798	5.227765	-4.364106
H	2.024470	-6.467261	1.281951
H	2.538726	-5.107776	0.248677
H	1.008545	-5.965491	-0.091516
H	3.818251	-5.275027	-3.109818
H	2.102038	-5.262604	-3.586036
H	2.563643	-5.721230	-1.920293
H	3.388291	3.871733	0.112820
H	-0.980949	-5.742040	1.735369
H	-0.687574	-4.753318	3.189964
H	0.228079	-6.258924	2.934073
H	-6.500786	1.265575	-2.121020

H	-7.183756	1.537713	-0.488117
H	-7.505029	0.001948	-1.351978
H	-3.743058	5.195590	0.609235
H	-4.528827	3.714909	1.235214
H	-3.925960	3.758222	-0.441075
H	0.189174	0.411343	-0.010613
H	-2.848706	0.233680	0.624924
C	5.794898	4.646655	0.180349
C	7.361559	2.869936	-0.644640
H	8.079403	3.690478	-0.478866
H	7.625470	2.036001	0.043748
H	7.510743	2.501013	-1.680340
H	6.745829	5.203561	0.219760
H	5.072611	5.262804	-0.395668
H	5.400783	4.564409	1.219040

Table S59. Cartesian geometry of 3g-INT2 (23.4 kcal/mol) in Figure S145 in Angstrom [Å].

Atomtype	X Coordinates	Y Coordinates	Z Coordinates
C	2.821737	2.845562	-2.159132
C	1.423702	2.939726	-2.311153
C	0.909377	3.796722	-3.309131
C	1.773397	4.546153	-4.124941
C	3.165136	4.454509	-3.953644
C	3.686419	3.600283	-2.967648
P	0.308090	1.982335	-1.159325
C	-1.225905	1.718412	-2.150838
Si	-2.181489	0.041768	-1.952118
C	-3.674182	0.354798	-3.143908
C	-4.798545	-0.698070	-3.148505
C	-0.126201	3.316633	0.062755
C	0.188922	3.119493	1.418747
C	-0.057967	4.124943	2.367046
C	-0.619477	5.346121	1.964116
C	-0.942124	5.551792	0.609681
C	-0.698714	4.543973	-0.335613
Ni	1.167090	0.226804	-0.212767
Ge	-1.374277	-1.005439	1.117495
N	-2.003616	0.012002	2.659334
C	2.363118	-1.026950	0.552166
N	3.058629	-0.891305	1.733584
C	4.085600	-1.833488	1.839325
C	4.026675	-2.600064	0.713789
N	2.969473	-2.105031	-0.049537
C	2.728876	0.000410	2.817761
C	2.130640	-0.570184	3.969715
C	1.881734	0.284632	5.062297
C	2.191903	1.646699	4.994288
C	2.768714	2.187680	3.833865
C	3.064357	1.379005	2.720068
C	2.592021	-2.702371	-1.304948
C	3.311955	-2.337469	-2.466416
C	3.038340	-3.050917	-3.650261
C	2.074206	-4.066608	-3.670288
C	1.348385	-4.379510	-2.510949
C	1.587583	-3.705474	-1.297985
C	1.789709	-2.060468	4.066829
C	0.329008	-2.328789	4.483636
C	3.703685	1.947433	1.448877
C	3.669121	3.481417	1.354241
C	4.370169	-1.236036	-2.445059

C	4.137959	-0.189328	-3.552880
C	0.838835	-4.069375	-0.014847
C	1.620221	-5.119336	0.808333
C	2.765380	-2.773728	5.030370
C	5.154658	1.453448	1.247976
C	5.790419	-1.835879	-2.527301
C	-0.602779	-4.550634	-0.251931
C	-0.982883	-1.323240	-2.591391
C	-1.744694	-2.533602	-3.173199
N	-2.674229	-0.321670	-0.314680
C	-4.016576	-0.192505	0.135179
C	-4.559365	1.084693	0.488545
C	-5.893224	1.172205	0.931051
C	-6.696363	0.033852	1.071239
C	-6.157387	-1.223028	0.765979
C	-4.837205	-1.364055	0.298878
C	-3.717031	2.358977	0.440919
C	-4.234243	3.368342	-0.605060
C	-4.306556	-2.773561	0.016731
C	-4.059224	-3.556686	1.326388
C	0.078429	-0.827626	-3.595928
C	-3.620236	3.036086	1.822199
C	-5.218426	-3.603226	-0.912414
C	-3.223798	0.650748	-4.593678
H	-0.432220	-1.666981	-1.687922
H	4.627454	-3.444777	0.379572
H	4.745881	-1.864293	2.704293
H	3.231680	2.153626	-1.415807
H	1.917128	-2.507576	3.065411
H	-0.910438	1.736549	-3.208099
H	-1.917772	2.565191	-2.000500
H	-5.244018	3.734419	-0.337549
H	-4.303116	2.921818	-1.612951
H	-3.561764	4.245449	-0.659513
H	-2.221378	-2.275288	-4.137433
H	-2.540951	-2.902651	-2.502235
H	-1.051478	-3.373914	-3.368238
H	1.977295	2.299784	5.849914
H	-6.778821	-2.118976	0.894206
H	-2.692575	2.060254	0.167113
H	-4.116462	1.288708	-2.740810
H	0.763688	-3.147183	0.591732
H	0.643575	2.173240	1.725960
H	-7.731652	0.123307	1.425731
H	-0.950183	4.724261	-1.387263
H	-6.306547	2.156395	1.189170
H	3.583776	-2.801270	-4.568092
H	-0.173170	3.879554	-3.464624
H	2.991011	3.258627	3.800011
H	-3.332548	-2.653584	-0.487588
H	3.094887	1.547036	0.611822
H	4.282569	-0.706945	-1.479654
H	1.420413	-0.123516	5.968947
H	1.872858	-4.610927	-4.601804
H	4.772232	3.509865	-2.832982
H	1.084113	-5.338834	1.751100
H	2.634134	-4.771107	1.069660
H	1.717011	-6.064257	0.239930
H	0.698054	-1.671457	-3.950575
H	0.768524	-0.101634	-3.130831

H	-0.378912	-0.352586	-4.484983
H	0.586715	-5.165283	-2.549814
H	-3.316185	-3.053017	1.969801
H	-3.680557	-4.573431	1.106051
H	-4.996741	-3.657643	1.906325
H	3.839551	5.039539	-4.592064
H	1.355804	5.202041	-4.899870
H	-2.954214	3.915190	1.769471
H	-3.205069	2.332736	2.558606
H	-4.610597	3.378365	2.178821
H	-1.390107	6.501040	0.288931
H	0.113226	-1.945247	5.497809
H	0.142284	-3.419025	4.494563
H	-0.384858	-1.868280	3.780479
H	-0.816380	6.134893	2.701371
H	0.192539	3.942444	3.419195
H	4.268768	-0.623423	-4.561708
H	4.860900	0.641104	-3.451255
H	3.122693	0.236850	-3.484833
H	-0.633829	-5.543630	-0.739527
H	-1.167728	-3.836823	-0.870628
H	-1.127015	-4.635720	0.715908
H	2.651771	3.883096	1.490133
H	4.022280	3.798909	0.357004
H	4.334961	3.948068	2.105413
H	3.819581	-2.628931	4.732043
H	2.560428	-3.860576	5.054716
H	2.652515	-2.382927	6.059374
H	-5.618575	-0.381135	-3.825230
H	-5.231409	-0.837509	-2.145569
H	-4.441548	-1.679027	-3.510261
H	-6.181370	-3.849274	-0.425761
H	-4.726918	-4.561853	-1.167716
H	-5.440781	-3.071824	-1.852007
H	-2.762562	-0.237697	-5.064776
H	-2.494990	1.478902	-4.663858
H	-4.095285	0.930523	-5.219866
H	5.977175	-2.542604	-1.697772
H	6.554175	-1.036696	-2.476887
H	5.932365	-2.384561	-3.477783
H	5.803750	1.814541	2.068626
H	5.557880	1.850301	0.296856
H	5.227835	0.354604	1.208204
H	-0.066240	0.222465	0.858419
H	2.221770	0.319866	-1.229468
C	-3.227958	-0.359692	3.348176
C	-1.102958	0.736833	3.528982
H	-1.472919	1.766455	3.755439
H	-0.957630	0.237378	4.516608
H	-0.101321	0.838397	3.083508
H	-3.762292	0.535828	3.739786
H	-3.937044	-0.873310	2.679116
H	-3.039251	-1.026671	4.228651

Table S60. Cartesian geometry of 3a-TS2 (20.8 kcal/mol) in Figure S145 in Angstrom [Å].

Atomtype	X Coordinates	Y Coordinates	Z Coordinates
C	-2.119581	-1.509711	4.517523
C	-2.147297	-0.739165	3.334871
C	-3.303876	0.008561	3.040007
C	-4.393260	0.025018	3.924842

C	-4.353236	-0.733535	5.106264
C	-3.217811	-1.511299	5.393188
P	-0.669549	-0.588189	2.208299
C	0.368466	0.575424	3.240528
C	0.620370	1.875977	2.769419
C	1.326802	2.802970	3.552714
C	1.787212	2.439611	4.827210
C	1.543998	1.140015	5.307951
C	0.840530	0.214895	4.521734
Ni	-1.174600	0.141049	0.231403
C	-2.239697	0.999098	-1.098897
N	-2.337966	2.344321	-1.384134
C	-3.346557	2.601463	-2.315855
C	-3.884518	1.394509	-2.651020
N	-3.199121	0.433428	-1.909839
C	-1.488812	3.400551	-0.888790
C	-0.583072	3.994327	-1.804507
C	0.175026	5.093176	-1.351386
C	0.054221	5.558259	-0.038772
C	-0.829377	4.933652	0.855952
C	-1.632360	3.848863	0.454051
C	-3.443087	-0.976964	-2.092265
C	-4.468387	-1.594799	-1.337149
C	-4.781094	-2.933484	-1.641704
C	-4.085053	-3.627841	-2.640049
C	-3.049876	-3.000932	-3.346362
C	-2.704295	-1.659409	-3.091076
C	-0.420273	3.502961	-3.246105
C	1.048947	3.216262	-3.621119
C	-2.592221	3.153594	1.422978
C	-2.422495	3.559928	2.895525
C	-5.230194	-0.833035	-0.255198
C	-5.472426	-1.683475	1.005370
C	-1.617027	-0.956405	-3.902424
C	-2.200108	-0.358415	-5.202858
Ge	1.030358	-0.107315	-1.052973
C	2.520026	1.317681	-0.799474
C	2.196413	2.595616	-0.276952
C	3.138098	3.625199	-0.175942
C	4.467710	3.412595	-0.611890
C	4.811527	2.157017	-1.166659
C	3.850068	1.142449	-1.261060
C	5.444428	4.458647	-0.496318
N	6.234743	5.319359	-0.402213
N	1.898825	-1.650412	-0.103840
C	3.320174	-1.762545	-0.177947
C	4.155490	-1.335870	0.901648
C	5.552708	-1.475166	0.791557
C	6.149687	-1.990497	-0.364682
C	5.337376	-2.367838	-1.441924
C	3.934658	-2.265933	-1.376749
C	3.581005	-0.687000	2.159002
C	3.807978	-1.551752	3.416818
C	3.118109	-2.643537	-2.616329
C	3.244409	-1.566961	-3.718844
C	0.182486	-2.222678	2.341608
Si	0.920013	-2.893456	0.686152
C	-0.571894	-3.406167	-0.416970
C	-0.210536	-4.497935	-1.447173
C	1.971852	-4.390518	1.302250

C	2.849557	-5.127639	0.273193
C	-1.038500	4.512545	-4.239682
C	-4.078633	3.310351	1.026938
C	-6.560819	-0.276571	-0.808541
C	-0.416097	-1.864151	-4.219487
C	4.146664	0.730588	2.389759
C	3.477735	-4.025550	-3.201568
C	1.103363	-5.409639	2.077161
C	-1.824261	-3.824204	0.382907
H	-0.008768	1.131297	0.156948
H	-0.843691	-2.484678	-0.972903
H	-4.682205	1.125751	-3.341813
H	-3.582904	3.613768	-2.638413
H	-3.346516	0.557520	2.094375
H	-0.966879	2.548545	-3.346819
H	-0.560425	-2.956600	2.698026
H	0.983591	-2.165703	3.098758
H	4.888114	-1.663866	3.629833
H	3.387161	-2.566582	3.302771
H	3.336767	-1.078723	4.298078
H	-0.050168	-5.475087	-0.953250
H	0.703639	-4.265480	-2.021254
H	-1.035688	-4.629483	-2.173787
H	0.660518	6.408652	0.298628
H	5.804816	-2.747895	-2.359274
H	2.495231	-0.581232	2.003127
H	2.662745	-3.920351	2.031270
H	-1.227827	-0.126188	-3.285218
H	0.248966	2.158556	1.780220
H	7.241252	-2.085348	-0.432049
H	0.661950	-0.790885	4.918754
H	6.187913	-1.154979	1.627537
H	4.169792	0.188560	-1.688439
H	-5.574038	-3.443689	-1.083712
H	-1.241573	-2.119671	4.762023
H	-0.897078	5.306540	1.882554
H	2.060714	-2.676274	-2.306557
H	5.836836	1.978378	-1.512051
H	-2.331661	2.077773	1.342566
H	-4.600874	0.024821	0.043097
H	1.176205	2.809141	0.053523
H	0.885850	5.573162	-2.033405
H	-4.341438	-4.672124	-2.859551
H	-5.281463	0.622215	3.681261
H	-1.412854	0.178960	-5.765084
H	-3.017237	0.354820	-4.996713
H	-2.600912	-1.159404	-5.853087
H	-2.628458	-4.143087	-0.306184
H	-2.228173	-2.986096	0.977626
H	-1.619518	-4.668019	1.069525
H	-2.505063	-3.560975	-4.113954
H	2.820059	-0.598361	-3.398879
H	2.709098	-1.882929	-4.633809
H	4.305760	-1.400324	-3.984253
H	-5.206631	-0.728417	5.796326
H	-3.184535	-2.120705	6.305577
H	3.655071	1.194007	3.262439
H	3.974938	1.377374	1.515198
H	5.234846	0.702777	2.588252
H	1.908913	0.842790	6.299456

H	1.664572	4.134414	-3.603600
H	1.097156	2.803844	-4.646404
H	1.509472	2.490443	-2.932397
H	2.343979	3.158692	5.441264
H	1.518725	3.807539	3.155555
H	-6.165195	-2.522649	0.808317
H	-5.924646	-1.064449	1.799811
H	-4.525047	-2.093252	1.395546
H	-0.677295	-2.664453	-4.937278
H	-0.018109	-2.329914	-3.303800
H	0.392879	-1.264715	-4.671490
H	-1.381445	3.460828	3.242119
H	-3.044869	2.903568	3.529612
H	-2.752425	4.602779	3.066223
H	-2.102708	4.712619	-4.020189
H	-0.966885	4.129805	-5.275122
H	-0.501238	5.478756	-4.196688
H	3.427017	-5.932938	0.771440
H	3.572827	-4.451032	-0.207696
H	2.243269	-5.605591	-0.517044
H	4.492626	-4.032201	-3.642172
H	2.768838	-4.288065	-4.009947
H	3.434452	-4.818847	-2.437826
H	2.848984	4.596986	0.240645
H	0.391970	-5.925335	1.404529
H	0.516917	-4.951029	2.894375
H	1.741750	-6.193421	2.532556
H	-6.393467	0.389562	-1.673850
H	-7.094467	0.301371	-0.030149
H	-7.221065	-1.101816	-1.136927
H	-4.377339	4.375393	1.062456
H	-4.714633	2.757717	1.744002
H	-4.300388	2.923683	0.019996
H	-2.325526	-0.768620	0.426815

Table S61. Cartesian geometry of 3b-TS2 (22.3 kcal/mol) in Figure S145 in Angstrom [Å].

Atomtype	X Coordinates	Y Coordinates	Z Coordinates
Ge	0.935483	-0.606601	-1.051606
H	-0.082422	0.392524	0.033321
Ni	-1.761394	0.274892	0.144993
H	-3.181886	0.169499	0.420015
P	-1.486394	-0.208088	2.247488
C	-0.762825	-1.865613	2.609159
Si	-0.017614	-2.899724	1.151620
N	1.276811	-2.079340	0.304729
C	2.640299	-2.455601	0.487718
C	3.292472	-3.294474	-0.483007
C	4.646453	-3.633674	-0.302034
C	5.376085	-3.173278	0.800739
C	4.750182	-2.340169	1.733834
C	3.401942	-1.958150	1.593047
C	-2.428357	0.956684	-1.493213
N	-2.383163	2.272204	-1.895523
C	-3.213112	2.504317	-2.994477
C	-3.790232	1.309351	-3.304346
N	-3.299958	0.379349	-2.386791
C	-1.515748	3.293904	-1.362044
C	-0.393892	3.669128	-2.142653
C	0.415423	4.711803	-1.653103
C	0.129741	5.335828	-0.434512

C	-0.985003	4.940913	0.319870
C	-1.842456	3.916875	-0.126258
C	-3.749047	-0.990782	-2.399995
C	-4.972069	-1.293432	-1.753954
C	-5.487591	-2.594223	-1.916221
C	-4.803702	-3.548145	-2.680547
C	-3.580029	-3.226669	-3.285685
C	-3.019800	-1.940497	-3.159992
C	-0.074357	3.028258	-3.495850
C	-0.426899	4.000113	-4.645346
C	-3.065373	3.475675	0.683916
C	-4.394376	3.890271	0.012651
C	-5.741720	-0.246006	-0.950539
C	-6.973974	0.250604	-1.737825
C	-1.722449	-1.561882	-3.873604
C	-0.690822	-2.702003	-3.926481
C	2.576740	-3.793142	-1.743310
C	2.790015	-5.296393	-2.025311
C	2.808064	-0.987206	2.613471
C	3.629033	0.315258	2.741617
C	2.616101	0.574934	-0.643190
C	2.607879	1.945825	-0.294985
C	3.812077	2.685421	-0.204647
C	5.050284	2.070364	-0.437283
C	5.074270	0.718376	-0.807713
C	3.878747	-0.003688	-0.926839
C	3.799431	4.163402	0.116774
F	2.976633	4.461236	1.158145
C	6.413466	0.047156	-1.031481
F	7.010835	-0.279118	0.145761
C	-0.381799	1.014038	3.103339
C	0.014655	0.848155	4.447311
C	0.785530	1.830864	5.086234
C	1.152656	2.999711	4.395656
C	0.755329	3.174533	3.061087
C	0.003511	2.180496	2.414886
C	-2.991653	-0.140987	3.350970
C	-4.113567	0.618349	2.965285
C	-5.232126	0.731150	3.806852
C	-5.249256	0.077372	5.049377
C	-4.140178	-0.691659	5.442072
C	-3.020683	-0.799003	4.601301
C	1.392098	2.568585	-3.617492
C	-3.056004	3.949638	2.146617
C	-6.142813	-0.761176	0.445454
C	-2.016518	-1.036237	-5.297517
C	-1.480117	-3.262232	-0.046489
C	-2.872097	-3.209987	0.619041
C	0.572775	-4.468297	2.120095
C	-0.588581	-5.160843	2.872800
C	-1.301155	-4.595869	-0.803608
C	1.385536	-5.518048	1.339127
C	2.996995	-2.982863	-2.993010
C	2.664188	-1.637483	4.006336
F	5.026008	4.640184	0.436459
F	3.364482	4.911087	-0.941324
F	6.309634	-1.091313	-1.756882
F	7.278625	0.866328	-1.688819
H	1.660423	2.456687	-0.084734
H	5.980085	2.639490	-0.350242

H	3.956398	-1.048417	-1.234506
H	-1.449796	-2.437278	-0.791945
H	-4.502204	1.027292	-4.078515
H	-3.306327	3.492115	-3.442047
H	-4.117023	1.110133	1.987166
H	-0.701691	2.125927	-3.607617
H	-1.592381	-2.480472	2.996475
H	-0.015862	-1.777472	3.415563
H	3.658051	-1.891542	4.421561
H	2.076338	-2.571412	3.971585
H	2.171573	-0.940804	4.709846
H	-1.458085	-5.457419	-0.127900
H	-0.297621	-4.708574	-1.250775
H	-2.043014	-4.681521	-1.619451
H	0.790960	6.126895	-0.062559
H	5.146985	-4.259883	-1.050918
H	1.805457	-0.705082	2.249586
H	1.254747	-4.044988	2.885447
H	-1.254480	-0.743268	-3.295899
H	-0.301492	2.317240	1.370792
H	6.434496	-3.438260	0.915525
H	-0.278341	-0.047691	5.006529
H	5.329336	-1.953408	2.581985
H	-6.436742	-2.861427	-1.436914
H	-2.170302	-1.410335	4.926444
H	-1.182567	5.435890	1.275506
H	1.497903	-3.623376	-1.586570
H	-3.024216	2.368420	0.694558
H	-5.072564	0.618701	-0.795919
H	1.292967	5.024575	-2.224906
H	-5.221517	-4.556105	-2.798497
H	-6.096411	1.325176	3.482555
H	-1.078404	-0.713991	-5.787642
H	-2.706291	-0.174225	-5.284512
H	-2.473676	-1.830673	-5.918149
H	-3.655736	-3.515298	-0.098128
H	-3.122914	-2.186309	0.948381
H	-2.943417	-3.882068	1.495329
H	-3.054754	-3.986990	-3.873263
H	2.681304	-1.926657	-2.924740
H	2.533291	-3.407186	-3.903805
H	4.095712	-3.003239	-3.121501
H	-6.124607	0.159409	5.706285
H	-4.146268	-1.214368	6.407284
H	3.066075	1.059127	3.334911
H	3.844226	0.753296	1.755658
H	4.594588	0.135856	3.251766
H	1.097874	1.684477	6.128129
H	2.096413	3.419598	-3.578055
H	1.542827	2.054166	-4.584827
H	1.664238	1.868574	-2.812374
H	1.751597	3.770336	4.897338
H	1.030744	4.079754	2.512808
H	-6.883618	-1.580012	0.383835
H	-6.598519	0.054607	1.036341
H	-5.263448	-1.128888	1.000283
H	-1.024215	-3.535362	-4.573478
H	-0.479990	-3.098084	-2.920797
H	0.260670	-2.321267	-4.336224
H	-2.127417	3.662552	2.667785

H	-3.900464	3.492359	2.691772
H	-3.174329	5.047630	2.218045
H	-1.481400	4.327503	-4.603759
H	-0.253657	3.519073	-5.626355
H	0.206248	4.905865	-4.593022
H	1.672273	-6.355811	2.007212
H	2.312733	-5.089879	0.929135
H	0.806428	-5.952112	0.504943
H	3.824979	-5.504540	-2.356632
H	2.116452	-5.625944	-2.839536
H	2.587522	-5.919186	-1.139117
H	-1.313288	-5.614002	2.170680
H	-1.151770	-4.479182	3.536103
H	-0.199581	-5.982722	3.507141
H	-6.681183	0.684401	-2.711390
H	-7.514792	1.027212	-1.164353
H	-7.676427	-0.581374	-1.934731
H	-4.483530	4.992946	-0.020635
H	-5.249901	3.497567	0.594788
H	-4.486659	3.505977	-1.016314

Table S62. Cartesian geometry of 3c-TS2 (21.6 kcal/mol) in Figure S145 in Angstrom [Å].

Atomtype	X Coordinates	Y Coordinates	Z Coordinates
C	0.146147	2.559310	2.325717
C	0.282240	1.351750	3.032822
C	0.852683	1.382066	4.324272
C	1.270923	2.596293	4.889487
C	1.127028	3.798747	4.173406
C	0.567567	3.775428	2.887057
P	-0.385703	-0.225010	2.282682
Ni	-1.094513	-0.032050	0.245081
C	-2.343925	0.257516	-1.163565
N	-2.818756	1.454032	-1.657804
C	-3.856002	1.258674	-2.573271
C	-4.028911	-0.088785	-2.684932
N	-3.101652	-0.681296	-1.829231
C	-2.310649	2.773245	-1.369612
C	-1.610753	3.445766	-2.403730
C	-1.197482	4.770364	-2.155778
C	-1.449481	5.384270	-0.925800
C	-2.123383	4.687539	0.089641
C	-2.582028	3.370889	-0.106965
C	-2.947805	-2.113664	-1.755594
C	-3.764254	-2.840209	-0.856141
C	-3.695329	-4.245599	-0.907974
C	-2.833911	-4.892920	-1.803729
C	-2.012382	-4.146476	-2.659023
C	-2.050104	-2.738274	-2.657183
C	-1.316487	2.800300	-3.761304
C	0.169641	2.894211	-4.166020
C	-3.308996	2.597425	0.996475
C	-3.263570	3.263565	2.380830
C	-4.709762	-2.132386	0.111548
C	-4.702730	-2.769095	1.513459
C	-1.202433	-1.923692	-3.632958
C	-1.933030	-1.752655	-4.984195
C	-1.734755	-0.588976	3.518068
C	-3.066100	-0.272161	3.185080
C	-4.095906	-0.415214	4.128158
C	-3.812408	-0.906856	5.412906

C	-2.492459	-1.258838	5.744977
C	-1.459893	-1.095966	4.806895
C	0.908842	-1.496897	2.630170
Si	1.792787	-2.216840	1.068808
C	3.217977	-3.240323	1.877341
C	4.267947	-3.875857	0.946446
C	0.493776	-3.305709	0.156018
C	1.140112	-4.423382	-0.690673
N	2.382039	-0.922471	0.025283
C	3.776461	-0.650988	-0.105055
C	4.462702	0.176685	0.838245
C	5.843388	0.405822	0.682109
C	6.558222	-0.127540	-0.396569
C	5.880353	-0.899372	-1.349109
C	4.504780	-1.173918	-1.229696
C	3.731992	0.861841	1.991047
C	4.189147	0.333037	3.366792
C	3.822932	-1.978429	-2.340658
C	3.646354	-1.129438	-3.620643
C	-0.590745	-3.899224	1.080080
Ge	1.119271	0.136440	-1.142893
C	2.139082	1.937273	-1.195852
C	1.480607	3.145302	-0.855982
C	2.105427	4.396874	-0.984612
C	3.421374	4.483287	-1.468178
C	4.090453	3.304574	-1.835990
C	3.455790	2.056539	-1.707466
C	3.884608	2.397001	1.942344
C	4.549065	-3.294199	-2.691794
C	-2.208599	3.418699	-4.861867
C	-4.777996	2.268635	0.643951
C	-6.142874	-2.077417	-0.462292
C	0.202769	-2.509886	-3.851878
C	2.667937	-4.309023	2.851458
H	-0.024215	-2.606306	-0.533442
H	-4.716291	-0.681885	-3.286170
H	-4.369319	2.095316	-3.043396
H	-3.286740	0.063980	2.167308
H	-1.560318	1.725464	-3.689131
H	0.415326	-2.337360	3.147652
H	1.666505	-1.081789	3.317033
H	5.259383	0.555841	3.539841
H	4.062301	-0.760850	3.453216
H	3.608521	0.813758	4.175749
H	1.565207	-5.216337	-0.046296
H	1.953681	-4.057362	-1.341415
H	0.382313	-4.901524	-1.341285
H	-1.108795	6.412192	-0.746975
H	6.432906	-1.296437	-2.210415
H	2.658832	0.640162	1.873180
H	3.750219	-2.480226	2.484779
H	-1.057812	-0.921935	-3.188709
H	-0.300826	2.537803	1.327742
H	7.633367	0.067867	-0.502798
H	0.975177	0.458480	4.901425
H	6.365979	1.033043	1.416032
H	4.022153	1.167363	-1.998226
H	-4.317465	-4.842578	-0.231842
H	-0.436509	-1.373918	5.086385
H	-2.297491	5.184281	1.049048

H	2.815626	-2.238398	-1.975868
H	5.119196	3.349724	-2.218328
H	-2.756358	1.637402	1.070866
H	-4.347684	-1.094283	0.220914
H	0.448024	3.123413	-0.495893
H	-0.648196	5.315067	-2.931688
H	-2.791678	-5.989401	-1.825664
H	-5.125559	-0.154005	3.851433
H	-1.328234	-1.131030	-5.671562
H	-2.916011	-1.265125	-4.861157
H	-2.096896	-2.736341	-5.464567
H	-1.278687	-4.541141	0.498550
H	-1.208069	-3.110661	1.545250
H	-0.157725	-4.520466	1.887554
H	-1.334535	-4.666221	-3.344655
H	2.975334	-0.268181	-3.453314
H	3.214495	-1.740518	-4.435562
H	4.621032	-0.736315	-3.967579
H	-4.617013	-1.026487	6.149730
H	-2.263892	-1.660469	6.740723
H	3.274003	2.859946	2.737095
H	3.553603	2.800875	0.972969
H	4.936760	2.701482	2.101921
H	1.717502	2.602755	5.892106
H	0.486265	3.941476	-4.325305
H	0.329801	2.350884	-5.116277
H	0.829005	2.458935	-3.398776
H	1.462521	4.745710	4.615132
H	0.461058	4.700220	2.306591
H	-5.134783	-3.787116	1.505093
H	-5.307413	-2.161784	2.209670
H	-3.677550	-2.825277	1.917645
H	3.918782	5.458172	-1.559794
H	0.173132	-3.468649	-4.403301
H	0.718540	-2.671172	-2.892022
H	0.810484	-1.804544	-4.444594
H	-2.236465	3.510949	2.693680
H	-3.680451	2.571582	3.134098
H	-3.871040	4.188878	2.402915
H	-3.283986	3.337128	-4.621792
H	-2.033333	2.914443	-5.830852
H	-1.976310	4.492817	-4.990915
H	5.044543	-4.395036	1.544749
H	4.776987	-3.121240	0.326904
H	3.818848	-4.630029	0.275523
H	5.526486	-3.105714	-3.175079
H	3.939947	-3.882135	-3.404885
H	4.726623	-3.916359	-1.799725
H	1.555979	5.305316	-0.703650
H	2.127792	-5.107948	2.308832
H	1.977372	-3.895732	3.609291
H	3.499490	-4.797360	3.398843
H	-6.168617	-1.562598	-1.439471
H	-6.817747	-1.535852	0.227692
H	-6.544584	-3.098787	-0.604816
H	-5.366912	3.199513	0.535944
H	-5.232238	1.674548	1.459456
H	-4.878725	1.687360	-0.285946
H	-1.983577	-1.135424	0.664614
H	-0.185345	1.169183	-0.067239

Table S63. Cartesian geometry of 3d-TS2 (21.4 kcal/mol) in Figure S145 in Angstrom [Å].

Atomtype	X Coordinates	Y Coordinates	Z Coordinates
C	-1.859238	-1.504036	4.617498
C	-2.009038	-0.802347	3.401396
C	-3.261172	-0.233184	3.098372
C	-4.329343	-0.323259	4.004760
C	-4.168380	-1.011042	5.218680
C	-2.932866	-1.612046	5.516497
P	-0.580177	-0.514505	2.237502
C	0.308209	0.826568	3.189431
C	0.387431	2.118537	2.640390
C	0.980868	3.171211	3.355357
C	1.498671	2.943958	4.639284
C	1.427002	1.654851	5.198311
C	0.837095	0.603945	4.479704
Ni	-1.174398	0.045671	0.230806
C	-2.290906	0.717709	-1.157557
N	-2.535425	2.030155	-1.502958
C	-3.545691	2.130865	-2.462983
C	-3.935790	0.857582	-2.753739
N	-3.162844	0.013067	-1.958527
C	-1.832981	3.196425	-1.025170
C	-0.984212	3.872117	-1.938881
C	-0.370330	5.062477	-1.499355
C	-0.575044	5.541518	-0.202563
C	-1.403261	4.841840	0.689102
C	-2.063084	3.660258	0.300526
C	-3.259636	-1.422462	-2.061171
C	-4.223429	-2.096802	-1.273637
C	-4.391660	-3.476981	-1.495484
C	-3.617416	-4.154189	-2.447116
C	-2.648311	-3.466178	-3.189370
C	-2.446377	-2.082819	-3.015675
C	-0.738508	3.374586	-3.366594
C	0.759323	3.273350	-3.723592
C	-2.964078	2.886032	1.266810
C	-2.873377	3.343967	2.731258
C	-5.074626	-1.353079	-0.247036
C	-5.242393	-2.143505	1.063497
C	-1.436070	-1.318537	-3.869576
C	-2.073082	-0.873832	-5.205774
Ge	1.099668	0.011340	-1.100959
C	2.399683	1.603556	-0.889432
C	1.944105	2.848931	-0.393326
C	2.774671	3.979062	-0.334964
C	4.111565	3.923953	-0.775764
C	4.570540	2.699366	-1.301323
C	3.735637	1.571958	-1.361598
C	5.031545	5.119968	-0.659594
N	2.123906	-1.380959	-0.050088
C	3.547639	-1.330584	-0.121320
C	4.323869	-0.750956	0.931289
C	5.728280	-0.736660	0.828907
C	6.387153	-1.243434	-0.297137
C	5.629043	-1.768180	-1.351801
C	4.223636	-1.823529	-1.291414
C	3.672210	-0.101580	2.150297
C	3.981781	-0.868201	3.453270
C	3.463167	-2.361554	-2.507399

C	3.475758	-1.344882	-3.672082
C	0.478405	-2.015635	2.436138
Si	1.290719	-2.674252	0.809640
C	-0.135272	-3.407995	-0.256594
C	0.347815	-4.510819	-1.223097
C	2.493713	-4.017435	1.504592
C	3.458333	-4.699290	0.515951
C	-1.469119	4.275852	-4.388270
C	-4.448167	2.871722	0.833796
C	-6.448681	-0.975173	-0.843183
C	-0.140259	-2.106212	-4.127695
C	4.079048	1.379540	2.304126
C	3.979113	-3.726501	-3.010072
C	1.737504	-5.090243	2.323287
C	-1.340760	-3.911778	0.565081
H	-0.047227	1.085311	0.087454
H	-0.499124	-2.553410	-0.864900
H	-4.683534	0.470729	-3.444338
H	-3.889105	3.094926	-2.833184
H	-3.393762	0.259430	2.130184
H	-1.156295	2.355589	-3.450410
H	-0.164003	-2.820635	2.832353
H	1.267444	-1.820952	3.182948
H	5.066961	-0.855165	3.670604
H	3.670460	-1.926381	3.395075
H	3.459936	-0.400396	4.308514
H	0.607611	-5.436248	-0.674695
H	1.236752	-4.214222	-1.807141
H	-0.452565	-4.770614	-1.942954
H	-0.077458	6.463148	0.125594
H	6.141635	-2.141907	-2.247590
H	2.582620	-0.121911	1.985504
H	3.121341	-3.440758	2.214211
H	-1.145119	-0.412927	-3.306587
H	-0.027724	2.294347	1.643968
H	7.483005	-1.217601	-0.359484
H	0.793256	-0.391417	4.936310
H	6.317858	-0.300593	1.645864
H	4.159238	0.651060	-1.772601
H	-5.132621	-4.032318	-0.909689
H	-0.902906	-1.977992	4.869159
H	-1.538997	5.229414	1.703401
H	2.413935	-2.493635	-2.196258
H	5.605531	2.620188	-1.663420
H	-2.589080	1.842071	1.220850
H	-4.542975	-0.416689	0.000327
H	0.909710	2.959406	-0.056005
H	0.297537	5.603788	-2.178570
H	-3.760871	-5.231199	-2.602076
H	-5.294662	0.134977	3.753471
H	-1.345707	-0.291433	-5.802751
H	-2.964724	-0.242403	-5.046297
H	-2.380137	-1.754487	-5.801952
H	-2.101026	-4.356192	-0.104261
H	-1.837450	-3.088780	1.108510
H	-1.050367	-4.686573	1.300527
H	-2.042212	-4.011238	-3.921029
H	2.947675	-0.411327	-3.408165
H	2.981482	-1.771272	-4.565372
H	4.513839	-1.079642	-3.949479

H	-5.004237	-1.088911	5.925715
H	-2.802503	-2.166625	6.454853
H	3.517741	1.838388	3.136753
H	3.863903	1.949329	1.386927
H	5.158742	1.478426	2.527571
H	1.838864	1.464615	6.197761
H	1.247920	4.265139	-3.727839
H	0.870074	2.844667	-4.737392
H	1.305459	2.634696	-3.011793
H	1.968055	3.761794	5.200779
H	1.040204	4.165629	2.895862
H	-5.847656	-3.057614	0.917937
H	-5.760060	-1.524236	1.817046
H	-4.262338	-2.433041	1.479751
H	-0.306954	-2.977787	-4.788539
H	0.303300	-2.459466	-3.183414
H	0.599088	-1.451824	-4.620907
H	-1.835773	3.359953	3.101861
H	-3.441297	2.644467	3.370070
H	-3.311296	4.351750	2.866516
H	-2.552796	4.345208	-4.184389
H	-1.336533	3.884033	-5.414337
H	-1.058763	5.303096	-4.361192
H	4.115263	-5.413476	1.053562
H	4.108007	-3.969313	0.008445
H	2.916284	-5.275750	-0.255053
H	4.991909	-3.644902	-3.448338
H	3.310359	-4.113702	-3.802588
H	4.019366	-4.473873	-2.201239
H	2.371542	4.921032	0.062805
H	1.092675	-5.712230	1.673904
H	1.097387	-4.662747	3.116675
H	2.455047	-5.776840	2.816579
H	-6.339778	-0.350918	-1.748161
H	-7.049438	-0.408110	-0.106722
H	-7.015299	-1.883985	-1.122293
H	-4.864251	3.897267	0.847089
H	-5.036910	2.259015	1.542569
H	-4.599327	2.451739	-0.172982
H	-2.264101	-0.914871	0.496820
H	5.745030	5.168491	-1.503515
H	4.466135	6.069913	-0.628832
H	5.634154	5.066499	0.270134

Table S64. Cartesian geometry of 3e-TS2 (21.7 kcal/mol) in Figure S145 in Angstrom [Å].

Atomtype	X Coordinates	Y Coordinates	Z Coordinates
C	-2.345927	-1.526330	4.463979
C	-2.326357	-0.719455	3.305421
C	-3.446728	0.086329	3.023313
C	-4.545038	0.121003	3.896748
C	-4.551234	-0.674542	5.054248
C	-3.452792	-1.507834	5.328510
P	-0.831316	-0.604580	2.196276
C	0.240216	0.498644	3.258478
C	0.576196	1.782557	2.794360
C	1.318281	2.667275	3.593148
C	1.728801	2.277866	4.876927
C	1.400755	0.994494	5.351277
C	0.662984	0.110933	4.548861
Ni	-1.252500	0.181479	0.221629

C	-2.184752	1.126240	-1.143238
N	-2.187946	2.480836	-1.401036
C	-3.135436	2.818791	-2.370808
C	-3.730157	1.654903	-2.757421
N	-3.139313	0.638220	-2.008804
C	-1.308895	3.473669	-0.832388
C	-0.332433	4.050132	-1.684610
C	0.463519	5.086293	-1.155750
C	0.309647	5.508114	0.167665
C	-0.649347	4.905227	0.996765
C	-1.491829	3.883425	0.518222
C	-3.480237	-0.748463	-2.212628
C	-4.573439	-1.291654	-1.495940
C	-4.972372	-2.603229	-1.817141
C	-4.296175	-3.343301	-2.796199
C	-3.196898	-2.790560	-3.466766
C	-2.762389	-1.478875	-3.192521
C	-0.134412	3.609004	-3.138067
C	1.332468	3.264408	-3.471078
C	-2.538540	3.216390	1.415189
C	-2.414157	3.561075	2.907840
C	-5.316979	-0.477872	-0.439291
C	-5.658720	-1.308082	0.811541
C	-1.604985	-0.851183	-3.967551
C	-2.109089	-0.205327	-5.278155
Ge	1.033848	-0.156493	-1.072380
C	2.575144	1.168128	-0.717545
C	2.320850	2.446395	-0.155479
C	3.320378	3.411710	0.002200
C	4.643130	3.138011	-0.406508
C	4.928068	1.889185	-0.994052
C	3.902923	0.936520	-1.145518
O	5.558112	4.133872	-0.198586
C	6.907899	3.898938	-0.558481
N	1.763600	-1.776043	-0.095011
C	3.175786	-1.965993	-0.127384
C	4.000927	-1.597070	0.981726
C	5.390707	-1.813826	0.913506
C	5.994745	-2.349369	-0.230007
C	5.196921	-2.667553	-1.336865
C	3.800865	-2.487806	-1.313407
C	3.423713	-0.930243	2.228482
C	3.553499	-1.823817	3.479844
C	3.003345	-2.804372	-2.582316
C	3.229456	-1.727715	-3.668612
C	-0.047563	-2.274198	2.310020
Si	0.690872	-2.953801	0.656438
C	-0.807642	-3.352955	-0.485869
C	-0.492629	-4.457331	-1.517952
C	1.620924	-4.531482	1.274501
C	2.480653	-5.302169	0.255072
C	-0.661944	4.689353	-4.109778
C	-3.990539	3.482493	0.955450
C	-6.586858	0.167555	-1.036883
C	-0.462907	-1.839083	-4.260607
C	4.065336	0.447002	2.498780
C	3.298113	-4.199800	-3.171999
C	0.667589	-5.508376	2.002212
C	-2.107690	-3.691407	0.274399
H	0.055641	0.997465	0.172302

H	-0.999067	-2.410036	-1.040269
H	-4.511758	1.450550	-3.487528
H	-3.294649	3.850118	-2.679721
H	-3.456700	0.666284	2.095319
H	-0.722656	2.688344	-3.300014
H	-0.828723	-2.982906	2.634387
H	0.737765	-2.266144	3.085556
H	4.617053	-2.003472	3.728277
H	3.078014	-2.810280	3.335486
H	3.080195	-1.335482	4.351814
H	-0.414959	-5.447953	-1.030860
H	0.452773	-4.283203	-2.061578
H	-1.302392	-4.525105	-2.270216
H	0.949648	6.305952	0.565660
H	5.670748	-3.063100	-2.244618
H	2.352730	-0.755148	2.035861
H	2.318608	-4.122519	2.033547
H	-1.174843	-0.055615	-3.332022
H	0.243897	2.086369	1.797817
H	7.080931	-2.506346	-0.264291
H	0.418648	-0.883354	4.939990
H	6.015602	-1.539314	1.773436
H	4.183123	-0.018700	-1.598784
H	-5.818585	-3.055725	-1.288213
H	-1.498201	-2.181799	4.697252
H	-0.743006	5.243553	2.033170
H	1.936593	-2.775192	-2.305161
H	5.939128	1.634594	-1.328643
H	-2.344732	2.128282	1.307378
H	-4.641483	0.335448	-0.118559
H	1.308785	2.714841	0.160202
H	1.232531	5.547014	-1.785622
H	-4.620109	-4.365715	-3.029234
H	-5.404603	0.762397	3.662767
H	-1.270866	0.278485	-5.814768
H	-2.879458	0.562529	-5.088644
H	-2.547522	-0.972040	-5.945421
H	-2.912199	-3.949477	-0.439557
H	-2.471093	-2.832783	0.866130
H	-1.979796	-4.553035	0.957665
H	-2.670695	-3.385664	-4.220868
H	2.865574	-0.735901	-3.346237
H	2.695993	-1.996987	-4.599770
H	4.306132	-1.632082	-3.906859
H	-5.412103	-0.654830	5.734793
H	-3.455626	-2.146187	6.221582
H	3.566986	0.931618	3.356295
H	3.969344	1.109646	1.624775
H	5.140640	0.348998	2.742714
H	1.726556	0.676986	6.350180
H	1.990042	4.149694	-3.392012
H	1.398446	2.893231	-4.511195
H	1.731535	2.489767	-2.797327
H	2.313125	2.963443	5.503802
H	1.578082	3.657336	3.198577
H	-6.405919	-2.093486	0.592459
H	-6.086876	-0.657253	1.594139
H	-4.756168	-1.787399	1.227710
H	-0.761428	-2.617115	-4.988470
H	-0.119452	-2.332808	-3.337964

H	0.396454	-1.295150	-4.689355
H	-1.400390	3.375567	3.297693
H	-3.111540	2.931928	3.489024
H	-2.677638	4.618973	3.100484
H	-1.722064	4.937837	-3.921907
H	-0.569517	4.346122	-5.157444
H	-0.077477	5.623058	-4.005349
H	2.989962	-6.154219	0.750404
H	3.259917	-4.663034	-0.188348
H	1.869467	-5.722171	-0.563839
H	4.325872	-4.263948	-3.577201
H	2.603662	-4.410148	-4.007909
H	3.179933	-4.997204	-2.420585
H	3.098474	4.390281	0.444055
H	-0.057705	-5.961885	1.300264
H	0.090252	-5.030092	2.814561
H	1.240816	-6.342300	2.455753
H	-6.344549	0.818680	-1.895892
H	-7.105118	0.782714	-0.276732
H	-7.291088	-0.610898	-1.387589
H	-4.226411	4.561590	1.025950
H	-4.694977	2.939082	1.613390
H	-4.182244	3.154952	-0.078373
H	-2.507161	-0.574334	0.400394
H	7.469721	4.810617	-0.290740
H	7.337904	3.034093	-0.009628
H	7.020889	3.712538	-1.647991

Table S65. Cartesian geometry of 3f-TS2 (22.2 kcal/mol) in Figure S145 in Angstrom [Å].

Atomtype	X Coordinates	Y Coordinates	Z Coordinates
C	-2.540984	-1.049688	4.552378
C	-2.442135	-0.305732	3.356143
C	-3.454850	0.625157	3.053731
C	-4.521558	0.840175	3.940779
C	-4.605505	0.105557	5.134835
C	-3.617179	-0.848972	5.432120
P	-0.969080	-0.444815	2.219233
C	0.267666	0.540262	3.216012
C	0.761895	1.747913	2.692092
C	1.635714	2.554437	3.438568
C	2.022179	2.163390	4.729275
C	1.536491	0.955650	5.263173
C	0.666932	0.149563	4.512829
Ni	-1.303681	0.312393	0.216846
C	-2.085222	1.332291	-1.186854
N	-1.890529	2.662645	-1.494778
C	-2.778499	3.098499	-2.481992
C	-3.536996	2.020353	-2.827972
N	-3.101006	0.957208	-2.038805
C	-0.880885	3.538252	-0.951125
C	0.172828	3.941447	-1.811111
C	1.109499	4.865068	-1.304672
C	1.012476	5.344139	0.004562
C	-0.029667	4.914817	0.841210
C	-1.009790	4.012826	0.384605
C	-3.650897	-0.368461	-2.181894
C	-4.808220	-0.706121	-1.439829
C	-5.403377	-1.955571	-1.698742
C	-4.855123	-2.833397	-2.643407
C	-3.691746	-2.483862	-3.342006

C	-3.061363	-1.241983	-3.129597
C	0.307316	3.438331	-3.251642
C	1.713107	2.891109	-3.576254
C	-2.150032	3.536925	1.289660
C	-1.980130	3.898631	2.773840
C	-5.413581	0.259479	-0.423544
C	-5.865271	-0.449480	0.866346
C	-1.830939	-0.831184	-3.936675
C	-2.246531	-0.174724	-5.272812
Ge	0.907039	-0.429895	-1.092809
C	2.618980	0.665668	-0.796692
C	2.562877	1.980831	-0.277353
C	3.688365	2.804544	-0.153059
C	4.975079	2.352756	-0.559363
C	5.045377	1.040892	-1.106968
C	3.901598	0.239929	-1.222850
N	6.105666	3.153337	-0.426847
C	5.998165	4.443232	0.227191
N	1.400088	-2.096609	-0.044997
C	2.769644	-2.489184	-0.076442
C	3.654028	-2.191835	1.008445
C	4.997296	-2.609074	0.942381
C	5.504061	-3.277492	-0.178089
C	4.654773	-3.527699	-1.263785
C	3.299239	-3.148592	-1.240352
C	3.195104	-1.392465	2.226018
C	3.205834	-2.241082	3.514823
C	2.449324	-3.405651	-2.488274
C	2.814709	-2.423664	-3.625046
C	-0.428240	-2.203261	2.396541
Si	0.172886	-3.058994	0.768982
C	-1.385311	-3.271295	-0.343408
C	-1.255341	-4.452876	-1.328743
C	0.855368	-4.733092	1.452674
C	1.587514	-5.660683	0.464885
C	-0.071878	4.554792	-4.251784
C	-3.541167	4.010548	0.809450
C	-6.577205	1.058306	-1.051246
C	-0.854433	-1.992165	-4.190258
C	4.035361	-0.114465	2.431806
C	2.533658	-4.854811	-3.012514
C	-0.232112	-5.527805	2.213073
C	-2.711783	-3.373640	0.439026
C	7.415071	2.605557	-0.732831
H	0.127019	0.881527	0.116804
H	-1.437195	-2.333922	-0.936950
H	-4.341015	1.905163	-3.553203
H	-2.786236	4.129667	-2.829643
H	-3.409638	1.160083	2.100094
H	-0.400739	2.601718	-3.388694
H	-1.296234	-2.776652	2.764872
H	0.365342	-2.274168	3.160336
H	4.234244	-2.565495	3.764537
H	2.588580	-3.151790	3.416239
H	2.820761	-1.651975	4.367768
H	-1.322006	-5.422744	-0.799912
H	-0.301728	-4.447091	-1.885874
H	-2.075884	-4.428382	-2.071897
H	1.760126	6.051902	0.385505
H	5.055678	-4.027519	-2.155104

H	2.159947	-1.069128	2.029292
H	1.608133	-4.405303	2.198497
H	-1.278398	-0.083144	-3.338978
H	0.449596	2.055741	1.690318
H	6.556235	-3.589671	-0.211797
H	0.302467	-0.787107	4.949777
H	5.666151	-2.387425	1.784304
H	4.043356	-0.760302	-1.643359
H	-6.303700	-2.249856	-1.147841
H	-1.780370	-1.798858	4.802937
H	-0.078767	5.294635	1.866215
H	1.401208	-3.211109	-2.207169
H	6.001201	0.623332	-1.438015
H	-2.126654	2.429434	1.211981
H	-4.622162	0.977273	-0.142826
H	1.604624	2.403203	0.038099
H	1.940685	5.188508	-1.940886
H	-5.331367	-3.804901	-2.827707
H	-5.296515	1.575893	3.689492
H	-1.350889	0.151910	-5.834754
H	-2.889549	0.708819	-5.115063
H	-2.803681	-0.894722	-5.902542
H	-3.555926	-3.533765	-0.257831
H	-2.930706	-2.445252	0.995430
H	-2.708802	-4.215367	1.158060
H	-3.268696	-3.185239	-4.069200
H	2.603178	-1.376057	-3.346211
H	2.235551	-2.654032	-4.539277
H	3.890688	-2.496478	-3.874208
H	-5.442477	0.266867	5.826304
H	-3.682832	-1.440466	6.354587
H	3.617051	0.479667	3.263023
H	4.036253	0.511200	1.525890
H	5.084907	-0.359558	2.684972
H	1.841916	0.635585	6.267683
H	2.483068	3.682952	-3.525340
H	1.722659	2.484947	-4.605253
H	2.009384	2.090541	-2.880287
H	2.708742	2.787267	5.315785
H	2.015368	3.484323	2.997350
H	-6.724497	-1.122355	0.686902
H	-6.183305	0.294825	1.617723
H	-5.041495	-1.039761	1.302763
H	-1.275622	-2.747982	-4.879901
H	-0.577729	-2.489340	-3.247325
H	0.071792	-1.603704	-4.647835
H	-1.009288	3.567268	3.176477
H	-2.769676	3.402624	3.365662
H	-2.075830	4.989489	2.936988
H	-1.087165	4.951595	-4.071483
H	-0.030441	4.174684	-5.289929
H	0.635446	5.402005	-4.171768
H	1.965031	-6.560927	0.992027
H	2.451858	-5.161356	0.000037
H	0.917802	-6.013597	-0.339730
H	3.535898	-5.084486	-3.421678
H	1.804613	-5.002248	-3.832356
H	2.313494	-5.591197	-2.222657
H	3.549251	3.804267	0.268522
H	-1.019183	-5.892388	1.526065

H	-0.729192	-4.938143	3.005152
H	0.209982	-6.421141	2.699216
H	-6.246316	1.624057	-1.940668
H	-6.991815	1.779811	-0.321568
H	-7.392640	0.378419	-1.363924
H	-3.610798	5.114271	0.855034
H	-4.325068	3.594626	1.470508
H	-3.772026	3.693074	-0.219560
H	-2.665519	-0.208486	0.444147
H	8.189222	3.371185	-0.558700
H	7.660592	1.718937	-0.107165
H	7.489635	2.290054	-1.794295
H	6.983785	4.937241	0.243510
H	5.296017	5.117187	-0.307755
H	5.637847	4.360601	1.277470

Table S66. Cartesian geometry of 3g-TS2 (24.6 kcal/mol) in Figure S145 in Angstrom [Å].

Atomtype	X Coordinates	Y Coordinates	Z Coordinates
C	-0.780566	4.544151	-0.401218
C	-0.182502	3.338696	0.026000
C	0.152528	3.190020	1.383490
C	-0.098598	4.220621	2.303377
C	-0.684837	5.419849	1.870901
C	-1.028462	5.577161	0.515324
P	0.264393	1.977100	-1.164789
Ni	1.106562	0.227604	-0.196079
C	2.331755	-1.009830	0.554229
N	3.016115	-0.889064	1.743586
C	4.038133	-1.836324	1.850967
C	3.987863	-2.591464	0.717426
N	2.943204	-2.082579	-0.053374
C	2.701277	0.009284	2.826404
C	2.127230	-0.554079	3.993970
C	1.902999	0.306665	5.087378
C	2.211307	1.668079	5.005564
C	2.760890	2.202745	3.829001
C	3.033799	1.387983	2.713845
C	2.576574	-2.673427	-1.315570
C	3.286997	-2.282096	-2.474792
C	3.022131	-2.985720	-3.665998
C	2.076395	-4.018804	-3.696233
C	1.363733	-4.361883	-2.538061
C	1.596109	-3.698621	-1.317606
C	1.778943	-2.041624	4.106754
C	0.318387	-2.294511	4.534847
C	3.643147	1.952014	1.425973
C	3.630042	3.486521	1.337250
C	4.331999	-1.168754	-2.438570
C	4.135733	-0.147790	-3.575958
C	0.872924	-4.110207	-0.034950
C	1.655853	-5.218975	0.705085
C	1.413341	2.909663	-2.302531
C	0.929828	3.786396	-3.298597
C	1.819982	4.524041	-4.096549
C	3.207289	4.401574	-3.907753
C	3.697567	3.525356	-2.925359
C	2.806706	2.780523	-2.136311
C	-1.252048	1.710874	-2.182713
Si	-2.189452	0.026707	-1.966440
C	-3.717614	0.325198	-3.115458

C	-4.830900	-0.739211	-3.101004
C	-0.998428	-1.329253	-2.638827
C	-1.764619	-2.553022	-3.186400
N	-2.628147	-0.320563	-0.308457
Ge	-1.273317	-0.962973	1.103491
N	-1.923506	0.008190	2.667784
C	-3.144756	-0.385797	3.350322
C	0.019123	-0.827128	-3.684723
C	-3.966591	-0.220380	0.160824
C	-4.757760	-1.409522	0.342593
C	-6.075304	-1.296509	0.824535
C	-6.640356	-0.050836	1.128204
C	-5.866138	1.105068	0.970162
C	-4.536360	1.045892	0.511160
C	-4.198710	-2.808390	0.063544
C	-3.923791	-3.580459	1.374172
C	-3.725535	2.339264	0.444617
C	-4.285200	3.336957	-0.590414
C	-5.098672	-3.661377	-0.855945
C	-3.617418	3.017846	1.824265
C	-1.058598	0.777879	3.536361
C	2.755252	-2.753754	5.070233
C	5.079388	1.438681	1.173300
C	5.759736	-1.756986	-2.455018
C	-0.584589	-4.546550	-0.261168
C	-3.305053	0.634835	-4.573555
H	0.028525	0.332028	0.957014
H	-0.412018	-1.660321	-1.753739
H	4.588964	-3.435277	0.381795
H	4.691853	-1.875362	2.720514
H	3.190358	2.067605	-1.399323
H	1.896345	-2.498177	3.108373
H	-0.928099	1.736818	-3.237256
H	-1.954296	2.549377	-2.033387
H	-5.299065	3.677796	-0.305698
H	-4.359168	2.890853	-1.598106
H	-3.635543	4.230367	-0.654018
H	-2.272066	-2.308499	-4.138595
H	-2.536867	-2.924484	-2.489479
H	-1.068362	-3.388208	-3.393063
H	2.016055	2.325857	5.862199
H	-6.673582	-2.206116	0.966201
H	-2.700660	2.063616	0.149610
H	-4.159757	1.251465	-2.695417
H	0.833718	-3.221266	0.621665
H	0.623415	2.260525	1.715702
H	-7.673105	0.016608	1.494752
H	-1.052155	4.688044	-1.453346
H	-6.299557	2.081129	1.226276
H	3.559851	-2.715938	-4.582452
H	-0.148713	3.892925	-3.466615
H	2.982741	3.273433	3.785112
H	-3.231991	-2.668855	-0.448792
H	3.000718	1.563438	0.607196
H	4.202529	-0.622180	-1.487622
H	1.461272	-0.096810	6.005868
H	1.881633	-4.554204	-4.634285
H	4.779365	3.409178	-2.778990
H	1.144912	-5.481486	1.650891
H	2.684599	-4.903657	0.951579

H	1.717078	-6.132092	0.082392
H	0.637868	-1.665712	-4.053841
H	0.714064	-0.085044	-3.253896
H	-0.477861	-0.368396	-4.560978
H	0.619542	-5.164216	-2.581891
H	-3.171240	-3.068975	2.000069
H	-3.545036	-4.597063	1.154280
H	-4.849821	-3.681873	1.972063
H	3.902138	4.979103	-4.530890
H	1.426730	5.195612	-4.870767
H	-2.970893	3.910622	1.760953
H	-3.175488	2.322994	2.553100
H	-4.608433	3.338652	2.198634
H	-1.497242	6.507930	0.170989
H	0.112964	-1.900431	5.547166
H	0.122777	-3.383050	4.556753
H	-0.395282	-1.832977	3.831938
H	-0.885513	6.227857	2.585966
H	0.166576	4.074472	3.357796
H	4.321330	-0.597891	-4.569021
H	4.840505	0.695426	-3.457397
H	3.112193	0.263672	-3.563821
H	-0.651792	-5.504645	-0.810622
H	-1.148071	-3.781638	-0.817005
H	-1.084983	-4.684418	0.713079
H	2.624135	3.905056	1.503243
H	3.958846	3.801463	0.331034
H	4.324952	3.938914	2.070628
H	3.808825	-2.619872	4.764396
H	2.542120	-3.838719	5.105584
H	2.652193	-2.353635	6.096662
H	-5.670249	-0.425064	-3.754984
H	-5.237963	-0.891596	-2.089166
H	-4.472680	-1.713123	-3.480228
H	-6.053041	-3.925884	-0.362125
H	-4.588089	-4.610073	-1.110855
H	-5.338093	-3.138573	-1.796063
H	-2.852898	-0.248580	-5.062654
H	-2.580917	1.466037	-4.655067
H	-4.193084	0.916014	-5.175419
H	5.922716	-2.443054	-1.603692
H	6.514260	-0.949673	-2.394857
H	5.939542	-2.325146	-3.387534
H	5.760900	1.786936	1.972894
H	5.454956	1.835963	0.211129
H	5.137668	0.339562	1.124957
H	2.056007	0.231838	-1.316828
H	-3.707373	0.500926	3.721496
H	-3.832789	-0.930149	2.684594
H	-2.944692	-1.031942	4.243328
H	-1.470647	1.794556	3.748183
H	-0.905610	0.294451	4.530214
H	-0.057114	0.911829	3.100482

Table S67. Cartesian geometry of 3a-INT3 (17.1 kcal/mol) in Figure S145 in Angstrom [Å].

Atomtype	X Coordinates	Y Coordinates	Z Coordinates
C	-1.899867	3.491137	-1.981595
C	-2.627512	2.881554	-0.932049
C	-2.745795	3.452471	0.360667
C	-2.096099	4.680342	0.584528

C	-1.359188	5.305763	-0.434769
C	-1.265432	4.718936	-1.703021
N	-3.233032	1.600628	-1.199065
C	-2.604987	0.390317	-0.972540
N	-3.440059	-0.516663	-1.603391
C	-4.540173	0.114449	-2.187339
C	-4.409751	1.449089	-1.930744
Ni	-0.976430	0.067054	-0.024582
Ge	1.154015	-0.104498	-0.809413
C	2.381577	1.476865	-1.213303
C	1.794301	2.755318	-1.043650
C	2.507103	3.935880	-1.276143
C	3.851932	3.870505	-1.714752
C	4.447267	2.603583	-1.926585
C	3.718691	1.432576	-1.680188
C	-3.144915	-1.913326	-1.818139
C	-3.897601	-2.895461	-1.130707
C	-3.628801	-4.248648	-1.422112
C	-2.632658	-4.607687	-2.335309
C	-1.894700	-3.615643	-2.996871
C	-2.143910	-2.249726	-2.770940
C	-4.996116	-2.538252	-0.129591
C	-4.888377	-3.350160	1.177505
C	-1.407754	-1.170931	-3.564683
C	-2.260297	-0.694660	-4.762721
C	-3.525181	2.724148	1.452081
C	-3.144850	3.124896	2.886399
C	-1.778624	2.857944	-3.368717
C	-0.320652	2.502241	-3.718314
P	-0.451493	-0.106349	2.073842
C	0.441719	1.329254	2.869218
C	1.144167	1.246032	4.091789
C	1.701569	2.395022	4.676300
C	1.575268	3.644466	4.043960
C	0.896843	3.733513	2.817921
C	0.338281	2.584315	2.237498
C	0.526586	-1.608687	2.581598
Si	1.497740	-2.542195	1.193274
C	2.797097	-3.555561	2.189608
C	3.860378	-4.349950	1.404052
N	2.305242	-1.336397	0.138891
C	3.728756	-1.271825	0.011315
C	4.522108	-0.534199	0.942985
C	5.917850	-0.479432	0.758531
C	6.541905	-1.113520	-0.321889
C	5.759793	-1.818366	-1.246306
C	4.362976	-1.914850	-1.101590
C	3.907025	0.254236	2.098299
C	4.443829	-0.191389	3.474962
C	3.562435	-2.660953	-2.170564
C	3.492857	-1.847287	-3.481911
C	0.263963	-3.651584	0.210742
C	0.902905	-4.900701	-0.432488
C	-1.984997	-0.214778	3.145291
C	-1.985611	0.070051	4.527545
C	-3.181505	0.028656	5.263238
C	-4.394395	-0.288105	4.627199
C	-4.399624	-0.581357	3.253385
C	-3.202550	-0.552386	2.520872
C	4.103481	-4.079326	-2.445955

C	4.120418	1.774079	1.921244
C	-6.393759	-2.724652	-0.763042
C	-0.008752	-1.597363	-4.034579
C	-5.051197	2.870298	1.255430
C	-2.416817	3.759105	-4.447047
C	-0.963301	-4.063657	1.051638
C	2.101423	-4.477003	3.219772
H	-0.108835	-3.002185	-0.605000
H	-5.295977	-0.441263	-2.740170
H	-5.025424	2.301847	-2.215170
H	-3.191723	-0.760867	1.445250
H	-2.339018	1.908593	-3.366163
H	-0.192794	-2.314867	3.031908
H	1.246920	-1.347786	3.377191
H	5.524989	0.021971	3.572216
H	4.302532	-1.273796	3.645342
H	3.923231	0.357590	4.281417
H	1.194863	-5.646672	0.330701
H	1.800322	-4.669631	-1.030702
H	0.173056	-5.393447	-1.105972
H	-0.851648	6.258503	-0.234781
H	6.245100	-2.301504	-2.103776
H	2.819905	0.074658	2.083374
H	3.334823	-2.769062	2.756528
H	-1.260695	-0.314730	-2.883412
H	-0.200388	2.646533	1.285302
H	7.630442	-1.051736	-0.448763
H	1.266826	0.283432	4.602425
H	6.527178	0.090388	1.471605
H	4.224255	0.476272	-1.837218
H	-4.200289	-5.032704	-0.912560
H	-1.057508	0.350139	5.037270
H	-2.147465	5.146457	1.574012
H	2.530578	-2.764576	-1.794216
H	5.486629	2.544106	-2.270917
H	-3.270767	1.655762	1.338957
H	-4.884574	-1.469194	0.128281
H	0.747322	2.832463	-0.727325
H	-0.679715	5.211625	-2.488581
H	-2.425713	-5.667109	-2.533126
H	-5.340587	-0.823091	2.744524
H	-1.738633	0.115873	-5.304880
H	-3.241508	-0.306842	-4.438236
H	-2.436349	-1.526114	-5.471619
H	-1.631932	-4.716338	0.459102
H	-1.558225	-3.184544	1.354663
H	-0.676330	-4.621086	1.964542
H	-1.117827	-3.913802	-3.708402
H	2.997592	-0.871863	-3.330227
H	2.923924	-2.397266	-4.254812
H	4.508125	-1.651102	-3.875946
H	-5.330767	-0.301924	5.199781
H	-3.166733	0.260812	6.336124
H	3.618085	2.325391	2.734554
H	3.711051	2.129076	0.963255
H	5.197303	2.027279	1.947593
H	2.246188	2.310835	5.625518
H	0.335719	3.391147	-3.709586
H	-0.269779	2.050144	-4.726407
H	0.085712	1.778312	-2.994139

H	2.018575	4.539268	4.499104
H	0.800334	4.697891	2.303129
H	-5.058776	-4.427988	1.001186
H	-5.653268	-3.014248	1.900483
H	-3.897551	-3.229923	1.645706
C	4.597192	5.076177	-1.947413
H	-0.049498	-2.353353	-4.841863
H	0.582724	-2.012265	-3.200027
H	0.536241	-0.722316	-4.431476
H	-2.056808	3.066113	3.053261
H	-3.631260	2.435759	3.598880
H	-3.480819	4.151919	3.127317
H	-3.472424	3.986150	-4.207058
H	-2.385554	3.259652	-5.433798
H	-1.877027	4.720339	-4.538712
H	4.573339	-4.826460	2.107374
H	4.444373	-3.708284	0.725207
H	3.407292	-5.160105	0.805525
H	5.096517	-4.051117	-2.932484
H	3.419892	-4.624066	-3.124364
H	4.200503	-4.661305	-1.513361
H	2.028888	4.910650	-1.121107
H	1.547070	-5.293394	2.719049
H	1.386592	-3.938543	3.869005
H	2.851951	-4.955092	3.881077
H	-6.513381	-2.119028	-1.679081
H	-7.187860	-2.430923	-0.050598
H	-6.555903	-3.783940	-1.038387
H	-5.356057	3.929170	1.360561
H	-5.588279	2.279659	2.021317
H	-5.379132	2.516015	0.263401
H	-1.408614	-1.349865	0.181618
H	-0.607461	1.500936	-0.236484
N	5.199829	6.063317	-2.137256

Table S68. Cartesian geometry of 3b-INT3 (16.1 kcal/mol) in Figure S145 in Angstrom [Å].

Atomtype	X Coordinates	Y Coordinates	Z Coordinates
C	-1.557166	3.837347	-1.701136
C	-2.471361	3.216358	-0.816775
C	-2.711642	3.685453	0.498292
C	-1.991579	4.818187	0.921830
C	-1.071825	5.448916	0.070886
C	-0.857765	4.963654	-1.224976
N	-3.176453	2.052760	-1.295983
C	-2.734977	0.751164	-1.158743
N	-3.609573	0.039530	-1.964872
C	-4.549015	0.875732	-2.570960
C	-4.277561	2.143732	-2.145704
Ni	-1.278066	0.093707	-0.102903
Ge	0.858642	-0.494529	-0.612937
C	2.458562	0.777884	-0.736718
C	2.203804	2.144162	-0.481330
C	3.234644	3.101175	-0.537916
C	4.548153	2.719550	-0.852718
C	4.810790	1.369698	-1.129175
C	3.780635	0.415510	-1.080957
C	-3.496867	-1.360658	-2.296644
C	-4.474253	-2.267368	-1.819030
C	-4.366845	-3.615468	-2.219217
C	-3.317709	-4.046682	-3.036317

C	-2.362041	-3.128998	-3.495938
C	-2.438762	-1.766002	-3.157030
C	-5.648448	-1.831087	-0.941485
C	-5.854981	-2.752652	0.278132
C	-1.458023	-0.749409	-3.740217
C	-2.080199	-0.016154	-4.950128
C	-3.708257	2.969309	1.403611
C	-3.440021	3.147435	2.906695
C	-1.278149	3.292520	-3.102591
C	0.114607	2.633940	-3.162920
P	-1.099866	-0.319561	2.027294
C	-0.111328	0.887028	3.055153
C	0.423595	0.587483	4.327594
C	1.102844	1.568472	5.068264
C	1.261846	2.864169	4.547437
C	0.741977	3.170417	3.279348
C	0.064572	2.187867	2.539857
C	-0.465948	-2.001545	2.533644
Si	0.445872	-3.062818	1.204175
C	1.371895	-4.374627	2.268970
C	2.342717	-5.339802	1.558932
N	1.604618	-1.996046	0.346376
C	3.016713	-2.220704	0.408944
C	3.805329	-1.678215	1.470203
C	5.196617	-1.900101	1.466513
C	5.823321	-2.620575	0.444229
C	5.051342	-3.131441	-0.606647
C	3.656635	-2.947457	-0.647462
C	3.207638	-0.812722	2.580130
C	3.391958	-1.440111	3.978409
C	2.877550	-3.472106	-1.855588
C	3.136093	-2.598779	-3.104301
C	-0.850760	-3.841034	0.010053
C	-0.410044	-5.170705	-0.637965
C	-2.763952	-0.272694	2.884165
C	-2.904358	-0.118041	4.279801
C	-4.179744	-0.065959	4.865521
C	-5.331395	-0.155576	4.064185
C	-5.198056	-0.316079	2.675130
C	-3.923017	-0.383625	2.091332
C	3.172836	-4.953620	-2.168172
C	3.793003	0.617465	2.574086
C	-6.948723	-1.754569	-1.774671
C	-0.096390	-1.351786	-4.116247
C	-5.162764	3.374633	1.074758
C	-1.444348	4.373188	-4.189797
C	-2.230841	-4.012044	0.679787
C	0.375094	-5.173533	3.142259
H	-0.974625	-3.094744	-0.798560
H	-5.304206	0.492337	-3.254992
H	-4.742942	3.099795	-2.383195
H	-3.800896	-0.496218	1.008337
H	-2.013277	2.499153	-3.320118
H	-1.338353	-2.570761	2.898824
H	0.223476	-1.904389	3.390593
H	4.464390	-1.536828	4.232276
H	2.943215	-2.447228	4.044011
H	2.921605	-0.801404	4.748454
H	-0.371427	-5.991425	0.102890
H	0.580989	-5.110760	-1.119312

H	-1.139388	-5.469823	-1.417235
H	-0.501737	6.315485	0.427434
H	5.546103	-3.671009	-1.423702
H	2.126213	-0.721902	2.387996
H	1.982514	-3.748430	2.949874
H	-1.263601	-0.004621	-2.949711
H	-0.342893	2.422736	1.548769
H	6.911201	-2.761944	0.452744
H	0.322294	-0.417060	4.755010
H	5.806295	-1.474418	2.273259
H	4.040141	-0.621499	-1.299761
H	-5.112610	-4.340212	-1.873347
H	-2.019856	-0.015250	4.917990
H	-2.136238	5.202254	1.937115
H	1.804340	-3.388012	-1.614514
C	6.233229	0.920897	-1.396252
H	-3.603052	1.893609	1.183923
H	-5.431034	-0.815865	-0.562154
H	1.189196	2.470812	-0.224115
H	-0.114204	5.448587	-1.866091
H	-3.242133	-5.104077	-3.320019
H	-6.089231	-0.379660	2.039502
H	-1.379930	0.749153	-5.333683
H	-3.021655	0.493191	-4.680532
H	-2.296285	-0.727938	-5.769658
H	-2.946177	-4.473035	-0.027004
H	-2.655823	-3.038880	0.980794
H	-2.183087	-4.660453	1.576161
H	-1.549513	-3.482427	-4.138978
H	2.789317	-1.560934	-2.956287
H	2.602636	-3.005266	-3.984161
H	4.216143	-2.558987	-3.340337
H	-6.327631	-0.096320	4.521125
H	-4.273489	0.061856	5.951818
H	3.266463	1.244043	3.315892
H	3.686633	1.093104	1.587618
H	4.869012	0.608647	2.831562
H	1.517370	1.314609	6.052446
H	0.913890	3.359341	-2.928155
H	0.305943	2.220200	-4.170776
H	0.172696	1.812611	-2.430867
H	1.800249	3.627211	5.124074
H	0.871087	4.170809	2.851595
H	-6.128675	-3.778486	-0.029108
H	-6.679590	-2.372038	0.907505
H	-4.947283	-2.810155	0.899950
H	5.351146	3.461870	-0.887158
H	-0.156773	-2.000255	-5.011107
H	0.317762	-1.947829	-3.284734
H	0.622072	-0.543502	-4.342942
H	-2.395188	2.906824	3.164734
H	-4.094603	2.467448	3.478861
H	-3.653546	4.180741	3.241355
H	-2.446337	4.839491	-4.144517
H	-1.314497	3.927145	-5.193924
H	-0.691491	5.176188	-4.083114
H	2.840629	-5.992282	2.304821
H	3.132549	-4.805523	1.007416
H	1.816753	-6.002997	0.849755
H	4.208460	-5.097090	-2.529318

H	2.494327	-5.318507	-2.962415
H	3.033738	-5.589448	-1.277197
C	2.918458	4.564205	-0.321492
H	-0.270578	-5.824321	2.522651
H	-0.286417	-4.526199	3.746943
H	0.920461	-5.835205	3.845030
H	-6.845117	-1.076091	-2.639969
H	-7.790064	-1.393586	-1.153222
H	-7.216522	-2.754477	-2.165272
H	-5.320522	4.451567	1.276236
H	-5.868057	2.798958	1.703607
H	-5.416479	3.183583	0.017749
H	-1.955350	-1.237142	-0.072603
H	-0.643836	1.456209	-0.164017
F	4.007262	5.282275	0.040727
F	2.431586	5.142646	-1.460773
F	1.974473	4.749625	0.637146
F	6.282277	-0.151947	-2.223028
F	6.977725	1.905064	-1.962701
F	6.863990	0.564993	-0.245549

Table S69. Cartesian geometry of 3c-INT3 (17.5 kcal/mol) in Figure S145 in Angstrom [Å].

Atomtype	X Coordinates	Y Coordinates	Z Coordinates
C	-4.652597	-0.656963	0.583495
C	-3.986299	0.424694	-0.070174
C	-4.697396	1.214470	-1.031340
C	-6.046510	0.918231	-1.301272
C	-6.708476	-0.127680	-0.644575
C	-6.007445	-0.903235	0.286114
N	-2.605435	0.700994	0.178894
Si	-2.077227	1.716270	1.559885
C	-0.995761	0.643453	2.751867
P	0.276471	-0.478011	1.975537
Ni	0.882916	-0.009238	-0.058862
Ge	-1.213214	-0.050040	-0.933651
C	-2.098732	-1.636271	-1.840706
C	-1.288439	-2.793388	-1.949601
C	-1.767140	-3.974719	-2.538151
C	-3.072530	-4.022717	-3.056544
C	-3.883376	-2.877095	-2.987613
C	-3.403666	-1.699897	-2.387929
C	-4.016733	2.338132	-1.814594
C	-4.808982	3.661348	-1.780523
C	-3.931926	-1.600222	1.545919
C	-3.846218	-3.032991	0.974401
C	1.715170	-0.363375	3.171421
C	1.727615	-0.982420	4.439597
C	2.865883	-0.905993	5.259266
C	4.010592	-0.218335	4.820162
C	4.001759	0.408393	3.562886
C	2.859636	0.342618	2.749465
C	-0.349270	-2.204486	2.325175
C	-1.090522	-2.564987	3.472222
C	-1.440897	-3.904790	3.706016
C	-1.065108	-4.905234	2.792301
C	-0.347362	-4.553488	1.637941
C	0.003157	-3.214171	1.407784
C	2.587419	0.223951	-0.883181
N	3.255024	1.399263	-1.192865
C	4.484804	1.148694	-1.805903

C	4.611770	-0.207561	-1.894100
N	3.456836	-0.752929	-1.335671
C	2.703676	2.729086	-1.087634
C	3.231628	3.625673	-0.127373
C	2.722799	4.940722	-0.108952
C	1.709498	5.336768	-0.987018
C	1.195931	4.427253	-1.922640
C	1.691266	3.113622	-2.009992
C	3.107892	-2.147033	-1.445964
C	3.286753	-3.000858	-0.328131
C	2.883193	-4.341355	-0.469290
C	2.322698	-4.805372	-1.670626
C	2.164632	-3.938936	-2.759502
C	2.556083	-2.587579	-2.672311
C	4.339411	3.229565	0.848825
C	5.697249	3.819938	0.405120
C	1.201769	2.157190	-3.096265
C	-0.235836	2.435239	-3.560455
C	3.867952	-2.450882	0.970990
C	5.401422	-2.281570	0.879930
C	2.365966	-1.651091	-3.866362
C	3.209619	-2.113613	-5.073186
C	4.021975	3.649635	2.298899
C	2.172039	2.154808	-4.299203
C	3.505820	-3.259701	2.226809
C	0.880954	-1.486415	-4.242300
C	-1.051392	3.245882	0.987595
C	0.050341	3.633902	1.997019
C	-3.580684	2.190555	2.668547
C	-3.109977	2.918764	3.950078
C	-1.897766	4.486075	0.631156
C	-3.742414	1.911610	-3.273793
C	-4.581541	-1.632741	2.945504
C	-4.750786	2.962131	2.024534
H	-0.538176	2.907356	0.066723
H	5.139131	1.955007	-2.133568
H	5.397604	-0.832066	-2.317932
H	2.841769	0.809259	1.757932
H	2.728909	-0.649871	-3.581625
H	-0.446413	1.325160	3.424444
H	-1.682828	0.061463	3.391691
H	-5.606264	-2.047889	2.902845
H	-4.649876	-0.625851	3.395281
H	-3.990294	-2.275803	3.623523
H	-2.351439	4.941591	1.531711
H	-2.714747	4.263682	-0.075688
H	-1.255813	5.259846	0.163691
H	2.002830	-5.852317	-1.754369
H	-6.588943	1.515950	-2.044806
H	-2.898573	-1.235055	1.660588
H	-3.976509	1.201079	2.974184
H	1.195226	1.145722	-2.654148
H	0.567083	-2.930898	0.511832
H	-7.762341	-0.342221	-0.864696
H	-1.406078	-1.804263	4.196367
H	-6.518820	-1.736483	0.785000
H	-4.074477	-0.838435	-2.334633
H	3.117830	5.660506	0.616971
H	0.858452	-1.550330	4.788508
H	2.986640	-5.029059	0.376550

H	-3.038719	2.521187	-1.338100
H	-4.904162	-2.899308	-3.392210
H	3.425356	-1.447660	1.096731
H	4.429915	2.128174	0.829821
H	-0.258388	-2.771851	-1.572939
H	1.715174	-4.308997	-3.688979
H	1.313191	6.359211	-0.942662
H	4.891420	0.941313	3.206216
H	1.833996	1.428889	-5.061897
H	3.196503	1.875317	-3.997106
H	2.213253	3.155710	-4.769957
H	0.599059	4.526492	1.641324
H	0.792896	2.825349	2.112997
H	-0.363120	3.872936	2.996318
H	0.401981	4.752556	-2.602754
H	-3.078541	1.030591	-3.322333
H	-3.258621	2.731514	-3.837289
H	-4.686184	1.649106	-3.788713
H	4.905900	-0.175480	5.453847
H	2.863065	-1.400510	6.239488
H	-3.264329	-3.678052	1.655439
H	-3.357530	-3.044261	-0.011854
H	-4.855670	-3.472705	0.861401
H	-2.019158	-4.165993	4.601732
H	0.411489	-2.450564	-4.508617
H	0.779951	-0.805715	-5.108236
H	0.313520	-1.061884	-3.399192
H	-1.346707	-5.950389	2.973959
H	-0.059580	-5.318303	0.905406
H	3.980765	4.749309	2.403080
H	4.809863	3.287547	2.983599
H	3.057631	3.235084	2.635765
H	-3.453962	-4.944636	-3.515578
H	-0.312271	3.363589	-4.158340
H	-0.922237	2.519558	-2.700022
H	-0.592219	1.604795	-4.195706
H	2.419781	-3.433187	2.301984
H	3.819106	-2.699116	3.124958
H	4.018906	-4.240684	2.244531
H	4.279398	-2.201078	-4.805705
H	3.118701	-1.390843	-5.906043
H	2.872707	-3.099782	-5.444685
H	-5.564838	3.102681	2.764750
H	-5.177870	2.428598	1.160562
H	-4.443029	3.968320	1.688720
H	-5.758981	3.583967	-2.342129
H	-4.217549	4.473182	-2.244814
H	-5.051377	3.959105	-0.745797
H	-1.114078	-4.856573	-2.591809
H	-2.703569	3.921232	3.716541
H	-2.329203	2.363436	4.501651
H	-3.960655	3.069719	4.645123
H	5.970111	3.499908	-0.616329
H	6.504595	3.502221	1.092019
H	5.659170	4.925737	0.410522
H	5.893705	-3.264886	0.752724
H	5.786123	-1.821282	1.809553
H	5.698794	-1.637466	0.034851
H	0.993693	1.371908	0.502670
H	0.823772	-1.383522	-0.646843

Table S70. Cartesian geometry of 3d-INT3 (17.8 kcal/mol) in Figure S145 in Angstrom [Å].

Atomtype	X Coordinates	Y Coordinates	Z Coordinates
C	-2.178088	3.226135	-2.201141
C	-2.834920	2.637725	-1.094406
C	-2.973444	3.289200	0.157654
C	-2.419315	4.576606	0.279824
C	-1.753550	5.182801	-0.798175
C	-1.637889	4.516155	-2.024236
N	-3.338707	1.296911	-1.254438
C	-2.608323	0.159944	-0.955502
N	-3.381038	-0.855165	-1.499491
C	-4.539046	-0.357319	-2.101285
C	-4.512205	0.998508	-1.945229
Ni	-0.938304	0.041301	-0.043109
Ge	1.190460	0.003094	-0.833839
C	2.280877	1.612748	-1.402028
C	1.605959	2.854648	-1.322597
C	2.239286	4.061827	-1.654127
C	3.575563	4.080614	-2.102231
C	4.243098	2.843519	-2.222173
C	3.614221	1.636776	-1.879827
C	-2.983321	-2.237227	-1.621744
C	-3.654785	-3.223021	-0.859110
C	-3.293817	-4.570430	-1.065934
C	-2.285222	-4.918638	-1.969384
C	-1.627403	-3.922818	-2.705575
C	-1.971729	-2.566137	-2.566215
C	-4.761184	-2.880564	0.138660
C	-4.565103	-3.585567	1.496780
C	-1.323672	-1.494547	-3.441319
C	-2.219013	-1.167069	-4.657696
C	-3.670593	2.581447	1.315796
C	-3.292896	3.108651	2.709176
C	-2.030188	2.505977	-3.542328
C	-0.554597	2.240696	-3.897956
P	-0.387007	0.060178	2.059761
C	0.405013	1.610042	2.742603
C	1.115993	1.668948	3.961787
C	1.595637	2.894423	4.452293
C	1.382968	4.080022	3.726865
C	0.699307	4.027838	2.501637
C	0.218814	2.802195	2.015175
C	0.716817	-1.323682	2.648351
Si	1.738147	-2.275628	1.308788
C	3.125374	-3.110917	2.354406
C	4.238982	-3.878329	1.612807
N	2.439485	-1.092952	0.157191
C	3.852139	-0.932315	0.004570
C	4.595018	-0.069392	0.867773
C	5.980997	0.074731	0.660606
C	6.642156	-0.590894	-0.378113
C	5.907686	-1.418713	-1.237529
C	4.523415	-1.606805	-1.066796
C	3.930257	0.756431	1.968319
C	4.510394	0.460474	3.367484
C	3.768642	-2.484857	-2.066007
C	3.616617	-1.772408	-3.428239
C	0.586336	-3.549192	0.430745
C	1.313419	-4.790735	-0.127279

C	-1.887429	-0.101270	3.172220
C	-1.896141	0.272716	4.532971
C	-3.076896	0.185837	5.289118
C	-4.267441	-0.267261	4.694783
C	-4.263735	-0.651061	3.343386
C	-3.080681	-0.576069	2.591617
C	4.413027	-3.873832	-2.253702
C	4.022342	2.270059	1.672519
C	-6.150856	-3.220774	-0.446184
C	0.100301	-1.847996	-3.896352
C	-5.206950	2.594748	1.149345
C	-2.755939	3.274853	-4.666771
C	-0.599135	-3.987897	1.316660
C	2.515974	-4.003450	3.461732
H	0.158220	-2.991917	-0.424928
H	-5.257676	-1.010408	-2.593979
H	-5.200123	1.776131	-2.275789
H	-3.064060	-0.852989	1.531289
H	-2.513854	1.518735	-3.460106
H	0.063295	-2.050471	3.162015
H	1.427527	-0.952465	3.408182
H	5.572979	0.761834	3.433213
H	4.453562	-0.613137	3.622206
H	3.955257	1.029104	4.136464
H	1.672744	-5.451371	0.684375
H	2.182077	-4.536542	-0.757685
H	0.616505	-5.388858	-0.748523
H	-1.317269	6.182970	-0.676674
H	6.420616	-1.926836	-2.064027
H	2.860212	0.493414	1.982681
H	3.606779	-2.244940	2.851891
H	-1.234729	-0.585323	-2.821840
H	-0.319551	2.752474	1.061972
H	7.722159	-0.458581	-0.523862
H	1.306915	0.758588	4.542721
H	6.551447	0.740606	1.320862
H	4.191734	0.713385	-1.973260
H	-3.801922	-5.357135	-0.496660
H	-0.988175	0.658549	5.008617
H	-2.487840	5.106121	1.235851
H	2.753723	-2.638560	-1.661952
H	5.282379	2.823800	-2.579629
H	-3.334942	1.530967	1.268453
H	-4.730088	-1.790323	0.317845
H	0.557638	2.884403	-1.001067
H	-1.104840	4.993575	-2.855331
H	-2.005336	-5.971667	-2.100642
H	-5.187911	-0.999252	2.866496
H	-1.763380	-0.360521	-5.261743
H	-3.223360	-0.830239	-4.346625
H	-2.339710	-2.056766	-5.305006
H	-1.220685	-4.732685	0.784333
H	-1.256555	-3.134980	1.559516
H	-0.263791	-4.450637	2.265330
H	-0.838719	-4.211487	-3.408030
H	3.053180	-0.827610	-3.332700
H	3.078375	-2.416196	-4.149116
H	4.608194	-1.530005	-3.855770
H	-5.193673	-0.316594	5.281846
H	-3.069235	0.489352	6.344218

H	3.481003	2.840647	2.446869
H	3.583476	2.516045	0.693467
H	5.076833	2.606744	1.670147
H	2.147108	2.920617	5.401051
H	0.030696	3.175712	-3.962174
H	-0.484503	1.725005	-4.874003
H	-0.080443	1.603900	-3.134519
H	1.765162	5.035230	4.109206
H	0.539637	4.939934	1.912606
H	-4.648078	-4.683571	1.399825
H	-5.342971	-3.262484	2.211775
H	-3.580039	-3.351516	1.932916
C	4.283564	5.379339	-2.421074
H	0.111876	-2.658235	-4.650355
H	0.724962	-2.160890	-3.041621
H	0.577249	-0.964130	-4.356178
H	-2.200224	3.143663	2.850696
H	-3.709634	2.435929	3.479199
H	-3.701884	4.121800	2.888718
H	-3.822083	3.436168	-4.419891
H	-2.701870	2.709806	-5.616567
H	-2.295393	4.266179	-4.837859
H	4.994686	-4.243881	2.337859
H	4.763735	-3.246956	0.878231
H	3.844269	-4.764190	1.084318
H	5.393464	-3.804885	-2.761524
H	3.763297	-4.515537	-2.878620
H	4.568836	-4.379551	-1.285188
H	1.680866	5.004735	-1.564698
H	2.016638	-4.892514	3.031763
H	1.772589	-3.472992	4.084899
H	3.309698	-4.376048	4.140511
H	-6.334732	-2.694858	-1.400049
H	-6.952727	-2.938306	0.262205
H	-6.233810	-4.306776	-0.641118
H	-5.593068	3.631371	1.191854
H	-5.679524	2.017265	1.966177
H	-5.525403	2.149212	0.191537
H	-1.226023	-1.397298	0.247342
H	-0.706096	1.484381	-0.358844
H	4.937576	5.278271	-3.307540
H	3.568017	6.200536	-2.609604
H	4.931289	5.692377	-1.577065

Table S71. Cartesian geometry of 3e-INT3 (17.7 kcal/mol) in Figure S145 in Angstrom [Å].

Atomtype	X Coordinates	Y Coordinates	Z Coordinates
C	-1.679994	3.657597	-1.880470
C	-2.462781	3.056422	-0.866420
C	-2.566833	3.585684	0.445332
C	-1.845141	4.760350	0.725829
C	-1.053979	5.375956	-0.258175
C	-0.975892	4.832313	-1.546285
N	-3.140239	1.826027	-1.189469
C	-2.591937	0.570385	-0.992264
N	-3.470871	-0.260033	-1.672306
C	-4.515952	0.460193	-2.255668
C	-4.307430	1.774034	-1.950085
Ni	-1.004338	0.117376	-0.040146
Ge	1.138580	-0.131453	-0.749450
C	2.460415	1.354145	-1.096874

C	1.950606	2.668298	-0.924581
C	2.746455	3.803106	-1.096586
C	4.101226	3.666808	-1.474566
C	4.626925	2.374862	-1.689289
C	3.811283	1.245361	-1.499616
C	-3.262968	-1.663246	-1.937959
C	-4.102749	-2.619431	-1.317722
C	-3.924240	-3.973834	-1.667260
C	-2.930580	-4.362810	-2.570706
C	-2.103545	-3.397809	-3.163339
C	-2.261152	-2.029242	-2.879122
C	-5.197308	-2.232000	-0.323512
C	-5.165422	-3.100266	0.951277
C	-1.426928	-0.972929	-3.601800
C	-2.204270	-0.392956	-4.805068
C	-3.405270	2.864816	1.496899
C	-3.030342	3.193640	2.950627
C	-1.569864	3.067151	-3.287165
C	-0.128622	2.639864	-3.626092
P	-0.566404	-0.174492	2.070066
C	0.402248	1.157171	2.955521
C	1.043634	0.978482	4.201328
C	1.663328	2.059747	4.848944
C	1.663314	3.334973	4.256410
C	1.051883	3.516498	3.005617
C	0.430281	2.434964	2.362446
C	0.298567	-1.761846	2.529321
Si	1.253091	-2.691317	1.125529
C	2.454663	-3.826026	2.118169
C	3.492829	-4.654380	1.334111
N	2.171930	-1.502309	0.147065
C	3.598545	-1.529778	0.065279
C	4.407945	-0.886245	1.051551
C	5.809390	-0.917619	0.911563
C	6.425794	-1.547046	-0.176160
C	5.629752	-2.158868	-1.153682
C	4.225835	-2.166582	-1.054880
C	3.807357	-0.105742	2.219715
C	4.279965	-0.629515	3.592268
C	3.410027	-2.808542	-2.177661
C	3.427955	-1.930642	-3.448498
C	-0.010010	-3.685849	0.059587
C	0.571744	-4.948782	-0.609838
C	-2.129915	-0.233544	3.103021
C	-2.159677	-0.007548	4.495723
C	-3.380260	0.000444	5.191270
C	-4.588849	-0.208575	4.504627
C	-4.566053	-0.444381	3.119840
C	-3.345247	-0.464413	2.427477
C	3.866174	-4.244791	-2.507449
C	4.108821	1.404813	2.101885
C	-6.591319	-2.304712	-0.987077
C	-0.042842	-1.473819	-4.041186
C	-4.917476	3.099438	1.281187
C	-2.135927	4.041253	-4.342118
C	-1.291588	-4.047680	0.840064
C	1.668998	-4.742946	3.085592
H	-0.310242	-2.982957	-0.741562
H	-5.293175	-0.027038	-2.842408
H	-4.862330	2.673166	-2.216259

H	-3.310184	-0.625730	1.343982
H	-2.183959	2.152599	-3.327460
H	-0.478926	-2.440762	2.921577
H	1.007321	-1.588986	3.358978
H	5.367283	-0.476120	3.728367
H	4.078952	-1.708895	3.716406
H	3.763483	-0.084700	4.404250
H	0.790371	-5.739324	0.132854
H	1.501714	-4.753072	-1.169642
H	-0.163644	-5.369696	-1.325005
H	-0.489156	6.285138	-0.013816
H	6.109754	-2.637979	-2.016519
H	2.712697	-0.222268	2.171507
H	3.020443	-3.098410	2.734461
H	-1.246417	-0.157540	-2.879985
H	-0.053724	2.567999	1.388116
H	7.519879	-1.554541	-0.266805
H	1.069152	-0.007497	4.680921
H	6.430744	-0.419992	1.667266
H	4.270309	0.265752	-1.656597
H	-4.565063	-4.736428	-1.210067
H	-1.233633	0.189636	5.046137
H	-1.882463	5.190619	1.732098
H	2.364331	-2.860885	-1.830296
H	5.669628	2.228855	-1.989588
H	-3.205698	1.789169	1.349128
H	-5.023608	-1.183208	-0.020967
H	0.897873	2.810606	-0.651004
H	-0.344111	5.313457	-2.302666
H	-2.794702	-5.424362	-2.813826
H	-5.502897	-0.601252	2.571602
H	-1.611063	0.399849	-5.297470
H	-3.167029	0.049073	-4.494153
H	-2.413266	-1.182220	-5.552439
H	-1.975670	-4.638422	0.201844
H	-1.841956	-3.142070	1.149159
H	-1.075516	-4.650243	1.743839
H	-1.328784	-3.718399	-3.867500
H	3.000060	-0.930754	-3.257599
H	2.840533	-2.401907	-4.258828
H	4.463959	-1.788949	-3.811228
H	-5.543256	-0.183053	5.046349
H	-3.387141	0.187268	6.273165
H	3.621467	1.954269	2.925568
H	3.739880	1.814725	1.149138
H	5.198111	1.593638	2.158252
H	2.157241	1.901990	5.816496
H	0.578919	3.486631	-3.569135
H	-0.085211	2.225843	-4.650920
H	0.221906	1.866013	-2.924866
H	2.154655	4.176533	4.761417
H	1.059107	4.499897	2.518598
H	-5.394928	-4.158174	0.727391
H	-5.923786	-2.748327	1.673574
H	-4.179153	-3.058060	1.442049
O	4.805723	4.827559	-1.611826
H	-0.102316	-2.192513	-4.880885
H	0.487742	-1.960784	-3.204557
H	0.573361	-0.622155	-4.380435
H	-1.950410	3.068176	3.132929

H	-3.567338	2.508391	3.629811
H	-3.313254	4.229110	3.222314
H	-3.181183	4.320208	-4.111346
H	-2.115095	3.576516	-5.346094
H	-1.540584	4.972623	-4.389266
H	4.149318	-5.204787	2.038790
H	4.139667	-4.024726	0.702684
H	3.010728	-5.408178	0.686737
H	4.871464	-4.259264	-2.969117
H	3.167126	-4.712543	-3.226449
H	3.901332	-4.873720	-1.601143
H	2.340461	4.810802	-0.945356
H	1.078149	-5.497776	2.532626
H	0.970814	-4.186459	3.737630
H	2.365407	-5.298507	3.745858
H	-6.654613	-1.658416	-1.880691
H	-7.379031	-1.988005	-0.277270
H	-6.814999	-3.340299	-1.306207
H	-5.168181	4.168616	1.420902
H	-5.498414	2.510447	2.015766
H	-5.245611	2.799213	0.271557
H	-1.496223	-1.291365	0.061853
H	-0.565517	1.541368	-0.167418
C	6.175476	4.757446	-1.971760
H	6.543827	5.796930	-2.012106
H	6.769818	4.190779	-1.224199
H	6.318199	4.284395	-2.966400

Table S72. Cartesian geometry of 3f-INT3 (17.7 kcal/mol) in Figure S145 in Angstrom [Å].

Atomtype	X Coordinates	Y Coordinates	Z Coordinates
C	-1.143923	3.902029	-1.669982
C	-2.016347	3.357291	-0.698003
C	-2.054409	3.809851	0.646341
C	-1.175170	4.848766	1.001868
C	-0.297708	5.409401	0.058590
C	-0.285041	4.942877	-1.261387
N	-2.865546	2.265655	-1.102130
C	-2.524593	0.930219	-0.964844
N	-3.502444	0.285783	-1.709840
C	-4.406272	1.191016	-2.271149
C	-4.004856	2.437649	-1.887552
Ni	-1.053191	0.187009	-0.014885
Ge	1.045374	-0.319694	-0.718583
C	2.559647	0.972476	-0.972269
C	2.229037	2.330981	-0.743548
C	3.167273	3.363525	-0.827005
C	4.522612	3.091499	-1.175353
C	4.856110	1.732245	-1.449246
C	3.901861	0.714395	-1.347123
C	-3.504194	-1.114445	-2.057977
C	-4.492451	-1.965906	-1.508378
C	-4.516411	-3.307787	-1.940967
C	-3.575596	-3.789427	-2.856354
C	-2.597814	-2.929384	-3.376769
C	-2.550038	-1.572794	-3.008065
C	-5.533096	-1.475121	-0.502058
C	-5.659128	-2.414164	0.715362
C	-1.547784	-0.615632	-3.650768
C	-2.197039	0.143644	-4.829911
C	-2.987073	3.139619	1.651133

C	-2.601131	3.347749	3.124077
C	-1.104230	3.391414	-3.111462
C	0.274220	2.817163	-3.492680
P	-0.705401	-0.307339	2.074172
C	0.442474	0.796010	3.056091
C	1.011874	0.442644	4.299706
C	1.773367	1.370837	5.028458
C	1.991197	2.663506	4.519506
C	1.457015	3.013322	3.269161
C	0.692820	2.084927	2.545246
C	-0.095038	-2.035236	2.423981
Si	0.750729	-2.992047	0.967676
C	1.759261	-4.350149	1.893107
C	2.684222	-5.265257	1.064663
N	1.850768	-1.888748	0.080555
C	3.259679	-2.118446	0.009926
C	4.133763	-1.676176	1.050320
C	5.518208	-1.902779	0.922309
C	6.059121	-2.533666	-0.204021
C	5.202887	-2.947590	-1.232973
C	3.811141	-2.756241	-1.148652
C	3.629972	-0.901664	2.267002
C	4.011671	-1.571779	3.603662
C	2.934559	-3.188485	-2.324272
C	3.111950	-2.235708	-3.526999
C	-0.616969	-3.723016	-0.178974
C	-0.203051	-5.006672	-0.928790
C	-2.276463	-0.196197	3.093184
C	-2.300191	-0.062908	4.497849
C	-3.518942	0.092018	5.179629
C	-4.730667	0.122775	4.467896
C	-4.715892	-0.020797	3.070353
C	-3.498891	-0.186116	2.391104
C	3.180511	-4.650214	-2.751563
C	4.133198	0.558738	2.247632
C	-6.908543	-1.288691	-1.181748
C	-0.244137	-1.295318	-4.096151
C	-4.460652	3.552766	1.437311
C	-1.551433	4.492243	-4.096900
C	-1.954605	-3.951155	0.556367
C	0.834338	-5.206607	2.790014
H	-0.795815	-2.931209	-0.931930
H	-5.233474	0.863331	-2.898988
H	-4.407078	3.425385	-2.110689
H	-3.466429	-0.274996	1.299002
H	-1.823276	2.561143	-3.203675
H	-0.975779	-2.618372	2.745699
H	0.604918	-2.032542	3.278969
H	5.107296	-1.566520	3.758570
H	3.674538	-2.622997	3.652387
H	3.554753	-1.021127	4.446981
H	-0.116112	-5.869579	-0.241579
H	0.758790	-4.906948	-1.459331
H	-0.972586	-5.268480	-1.683037
H	0.386441	6.213204	0.361096
H	5.625593	-3.426829	-2.125249
H	2.530193	-0.865922	2.209004
H	2.411027	-3.751910	2.561514
H	-1.265208	0.120028	-2.877377
H	0.268177	2.349703	1.570081

H	7.142125	-2.694887	-0.284274
H	0.868784	-0.562509	4.714563
H	6.189540	-1.558568	1.719577
H	4.236964	-0.306207	-1.551059
H	-5.275452	-3.989080	-1.539718
H	-1.366893	-0.052657	5.070672
H	-1.158799	5.213678	2.034174
H	1.885132	-3.108501	-1.993479
H	5.875720	1.454364	-1.732798
H	-2.903482	2.057605	1.446312
H	-5.205208	-0.486744	-0.131760
H	1.196071	2.600723	-0.489265
H	0.415328	5.375877	-1.985898
H	-3.598847	-4.842454	-3.164738
H	-5.653915	0.008824	2.502694
H	-1.479312	0.865915	-5.261424
H	-3.091728	0.705791	-4.509712
H	-2.500579	-0.560338	-5.628371
H	-2.699437	-4.395716	-0.130645
H	-2.379919	-2.999086	0.918692
H	-1.846302	-4.636718	1.419510
H	-1.866079	-3.322248	-4.090349
H	2.836599	-1.198589	-3.266611
H	2.477897	-2.553580	-4.375945
H	4.165108	-2.227966	-3.867207
H	-5.680442	0.262533	5.000305
H	-3.519243	0.204743	6.271804
H	3.728430	1.111400	3.112655
H	3.820292	1.078078	1.328642
H	5.238339	0.593073	2.301840
H	2.206579	1.078792	5.993907
H	1.080575	3.564838	-3.383685
H	0.264831	2.476300	-4.545048
H	0.531372	1.957888	-2.852905
H	2.591942	3.384819	5.088399
H	1.635979	4.009460	2.844656
H	-6.046283	-3.407592	0.423362
H	-6.365702	-1.992744	1.452838
H	-4.686799	-2.556428	1.215257
N	5.469869	4.101795	-1.249093
H	-0.389311	-1.945438	-4.980188
H	0.183781	-1.907076	-3.282954
H	0.503088	-0.529568	-4.370465
H	-1.547103	3.086287	3.313321
H	-3.226931	2.696662	3.759598
H	-2.765820	4.394345	3.446341
H	-2.556139	4.877261	-3.840104
H	-1.586200	4.095444	-5.129260
H	-0.849762	5.347806	-4.087062
H	3.251186	-5.940696	1.737685
H	3.418264	-4.693932	0.474477
H	2.112260	-5.907272	0.371264
H	4.182751	-4.781948	-3.201224
H	2.436075	-4.957658	-3.510373
H	3.102670	-5.339006	-1.892496
H	2.830665	4.383965	-0.619290
H	0.151111	-5.830243	2.182556
H	0.211871	-4.599808	3.473132
H	1.432694	-5.899230	3.416132
H	-6.853890	-0.584057	-2.030783

H	-7.651817	-0.899925	-0.459873
H	-7.282767	-2.254490	-1.571089
H	-4.593565	4.633645	1.636625
H	-5.111621	2.991367	2.133579
H	-4.808122	3.347597	0.410789
H	-1.738487	-1.143129	-0.030687
H	-0.419606	1.540194	-0.017342
C	6.847949	3.783390	-1.581945
C	5.092426	5.470915	-0.947160
H	7.453374	4.704484	-1.581447
H	7.299830	3.077972	-0.852271
H	6.936099	3.320921	-2.587857
H	5.969517	6.130712	-1.048410
H	4.305256	5.846656	-1.635482
H	4.703650	5.577793	0.088568

Table S73. Cartesian geometry of 3g-INT3 (18.3 kcal/mol) in Figure S145 in Angstrom [Å].

Atomtype	X Coordinates	Y Coordinates	Z Coordinates
C	3.014260	1.134005	3.397121
C	3.359631	1.237204	2.029128
C	3.505364	2.479502	1.358535
C	3.299843	3.646554	2.116028
C	2.962971	3.576428	3.478681
C	2.822623	2.335072	4.110630
N	3.532723	0.010791	1.294006
C	2.527747	-0.615189	0.573345
N	3.088636	-1.853478	0.282234
C	4.381522	-1.970360	0.799070
C	4.660567	-0.796157	1.434257
Ni	0.839597	0.099909	0.086413
Ge	-1.179205	-0.043403	1.155031
N	-1.775174	0.989848	2.624732
C	2.407500	-2.988476	-0.291377
C	2.795187	-3.457431	-1.570068
C	2.197909	-4.644288	-2.042546
C	1.228120	-5.315552	-1.291587
C	0.846252	-4.820571	-0.036390
C	1.439488	-3.664242	0.501244
C	3.842363	-2.746069	-2.427291
C	3.351838	-2.514863	-3.871939
C	1.111107	-3.197162	1.917308
C	2.141923	-3.761744	2.920844
C	3.812778	2.507192	-0.136099
C	3.462737	3.829457	-0.836786
C	2.849787	-0.217054	4.097249
C	1.432300	-0.423873	4.667254
P	0.086786	1.311945	-1.551969
C	-0.478141	3.054508	-1.178145
C	-1.251912	3.834274	-2.066444
C	-1.526523	5.182736	-1.784989
C	-1.044539	5.770665	-0.602265
C	-0.305730	4.993778	0.304738
C	-0.030254	3.646787	0.019895
C	-1.310762	0.571513	-2.540393
Si	-2.400211	-0.786983	-1.684033
C	-4.030401	-0.714333	-2.715078
C	-5.214262	-1.604341	-2.281802
N	-2.730615	-0.346433	0.027507
C	-4.070298	-0.137013	0.478780
C	-4.728717	1.113662	0.269019

C	-6.034747	1.290173	0.765546
C	-6.699687	0.273356	1.461818
C	-6.052174	-0.951574	1.668020
C	-4.751115	-1.181430	1.183487
C	-4.029945	2.294076	-0.402483
C	-4.885162	2.975691	-1.489764
C	-4.082370	-2.525799	1.463234
C	-3.648944	-2.633309	2.941609
C	-1.501068	-2.486790	-1.834438
C	-2.440924	-3.710294	-1.856624
C	1.415819	1.629875	-2.836739
C	1.414435	2.715527	-3.738191
C	2.496073	2.919001	-4.612020
C	3.596912	2.045540	-4.593934
C	3.601770	0.957181	-3.705198
C	2.517910	0.749637	-2.838519
C	-4.965368	-3.726154	1.064911
C	-3.575410	3.326453	0.651409
C	5.175932	-3.526921	-2.432483
C	-0.320860	-3.529893	2.361730
C	5.281786	2.134820	-0.436707
C	3.926613	-0.403829	5.187467
C	-0.532856	-2.543270	-3.034915
C	-3.734824	-0.935662	-4.218154
H	-0.880807	-2.551876	-0.919830
H	4.967589	-2.880001	0.678357
H	5.540607	-0.464811	1.984492
H	2.520403	-0.076785	-2.117802
H	3.000751	-1.013253	3.350728
H	-0.844942	0.110048	-3.428829
H	-1.980872	1.362743	-2.922664
H	-5.748823	3.512866	-1.054356
H	-5.279558	2.247557	-2.222161
H	-4.276244	3.722707	-2.032689
H	-3.006306	-3.771697	-2.805705
H	-3.173995	-3.707954	-1.032568
H	-1.847801	-4.643130	-1.771159
H	2.802558	4.501020	4.048509
H	-6.567979	-1.747306	2.220348
H	-3.115986	1.913323	-0.885280
H	-4.352178	0.339653	-2.590856
H	1.188353	-2.096070	1.920460
H	0.549324	3.032675	0.721088
H	-7.715376	0.435908	1.845231
H	-1.645738	3.399038	-2.993010
H	-6.536772	2.254554	0.617044
H	2.486674	-5.037323	-3.024228
H	0.583405	3.428688	-3.748115
H	3.387493	4.626435	1.634695
H	-3.167621	-2.564309	0.847754
H	3.161699	1.728952	-0.573405
H	4.034134	-1.756160	-1.974839
H	2.547356	2.292059	5.171759
H	0.760941	-6.226680	-1.686529
H	4.459307	0.274111	-3.674292
H	1.929108	-3.390863	3.940410
H	3.171600	-3.461507	2.658105
H	2.101167	-4.867672	2.940440
H	-0.074692	-3.546605	-3.113014
H	0.293003	-1.820927	-2.914292

H	-1.042996	-2.335067	-3.995577
H	0.082952	-5.354239	0.539567
H	-2.918330	-1.848139	3.204735
H	-3.180122	-3.614669	3.142745
H	-4.521455	-2.523197	3.613390
H	4.448887	2.216649	-5.264658
H	2.484001	3.774795	-5.299766
H	-3.122528	4.204985	0.162446
H	-2.822880	2.884389	1.323051
H	-4.435801	3.668685	1.257632
H	-2.125886	5.773629	-2.489693
H	1.165967	0.360468	5.399071
H	1.372208	-1.399321	5.185204
H	0.677308	-0.416120	3.863306
H	-1.261620	6.823922	-0.382675
H	0.060034	5.432553	1.242131
H	3.188992	-3.470844	-4.402535
H	4.105092	-1.946028	-4.446244
H	2.407662	-1.946040	-3.889656
H	-0.466380	-4.612475	2.540055
H	-1.058035	-3.202212	1.608541
H	-0.550215	-3.008244	3.307710
H	2.424879	4.140278	-0.632344
H	3.564296	3.700941	-1.928900
H	4.140931	4.648779	-0.528357
H	4.943966	-0.291768	4.768434
H	3.848192	-1.409916	5.641408
H	3.811400	0.342929	5.995819
H	-6.093521	-1.404768	-2.927976
H	-5.520954	-1.420234	-1.240240
H	-4.976935	-2.678103	-2.383171
H	-5.847592	-3.823309	1.725347
H	-4.389688	-4.667908	1.143034
H	-5.329832	-3.628125	0.027053
H	-3.413754	-1.976236	-4.414686
H	-2.947797	-0.265185	-4.609324
H	-4.647965	-0.760620	-4.822400
H	5.571464	-3.671202	-1.411213
H	5.940925	-2.988853	-3.023927
H	5.038434	-4.528126	-2.882966
H	5.968765	2.894493	-0.016345
H	5.440197	2.094036	-1.530580
H	5.559629	1.152438	-0.019358
H	0.662891	-1.045443	-0.862028
H	1.109669	1.260875	0.982609
C	-3.047967	1.032347	3.330993
C	-0.781798	1.850856	3.254205
H	-1.057257	2.924702	3.137970
H	-0.691110	1.649909	4.343989
H	0.213359	1.712045	2.795695
H	-3.460278	2.065070	3.360874
H	-3.804136	0.389815	2.861905
H	-2.922627	0.702183	4.388086

Table S74. Cartesian geometry of 3a-TS3 (24.6 kcal/mol) in Figure S145 in Angstrom [Å].

Atomtype	X Coordinates	Y Coordinates	Z Coordinates
C	-2.062655	3.593896	-1.604864
C	-2.777885	2.855790	-0.629552
C	-2.975843	3.321574	0.693383
C	-2.413358	4.566863	1.033652

C	-1.682481	5.310509	0.094549
C	-1.515340	4.831159	-1.212022
N	-3.296825	1.567859	-1.019323
C	-2.554733	0.396325	-0.964144
N	-3.324256	-0.485069	-1.715695
C	-4.479052	0.127841	-2.213339
C	-4.460815	1.418491	-1.773710
Ni	-0.843749	0.188824	-0.168590
Ge	1.271495	0.120829	-0.558838
C	2.510735	1.583735	-1.182192
C	1.971485	2.882934	-1.030437
C	2.722832	4.027853	-1.314592
C	4.056878	3.898454	-1.770424
C	4.607863	2.605262	-1.937184
C	3.842743	1.468863	-1.645259
C	4.841697	5.067033	-2.057857
N	5.478146	6.022377	-2.293180
C	-2.937632	-1.815937	-2.112948
C	-3.673197	-2.926610	-1.632138
C	-3.316661	-4.205940	-2.108187
C	-2.248569	-4.376811	-2.993974
C	-1.531733	-3.261948	-3.452369
C	-1.872796	-1.958803	-3.045996
C	-4.848226	-2.787883	-0.662936
C	-4.719526	-3.724986	0.556361
C	-1.193663	-0.739351	-3.668178
C	-2.036948	-0.206716	-4.848289
C	-3.764399	2.481284	1.692962
C	-3.424076	2.754413	3.167302
C	-1.901026	3.102568	-3.044179
C	-0.425483	2.947503	-3.456756
P	-0.638654	-0.286771	1.982686
C	0.141066	1.121490	2.947364
C	0.781844	0.984200	4.198321
C	1.298624	2.106471	4.867848
C	1.184551	3.386410	4.297917
C	0.558878	3.533167	3.049091
C	0.049629	2.409918	2.379660
C	0.400298	-1.760108	2.482891
Si	1.519635	-2.548173	1.131200
C	2.797820	-3.532058	2.187527
C	3.931171	-4.295076	1.473227
N	2.347453	-1.243169	0.209211
C	3.775546	-1.172058	0.145910
C	4.511601	-0.444961	1.129702
C	5.912144	-0.356804	1.002401
C	6.590003	-0.965260	-0.060715
C	5.863756	-1.684228	-1.020270
C	4.463811	-1.806177	-0.937672
C	3.826503	0.285852	2.284897
C	4.358640	-0.151587	3.665745
C	3.717018	-2.565391	-2.037239
C	3.670955	-1.750102	-3.348991
C	0.433254	-3.642534	-0.029985
C	1.077276	-4.974190	-0.467316
C	-2.210584	-0.580354	2.956579
C	-2.309225	-0.463832	4.359423
C	-3.538241	-0.671404	5.006864
C	-4.687138	-0.991188	4.261360
C	-4.595401	-1.116746	2.865287

C	-3.363543	-0.918470	2.220998
C	4.307838	-3.965164	-2.307582
C	3.946262	1.818566	2.133620
C	-6.189050	-3.034857	-1.391144
C	0.257517	-0.994687	-4.099310
C	-5.287196	2.624698	1.471407
C	-2.668814	4.020613	-4.019763
C	-0.974057	-3.883121	0.550731
C	2.070837	-4.476473	3.176376
H	0.294102	-3.023184	-0.937717
H	-5.191443	-0.410668	-2.836330
H	-5.146370	2.247414	-1.946398
H	-3.281318	-0.991580	1.129803
H	-2.352080	2.100202	-3.118614
H	-0.298214	-2.537606	2.837880
H	1.045579	-1.510179	3.343902
H	5.416438	0.141706	3.803473
H	4.295393	-1.246104	3.804875
H	3.770872	0.334958	4.466409
H	1.208832	-5.667974	0.384355
H	2.064551	-4.835120	-0.939710
H	0.426206	-5.485071	-1.205215
H	-1.243606	6.274406	0.383939
H	6.396092	-2.155689	-1.855896
H	2.752291	0.039309	2.250996
H	3.274225	-2.727972	2.784968
H	-1.152257	0.040230	-2.889446
H	-0.427570	2.514801	1.398247
H	7.681175	-0.879084	-0.143224
H	0.893404	-0.001467	4.666369
H	6.481278	0.212537	1.748210
H	4.315550	0.492439	-1.762299
H	-3.876094	-5.082823	-1.762113
H	-1.431818	-0.186783	4.954290
H	-2.529596	4.951895	2.052113
H	2.675709	-2.696724	-1.697062
H	5.641975	2.496254	-2.284624
H	-3.494775	1.432219	1.486520
H	-4.859519	-1.749371	-0.286286
H	0.938317	2.996635	-0.674358
H	-0.949362	5.424421	-1.940985
H	-1.973043	-5.383232	-3.334289
H	-5.485978	-1.359848	2.272926
H	-1.568062	0.697067	-5.279091
H	-3.058884	0.061937	-4.526974
H	-2.116799	-0.968390	-5.647325
H	-1.573977	-4.505654	-0.137474
H	-1.515251	-2.928749	0.680708
H	-0.942987	-4.404593	1.527891
H	-0.705644	-3.409893	-4.155939
H	3.148356	-0.787754	-3.216669
H	3.140416	-2.313083	-4.139627
H	4.694260	-1.532736	-3.710088
H	-5.649847	-1.138157	4.767851
H	-3.602273	-0.568601	6.098096
H	3.386358	2.325547	2.939330
H	3.541386	2.161027	1.168043
H	5.004440	2.137758	2.190412
H	1.798913	1.977689	5.836502
H	0.133584	3.896816	-3.368734

H	-0.356330	2.613869	-4.508699
H	0.072826	2.198624	-2.821201
H	1.593426	4.260633	4.820660
H	0.465839	4.523329	2.584731
H	-4.742707	-4.788221	0.254781
H	-5.562106	-3.562773	1.252804
H	-3.782314	-3.543240	1.106862
H	0.326018	-1.643453	-4.993196
H	0.830032	-1.470136	-3.284820
H	0.749866	-0.037463	-4.349297
H	-2.338062	2.708490	3.351193
H	-3.906839	1.990277	3.801374
H	-3.792374	3.745728	3.494986
H	-3.735905	4.099684	-3.739820
H	-2.609989	3.623239	-5.050792
H	-2.244642	5.042483	-4.025140
H	4.621429	-4.734347	2.221856
H	4.528653	-3.641714	0.818011
H	3.540628	-5.130813	0.866203
H	5.309253	-3.901554	-2.773222
H	3.656719	-4.525225	-3.004804
H	4.404850	-4.552037	-1.378677
H	2.285788	5.024643	-1.180604
H	1.526014	-5.279974	2.644726
H	1.343263	-3.951523	3.821209
H	2.803554	-4.972245	3.844493
H	-6.320774	-2.354074	-2.251394
H	-7.041012	-2.886595	-0.700812
H	-6.238415	-4.071036	-1.775679
H	-5.610219	3.666398	1.660704
H	-5.834787	1.961277	2.167124
H	-5.583522	2.355736	0.443276
H	-0.209936	-1.055697	-0.918369
H	-0.381103	1.615404	-0.452013

Table S75. Cartesian geometry of 3b-TS3 (23.3 kcal/mol) in Figure S145 in Angstrom [Å].

Atomtype	X Coordinates	Y Coordinates	Z Coordinates
C	-1.531669	3.829887	-1.438661
C	-2.486328	3.194912	-0.606123
C	-2.755418	3.622305	0.716649
C	-2.018290	4.717133	1.207890
C	-1.051038	5.350057	0.414034
C	-0.815850	4.914760	-0.896926
N	-3.190068	2.057917	-1.147417
C	-2.695850	0.762586	-1.118909
N	-3.538102	0.096890	-2.003060
C	-4.496151	0.955029	-2.552759
C	-4.276670	2.187503	-2.012143
Ni	-1.152425	0.163359	-0.186414
Ge	0.922800	-0.365119	-0.376473
C	2.527416	0.806116	-0.746016
C	2.302308	2.177440	-0.503878
C	3.354348	3.108031	-0.569114
C	4.655924	2.690561	-0.886565
C	4.885904	1.330773	-1.147158
C	3.836948	0.398458	-1.078847
C	3.068166	4.574531	-0.322967
F	2.180185	4.755271	0.687083
C	6.296995	0.846708	-1.418868
F	6.932363	0.504634	-0.267548

C	-3.377368	-1.260844	-2.459565
C	-4.361305	-2.226091	-2.133506
C	-4.202822	-3.526959	-2.656239
C	-3.094570	-3.862254	-3.439668
C	-2.134154	-2.888554	-3.750557
C	-2.264016	-1.564621	-3.292505
C	-5.595746	-1.902505	-1.289977
C	-5.805400	-2.904267	-0.135241
C	-1.300867	-0.472751	-3.757200
C	-1.900879	0.294319	-4.956791
C	-3.806845	2.907100	1.558663
C	-3.573335	2.994027	3.076119
C	-1.280386	3.388520	-2.881360
C	0.171018	2.923959	-3.108998
P	-1.293067	-0.437707	1.940149
C	-0.365458	0.745455	3.062772
C	0.094654	0.429494	4.360015
C	0.765600	1.388828	5.137375
C	0.991016	2.680252	4.629904
C	0.544249	3.004229	3.338299
C	-0.123832	2.043294	2.563735
C	-0.627594	-2.110817	2.455562
Si	0.421338	-3.080647	1.168126
C	1.331093	-4.372334	2.273174
C	2.341805	-5.335145	1.619142
N	1.601534	-1.953447	0.411946
C	3.011389	-2.185436	0.503377
C	3.769056	-1.650566	1.589184
C	5.162427	-1.859194	1.611930
C	5.812460	-2.572622	0.598955
C	5.064853	-3.096630	-0.463839
C	3.670243	-2.920589	-0.533742
C	3.130981	-0.813670	2.698906
C	3.340978	-1.429983	4.098191
C	2.911669	-3.461706	-1.748557
C	3.171910	-2.593075	-3.000295
C	-0.749239	-3.858660	-0.154062
C	-0.359372	-5.274207	-0.627358
C	-2.992239	-0.459958	2.725247
C	-3.220111	-0.410748	4.117027
C	-4.529375	-0.411559	4.624992
C	-5.629281	-0.454105	3.749408
C	-5.410515	-0.511052	2.363162
C	-4.100247	-0.520771	1.857749
C	3.237109	-4.938539	-2.054675
C	3.647417	0.642520	2.684085
C	-6.858864	-1.838369	-2.179023
C	0.106625	-0.990645	-4.084711
C	-5.230675	3.399501	1.214760
C	-1.678672	4.504762	-3.870637
C	-2.224129	-3.819323	0.291381
C	0.308487	-5.172929	3.116487
F	6.313711	-0.245131	-2.221435
F	7.052278	1.803062	-2.015922
F	4.185199	5.272031	-0.011897
F	2.528178	5.171883	-1.426855
H	-0.668068	-3.180721	-1.026132
H	-5.229245	0.607275	-3.278770
H	-4.768285	3.145120	-2.180026
H	-3.915216	-0.545164	0.777185

H	-1.924989	2.520641	-3.095091
H	-1.498141	-2.733334	2.725710
H	-0.015873	-2.020687	3.371015
H	4.412496	-1.446329	4.373084
H	2.967404	-2.468412	4.151722
H	2.807325	-0.830209	4.858736
H	-0.462604	-6.022902	0.180595
H	0.677302	-5.328323	-1.001731
H	-1.026622	-5.593215	-1.453393
H	-0.467718	6.184802	0.822290
H	5.579586	-3.638538	-1.266662
H	2.044564	-0.776112	2.513591
H	1.903754	-3.726989	2.970044
H	-1.184666	0.233072	-2.918169
H	-0.463359	2.285991	1.550009
H	6.901155	-2.704798	0.626990
H	-0.054665	-0.572881	4.779505
H	5.752806	-1.434470	2.433484
H	4.072303	-0.648752	-1.268060
H	-4.954268	-4.292098	-2.429227
H	-2.376541	-0.347968	4.813419
H	-2.185313	5.064030	2.233076
H	1.834324	-3.392971	-1.521349
H	-3.748189	1.841552	1.282698
H	-5.451658	-0.902919	-0.842340
H	1.293273	2.518936	-0.236742
H	-0.050688	5.412993	-1.502037
H	-2.978626	-4.886313	-3.817131
H	-6.260539	-0.539049	1.670565
H	-1.223470	1.111184	-5.267169
H	-2.877663	0.742724	-4.703129
H	-2.046133	-0.381718	-5.821036
H	-2.873437	-4.267354	-0.482520
H	-2.563354	-2.778194	0.437588
H	-2.395015	-4.377865	1.232940
H	-1.279560	-3.162543	-4.378078
H	2.816058	-1.557881	-2.863323
H	2.647950	-3.010079	-3.880821
H	4.253316	-2.547953	-3.229147
H	-6.652023	-0.439683	4.147807
H	-4.692004	-0.363906	5.709757
H	3.102340	1.246171	3.431589
H	3.505139	1.110921	1.697580
H	4.726817	0.682473	2.924572
H	1.121658	1.121284	6.140827
H	0.901683	3.715622	-2.867354
H	0.319015	2.635208	-4.166338
H	0.390453	2.049314	-2.476049
H	1.523714	3.424644	5.235686
H	0.722877	4.001457	2.918693
H	-5.992463	-3.926028	-0.513319
H	-6.684230	-2.611598	0.467631
H	-4.930104	-2.938683	0.533083
H	5.477663	3.411773	-0.928232
H	0.132880	-1.582568	-5.019408
H	0.491160	-1.624033	-3.267278
H	0.800047	-0.140011	-4.214147
H	-2.545650	2.700374	3.347067
H	-4.268890	2.310532	3.593777
H	-3.756907	4.015855	3.460384

H	-2.730252	4.814563	-3.723259
H	-1.563629	4.154632	-4.914070
H	-1.041332	5.399685	-3.741326
H	2.832480	-5.954118	2.397563
H	3.135892	-4.800712	1.074133
H	1.848661	-6.031052	0.917886
H	4.275725	-5.061166	-2.414877
H	2.567491	-5.318689	-2.848995
H	3.111074	-5.575657	-1.163095
H	-0.345210	-5.796957	2.477580
H	-0.344802	-4.527003	3.730313
H	0.834368	-5.860678	3.808874
H	-6.747448	-1.107223	-2.999858
H	-7.743768	-1.551760	-1.579704
H	-7.062390	-2.824483	-2.637443
H	-5.341723	4.471762	1.466321
H	-5.980568	2.828900	1.794786
H	-5.463322	3.274162	0.143283
H	-0.723844	-1.160968	-0.949493
H	-0.366404	1.465781	-0.331913

Table S76. Cartesian geometry of 3c-TS3 (23.8 kcal/mol) in Figure S145 in Angstrom [Å].

Atomtype	X Coordinates	Y Coordinates	Z Coordinates
C	1.901143	-0.587371	4.432442
C	1.844339	-0.079438	3.117121
C	2.942128	0.654508	2.626081
C	4.082203	0.861908	3.419270
C	4.134138	0.345863	4.724778
C	3.036611	-0.373189	5.231114
P	0.404369	-0.336162	1.945986
C	-0.130722	-2.079980	2.391230
C	-0.840025	-2.433788	3.559988
C	-1.165361	-3.774993	3.824995
C	-0.787718	-4.786746	2.925047
C	-0.091153	-4.445091	1.754275
C	0.226510	-3.104254	1.489166
Ni	0.786381	-0.100179	-0.218512
C	2.536372	0.232078	-0.869764
N	3.155143	1.415068	-1.262956
C	4.426460	1.184410	-1.799099
C	4.637870	-0.161555	-1.746593
N	3.493229	-0.724995	-1.181922
C	2.537773	2.716720	-1.300461
C	3.025153	3.742639	-0.454484
C	2.447887	5.024177	-0.576556
C	1.403229	5.264722	-1.474027
C	0.934782	4.231717	-2.298961
C	1.504636	2.946190	-2.251781
C	3.216514	-2.139318	-1.227846
C	3.443594	-2.937527	-0.079966
C	3.121710	-4.305588	-0.166685
C	2.592308	-4.849800	-1.346795
C	2.390687	-4.038324	-2.471695
C	2.701451	-2.664800	-2.438610
C	4.165958	3.525240	0.541085
C	3.814822	4.021349	1.959396
C	1.101238	1.870345	-3.259293
C	2.082095	1.867259	-4.453141
C	4.004728	-2.309526	1.191881
C	3.661910	-3.067483	2.484886

C	2.506404	-1.800668	-3.685253
C	1.048361	-1.792594	-4.180373
Ge	-1.283123	-0.290530	-0.786136
C	-2.191477	-1.679803	-1.915573
C	-1.428180	-2.859092	-2.081910
C	-1.948921	-3.976089	-2.753891
C	-3.250285	-3.935180	-3.281735
C	-4.019393	-2.768722	-3.132165
C	-3.498856	-1.653013	-2.454916
N	-2.625503	0.596497	0.230637
Si	-2.098180	1.681797	1.564188
C	-1.178619	3.237031	0.885192
C	-2.037039	4.513571	0.776110
C	-4.011771	0.313797	0.017096
C	-4.761868	1.120098	-0.897976
C	-6.112533	0.803776	-1.136322
C	-6.733089	-0.274230	-0.489962
C	-5.996571	-1.053942	0.409802
C	-4.640612	-0.783321	0.680201
C	-4.123159	2.281517	-1.664010
C	-3.876948	1.909518	-3.143394
C	-3.876909	-1.706972	1.629841
C	-4.524765	-1.793383	3.027671
C	-3.584881	2.089196	2.725373
C	-4.803092	2.837475	2.148924
C	-0.920993	0.718657	2.742613
C	-3.091071	2.800406	4.009199
C	-3.715829	-3.122612	1.032463
C	-4.947541	3.583331	-1.579365
C	5.462478	4.202293	0.041183
C	-0.351920	1.988689	-3.739886
C	5.533466	-2.105584	1.093642
C	3.482290	-2.231743	-4.801411
C	0.123733	3.519816	1.658860
H	-0.875534	2.947975	-0.140103
H	5.048258	1.996710	-2.172097
H	5.476780	-0.771627	-2.079784
H	2.896372	1.032075	1.597325
H	2.752034	-0.759065	-3.423933
H	-0.403078	1.454922	3.381516
H	-1.551649	0.113857	3.418575
H	-5.520409	-2.273941	2.983306
H	-4.656290	-0.795670	3.484496
H	-3.890341	-2.401556	3.699197
H	-2.345772	4.890234	1.769580
H	-2.951631	4.360819	0.178276
H	-1.454069	5.321166	0.288724
H	2.337520	-5.916985	-1.389142
H	-6.689174	1.409427	-1.846809
H	-2.863314	-1.293101	1.760657
H	-3.935452	1.078755	3.019447
H	1.177523	0.899105	-2.742880
H	0.755157	-2.827267	0.569420
H	-7.787330	-0.506269	-0.689725
H	-1.156405	-1.666866	4.277509
H	-6.480663	-1.903973	0.907452
H	-4.138364	-0.776934	-2.335489
H	2.813038	5.840337	0.057711
H	1.067778	-1.174155	4.834601
H	3.265800	-4.950620	0.706410

H	-3.137746	2.474026	-1.206557
H	-5.040045	-2.725014	-3.534703
H	3.539388	-1.312770	1.267005
H	4.360183	2.439657	0.606170
H	-0.410090	-2.902590	-1.671267
H	1.980944	-4.474328	-3.391261
H	0.951857	6.263247	-1.536409
H	4.934280	1.419369	3.011290
H	1.817025	1.065082	-5.166351
H	3.121880	1.696558	-4.122027
H	2.046744	2.833718	-4.991574
H	0.630252	4.412448	1.249227
H	0.827817	2.673657	1.564386
H	-0.057631	3.706253	2.735932
H	0.124413	4.438142	-3.006095
H	-3.195344	1.048216	-3.242545
H	-3.426238	2.759718	-3.689129
H	-4.828814	1.646223	-3.642979
H	5.026541	0.499488	5.345329
H	3.070320	-0.782205	6.249544
H	-3.092574	-3.746683	1.697648
H	-3.235244	-3.089493	0.041756
H	-4.701075	-3.613622	0.915925
H	-1.723230	-4.028344	4.735999
H	0.685967	-2.807157	-4.427018
H	0.958838	-1.172825	-5.091903
H	0.383487	-1.374679	-3.408380
H	-1.047835	-5.832959	3.131770
H	0.205962	-5.219448	1.035324
H	3.645070	5.113549	1.976810
H	4.646571	3.807273	2.654994
H	2.910271	3.525622	2.347017
H	-3.664446	-4.807742	-3.804174
H	-0.506791	2.853582	-4.412806
H	-1.039607	2.091742	-2.883418
H	-0.638441	1.080081	-4.299684
H	2.581185	-3.269970	2.565259
H	3.956831	-2.456308	3.355855
H	4.204014	-4.030727	2.550044
H	4.531144	-2.199531	-4.451673
H	3.388582	-1.560901	-5.676496
H	3.271110	-3.263684	-5.140474
H	-5.596951	2.917991	2.919317
H	-5.238824	2.320000	1.279874
H	-4.544187	3.867185	1.844994
H	-5.902221	3.497347	-2.131752
H	-4.382724	4.421717	-2.028862
H	-5.185056	3.847727	-0.535214
H	-1.334534	-4.880406	-2.859514
H	-2.674057	3.800951	3.785143
H	-2.313855	2.229513	4.548369
H	-3.933720	2.953461	4.713389
H	5.754350	3.840728	-0.961287
H	6.299926	4.003324	0.736600
H	5.328432	5.298474	-0.026079
H	6.050448	-3.080902	1.009841
H	5.905864	-1.593876	2.001147
H	5.814231	-1.492790	0.220252
H	-0.031012	1.186142	-0.641508
H	0.625667	-1.442781	-0.925509

Table S77. Cartesian geometry of 3d-TS3 (23.5 kcal/mol) in Figure S145 in Angstrom [Å].

Atomtype	X Coordinates	Y Coordinates	Z Coordinates
C	-2.337672	3.310878	-1.887992
C	-2.971850	2.608472	-0.833637
C	-3.183863	3.174882	0.447275
C	-2.722013	4.487769	0.660952
C	-2.073388	5.200479	-0.359079
C	-1.889206	4.619324	-1.621138
N	-3.391626	1.254066	-1.095090
C	-2.556352	0.153186	-0.954130
N	-3.267372	-0.849002	-1.607976
C	-4.474709	-0.374284	-2.131307
C	-4.551759	0.947914	-1.807191
Ni	-0.822966	0.149257	-0.185916
Ge	1.284097	0.222937	-0.627714
C	2.390345	1.702845	-1.400874
C	1.765985	2.968870	-1.339144
C	2.439381	4.136845	-1.728590
C	3.765282	4.086122	-2.201405
C	4.387774	2.821297	-2.274675
C	3.720526	1.652597	-1.881869
C	4.512777	5.335588	-2.612274
C	-2.783092	-2.176287	-1.891781
C	-3.417786	-3.291647	-1.292668
C	-2.964097	-4.577824	-1.653778
C	-1.898893	-4.745925	-2.543530
C	-1.284167	-3.625901	-3.122072
C	-1.725174	-2.321670	-2.832289
C	-4.587944	-3.155201	-0.317089
C	-4.373444	-3.963230	0.979877
C	-1.157903	-1.115714	-3.580015
C	-2.061163	-0.762959	-4.782930
C	-3.877303	2.365134	1.538269
C	-3.536207	2.800571	2.972772
C	-2.156134	2.703845	-3.279923
C	-0.678312	2.631590	-3.706430
P	-0.543467	-0.100902	1.992377
C	0.136140	1.448570	2.807594
C	0.805211	1.479713	4.050809
C	1.246294	2.695970	4.599456
C	1.026688	3.903422	3.913804
C	0.371746	3.883043	2.671528
C	-0.061519	2.666244	2.123136
C	0.625290	-1.431632	2.601217
Si	1.760487	-2.263103	1.289262
C	3.128437	-3.065177	2.389125
C	4.295270	-3.808291	1.709035
N	2.469422	-0.995359	0.229876
C	3.886374	-0.825686	0.129672
C	4.580274	0.042412	1.026379
C	5.969643	0.213884	0.867551
C	6.677068	-0.443448	-0.146027
C	5.991972	-1.294931	-1.023878
C	4.605332	-1.505380	-0.905287
C	3.857768	0.834804	2.116520
C	4.418665	0.554862	3.526605
C	3.900198	-2.408991	-1.919949
C	3.775410	-1.714800	-3.294833
C	0.725162	-3.535672	0.272497

C	1.456874	-4.845117	-0.085810
C	-2.066059	-0.430046	3.033815
C	-2.146463	-0.198118	4.423437
C	-3.341508	-0.443801	5.119490
C	-4.475322	-0.918433	4.436005
C	-4.401307	-1.159415	3.054037
C	-3.202650	-0.921905	2.362097
C	4.586108	-3.781570	-2.083501
C	3.887517	2.352244	1.827145
C	-5.913720	-3.570354	-0.994738
C	0.301792	-1.294232	-4.020384
C	-5.410487	2.353067	1.344695
C	-3.010244	3.462602	-4.318475
C	-0.633483	-3.834568	0.935965
C	2.488543	-3.968716	3.471700
H	0.499954	-3.007134	-0.674283
H	-5.152439	-1.019530	-2.688096
H	-5.303567	1.702332	-2.036663
H	-3.136435	-1.083609	1.279216
H	-2.524173	1.665780	-3.253634
H	0.003583	-2.218698	3.062111
H	1.277491	-1.043844	3.404345
H	5.461951	0.910257	3.624324
H	4.409959	-0.523563	3.767879
H	3.812580	1.084860	4.284890
H	1.676317	-5.454868	0.810983
H	2.410944	-4.669338	-0.611320
H	0.821403	-5.463426	-0.751757
H	-1.710787	6.219011	-0.167426
H	6.546362	-1.802307	-1.823471
H	2.799639	0.524772	2.114267
H	3.562550	-2.184542	2.905165
H	-1.166974	-0.266595	-2.876699
H	-0.558144	2.639531	1.145926
H	7.758840	-0.291214	-0.254748
H	0.999064	0.553813	4.606041
H	6.505052	0.889124	1.547281
H	4.264437	0.707957	-1.934712
H	-3.443989	-5.459857	-1.213920
H	-1.282709	0.198823	4.968280
H	-2.850756	4.952440	1.644107
H	2.876420	-2.584354	-1.547719
H	5.423378	2.749309	-2.635608
H	-3.518073	1.329510	1.419558
H	-4.675905	-2.090383	-0.036312
H	0.733608	3.043124	-0.970925
H	-1.384700	5.186313	-2.413351
H	-1.545730	-5.754761	-2.792952
H	-5.280703	-1.523831	2.508978
H	-1.675323	0.133052	-5.303354
H	-3.096453	-0.549466	-4.462766
H	-2.090794	-1.598048	-5.508871
H	-1.206431	-4.561803	0.332427
H	-1.245544	-2.917689	1.008094
H	-0.521424	-4.261365	1.952388
H	-0.458950	-3.773453	-3.826744
H	3.190259	-0.782009	-3.232313
H	3.273060	-2.379203	-4.022865
H	4.775358	-1.459914	-3.695025
H	-5.412655	-1.095849	4.979155

H	-3.391903	-0.250017	6.199098
H	3.288353	2.893695	2.580984
H	3.475741	2.580874	0.831260
H	4.924377	2.738414	1.863870
H	1.770361	2.697886	5.564192
H	-0.200454	3.628044	-3.722916
H	-0.595090	2.201362	-4.721840
H	-0.109077	1.995705	-3.010552
H	1.376780	4.851547	4.342162
H	0.197394	4.814081	2.116918
H	-4.315216	-5.048324	0.776794
H	-5.219566	-3.803822	1.672801
H	-3.448496	-3.658994	1.495749
H	0.408620	-2.019660	-4.849386
H	0.924377	-1.640302	-3.177915
H	0.709922	-0.329484	-4.372000
H	-2.447135	2.862657	3.132326
H	-3.940416	2.060315	3.685318
H	-3.982462	3.783611	3.218676
H	-4.076617	3.479842	-4.025214
H	-2.931884	2.978480	-5.310490
H	-2.672539	4.510988	-4.424817
H	5.029424	-4.138911	2.471908
H	4.832800	-3.171689	0.988653
H	3.950370	-4.714624	1.180405
H	5.574343	-3.687721	-2.571867
H	3.967659	-4.442896	-2.719232
H	4.736473	-4.279875	-1.111034
H	1.923472	5.104761	-1.657209
H	1.979679	-4.842978	3.022388
H	1.747322	-3.437067	4.095165
H	3.267758	-4.364790	4.153853
H	-6.107802	-2.983996	-1.910887
H	-6.766893	-3.424818	-0.305214
H	-5.886140	-4.638361	-1.282306
H	-5.822745	3.375344	1.447453
H	-5.883357	1.711076	2.111731
H	-5.699793	1.964805	0.353353
H	-0.118946	-1.106877	-0.838256
H	-0.488372	1.581644	-0.589655
H	4.864725	5.264368	-3.659961
H	3.883101	6.238961	-2.523817
H	5.412513	5.484549	-1.983458

Table S78. Cartesian geometry of 3e-TS3 (23.9 kcal/mol) in Figure S145 in Angstrom [Å].

Atomtype	X Coordinates	Y Coordinates	Z Coordinates
C	-1.859244	3.722120	-1.543386
C	-2.637997	3.001344	-0.604673
C	-2.838874	3.442831	0.726120
C	-2.214473	4.644179	1.112634
C	-1.420638	5.368540	0.210177
C	-1.250976	4.914716	-1.105048
N	-3.220012	1.756231	-1.040017
C	-2.546734	0.541853	-0.996514
N	-3.347810	-0.273812	-1.790831
C	-4.451461	0.418109	-2.301148
C	-4.370055	1.694059	-1.827394
Ni	-0.874786	0.216215	-0.163928
Ge	1.245979	0.063834	-0.514151
C	2.566498	1.453277	-1.069846

C	2.106213	2.783884	-0.898639
C	2.935905	3.884609	-1.119490
C	4.274305	3.692356	-1.530751
C	4.752871	2.377547	-1.718363
C	3.905090	1.281464	-1.485972
O	5.013130	4.822202	-1.714937
C	6.371528	4.697827	-2.105988
C	-3.030599	-1.614780	-2.212513
C	-3.842566	-2.690837	-1.778209
C	-3.552009	-3.977230	-2.279417
C	-2.473919	-4.190433	-3.143700
C	-1.679982	-3.109942	-3.555072
C	-1.952988	-1.799011	-3.123239
C	-5.028485	-2.505281	-0.830190
C	-4.983138	-3.478151	0.366776
C	-1.186544	-0.607671	-3.696076
C	-1.962690	0.001016	-4.885326
C	-3.693368	2.620009	1.685072
C	-3.374875	2.836427	3.173435
C	-1.691584	3.258866	-2.991330
C	-0.218252	3.028814	-3.375546
P	-0.747276	-0.315681	1.976711
C	0.090646	1.017760	3.000428
C	0.691355	0.809625	4.261420
C	1.259964	1.880118	4.972487
C	1.239120	3.178623	4.434247
C	0.654881	3.395242	3.175582
C	0.093563	2.323167	2.465163
C	0.198576	-1.857947	2.458323
Si	1.293704	-2.670500	1.101204
C	2.486483	-3.765682	2.151633
C	3.585426	-4.576595	1.436348
N	2.217225	-1.395893	0.233372
C	3.646766	-1.410838	0.205832
C	4.401497	-0.756970	1.226790
C	5.807595	-0.756394	1.138463
C	6.474664	-1.379015	0.076622
C	5.730346	-2.021753	-0.922342
C	4.323825	-2.055529	-0.878794
C	3.733066	-0.009070	2.380757
C	4.193959	-0.513026	3.764479
C	3.560555	-2.730564	-2.020904
C	3.602235	-1.871784	-3.304808
C	0.166025	-3.667784	-0.106944
C	0.738127	-5.020420	-0.577513
C	-2.352379	-0.548671	2.915973
C	-2.474949	-0.465754	4.319309
C	-3.727259	-0.621063	4.936297
C	-4.876147	-0.855060	4.159616
C	-4.761542	-0.947150	2.762522
C	-3.506989	-0.800707	2.148861
C	4.067791	-4.156276	-2.323232
C	3.957521	1.515515	2.270205
C	-6.365471	-2.652159	-1.591594
C	0.259592	-0.937533	-4.091775
C	-5.201194	2.846563	1.433267
C	-2.382453	4.244819	-3.958139
C	-1.261870	-3.841538	0.445969
C	1.680543	-4.692016	3.095076
H	0.077464	-3.013533	-0.996163

H	-5.177442	-0.061885	-2.955554
H	-5.001854	2.565605	-1.995558
H	-3.405407	-0.847429	1.057671
H	-2.198155	2.286864	-3.102663
H	-0.547670	-2.604085	2.782432
H	0.843399	-1.666498	3.334804
H	5.265392	-0.297584	3.937601
H	4.050922	-1.603289	3.875528
H	3.618541	-0.004566	4.560473
H	0.819173	-5.746710	0.253225
H	1.737764	-4.923381	-1.034360
H	0.068781	-5.470291	-1.338639
H	-0.932309	6.296435	0.535921
H	6.253928	-2.502934	-1.758132
H	2.646491	-0.182759	2.311050
H	2.999873	-3.012602	2.783949
H	-1.122958	0.149505	-2.897174
H	-0.349631	2.481605	1.474868
H	7.571256	-1.362977	0.025596
H	0.730996	-0.193079	4.704306
H	6.390647	-0.244598	1.914853
H	4.329568	0.284576	-1.616868
H	-4.171188	-4.827082	-1.969298
H	-1.596318	-0.255539	4.939234
H	-2.330895	5.007543	2.139056
H	2.504289	-2.805359	-1.710920
H	5.784674	2.187711	-2.030986
H	-3.471415	1.564459	1.456306
H	-4.985241	-1.477697	-0.426923
H	1.070888	2.956770	-0.574443
H	-0.631976	5.490737	-1.804058
H	-2.250309	-5.203047	-3.503334
H	-5.651349	-1.123270	2.145636
H	-1.429791	0.886040	-5.279216
H	-2.976273	0.321295	-4.585292
H	-2.063227	-0.735198	-5.705794
H	-1.886276	-4.408294	-0.268220
H	-1.746423	-2.859733	0.594674
H	-1.277599	-4.390089	1.408632
H	-0.845790	-3.290026	-4.241449
H	3.142298	-0.881219	-3.150854
H	3.056600	-2.372347	-4.126670
H	4.647179	-1.712348	-3.633165
H	-5.856212	-0.961425	4.642592
H	-3.808698	-0.544773	6.028646
H	3.403309	2.040010	3.068889
H	3.611160	1.903920	1.299157
H	5.031998	1.761882	2.372363
H	1.727603	1.695937	5.948598
H	0.393499	3.940207	-3.247538
H	-0.144685	2.720051	-4.434948
H	0.222129	2.236043	-2.750751
H	1.688570	4.012244	4.989273
H	0.636443	4.400106	2.734345
H	-5.062183	-4.530370	0.037115
H	-5.830194	-3.283096	1.049344
H	-4.049544	-3.366180	0.941425
H	0.317494	-1.568937	-4.998923
H	0.778550	-1.463387	-3.272497
H	0.814016	-0.005321	-4.302826

H	-2.297967	2.724123	3.380727
H	-3.914769	2.084727	3.775611
H	-3.695140	3.838502	3.518785
H	-3.449579	4.378523	-3.699586
H	-2.322359	3.871756	-4.998266
H	-1.900170	5.240210	-3.926028
H	4.231575	-5.080194	2.184151
H	4.235600	-3.941983	0.813675
H	3.158028	-5.368392	0.795760
H	5.084004	-4.143047	-2.760458
H	3.401849	-4.650314	-3.055620
H	4.100823	-4.777821	-1.412537
H	2.573023	4.909333	-0.974263
H	1.092486	-5.437892	2.526752
H	0.977933	-4.140873	3.745671
H	2.365690	-5.258867	3.757407
H	-6.437253	-1.942694	-2.435608
H	-7.222017	-2.470370	-0.914968
H	-6.468414	-3.673331	-2.004801
H	-5.476033	3.898074	1.643916
H	-5.798082	2.193697	2.097802
H	-5.485723	2.620131	0.391634
H	-0.302605	-1.038539	-0.934291
H	-0.333672	1.626679	-0.377652
H	6.770479	5.723480	-2.185095
H	6.965567	4.134021	-1.356341
H	6.472951	4.192810	-3.089644

Table S79. Cartesian geometry of 3f-TS3 (23.9 kcal/mol) in Figure S145 in Angstrom [Å].

Atomtype	X Coordinates	Y Coordinates	Z Coordinates
C	-1.375248	3.954485	-1.329506
C	-2.261077	3.289514	-0.446186
C	-2.425575	3.672464	0.907919
C	-1.655458	4.754840	1.374480
C	-0.757243	5.421104	0.526604
C	-0.623758	5.027744	-0.811880
N	-2.997062	2.163989	-0.965418
C	-2.499126	0.866581	-0.982256
N	-3.383818	0.217965	-1.840186
C	-4.366567	1.085377	-2.329020
C	-4.122408	2.308218	-1.777771
Ni	-0.911078	0.263648	-0.143624
Ge	1.180245	-0.148587	-0.471659
C	2.678441	1.074294	-0.921892
C	2.400896	2.440650	-0.679414
C	3.368657	3.439051	-0.814335
C	4.698863	3.119483	-1.216336
C	4.980676	1.744611	-1.471212
C	3.998874	0.760236	-1.324434
N	5.671983	4.096252	-1.348655
C	5.352628	5.482231	-1.051531
C	-3.241220	-1.126603	-2.338060
C	-4.200810	-2.105619	-1.982604
C	-4.074184	-3.388560	-2.555762
C	-3.014471	-3.695806	-3.414416
C	-2.072710	-2.711241	-3.747192
C	-2.176470	-1.402658	-3.240780
C	-5.374021	-1.814726	-1.045272
C	-5.495096	-2.855603	0.087441
C	-1.241467	-0.295942	-3.726286

C	-1.899195	0.483170	-4.887141
C	-3.391116	2.906909	1.807353
C	-3.089515	3.015341	3.310941
C	-1.238334	3.557305	-2.800093
C	0.200400	3.157118	-3.175621
P	-0.904615	-0.409610	1.959600
C	0.082066	0.732207	3.079155
C	0.618421	0.368903	4.333971
C	1.310076	1.306976	5.119215
C	1.479330	2.625694	4.662435
C	0.960897	2.994752	3.410390
C	0.275948	2.054115	2.626058
C	-0.185376	-2.092492	2.356413
Si	0.819643	-2.961576	0.964140
C	1.832844	-4.270725	1.957482
C	2.826333	-5.175834	1.202387
N	1.924801	-1.774809	0.191020
C	3.339737	-1.979844	0.182192
C	4.151988	-1.499965	1.254417
C	5.546966	-1.684564	1.186594
C	6.148682	-2.323671	0.095843
C	5.348143	-2.793520	-0.954512
C	3.949346	-2.638025	-0.933766
C	3.563381	-0.740867	2.443857
C	3.923413	-1.383163	3.799810
C	3.129162	-3.126085	-2.130121
C	3.323254	-2.202957	-3.353996
C	-0.406700	-3.723132	-0.318045
C	-0.006800	-5.105040	-0.873516
C	-2.547671	-0.477089	2.859991
C	-2.692817	-0.454101	4.263374
C	-3.969692	-0.469169	4.848915
C	-5.120030	-0.500924	4.040556
C	-4.984255	-0.531940	2.642552
C	-3.706675	-0.526065	2.059965
C	3.441019	-4.587194	-2.516593
C	3.988262	0.744427	2.430223
C	-6.698124	-1.727363	-1.837779
C	0.155222	-0.797168	-4.119632
C	-4.858984	3.309228	1.539732
C	-1.772948	4.678990	-3.716593
C	-1.854677	-3.745862	0.209166
C	0.891960	-5.136575	2.830485
C	7.029215	3.726005	-1.714824
H	-0.393652	-3.005478	-1.161407
H	-5.133614	0.750170	-3.025440
H	-4.623283	3.267485	-1.904300
H	-3.584537	-0.525221	0.969685
H	-1.864884	2.668445	-2.976255
H	-1.032334	-2.748118	2.623702
H	0.460809	-2.043631	3.251308
H	5.009678	-1.321363	4.001548
H	3.637048	-2.449889	3.840462
H	3.401548	-0.852202	4.617819
H	-0.038038	-5.889198	-0.093430
H	1.005752	-5.110515	-1.311740
H	-0.713950	-5.411625	-1.670877
H	-0.157888	6.255462	0.914433
H	5.821540	-3.285991	-1.813400
H	2.465274	-0.764014	2.348010

H	2.430168	-3.634258	2.642108
H	-1.093690	0.395966	-2.880493
H	-0.117001	2.328861	1.639745
H	7.238373	-2.453486	0.061800
H	0.510419	-0.654381	4.714056
H	6.175485	-1.306184	2.003013
H	4.297487	-0.272773	-1.511482
H	-4.809286	-4.162964	-2.307338
H	-1.809470	-0.399646	4.909272
H	-1.741096	5.067608	2.420357
H	2.065234	-3.070437	-1.842614
H	5.982168	1.427011	-1.776162
H	-3.278963	1.845623	1.529307
H	-5.199856	-0.829568	-0.576511
H	1.390992	2.734038	-0.361233
H	0.079373	5.556340	-1.467574
H	-2.920608	-4.706994	-3.830877
H	-5.874140	-0.549932	2.001385
H	-1.241419	1.308113	-5.217635
H	-2.866347	0.921562	-4.583102
H	-2.080037	-0.182398	-5.752979
H	-2.534477	-4.178811	-0.547054
H	-2.211890	-2.721743	0.419447
H	-1.957391	-4.349440	1.132827
H	-1.253978	-2.963319	-4.429489
H	3.001818	-1.169300	-3.142829
H	2.735036	-2.569873	-4.216088
H	4.388434	-2.171474	-3.653180
H	-6.117197	-0.497460	4.499694
H	-4.067126	-0.441456	5.942305
H	3.492651	1.288328	3.254129
H	3.713618	1.231495	1.480925
H	5.083819	0.839579	2.558510
H	1.724480	1.002702	6.089342
H	0.923280	3.971214	-2.985557
H	0.256266	2.900938	-4.250044
H	0.520532	2.280145	-2.591627
H	2.024526	3.355119	5.275487
H	1.090363	4.016971	3.031873
H	-5.712010	-3.864051	-0.310171
H	-6.323619	-2.585484	0.767190
H	-4.569744	-2.911748	0.683161
H	0.148065	-1.375893	-5.063063
H	0.578597	-1.436450	-3.326475
H	0.836146	0.060926	-4.264042
H	-2.038001	2.772299	3.535904
H	-3.726116	2.301862	3.863022
H	-3.305133	4.029935	3.698657
H	-2.819227	4.936780	-3.466862
H	-1.738285	4.361257	-4.776090
H	-1.166064	5.599119	-3.618082
H	3.387108	-5.806745	1.922042
H	3.564908	-4.596528	0.626225
H	2.307648	-5.861482	0.509267
H	4.460174	-4.688997	-2.935044
H	2.731896	-4.936625	-3.290601
H	3.364658	-5.262919	-1.648040
H	3.079433	4.471544	-0.595626
H	0.221219	-5.760221	2.208847
H	0.256078	-4.536812	3.506335

H	1.481745	-5.830187	3.463472
H	-6.649471	-0.964318	-2.635386
H	-7.539080	-1.470294	-1.165971
H	-6.929394	-2.697094	-2.317708
H	-5.023376	4.372770	1.799648
H	-5.537624	2.693108	2.159267
H	-5.142845	3.166207	0.483479
H	-0.497514	-1.003652	-0.988909
H	-0.178640	1.597940	-0.253036
H	6.240675	6.113131	-1.218681
H	4.538137	5.866204	-1.701210
H	5.029356	5.620254	0.002712
H	7.659588	4.628535	-1.767006
H	7.485966	3.032778	-0.976563
H	7.068306	3.228330	-2.706435

Table S80. Cartesian geometry of 3g-TS3 (27.6 kcal/mol) in Figure S145 in Angstrom [Å].

Atomtype	X Coordinates	Y Coordinates	Z Coordinates
C	-2.592231	-0.261950	-2.824934
C	-1.536332	-1.191885	-2.899410
C	-1.543373	-2.143975	-3.940818
C	-2.589619	-2.168310	-4.877881
C	-3.646545	-1.245188	-4.784841
C	-3.643312	-0.289060	-3.755559
P	-0.216931	-1.083247	-1.573046
C	1.260796	-0.494228	-2.557545
Si	2.438259	0.748790	-1.678027
C	1.622005	2.497675	-1.703863
C	0.405023	2.556426	-2.647067
Ni	-0.731520	-0.044196	0.290490
Ge	1.260390	-0.203761	1.112546
N	2.769145	0.197758	0.002405
C	4.114301	-0.055368	0.411212
C	4.699795	-1.343543	0.225894
C	6.009905	-1.569599	0.691922
C	6.746432	-0.556666	1.318848
C	6.176003	0.713779	1.479553
C	4.871921	0.991548	1.029539
C	3.918085	-2.493703	-0.407678
C	3.440257	-3.492727	0.667494
C	4.284473	2.388216	1.250941
C	5.233930	3.515755	0.793764
C	-2.473091	0.622659	0.591927
N	-3.547767	-0.091733	1.106816
C	-4.677997	0.707912	1.282016
C	-4.337618	1.961816	0.869708
N	-3.004288	1.902207	0.450172
C	-3.412750	-1.394176	1.709466
C	-3.060140	-1.442704	3.080954
C	-2.886657	-2.713359	3.663384
C	-3.066548	-3.881425	2.909771
C	-3.438760	-3.803117	1.559045
C	-3.618314	-2.557333	0.927648
C	-2.916678	-0.177427	3.928757
C	-4.051547	-0.099402	4.973083
C	-4.011700	-2.433615	-0.541013
C	-5.515540	-2.113634	-0.697901
C	-2.268552	3.086419	0.086509
C	-2.582046	3.756241	-1.121655
C	-1.896415	4.956415	-1.404120

C	-0.911882	5.447499	-0.541385
C	-0.617743	4.765857	0.648511
C	-1.304339	3.590258	1.003888
C	-3.651365	3.257125	-2.095136
C	-4.906977	4.156564	-2.033401
C	-1.105751	2.949537	2.377376
C	0.299621	3.157901	2.959410
N	1.884171	-0.968894	2.702454
C	1.058960	-1.966838	3.372964
C	3.184789	-0.858802	3.350284
C	-3.132570	3.159691	-3.545139
C	-2.190928	3.456810	3.353848
C	-3.647837	-3.650162	-1.407940
C	-1.532889	-0.048738	4.590935
C	0.177526	-2.899121	-1.292362
C	0.926435	-3.708127	-2.174708
C	1.133249	-5.071151	-1.901682
C	0.596060	-5.649741	-0.738521
C	-0.139189	-4.851722	0.153336
C	-0.338699	-3.489619	-0.119368
C	4.035563	0.597701	-2.755704
C	3.696128	0.803486	-4.253074
C	5.276218	1.436114	-2.388320
C	4.699000	-3.223923	-1.518245
C	3.876625	2.611908	2.724726
C	2.583615	3.669662	-1.988327
H	1.227710	2.624569	-0.676502
H	-4.907092	2.889394	0.840355
H	-5.601569	0.316584	1.707138
H	-2.587881	0.462931	-2.002029
H	-3.025660	0.695285	3.266171
H	0.866884	-0.003489	-3.464267
H	1.869555	-1.346383	-2.909292
H	5.565112	-3.780983	-1.113626
H	5.079747	-2.520637	-2.281725
H	4.039614	-3.958035	-2.018266
H	2.983568	3.632938	-3.019402
H	3.444178	3.693613	-1.298547
H	2.047288	4.634421	-1.881952
H	-2.920186	-4.863063	3.379298
H	6.756354	1.505482	1.969922
H	3.011839	-2.066830	-0.868051
H	4.311456	-0.468762	-2.620139
H	-1.230539	1.861597	2.248930
H	-0.901845	-2.860465	0.579797
H	7.764067	-0.755184	1.679687
H	1.365552	-3.284581	-3.086263
H	6.458990	-2.563327	0.568392
H	-2.126569	5.501980	-2.326751
H	-0.742997	-2.888593	-4.014093
H	-3.570504	-4.724651	0.982342
H	3.362933	2.452996	0.648008
H	-3.444135	-1.573300	-0.932654
H	-3.952715	2.241971	-1.780920
H	-2.606123	-2.786424	4.721463
H	-0.372353	6.369824	-0.792206
H	-4.464863	0.432768	-3.669162
H	-2.076060	2.974668	4.342011
H	-3.206692	3.232321	2.982786
H	-2.110061	4.551883	3.493091

H	-0.041139	3.567015	-2.639091
H	-0.380332	1.851025	-2.320469
H	0.672372	2.321043	-3.696311
H	0.144965	5.170849	1.321960
H	3.084388	1.913902	3.041698
H	3.492609	3.639461	2.868328
H	4.744627	2.471671	3.396896
H	-4.469891	-1.274443	-5.510333
H	-2.586258	-2.920807	-5.677504
H	2.841501	-4.299199	0.208548
H	2.811752	-2.982569	1.414654
H	4.303787	-3.947484	1.190071
H	1.723003	-5.681088	-2.598488
H	-1.310136	-0.907816	5.249573
H	-1.492959	0.865931	5.210924
H	-0.743201	0.017396	3.825435
H	0.761934	-6.713703	-0.525299
H	-0.561788	-5.283149	1.069971
H	-2.840803	4.150488	-3.939135
H	-3.923596	2.761970	-4.206709
H	-2.260736	2.489884	-3.616715
H	0.474419	4.203121	3.278601
H	1.071266	2.886966	2.219629
H	0.437859	2.514373	3.847010
H	-2.589538	-3.934857	-1.290247
H	-3.814958	-3.403523	-2.471159
H	-4.275911	-4.528617	-1.163308
H	-5.044785	-0.152700	4.489576
H	-3.992346	0.850201	5.538212
H	-3.984563	-0.932345	5.698527
H	6.120764	1.175294	-3.058373
H	5.610359	1.260019	-1.354158
H	5.089245	2.517535	-2.512720
H	6.121269	3.592674	1.450055
H	4.712823	4.490878	0.832150
H	5.590203	3.355054	-0.237741
H	3.351321	1.836128	-4.453313
H	2.914153	0.114854	-4.619696
H	4.597218	0.638683	-4.877767
H	-5.316820	4.220300	-1.009194
H	-5.700242	3.765496	-2.698453
H	-4.665697	5.185801	-2.359745
H	-6.131965	-2.947653	-0.310629
H	-5.761327	-1.969467	-1.766876
H	-5.803254	-1.195568	-0.158465
H	0.292861	1.235718	0.557316
H	-0.646201	-0.971388	1.513748
H	1.541233	-2.970968	3.334024
H	0.913619	-1.713085	4.444855
H	0.063335	-2.044241	2.903527
H	3.721311	-1.833566	3.340117
H	3.836875	-0.127898	2.862516
H	3.065305	-0.557842	4.415680

Table S81. Cartesian geometry of 4a-Prod. (0.0 kcal/mol) in Figure S145 in Angstrom [Å].

Atomtype	X Coordinates	Y Coordinates	Z Coordinates
C	-2.185560	-1.060431	4.103782
C	-2.142521	-0.473959	2.820108
C	-3.312401	0.116968	2.310350
C	-4.494234	0.154171	3.064664

C	-4.527925	-0.425375	4.343202
C	-3.372030	-1.040775	4.855969
P	-0.579378	-0.300558	1.802900
C	0.083325	1.231083	2.637791
C	0.494892	1.249981	3.987062
C	0.922441	2.447051	4.581836
C	0.942599	3.641248	3.837241
C	0.522697	3.632315	2.498347
C	0.090758	2.434361	1.907738
Ni	-0.713035	0.074255	-0.373084
C	-2.514803	0.392231	-0.971415
N	-3.465685	-0.496067	-1.443234
C	-4.596924	0.171468	-1.931606
C	-4.365806	1.506226	-1.780911
N	-3.099711	1.625851	-1.205578
C	-3.353924	-1.934557	-1.570181
C	-4.221417	-2.750416	-0.800229
C	-4.254922	-4.131205	-1.084654
C	-3.433206	-4.686745	-2.069481
C	-2.547013	-3.868483	-2.784241
C	-2.493748	-2.478677	-2.566746
C	-2.398151	2.880339	-1.077003
C	-2.643315	3.710045	0.044025
C	-1.909763	4.910673	0.139843
C	-0.991616	5.277481	-0.851404
C	-0.811821	4.466414	-1.981952
C	-1.513893	3.255522	-2.126003
C	-5.114415	-2.198396	0.310033
C	-4.837783	-2.898863	1.656948
C	-1.595347	-1.588458	-3.424129
C	-2.403699	-0.929953	-4.564272
C	-3.731483	3.395503	1.068450
C	-3.245709	3.475053	2.527933
C	-1.411338	2.435177	-3.411113
C	0.003897	2.375195	-4.012183
Ge	1.521102	-0.164797	-0.996096
C	2.617160	1.508693	-1.068444
C	1.942550	2.739104	-0.911499
C	2.616232	3.965220	-0.946346
C	4.016870	3.988700	-1.146138
C	4.709804	2.767422	-1.329050
C	4.014157	1.551433	-1.293918
C	4.722466	5.240282	-1.166543
N	5.290076	6.265417	-1.183096
N	2.485932	-1.427370	0.146970
Si	1.519345	-2.563210	1.072990
C	0.222131	-3.396552	-0.080210
C	0.792806	-4.561404	-0.914206
C	3.901296	-1.286356	0.225602
C	4.743645	-1.858986	-0.786671
C	6.139125	-1.701497	-0.688837
C	6.723832	-0.993114	0.370046
C	5.900077	-0.402541	1.335871
C	4.497352	-0.515514	1.275314
C	4.168042	-2.579393	-2.008240
C	4.379469	-1.745562	-3.292793
C	3.654131	0.234346	2.307264
C	3.822492	-0.347850	3.726009
C	2.597153	-3.808949	2.069657
C	3.692145	-4.557551	1.287362

C	0.493820	-1.676957	2.438371
C	1.727614	-4.813570	2.860607
C	3.947097	1.750206	2.321165
C	4.737467	-3.998875	-2.213632
C	-6.609446	-2.298177	-0.062544
C	-0.352758	-2.302190	-3.982934
C	-4.947803	4.321869	0.837448
C	-2.423883	2.972491	-4.448947
C	-1.065821	-3.826058	0.652661
H	-0.076186	-2.588759	-0.785464
H	-5.443827	-0.372315	-2.347584
H	-4.970077	2.377710	-2.030401
H	-3.276384	0.548630	1.305895
H	-1.700776	1.401651	-3.168252
H	-0.163423	-2.437609	2.892720
H	1.167528	-1.312935	3.232771
H	4.863427	-0.226060	4.081560
H	3.585697	-1.426165	3.758264
H	3.157843	0.175190	4.438973
H	1.027032	-5.433797	-0.274540
H	1.717860	-4.286470	-1.452128
H	0.053308	-4.899918	-1.666891
H	-0.425018	6.212684	-0.754705
H	6.782207	-2.139719	-1.463192
H	2.598598	0.118465	2.013519
H	3.111647	-3.161718	2.809666
H	-1.223917	-0.788128	-2.762714
H	-0.274909	2.429830	0.875646
H	7.814977	-0.889223	0.432236
H	0.476203	0.330718	4.584972
H	6.354722	0.178478	2.148777
H	4.586268	0.628639	-1.425919
H	-4.928512	-4.779572	-0.511967
H	-1.289964	-1.527487	4.531578
H	-2.069105	5.568508	1.002046
H	3.081778	-2.669498	-1.843002
H	5.794345	2.778171	-1.489115
H	-4.075005	2.363614	0.887663
H	-4.872079	-1.130906	0.445116
H	0.858027	2.744022	-0.773081
H	-0.108581	4.777605	-2.761645
H	-3.468086	-5.764719	-2.272177
H	-5.392328	0.626290	2.645378
H	-1.755558	-0.250833	-5.148614
H	-3.251519	-0.337553	-4.178499
H	-2.804963	-1.699406	-5.251418
H	-1.752196	-4.341499	-0.041307
H	-1.618783	-2.958351	1.053614
H	-0.861550	-4.517136	1.492306
H	-1.896170	-4.319135	-3.540174
H	3.946266	-0.735566	-3.199507
H	3.898589	-2.240505	-4.158434
H	5.458047	-1.637510	-3.518404
H	-5.451298	-0.406662	4.936215
H	-3.391626	-1.502027	5.852027
H	3.253211	2.259233	3.013291
H	3.816711	2.192201	1.320693
H	4.979759	1.961532	2.658024
H	1.246810	2.447603	5.630417
H	0.330393	3.355838	-4.405830

H	0.017764	1.663287	-4.858003
H	0.746037	2.039306	-3.269501
H	1.286966	4.573491	4.302722
H	0.523375	4.554532	1.905124
H	-5.064535	-3.979881	1.606826
H	-5.464378	-2.456510	2.451051
H	-3.783861	-2.776678	1.958567
H	-0.611325	-3.034176	-4.772017
H	0.203395	-2.825976	-3.186843
H	0.328726	-1.557855	-4.431743
H	-2.390490	2.803378	2.705114
H	-4.059720	3.176470	3.213020
H	-2.936343	4.501634	2.798559
H	-3.454890	2.959447	-4.052089
H	-2.401802	2.351970	-5.364326
H	-2.178589	4.014201	-4.730237
H	4.268141	-5.218710	1.966543
H	4.405216	-3.863883	0.813854
H	3.260358	-5.199549	0.498064
H	5.820070	-3.972882	-2.441216
H	4.233725	-4.489313	-3.068601
H	4.594276	-4.632640	-1.323792
H	2.064830	4.904100	-0.818339
H	1.200519	-5.510137	2.181278
H	0.964588	-4.323600	3.493417
H	2.360462	-5.431858	3.528714
H	-6.831910	-1.772996	-1.009371
H	-7.236071	-1.852355	0.732954
H	-6.918578	-3.353428	-0.184368
H	-4.673515	5.380350	1.005585
H	-5.767656	4.066365	1.535315
H	-5.332012	4.235098	-0.196000
H	1.747843	-0.802255	-2.403543
H	-0.195187	0.583173	-1.679816

Table S82. Cartesian geometry of 4b-Prod. (0.8 kcal/mol) in Figure S145 in Angstrom [Å].

Atomtype	X Coordinates	Y Coordinates	Z Coordinates
C	3.626297	-3.170711	-0.444090
C	2.997845	-2.357941	0.560170
C	3.800323	-1.804507	1.610204
C	5.168007	-2.133498	1.679036
C	5.770891	-2.959144	0.723861
C	5.000163	-3.457334	-0.334092
N	1.609621	-2.044592	0.478009
Si	0.337434	-2.904094	1.337157
C	1.018754	-4.342631	2.422898
C	-0.117500	-5.039628	3.207562
C	3.232739	-0.818298	2.632918
C	3.993351	0.526401	2.630106
C	2.870125	-3.690359	-1.669764
C	2.936888	-5.225011	-1.821261
Ge	1.091657	-0.559907	-0.694983
C	2.634498	0.728971	-0.710097
C	2.456124	2.120034	-0.562585
C	3.548727	3.005900	-0.678482
C	4.845329	2.517283	-0.899159
C	5.034681	1.135196	-1.039956
C	3.941176	0.257478	-0.970826
Ni	-1.090006	0.108829	-0.284509
C	-2.762347	0.655413	-1.087352

N	-3.762624	-0.048619	-1.743542
C	-4.683371	0.816701	-2.351779
C	-4.270669	2.087100	-2.085092
N	-3.108504	1.972070	-1.323815
C	-3.892575	-1.479389	-1.924340
C	-5.020441	-2.132812	-1.361746
C	-5.237685	-3.485247	-1.697861
C	-4.353691	-4.175009	-2.531118
C	-3.230300	-3.518948	-3.051881
C	-2.980536	-2.159997	-2.781816
C	-2.261320	3.094189	-1.003209
C	-2.526981	3.852142	0.162252
C	-1.696835	4.961413	0.421018
C	-0.639870	5.284876	-0.437612
C	-0.402744	4.520720	-1.590026
C	-1.215289	3.417229	-1.908837
C	-6.009399	-1.447224	-0.417010
C	-7.395466	-1.275016	-1.077062
C	-1.814216	-1.435746	-3.452378
C	-0.663979	-2.362888	-3.879905
C	-3.691326	3.513183	1.087249
C	-4.906528	4.415930	0.778879
C	-1.041225	2.668093	-3.229774
C	-1.784047	3.421581	-4.357033
P	-1.116866	-0.150260	1.906055
C	-0.494264	-1.747511	2.628067
C	-0.078297	1.170836	2.705376
C	0.514479	1.046484	3.979434
C	1.211053	2.122301	4.552424
C	1.321668	3.340843	3.861264
C	0.739513	3.473718	2.590588
C	0.053563	2.393327	2.014983
C	-2.725217	0.097206	2.833962
C	-3.914366	0.210259	2.094237
C	-5.141260	0.450726	2.735514
C	-5.193640	0.561860	4.133017
C	-4.012205	0.423819	4.885937
C	-2.787044	0.195176	4.241701
C	-6.137847	-2.215739	0.916282
C	-2.305401	-0.593835	-4.651819
C	-3.323490	3.578524	2.581298
C	0.426605	2.417123	-3.618292
C	-1.048387	-3.437909	0.111114
C	-2.456832	-3.446297	0.740081
C	-0.760837	-4.777095	-0.597380
C	3.215697	-1.407210	4.058971
C	3.378771	-3.022414	-2.968026
C	1.910961	-5.396029	1.740342
H	-1.054450	-2.639868	-0.664545
H	-5.528454	0.437114	-2.923577
H	-4.679181	3.055370	-2.371298
H	-3.856891	0.123855	1.004082
H	-1.516676	1.680445	-3.119339
H	-1.360785	-2.262097	3.078283
H	0.226350	-1.560033	3.442919
H	4.240463	-1.649925	4.398746
H	2.619401	-2.335320	4.112897
H	2.788673	-0.679542	4.774575
H	-0.832027	-5.625783	0.109412
H	0.243298	-4.809733	-1.053885

H	-1.501971	-4.957902	-1.399995
H	0.014836	6.134072	-0.209123
H	5.480538	-4.074804	-1.103939
H	2.191227	-0.604160	2.340301
H	1.653840	-3.811235	3.161178
H	-1.400846	-0.740208	-2.703219
H	-0.402819	2.499690	1.023343
H	6.841905	-3.189566	0.786666
H	0.448717	0.105287	4.536652
H	5.780301	-1.710911	2.485650
H	4.132214	-0.808179	-1.113991
H	-6.108514	-4.007219	-1.284504
H	-1.873612	0.109044	4.841395
H	-1.872191	5.565850	1.318018
H	1.812536	-3.406808	-1.540955
C	6.433473	0.574812	-1.198702
H	-3.987657	2.473654	0.871883
H	-5.624566	-0.438836	-0.186270
H	1.458622	2.530919	-0.373053
H	0.427460	4.792241	-2.248198
H	-4.533207	-5.230428	-2.772227
H	-6.054647	0.559443	2.137961
H	-1.461960	-0.027170	-5.088401
H	-3.081452	0.132814	-4.355257
H	-2.727578	-1.246414	-5.440045
H	-3.205421	-3.799317	0.009060
H	-2.770240	-2.434749	1.053904
H	-2.516176	-4.110105	1.623909
H	-2.544115	-4.073122	-3.699319
H	3.302180	-1.923238	-2.919815
H	2.781387	-3.363737	-3.835154
H	4.437495	-3.281886	-3.159161
H	-6.148991	0.755431	4.637645
H	-4.045506	0.505383	5.980232
H	3.487219	1.246955	3.297671
H	4.030511	0.964851	1.620635
H	5.034263	0.404549	2.984542
H	1.674994	2.003252	5.539983
H	0.970732	3.355671	-3.830245
H	0.469440	1.796674	-4.532637
H	0.967521	1.883987	-2.819644
H	1.867634	4.181157	4.308791
H	0.820744	4.413400	2.036756
H	-6.552944	-3.227985	0.758657
H	-6.817665	-1.681946	1.604258
H	-5.159883	-2.316980	1.415164
H	5.693441	3.204131	-0.972786
H	-0.946586	-3.010803	-4.731876
H	-0.330045	-3.004359	-3.045837
H	0.201156	-1.755853	-4.198792
H	-2.426817	2.975923	2.799022
H	-4.154477	3.177943	3.188683
H	-3.132243	4.616500	2.912375
H	-2.853188	3.560084	-4.114690
H	-1.718173	2.857325	-5.306418
H	-1.337909	4.421522	-4.516719
H	2.289554	-6.120929	2.489581
H	2.784197	-4.941593	1.245444
H	1.348293	-5.975628	0.986182
H	3.971489	-5.567200	-2.012764

H	2.317170	-5.549722	-2.678935
H	2.572215	-5.743502	-0.920463
C	3.326164	4.501858	-0.653295
H	-0.795455	-5.591091	2.528589
H	-0.734704	-4.335391	3.795111
H	0.300662	-5.781227	3.917794
H	-7.334274	-0.700953	-2.019128
H	-8.085552	-0.742827	-0.395325
H	-7.844734	-2.257827	-1.313044
H	-4.661016	5.478574	0.966885
H	-5.764266	4.142516	1.422483
H	-5.224088	4.324190	-0.275970
H	1.307324	-1.219655	-2.090710
H	-0.387599	0.487820	-1.548707
F	4.460677	5.192708	-0.395714
F	2.857593	4.954313	-1.855056
F	2.409795	4.875479	0.278164
F	6.449741	-0.542117	-1.964240
F	7.278554	1.472208	-1.768639
F	6.968699	0.240654	0.005512

Table S83. Cartesian geometry of 4c-Prod. (-0.3 kcal/mol) in Figure S145 in Angstrom [Å].

Atomtype	X Coordinates	Y Coordinates	Z Coordinates
C	-0.402620	-1.867377	3.786536
C	0.012401	-1.634078	2.458499
C	0.219261	-2.735148	1.606955
C	-0.002978	-4.045665	2.058006
C	-0.425103	-4.269854	3.377295
C	-0.620283	-3.176729	4.242192
P	0.402210	0.069177	1.802948
C	1.890232	0.413404	2.889714
C	3.158481	0.113491	2.361627
C	4.315165	0.215328	3.148325
C	4.220133	0.645150	4.481687
C	2.960967	0.971861	5.016158
C	1.803351	0.851957	4.229106
Ni	0.644620	-0.044531	-0.388622
C	2.480777	0.035228	-0.953142
N	3.264124	1.123104	-1.301264
C	4.507983	0.722224	-1.807574
C	4.521086	-0.640583	-1.794555
N	3.286598	-1.043273	-1.282627
C	2.895646	2.523489	-1.292191
C	3.589049	3.399306	-0.418347
C	3.376173	4.785497	-0.565814
C	2.481569	5.282328	-1.517925
C	1.768656	4.395466	-2.336998
C	1.965049	3.003701	-2.257959
C	2.823406	-2.409767	-1.300238
C	3.195062	-3.290754	-0.255830
C	2.691718	-4.607211	-0.302139
C	1.872351	-5.030459	-1.355250
C	1.566286	-4.154709	-2.407425
C	2.039575	-2.829869	-2.410248
C	4.548839	2.906368	0.663691
C	4.125374	3.403926	2.061747
C	1.253964	2.059086	-3.225753
C	2.187010	1.666541	-4.392962
C	4.184284	-2.889947	0.836426
C	3.685135	-3.199082	2.260503

C	1.808042	-1.914737	-3.611737
C	0.420291	-2.060839	-4.260078
Ge	-1.591424	-0.160894	-1.073420
N	-2.781562	0.797934	0.158343
Si	-2.052908	1.983734	1.225409
C	-0.908156	3.151267	0.209163
C	-1.665857	4.281073	-0.516844
C	-2.367879	-1.975818	-1.359876
C	-1.493998	-3.083002	-1.304681
C	-1.951054	-4.395862	-1.506991
C	-3.310278	-4.630408	-1.772409
C	-4.195211	-3.541082	-1.849725
C	-3.729602	-2.229666	-1.650132
C	-4.150875	0.407718	0.172881
C	-4.620588	-0.559256	1.118990
C	-5.983551	-0.914140	1.122528
C	-6.882568	-0.373241	0.195448
C	-6.412987	0.530901	-0.767350
C	-5.064203	0.931931	-0.803508
C	-3.674638	-1.260163	2.094579
C	-3.966559	-0.879408	3.560776
C	-4.602308	1.863682	-1.926607
C	-4.637953	1.142846	-3.293764
C	-3.692511	-2.795436	1.931960
C	-5.409394	3.176146	-2.012784
C	6.006756	3.311143	0.356355
C	-0.084199	2.597087	-3.759794
C	5.554064	-3.556343	0.571753
C	2.926042	-2.139455	-4.656049
C	-0.911778	1.158185	2.535406
C	-3.355086	2.907495	2.303370
C	-4.552795	3.530932	1.562928
C	-2.694716	3.959223	3.224580
C	0.273803	3.716962	1.023244
H	-0.464177	2.487873	-0.566103
H	5.249918	1.448363	-2.136601
H	5.279339	-1.359483	-2.103052
H	3.218935	-0.201987	1.315963
H	1.888185	-0.876478	-3.256257
H	-0.410691	1.970263	3.089073
H	-1.524399	0.597675	3.262215
H	-4.974772	-1.220351	3.864769
H	-3.925662	0.213323	3.717401
H	-3.230571	-1.354569	4.236368
H	-2.060368	5.024391	0.202274
H	-2.519981	3.906852	-1.109388
H	-0.988851	4.823575	-1.206452
H	1.481053	-6.055971	-1.367344
H	-7.110339	0.932375	-1.514306
H	-2.652263	-0.928166	1.853228
H	-3.758648	2.103922	2.953628
H	1.017545	1.145229	-2.655238
H	0.581899	-2.556640	0.589447
H	-7.940572	-0.666382	0.211518
H	-0.551551	-1.027522	4.476024
H	-6.342768	-1.646689	1.857195
H	-4.450500	-1.408568	-1.703445
H	3.912371	5.482098	0.089486
H	0.829434	1.093626	4.672118
H	2.952256	-5.308530	0.499028

H	-3.552262	2.123656	-1.713758
H	-5.259796	-3.709902	-2.059546
H	4.338475	-1.800599	0.766719
H	4.503324	1.804654	0.685171
H	-0.429347	-2.918648	-1.116193
H	0.939581	-4.504246	-3.234434
H	2.323000	6.364073	-1.613243
H	5.292547	-0.029814	2.712405
H	1.679847	0.945582	-5.060560
H	3.118967	1.195848	-4.034611
H	2.458423	2.557895	-4.990672
H	0.866478	4.417835	0.410014
H	0.966854	2.920390	1.346691
H	-0.059790	4.264987	1.924880
H	1.057119	4.796746	-3.065542
H	-4.031761	0.221711	-3.284734
H	-4.239585	1.802547	-4.088715
H	-5.675663	0.868926	-3.566363
H	5.121812	0.734656	5.101135
H	2.878208	1.315517	6.055615
H	-2.929091	-3.250021	2.588364
H	-3.471754	-3.089880	0.893675
H	-4.676513	-3.220092	2.208971
H	-0.949384	-3.345209	5.275742
H	0.300249	-3.036178	-4.767947
H	0.284741	-1.275245	-5.026269
H	-0.387660	-1.960356	-3.516982
H	-0.605925	-5.291852	3.734330
H	0.158009	-4.884387	1.370348
H	4.150346	4.507568	2.124738
H	4.808760	3.003614	2.830990
H	3.106789	3.062877	2.312223
H	-3.678084	-5.654336	-1.922189
H	0.056262	3.437438	-4.466693
H	-0.739587	2.934158	-2.938677
H	-0.614558	1.793258	-4.300449
H	2.716442	-2.714101	2.461455
H	4.412341	-2.826813	3.004659
H	3.563213	-4.286364	2.421445
H	3.927141	-1.970997	-4.220108
H	2.802411	-1.443760	-5.507095
H	2.892318	-3.174338	-5.046669
H	-5.252308	3.999230	2.285358
H	-5.118527	2.777877	0.991398
H	-4.229900	4.323458	0.863380
H	-6.465689	2.986399	-2.282829
H	-4.983220	3.832511	-2.795740
H	-5.398405	3.729880	-1.060232
H	-1.240444	-5.231751	-1.455185
H	-2.283310	4.803722	2.639535
H	-1.871587	3.545317	3.835976
H	-3.441464	4.385489	3.924684
H	6.337738	2.934175	-0.628634
H	6.690432	2.906324	1.126463
H	6.120148	4.411662	0.349024
H	5.473116	-4.658257	0.629104
H	6.296488	-3.228797	1.324184
H	5.941197	-3.300308	-0.432152
H	-1.889797	0.597610	-2.406260
H	0.237952	-0.503482	-1.752506

Table S84. Cartesian geometry of 4d-Prod. (0.3 kcal/mol) in Figure S145 in Angstrom [Å].

Atomtype	X Coordinates	Y Coordinates	Z Coordinates
C	4.892869	-1.492099	-0.773291
C	4.016098	-0.942520	0.222484
C	4.561491	-0.095129	1.240033
C	5.953359	0.114251	1.289657
C	6.812888	-0.454189	0.341856
C	6.274615	-1.237503	-0.688494
N	2.614686	-1.185779	0.158740
Si	1.740079	-2.355507	1.128787
C	2.912717	-3.485567	2.158362
C	2.124769	-4.520213	2.994767
C	3.671235	0.633497	2.247138
C	3.852835	2.165876	2.193730
C	4.362619	-2.292439	-1.965209
C	5.032975	-3.672405	-2.131760
Ge	1.560344	-0.024367	-1.021904
C	2.528869	1.709802	-1.161635
C	1.781978	2.898500	-1.029227
C	2.383024	4.164660	-1.113633
C	3.768074	4.292895	-1.334849
C	4.518711	3.108501	-1.493926
C	3.915397	1.843967	-1.412751
Ni	-0.689733	0.057650	-0.376247
C	-2.510187	0.218590	-0.972908
N	-3.397745	-0.750795	-1.409979
C	-4.579716	-0.182946	-1.904616
C	-4.446880	1.168996	-1.794036
N	-3.188195	1.398518	-1.236192
C	-3.181493	-2.179969	-1.495543
C	-3.983809	-3.034275	-0.696786
C	-3.915802	-4.421914	-0.938626
C	-3.057326	-4.945377	-1.909478
C	-2.236208	-4.086031	-2.653028
C	-2.285778	-2.689931	-2.478981
C	-2.580950	2.704346	-1.147451
C	-2.880516	3.544858	-0.047907
C	-2.238015	4.798813	0.009190
C	-1.352485	5.203064	-0.996765
C	-1.116905	4.375323	-2.104446
C	-1.730159	3.113665	-2.211213
C	-4.910699	-2.516978	0.402488
C	-6.395849	-2.739892	0.043915
C	-1.457178	-1.763058	-3.367126
C	-0.173672	-2.404709	-3.920430
C	-3.933009	3.178144	0.996179
C	-5.216973	4.005578	0.757022
C	-1.570499	2.265800	-3.472254
C	-2.632799	2.684920	-4.514834
P	-0.510268	-0.231954	1.804888
C	0.664032	-1.504452	2.477156
C	0.042246	1.369939	2.587345
C	0.449571	1.464853	3.934799
C	0.792534	2.708823	4.486432
C	0.732262	3.874144	3.699372
C	0.318356	3.788517	2.361361
C	-0.029889	2.543608	1.814363
C	-2.050442	-0.489021	2.842227
C	-3.267853	-0.019632	2.317918

C	-4.445757	-0.049815	3.078754
C	-4.426095	-0.576810	4.380085
C	-3.220705	-1.072353	4.908687
C	-2.039927	-1.024265	4.148854
C	-4.575570	-3.153261	1.767914
C	-2.317666	-1.191119	-4.515863
C	-3.438753	3.330838	2.446991
C	-0.160209	2.304385	-4.086345
C	0.499999	-3.321144	0.016198
C	-0.751320	-3.813613	0.772439
C	1.149469	-4.472086	-0.778335
C	3.888300	0.124411	3.687172
C	4.500698	-1.487569	-3.277883
C	4.053335	-4.182138	1.393549
H	0.143635	-2.563330	-0.716618
H	-5.387460	-0.799520	-2.296206
H	-5.116498	1.985685	-2.061611
H	-3.271627	0.370292	1.295860
H	-1.771080	1.220077	-3.194697
H	0.067010	-2.294545	2.963320
H	1.316579	-1.065735	3.251211
H	4.918848	0.337233	4.030473
H	3.732429	-0.966418	3.765228
H	3.189388	0.625739	4.382885
H	1.452440	-5.299802	-0.108725
H	2.047624	-4.149216	-1.334743
H	0.432305	-4.892732	-1.511141
H	-0.852807	6.178015	-0.927995
H	6.943621	-1.658526	-1.450475
H	2.625628	0.428924	1.966666
H	3.384783	-2.775429	2.868589
H	-1.136214	-0.922330	-2.729478
H	-0.387688	2.476199	0.781930
H	7.894874	-0.275058	0.394990
H	0.494261	0.568178	4.564705
H	6.369199	0.754317	2.078853
H	4.549835	0.960275	-1.528350
H	-4.537729	-5.100309	-0.342687
H	-1.106871	-1.398398	4.587801
H	-2.441301	5.466704	0.854316
H	3.287306	-2.456068	-1.785487
H	5.600356	3.175981	-1.676977
H	-4.200717	2.119411	0.845713
H	-4.748669	-1.430756	0.503561
H	0.701074	2.840933	-0.873380
H	-0.435924	4.711678	-2.893095
H	-3.012066	-6.028802	-2.078461
H	-5.381831	0.327850	2.646860
H	-1.721815	-0.484967	-5.123345
H	-3.200996	-0.647837	-4.137646
H	-2.670571	-2.003965	-5.179335
H	-1.401456	-4.403669	0.103273
H	-1.363628	-2.972529	1.142687
H	-0.495872	-4.455232	1.637097
H	-1.554978	-4.510591	-3.397148
H	3.990875	-0.511908	-3.213195
H	4.054839	-2.047158	-4.122841
H	5.566539	-1.303689	-3.515311
H	-5.345516	-0.610766	4.978647
H	-3.197728	-1.492622	5.922731

H	3.126462	2.652178	2.869093
H	3.687021	2.552166	1.175528
H	4.869398	2.463854	2.515137
H	1.114053	2.768978	5.534261
H	0.076192	3.293952	-4.520581
H	-0.092278	1.564321	-4.905067
H	0.614538	2.064429	-3.339825
H	1.010904	4.843827	4.131553
H	0.261046	4.685342	1.733166
H	-4.719713	-4.249408	1.751506
H	-5.230156	-2.735813	2.552942
H	-3.532562	-2.942416	2.058281
C	4.441371	5.647790	-1.377221
H	-0.388700	-3.172050	-4.688924
H	0.423502	-2.869007	-3.117208
H	0.452052	-1.627173	-4.393303
H	-2.534016	2.728967	2.628957
H	-4.220421	2.990284	3.149965
H	-3.203164	4.384117	2.687891
H	-3.656253	2.597018	-4.108010
H	-2.565292	2.041658	-5.412189
H	-2.478025	3.734838	-4.828586
H	4.682148	-4.772398	2.091257
H	4.709407	-3.456996	0.886045
H	3.663820	-4.884660	0.634267
H	6.108412	-3.575017	-2.374085
H	4.557911	-4.226678	-2.963973
H	4.946185	-4.285330	-1.220195
H	1.761630	5.064665	-1.003868
H	1.637867	-5.273499	2.346459
H	1.337919	-4.062693	3.622779
H	2.806970	-5.071406	3.673244
H	-6.661746	-2.262097	-0.916772
H	-7.050391	-2.318122	0.830081
H	-6.625521	-3.818606	-0.043861
H	-5.020291	5.085587	0.894704
H	-6.008438	3.708701	1.471228
H	-5.604341	3.863431	-0.269133
H	1.802184	-0.723106	-2.398058
H	-0.202409	0.561260	-1.697381
H	5.191496	5.702351	-2.188787
H	3.711053	6.464136	-1.526046
H	4.978756	5.851560	-0.428982

Table S85. Cartesian geometry of 4e-Prod. (0.6 kcal/mol) in Figure S145 in Angstrom [Å].

Atomtype	X Coordinates	Y Coordinates	Z Coordinates
C	4.578929	-2.159736	-0.744105
C	3.757816	-1.552325	0.265677
C	4.386094	-0.843903	1.340246
C	5.791683	-0.828495	1.427778
C	6.591599	-1.455360	0.464686
C	5.979741	-2.100214	-0.619253
N	2.339018	-1.599663	0.162375
Si	1.292328	-2.690358	1.050231
C	2.278452	-4.020250	2.035516
C	1.339039	-4.983049	2.797499
C	3.575749	-0.055998	2.369580
C	3.961220	1.438460	2.402669
C	3.977066	-2.812746	-1.990426
C	4.457944	-4.260116	-2.224629

Ge	1.480133	-0.246736	-0.975133
C	2.673737	1.342960	-1.011476
C	2.088400	2.620552	-0.853163
C	2.845686	3.795743	-0.866095
C	4.245390	3.730181	-1.038838
C	4.855597	2.470912	-1.220146
C	4.071438	1.302764	-1.208849
Ni	-0.753688	0.104029	-0.368231
C	-2.521135	0.543410	-0.983534
N	-3.522325	-0.271955	-1.484725
C	-4.604111	0.476872	-1.967640
C	-4.290429	1.790579	-1.784232
N	-3.025291	1.817013	-1.195197
C	-3.499053	-1.711074	-1.642588
C	-4.426383	-2.487973	-0.902351
C	-4.540116	-3.858101	-1.216000
C	-3.738439	-4.443471	-2.199992
C	-2.793105	-3.666880	-2.884775
C	-2.658611	-2.287463	-2.637985
C	-2.245178	3.020433	-1.034055
C	-2.442686	3.838609	0.104544
C	-1.633140	4.986188	0.232167
C	-0.685941	5.314342	-0.744948
C	-0.551153	4.518869	-1.892375
C	-1.329209	3.359946	-2.067891
C	-5.299880	-1.905406	0.207830
C	-6.792936	-1.906456	-0.185407
C	-1.695112	-1.437323	-3.464696
C	-0.493917	-2.215797	-4.027189
C	-3.553773	3.571458	1.117580
C	-4.704572	4.582928	0.909994
C	-1.263703	2.558804	-3.367315
C	-2.234324	3.172276	-4.402842
P	-0.670396	-0.320494	1.797335
C	0.304855	-1.772610	2.423395
C	0.076493	1.149611	2.671692
C	0.437282	1.127531	4.035492
C	0.934670	2.283731	4.656212
C	1.078459	3.476340	3.922423
C	0.714521	3.507390	2.567649
C	0.210632	2.351114	1.951822
C	-2.255805	-0.416798	2.792717
C	-3.381765	0.249231	2.276836
C	-4.570656	0.344299	3.014777
C	-4.657944	-0.252962	4.282626
C	-3.548219	-0.944345	4.800592
C	-2.353638	-1.021531	4.064974
C	-5.084700	-2.648751	1.542902
C	-2.447003	-0.704503	-4.598098
C	-3.068043	3.578644	2.579237
C	0.151029	2.426594	-3.958046
C	-0.038597	-3.420636	-0.134667
C	-1.358081	-3.791353	0.573613
C	0.473573	-4.596708	-0.990389
C	3.684223	-0.663088	3.783521
C	4.257584	-1.961889	-3.250220
C	3.335705	-4.822112	1.254150
H	-0.280067	-2.580450	-0.823480
H	-5.479471	-0.002365	-2.403627
H	-4.835938	2.703902	-2.019295

H	-3.304980	0.691961	1.279462
H	-1.614443	1.539907	-3.144960
H	-0.404456	-2.498865	2.855318
H	0.988822	-1.464284	3.232740
H	4.723932	-0.607648	4.159316
H	3.383280	-1.725831	3.798519
H	3.038413	-0.109582	4.490990
H	0.652649	-5.495446	-0.369475
H	1.417577	-4.361024	-1.513715
H	-0.276102	-4.876050	-1.757352
H	-0.056708	6.205251	-0.621797
H	6.605595	-2.565871	-1.391879
H	2.520429	-0.103120	2.057750
H	2.822326	-3.418363	2.792790
H	-1.281762	-0.677075	-2.780885
H	-0.108350	2.378423	0.905161
H	7.686129	-1.429061	0.548632
H	0.323978	0.208625	4.623514
H	6.268811	-0.295478	2.260714
H	4.589568	0.347726	-1.336742
H	-5.260713	-4.475133	-0.666296
H	-1.494128	-1.549029	4.496663
H	-1.753993	5.631792	1.109805
H	2.885164	-2.836912	-1.840769
H	5.937337	2.379094	-1.361218
H	-3.965162	2.570339	0.908389
H	-4.995191	-0.857698	0.368456
H	1.004794	2.705247	-0.732684
H	0.181850	4.796561	-2.656861
H	-3.835383	-5.513209	-2.425146
H	-5.432995	0.875163	2.590548
H	-1.750475	-0.055210	-5.160163
H	-3.260389	-0.068202	-4.208210
H	-2.887559	-1.432259	-5.306422
H	-2.067055	-4.244758	-0.140883
H	-1.861301	-2.902969	0.994279
H	-1.205448	-4.516585	1.395371
H	-2.158820	-4.141547	-3.640058
H	3.884570	-0.930413	-3.136005
H	3.760946	-2.405654	-4.134674
H	5.344350	-1.914037	-3.457655
H	-5.587604	-0.189582	4.862702
H	-3.610228	-1.420737	5.787839
H	3.292523	1.982219	3.093356
H	3.868786	1.895663	1.404682
H	5.001684	1.580367	2.753256
H	1.217000	2.253136	5.716567
H	0.535407	3.392847	-4.334978
H	0.131033	1.727082	-4.814177
H	0.867058	2.039862	-3.214397
H	1.477610	4.375696	4.408749
H	0.818352	4.426835	1.979514
H	-5.375892	-3.712719	1.466877
H	-5.693682	-2.185284	2.338705
H	-4.029399	-2.597242	1.859527
O	4.909950	4.924154	-1.022127
H	-0.789305	-2.911825	-4.835869
H	0.019011	-2.790924	-3.237650
H	0.238536	-1.506033	-4.450874
H	-2.259903	2.846837	2.737939

H	-3.901800	3.316542	3.255540
H	-2.690950	4.574487	2.877596
H	-3.268159	3.210897	-4.015010
H	-2.239303	2.568879	-5.329928
H	-1.927110	4.203379	-4.661971
H	3.863748	-5.525814	1.929878
H	4.094050	-4.165864	0.798070
H	2.875736	-5.426987	0.451386
H	5.542909	-4.298212	-2.439652
H	3.934902	-4.696882	-3.097060
H	4.263392	-4.904734	-1.352354
H	2.374257	4.777970	-0.741793
H	0.777148	-5.631275	2.098367
H	0.600702	-4.457996	3.431411
H	1.923456	-5.653287	3.459923
H	-6.970003	-1.349790	-1.123835
H	-7.402375	-1.439320	0.611297
H	-7.163840	-2.938299	-0.333149
H	-4.360698	5.615968	1.106599
H	-5.542041	4.364557	1.599666
H	-5.090316	4.549337	-0.126043
H	1.653554	-0.911228	-2.378532
H	-0.171605	0.608835	-1.650368
C	6.319987	4.920622	-1.167075
H	6.645286	5.973720	-1.109727
H	6.816956	4.343309	-0.358744
H	6.635032	4.498083	-2.144850

Table S86. Cartesian geometry of 4f-Prod. (1 kcal/mol) in Figure S145 in Angstrom [Å].

Atomtype	X Coordinates	Y Coordinates	Z Coordinates
C	4.215772	-2.780459	-0.736763
C	3.468901	-2.082408	0.272018
C	4.173264	-1.455591	1.350055
C	5.570485	-1.606262	1.441563
C	6.293829	-2.320977	0.479142
C	5.613393	-2.886976	-0.607899
N	2.055203	-1.960127	0.163305
Si	0.875038	-2.914880	1.037578
C	1.677346	-4.365988	2.018674
C	0.619913	-5.213478	2.763009
C	3.457893	-0.581945	2.380277
C	4.019580	0.855150	2.425879
C	3.544986	-3.355045	-1.986565
C	3.855957	-4.847392	-2.226151
Ge	1.377049	-0.502304	-0.969973
C	2.749916	0.924834	-0.981850
C	2.329958	2.256860	-0.787673
C	3.217482	3.339004	-0.767409
C	4.615375	3.135687	-0.946465
C	5.047052	1.795268	-1.166833
C	4.136558	0.731391	-1.186602
Ni	-0.802434	0.118943	-0.364250
C	-2.494302	0.779948	-0.990693
N	-3.585284	0.100844	-1.508751
C	-4.561750	0.983428	-1.990245
C	-4.088972	2.245949	-1.789652
N	-2.835185	2.108711	-1.191697
C	-3.740462	-1.328265	-1.681996
C	-4.763804	-1.990701	-0.956989
C	-5.044092	-3.333239	-1.284992

C	-4.312733	-4.005004	-2.268469
C	-3.272200	-3.345768	-2.937964
C	-2.969186	-1.996051	-2.676428
C	-1.916207	3.205112	-1.004155
C	-2.031121	4.028968	0.141845
C	-1.091683	5.069291	0.295188
C	-0.095271	5.289344	-0.663169
C	-0.037765	4.494342	-1.817362
C	-0.949053	3.441740	-2.019789
C	-5.567983	-1.314850	0.152680
C	-7.045668	-1.124950	-0.252553
C	-1.896578	-1.266785	-3.484157
C	-0.803784	-2.187736	-4.052321
C	-3.180096	3.884470	1.137452
C	-4.201188	5.026090	0.926735
C	-0.962175	2.653289	-3.328491
C	-1.845326	3.386117	-4.364922
P	-0.782366	-0.325807	1.796448
C	0.003657	-1.891508	2.415014
C	0.129027	1.035797	2.691569
C	0.487863	0.951392	4.053459
C	1.114762	2.033278	4.690604
C	1.391858	3.213381	3.975231
C	1.030545	3.307166	2.622675
C	0.397214	2.225846	1.990331
C	-2.375481	-0.236548	2.781977
C	-3.413655	0.554416	2.258754
C	-4.587681	0.788388	2.989560
C	-4.751244	0.207671	4.257470
C	-3.732528	-0.607597	4.782448
C	-2.550822	-0.824143	4.053969
C	-5.460302	-2.093077	1.480937
C	-2.535378	-0.423579	-4.610267
C	-2.717488	3.820167	2.605228
C	0.433701	2.366081	-3.908626
C	-0.528721	-3.463567	-0.161556
C	-1.891257	-3.663386	0.533659
C	-0.163016	-4.693508	-1.016443
C	3.485032	-1.208228	3.789805
C	3.924534	-2.537505	-3.242538
C	2.636141	-5.283387	1.237209
H	-0.653670	-2.598349	-0.850146
H	-5.486191	0.621504	-2.437941
H	-4.515248	3.222255	-2.017870
H	-3.280352	0.982141	1.260773
H	-1.430220	1.679179	-3.121367
H	-0.791289	-2.526641	2.841721
H	0.719329	-1.675236	3.226625
H	4.522152	-1.281385	4.169821
H	3.058490	-2.227273	3.794832
H	2.907377	-0.586432	4.499505
H	-0.105362	-5.608993	-0.396902
H	0.808241	-4.578486	-1.530259
H	-0.935021	-4.873316	-1.791065
H	0.634709	6.096673	-0.519666
H	6.182404	-3.421225	-1.380126
H	2.406538	-0.500090	2.062036
H	2.283295	-3.842871	2.787403
H	-1.392588	-0.577935	-2.785479
H	0.081369	2.307096	0.945478

H	7.383680	-2.423537	0.565744
H	0.273012	0.042013	4.627657
H	6.104509	-1.134501	2.276927
H	4.541471	-0.273115	-1.343083
H	-5.840446	-3.860728	-0.746693
H	-1.761186	-1.447668	4.490859
H	-1.148416	5.715658	1.178723
H	2.457156	-3.253866	-1.838792
H	6.106966	1.567293	-1.313746
H	-3.702620	2.940455	0.911757
H	-5.136237	-0.315225	0.327326
H	1.264774	2.470270	-0.660505
H	0.737625	4.686217	-2.566118
H	-4.539677	-5.052395	-2.504931
H	-5.380174	1.414790	2.559409
H	-1.753743	0.134711	-5.158077
H	-3.259204	0.310547	-4.215897
H	-3.063651	-1.075387	-5.332505
H	-2.646089	-4.021004	-0.188333
H	-2.280294	-2.718186	0.951891
H	-1.840028	-4.403616	1.354731
H	-2.694800	-3.889187	-3.692487
H	3.674328	-1.470081	-3.124157
H	3.380933	-2.916795	-4.129461
H	5.009844	-2.615768	-3.448426
H	-5.670533	0.379769	4.832025
H	-3.855325	-1.072269	5.769620
H	3.414533	1.471444	3.114536
H	3.992019	1.324599	1.429739
H	5.066262	0.869143	2.786531
H	1.394171	1.953390	5.749170
H	0.927684	3.285667	-4.275006
H	0.341879	1.679734	-4.770718
H	1.095373	1.896118	-3.162572
H	1.891665	4.053549	4.474393
H	1.236221	4.218070	2.048124
H	-5.881900	-3.111243	1.390198
H	-6.014100	-1.565287	2.277045
H	-4.409952	-2.177903	1.806805
N	5.514870	4.194366	-0.909247
H	-1.182469	-2.827060	-4.873094
H	-0.376279	-2.836642	-3.269079
H	0.018504	-1.574162	-4.460970
H	-2.003728	2.996439	2.766292
H	-3.585887	3.651397	3.267518
H	-2.229988	4.761667	2.919753
H	-2.872513	3.537593	-3.987245
H	-1.908360	2.798381	-5.299899
H	-1.419647	4.378483	-4.607562
H	3.066576	-6.054697	1.908469
H	3.474031	-4.719993	0.796129
H	2.113624	-5.817260	0.422368
H	4.929233	-5.009578	-2.442095
H	3.285953	-5.217917	-3.099881
H	3.588732	-5.468601	-1.356068
H	2.807075	4.340650	-0.608351
H	-0.008061	-5.785815	2.053818
H	-0.057164	-4.607148	3.392780
H	1.111755	-5.953092	3.426905
H	-7.142811	-0.541227	-1.186197

H	-7.599411	-0.593179	0.544323
H	-7.541282	-2.100735	-0.414755
H	-3.741182	6.009595	1.139535
H	-5.068132	4.900913	1.603067
H	-4.573616	5.048307	-0.114457
H	1.464225	-1.183749	-2.373162
H	-0.145077	0.553801	-1.635869
C	6.940529	3.936679	-1.012079
H	7.495675	4.887477	-0.955447
H	7.309266	3.275168	-0.197806
H	7.202726	3.451862	-1.975600
C	5.039966	5.531082	-0.605727
H	5.885634	6.238270	-0.611166
H	4.301098	5.886668	-1.355176
H	4.550998	5.589726	0.392181

Table S87. Cartesian geometry of 4g-Prod. (5.2 kcal/mol) in Figure S145 in Angstrom [Å].

Atomtype	X Coordinates	Y Coordinates	Z Coordinates
C	5.035153	0.849581	0.910892
C	4.225339	-0.037145	0.121621
C	4.794681	-1.267541	-0.342135
C	6.148322	-1.548092	-0.072030
C	6.945578	-0.667944	0.669097
C	6.378673	0.513620	1.163795
N	2.865583	0.279247	-0.164889
Si	2.281434	0.980106	-1.664489
C	3.695925	1.304128	-2.931492
C	3.150553	1.897399	-4.251473
C	3.966697	-2.319902	-1.081203
C	3.890219	-3.636358	-0.280491
C	4.487711	2.147474	1.509668
C	5.255671	3.400034	1.035457
Ge	1.565909	-0.155233	1.248591
N	2.098483	-1.754968	2.068886
Ni	-0.601950	-0.040496	0.404112
C	-2.394974	0.578478	0.781419
N	-2.983257	1.835742	0.754735
C	-4.252850	1.823128	1.349838
C	-4.483831	0.547072	1.766269
N	-3.353018	-0.190627	1.418066
C	-2.422304	3.086799	0.290767
C	-3.030557	3.730028	-0.818392
C	-2.605104	5.035409	-1.141003
C	-1.593246	5.666894	-0.413062
C	-0.983460	5.000169	0.658346
C	-1.388400	3.709343	1.047373
C	-3.125664	-1.544065	1.856770
C	-3.645106	-2.615602	1.090409
C	-3.423018	-3.923354	1.567269
C	-2.699761	-4.147112	2.746033
C	-2.209994	-3.065786	3.493816
C	-2.424820	-1.738982	3.077295
C	-4.120142	3.079733	-1.672566
C	-5.481082	3.786442	-1.487544
C	-0.782905	3.040611	2.280211
C	0.635618	3.526032	2.623676
C	-4.461548	-2.369291	-0.175947
C	-5.971561	-2.474575	0.135676
C	-2.021949	-0.557943	3.960586
C	-3.118709	-0.311166	5.022310

P	-0.251775	-0.927846	-1.583559
C	1.143929	-0.253127	-2.609749
C	0.064380	-2.759568	-1.472314
C	0.869441	-3.490944	-2.369373
C	0.985554	-4.885769	-2.250831
C	0.297788	-5.570871	-1.234748
C	-0.509037	-4.850877	-0.338362
C	-0.621648	-3.458332	-0.459076
C	-1.668802	-0.962452	-2.809475
C	-2.851894	-0.274022	-2.493522
C	-3.964158	-0.325061	-3.350589
C	-3.896905	-1.056970	-4.545762
C	-2.708579	-1.733394	-4.881858
C	-1.603006	-1.688718	-4.019078
C	-3.722544	3.050365	-3.164290
C	-1.717460	3.184551	3.502331
C	-4.078830	-3.292289	-1.347853
C	-0.641312	-0.703793	4.625130
C	1.195536	2.526348	-1.292431
C	0.063551	2.752772	-2.315091
C	2.018815	3.814721	-1.094497
C	4.487950	-2.591375	-2.507343
C	4.479208	2.101022	3.053968
C	4.913714	2.115871	-2.451349
H	0.702676	2.285808	-0.323862
H	-4.853549	2.727917	1.427214
H	-5.330117	0.097188	2.283761
H	-2.887143	0.288968	-1.554497
H	-1.974118	0.336232	3.319474
H	0.691594	0.260361	-3.475871
H	1.771039	-1.065250	-3.016227
H	5.514111	-3.005101	-2.487188
H	4.513663	-1.671889	-3.119212
H	3.843889	-3.330479	-3.021203
H	2.471218	4.149751	-2.047383
H	2.837821	3.686109	-0.366332
H	1.372779	4.638073	-0.733054
H	-2.520902	-5.173676	3.091115
H	6.996158	1.196763	1.761801
H	2.937595	-1.933681	-1.155947
H	4.057930	0.279492	-3.154388
H	-0.701425	1.966215	2.046263
H	-1.251592	-2.899285	0.240452
H	7.998160	-0.906357	0.870849
H	1.422254	-2.983263	-3.167347
H	6.583002	-2.487203	-0.439230
H	-3.068009	5.556464	-1.987226
H	-0.689976	-2.237356	-4.281340
H	-3.808641	-4.775833	0.996680
H	3.443011	2.242361	1.170930
H	-4.259770	-1.334752	-0.500353
H	-4.240693	2.034517	-1.339005
H	-1.659067	-3.256375	4.421341
H	-1.268809	6.679454	-0.684743
H	-4.886119	0.203093	-3.077037
H	-1.294225	2.643394	4.369106
H	-2.721432	2.770591	3.304324
H	-1.832789	4.249160	3.783154
H	-0.499556	3.671724	-2.073886
H	-0.664145	1.921682	-2.309017

H	0.448412	2.860139	-3.347455
H	-0.186819	5.503301	1.214829
H	3.929768	1.221095	3.428682
H	3.992838	3.007365	3.463572
H	5.510125	2.059631	3.455547
H	-4.765218	-1.103930	-5.215706
H	-2.648445	-2.306344	-5.816496
H	3.228017	-4.358681	-0.791585
H	3.478389	-3.443184	0.721221
H	4.888110	-4.102370	-0.171077
H	1.625565	-5.436708	-2.951955
H	-0.617332	-1.531986	5.357640
H	-0.395689	0.224409	5.173784
H	0.151087	-0.876273	3.878604
H	0.396073	-6.659930	-1.140090
H	-1.051139	-5.364767	0.464741
H	-3.616836	4.072068	-3.572951
H	-4.496712	2.532642	-3.758511
H	-2.768479	2.518027	-3.312398
H	0.639639	4.564947	3.005997
H	1.304213	3.472156	1.747412
H	1.067251	2.881256	3.408816
H	-3.001413	-3.232901	-1.571327
H	-4.625274	-2.985440	-2.257477
H	-4.333628	-4.348569	-1.140717
H	-4.105306	-0.145907	4.552700
H	-2.873933	0.581175	5.628775
H	-3.206204	-1.179230	5.703136
H	5.684316	2.159212	-3.248129
H	5.382514	1.673119	-1.557508
H	4.635742	3.159262	-2.215089
H	6.304445	3.387669	1.388232
H	4.780277	4.314652	1.439240
H	5.272111	3.479480	-0.063124
H	2.770361	2.926075	-4.103104
H	2.330118	1.299365	-4.689160
H	3.955296	1.957680	-5.011987
H	-5.802234	3.788384	-0.430298
H	-6.264523	3.280661	-2.083145
H	-5.427827	4.839026	-1.823409
H	-6.229687	-3.496361	0.473611
H	-6.570120	-2.250495	-0.767690
H	-6.272581	-1.769922	0.932354
H	2.004223	0.900352	2.308710
H	-0.288987	0.089485	1.856384
C	3.268628	-1.794923	2.928325
C	1.174023	-2.845354	2.293735
H	3.797973	-2.770319	2.843012
H	3.999398	-1.017480	2.649257
H	3.011315	-1.660303	4.009222
H	1.531919	-3.811216	1.866559
H	0.991537	-3.020749	3.381040
H	0.195064	-2.624551	1.840823

Table S88. Cartesian geometry of PhSiH3 in Figure S146 in Angstrom [Å].

Atomtype	X Coordinates	Y Coordinates	Z Coordinates
Si	0.007559	2.363825	0.000000
H	-1.405352	2.858756	0.000000
H	0.715764	2.894998	1.210047
C	0.000000	0.471169	0.000000

H	0.715764	2.894998	-1.210047
C	1.212521	-0.260754	0.000000
C	1.211043	-1.664842	0.000000
C	-0.007082	-2.367379	0.000000
C	-1.220269	-1.659728	0.000000
C	-1.215079	-0.253772	0.000000
H	2.176687	0.268197	0.000000
H	2.162102	-2.213427	0.000000
H	-0.009289	-3.465195	0.000000
H	-2.174300	-2.203092	0.000000
H	-2.174012	0.283041	0.000000

Table S89. Cartesian geometry of 3a-INT1 (14.7 kcal/mol) in Figure S146 in Angstrom [Å].

Atomtype	X Coordinates	Y Coordinates	Z Coordinates
C	5.161869	0.028103	0.326793
C	4.007733	0.043775	1.174306
C	4.078663	0.735564	2.432839
C	5.298406	1.314015	2.832961
C	6.438089	1.260044	2.022280
C	6.353025	0.633613	0.775162
N	2.775120	-0.587119	0.773556
Si	2.348379	-2.222055	1.431866
C	3.614333	-2.657089	2.817276
C	4.928982	-3.272993	2.297839
C	2.856322	0.963341	3.328199
C	3.012547	0.419796	4.764782
C	5.160728	-0.583718	-1.074134
C	6.182202	-1.734311	-1.210439
Ge	1.428944	0.320514	-0.253913
Ni	-0.792657	-0.004436	-0.089751
P	-0.921489	-1.808051	1.260783
C	-1.306447	-3.574837	0.784540
C	-1.740954	-4.512592	1.749730
C	-1.948406	-5.855078	1.400238
C	-1.712764	-6.282191	0.081078
C	-1.287068	-5.356056	-0.883115
C	-1.097759	-4.006606	-0.537919
C	2.420605	1.945889	-0.988078
C	3.359633	2.761829	-0.311730
C	3.970534	3.855054	-0.939799
C	3.667145	4.160899	-2.287936
C	2.720000	3.367883	-2.979285
C	2.103815	2.295208	-2.323901
C	2.224850	-3.598396	0.097604
C	1.836286	-4.941881	0.757633
C	0.624477	-2.094447	2.277635
C	3.417035	-3.823530	-0.849364
Si	-0.731750	1.651875	1.766602
C	-2.071394	2.629901	2.703653
C	-1.887751	3.989374	3.041492
C	-2.849187	4.687403	3.792683
C	-4.017491	4.034472	4.221992
C	-4.212990	2.679808	3.901389
C	-3.248126	1.989387	3.150756
C	-1.776552	0.403805	-1.722221
N	-1.796022	-0.205374	-2.972532
C	-2.430883	0.577750	-3.940823
C	-2.849468	1.707463	-3.308785
N	-2.462504	1.590608	-1.973103
C	-1.481386	-1.576159	-3.282052

C	-2.536373	-2.522496	-3.243633
C	-2.266433	-3.820321	-3.718734
C	-0.992556	-4.168495	-4.186269
C	0.043028	-3.222735	-4.185990
C	-0.187241	-1.902944	-3.751947
C	-2.914555	2.576746	-1.017394
C	-2.176093	3.778558	-0.865581
C	-2.753311	4.801060	-0.088049
C	-3.994787	4.624020	0.533327
C	-4.672804	3.405069	0.419716
C	-4.152773	2.352595	-0.358164
C	-3.929892	-2.161242	-2.734429
C	-4.394170	-3.093289	-1.596853
C	0.889949	-0.830997	-3.834046
C	0.895003	-0.145676	-5.216959
C	-0.798243	3.953892	-1.505523
C	-0.885107	4.479937	-2.956260
C	-4.895814	1.018869	-0.486051
C	-5.738564	0.945624	-1.780703
C	-4.949712	-2.136821	-3.892449
C	2.290033	-1.341512	-3.466718
C	-2.217941	-1.597757	2.586731
C	-3.573232	-1.668650	2.186440
C	-4.608270	-1.525620	3.121135
C	-4.312540	-1.272114	4.473186
C	-2.972883	-1.171391	4.877414
C	-1.933708	-1.343719	3.945163
C	2.488385	2.463839	3.391778
C	5.429991	0.469251	-2.172998
C	3.022053	-3.507382	3.961760
C	0.138536	4.871698	-0.695309
C	-5.804471	0.702582	0.715862
H	-1.734641	0.966123	0.715995
H	-2.542957	0.240629	-4.970590
H	-3.405056	2.571470	-3.670421
H	0.465824	-2.978641	2.922356
H	0.713251	-1.220322	2.947992
H	3.886704	-1.676020	3.243849
H	1.369465	-3.266618	-0.525131
H	0.232704	2.698972	1.322848
H	-0.101263	0.832043	2.845830
H	-0.984380	4.517660	2.706630
H	-2.688451	5.745500	4.039798
H	-4.770581	4.578887	4.807218
H	-5.118997	2.156123	4.234833
H	-3.413191	0.932556	2.914569
H	-2.212974	5.742902	0.052657
H	-4.417267	5.426059	1.149839
H	-5.615688	3.269578	0.957930
H	-3.063935	-4.572558	-3.704899
H	-0.801280	-5.187668	-4.546467
H	1.037589	-3.507426	-4.547655
H	-3.818302	-1.842313	1.131086
H	-5.651612	-1.597743	2.790855
H	-5.122402	-1.145543	5.202783
H	-2.728629	-0.960438	5.926468
H	-0.897921	-1.276833	4.293376
H	-1.917621	-4.189943	2.783593
H	-2.288408	-6.571064	2.160040
H	-1.863735	-7.334371	-0.193219

H	-1.104873	-5.677142	-1.914173
H	-0.786874	-3.283267	-1.299970
H	3.798212	-3.716514	4.725421
H	2.188129	-2.995328	4.475815
H	2.644384	-4.483757	3.603631
H	5.660050	-3.373921	3.125046
H	4.774364	-4.283260	1.875619
H	5.397155	-2.641445	1.523335
H	3.144942	-4.560836	-1.631647
H	3.735972	-2.905011	-1.361201
H	4.295258	-4.225704	-0.315473
H	1.578276	-5.690066	-0.016409
H	2.681171	-5.351325	1.343158
H	0.962840	-4.863369	1.425947
H	0.613751	-0.075235	-3.078916
H	2.989003	-0.492281	-3.373654
H	2.266254	-1.877326	-2.503923
H	2.701167	-2.026824	-4.231330
H	1.656030	0.657368	-5.244355
H	1.138704	-0.875603	-6.012242
H	-0.085978	0.303851	-5.451593
H	-3.881135	-1.143847	-2.315438
H	-5.948919	-1.840745	-3.522668
H	-4.647618	-1.417409	-4.675345
H	-5.041029	-3.134701	-4.361794
H	-5.350717	-2.729192	-1.176583
H	-4.557514	-4.127181	-1.954167
H	-3.645617	-3.135676	-0.788283
H	5.349065	1.834169	3.798073
H	7.379410	1.717296	2.353134
H	7.235231	0.616401	0.122863
H	2.006773	0.442252	2.863196
H	4.152727	-0.993123	-1.245669
H	5.391795	-0.006041	-3.171617
H	4.691686	1.285002	-2.150339
H	6.434785	0.917303	-2.058903
H	6.091442	-2.213920	-2.203326
H	7.217712	-1.357445	-1.114577
H	6.033601	-2.512622	-0.444787
H	2.076891	0.584588	5.332267
H	3.234586	-0.660308	4.785601
H	3.824190	0.941236	5.306426
H	1.564725	2.608747	3.982358
H	3.295668	3.049478	3.870571
H	2.309002	2.885455	2.389134
H	1.354701	1.719352	-2.879955
H	2.471827	3.602920	-4.020993
C	4.303235	5.268132	-2.945997
H	4.697883	4.468695	-0.395399
H	3.650436	2.538412	0.717250
H	-4.117983	0.231772	-0.528840
H	-0.338825	2.948320	-1.530584
H	1.170420	4.773053	-1.072884
H	-0.150471	5.935163	-0.794064
H	0.140536	4.612613	0.376464
H	0.131528	4.625918	-3.365741
H	-1.420600	3.785410	-3.623800
H	-1.405686	5.456278	-2.980494
H	-6.164111	-0.340080	0.642803
H	-5.275328	0.817010	1.673999

H	-6.698298	1.354474	0.731672
H	-6.266186	-0.024813	-1.837139
H	-6.499420	1.749201	-1.785960
H	-5.124842	1.044217	-2.689633
N	4.818667	6.171906	-3.484859

Table S90. Cartesian geometry of 3b-INT1 (12.3 kcal/mol) in Figure S146 in Angstrom [Å].

Atomtype	X Coordinates	Y Coordinates	Z Coordinates
C	4.331257	2.250248	-0.470278
C	3.167315	1.999125	-1.265836
C	3.331478	1.693413	-2.660875
C	4.618497	1.744219	-3.229568
C	5.749518	2.041100	-2.461230
C	5.596352	2.269503	-1.090707
N	1.848006	2.002451	-0.686448
Si	0.784217	3.457763	-0.865075
C	1.640658	4.679383	-2.084233
C	2.698016	5.586073	-1.423541
C	2.180988	1.202685	-3.544683
C	1.870465	2.117968	-4.747888
C	4.278692	2.452700	1.044280
C	4.829978	3.829976	1.473135
Ge	1.029263	0.424637	0.031537
Ni	-1.149433	-0.107487	-0.001039
P	-2.073085	1.784596	-0.819778
C	-3.023919	3.081331	0.133134
C	-3.872079	4.000915	-0.525498
C	-4.516182	5.019720	0.192237
C	-4.312778	5.139222	1.578697
C	-3.477738	4.225391	2.239835
C	-2.846346	3.193623	1.524251
C	2.618491	-0.826079	0.288422
C	3.754171	-0.996091	-0.531930
C	4.788892	-1.876320	-0.166245
C	4.723948	-2.606655	1.030150
C	3.588967	-2.464915	1.845098
C	2.551438	-1.598247	1.467081
C	0.314855	4.284120	0.803944
C	-0.612367	5.496193	0.553765
C	-0.851262	2.894229	-1.708374
C	1.441887	4.694536	1.769173
Si	-0.706992	-1.087609	-2.267799
C	-1.656115	-2.262285	-3.425489
C	-1.015568	-3.358514	-4.044072
C	-1.703589	-4.182906	-4.951641
C	-3.051479	-3.926161	-5.254544
C	-3.702585	-2.834251	-4.653192
C	-3.008345	-2.012159	-3.751661
C	-1.611808	-1.288684	1.482705
N	-1.638980	-1.070565	2.856631
C	-1.697957	-2.260796	3.586820
C	-1.733990	-3.271423	2.676365
N	-1.699642	-2.679106	1.412497
C	-1.852869	0.183920	3.532876
C	-3.189505	0.605690	3.750583
C	-3.394010	1.754282	4.538690
C	-2.313612	2.463460	5.079868
C	-1.001128	2.029406	4.849106
C	-0.747649	0.870614	4.089528
C	-1.895802	-3.494245	0.235339

C	-0.838854	-4.328737	-0.208157
C	-1.117419	-5.245106	-1.242323
C	-2.384275	-5.312465	-1.828103
C	-3.402473	-4.451866	-1.399522
C	-3.189774	-3.533164	-0.355513
C	-4.382857	-0.162736	3.191477
C	-5.246608	0.708308	2.258830
C	0.663793	0.326201	3.934355
C	1.117915	-0.423368	5.204810
C	0.552758	-4.300056	0.422474
C	0.772983	-5.520431	1.344759
C	-4.327035	-2.647959	0.158747
C	-4.954715	-3.248704	1.438401
C	-5.221086	-0.783613	4.328343
C	1.681835	1.401156	3.528676
C	-3.326794	1.454914	-2.164696
C	-4.553548	0.866986	-1.777490
C	-5.550541	0.589153	-2.723330
C	-5.331172	0.863547	-4.085636
C	-4.108044	1.422266	-4.486416
C	-3.117911	1.724689	-3.534079
C	2.443166	-0.234725	-4.052078
C	5.032394	1.339868	1.808120
C	0.653998	5.511822	-2.931311
C	1.677178	-4.227074	-0.630648
C	-5.437401	-2.395219	-0.875659
H	-1.734227	-1.145972	-1.040750
H	-1.729789	-2.266509	4.675701
H	-1.805767	-4.350575	2.801204
H	-1.392884	3.783299	-2.080933
H	-0.514489	2.328446	-2.596329
H	2.194250	4.018951	-2.775555
H	-0.282371	3.500038	1.312300
H	0.644109	-1.711359	-2.158175
H	-0.605377	0.164793	-3.076476
H	0.034024	-3.581113	-3.807845
H	-1.187638	-5.032774	-5.418553
H	-3.591109	-4.571297	-5.960671
H	-4.753832	-2.618590	-4.887298
H	-3.528061	-1.158624	-3.302090
H	-0.321568	-5.909228	-1.598525
H	-2.575666	-6.017524	-2.645228
H	-4.377335	-4.497840	-1.893382
H	-4.417601	2.103060	4.719766
H	-2.495038	3.359763	5.687145
H	-0.160233	2.581921	5.284657
H	-4.729977	0.624899	-0.722499
H	-6.497255	0.141112	-2.397305
H	-6.105335	0.633736	-4.828678
H	-3.918499	1.628992	-5.547606
H	-2.179844	2.173844	-3.875801
H	-4.029008	3.920273	-1.608624
H	-5.173675	5.725613	-0.332156
H	-4.807411	5.941637	2.141379
H	-3.316269	4.306626	3.320066
H	-2.213817	2.468210	2.048843
H	1.208875	6.186939	-3.613603
H	0.000849	4.878016	-3.558773
H	-0.000437	6.146356	-2.304248
H	3.243359	6.166950	-2.194303

H	2.241447	6.315476	-0.728973
H	3.447137	4.998061	-0.866374
H	1.009123	5.046827	2.727438
H	2.126735	3.867829	2.003978
H	2.049152	5.522078	1.363358
H	-1.037254	5.859220	1.509403
H	-0.049562	6.335687	0.103077
H	-1.465981	5.263124	-0.104329
H	0.616985	-0.404663	3.111738
H	2.649033	0.932854	3.277927
H	1.324423	1.954914	2.644533
H	1.864145	2.133030	4.337353
H	2.128469	-0.847202	5.062697
H	1.147757	0.263956	6.071671
H	0.432380	-1.254208	5.449907
H	-3.991201	-0.990342	2.582472
H	-6.055929	-1.378058	3.912927
H	-4.604881	-1.451154	4.958583
H	-5.653536	-0.001712	4.980866
H	-6.044230	0.096304	1.796979
H	-5.730257	1.539906	2.804431
H	-4.631263	1.149953	1.458078
H	4.737225	1.519331	-4.297073
H	6.745282	2.064164	-2.921406
H	6.485973	2.457038	-0.476847
H	1.279126	1.165041	-2.916584
H	3.219469	2.398687	1.338755
H	4.988338	1.528796	2.897418
H	4.601467	0.345878	1.615447
H	6.098652	1.304369	1.517765
H	4.694464	3.976223	2.561402
H	5.911854	3.909166	1.256634
H	4.319559	4.658425	0.956033
H	1.028973	1.699213	-5.332058
H	1.590788	3.138675	-4.438430
H	2.741640	2.196903	-5.425475
H	1.558509	-0.616073	-4.595106
H	3.305478	-0.262813	-4.744105
H	2.653357	-0.932726	-3.224599
H	1.672812	-1.532681	2.117086
C	3.502456	-3.214460	3.156508
H	5.533539	-3.287246	1.311644
C	6.026134	-1.956047	-1.038652
H	3.865729	-0.430860	-1.459160
H	-3.877824	-1.667119	0.408059
H	0.620330	-3.389884	1.042421
H	2.654663	-4.110590	-0.131903
H	1.724730	-5.147908	-1.241053
H	1.537302	-3.369333	-1.308483
H	1.777193	-5.483770	1.803475
H	0.030140	-5.559895	2.160677
H	0.692115	-6.460656	0.766898
H	-6.128023	-1.620602	-0.495009
H	-5.030115	-2.048457	-1.837660
H	-6.038605	-3.305735	-1.059654
H	-5.770895	-2.600000	1.806322
H	-5.382706	-4.245214	1.218405
H	-4.221158	-3.362793	2.253027
F	4.073404	-4.442667	3.079145
F	4.138297	-2.544943	4.157138

F	2.217141	-3.389796	3.560950
F	6.698343	-3.121372	-0.865137
F	5.722508	-1.850740	-2.354446
F	6.894357	-0.950455	-0.749330

Table S91. Cartesian geometry of 3c-INT1 (13.9 kcal/mol) in Figure S146 in Angstrom [Å].

Atomtype	X Coordinates	Y Coordinates	Z Coordinates
C	2.871208	-3.117915	1.529538
C	2.047971	-2.010880	1.846951
C	1.608121	-1.894439	3.188211
C	2.004744	-2.808181	4.179118
C	2.832100	-3.892553	3.841849
C	3.251204	-4.050355	2.510167
Ge	1.397638	-0.557149	0.588265
N	2.932006	-0.196923	-0.517938
Si	2.831345	1.213907	-1.649355
C	2.874728	2.926513	-0.778738
C	4.033491	3.240554	0.184358
Ni	-0.721687	0.045532	0.097199
Si	-0.841272	-2.062682	-1.193314
C	-2.281858	-3.064056	-1.941421
C	-2.318927	-4.474141	-1.862054
C	-3.347425	-5.210097	-2.475840
C	-4.362693	-4.545371	-3.185078
C	-4.337466	-3.143141	-3.281064
C	-3.307186	-2.415406	-2.664559
C	-1.844940	0.299808	1.669466
N	-1.823455	1.248096	2.687683
C	-2.637916	0.895091	3.767477
C	-3.215876	-0.292615	3.441848
N	-2.744269	-0.637904	2.174757
C	-1.289326	2.583776	2.627099
C	-2.157572	3.626005	2.214440
C	-1.688942	4.949033	2.327476
C	-0.399833	5.220898	2.804090
C	0.451179	4.170816	3.178340
C	0.014917	2.833230	3.115925
C	-3.310811	-1.782909	1.498343
C	-2.784473	-3.072353	1.768191
C	-3.488986	-4.180258	1.259015
C	-4.647890	-4.010925	0.492879
C	-5.109853	-2.725618	0.187806
C	-4.455754	-1.580625	0.682350
C	-3.564650	3.346598	1.690969
C	-4.630478	3.814352	2.704438
C	0.878127	1.682202	3.611667
C	2.364261	1.835733	3.260916
P	-0.463287	1.368452	-1.709302
C	1.161891	1.104510	-2.600457
C	-1.702282	0.974256	-3.048584
C	-3.044394	1.363143	-2.825744
C	-4.037056	1.105486	-3.781541
C	-3.717602	0.419872	-4.968101
C	-2.395810	0.004288	-5.188029
C	-1.393911	0.288547	-4.242437
C	-0.560487	3.234933	-1.799066
C	-0.771624	3.897894	-3.030259
C	-0.761385	5.298842	-3.099954
C	-0.527793	6.058465	-1.939572
C	-0.322893	5.407710	-0.713553

C	-0.352430	4.004263	-0.639948
C	4.058860	-1.091257	-0.601814
C	4.076800	-2.124163	-1.601865
C	5.202390	-2.963222	-1.706274
C	6.294060	-2.838638	-0.839223
C	6.251307	-1.870266	0.168536
C	5.155647	-0.995788	0.313739
C	2.878087	-2.424925	-2.507304
C	2.272218	-3.810047	-2.183085
C	5.184538	-0.008993	1.480931
C	5.209223	-0.724994	2.850124
C	4.228975	1.031649	-2.965083
C	3.856597	1.588472	-4.356061
C	5.598570	1.570769	-2.506552
C	2.762338	4.057831	-1.826901
C	-3.805201	3.965547	0.299089
C	0.687261	1.450093	5.125499
C	3.188560	-2.349259	-4.017658
C	6.378436	0.967072	1.393837
H	-1.778295	-0.958379	-0.487393
H	-2.742690	1.535384	4.642479
H	-3.933492	-0.914765	3.974678
H	1.195900	1.770437	-3.482458
H	1.132904	0.067367	-2.981079
H	4.352639	-0.060343	-3.070648
H	1.942280	2.924895	-0.178899
H	-0.091546	-3.069334	-0.387092
H	-0.034055	-1.709791	-2.402072
H	-1.537816	-5.009358	-1.304556
H	-3.359328	-6.305668	-2.396565
H	-5.167974	-5.118617	-3.663712
H	-5.121643	-2.610933	-3.836256
H	-3.299817	-1.323609	-2.753373
C	-1.490974	-3.253933	2.562958
H	-3.111726	-5.191256	1.444654
H	-5.170104	-4.886544	0.089718
H	-5.985806	-2.615693	-0.458510
C	-4.959765	-0.172724	0.348783
H	-2.339515	5.776070	2.019440
H	-0.050932	6.259194	2.877253
H	1.459915	4.393935	3.544075
H	-3.311454	1.876484	-1.893415
H	-5.068407	1.427832	-3.593582
H	-4.496971	0.204288	-5.709996
H	-2.135892	-0.543424	-6.103035
H	-0.368086	-0.033326	-4.449154
H	-0.944215	3.313267	-3.942726
H	-0.929349	5.799063	-4.062885
H	-0.508161	7.154833	-1.992598
H	-0.143707	5.989433	0.196854
H	-0.213762	3.501703	0.323751
H	4.697961	1.446366	-5.064235
H	2.976021	1.079640	-4.789064
H	3.634259	2.671910	-4.325753
H	6.381110	1.317336	-3.249918
H	5.594885	2.671455	-2.400613
H	5.908131	1.130668	-1.543129
H	3.847951	4.205599	0.698300
H	4.162619	2.474483	0.961345
H	4.995630	3.335751	-0.348043

H	2.595214	5.031083	-1.326379
H	3.696719	4.144683	-2.413307
H	1.927096	3.917216	-2.532757
H	0.512736	0.786362	3.081400
H	2.902620	0.898657	3.485306
H	2.486936	2.056352	2.188242
H	2.850499	2.645773	3.836279
H	1.288877	0.582801	5.457842
H	1.015774	2.337041	5.700286
H	-0.369552	1.249806	5.375707
H	-3.670066	2.255949	1.579330
H	-5.645963	3.577839	2.336035
H	-4.496941	3.316953	3.682406
H	-4.571297	4.907534	2.865627
H	-4.789246	3.644313	-0.091733
H	-3.804306	5.070999	0.335501
H	-3.023500	3.655846	-0.414250
H	5.213466	-3.744509	-2.477168
H	7.162671	-3.502485	-0.937916
H	7.090408	-1.790363	0.871253
H	2.105038	-1.674512	-2.286945
H	4.253247	0.576821	1.429823
H	5.199195	0.020734	3.667994
H	4.344572	-1.393138	2.979270
H	6.127994	-1.330366	2.965806
H	6.316721	1.723194	2.199313
H	7.338346	0.430143	1.512531
H	6.407495	1.501796	0.430802
H	2.267065	-2.539680	-4.600233
H	3.580934	-1.363551	-4.318987
H	3.931910	-3.113154	-4.314649
H	1.363228	-3.983126	-2.788785
H	2.992472	-4.619269	-2.408061
H	1.984651	-3.893665	-1.122230
H	0.938162	-1.075943	3.478283
H	1.661021	-2.675621	5.213625
H	3.142452	-4.612579	4.610280
H	3.891978	-4.897152	2.230893
H	3.249346	-3.255295	0.513818
C	-5.737760	-0.095924	-0.977565
C	-5.840389	0.412697	1.477227
H	-4.059079	0.462714	0.241436
C	-0.687360	-4.501465	2.146427
H	-0.865435	-2.369218	2.343543
C	-1.737129	-3.301103	4.087954
H	0.327224	-4.447358	2.576254
H	-1.160043	-5.430743	2.518230
H	-0.590671	-4.578492	1.050794
H	-0.779401	-3.461488	4.616377
H	-2.180741	-2.367737	4.471234
H	-2.415210	-4.138322	4.342441
H	-5.911120	0.962886	-1.243542
H	-5.190309	-0.572731	-1.804665
H	-6.730646	-0.577115	-0.894160
H	-6.193152	1.423501	1.199258
H	-6.728922	-0.227201	1.638045
H	-5.299067	0.500069	2.432150

Table S92. Cartesian geometry of 3d-INT1 (13.0 kcal/mol) in Figure S146 in Angstrom [Å].

Atomtype	X Coordinates	Y Coordinates	Z Coordinates

C	-5.177642	-0.488168	0.129648
C	-4.044982	-0.499846	1.005814
C	-4.093405	-1.311400	2.191674
C	-5.276925	-2.007128	2.503502
C	-6.400236	-1.955751	1.670131
C	-6.332088	-1.213049	0.487358
N	-2.855729	0.255751	0.704554
Si	-2.575947	1.844375	1.526995
C	-3.912873	2.052980	2.900410
C	-5.256031	2.611964	2.390587
C	-2.875886	-1.538521	3.093350
C	-3.094867	-1.138585	4.568219
C	-5.186966	0.245068	-1.211624
C	-6.287908	1.326189	-1.281382
Ge	-1.419457	-0.452831	-0.364823
Ni	0.766005	0.035937	-0.085895
P	0.718113	1.698865	1.436115
C	0.978302	3.529480	1.149946
C	1.305529	4.398005	2.216785
C	1.418569	5.780107	2.005509
C	1.194259	6.315766	0.724515
C	0.874680	5.459324	-0.340000
C	0.780317	4.072228	-0.132573
C	-2.247777	-2.056965	-1.280503
C	-3.140345	-3.004303	-0.724829
C	-3.635190	-4.076469	-1.483914
C	-3.277585	-4.244753	-2.837476
C	-2.385071	-3.308847	-3.399010
C	-1.870100	-2.253305	-2.630686
C	-2.521995	3.349276	0.332553
C	-2.257978	4.649880	1.125959
C	-0.873794	1.768811	2.420100
C	-3.701847	3.571422	-0.630105
Si	0.780961	-1.784668	1.592476
C	2.167272	-2.754893	2.472480
C	2.078209	-4.151291	2.668176
C	3.069819	-4.850940	3.377601
C	4.174608	-4.162578	3.907933
C	4.275549	-2.771764	3.730023
C	3.280963	-2.080413	3.020066
C	1.813385	-0.132152	-1.720702
N	1.813050	0.593168	-2.908598
C	2.533920	-0.040298	-3.925058
C	3.027924	-1.188730	-3.388710
N	2.600277	-1.231823	-2.061135
C	1.398517	1.959021	-3.096986
C	2.375345	2.974578	-2.941615
C	2.012036	4.289074	-3.292710
C	0.722345	4.583525	-3.753777
C	-0.234806	3.565300	-3.874057
C	0.091606	2.230224	-3.567278
C	3.107184	-2.267728	-1.189857
C	2.465520	-3.532867	-1.177658
C	3.104616	-4.577749	-0.482732
C	4.312136	-4.365566	0.192297
C	4.891559	-3.091845	0.217300
C	4.306417	-2.012566	-0.472647
C	3.783800	2.672205	-2.434883
C	4.137416	3.494342	-1.179257
C	-0.892858	1.090269	-3.783584

C	-0.796001	0.529375	-5.218289
C	1.122577	-3.751342	-1.875123
C	1.286676	-4.129486	-3.364578
C	4.939744	-0.617747	-0.448159
C	5.808301	-0.352731	-1.699940
C	4.828393	2.872798	-3.552727
C	-2.341748	1.454822	-3.433983
C	1.986690	1.457292	2.784984
C	3.343939	1.672275	2.447651
C	4.358050	1.511147	3.402153
C	4.042200	1.096117	4.709005
C	2.702850	0.852988	5.048422
C	1.682496	1.042436	4.098894
C	-2.407086	-3.010656	3.030560
C	-5.345565	-0.723253	-2.405580
C	-3.424882	2.836793	4.137809
C	0.239059	-4.808728	-1.184127
C	5.785738	-0.348288	0.809589
H	1.766899	-0.925427	0.652552
H	2.643983	0.402602	-4.914202
H	3.660641	-1.967510	-3.811898
H	-0.803738	2.596070	3.150350
H	-0.913776	0.829855	3.001505
H	-4.119353	1.018866	3.227559
H	-1.626856	3.142850	-0.288381
H	-0.093426	-2.853482	1.028338
H	0.071704	-1.130466	2.735286
H	1.225588	-4.706378	2.253047
H	2.982699	-5.937588	3.513000
H	4.951387	-4.707770	4.460720
H	5.130674	-2.220334	4.143708
H	3.371671	-0.995697	2.896562
H	2.637860	-5.567730	-0.449031
H	4.782486	-5.187993	0.743959
H	5.806499	-2.935661	0.796687
H	2.747440	5.095155	-3.184550
H	0.456690	5.616293	-4.014493
H	-1.242582	3.807419	-4.230057
H	3.607291	1.972626	1.425763
H	5.402088	1.695579	3.121027
H	4.836831	0.955066	5.452627
H	2.443720	0.515471	6.060215
H	0.645208	0.860505	4.398043
H	1.472065	3.989555	3.221592
H	1.675775	6.441627	2.843322
H	1.271229	7.398176	0.558062
H	0.701865	5.865272	-1.342370
H	0.553243	3.404813	-0.971437
H	-4.236288	2.910411	4.890053
H	-2.565443	2.347467	4.631656
H	-3.119927	3.869798	3.884316
H	-6.017286	2.580609	3.196037
H	-5.168782	3.665600	2.066471
H	-5.649330	2.020456	1.546060
H	-3.462443	4.386911	-1.342487
H	-3.943113	2.677884	-1.222054
H	-4.619312	3.868197	-0.093184
H	-2.035218	5.484788	0.433794
H	-3.149022	4.938591	1.715243
H	-1.401463	4.573391	1.816060

H	-0.582938	0.294943	-3.084345
H	-2.967837	0.545880	-3.433523
H	-2.394969	1.911561	-2.432797
H	-2.783243	2.163827	-4.159374
H	-1.488961	-0.324510	-5.341773
H	-1.070585	1.304984	-5.958589
H	0.224734	0.177914	-5.450268
H	3.815095	1.611356	-2.141318
H	5.839742	2.612857	-3.188493
H	4.604736	2.234302	-4.426987
H	4.848923	3.925073	-3.894443
H	5.117398	3.172254	-0.779047
H	4.203719	4.575727	-1.401724
H	3.374290	3.363070	-0.394433
H	-5.310221	-2.617635	3.415096
H	-7.314089	-2.504621	1.931974
H	-7.198324	-1.197024	-0.186233
H	-2.055610	-0.921939	2.698219
H	-4.208243	0.739697	-1.313754
H	-5.318442	-0.158717	-3.357280
H	-4.547420	-1.480490	-2.428386
H	-6.316525	-1.251880	-2.362581
H	-6.206779	1.897452	-2.225545
H	-7.294667	0.868487	-1.253217
H	-6.219098	2.042510	-0.446883
H	-2.162176	-1.296720	5.142595
H	-3.384831	-0.080314	4.679587
H	-3.883087	-1.755526	5.039877
H	-1.483241	-3.145210	3.623472
H	-3.178654	-3.689585	3.440460
H	-2.187497	-3.326714	1.997554
H	-1.154915	-1.572340	-3.107872
H	-2.083258	-3.412115	-4.450746
C	-3.796841	-5.412940	-3.645956
H	-4.328323	-4.789673	-1.016450
H	-3.484762	-2.904726	0.307261
H	4.100625	0.104931	-0.443430
H	0.584686	-2.787021	-1.823438
H	-0.788558	-4.745890	-1.580908
H	0.611232	-5.834077	-1.372536
H	0.192925	-4.653639	-0.093517
H	0.293304	-4.304003	-3.817551
H	1.787157	-3.338222	-3.946329
H	1.878538	-5.059894	-3.462528
H	6.058866	0.722117	0.849375
H	5.241610	-0.598239	1.732927
H	6.730037	-0.924831	0.795040
H	6.251769	0.659312	-1.648829
H	6.635396	-1.086256	-1.752066
H	5.230278	-0.416335	-2.635100
H	-4.797379	-5.733030	-3.301970
H	-3.860232	-5.167748	-4.722054
H	-3.122404	-6.288019	-3.548828

Table S93. Cartesian geometry of 3e-INT1 (13.5 kcal/mol) in Figure S146 in Angstrom [Å].

Atomtype	X Coordinates	Y Coordinates	Z Coordinates
C	5.109499	-0.403175	0.378326
C	3.950113	-0.299213	1.212794
C	4.063605	0.379173	2.475572
C	5.321473	0.853362	2.893907

C	6.462720	0.710043	2.096331
C	6.340785	0.100050	0.844001
N	2.676308	-0.826992	0.796624
Si	2.121301	-2.426798	1.436053
C	3.337926	-2.966049	2.830825
C	4.610133	-3.670134	2.318397
C	2.852385	0.703902	3.355330
C	2.941771	0.143314	4.790876
C	5.074025	-1.003180	-1.026988
C	6.007704	-2.225659	-1.166554
Ge	1.417824	0.201380	-0.240640
Ni	-0.828627	0.027666	-0.086282
P	-1.106049	-1.770135	1.242731
C	-1.617994	-3.501420	0.750685
C	-2.123931	-4.414372	1.704691
C	-2.427891	-5.734566	1.341028
C	-2.218830	-6.164877	0.018383
C	-1.722285	-5.262989	-0.934955
C	-1.436123	-3.934514	-0.575264
C	2.521566	1.731835	-0.956916
C	3.530864	2.459129	-0.286680
C	4.235450	3.508150	-0.903008
C	3.956357	3.851037	-2.242966
C	2.944492	3.147215	-2.934097
C	2.238361	2.126586	-2.290728
C	1.908622	-3.786394	0.093276
C	1.413609	-5.099745	0.742202
C	0.405086	-2.182095	2.269640
C	3.090124	-4.096462	-0.842841
Si	-0.655863	1.645778	1.773195
C	-1.925571	2.717013	2.711211
C	-1.639296	4.053539	3.068847
C	-2.549999	4.816676	3.820070
C	-3.770572	4.253355	4.230706
C	-4.068890	2.922357	3.890727
C	-3.154017	2.167298	3.139595
C	-1.756621	0.519578	-1.728018
N	-1.806182	-0.072099	-2.986868
C	-2.372483	0.765677	-3.952207
C	-2.715636	1.915344	-3.310994
N	-2.353068	1.755969	-1.972970
C	-1.594345	-1.459570	-3.307818
C	-2.720075	-2.321200	-3.295499
C	-2.543820	-3.633074	-3.776050
C	-1.293046	-4.075884	-4.225654
C	-0.187873	-3.212607	-4.202600
C	-0.322335	-1.881965	-3.761565
C	-2.742521	2.762436	-1.011274
C	-1.919018	3.904344	-0.836753
C	-2.428046	4.957751	-0.052909
C	-3.686720	4.866769	0.552217
C	-4.451964	3.702937	0.415927
C	-4.001522	2.623477	-0.368395
C	-4.089122	-1.856972	-2.803584
C	-4.626009	-2.743330	-1.661762
C	0.834266	-0.894987	-3.817458
C	0.904593	-0.189054	-5.188137
C	-0.524289	3.982419	-1.458001
C	-0.551875	4.527160	-2.904007
C	-4.838078	1.348887	-0.519196

C	-5.672961	1.353337	-1.820909
C	-5.095457	-1.770712	-3.970136
C	2.188030	-1.515587	-3.447653
C	-2.392683	-1.478254	2.564490
C	-3.748147	-1.451583	2.159111
C	-4.774508	-1.235057	3.089208
C	-4.467615	-1.004708	4.442945
C	-3.125909	-1.001509	4.852840
C	-2.097739	-1.247005	3.924837
C	2.609090	2.229463	3.419109
C	5.426852	0.036040	-2.114777
C	2.676864	-3.780979	3.963362
C	0.469941	4.817354	-0.627144
C	-5.776948	1.084410	0.671976
H	-1.715345	1.046158	0.716889
H	-2.497059	0.449195	-4.987091
H	-3.202342	2.821694	-3.668147
H	0.176104	-3.058441	2.903947
H	0.553905	-1.323763	2.949778
H	3.675771	-2.010791	3.269191
H	1.087225	-3.389180	-0.536712
H	0.388637	2.624950	1.354796
H	-0.101409	0.782644	2.861940
H	-0.693491	4.512494	2.748992
H	-2.308641	5.855861	4.082025
H	-4.484203	4.848519	4.816134
H	-5.016665	2.467632	4.209139
H	-3.399884	1.129857	2.888229
H	-1.819529	5.854043	0.106463
H	-4.055406	5.690372	1.174914
H	-5.408431	3.631270	0.942458
H	-3.397369	-4.321373	-3.779761
H	-1.174915	-5.104808	-4.589691
H	0.787719	-3.570725	-4.550437
H	-4.001370	-1.605941	1.102764
H	-5.818774	-1.230998	2.753679
H	-5.269468	-0.820050	5.169068
H	-2.871499	-0.809377	5.903157
H	-1.061352	-1.253936	4.277494
H	-2.279951	-4.089749	2.741278
H	-2.822408	-6.431242	2.092500
H	-2.445364	-7.200482	-0.267040
H	-1.559468	-5.586295	-1.968583
H	-1.070386	-3.227295	-1.328188
H	3.428888	-4.047627	4.733492
H	1.874675	-3.216118	4.472544
H	2.236679	-4.727222	3.595601
H	5.323519	-3.832329	3.151526
H	4.386862	-4.662150	1.883724
H	5.130838	-3.066414	1.555314
H	2.770935	-4.804592	-1.634418
H	3.484475	-3.201225	-1.342986
H	3.928922	-4.569291	-0.303223
H	1.107995	-5.823386	-0.038012
H	2.219363	-5.573857	1.334307
H	0.541798	-4.958343	1.402193
H	0.610224	-0.132365	-3.052360
H	2.945033	-0.721316	-3.329559
H	2.113430	-2.066521	-2.496177
H	2.555595	-2.214612	-4.222282

H	1.724597	0.553938	-5.193463
H	1.099101	-0.922121	-5.994241
H	-0.036833	0.338516	-5.422407
H	-3.971801	-0.841762	-2.393365
H	-6.072585	-1.396867	-3.611711
H	-4.734745	-1.084870	-4.758416
H	-5.257954	-2.764161	-4.429656
H	-5.561489	-2.313600	-1.256178
H	-4.850350	-3.769041	-2.009490
H	-3.890036	-2.823448	-0.844743
H	5.402432	1.362174	3.863126
H	7.434862	1.085247	2.441649
H	7.226117	0.013817	0.201192
H	1.969516	0.256210	2.876447
H	4.039321	-1.333933	-1.207979
H	5.367010	-0.429006	-3.117335
H	4.745221	0.899561	-2.091833
H	6.459476	0.411905	-1.985880
H	5.886500	-2.690369	-2.163369
H	7.068212	-1.927997	-1.063539
H	5.797887	-2.996380	-0.407461
H	2.016475	0.386677	5.347118
H	3.068110	-0.952323	4.808404
H	3.788864	0.589351	5.345732
H	1.687995	2.448228	3.990756
H	3.452105	2.743963	3.918187
H	2.487754	2.666464	2.414299
H	1.441188	1.628121	-2.855404
H	2.727231	3.429011	-3.971231
O	4.588511	4.833616	-2.944342
H	5.006687	4.034532	-0.331612
H	3.809364	2.206137	0.739057
H	-4.118691	0.508246	-0.565926
H	-0.140264	2.946359	-1.488291
H	1.495789	4.635488	-0.990438
H	0.267018	5.901452	-0.721299
H	0.434676	4.550816	0.442161
H	0.478878	4.592939	-3.298049
H	-1.132310	3.882979	-3.584550
H	-0.994354	5.541726	-2.924340
H	-6.208832	0.070621	0.584400
H	-5.248688	1.150322	1.635119
H	-6.622502	1.797927	0.689166
H	-6.267852	0.423783	-1.895268
H	-6.374609	2.209226	-1.820438
H	-5.045845	1.420617	-2.723632
C	5.613569	5.579375	-2.306078
H	5.982737	6.305540	-3.050138
H	5.231726	6.129673	-1.420535
H	6.454648	4.930376	-1.983509

Table S94. Cartesian geometry of 3f-INT1 (13.7 kcal/mol) in Figure S146 in Angstrom [Å].

Atomtype	X Coordinates	Y Coordinates	Z Coordinates
C	-4.923062	-1.204164	-0.572604
C	-3.740968	-1.054771	-1.367356
C	-3.869222	-0.569117	-2.714551
C	-5.151503	-0.327185	-3.242780
C	-6.308367	-0.517607	-2.478257
C	-6.180311	-0.936951	-1.150512
N	-2.435506	-1.345754	-0.833766

Si	-1.641340	-2.922648	-1.224737
C	-2.690042	-3.796757	-2.586185
C	-3.891230	-4.593165	-2.038503
C	-2.661132	-0.200492	-3.580841
C	-2.603865	-0.934479	-4.937528
C	-4.890979	-1.605131	0.901842
C	-5.670144	-2.911366	1.170672
Ge	-1.386992	-0.026130	0.104990
Ni	0.872618	0.083334	0.073038
P	1.458393	-1.821327	-0.974215
C	2.152102	-3.387595	-0.220358
C	2.831369	-4.343336	-1.010789
C	3.277280	-5.549413	-0.450290
C	3.039541	-5.822893	0.908710
C	2.371009	-4.876963	1.700656
C	1.941493	-3.659812	1.143661
C	-2.705699	1.418017	0.554478
C	-3.776223	1.909462	-0.232646
C	-4.626151	2.928953	0.208882
C	-4.470882	3.516405	1.498845
C	-3.392116	3.034809	2.296718
C	-2.538953	2.034218	1.818538
C	-1.339375	-4.057945	0.297359
C	-0.641727	-5.365214	-0.144219
C	0.080083	-2.561107	-2.003946
C	-2.525757	-4.401958	1.215164
Si	0.613865	1.403311	-1.998559
C	1.800951	2.492313	-3.022589
C	1.377065	3.720050	-3.578694
C	2.233181	4.486493	-4.388549
C	3.536852	4.036038	-4.659501
C	3.973379	2.813160	-4.120892
C	3.111990	2.054245	-3.312580
C	1.624979	0.911536	1.666618
N	1.670829	0.505270	2.997745
C	2.065335	1.532939	3.859719
C	2.298735	2.622445	3.079275
N	2.043681	2.239049	1.761980
C	1.618967	-0.842767	3.501326
C	2.844459	-1.537487	3.661091
C	2.808740	-2.783732	4.315815
C	1.599772	-3.327389	4.768726
C	0.396279	-2.634238	4.572927
C	0.385632	-1.369804	3.952973
C	2.363888	3.150821	0.686869
C	1.418180	4.142235	0.318546
C	1.839209	5.138270	-0.583516
C	3.133494	5.132314	-1.115729
C	4.027349	4.108057	-0.783410
C	3.667418	3.093911	0.125024
C	4.170697	-0.961981	3.169870
C	4.896363	-1.917991	2.201512
C	-0.890364	-0.553378	3.810597
C	-1.129792	0.339503	5.046513
C	-0.009323	4.123171	0.865040
C	-0.131769	4.866470	2.214579
C	4.646162	1.973480	0.490917
C	5.387652	2.261884	1.817049
C	5.076049	-0.571388	4.356952
C	-2.130499	-1.401182	3.498672

C	2.781385	-1.541728	-2.262499
C	4.094158	-1.282962	-1.802058
C	5.143628	-1.059536	-2.704265
C	4.898562	-1.054921	-4.089899
C	3.596027	-1.283165	-4.558423
C	2.548399	-1.535145	-3.654180
C	-2.604568	1.325495	-3.822284
C	-5.430629	-0.487552	1.822517
C	-1.862255	-4.653905	-3.567701
C	-1.050823	4.694290	-0.117424
C	5.686799	1.681847	-0.605601
H	1.675684	1.089552	-0.826476
H	2.164933	1.377142	4.933267
H	2.644758	3.624849	3.327006
H	0.457541	-3.473185	-2.502259
H	-0.130596	-1.823669	-2.799793
H	-3.118898	-2.959830	-3.164399
H	-0.615733	-3.474795	0.902159
H	-0.561232	2.292158	-1.767180
H	0.232096	0.342136	-2.982082
H	0.364176	4.091440	-3.369667
H	1.883735	5.440798	-4.805767
H	4.207886	4.634257	-5.290484
H	4.987758	2.446295	-4.328119
H	3.466621	1.101372	-2.905074
H	1.135114	5.917728	-0.893153
H	3.434340	5.905538	-1.832399
H	5.015437	4.092497	-1.253042
H	3.741759	-3.342433	4.455617
H	1.592366	-4.303824	5.270449
H	-0.544983	-3.072492	4.923417
H	4.295213	-1.260143	-0.723644
H	6.155551	-0.873193	-2.324204
H	5.716536	-0.865202	-4.796501
H	3.388041	-1.268300	-5.636093
H	1.545536	-1.724640	-4.050301
H	3.011433	-4.142588	-2.074506
H	3.805300	-6.280691	-1.076541
H	3.377482	-6.770584	1.348020
H	2.184006	-5.077838	2.760913
H	1.439605	-2.914582	1.771070
H	-2.525103	-5.115987	-4.327310
H	-1.108907	-4.055759	-4.112312
H	-1.328362	-5.477189	-3.056206
H	-4.527137	-4.954660	-2.871813
H	-3.570287	-5.482584	-1.464920
H	-4.529044	-3.971771	-1.386461
H	-2.167373	-4.954677	2.107417
H	-3.060709	-3.511731	1.573524
H	-3.263490	-5.047537	0.708002
H	-0.294352	-5.934111	0.739881
H	-1.342460	-6.016275	-0.700817
H	0.243496	-5.194190	-0.778694
H	-0.724554	0.105268	2.941104
H	-2.973679	-0.743505	3.226839
H	-1.933784	-2.078828	2.652245
H	-2.449185	-2.013281	4.363446
H	-2.039623	0.952652	4.902151
H	-1.271680	-0.279731	5.952945
H	-0.281341	1.023160	5.225286

H	3.948862	-0.041711	2.607137
H	6.018859	-0.121849	3.993631
H	4.577238	0.164184	5.014175
H	5.333561	-1.456969	4.968532
H	5.789604	-1.421237	1.777666
H	5.235115	-2.839032	2.711774
H	4.234367	-2.221451	1.373615
H	-5.241815	0.036107	-4.274623
H	-7.299449	-0.324697	-2.909177
H	-7.082323	-1.056792	-0.536914
H	-1.757334	-0.471955	-3.016142
H	-3.834557	-1.770122	1.165471
H	-5.374761	-0.807987	2.880559
H	-4.857746	0.445431	1.710318
H	-6.491632	-0.266441	1.599588
H	-5.545411	-3.223936	2.224801
H	-6.752594	-2.771674	0.989651
H	-5.324729	-3.739027	0.530226
H	-1.683210	-0.647716	-5.480673
H	-2.600802	-2.031734	-4.822679
H	-3.463999	-0.665075	-5.579400
H	-1.689658	1.593259	-4.382938
H	-3.478793	1.664703	-4.409861
H	-2.590591	1.887799	-2.874150
H	-1.709850	1.733242	2.470758
H	-3.204758	3.446384	3.292784
N	-5.319225	4.513689	1.951430
H	-5.425985	3.258105	-0.460949
H	-3.978802	1.485003	-1.218968
H	4.036898	1.057769	0.620341
H	-0.263346	3.060988	1.033128
H	-2.064871	4.428028	0.227028
H	-0.992058	5.798377	-0.174748
H	-0.919213	4.288493	-1.134051
H	-1.183879	4.850484	2.554209
H	0.483877	4.406854	3.005059
H	0.179916	5.922844	2.101656
H	6.236315	0.755596	-0.356597
H	5.218792	1.548238	-1.592541
H	6.434896	2.493593	-0.681912
H	6.089933	1.439056	2.047639
H	5.973318	3.196971	1.730235
H	4.700000	2.362205	2.671291
C	-6.400993	4.987426	1.103894
C	-5.093874	5.121677	3.251582
H	-6.978288	5.760951	1.635854
H	-6.026702	5.432072	0.156894
H	-7.100352	4.168981	0.833335
H	-5.852364	5.899716	3.435412
H	-5.163996	4.379347	4.074741
H	-4.092936	5.599646	3.319417

Table S95. Cartesian geometry of 3g-INT1 (14.5 kcal/mol) in Figure S146 in Angstrom [Å].

Atomtype	X Coordinates	Y Coordinates	Z Coordinates
C	-5.168311	-1.186377	-1.147330
C	-4.106094	-1.550696	-0.259649
C	-4.109785	-2.862258	0.327759
C	-5.185375	-3.731867	0.067858
C	-6.240808	-3.361096	-0.774044
C	-6.213005	-2.102159	-1.383404

N	-3.034470	-0.638582	0.033066
Si	-3.112502	0.327379	1.558928
C	-4.559472	-0.361117	2.632762
C	-5.940762	0.223526	2.275943
C	-2.934133	-3.394845	1.150767
C	-3.317528	-3.867149	2.568996
C	-5.194393	0.143618	-1.898633
C	-6.492161	0.943372	-1.657720
Ge	-1.436135	-0.452165	-1.045396
Ni	0.630536	0.051381	-0.200581
P	0.148782	0.623852	1.910072
C	0.092586	2.363546	2.596638
C	0.160024	2.606450	3.987988
C	0.030840	3.907959	4.494574
C	-0.182471	4.985531	3.616222
C	-0.246511	4.752712	2.234037
C	-0.094947	3.451137	1.724662
N	-1.790851	-1.430285	-2.629561
C	-3.243113	2.230935	1.307100
C	-3.279919	2.939761	2.681307
C	-1.496664	-0.001878	2.545183
C	-4.362974	2.799703	0.416925
Si	0.859483	-2.416954	0.319210
C	2.343365	-3.470997	0.884865
C	2.507773	-4.809237	0.459073
C	3.575919	-5.595049	0.924585
C	4.501181	-5.056185	1.836069
C	4.349315	-3.730481	2.276112
C	3.284701	-2.948958	1.799491
C	1.862787	0.880489	-1.463027
N	1.804200	2.088356	-2.157895
C	2.755334	2.165993	-3.179712
C	3.460856	1.003814	-3.141274
N	2.927616	0.238473	-2.102243
C	1.135882	3.296897	-1.742475
C	1.874178	4.223185	-0.962129
C	1.278086	5.468387	-0.684454
C	-0.008044	5.773070	-1.146836
C	-0.725693	4.834690	-1.902277
C	-0.160098	3.587679	-2.231844
C	3.607593	-0.979830	-1.714464
C	3.300758	-2.193665	-2.383188
C	4.123315	-3.307240	-2.124235
C	5.194644	-3.225622	-1.228692
C	5.443985	-2.033313	-0.541939
C	4.658781	-0.885536	-0.763586
C	3.277257	3.915808	-0.443949
C	3.347659	3.962060	1.095569
C	-0.878991	2.594796	-3.132752
C	-0.508826	2.814640	-4.615038
C	2.099274	-2.301246	-3.323427
C	2.408948	-1.772310	-4.742791
C	4.939288	0.411477	-0.000709
C	5.855876	1.364897	-0.801405
C	4.323450	4.850297	-1.086385
C	-2.401951	2.585391	-2.943201
C	1.321617	-0.122476	3.158586
C	2.606871	0.454310	3.288593
C	3.537712	-0.056450	4.204488
C	3.219440	-1.181071	4.987036

C	1.958007	-1.779099	4.848703
C	1.013498	-1.248276	3.952661
C	-2.211562	-4.538698	0.403551
C	-4.972211	-0.052183	-3.413834
C	-4.305292	-0.282448	4.153586
C	1.542065	-3.734106	-3.432618
C	5.555746	0.177481	1.391192
H	1.690857	-1.089537	0.034022
H	2.853519	3.050446	-3.808191
H	4.299602	0.650886	-3.739766
H	-1.619237	0.328319	3.593075
H	-1.418963	-1.104016	2.562869
H	-4.602966	-1.431138	2.363896
H	-2.280477	2.487053	0.820613
H	0.285793	-3.137614	-0.852059
H	-0.095152	-2.522231	1.463472
H	1.792336	-5.248237	-0.250799
H	3.686897	-6.630984	0.576420
H	5.335564	-5.669165	2.202478
H	5.063303	-3.297488	2.989445
H	3.184626	-1.915572	2.149040
H	3.912024	-4.262901	-2.613471
H	5.810154	-4.110681	-1.029221
H	6.254954	-2.002304	0.192170
H	1.826423	6.201468	-0.080882
H	-0.458937	6.746147	-0.912397
H	-1.732416	5.082167	-2.256728
H	2.877426	1.319970	2.671738
H	4.521881	0.418775	4.298831
H	3.952780	-1.590417	5.693377
H	1.700195	-2.665007	5.443102
H	0.032364	-1.727906	3.880559
H	0.313178	1.769925	4.681346
H	0.088022	4.081026	5.577373
H	-0.296573	6.003918	4.010123
H	-0.409300	5.584814	1.541088
H	-0.120230	3.275190	0.642974
H	-5.169375	-0.700374	4.708995
H	-3.411991	-0.857514	4.458776
H	-4.166398	0.758777	4.500868
H	-6.740094	-0.308058	2.830752
H	-6.017362	1.294190	2.541908
H	-6.162745	0.116748	1.200299
H	-4.246253	3.898352	0.319727
H	-4.356793	2.380050	-0.598972
H	-5.364541	2.615549	0.842384
H	-3.147910	4.031693	2.554856
H	-4.254742	2.780552	3.180118
H	-2.485949	2.602636	3.367928
H	-0.509914	1.599729	-2.834028
H	-2.840425	1.723252	-3.472234
H	-2.662709	2.501536	-1.875308
H	-2.879584	3.499413	-3.344028
H	-1.008001	2.057823	-5.249094
H	-0.832769	3.817445	-4.953744
H	0.579912	2.731258	-4.778961
H	3.529578	2.887691	-0.743421
H	5.340334	4.588438	-0.739829
H	4.305740	4.770178	-2.188940
H	4.135501	5.907160	-0.818508

H	4.351205	3.647607	1.439325
H	3.159662	4.980317	1.484072
H	2.594506	3.292851	1.543221
H	-5.184976	-4.730510	0.523642
H	-7.070115	-4.054232	-0.965712
H	-7.023443	-1.819627	-2.067571
H	-2.212006	-2.571637	1.254704
H	-4.348044	0.738057	-1.521968
H	-5.054241	0.915857	-3.943663
H	-3.971691	-0.468247	-3.606935
H	-5.726928	-0.736613	-3.845065
H	-6.418015	1.942945	-2.126263
H	-7.368147	0.433286	-2.100767
H	-6.692594	1.088928	-0.583325
H	-2.412142	-4.197115	3.113467
H	-3.796475	-3.069191	3.161657
H	-4.014306	-4.726103	2.534280
H	-1.322248	-4.868763	0.972551
H	-2.879568	-5.411526	0.275209
H	-1.870650	-4.219670	-0.594848
H	3.960324	0.904969	0.150303
H	1.313157	-1.665441	-2.876457
H	0.561215	-3.718586	-3.940254
H	2.209108	-4.383357	-4.030991
H	1.404088	-4.198364	-2.442424
H	1.537839	-1.925228	-5.406868
H	2.644789	-0.696266	-4.747224
H	3.267097	-2.319801	-5.177605
H	5.534264	1.118512	1.970861
H	5.005265	-0.587583	1.959668
H	6.614065	-0.137276	1.319727
H	6.049757	2.285570	-0.220301
H	6.828131	0.876499	-1.003643
H	5.411911	1.663780	-1.764090
C	-1.097066	-1.001277	-3.834481
C	-2.558338	-2.645509	-2.862612
H	-3.276335	-2.529172	-3.704438
H	-1.880534	-3.489350	-3.128572
H	-3.138823	-2.941204	-1.980125
H	-1.797436	-0.557201	-4.580846
H	-0.324449	-0.244381	-3.610794
H	-0.590680	-1.850442	-4.338216

Table S96. Cartesian geometry of 3a-TS1 (31.4 kcal/mol) in Figure S146 in Angstrom [Å].

Atomtype	X Coordinates	Y Coordinates	Z Coordinates
C	-1.671559	-1.518790	4.053023
C	-2.030837	-1.850807	2.732874
C	-3.398955	-2.033983	2.426976
C	-4.380209	-1.885779	3.416045
C	-4.013240	-1.537577	4.728759
C	-2.658637	-1.355967	5.041903
P	-0.810197	-1.964295	1.331474
C	0.804587	-2.295884	2.195474
Si	2.423660	-2.252521	1.151018
C	3.860215	-2.869753	2.280528
C	3.422977	-3.929376	3.315489
Ni	-0.982264	-0.266987	-0.096695
C	-1.703889	0.572525	-1.648047
N	-1.680222	0.105021	-2.955595
C	-2.371374	0.958857	-3.821650

C	-2.838537	1.986971	-3.065485
N	-2.441119	1.740666	-1.749901
C	-1.356063	-1.230851	-3.411206
C	-2.422287	-2.168814	-3.459581
C	-2.151524	-3.441623	-3.997886
C	-0.877070	-3.766088	-4.474731
C	0.151078	-2.814183	-4.442556
C	-0.067603	-1.518014	-3.933687
C	-2.996280	2.572952	-0.698636
C	-2.351148	3.789240	-0.369786
C	-3.018332	4.667171	0.505096
C	-4.279374	4.353515	1.020871
C	-4.891394	3.140480	0.686887
C	-4.268008	2.220849	-0.175632
C	-3.816899	-1.849132	-2.920651
C	-4.948881	-2.334279	-3.847221
C	1.028879	-0.456025	-3.972231
C	2.442092	-1.019082	-4.182858
C	-0.999760	4.162336	-0.967254
C	-0.085461	4.925181	0.008398
C	-4.970139	0.917596	-0.555508
C	-5.719610	0.264225	0.621241
Ge	1.204439	0.392330	-0.121572
C	2.045038	2.077099	-0.820898
C	2.954824	2.845035	-0.055686
C	3.539884	4.010936	-0.564123
C	3.218921	4.453449	-1.870442
C	2.288012	3.714548	-2.640033
C	1.714758	2.551749	-2.111085
C	3.818684	5.642784	-2.406964
N	4.301967	6.615356	-2.847213
Si	-0.166616	1.472871	1.927502
C	-1.755846	2.250775	2.668870
C	-1.661659	3.492536	3.344479
C	-2.717098	3.973391	4.137583
C	-3.891041	3.214661	4.284815
C	-4.005654	1.986430	3.614130
C	-2.955441	1.522166	2.806724
N	2.703934	-0.536750	0.706634
C	3.971371	0.062467	1.043801
C	5.023943	0.178656	0.075213
C	6.292398	0.638453	0.486090
C	6.555715	1.003021	1.809223
C	5.512791	0.960422	2.740114
C	4.221808	0.526730	2.383526
C	4.856527	-0.153302	-1.409735
C	5.101782	1.081966	-2.306254
C	3.118576	0.666113	3.434239
C	2.991865	2.131500	3.913570
C	-1.236872	-3.655695	0.683314
C	-1.543076	-4.701958	1.582135
C	-1.785212	-5.999600	1.107716
C	-1.718943	-6.266816	-0.271415
C	-1.424046	-5.228446	-1.168598
C	-1.192859	-3.926026	-0.695303
C	-3.978744	-2.405256	-1.492148
C	0.764035	0.623100	-5.049008
C	-1.164000	4.977318	-2.269623
C	-5.931677	1.124744	-1.746910
C	5.801267	-1.289106	-1.865083

C	3.273885	-0.244397	4.671742
C	2.135353	-3.446509	-0.327625
C	3.193438	-3.477553	-1.439918
C	1.867070	-4.885364	0.166915
C	5.108133	-3.343594	1.508010
H	-2.375795	-0.263511	0.344354
H	-2.484550	0.727734	-4.879934
H	-3.432003	2.862076	-3.324045
H	0.702834	-3.250889	2.742567
H	0.933077	-1.496760	2.946193
H	4.176538	-1.971981	2.838827
H	1.204085	-3.058613	-0.784659
H	0.819362	2.595344	2.119601
H	0.178409	0.516089	3.035724
H	-0.741130	4.087008	3.269203
H	-2.617219	4.939448	4.650966
H	-4.712065	3.581887	4.915044
H	-4.917936	1.384180	3.712716
H	-3.060392	0.565489	2.285851
H	-2.537661	5.607749	0.792924
H	-4.777146	5.045411	1.710456
H	-5.867794	2.897749	1.118625
H	-2.949008	-4.191991	-4.029799
H	-0.681465	-4.766482	-4.881921
H	1.136185	-3.083559	-4.835242
H	-3.698990	-2.283954	1.402763
H	-5.436957	-2.030410	3.157549
H	-4.781564	-1.404046	5.500759
H	-2.361026	-1.076707	6.060463
H	-0.622233	-1.366675	4.324141
H	-1.593968	-4.500404	2.659708
H	-2.022183	-6.804317	1.815697
H	-1.899395	-7.283282	-0.644469
H	-1.373128	-5.424431	-2.244995
H	-0.982907	-3.113248	-1.400178
H	4.283591	-4.225002	3.948886
H	2.632593	-3.557555	3.993479
H	3.040371	-4.848977	2.834866
H	5.933873	-3.565933	2.213522
H	4.914501	-4.268095	0.933361
H	5.473720	-2.570034	0.811155
H	2.823586	-4.061194	-2.307354
H	3.444272	-2.469907	-1.795391
H	4.131696	-3.952876	-1.105574
H	1.501078	-5.516600	-0.665918
H	2.796438	-5.349598	0.547168
H	1.108040	-4.940955	0.964895
H	1.006735	0.025384	-2.976249
H	3.182665	-0.218198	-4.024905
H	2.669302	-1.836163	-3.483606
H	2.578078	-1.396711	-5.214124
H	1.606540	1.338631	-5.075666
H	0.682854	0.156148	-6.048840
H	-0.154722	1.199941	-4.860056
H	-3.915115	-0.753018	-2.853519
H	-5.920234	-1.953979	-3.480470
H	-4.806365	-1.976591	-4.883774
H	-5.020001	-3.437541	-3.872687
H	-4.977249	-2.153994	-1.091822
H	-3.861314	-3.503322	-1.475862

H	-3.211953	-1.966495	-0.831948
H	5.697932	1.285354	3.771987
H	7.557582	1.338594	2.106680
H	7.097783	0.707895	-0.255731
H	2.173899	0.394234	2.944347
H	3.813147	-0.480943	-1.559565
H	4.991374	0.811192	-3.373091
H	4.402548	1.899742	-2.083961
H	6.129315	1.468956	-2.176369
H	5.565364	-1.592768	-2.902420
H	6.854676	-0.952480	-1.847430
H	5.723522	-2.180642	-1.223804
H	2.480151	-0.014269	5.407856
H	3.196261	-1.314703	4.424437
H	4.249184	-0.081432	5.168719
H	2.063020	2.260140	4.499460
H	3.844932	2.415644	4.558549
H	2.953405	2.842074	3.070900
H	0.985346	2.013970	-2.724485
H	2.022979	4.056782	-3.647238
H	4.250625	4.583165	0.043314
H	3.229697	2.527766	0.952593
H	-4.187139	0.208677	-0.866173
H	-0.492057	3.217585	-1.220868
H	0.930344	4.993179	-0.414707
H	-0.443318	5.957672	0.180327
H	-0.016312	4.415380	0.982331
H	-0.171947	5.237532	-2.682736
H	-1.718660	4.419778	-3.043754
H	-1.709421	5.918849	-2.067852
H	-6.097320	-0.730574	0.321486
H	-5.062268	0.130561	1.494059
H	-6.596431	0.860542	0.934913
H	-6.404632	0.165966	-2.032654
H	-6.734304	1.837317	-1.477415
H	-5.406506	1.519662	-2.633195

Table S97. Cartesian geometry of 3b-TS1 (27.2 kcal/mol) in Figure S146 in Angstrom [Å].

C	-2.927037	1.939008	-3.585411
C	-3.253550	1.688685	-2.239040
C	-4.525581	1.149889	-1.937734
C	-5.442593	0.863363	-2.957568
C	-5.100455	1.099027	-4.301739
C	-3.843027	1.637698	-4.609936
P	-2.065399	1.931938	-0.825210
C	-0.814519	3.127202	-1.515021
Si	0.774983	3.502714	-0.486745
C	1.694182	4.951776	-1.366618
C	0.748003	5.961395	-2.052650
Ni	-1.385488	-0.001324	0.061436
C	-1.486275	-1.464142	1.306078
N	-1.504000	-1.412377	2.694307
C	-1.544370	-2.686088	3.265322
C	-1.575337	-3.571169	2.234071
N	-1.561603	-2.824559	1.053865
C	-1.732839	-0.253751	3.524069
C	-3.074021	0.171759	3.715174
C	-3.290497	1.282986	4.551844
C	-2.222466	1.935426	5.183196
C	-0.919187	1.440547	5.046039

C	-0.655100	0.309548	4.248191
C	-1.810579	-3.514963	-0.197098
C	-0.762231	-4.266385	-0.781721
C	-1.080841	-5.093974	-1.875059
C	-2.385631	-5.169183	-2.368890
C	-3.403299	-4.413068	-1.776253
C	-3.147900	-3.579587	-0.672748
C	-4.255431	-0.552457	3.071976
C	-5.370308	-0.870101	4.088875
C	0.720632	-0.342623	4.275011
C	1.827731	0.582355	3.754069
C	0.663490	-4.241201	-0.240686
C	1.712023	-4.024632	-1.348250
C	-4.292086	-2.841501	0.022532
C	-5.402633	-2.372370	-0.935207
Ge	0.855511	0.381345	-0.079809
C	2.374877	-0.888013	0.194256
C	3.484492	-1.003052	-0.669138
C	4.534056	-1.891649	-0.377132
C	4.496498	-2.696391	0.773892
C	3.381198	-2.608785	1.622601
C	2.338098	-1.715026	1.330205
C	5.751107	-1.918075	-1.279117
F	6.637045	-0.946712	-0.936961
C	3.325564	-3.411517	2.902020
F	3.858786	-2.717844	3.949168
Si	-0.140517	-0.624130	-2.358205
C	-1.292038	-1.822448	-3.312922
C	-0.728538	-2.762133	-4.209301
C	-1.525852	-3.451036	-5.139478
C	-2.908812	-3.210157	-5.199338
C	-3.487416	-2.293399	-4.306093
C	-2.687801	-1.620818	-3.368952
N	1.778773	2.022341	-0.563440
C	3.137563	2.112642	-1.040235
C	4.243786	2.224361	-0.131648
C	5.541739	2.427594	-0.642873
C	5.791685	2.516592	-2.014296
C	4.726647	2.350398	-2.904635
C	3.412552	2.123851	-2.452469
C	4.106378	2.119135	1.387676
C	4.902318	0.926728	1.963796
C	2.353318	1.805570	-3.507395
C	2.790627	0.617969	-4.396962
C	-3.085752	3.066577	0.240731
C	-3.871327	4.080396	-0.351691
C	-4.571433	4.996091	0.447976
C	-4.490120	4.911204	1.849368
C	-3.718307	3.899395	2.441681
C	-3.026678	2.975122	1.641456
C	-4.798213	0.231478	1.863421
C	1.049150	-0.882520	5.684283
C	0.988222	-5.525078	0.554477
C	-4.896150	-3.706750	1.151732
C	4.549918	3.408702	2.113217
C	1.965026	2.993273	-4.413997
C	0.170089	3.986353	1.273688
C	1.197271	4.097925	2.412114
C	-0.682150	5.274060	1.214873
C	2.717301	5.677952	-0.470546

F	2.047297	-3.715808	3.256297
F	4.009563	-4.576868	2.811606
F	6.411960	-3.101296	-1.205983
F	5.422079	-1.715405	-2.577986
H	-2.664854	-0.494778	-0.439938
H	-1.554918	-2.825506	4.345648
H	-1.623274	-4.658341	2.215339
H	-1.353321	4.055218	-1.779151
H	-0.436158	2.686299	-2.453889
H	2.283997	4.458774	-2.159777
H	-0.499962	3.146062	1.538528
H	1.210441	-1.097985	-2.828917
H	-0.368006	0.631126	-3.154603
H	0.353873	-2.947245	-4.196086
H	-1.062265	-4.173273	-5.825211
H	-3.531278	-3.737387	-5.934666
H	-4.567244	-2.097306	-4.334191
H	-3.153488	-0.909015	-2.680681
H	-0.288254	-5.682116	-2.350975
H	-2.610008	-5.804061	-3.233940
H	-4.416973	-4.472311	-2.184184
H	-4.312836	1.647657	4.701437
H	-2.411777	2.814600	5.812537
H	-0.094594	1.914656	5.592034
H	-4.795043	0.939652	-0.896768
H	-6.423153	0.441720	-2.702318
H	-5.810222	0.858868	-5.103449
H	-3.562742	1.819861	-5.655096
H	-1.951328	2.355206	-3.854701
H	-3.937602	4.153608	-1.444721
H	-5.178707	5.779591	-0.023557
H	-5.029459	5.632513	2.476839
H	-3.648376	3.824203	3.532379
H	-2.439279	2.175588	2.106319
H	1.335045	6.756403	-2.555191
H	0.114146	5.488115	-2.825216
H	0.074520	6.459862	-1.330843
H	3.259808	6.449578	-1.053208
H	2.237035	6.191537	0.382855
H	3.472365	4.978041	-0.075207
H	0.675500	4.263835	3.376843
H	1.803666	3.188237	2.520529
H	1.887165	4.947223	2.265383
H	-1.206356	5.433661	2.176969
H	-0.045795	6.160984	1.035022
H	-1.458772	5.247751	0.431418
H	0.680832	-1.218513	3.608666
H	2.788003	0.043015	3.709821
H	1.583440	0.929201	2.736732
H	1.957325	1.471912	4.397259
H	2.001544	-1.442523	5.659298
H	1.150602	-0.058812	6.415620
H	0.256665	-1.562404	6.049276
H	-3.888174	-1.520088	2.691467
H	-6.141891	-1.503783	3.613321
H	-4.974003	-1.409250	4.969162
H	-5.874359	0.046452	4.447705
H	-5.631566	-0.318073	1.389712
H	-5.162684	1.230365	2.162490
H	-3.994149	0.363150	1.121031

H	4.918550	2.379346	-3.984589
H	6.809837	2.688178	-2.386195
H	6.379931	2.518041	0.059081
H	1.447094	1.500864	-2.967350
H	3.040214	1.952549	1.601582
H	4.821350	0.911181	3.066330
H	4.536951	-0.035727	1.580663
H	5.975972	1.002528	1.711777
H	4.338290	3.330759	3.196349
H	5.637441	3.573539	1.995314
H	4.029980	4.300736	1.732401
H	1.259879	2.653507	-5.196538
H	1.475812	3.808534	-3.857307
H	2.853915	3.414689	-4.921158
H	1.929533	0.245091	-4.981640
H	3.579778	0.922035	-5.110543
H	3.184354	-0.223284	-3.801996
H	1.478993	-1.669163	2.004249
H	5.314701	-3.387861	0.996489
H	3.551054	-0.393707	-1.573083
H	-3.862662	-1.934625	0.475823
H	0.740159	-3.389822	0.454051
H	2.714671	-3.921038	-0.900893
H	1.747394	-4.879050	-2.049253
H	1.504434	-3.111674	-1.928535
H	2.018322	-5.481888	0.952562
H	0.302992	-5.666487	1.408696
H	0.908738	-6.415663	-0.097481
H	-6.120016	-1.731673	-0.390322
H	-4.993124	-1.789558	-1.774896
H	-5.978860	-3.221165	-1.348589
H	-5.695998	-3.151717	1.677547
H	-5.336182	-4.633048	0.735748
H	-4.136548	-3.993786	1.898897
C	-2.927037	1.939008	-3.585411

Table S98. Cartesian geometry of 3c-TS1 (30.5 kcal/mol) in Figure S146 in Angstrom [Å].

Atomtype	X Coordinates	Y Coordinates	Z Coordinates
C	3.792583	1.266677	4.126572
C	2.839111	1.559159	3.142542
C	1.511836	1.086152	3.257895
C	1.164498	0.316391	4.384427
C	2.126201	0.010521	5.364527
C	3.440778	0.482272	5.240194
P	0.344698	1.437658	1.848733
C	-1.333095	1.215530	2.622173
Si	-2.876950	1.246793	1.470085
C	-4.441757	1.237742	2.600819
C	-4.236596	1.966634	3.946762
Ni	0.874645	0.331141	-0.004005
C	1.781970	0.152580	-1.667771
N	1.720107	0.999837	-2.767544
C	2.576772	0.580867	-3.791026
C	3.190389	-0.548329	-3.348857
N	2.715305	-0.793451	-2.059195
C	1.187412	2.345363	-2.816929
C	2.076463	3.400312	-2.476811
C	1.611733	4.721918	-2.619644
C	0.320279	4.984689	-3.088560
C	-0.528830	3.927931	-3.444113

C	-0.110412	2.585866	-3.340137
C	3.366854	-1.822872	-1.271495
C	2.945541	-3.166501	-1.414581
C	3.722720	-4.164913	-0.797653
C	4.875517	-3.840923	-0.076503
C	5.266001	-2.503846	0.056688
C	4.526026	-1.464170	-0.534662
C	3.482894	3.141174	-1.936505
C	4.550257	4.056372	-2.567724
C	-1.006375	1.438828	-3.801172
C	-2.486119	1.819678	-3.955025
C	1.720363	-3.536793	-2.240864
C	0.877351	-4.661891	-1.614338
C	4.995098	-0.014682	-0.415371
C	5.583036	0.324737	0.967207
Ge	-1.150046	-0.643097	-0.433087
N	-2.825830	-0.256901	0.495369
C	-3.982850	-1.116628	0.511577
C	-4.966457	-1.066373	-0.532062
C	-6.155564	-1.812593	-0.396869
C	-6.402726	-2.617417	0.718551
C	-5.414047	-2.727730	1.701705
C	-4.199871	-2.021256	1.610262
C	-4.801928	-0.254205	-1.818878
C	-4.786109	-1.161874	-3.069716
C	-3.125203	-2.331211	2.653950
C	-2.788535	-3.840782	2.672170
Si	0.297715	-2.076433	1.320525
C	1.972982	-2.802166	1.913008
C	2.089078	-4.198409	2.121471
C	3.183385	-4.744795	2.813250
C	4.185828	-3.903332	3.325386
C	4.093687	-2.517573	3.117420
C	3.008006	-1.980917	2.407665
C	-1.654386	-2.109019	-1.704963
C	-2.444462	-3.213471	-1.309974
C	-2.798111	-4.220379	-2.223877
C	-2.360398	-4.152720	-3.557760
C	-1.549766	-3.078698	-3.962808
C	-1.201853	-2.074137	-3.042971
C	0.486676	3.293472	1.791508
C	0.571021	4.035662	2.990813
C	0.597157	5.437831	2.960242
C	0.534179	6.114610	1.729139
C	0.459366	5.381300	0.534633
C	0.445180	3.976728	0.563761
C	3.490674	3.240553	-0.398314
C	-0.520787	0.812458	-5.129936
C	2.110273	-3.916507	-3.686571
C	6.010735	0.338418	-1.524749
C	-5.914400	0.805400	-1.990217
C	-3.460304	-1.882058	4.092709
C	-2.752391	2.885177	0.470804
C	-3.754768	3.113049	-0.669575
C	-2.761111	4.110479	1.411743
C	-5.724108	1.733621	1.902294
H	2.228699	0.403643	0.546517
H	2.682983	1.149386	-4.713887
H	3.934104	-1.194679	-3.811512
H	-1.426161	1.949010	3.443739

H	-1.341907	0.208409	3.074222
H	-4.615938	0.171227	2.824518
H	-1.750564	2.814107	0.004127
H	-0.491242	-3.339406	1.092920
H	-0.229320	-1.573763	2.637626
H	1.301871	-4.871671	1.756248
H	3.247331	-5.831494	2.961670
H	5.034856	-4.326647	3.878778
H	4.870634	-1.845783	3.504827
H	2.951260	-0.899576	2.249840
H	3.412737	-5.212161	-0.877216
H	5.459162	-4.631709	0.409363
H	6.157596	-2.263816	0.644858
H	2.268353	5.554745	-2.345045
H	-0.028939	6.020945	-3.184386
H	-1.529944	4.155710	-3.822486
H	3.131088	2.151813	2.268093
H	4.818115	1.641688	4.016431
H	4.190468	0.237165	6.003182
H	1.841851	-0.608518	6.224811
H	0.147087	-0.068446	4.504638
H	0.617508	3.513657	3.955023
H	0.662755	6.003235	3.898914
H	0.545570	7.211985	1.702834
H	0.412832	5.897654	-0.430179
H	0.406577	3.407245	-0.372186
H	-5.159676	1.910469	4.558549
H	-3.420673	1.524067	4.547637
H	-4.000985	3.038280	3.808787
H	-6.601866	1.588639	2.563989
H	-5.674696	2.811349	1.660306
H	-5.921711	1.175833	0.970963
H	-3.458945	3.996436	-1.271480
H	-3.810716	2.250018	-1.345031
H	-4.774313	3.304504	-0.292696
H	-2.479192	5.025325	0.855178
H	-3.772137	4.277685	1.828692
H	-2.056215	4.022130	2.254951
H	-0.939833	0.673452	-3.005047
H	-3.083194	0.907595	-4.116868
H	-2.876501	2.328233	-3.062273
H	-2.646041	2.480041	-4.828668
H	-1.223489	0.019929	-5.446721
H	-0.488736	1.579830	-5.926686
H	0.477425	0.355200	-5.047627
H	3.764117	2.106954	-2.194639
H	5.557398	3.732762	-2.245821
H	4.512418	4.021899	-3.672356
H	4.431165	5.110119	-2.254042
H	4.501464	3.032504	-0.003764
H	3.183385	4.246658	-0.061677
H	2.786932	2.504697	0.025529
H	-5.579660	-3.390655	2.560691
H	-7.346347	-3.170974	0.809549
H	-6.911246	-1.755135	-1.190365
H	-2.218316	-1.792551	2.348364
H	-3.827795	0.261428	-1.760452
H	-4.689349	-0.550814	-3.987012
H	-3.958324	-1.883592	-3.045735
H	-5.731376	-1.729284	-3.157697

H	-5.691889	1.460043	-2.853963
H	-6.890307	0.321322	-2.182224
H	-6.026521	1.443661	-1.100102
H	-2.669475	-2.226029	4.786376
H	-3.531970	-0.787394	4.189224
H	-4.419357	-2.316017	4.434625
H	-1.871554	-4.017484	3.264151
H	-3.608095	-4.427846	3.128707
H	-2.611697	-4.237088	1.658053
H	-0.553933	-1.259658	-3.382525
H	-1.186492	-3.019070	-4.997412
H	-2.641773	-4.935395	-4.274180
H	-3.422267	-5.059503	-1.889870
H	-2.805160	-3.291420	-0.281496
H	4.106585	0.621741	-0.547503
H	1.080859	-2.641389	-2.291334
H	-0.070304	-4.764726	-2.168000
H	1.399683	-5.636410	-1.657107
H	0.633140	-4.445464	-0.562290
H	1.203127	-4.157486	-4.271461
H	2.640504	-3.097260	-4.202916
H	2.770429	-4.805067	-3.688430
H	5.774845	1.411188	1.038436
H	4.892469	0.046154	1.777885
H	6.548564	-0.185394	1.142179
H	6.315605	1.399246	-1.445701
H	6.918380	-0.288014	-1.432405
H	5.587853	0.182721	-2.531863

Table S99. Cartesian geometry of 3d- TS1 (30.1 kcal/mol) in Figure S146 in Angstrom [Å].

Atomtype	X Coordinates	Y Coordinates	Z Coordinates
C	1.455627	1.233690	4.200304
C	1.807503	1.703802	2.920776
C	3.161858	2.015561	2.661304
C	4.136990	1.857454	3.654842
C	3.778796	1.370402	4.925152
C	2.437645	1.060937	5.192688
P	0.602651	1.846177	1.507117
C	-1.046431	1.957884	2.362510
Si	-2.637600	1.882373	1.278394
C	-4.140458	2.272883	2.425018
C	-3.813925	3.267419	3.560445
Ni	0.938577	0.314023	-0.066273
C	1.737224	-0.314435	-1.675283
N	1.691306	0.271322	-2.934559
C	2.459243	-0.439383	-3.863129
C	2.998169	-1.494781	-3.197830
N	2.567446	-1.406219	-1.872750
C	1.267598	1.615042	-3.268797
C	2.256140	2.633826	-3.204657
C	1.893154	3.928172	-3.624095
C	0.603948	4.197473	-4.095275
C	-0.345163	3.169572	-4.177358
C	-0.032003	1.850713	-3.790384
C	3.174201	-2.288702	-0.893705
C	2.628993	-3.580488	-0.699532
C	3.353174	-4.482023	0.103109
C	4.574110	-4.117953	0.678547
C	5.087186	-2.831838	0.477874
C	4.403424	-1.886911	-0.308204

C	3.664268	2.373626	-2.668908
C	4.766380	3.030031	-3.523246
C	-1.041263	0.716631	-3.956030
C	-2.489344	1.187560	-4.156565
C	1.324515	-4.003809	-1.362963
C	0.450181	-4.910175	-0.477970
C	5.000276	-0.500155	-0.545004
C	5.680682	0.091542	0.703926
Ge	-1.179325	-0.536523	-0.198632
C	-1.866981	-2.192771	-1.087013
C	-2.722046	-3.107630	-0.430577
C	-3.193169	-4.259848	-1.078118
C	-2.824687	-4.554705	-2.407560
C	-1.955761	-3.656908	-3.060161
C	-1.484890	-2.504407	-2.409795
C	-3.309107	-5.815041	-3.089833
Si	0.231945	-1.677320	1.786259
C	1.872731	-2.396997	2.475786
C	1.880023	-3.706554	3.016042
C	2.964175	-4.176582	3.776394
C	4.065731	-3.340144	4.026430
C	4.080829	-2.042877	3.489105
C	3.003648	-1.587933	2.712486
N	-2.767134	0.202639	0.666741
C	-3.988996	-0.521175	0.912851
C	-5.014596	-0.619636	-0.085988
C	-6.253900	-1.201763	0.252127
C	-6.512264	-1.705613	1.529835
C	-5.488574	-1.682271	2.482587
C	-4.225750	-1.130476	2.195540
C	-4.845275	-0.142651	-1.530454
C	-4.970619	-1.309722	-2.536243
C	-3.128420	-1.297662	3.248090
C	-2.916880	-2.789856	3.595885
C	0.896803	3.622917	1.033968
C	1.102086	4.599119	2.034529
C	1.244554	5.951737	1.691175
C	1.178897	6.344573	0.342419
C	0.984365	5.377054	-0.655742
C	0.852926	4.020855	-0.313414
C	3.762395	2.804376	-1.192177
C	-0.670924	-0.234085	-5.119219
C	1.579883	-4.693159	-2.721697
C	5.987973	-0.509535	-1.732879
C	-5.868723	0.951753	-1.910716
C	-3.350407	-0.511360	4.558201
C	-2.424143	3.235882	-0.070727
C	-3.458827	3.288414	-1.203541
C	-2.286444	4.637064	0.564853
C	-5.411628	2.707964	1.667847
H	2.323945	0.381970	0.403046
H	2.566027	-0.100809	-4.892746
H	3.662667	-2.292119	-3.525828
H	-1.035133	2.858037	3.003790
H	-1.118308	1.078345	3.026014
H	-4.387857	1.305077	2.893994
H	-1.455708	2.970968	-0.537589
H	-0.663103	-2.886282	1.868016
H	-0.189674	-0.855989	2.975468
H	1.015630	-4.365987	2.859513

H	2.943076	-5.196534	4.184223
H	4.908058	-3.700131	4.632403
H	4.935863	-1.378362	3.668797
H	3.030654	-0.576942	2.294486
H	2.947832	-5.482400	0.286908
H	5.116176	-4.830130	1.311843
H	6.031940	-2.552835	0.955637
H	2.629070	4.737606	-3.565759
H	0.334824	5.214647	-4.408156
H	-1.342829	3.398779	-4.563709
H	3.456635	2.373878	1.668314
H	5.183199	2.103022	3.431908
H	4.543741	1.228837	5.699152
H	2.147333	0.673146	6.177319
H	0.417099	0.980836	4.435106
H	1.152179	4.299498	3.089098
H	1.403377	6.700855	2.477752
H	1.281544	7.403457	0.071699
H	0.934820	5.670901	-1.709779
H	0.720528	3.265644	-1.096802
H	-4.707333	3.430475	4.196699
H	-3.005657	2.903959	4.221716
H	-3.504380	4.256042	3.173301
H	-6.266012	2.792060	2.369495
H	-5.289597	3.695456	1.185678
H	-5.695844	1.974547	0.893896
H	-3.121386	3.980028	-2.002271
H	-3.618208	2.302172	-1.657802
H	-4.439491	3.652954	-0.852150
H	-1.955352	5.373075	-0.193359
H	-3.259238	4.986114	0.959638
H	-1.552423	4.675964	1.386858
H	-1.004086	0.146104	-3.008841
H	-3.167315	0.320981	-4.093053
H	-2.796668	1.916358	-3.393419
H	-2.631382	1.647515	-5.153174
H	-1.453106	-1.007356	-5.231156
H	-0.607659	0.328994	-6.069831
H	0.286181	-0.754131	-4.959055
H	3.847951	1.287015	-2.701657
H	5.759990	2.694041	-3.173123
H	4.666610	2.760899	-4.591163
H	4.749814	4.133054	-3.445427
H	4.772677	2.596485	-0.796433
H	3.556433	3.883345	-1.076770
H	3.025126	2.245575	-0.591595
H	-5.665595	-2.116185	3.475164
H	-7.493381	-2.133792	1.773225
H	-7.040276	-1.256531	-0.511162
H	-2.197201	-0.926508	2.799613
H	-3.828583	0.276950	-1.621829
H	-4.861130	-0.937455	-3.572411
H	-4.211868	-2.085764	-2.365267
H	-5.967123	-1.784749	-2.467271
H	-5.634244	1.367203	-2.908993
H	-6.891556	0.532975	-1.956229
H	-5.879616	1.784217	-1.190154
H	-2.555438	-0.763516	5.285769
H	-3.326976	0.579347	4.407502
H	-4.322679	-0.768896	5.020185

H	-1.989808	-2.913729	4.185581
H	-3.758979	-3.183735	4.196312
H	-2.822530	-3.415304	2.692237
H	-0.794753	-1.850034	-2.951994
H	-1.642717	-3.860534	-4.093745
H	-3.865307	-4.942286	-0.539824
H	-3.044347	-2.920028	0.596536
H	4.164422	0.169976	-0.798421
H	0.748265	-3.084822	-1.555139
H	-0.546315	-5.019960	-0.936834
H	0.886851	-5.921433	-0.371311
H	0.314431	-4.483312	0.528343
H	0.617620	-4.977220	-3.186606
H	2.116670	-4.036603	-3.428150
H	2.182679	-5.610985	-2.581532
H	5.974817	1.139760	0.511752
H	5.005844	0.080469	1.573488
H	6.602290	-0.457712	0.972178
H	6.384515	0.507734	-1.913396
H	6.843038	-1.179248	-1.520426
H	5.505822	-0.854137	-2.663538
H	-4.333784	-6.080888	-2.770830
H	-3.303891	-5.711640	-4.190298
H	-2.656316	-6.675325	-2.836832

Table S100. Cartesian geometry of 3e-TS1 (31.0 kcal/mol) in Figure S146 in Angstrom [Å].

Atomtype	X Coordinates	Y Coordinates	Z Coordinates
C	1.836043	-1.909363	-3.877492
C	2.166493	-2.087283	-2.520584
C	3.530275	-2.190209	-2.162486
C	4.535086	-2.113111	-3.135805
C	4.196099	-1.917209	-4.487079
C	2.846069	-1.816950	-4.852403
P	0.910099	-2.094562	-1.144465
C	-0.671254	-2.547742	-2.015452
Si	-2.317635	-2.444143	-1.020134
C	-3.710502	-3.202179	-2.120436
C	-3.224848	-4.346769	-3.036191
Ni	1.005908	-0.266368	0.115091
C	1.648070	0.735741	1.598256
N	1.593021	0.396614	2.945275
C	2.230262	1.351758	3.744589
C	2.693259	2.316412	2.906626
N	2.349367	1.931158	1.609445
C	1.298084	-0.900730	3.517623
C	2.391618	-1.793500	3.679794
C	2.148173	-3.019926	4.327612
C	0.872980	-3.342649	4.802962
C	-0.183622	-2.433545	4.657512
C	0.005813	-1.183493	4.034026
C	2.915436	2.675756	0.499950
C	2.255854	3.843026	0.046215
C	2.927355	4.652525	-0.889458
C	4.206421	4.321376	-1.345751
C	4.833511	3.157433	-0.887845
C	4.207400	2.307066	0.041133
C	3.789818	-1.479063	3.147820
C	4.908219	-1.823946	4.150717
C	-1.128073	-0.164759	3.943283
C	-2.524495	-0.755092	4.188917

C	0.882835	4.241037	0.572933
C	-0.033791	4.849612	-0.503460
C	4.926687	1.061007	0.555803
C	5.731792	0.329028	-0.534462
Ge	-1.192129	0.356407	0.009782
C	-2.090074	2.055483	0.552165
C	-3.008314	2.742264	-0.281429
C	-3.622378	3.930815	0.124638
C	-3.332341	4.488820	1.390296
C	-2.405286	3.837634	2.230781
C	-1.800520	2.642375	1.799628
O	-3.980234	5.649438	1.698764
C	-3.716453	6.266296	2.947275
Si	0.190881	1.250240	-2.114515
C	1.782458	2.010516	-2.872713
C	1.684136	3.192618	-3.647208
C	2.753228	3.633856	-4.445255
C	3.946551	2.893183	-4.498249
C	4.066046	1.724874	-3.728368
C	3.001597	1.302069	-2.916829
N	-2.648513	-0.704978	-0.744633
C	-3.924283	-0.176757	-1.157881
C	-5.003112	-0.011026	-0.226138
C	-6.276484	0.362983	-0.703236
C	-6.521060	0.595547	-2.059350
C	-5.454585	0.509901	-2.960100
C	-4.158002	0.159887	-2.537937
C	-4.859406	-0.199301	1.285994
C	-5.148021	1.109931	2.055162
C	-3.033297	0.251128	-3.570857
C	-2.963677	1.666504	-4.190829
C	1.361988	-3.707246	-0.330438
C	1.718975	-4.824553	-1.118111
C	1.982482	-6.064423	-0.517333
C	1.887932	-6.202510	0.879001
C	1.543177	-5.092874	1.666456
C	1.289984	-3.848081	1.066256
C	4.013349	-2.173973	1.790260
C	-0.925869	1.028215	4.907546
C	0.997872	5.210591	1.770177
C	5.840249	1.396031	1.755689
C	-5.788579	-1.308812	1.829615
C	-3.105391	-0.785771	-4.712691
C	-2.038153	-3.492174	0.568646
C	-3.121262	-3.452192	1.655965
C	-1.719524	-4.962234	0.216667
C	-4.967970	-3.627266	-1.335534
H	2.412512	-0.268568	-0.292400
H	2.314584	1.227620	4.823329
H	3.252559	3.230707	3.097018
H	-0.534745	-3.546432	-2.468897
H	-0.796509	-1.823707	-2.839375
H	-4.030515	-2.370459	-2.771389
H	-1.129868	-3.037070	1.009615
H	-0.820984	2.312123	-2.458213
H	-0.075246	0.176832	-3.136106
H	0.749390	3.769436	-3.645894
H	2.649064	4.553944	-5.036582
H	4.778538	3.228083	-5.132254
H	4.993167	1.137654	-3.752529

H	3.110630	0.393044	-2.317268
H	2.434531	5.552084	-1.272626
H	4.707260	4.957831	-2.084772
H	5.824783	2.897581	-1.273141
H	2.968459	-3.736260	4.446676
H	0.698501	-4.307454	5.296588
H	-1.169745	-2.699786	5.049666
H	3.807703	-2.319842	-1.110132
H	5.587842	-2.192870	-2.835994
H	4.982158	-1.838397	-5.248771
H	2.569630	-1.656742	-5.902208
H	0.790792	-1.823653	-4.190057
H	1.792443	-4.724105	-2.208480
H	2.258427	-6.925222	-1.140230
H	2.085302	-7.173825	1.350872
H	1.469891	-5.187661	2.755245
H	1.039942	-2.978885	1.685544
H	-4.062626	-4.723642	-3.657234
H	-2.425935	-4.023812	-3.729089
H	-2.834358	-5.205633	-2.459157
H	-5.771012	-3.935739	-2.035060
H	-4.769890	-4.487080	-0.669254
H	-5.366486	-2.798240	-0.726030
H	-2.756314	-3.942771	2.581408
H	-3.407144	-2.423848	1.910894
H	-4.038283	-3.983578	1.347840
H	-1.357095	-5.502826	1.112610
H	-2.626815	-5.486145	-0.138926
H	-0.940019	-5.070114	-0.555716
H	-1.103003	0.212602	2.903469
H	-3.288609	-0.001819	3.937811
H	-2.710974	-1.646453	3.573550
H	-2.666563	-1.030622	5.251429
H	-1.792135	1.711886	4.840059
H	-0.853527	0.669313	5.951933
H	-0.023154	1.615614	4.678984
H	3.852491	-0.392968	2.969675
H	5.877654	-1.450365	3.772173
H	4.722588	-1.364444	5.139286
H	5.013850	-2.915251	4.295212
H	5.016236	-1.933118	1.394480
H	3.928466	-3.271142	1.884895
H	3.255250	-1.829859	1.066852
H	-5.625540	0.734169	-4.020871
H	-7.526957	0.864427	-2.407170
H	-7.101018	0.470521	0.012731
H	-2.091284	0.077974	-3.033547
H	-3.812043	-0.485619	1.483858
H	-5.064515	0.941694	3.145606
H	-4.453732	1.913727	1.774478
H	-6.177534	1.463908	1.860583
H	-5.563830	-1.511244	2.893896
H	-6.848387	-0.998300	1.766451
H	-5.682889	-2.253713	1.274254
H	-2.308790	-0.582961	-5.453753
H	-2.970395	-1.819152	-4.356477
H	-4.076916	-0.733186	-5.240375
H	-2.025738	1.783067	-4.764591
H	-3.810389	1.843141	-4.881196
H	-2.983496	2.456104	-3.420819

H	-1.068059	2.178508	2.467998
H	-2.142042	4.244447	3.212243
H	-4.336251	4.451762	-0.524058
H	-3.263551	2.342216	-1.265919
H	4.149400	0.361434	0.899726
H	0.395434	3.320555	0.931142
H	-1.058917	4.938124	-0.107815
H	0.300847	5.861348	-0.801664
H	-0.072792	4.217919	-1.404957
H	-0.011039	5.473404	2.139351
H	1.562652	4.770049	2.610201
H	1.510212	6.143042	1.464503
H	6.116496	-0.629217	-0.139615
H	5.109107	0.109826	-1.415327
H	6.608353	0.916338	-0.865780
H	6.328751	0.479252	2.137024
H	6.632352	2.107069	1.452728
H	5.273869	1.848016	2.587784
H	-4.336145	7.178396	2.985335
H	-3.988718	5.607949	3.799498
H	-2.647399	6.550858	3.049421

Table S101. Cartesian geometry of 3f-TS1 (30.8 kcal/mol) in Figure S146 in Angstrom [Å].

Atomtype	X Coordinates	Y Coordinates	Z Coordinates
C	2.356500	-1.673233	-3.787596
C	2.688829	-1.733661	-2.420890
C	4.036291	-1.535826	-2.041276
C	5.022331	-1.280677	-3.003307
C	4.677651	-1.204158	-4.365153
C	3.344441	-1.401910	-4.751681
P	1.433229	-1.959134	-1.062372
C	0.001850	-2.766322	-1.936879
Si	-1.648530	-2.977528	-0.964473
C	-2.829445	-4.052467	-2.049000
C	-2.096504	-5.097569	-2.918045
Ni	1.118397	-0.113768	0.130494
C	1.496063	1.058839	1.578513
N	1.488373	0.765042	2.937082
C	1.891495	1.862450	3.705376
C	2.153264	2.873145	2.835353
N	1.924019	2.377037	1.550514
C	1.463314	-0.543509	3.556604
C	2.717435	-1.177652	3.766992
C	2.725863	-2.404108	4.458982
C	1.538302	-2.972864	4.930716
C	0.316639	-2.313797	4.737591
C	0.249866	-1.074816	4.068461
C	2.340402	3.184988	0.419338
C	1.455267	4.165165	-0.090408
C	1.957286	5.064383	-1.050006
C	3.287055	4.999387	-1.476109
C	4.139603	4.016096	-0.962427
C	3.690910	3.086473	-0.007017
C	4.028062	-0.592262	3.241038
C	5.171484	-0.651511	4.272839
C	-1.071682	-0.322942	3.926426
C	-2.316240	-1.187112	4.177647
C	0.018310	4.277681	0.402687
C	-0.985167	4.647468	-0.704372
C	4.650038	2.045337	0.568555

C	5.612055	1.457464	-0.480985
Ge	-1.159520	0.034371	-0.026146
C	-2.404229	1.513633	0.444883
C	-3.429355	1.962080	-0.419550
C	-4.296846	3.004539	-0.074584
C	-4.182538	3.675090	1.178309
C	-3.133509	3.246659	2.042441
C	-2.278950	2.200540	1.671303
N	-5.042888	4.703464	1.534465
C	-6.089499	5.123254	0.618491
Si	0.039116	1.122929	-2.170689
C	1.454459	2.170467	-2.937903
C	1.128832	3.274979	-3.763152
C	2.099699	3.900209	-4.563775
C	3.422770	3.425887	-4.567807
C	3.766577	2.339480	-3.747178
C	2.795802	1.733595	-2.934127
N	-2.343870	-1.340515	-0.759017
C	-3.694827	-1.109613	-1.203762
C	-4.801148	-1.141657	-0.289983
C	-6.116025	-1.063840	-0.794166
C	-6.380305	-0.938025	-2.160682
C	-5.303818	-0.828420	-3.046783
C	-3.970468	-0.880250	-2.598174
C	-4.649399	-1.239304	1.229928
C	-5.227117	0.004462	1.942895
C	-2.872205	-0.590236	-3.622636
C	-3.089492	0.787584	-4.291016
C	2.198463	-3.410768	-0.181526
C	2.797433	-4.455470	-0.920344
C	3.305545	-5.588635	-0.268022
C	3.216562	-5.692147	1.131687
C	2.630886	-4.652427	1.870795
C	2.131995	-3.512280	1.218892
C	4.424456	-1.274066	1.916820
C	-1.145223	0.917687	4.848135
C	-0.100360	5.282879	1.569832
C	5.449387	2.617610	1.760254
C	-5.328920	-2.502323	1.806900
C	-2.707884	-1.655793	-4.727813
C	-1.182427	-3.889451	0.663927
C	-2.266342	-4.039161	1.740491
C	-0.558243	-5.271050	0.368211
C	-3.980066	-4.710350	-1.260394
C	-4.853843	5.400821	2.793643
H	2.505163	0.164982	-0.253150
H	1.979330	1.798190	4.789032
H	2.499759	3.892500	2.995924
H	0.356204	-3.728293	-2.350239
H	-0.256727	-2.113824	-2.789079
H	-3.308954	-3.331065	-2.732777
H	-0.395864	-3.239489	1.094225
H	-1.162626	1.939617	-2.568580
H	0.023191	-0.015198	-3.156829
H	0.094179	3.642116	-3.799182
H	1.818750	4.754481	-5.195034
H	4.180601	3.903769	-5.203188
H	4.796556	1.960174	-3.732365
H	3.078780	0.891520	-2.294896
H	1.290751	5.821239	-1.476440

H	3.655080	5.700110	-2.234829
H	5.171419	3.961169	-1.324336
H	3.676488	-2.925601	4.615935
H	1.561717	-3.934801	5.459042
H	-0.598598	-2.768810	5.128016
H	4.312917	-1.569326	-0.981247
H	6.062006	-1.127292	-2.686826
H	5.445145	-0.986104	-5.118620
H	3.062068	-1.337016	-5.810101
H	1.323091	-1.819190	-4.116794
H	2.867917	-4.381726	-2.013062
H	3.768275	-6.393956	-0.853259
H	3.605763	-6.581707	1.643867
H	2.559103	-4.720388	2.961716
H	1.692636	-2.693357	1.800321
H	-2.824436	-5.665043	-3.532737
H	-1.372713	-4.635618	-3.614725
H	-1.542274	-5.833546	-2.306270
H	-4.688480	-5.204508	-1.955720
H	-3.614452	-5.487331	-0.563728
H	-4.555253	-3.966820	-0.682406
H	-1.821763	-4.410091	2.686576
H	-2.764205	-3.084907	1.954315
H	-3.047390	-4.760770	1.444692
H	-0.102261	-5.692570	1.285150
H	-1.330653	-5.985726	0.026519
H	0.236312	-5.238760	-0.395588
H	-1.108043	0.017388	2.874358
H	-3.217013	-0.621637	3.889486
H	-2.298271	-2.118212	3.593901
H	-2.417464	-1.450066	5.248028
H	-2.134274	1.400280	4.742626
H	-1.020446	0.617921	5.906199
H	-0.382175	1.674490	4.609068
H	3.861870	0.474501	3.018308
H	6.047602	-0.094612	3.891973
H	4.870239	-0.204204	5.238290
H	5.503326	-1.688765	4.465168
H	5.361651	-0.839731	1.524905
H	4.572748	-2.359780	2.055539
H	3.626584	-1.126586	1.169804
H	-5.498953	-0.684597	-4.117368
H	-7.414041	-0.902583	-2.528762
H	-6.957310	-1.108391	-0.091083
H	-1.924258	-0.545528	-3.069854
H	-3.569111	-1.285705	1.450629
H	-5.127602	-0.100211	3.039903
H	-4.718532	0.928389	1.635331
H	-6.305849	0.118157	1.726163
H	-5.087466	-2.612029	2.881075
H	-6.429305	-2.430541	1.718626
H	-5.010989	-3.421990	1.291662
H	-1.956907	-1.316633	-5.466708
H	-2.369050	-2.626825	-4.333862
H	-3.659488	-1.822572	-5.267994
H	-2.187781	1.079177	-4.860584
H	-3.943844	0.760542	-4.994088
H	-3.285138	1.579625	-3.548602
H	-1.477415	1.936075	2.368845
H	-2.973316	3.728739	3.011198

H	-5.068213	3.292918	-0.794400
H	-3.574049	1.486387	-1.393009
H	4.034087	1.210423	0.936510
H	-0.270962	3.285280	0.782554
H	-2.012710	4.518999	-0.325702
H	-0.870701	5.700672	-1.024954
H	-0.867831	4.001244	-1.588697
H	-1.149800	5.333958	1.914954
H	0.526335	4.996052	2.432083
H	0.209769	6.293848	1.242041
H	6.186663	0.622472	-0.039625
H	5.065875	1.071395	-1.355197
H	6.347607	2.204740	-0.832571
H	6.112997	1.842241	2.188124
H	6.079239	3.466508	1.432317
H	4.784578	2.975009	2.565112
H	-6.686168	5.927417	1.079109
H	-5.679542	5.509080	-0.339993
H	-6.779385	4.289118	0.372056
H	-5.617990	6.187557	2.902543
H	-4.945836	4.717506	3.664729
H	-3.855438	5.885860	2.857344

Table S102. Cartesian geometry of 3g-TS1 (38.1 kcal/mol) in Figure S146 in Angstrom [Å].

Atomtype	X Coordinates	Y Coordinates	Z Coordinates
C	2.453358	0.266536	3.537417
C	1.160357	-0.265072	3.328006
C	0.789845	-1.431688	4.023941
C	1.697122	-2.063858	4.893245
C	2.978446	-1.530572	5.091993
C	3.351049	-0.354829	4.415621
P	0.067699	0.572652	2.071526
C	-1.638052	0.033157	2.572351
Si	-3.114630	0.446475	1.404726
C	-4.727951	-0.016410	2.362790
C	-4.624134	0.182110	3.891194
Ni	0.794005	0.293473	-0.014684
C	1.890025	0.768918	-1.492017
N	1.903548	1.978068	-2.173014
C	2.852379	1.990021	-3.197502
C	3.482036	0.785301	-3.155604
N	2.905622	0.055311	-2.112069
C	1.262786	3.212393	-1.781919
C	1.994278	4.111796	-0.961123
C	1.392878	5.347098	-0.654774
C	0.120952	5.676750	-1.142923
C	-0.560198	4.789057	-1.985173
C	0.012097	3.552938	-2.345007
C	3.585394	-1.150246	-1.669946
C	3.257157	-2.397950	-2.252946
C	4.084110	-3.497407	-1.952189
C	5.205008	-3.358263	-1.128814
C	5.500931	-2.118065	-0.554982
C	4.694536	-0.991817	-0.796993
C	3.369688	3.758957	-0.398644
C	4.372265	4.927009	-0.467198
C	-0.642051	2.665925	-3.391319
C	-2.123367	2.401711	-3.105566
C	2.071616	-2.546892	-3.201735
C	1.456216	-3.957476	-3.199773

C	5.035694	0.346446	-0.143100
C	5.439345	0.195005	1.336561
Ge	-1.163405	-0.590136	-0.904307
N	-2.923638	-0.618802	-0.021565
C	-4.052350	-1.447387	-0.374434
C	-5.016104	-1.026506	-1.350114
C	-6.179744	-1.795948	-1.557023
C	-6.429258	-2.968390	-0.838962
C	-5.467429	-3.414543	0.072974
C	-4.276977	-2.698875	0.300921
C	-4.843316	0.218847	-2.216814
C	-4.630129	-0.156273	-3.698556
C	-3.231514	-3.343734	1.208672
C	-2.833596	-4.745524	0.692269
Si	0.222463	-2.584432	0.304954
C	1.925785	-3.356174	0.750979
C	2.115321	-4.748497	0.563802
C	3.226206	-5.415183	1.107586
C	4.170713	-4.704767	1.868391
C	4.004781	-3.323048	2.055336
C	2.905491	-2.659512	1.488659
N	-1.556895	-1.375542	-2.576544
C	-0.913680	-0.929073	-3.794606
C	0.103913	2.310607	2.738433
C	0.073751	2.536839	4.132804
C	0.007099	3.842527	4.641081
C	-0.036563	4.937660	3.759845
C	0.002204	4.717804	2.374229
C	0.082191	3.411066	1.864708
C	3.234479	3.216312	1.037308
C	-0.427347	3.251568	-4.803654
C	2.459532	-2.159276	-4.647704
C	6.133761	1.099614	-0.925021
C	-2.184902	-2.681253	-2.703580
C	-6.026954	1.204769	-2.107110
C	-3.637880	-3.453022	2.693663
C	-3.027315	2.341726	1.064375
C	-3.977818	2.965054	0.027295
C	-3.151132	3.128490	2.389827
C	-6.003303	0.654660	1.814135
H	2.089164	0.214831	0.652739
H	2.994573	2.864151	-3.831951
H	4.297185	0.375684	-3.749736
H	-1.813157	0.395478	3.601460
H	-1.625112	-1.070404	2.606127
H	-4.854533	-1.097474	2.179442
H	-1.998521	2.477495	0.675767
H	-0.416938	-3.720313	-0.452005
H	-0.429275	-2.658245	1.661269
H	1.369107	-5.330165	0.005125
H	3.346893	-6.495533	0.948021
H	5.033086	-5.224713	2.306857
H	4.738532	-2.752033	2.638257
H	2.796113	-1.579347	1.631576
H	3.845400	-4.482114	-2.364399
H	5.832450	-4.229422	-0.906335
H	6.363756	-2.029311	0.113578
H	1.923673	6.055854	-0.009844
H	-0.334864	6.639369	-0.877411
H	-1.537996	5.068196	-2.394605

H	2.762642	1.171503	3.002339
H	4.350377	0.074458	4.562951
H	3.686459	-2.029087	5.766082
H	1.397022	-2.984572	5.409292
H	-0.202045	-1.872365	3.885593
H	0.103384	1.686256	4.825560
H	-0.015837	4.005348	5.726462
H	-0.099372	5.960026	4.154622
H	-0.029709	5.563648	1.679243
H	0.132805	3.243619	0.782608
H	-5.572694	-0.117539	4.381164
H	-3.819361	-0.426266	4.343520
H	-4.435009	1.236872	4.164446
H	-6.899313	0.257427	2.332596
H	-5.997154	1.749353	1.969744
H	-6.135155	0.455905	0.737278
H	-3.707953	4.027815	-0.141380
H	-3.938312	2.459809	-0.945708
H	-5.027335	2.947042	0.368444
H	-2.883211	4.191360	2.233857
H	-4.192269	3.103781	2.762876
H	-2.494757	2.750077	3.190931
H	-0.129537	1.693038	-3.354139
H	-2.525712	1.668312	-3.822057
H	-2.236412	1.986335	-2.092252
H	-2.738053	3.317917	-3.175565
H	-0.857100	2.576780	-5.567955
H	-0.919583	4.238110	-4.899978
H	0.647500	3.385878	-5.026656
H	3.787814	2.946374	-1.017128
H	5.379524	4.571750	-0.180530
H	4.431846	5.352644	-1.486027
H	4.104762	5.743031	0.229651
H	4.222497	2.923706	1.434291
H	2.798886	3.976658	1.709202
H	2.572784	2.333959	1.043222
H	-5.635206	-4.352383	0.618004
H	-7.353325	-3.538214	-1.001918
H	-6.913286	-1.458546	-2.300268
H	-2.338733	-2.706560	1.163503
H	-3.931914	0.723050	-1.865747
H	-4.558834	0.754337	-4.323311
H	-3.705124	-0.736266	-3.821390
H	-5.475873	-0.756904	-4.083126
H	-5.795960	2.139422	-2.652555
H	-6.945571	0.780251	-2.553989
H	-6.252007	1.471492	-1.062343
H	-2.850493	-3.986846	3.259510
H	-3.777635	-2.468211	3.167228
H	-4.580405	-4.021562	2.810372
H	-1.938700	-5.107974	1.231088
H	-3.648291	-5.477244	0.852611
H	-2.594776	-4.732372	-0.384567
H	4.120876	0.958560	-0.161647
H	1.298104	-1.845898	-2.845599
H	0.525184	-3.956851	-3.792790
H	2.133732	-4.698312	-3.664335
H	1.214905	-4.295847	-2.179283
H	1.606905	-2.320594	-5.333247
H	2.758702	-1.101689	-4.731935

H	3.301219	-2.786944	-4.997874
H	5.563373	1.190217	1.800891
H	4.673427	-0.354943	1.905245
H	6.402954	-0.335146	1.449618
H	6.338748	2.080554	-0.455253
H	7.075114	0.517737	-0.930505
H	5.836477	1.281150	-1.972137
H	-0.426674	-1.768689	-4.334908
H	-1.626265	-0.463352	-4.516902
H	-0.123746	-0.185144	-3.586792
H	-1.444890	-3.482296	-2.928580
H	-2.708573	-2.965246	-1.780065
H	-2.944569	-2.695070	-3.516167

Table S103. Cartesian geometry of 3a-TS2 (42.8 kcal/mol) in Figure S146 in Angstrom [Å].

Atomtype	X Coordinates	Y Coordinates	Z Coordinates
C	1.073282	4.462848	3.791758
C	1.439475	3.678221	2.668143
C	2.070213	4.345412	1.592399
C	2.348773	5.721257	1.636452
C	1.982498	6.477136	2.762738
C	1.338682	5.842456	3.839906
Si	1.048513	1.796112	2.642599
Ge	1.331213	-0.374293	0.096019
C	2.423623	-1.498152	1.320854
C	3.832506	-1.530995	1.462437
C	4.460942	-2.532632	2.210824
C	3.691463	-3.544313	2.836081
C	2.282685	-3.522453	2.705172
C	1.668766	-2.512418	1.957062
C	4.329135	-4.584998	3.593157
N	4.842000	-5.436658	4.213760
Ni	-0.882266	-0.031194	0.077251
C	-2.346218	0.141780	1.302038
N	-3.246031	1.187986	1.454901
C	-4.041412	1.029018	2.593979
C	-3.667409	-0.142474	3.175474
N	-2.652101	-0.680885	2.378811
C	-3.628864	2.107726	0.405097
C	-4.691526	1.702048	-0.447825
C	-5.086534	2.583901	-1.472175
C	-4.478786	3.835876	-1.613323
C	-3.473598	4.235145	-0.722311
C	-3.019052	3.385215	0.306936
C	-2.135939	-2.007522	2.607599
C	-2.574110	-3.065384	1.757415
C	-2.087058	-4.358677	2.024384
C	-1.256144	-4.609411	3.127308
C	-0.898265	-3.566138	3.987012
C	-1.315105	-2.241513	3.740209
C	-5.491208	0.422334	-0.200279
C	-6.837920	0.772839	0.473603
C	-1.933473	3.833508	1.289461
C	-1.193203	5.108589	0.854075
C	-3.642460	-2.832025	0.685315
C	-5.029044	-2.739315	1.370676
C	-0.919194	-1.130677	4.710672
C	-1.712742	-1.255344	6.031177
P	-1.054448	-0.749378	-2.093143
C	0.168894	0.109189	-3.234808

Si	1.566112	1.217393	-2.521060
C	2.681228	1.498767	-4.067287
C	1.858687	2.073531	-5.246394
C	-2.539947	-0.828741	-3.234023
C	-3.198035	-2.033077	-3.573412
C	-4.342190	-2.023447	-4.389864
C	-4.854316	-0.812674	-4.883490
C	-4.221242	0.393566	-4.536558
C	-3.085536	0.386835	-3.714205
C	-0.474252	-2.523522	-2.159295
C	-0.385476	-3.241287	-0.949975
C	-0.005182	-4.592646	-0.937014
C	0.281481	-5.249782	-2.143839
C	0.200053	-4.544023	-3.358792
C	-0.167843	-3.188925	-3.366119
C	-5.723343	-0.424136	-1.461040
C	-2.495471	4.062658	2.712987
C	-3.709953	-3.902570	-0.418155
C	0.594325	-1.098948	5.000444
N	2.470702	0.371257	-1.222483
C	3.872203	0.080874	-1.325943
C	4.308693	-1.107203	-1.989542
C	5.690721	-1.364281	-2.088338
C	6.639887	-0.497458	-1.534982
C	6.206599	0.651061	-0.860464
C	4.838641	0.962743	-0.747501
C	3.335490	-2.157306	-2.525909
C	3.431242	-3.457015	-1.695601
C	4.429991	2.207407	0.034140
C	5.111189	3.493450	-0.478763
C	3.538858	-2.461970	-4.024352
C	4.702149	2.038111	1.544842
C	0.769684	2.815255	-1.799719
C	-0.663401	3.045678	-2.319211
C	1.632101	4.079886	-1.995749
C	3.989218	2.298015	-3.900851
H	0.694463	2.620700	-0.708846
H	-4.810467	1.751080	2.863085
H	-4.031458	-0.653107	4.065100
H	-2.817648	-2.990248	-3.202454
H	-1.179609	-0.165588	4.243779
H	-0.428140	0.757519	-3.898676
H	0.633552	-0.646679	-3.895415
H	4.522842	-2.930696	-4.213808
H	3.480840	-1.546359	-4.640475
H	2.760137	-3.164693	-4.375172
H	1.676552	4.374430	-3.061048
H	2.669035	3.946766	-1.645897
H	1.205186	4.933917	-1.435333
H	-0.901094	-5.629383	3.323991
H	6.946402	1.323669	-0.408636
H	2.309779	-1.770010	-2.408483
H	2.969224	0.461559	-4.337226
H	-1.199983	3.002689	1.342403
H	-0.645567	-2.728518	-0.017045
H	7.711234	-0.720483	-1.620040
H	-0.244766	-2.657251	-4.322950
H	6.028337	-2.276323	-2.596644
H	4.461102	-0.784853	0.973803
H	-5.894811	2.290840	-2.152009

H	-2.631858	1.344479	-3.435113
H	-2.382152	-5.190415	1.376720
H	3.340312	2.315945	-0.097966
H	5.552869	-2.542801	2.308163
H	-3.413645	-1.865788	0.197350
H	-4.923050	-0.202694	0.503342
H	0.575962	-2.518650	1.869701
H	-0.268323	-3.773654	4.859383
H	-4.799568	4.515749	-2.413398
H	-4.832531	-2.972794	-4.642890
H	-1.687930	4.425840	3.372934
H	-2.901516	3.144306	3.162757
H	-3.294513	4.828530	2.690377
H	-1.056998	4.019898	-1.980601
H	-1.357119	2.275488	-1.935722
H	-0.715712	3.048335	-3.425380
H	-3.029058	5.227107	-0.841292
H	4.142078	1.188815	1.968345
H	4.397216	2.945351	2.096450
H	5.779690	1.866304	1.729579
H	-5.745300	-0.807523	-5.524162
H	-4.620766	1.350634	-4.896536
H	2.707814	-4.202423	-2.066453
H	3.215201	-3.268999	-0.630418
H	4.445339	-3.894040	-1.763872
H	0.427580	-5.051502	-4.305123
H	0.925507	-1.995847	5.556634
H	0.840251	-0.212841	5.612820
H	1.183027	-1.039562	4.071644
H	0.578181	-6.306350	-2.141055
H	0.054687	-5.125679	0.019589
H	-6.285844	0.124129	-2.237087
H	-6.311896	-1.325200	-1.209157
H	-4.770182	-0.748455	-1.900989
H	-1.878052	5.977388	0.828811
H	-0.725895	5.004354	-0.138376
H	-0.397705	5.341878	1.575990
H	-2.730464	-4.079917	-0.888602
H	-4.410485	-3.567991	-1.203831
H	-4.088100	-4.866637	-0.028047
H	-2.804872	-1.247603	5.863269
H	-1.463434	-0.418382	6.709986
H	-1.462348	-2.200565	6.549407
H	4.562210	2.285125	-4.850012
H	4.639695	1.879126	-3.116981
H	3.792415	3.357338	-3.658308
H	6.197014	3.483413	-0.268114
H	4.685962	4.376115	0.033580
H	4.982013	3.623341	-1.566262
H	1.675890	-4.295573	3.187489
H	1.478261	3.088013	-5.019692
H	0.992145	1.443804	-5.516876
H	2.493307	2.162323	-6.150788
H	-6.693519	1.363719	1.395600
H	-7.387556	-0.150286	0.738344
H	-7.472772	1.366682	-0.211063
H	-5.254566	-3.689124	1.891403
H	-5.819038	-2.565310	0.619083
H	-5.079707	-1.923605	2.109696
H	2.193174	1.109598	3.352195

H	-0.112256	1.669871	3.605480
H	0.571849	3.988111	4.646647
H	1.043141	6.424704	4.723143
H	2.189541	7.554629	2.799318
H	2.837275	6.205028	0.780151
H	2.331638	3.786436	0.690514
H	0.089381	0.756520	1.213019

Table S104. Cartesian geometry of 3b-TS2 (44.6 kcal/mol) in Figure S146 in Angstrom [Å].

Atomtype	X Coordinates	Y Coordinates	Z Coordinates
C	-0.231226	4.342239	-4.129290
C	-0.566293	3.836378	-2.846895
C	-0.809589	4.782332	-1.823861
C	-0.749564	6.164594	-2.064756
C	-0.422511	6.642374	-3.344964
C	-0.158979	5.724154	-4.376799
Si	-0.638114	1.934894	-2.563325
Ge	-0.965153	0.047844	0.158583
C	-2.556693	-0.861856	-0.661538
C	-3.822604	-0.258492	-0.813188
C	-4.949801	-1.013816	-1.168861
C	-4.840371	-2.396139	-1.374920
C	-3.586239	-3.010588	-1.241218
C	-2.448327	-2.251073	-0.900142
C	-6.316840	-0.360767	-1.245039
F	-6.988549	-0.487927	-0.071769
C	-3.495000	-4.505001	-1.472105
F	-3.205210	-4.806402	-2.769207
Ni	1.246844	-0.128982	-0.097515
C	2.511904	-0.407857	-1.504019
N	3.575862	0.359095	-1.958247
C	4.105714	-0.146660	-3.150744
C	3.389999	-1.262722	-3.459170
N	2.440411	-1.421124	-2.446849
C	4.336465	1.278663	-1.139119
C	5.408696	0.727621	-0.384932
C	6.164164	1.600781	0.421261
C	5.891109	2.972392	0.442480
C	4.866654	3.498495	-0.355882
C	4.061085	2.670640	-1.165092
C	1.610602	-2.596400	-2.348698
C	2.006685	-3.636430	-1.459309
C	1.223975	-4.805891	-1.436451
C	0.115235	-4.953444	-2.281950
C	-0.234491	-3.928718	-3.167293
C	0.499839	-2.727483	-3.217724
C	5.842599	-0.732137	-0.527540
C	6.064133	-1.452936	0.811408
C	2.954257	3.255869	-2.047165
C	3.289483	3.144732	-3.553947
C	3.290695	-3.533678	-0.633786
C	3.319460	-4.411457	0.630474
C	0.130116	-1.649738	-4.232495
C	-1.372376	-1.310012	-4.223957
P	1.584532	-0.612044	2.100775
C	3.186493	-0.877968	3.032293
C	4.056088	0.227147	3.199896
C	5.286579	0.083561	3.856314
C	5.690795	-1.173021	4.339960
C	4.852543	-2.283839	4.151910

C	3.612183	-2.139520	3.506296
C	0.656538	-2.168529	2.539142
C	0.372695	-2.549696	3.868065
C	-0.300396	-3.752744	4.135538
C	-0.700626	-4.589758	3.077915
C	-0.423339	-4.219774	1.752135
C	0.250071	-3.016050	1.489310
C	0.771148	0.658266	3.216019
Si	-0.442037	1.975770	2.517648
C	0.566722	3.253265	1.486730
C	0.125717	4.717549	1.690734
C	-1.198353	2.659105	4.158156
C	-2.307521	3.725195	4.094782
N	-1.693756	1.235757	1.461343
C	-3.095186	1.333939	1.765909
C	-3.714949	0.341489	2.589864
C	-5.086867	0.459974	2.888200
C	-5.859808	1.509545	2.381569
C	-5.256911	2.465117	1.556072
C	-3.887226	2.405643	1.237302
C	-2.967346	-0.882517	3.123314
C	-2.914279	-0.921079	4.665210
C	-3.313867	3.481149	0.312477
C	-3.891085	3.400230	-1.119673
C	-0.085006	3.124358	5.130001
C	7.111720	-0.812664	-1.406767
C	2.625808	4.725839	-1.736661
C	4.506122	-3.888947	-1.524529
C	0.577602	-2.067040	-5.651555
C	-3.584779	-2.193088	2.585262
C	-3.544609	4.913344	0.844957
F	-6.236481	0.961190	-1.521025
F	-7.089432	-0.933121	-2.203488
F	-2.531734	-5.083394	-0.709281
F	-4.660491	-5.131669	-1.184040
C	2.086818	3.111492	1.688475
H	0.321777	0.782957	-1.171573
H	0.353024	2.978607	0.432477
H	4.955141	0.321634	-3.645369
H	3.471096	-1.965001	-4.287183
H	2.973597	-3.019333	3.376043
H	0.674789	-0.729781	-3.959761
H	1.591866	1.224869	3.687869
H	0.257687	0.132295	4.042151
H	-3.929651	-0.994038	5.098380
H	-2.437926	-0.016036	5.082160
H	-2.335782	-1.800684	5.002835
H	0.368190	5.074705	2.709175
H	-0.956065	4.861056	1.539164
H	0.647571	5.382936	0.975575
H	-0.483638	-5.870362	-2.247426
H	-5.865956	3.276774	1.139515
H	-1.925023	-0.836670	2.763696
H	-1.655618	1.747397	4.593985
H	2.046929	2.646295	-1.856088
H	0.487342	-2.724444	0.458936
H	-6.930396	1.572457	2.613088
H	0.696616	-1.917528	4.704405
H	-5.563074	-0.301064	3.519130
H	-3.955233	0.806412	-0.636517

H	6.988789	1.199804	1.021940
H	3.782493	1.209177	2.799404
H	1.489645	-5.619722	-0.753944
H	-2.227499	3.297429	0.252437
H	3.380300	-2.482069	-0.303948
H	5.045093	-1.275518	-1.053756
H	-1.483866	-2.760819	-0.800790
H	-1.099709	-4.058130	-3.823748
H	6.490139	3.642870	1.072491
H	5.160495	-3.273657	4.514046
H	2.483386	3.614275	-4.145097
H	3.383362	2.102288	-3.892968
H	4.235328	3.675357	-3.776168
H	2.635286	3.894365	1.137291
H	2.453101	2.140510	1.306282
H	2.380446	3.205872	2.751955
H	4.686972	4.576937	-0.336429
H	-3.630662	2.461309	-1.631532
H	-3.490090	4.223380	-1.736913
H	-4.992907	3.483443	-1.102993
H	6.656372	-1.287223	4.848899
H	5.939929	0.957753	3.974485
H	-2.957499	-3.055496	2.872042
H	-3.666900	-2.177577	1.486673
H	-4.598979	-2.353486	2.996954
H	-0.513849	-4.038002	5.173791
H	-1.989717	-2.161831	-4.565176
H	-1.569928	-0.457189	-4.897471
H	-1.713093	-1.026724	-3.215628
H	-1.232148	-5.526476	3.289023
H	-0.728027	-4.860729	0.918687
H	6.849186	-0.972752	1.421610
H	6.383717	-2.495404	0.631122
H	5.141020	-1.475416	1.407540
H	-5.724202	-2.989813	-1.627621
H	3.491197	5.380705	-1.952990
H	2.326938	4.879200	-0.686669
H	1.794826	5.063013	-2.372380
H	2.422337	-4.268088	1.253495
H	4.203548	-4.146896	1.237426
H	3.399314	-5.486435	0.380421
H	1.660908	-2.279491	-5.697462
H	0.354113	-1.263175	-6.377898
H	0.042707	-2.980290	-5.975183
H	-2.694749	3.927292	5.113938
H	-3.160287	3.406777	3.475028
H	-1.924924	4.683769	3.701305
H	-4.616696	5.181784	0.801589
H	-2.999166	5.641123	0.215890
H	-3.212848	5.034764	1.887196
H	0.476598	3.987371	4.724193
H	0.645667	2.331041	5.366918
H	-0.529964	3.453800	6.090206
H	6.962732	-0.320154	-2.384393
H	7.386198	-1.868635	-1.590739
H	7.965536	-0.317464	-0.906559
H	4.411623	-4.927784	-1.893438
H	5.444450	-3.814657	-0.946726
H	4.592813	-3.221704	-2.397490
H	-2.035393	1.500169	-2.951195

H	0.227001	1.395803	-3.682025
H	-0.023707	3.643844	-4.951911
H	0.102857	6.085914	-5.380327
H	-0.365765	7.721797	-3.536936
H	-0.943661	6.868405	-1.244323
H	-1.033315	4.438014	-0.811310

Table S105. Cartesian geometry of 3c-TS2 (41.9 kcal/mol) in Figure S146 in Angstrom [Å].

Atomtype	X Coordinates	Y Coordinates	Z Coordinates
C	3.458318	-1.372471	2.522131
C	2.087471	-1.305316	2.179684
C	1.162527	-2.001461	2.992099
C	1.587769	-2.744088	4.105646
C	2.952192	-2.802940	4.430984
C	3.883799	-2.109453	3.638767
Ge	1.284063	-0.548064	0.531472
Ni	-0.831708	0.013644	0.074575
P	-0.965662	-1.431456	-1.675049
C	0.458555	-1.218378	-2.881643
Si	1.961325	-0.103660	-2.440181
C	3.211007	-0.576731	-3.831129
C	2.574240	-0.402940	-5.231914
Si	1.254571	2.462462	1.970322
C	1.936625	4.165555	1.384789
C	2.737602	4.312158	0.228226
C	3.219728	5.565032	-0.184995
C	2.894054	6.719915	0.546046
C	2.086439	6.605663	1.691655
C	1.617602	5.346025	2.103747
N	2.618562	-0.496359	-0.819222
C	3.956452	-0.980502	-0.632475
C	4.233679	-2.378310	-0.746733
C	5.558725	-2.827431	-0.578765
C	6.601250	-1.942787	-0.281067
C	6.320421	-0.578094	-0.139097
C	5.017432	-0.074744	-0.312674
C	3.138781	-3.422396	-0.969412
C	2.987798	-4.327658	0.273990
C	4.776166	1.418765	-0.108284
C	5.680168	2.302007	-0.994622
C	3.371710	-4.280123	-2.230460
C	4.952121	1.824946	1.371519
C	-2.299038	0.851104	0.971603
N	-3.016708	1.994434	0.643812
C	-3.884864	2.376461	1.671852
C	-3.741368	1.462728	2.669587
N	-2.791605	0.534848	2.231400
C	-3.189915	2.499352	-0.701324
C	-4.251644	1.940999	-1.464592
C	-4.443430	2.418515	-2.775318
C	-3.639123	3.440939	-3.289595
C	-2.637173	4.014983	-2.495820
C	-2.382382	3.561495	-1.184962
C	-2.509900	-0.669240	2.972510
C	-3.084135	-1.895223	2.525088
C	-2.836298	-3.048666	3.292474
C	-2.103672	-2.982271	4.487659
C	-1.602329	-1.756439	4.936125
C	-1.779359	-0.576746	4.184115
C	-5.253805	0.954378	-0.864450

C	-6.567519	1.692666	-0.519184
C	-1.299358	4.207453	-0.316623
C	-0.338157	5.117488	-1.099014
C	-4.043337	-1.933172	1.332576
C	-5.429597	-1.409708	1.784387
C	-1.230284	0.747619	4.709543
C	-2.078437	1.254606	5.897836
C	-5.536633	-0.266613	-1.752924
C	-1.902207	5.028361	0.848440
C	-4.225117	-3.315257	0.680686
C	0.255151	0.662895	5.111756
C	-0.689753	-3.165608	-1.042370
C	-0.806784	-3.390492	0.343000
C	-0.654327	-4.677166	0.882979
C	-0.391605	-5.763935	0.034213
C	-0.269896	-5.553202	-1.352070
C	-0.411900	-4.262478	-1.886407
C	-2.365812	-1.725337	-2.886602
C	-2.669251	-0.709346	-3.825615
C	-3.735802	-0.851608	-4.724607
C	-4.541136	-2.003527	-4.695059
C	-4.270653	-3.005806	-3.749376
C	-3.195640	-2.869612	-2.854100
C	1.390524	1.735311	-2.459125
C	0.038098	1.926905	-3.172296
C	2.448730	2.707448	-3.021523
C	4.617609	0.052938	-3.813716
H	1.234405	1.987224	-1.388874
H	-4.536846	3.243656	1.580956
H	-4.229449	1.371800	3.638298
H	-3.005205	-3.666007	-2.127295
H	-1.302945	1.489413	3.896170
H	0.024610	-0.800524	-3.806066
H	0.844789	-2.217314	-3.158406
H	4.286049	-4.896200	-2.138131
H	3.479550	-3.656823	-3.136552
H	2.518018	-4.966380	-2.383231
H	2.597030	2.557802	-4.107562
H	3.431254	2.593721	-2.534676
H	2.130747	3.757737	-2.874090
H	-1.934768	-3.893356	5.076445
H	7.131526	0.116685	0.112889
H	2.180561	-2.894200	-1.107751
H	3.344704	-1.664880	-3.660186
H	-0.712939	3.374949	0.123426
H	-1.043014	-2.538878	0.990898
H	7.625485	-2.315551	-0.150526
H	-0.331734	-4.116503	-2.971026
H	5.773163	-3.900071	-0.667438
H	4.210999	-0.871342	1.911661
H	-5.246905	1.995775	-3.389507
H	-2.079278	0.213501	-3.849734
H	-3.241646	-4.011465	2.964957
H	3.724685	1.607596	-0.383941
H	4.953463	-2.148279	3.882918
H	-3.632044	-1.255014	0.561319
H	-4.832960	0.573767	0.076988
H	0.091897	-1.964116	2.762686
H	-1.041827	-1.710452	5.876434
H	-3.801588	3.807120	-4.311785

H	-4.897844	-3.906176	-3.706542
H	-1.087983	5.509082	1.419019
H	-2.476592	4.407610	1.552221
H	-2.565284	5.822727	0.454686
H	-0.220732	2.996848	-3.254676
H	-0.782420	1.446631	-2.608255
H	0.043618	1.520128	-4.202385
H	-2.035548	4.826545	-2.914454
H	4.250154	1.290196	2.030861
H	4.766321	2.906608	1.497941
H	5.981655	1.608145	1.715006
H	-5.378626	-2.113435	-5.395754
H	-3.946160	-0.048564	-5.443025
H	2.165088	-5.047334	0.123804
H	2.767709	-3.734832	1.177419
H	3.917228	-4.898689	0.460012
H	-0.060070	-6.398846	-2.019777
H	0.405875	0.009020	5.991189
H	0.630357	1.669349	5.370187
H	0.875120	0.272360	4.289631
H	-0.270752	-6.772919	0.448852
H	-0.748470	-4.820382	1.966206
H	-5.962386	0.016272	-2.731761
H	-6.268615	-0.932477	-1.260423
H	-4.619195	-0.842481	-1.937874
H	3.289848	-3.387235	5.297148
H	-0.869951	5.993769	-1.516061
H	0.162212	4.587610	-1.925631
H	0.442286	5.499882	-0.425816
H	-3.263048	-3.781222	0.416594
H	-4.815684	-3.203023	-0.245984
H	-4.777617	-4.009274	1.342437
H	-3.145339	1.363298	5.631409
H	-1.708460	2.238677	6.241844
H	-2.017482	0.550479	6.749676
H	5.242325	-0.396990	-4.611834
H	5.137177	-0.110627	-2.856541
H	4.580808	1.139438	-4.008561
H	6.739023	2.228548	-0.682962
H	5.383350	3.362786	-0.899037
H	5.624449	2.017412	-2.058079
H	0.845521	-3.272514	4.716131
H	2.338101	0.657748	-5.443036
H	1.643589	-0.984262	-5.359494
H	3.279167	-0.738500	-6.018883
H	-6.387562	2.560414	0.140280
H	-7.269839	1.009026	-0.005531
H	-7.059195	2.063916	-1.438438
H	-5.834681	-2.060342	2.582482
H	-6.140539	-1.421676	0.939515
H	-5.383463	-0.379604	2.173012
H	2.216825	1.971428	3.029097
H	0.021941	2.850023	2.762509
H	0.989980	5.280800	3.003519
H	1.821446	7.502468	2.268099
H	3.259908	7.703276	0.222710
H	3.836671	5.638965	-1.090631
H	2.973191	3.433999	-0.378297
H	0.214617	1.052081	0.918988

Table S106. Cartesian geometry of 3d-TS2 (41.8 kcal/mol) in Figure S146 in Angstrom [Å].

Atomtype	X Coordinates	Y Coordinates	Z Coordinates
C	1.312613	4.994749	3.015657
C	1.664346	4.017547	2.049548
C	2.360153	4.466626	0.902797
C	2.711982	5.815485	0.730566
C	2.356388	6.764706	1.703610
C	1.650608	6.348863	2.846740
Si	1.159943	2.176058	2.303716
Ge	1.308917	-0.465451	0.267739
C	2.290623	-1.467292	1.663892
C	3.685097	-1.487624	1.905183
C	4.243927	-2.398809	2.810505
C	3.443037	-3.333107	3.503678
C	2.055816	-3.312543	3.266304
C	1.490576	-2.396613	2.364830
C	4.070556	-4.315645	4.467648
Ni	-0.873544	-0.023147	0.069021
C	-2.325400	0.478664	1.208267
N	-3.150094	1.595500	1.168658
C	-3.962732	1.682962	2.303803
C	-3.674316	0.603210	3.079701
N	-2.693925	-0.128345	2.402450
C	-3.463494	2.342992	-0.030068
C	-4.535059	1.856153	-0.827884
C	-4.862797	2.568493	-1.997506
C	-4.181077	3.742311	-2.335344
C	-3.168529	4.233304	-1.500491
C	-2.779538	3.549890	-0.329812
C	-2.265650	-1.427279	2.858858
C	-2.782221	-2.584233	2.205076
C	-2.388089	-3.841819	2.699080
C	-1.565979	-3.952441	3.831072
C	-1.120391	-2.802336	4.489662
C	-1.445759	-1.516072	4.012178
C	-5.412170	0.684715	-0.384415
C	-6.743826	1.221504	0.189091
C	-1.685187	4.103067	0.587107
C	-0.861267	5.237128	-0.045001
C	-3.827569	-2.467076	1.092742
C	-5.209670	-2.172624	1.727319
C	-0.954173	-0.280782	4.762790
C	-1.744003	-0.093980	6.077988
P	-1.035360	-1.086616	-1.931468
C	0.269439	-0.505681	-3.152994
Si	1.712834	0.628728	-2.582015
C	2.882074	0.562272	-4.114739
C	2.128976	0.961240	-5.407486
C	-2.499640	-1.255224	-3.090218
C	-3.233623	-2.453271	-3.248153
C	-4.361741	-2.502111	-4.085288
C	-4.781935	-1.357266	-4.781599
C	-4.072610	-0.154614	-4.617702
C	-2.953051	-0.101465	-3.775248
C	-0.580989	-2.881164	-1.691827
C	-0.575929	-3.391356	-0.379243
C	-0.286625	-4.741830	-0.128573
C	-0.007258	-5.606257	-1.198723
C	-0.006182	-5.109132	-2.515516
C	-0.285078	-3.754894	-2.760370

C	-5.677353	-0.351557	-1.487101
C	-2.256236	4.612137	1.932215
C	-3.954176	-3.699641	0.180013
C	0.559550	-0.316809	5.050745
N	2.524274	-0.022231	-1.122826
C	3.904928	-0.412505	-1.120624
C	4.277995	-1.726877	-1.541433
C	5.642129	-2.079968	-1.548538
C	6.634145	-1.184782	-1.133026
C	6.262185	0.091313	-0.691898
C	4.915367	0.500252	-0.680030
C	3.251785	-2.797948	-1.913095
C	3.260478	-3.944096	-0.876500
C	4.577311	1.892621	-0.153486
C	5.339877	3.015651	-0.888485
C	3.455431	-3.360798	-3.335039
C	4.835663	2.003630	1.365226
C	1.004707	2.373928	-2.181718
C	-0.408194	2.584773	-2.759211
C	1.941975	3.530348	-2.588462
C	4.235619	1.296717	-4.049859
H	0.911537	2.387670	-1.075159
H	-4.682940	2.488946	2.434342
H	-4.078297	0.275402	4.035849
H	-2.925639	-3.360144	-2.717585
H	-1.141149	0.598450	4.123076
H	-0.266317	0.048905	-3.942185
H	0.708091	-1.388780	-3.654328
H	4.417095	-3.900728	-3.423353
H	3.450623	-2.560997	-4.097602
H	2.646051	-4.074297	-3.577921
H	2.019987	3.618604	-3.688444
H	2.963683	3.407704	-2.192894
H	1.557944	4.496184	-2.206849
H	-1.282121	-4.944674	4.205491
H	7.034896	0.790128	-0.347385
H	2.249043	-2.340077	-1.885399
H	3.108197	-0.521885	-4.184073
H	-1.003458	3.255936	0.807054
H	-0.825464	-2.712539	0.444038
H	7.690635	-1.482808	-1.143377
H	-0.297670	-3.385452	-3.793583
H	5.930004	-3.088315	-1.872342
H	4.353815	-0.809165	1.373129
H	-5.676159	2.207361	-2.637427
H	-2.439613	0.857008	-3.641040
H	-2.747027	-4.751190	2.206391
H	3.496152	2.037211	-0.319554
H	5.330439	-2.391930	2.974879
H	-3.531366	-1.612925	0.455249
H	-4.891396	0.159617	0.428790
H	0.405379	-2.411246	2.215964
H	-0.487714	-2.896622	5.379178
H	-4.449034	4.290722	-3.247928
H	-4.912372	-3.445936	-4.194286
H	-1.439113	5.039988	2.539489
H	-2.723687	3.811848	2.525095
H	-3.007534	5.405542	1.753833
H	-0.750935	3.622662	-2.604621
H	-1.145642	1.931412	-2.257968

H	-0.451078	2.394038	-3.849297
H	-2.665226	5.163956	-1.776762
H	4.231860	1.282219	1.938296
H	4.574808	3.014602	1.726321
H	5.902831	1.819023	1.593387
H	-5.660775	-1.398409	-5.437652
H	-4.399813	0.755254	-5.137636
H	2.485869	-4.689192	-1.125851
H	3.061715	-3.566256	0.140210
H	4.241765	-4.455543	-0.864323
H	0.216370	-5.779768	-3.355577
H	0.821276	-1.115077	5.770576
H	0.880426	0.647637	5.483590
H	1.142336	-0.485191	4.131612
H	0.220235	-6.663411	-1.010867
H	-0.287773	-5.109054	0.904824
H	-6.197720	0.085529	-2.357474
H	-6.319707	-1.162645	-1.098174
H	-4.739375	-0.798549	-1.844484
H	-1.490554	6.126613	-0.238568
H	-0.386561	4.933395	-0.991985
H	-0.063329	5.546155	0.645186
H	-2.982614	-4.014219	-0.231808
H	-4.620930	-3.456188	-0.666331
H	-4.400116	-4.559792	0.714932
H	-2.833018	-0.026139	5.903816
H	-1.420417	0.830172	6.592642
H	-1.568188	-0.945531	6.762933
H	4.829168	1.075578	-4.960094
H	4.837541	0.988850	-3.180436
H	4.103484	2.392562	-4.010688
H	6.422452	2.975604	-0.664423
H	4.972476	4.003690	-0.554836
H	5.222606	2.949743	-1.982595
H	1.400972	-4.019038	3.792193
H	1.806067	2.019693	-5.380051
H	1.232814	0.343075	-5.594981
H	2.792353	0.850182	-6.288686
H	-6.574633	1.951092	1.001054
H	-7.351838	0.390196	0.593694
H	-7.332633	1.726002	-0.600450
H	-5.501079	-3.004967	2.395666
H	-5.981468	-2.077148	0.943301
H	-5.211095	-1.242497	2.318231
H	2.233192	1.569446	3.180428
H	-0.035124	2.282815	3.230187
H	0.762890	4.692623	3.918043
H	1.363030	7.083161	3.611540
H	2.619943	7.822138	1.570209
H	3.249764	6.126260	-0.175256
H	2.613944	3.753828	0.114005
H	0.153289	0.912660	1.044242
H	3.320796	-5.000431	4.902842
H	4.843679	-4.928766	3.964938
H	4.574778	-3.787637	5.300760

Table S107. Cartesian geometry of 3e-TS2 (41.6 kcal/mol) in Figure S146 in Angstrom [Å].

Atomtype	X Coordinates	Y Coordinates	Z Coordinates
C	0.870480	4.674717	3.644435
C	1.254285	3.911202	2.512255

C	1.786416	4.618695	1.409001
C	1.951330	6.013426	1.432613
C	1.566616	6.747919	2.567001
C	1.021218	6.072170	3.673225
Si	1.001887	2.000691	2.497259
Ge	1.299299	-0.267784	0.063994
C	2.511471	-1.333763	1.197966
C	3.911848	-1.210463	1.331622
C	4.673343	-2.162502	2.028144
C	4.040309	-3.284180	2.607512
C	2.640118	-3.423002	2.486917
C	1.897708	-2.462870	1.796216
O	4.682411	-4.271271	3.292436
C	6.092093	-4.197085	3.443356
Ni	-0.930279	-0.096881	0.083971
C	-2.331060	0.018586	1.380848
N	-3.290841	1.002722	1.579485
C	-4.003969	0.801964	2.765936
C	-3.513561	-0.332984	3.333860
N	-2.512738	-0.809093	2.481446
C	-3.800036	1.875930	0.543838
C	-4.870383	1.376161	-0.247598
C	-5.386544	2.208412	-1.259390
C	-4.886453	3.501243	-1.447233
C	-3.870257	3.990719	-0.615829
C	-3.297687	3.195305	0.398303
C	-1.883609	-2.089373	2.691459
C	-2.305674	-3.195358	1.896766
C	-1.713995	-4.445933	2.155286
C	-0.788387	-4.610357	3.197367
C	-0.435337	-3.521596	4.000423
C	-0.960985	-2.235728	3.757917
C	-5.550626	0.040474	0.054478
C	-6.880839	0.291418	0.801281
C	-2.201083	3.743787	1.315355
C	-1.584210	5.063644	0.823407
C	-3.450962	-3.058024	0.890128
C	-4.798301	-3.052643	1.654441
C	-0.562613	-1.070224	4.660001
C	-1.244185	-1.193870	6.041501
P	-1.132927	-0.858392	-2.047806
C	-0.022899	0.073213	-3.244086
Si	1.313356	1.292461	-2.593663
C	2.345561	1.619698	-4.190105
C	1.440425	2.100479	-5.350761
C	-2.661986	-1.047580	-3.116358
C	-3.255228	-2.297053	-3.409565
C	-4.436824	-2.373795	-4.166967
C	-5.052501	-1.206122	-4.646156
C	-4.484464	0.043748	-4.343596
C	-3.310960	0.122775	-3.580212
C	-0.440772	-2.589479	-2.133045
C	-0.253005	-3.285684	-0.923441
C	0.223850	-4.605805	-0.914088
C	0.509559	-5.253100	-2.126488
C	0.328521	-4.568460	-3.342906
C	-0.137408	-3.243867	-3.346502
C	-5.783914	-0.836700	-1.184989
C	-2.706117	3.955508	2.762676
C	-3.502728	-4.147154	-0.195939

C	0.963797	-0.938504	4.828019
N	2.322528	0.542440	-1.317603
C	3.734775	0.345759	-1.471280
C	4.228659	-0.828709	-2.120272
C	5.619951	-0.988493	-2.276617
C	6.527084	-0.039698	-1.791619
C	6.041105	1.093197	-1.126841
C	4.660423	1.309570	-0.958523
C	3.313857	-1.961435	-2.587784
C	3.566141	-3.243535	-1.762670
C	4.201102	2.545680	-0.189676
C	4.756477	3.861637	-0.774636
C	3.448767	-2.260875	-4.095272
C	4.570346	2.448952	1.306896
C	0.432125	2.844145	-1.870412
C	-1.040709	2.946343	-2.311820
C	1.179311	4.165989	-2.145619
C	3.595281	2.516868	-4.095740
H	0.434481	2.676633	-0.772610
H	-4.804985	1.471282	3.075344
H	-3.785759	-0.855833	4.248992
H	-2.794102	-3.221952	-3.048023
H	-0.917658	-0.139756	4.185182
H	-0.690570	0.663604	-3.894565
H	0.475560	-0.655284	-3.910797
H	4.458651	-2.638252	-4.344198
H	3.268082	-1.360811	-4.710331
H	2.717390	-3.035011	-4.393376
H	1.147190	4.431385	-3.219122
H	2.239658	4.125013	-1.846417
H	0.714301	5.000491	-1.585897
H	-0.349038	-5.598249	3.387667
H	6.749149	1.830437	-0.727700
H	2.269087	-1.657623	-2.408045
H	2.700671	0.600288	-4.446858
H	-1.403181	2.973436	1.343961
H	-0.508954	-2.778550	0.013606
H	7.607183	-0.186417	-1.922360
H	-0.290155	-2.728603	-4.303240
H	6.000039	-1.888267	-2.777070
H	4.444886	-0.375331	0.874294
H	-6.203496	1.842772	-1.892143
H	-2.908731	1.111459	-3.333020
H	-1.996805	-5.312009	1.548259
H	3.100358	2.569161	-0.260227
H	5.756361	-2.020927	2.100007
H	-3.320585	-2.086028	0.378271
H	-4.895807	-0.528866	0.729333
H	0.812697	-2.597937	1.731065
H	0.280899	-3.659911	4.817828
H	-5.300193	4.141548	-2.237307
H	-4.875243	-3.356722	-4.384973
H	-1.896793	4.392530	3.373788
H	-3.014111	3.016477	3.246290
H	-3.563524	4.655943	2.770807
H	-1.496004	3.890403	-1.965302
H	-1.645003	2.127925	-1.879464
H	-1.152963	2.923840	-3.413450
H	-3.510898	5.011769	-0.771020
H	4.108934	1.570947	1.786322

H	4.218665	3.345151	1.848816
H	5.667527	2.375760	1.433552
H	-5.973041	-1.268220	-5.240468
H	-4.964498	0.967671	-4.691735
H	2.870668	-4.040805	-2.075577
H	3.419884	-3.061437	-0.685026
H	4.600039	-3.609639	-1.910104
H	0.554943	-5.068533	-4.293525
H	1.390210	-1.793138	5.386136
H	1.199634	-0.015993	5.388175
H	1.474104	-0.881415	3.853694
H	0.883074	-6.285134	-2.127622
H	0.363649	-5.120304	0.044248
H	-6.437624	-0.347377	-1.928498
H	-6.275742	-1.782402	-0.892481
H	-4.834403	-1.082565	-1.680235
H	-2.333581	5.877986	0.823793
H	-1.163806	4.977754	-0.191641
H	-0.770947	5.368256	1.497550
H	-2.540186	-4.260476	-0.718618
H	-4.268078	-3.875591	-0.944731
H	-3.787310	-5.130198	0.225358
H	-2.344610	-1.251618	5.959153
H	-0.992616	-0.321488	6.673412
H	-0.900443	-2.105878	6.566177
H	4.133108	2.518555	-5.065544
H	4.302843	2.169411	-3.326710
H	3.328793	3.565497	-3.874153
H	5.850990	3.932687	-0.630335
H	4.301074	4.726775	-0.258311
H	4.555035	3.952968	-1.854646
H	2.154692	-4.290078	2.947231
H	0.988009	3.086887	-5.132214
H	0.618197	1.398651	-5.578416
H	2.035361	2.218170	-6.278747
H	-6.730809	0.900450	1.710510
H	-7.341050	-0.668981	1.101785
H	-7.597416	0.827697	0.150475
H	-4.925231	-4.007700	2.198645
H	-5.641355	-2.945999	0.949119
H	-4.862891	-2.230805	2.385640
H	2.210680	1.423571	3.201712
H	-0.114722	1.807725	3.505049
H	0.443882	4.167845	4.521300
H	0.712703	6.636735	4.563713
H	1.683605	7.839408	2.587807
H	2.365162	6.527518	0.554630
H	2.056808	4.074352	0.500484
H	0.046368	0.805576	1.124068
H	6.393029	-5.089276	4.018377
H	6.611693	-4.205091	2.462299
H	6.399301	-3.286662	3.999377

Table S108. Cartesian geometry of 3f-TS2 (41.4 kcal/mol) in Figure S146 in Angstrom [Å].

Atomtype	X Coordinates	Y Coordinates	Z Coordinates
C	-0.421387	4.191888	-4.290720
C	-0.832941	3.677003	-3.034752
C	-1.201960	4.616811	-2.043926
C	-1.185266	5.999577	-2.290357
C	-0.775943	6.485477	-3.543699

C	-0.389996	5.574568	-4.543310
Si	-0.827973	1.776987	-2.707398
Ge	-1.266059	-0.023995	0.068699
C	-2.667825	-1.077940	-0.813127
C	-4.051987	-0.797089	-0.908074
C	-4.961899	-1.732110	-1.407737
C	-4.536398	-3.024431	-1.837923
C	-3.143710	-3.308999	-1.743187
C	-2.249092	-2.356945	-1.247111
N	-5.434309	-3.959362	-2.324978
C	-4.957825	-5.259012	-2.765356
Ni	0.964534	-0.172373	-0.067114
C	2.280240	-0.452202	-1.425377
N	3.342001	0.349088	-1.825695
C	3.942954	-0.128754	-2.994852
C	3.274688	-1.259922	-3.348226
N	2.280305	-1.457002	-2.385070
C	4.027133	1.293506	-0.969769
C	5.071110	0.779033	-0.153092
C	5.755383	1.678444	0.686916
C	5.442512	3.041877	0.681652
C	4.448938	3.532750	-0.175810
C	3.713140	2.676598	-1.021141
C	1.480374	-2.656073	-2.356166
C	1.808072	-3.671973	-1.410932
C	1.047344	-4.855735	-1.436972
C	0.044931	-5.053703	-2.399078
C	-0.217989	-4.065057	-3.352183
C	0.479390	-2.839432	-3.343412
C	5.550488	-0.668778	-0.262318
C	6.855146	-0.723534	-1.089873
C	2.642194	3.222970	-1.968900
C	2.229816	4.673441	-1.668291
C	3.023941	-3.536738	-0.490686
C	4.308035	-3.835239	-1.303962
C	0.172974	-1.783477	-4.402560
C	0.742345	-2.207768	-5.775146
P	1.201236	-0.612614	2.146902
C	0.299803	0.638561	3.221090
Si	-0.901145	1.918052	2.434668
C	-1.772824	2.638807	3.997763
C	-0.737101	3.170685	5.018237
C	2.758217	-0.842072	3.166924
C	3.203713	-2.100611	3.632704
C	4.412460	-2.221041	4.339837
C	5.203375	-1.089231	4.596079
C	4.782194	0.164862	4.120387
C	3.581327	0.285367	3.406574
C	0.299309	-2.197838	2.543852
C	-0.056431	-3.040455	1.473211
C	-0.697297	-4.268413	1.700220
C	-0.981449	-4.675036	3.013386
C	-0.633651	-3.841523	4.092964
C	-0.003302	-2.608626	3.860028
C	5.738305	-1.368172	1.092417
C	3.081446	3.141092	-3.450364
C	3.005476	-4.439012	0.755800
C	-1.332165	-1.473789	-4.521673
N	-2.079362	1.127429	1.344683
C	-3.491936	1.150842	1.590258

C	-4.088368	0.170344	2.442956
C	-5.475256	0.228807	2.685229
C	-6.283516	1.205422	2.092570
C	-5.701500	2.143215	1.230370
C	-4.318572	2.139821	0.968132
C	-3.297166	-0.990732	3.046489
C	-3.767437	-2.338928	2.455445
C	-3.755940	3.164606	-0.013347
C	-4.102215	4.619903	0.366472
C	-3.365251	-1.028452	4.587290
C	-4.227022	2.879627	-1.456373
C	0.129136	3.196676	1.429649
C	1.626556	3.162815	1.791397
C	-0.423186	4.635310	1.510504
C	-2.899631	3.673291	3.807519
C	-6.851392	-3.641276	-2.394352
H	0.038143	2.858327	0.375795
H	4.801363	0.367838	-3.444186
H	3.416358	-1.950495	-4.177642
H	2.605083	-2.998170	3.446150
H	0.672383	-0.847713	-4.099038
H	1.077607	1.220420	3.744443
H	-0.251705	0.097103	4.012592
H	-4.396393	-1.215697	4.942314
H	-3.025360	-0.077701	5.036370
H	-2.723153	-1.841621	4.974083
H	-0.291527	5.061606	2.522867
H	-1.496521	4.692128	1.264504
H	0.109512	5.298541	0.801833
H	-0.525770	-5.991678	-2.408339
H	-6.334239	2.898395	0.747100
H	-2.237034	-0.865738	2.769990
H	-2.239308	1.734225	4.439837
H	1.752153	2.572819	-1.843433
H	0.198155	-2.723788	0.455627
H	-7.362636	1.229843	2.293085
H	0.277357	-1.976819	4.712203
H	-5.933485	-0.521648	3.342106
H	-4.449982	0.161473	-0.570638
H	6.555958	1.305404	1.336119
H	3.294717	1.269623	3.020100
H	1.256023	-5.646723	-0.709419
H	-2.658845	3.050761	0.007727
H	-6.017323	-1.447976	-1.452005
H	3.052191	-2.488089	-0.139587
H	4.787790	-1.238322	-0.812045
H	-1.188588	-2.628819	-1.208818
H	-0.996406	-4.229845	-4.105433
H	5.986074	3.733422	1.338707
H	4.733975	-3.209316	4.694643
H	2.297604	3.583033	-4.090466
H	3.239325	2.106226	-3.789119
H	4.017898	3.711238	-3.604911
H	2.179443	3.969185	1.278716
H	2.091524	2.211184	1.474794
H	1.801034	3.296776	2.876899
H	4.237286	4.605600	-0.175244
H	-3.912173	1.881324	-1.799849
H	-3.798802	3.621069	-2.154606
H	-5.330443	2.936558	-1.523953

H	6.145099	-1.184630	5.151549
H	5.398677	1.056769	4.292873
H	-3.159911	-3.165069	2.862628
H	-3.672384	-2.348562	1.356859
H	-4.827278	-2.531865	2.709092
H	-0.858224	-4.152522	5.121500
H	-1.898324	-2.337295	-4.919070
H	-1.484438	-0.622939	-5.209581
H	-1.767628	-1.202544	-3.547163
H	-1.482804	-5.633555	3.199466
H	-0.966078	-4.900324	0.845124
H	6.489903	-0.863914	1.725081
H	6.086508	-2.405908	0.938532
H	4.793971	-1.402533	1.653091
H	3.074705	5.369768	-1.829753
H	1.866205	4.800378	-0.635822
H	1.421041	4.978583	-2.347550
H	2.071475	-4.336525	1.330020
H	3.845094	-4.160054	1.417160
H	3.134855	-5.505550	0.489748
H	1.829574	-2.400679	-5.733673
H	0.562674	-1.416174	-6.526628
H	0.252398	-3.134226	-6.131562
H	-3.366131	3.908155	4.785845
H	-3.696287	3.306068	3.141569
H	-2.515646	4.624304	3.397676
H	-5.185889	4.814697	0.258849
H	-3.574886	5.321655	-0.305865
H	-3.821793	4.854633	1.406547
H	-2.741777	-4.273796	-2.061290
H	-0.175265	4.034939	4.614837
H	-0.000213	2.406891	5.325067
H	-1.247651	3.519910	5.938258
H	6.731907	-0.247165	-2.078920
H	7.166500	-1.773386	-1.249246
H	7.673704	-0.198202	-0.562021
H	4.275472	-4.871593	-1.690465
H	5.202068	-3.736842	-0.663253
H	4.429323	-3.153522	-2.161271
H	-2.143316	1.268661	-3.257495
H	0.186200	1.274890	-3.718451
H	-0.117704	3.498611	-5.087586
H	-0.063903	5.943385	-5.525478
H	-0.750569	7.565578	-3.739359
H	-1.475828	6.698090	-1.494028
H	-1.485500	4.264697	-1.048638
H	0.055960	0.679361	-1.192514
H	-7.406929	-4.504853	-2.794537
H	-7.270856	-3.400827	-1.394772
H	-7.050275	-2.772564	-3.056868
H	-5.804766	-5.863298	-3.128572
H	-4.223009	-5.172141	-3.594179
H	-4.465539	-5.822328	-1.943833

Table S109. Cartesian geometry of 3g-TS2 (46.3 kcal/mol) in Figure S146 in Angstrom [Å].

Atomtype	X Coordinates	Y Coordinates	Z Coordinates
C	2.070639	5.491649	1.277427
C	2.376967	4.174755	0.848306
C	3.296148	4.047025	-0.220291
C	3.894949	5.165209	-0.823855

C	3.572113	6.459817	-0.382102
C	2.654034	6.618332	0.671337
Si	1.508747	2.642349	1.642034
Ge	1.267439	-0.575095	0.916714
N	1.781793	-1.112016	2.606830
C	0.722213	-1.530480	3.511803
Ni	-0.781933	0.032852	0.198392
C	-2.252556	1.143545	0.693858
N	-2.819229	2.240758	0.067867
C	-3.719596	2.907182	0.905357
C	-3.748597	2.220523	2.079762
N	-2.868142	1.145331	1.934869
C	-2.823574	2.472825	-1.359986
C	-3.878949	1.873345	-2.100023
C	-3.912548	2.092831	-3.490503
C	-2.961261	2.912206	-4.108154
C	-1.965770	3.537380	-3.344995
C	-1.864188	3.333909	-1.953019
C	-2.741590	0.115694	2.934589
C	-3.391971	-1.133028	2.710481
C	-3.287271	-2.107285	3.720215
C	-2.615975	-1.837328	4.923665
C	-2.037104	-0.582879	5.140277
C	-2.074753	0.418090	4.147358
C	-5.030895	1.131565	-1.418904
C	-6.272271	2.050889	-1.351475
C	-0.780861	4.026977	-1.120916
C	0.324386	4.682866	-1.965642
C	-4.265495	-1.360059	1.472657
C	-5.621954	-0.636193	1.660503
C	-1.448813	1.785716	4.413775
C	-2.308402	2.585792	5.418328
P	-0.930362	-1.716376	-1.262461
C	0.591307	-1.835262	-2.353498
Si	2.173672	-0.787201	-2.010071
C	3.446469	-1.653371	-3.172492
C	2.923541	-1.709343	-4.628990
C	-2.262630	-2.118041	-2.517184
C	-3.198848	-3.162899	-2.343376
C	-4.201279	-3.397531	-3.300432
C	-4.291606	-2.593333	-4.448039
C	-3.382290	-1.535271	-4.619580
C	-2.387419	-1.293377	-3.661383
C	-0.868510	-3.315687	-0.305669
C	-1.121244	-3.268871	1.078353
C	-1.157715	-4.443478	1.846436
C	-0.944663	-5.687463	1.232007
C	-0.682110	-5.747235	-0.149600
C	-0.639580	-4.569574	-0.912752
C	-5.390000	-0.210483	-2.074533
C	-1.368257	5.104639	-0.177705
C	-4.537240	-2.834552	1.126004
C	0.008426	1.695404	4.904438
N	2.693350	-0.897498	-0.300918
C	3.956669	-1.466711	0.071702
C	4.095315	-2.883668	0.201638
C	5.350619	-3.418425	0.553263
C	6.457941	-2.595531	0.789070
C	6.314709	-1.207194	0.677387
C	5.085847	-0.621865	0.318194

C	2.914761	-3.842690	0.049030
C	2.501537	-4.406565	1.425649
C	4.997610	0.899825	0.230945
C	6.028615	1.498553	-0.750672
C	3.182345	-4.991053	-0.945289
C	5.161027	1.564510	1.615071
C	3.098270	-1.134909	3.224266
C	1.790808	1.043617	-2.455067
C	0.544155	1.182612	-3.350083
C	2.994468	1.785444	-3.073494
C	4.909809	-1.169278	-3.163158
H	1.560356	1.527618	-1.481860
H	-4.264921	3.788998	0.573689
H	-4.312635	2.381439	2.996814
H	-3.147240	-3.805210	-1.458174
H	-1.426142	2.342071	3.461033
H	0.263138	-1.563656	-3.370897
H	0.895134	-2.896888	-2.415009
H	3.964272	-5.679685	-0.573594
H	3.511334	-4.613848	-1.930682
H	2.260161	-5.584210	-1.091236
H	3.222712	1.407628	-4.087980
H	3.910130	1.684538	-2.468406
H	2.781635	2.867924	-3.164249
H	-2.557099	-2.610227	5.700941
H	7.177602	-0.557949	0.872137
H	2.054052	-3.270735	-0.334118
H	3.447774	-2.690837	-2.779374
H	-0.318590	3.239474	-0.489615
H	-1.314704	-2.295587	1.541839
H	7.425966	-3.033548	1.065056
H	-0.455408	-4.633678	-1.992629
H	5.457522	-4.505591	0.658081
H	-4.707314	1.632817	-4.089095
H	-1.712585	-0.442599	-3.806241
H	-3.754145	-3.086964	3.575547
H	3.983856	1.137515	-0.135379
H	-3.734260	-0.910034	0.612549
H	-4.729149	0.908039	-0.385208
H	-1.528738	-0.376321	6.089178
H	-3.002173	3.080260	-5.192339
H	-4.913379	-4.218928	-3.145885
H	-0.544650	5.609673	0.356573
H	-2.043416	4.684110	0.582434
H	-1.921530	5.865535	-0.761570
H	0.409153	2.225007	-3.686893
H	-0.375542	0.907651	-2.802433
H	0.609070	0.560211	-4.263902
H	-1.246954	4.188542	-3.850060
H	4.397914	1.217410	2.328784
H	5.055443	2.660831	1.525990
H	6.159476	1.344962	2.039543
H	-5.071851	-2.781145	-5.196572
H	-3.453858	-0.881794	-5.498669
H	1.645142	-5.093961	1.319983
H	2.204866	-3.588569	2.101603
H	3.339172	-4.960336	1.890726
H	-0.508681	-6.716653	-0.634339
H	0.083029	1.188293	5.884866
H	0.429800	2.710218	5.013690

H	0.640608	1.157322	4.180870
H	-0.972427	-6.609925	1.825991
H	-1.362753	-4.376394	2.921897
H	-5.703428	-0.093122	-3.126938
H	-6.232230	-0.681063	-1.534620
H	-4.537664	-0.903415	-2.051232
H	-0.079206	5.517171	-2.570644
H	0.815515	3.967387	-2.644107
H	1.098013	5.100611	-1.305402
H	-3.610076	-3.423973	1.053318
H	-5.053084	-2.888276	0.150618
H	-5.196205	-3.314251	1.874591
H	-3.353290	2.694352	5.074608
H	-1.887281	3.598089	5.564119
H	-2.332707	2.080760	6.402952
H	5.528921	-1.828498	-3.804876
H	5.349384	-1.183936	-2.153684
H	5.003013	-0.146032	-3.568139
H	7.060113	1.369978	-0.372141
H	5.855276	2.584162	-0.868155
H	5.977785	1.026674	-1.745592
H	2.831595	-0.697021	-5.066932
H	1.939582	-2.203379	-4.718968
H	3.631188	-2.273580	-5.269043
H	-6.042718	3.014070	-0.862089
H	-7.084759	1.560024	-0.782778
H	-6.648603	2.271175	-2.368487
H	-6.156414	-1.058534	2.532440
H	-6.258130	-0.775190	0.768367
H	-5.502199	0.446754	1.824250
H	2.367582	2.306731	2.850668
H	0.303054	3.296182	2.302881
H	1.358894	5.639134	2.101687
H	2.392493	7.625631	1.023870
H	4.027441	7.338793	-0.857238
H	4.601660	5.024095	-1.653113
H	3.529470	3.052470	-0.610262
H	0.100620	1.099944	0.933279
H	3.302393	-2.133031	3.670217
H	3.894242	-0.936661	2.495614
H	3.172416	-0.380240	4.038925
H	0.751632	-2.628866	3.698171
H	0.802385	-1.025751	4.495336
H	-0.270072	-1.282312	3.102478

Table S110. Cartesian geometry of 3a-TS3 (74.4 kcal/mol) in Figure S146 in Angstrom [Å].

Atomtype	X Coordinates	Y Coordinates	Z Coordinates
C	2.061812	-4.223615	-1.986133
C	1.486408	-3.734914	-0.792187
C	1.668376	-4.503803	0.380834
C	2.315981	-5.747583	0.347917
C	2.827231	-6.246773	-0.861083
C	2.712425	-5.468109	-2.022976
P	0.395563	-2.188516	-0.705991
C	-1.140362	-3.074792	-0.000291
Si	-2.329425	-2.206067	1.225822
C	-1.239820	-1.873468	2.778619
C	-0.809717	-3.197776	3.448967
Ni	0.671753	0.026576	0.115286
C	2.478517	0.790161	0.111913

N	3.663877	0.094788	0.405438
C	4.770025	0.942386	0.480626
C	4.329229	2.195908	0.205303
N	2.954533	2.110292	-0.021821
C	3.973193	-1.319224	0.228764
C	4.106281	-2.196240	1.337916
C	4.648712	-3.474689	1.103036
C	5.058412	-3.882317	-0.169425
C	4.921720	-3.005073	-1.245469
C	4.402742	-1.708611	-1.069667
C	2.294471	3.305794	-0.522661
C	2.012677	3.392568	-1.915098
C	1.491259	4.601900	-2.413907
C	1.249986	5.694970	-1.574525
C	1.589016	5.611761	-0.221490
C	2.143964	4.438475	0.328462
C	3.733690	-1.845711	2.775063
C	2.234949	-1.540197	2.912632
C	4.427900	-0.753289	-2.260640
C	5.882887	-0.480436	-2.701510
C	2.405234	2.287481	-2.893547
C	1.522505	2.204045	-4.151091
C	2.635710	4.484989	1.775014
C	3.933871	5.321329	1.877537
Ge	-1.581223	0.461728	-0.058639
C	-2.324695	1.830168	-1.320142
C	-1.383329	2.692770	-1.918777
C	-1.782982	3.717953	-2.783945
C	-3.155036	3.899621	-3.079331
C	-4.110551	3.047282	-2.474438
C	-3.695906	2.034113	-1.601226
C	-3.567172	4.941064	-3.978382
N	-3.894796	5.792386	-4.714392
Si	-0.457828	2.068218	1.845245
C	0.631926	1.754877	3.409821
C	-0.022950	1.902527	4.658325
C	0.692379	1.937081	5.865940
C	2.095253	1.869880	5.849665
C	2.762392	1.716408	4.623163
C	2.034674	1.616986	3.426046
N	-2.991190	-0.741765	0.460871
C	-4.411829	-0.524264	0.362467
C	-5.093552	0.278428	1.342031
C	-6.497148	0.379055	1.322196
C	-7.262790	-0.282903	0.359521
C	-6.601416	-1.003682	-0.637482
C	-5.193440	-1.122308	-0.689548
C	-4.352588	1.113813	2.382349
C	-4.422375	2.612312	2.013635
C	-4.685739	-1.859643	-1.948041
C	-3.440738	-1.277651	-2.623913
C	-0.098088	-2.062432	-2.507014
C	-0.562729	-3.178110	-3.239583
C	-0.945422	-3.046616	-4.581933
C	-0.899370	-1.787597	-5.206289
C	-0.461007	-0.668333	-4.482390
C	-0.050135	-0.807357	-3.145434
C	4.662663	-0.777523	3.385742
C	3.568837	-1.253896	-3.436184
C	3.879877	2.497887	-3.311042

C	1.597778	5.040444	2.773450
C	-3.786391	-3.402568	1.620720
C	-4.525543	-3.059928	2.930881
C	-3.400217	-4.896121	1.571754
C	-4.841771	0.895807	3.828226
C	-4.567003	-3.391832	-1.789184
C	-1.785325	-0.917642	3.850680
H	-1.931750	1.649536	1.111484
H	5.768398	0.559704	0.686945
H	4.862002	3.140872	0.126258
H	-0.794104	-3.998102	0.493595
H	-1.726816	-3.418765	-0.868895
H	-4.511606	-3.222687	0.807837
H	-0.340673	-1.391803	2.338232
H	-1.521656	2.798575	2.660330
H	0.050617	3.097908	0.908277
H	-1.113757	2.021265	4.688335
H	0.154202	2.044106	6.817102
H	2.665638	1.934108	6.785617
H	3.856822	1.670736	4.600193
H	2.569225	1.442609	2.489293
H	1.281421	-4.139985	1.339331
H	2.422283	-6.326310	1.274755
H	3.322176	-7.225827	-0.893517
H	3.128078	-5.829709	-2.972913
H	1.977464	-3.647673	-2.911819
H	0.297514	0.063006	-2.573680
H	-0.446092	0.317979	-4.958010
H	-1.216882	-1.677704	-6.251156
H	-1.295272	-3.926125	-5.137996
H	-0.614493	-4.163517	-2.760972
H	-1.045068	-0.783196	4.662834
H	-1.995096	0.084455	3.446854
H	-2.719921	-1.290903	4.304107
H	-0.046449	-3.010977	4.229130
H	-1.667259	-3.686684	3.947247
H	-0.383954	-3.931095	2.740726
H	-7.194145	-1.483508	-1.428758
H	-8.358016	-0.212154	0.365173
H	-6.997678	0.994384	2.080065
H	-5.505458	-1.716685	-2.680026
H	-3.299020	0.809300	2.348877
H	-4.176823	1.428278	4.534231
H	-5.864986	1.287863	3.979829
H	-4.844059	-0.172450	4.104187
H	-3.846050	3.217257	2.737302
H	-4.006347	2.798463	1.008286
H	-5.470253	2.968263	2.019808
H	-4.428605	-3.860609	-2.782268
H	-3.703845	-3.679455	-1.169596
H	-5.474869	-3.822229	-1.328091
H	-3.254752	-1.795590	-3.580300
H	-3.556894	-0.203915	-2.837216
H	-2.544657	-1.405923	-2.005478
H	-4.289514	-5.532118	1.757199
H	-2.984714	-5.191189	0.590489
H	-2.648992	-5.156549	2.340659
H	-5.421148	-3.703150	3.047771
H	-3.892031	-3.220771	3.822755
H	-4.871837	-2.011841	2.937914

H	-4.458183	1.400754	-1.140156
H	-5.176737	3.188644	-2.688064
H	-1.040018	4.382696	-3.234524
H	-0.318332	2.566514	-1.691952
H	1.283635	4.694234	-3.484697
H	0.822957	6.619726	-1.982626
H	1.440676	6.482868	0.426731
H	5.239947	-3.322117	-2.245260
H	5.466099	-4.887791	-0.322490
H	4.745336	-4.167226	1.948607
H	2.316389	1.321190	-2.366624
H	2.873766	3.458450	2.086085
H	1.946255	4.862891	3.806905
H	1.456655	6.130251	2.647786
H	0.614743	4.556078	2.664217
H	4.318513	5.303129	2.914695
H	4.732282	4.950279	1.210715
H	3.739845	6.375592	1.604122
H	4.192091	1.725859	-4.035907
H	3.996398	3.487232	-3.791912
H	4.563738	2.453325	-2.446534
H	1.768252	1.284985	-4.710710
H	0.449771	2.178162	-3.898983
H	1.694456	3.057507	-4.832946
H	4.000042	0.204997	-1.938454
H	3.918318	-2.765850	3.363883
H	3.599796	-0.530445	-4.271300
H	3.930158	-2.224694	-3.822671
H	2.516944	-1.372966	-3.132430
H	5.909457	0.257354	-3.523676
H	6.483763	-0.081956	-1.863728
H	6.372995	-1.404348	-3.060718
H	4.456203	-0.669789	4.465571
H	5.722006	-1.071032	3.261866
H	4.524392	0.208718	2.918128
H	1.653223	-2.438433	2.657629
H	1.972023	-1.235056	3.940431
H	1.906046	-0.740217	2.229921

Table S111. Cartesian geometry of 3b-TS3 (78.1 kcal/mol) in Figure S146 in Angstrom [Å].

Atomtype	X Coordinates	Y Coordinates	Z Coordinates
C	0.030263	0.346991	-3.341149
C	0.460587	-0.984653	-3.214822
C	0.224792	-1.881033	-4.282370
C	-0.419014	-1.447565	-5.449891
C	-0.853624	-0.114311	-5.559871
C	-0.631605	0.781171	-4.502813
P	1.190096	-1.534130	-1.587615
Ni	1.083421	0.043764	0.046957
Ge	-1.176361	-0.173693	0.416789
N	-2.036199	-1.919938	0.418738
C	-3.452528	-2.157207	0.531025
C	-4.075494	-2.206704	1.831290
C	-5.438477	-2.536662	1.947910
C	-6.225984	-2.801629	0.826435
C	-5.642640	-2.697678	-0.436632
C	-4.281220	-2.367028	-0.629767
C	-3.348737	-1.851760	3.132454

C	-3.351652	-2.989938	4.175835
C	-3.890535	-2.294100	-2.126181
C	-3.438099	-3.648448	-2.719538
C	2.719656	-2.463251	-2.169727
C	3.226013	-2.431161	-3.486762
C	4.231514	-3.324988	-3.893126
C	4.756264	-4.263434	-2.990919
C	4.300264	-4.266157	-1.662177
C	3.308892	-3.363235	-1.254077
C	0.119494	-3.088758	-1.313192
Si	-0.903562	-3.284004	0.288059
C	-1.903391	-4.928648	0.144900
C	-1.179331	-6.022815	-0.669681
C	0.446067	-3.357983	1.655649
C	0.034752	-3.117779	3.113738
C	1.263935	-4.666025	1.560963
C	2.565095	1.287450	0.400692
N	3.931915	0.997773	0.493424
C	4.679579	2.108935	0.891334
C	3.809374	3.140907	1.045120
N	2.536968	2.651899	0.743928
C	4.664620	-0.084980	-0.146940
C	5.218077	-1.157441	0.604612
C	6.094630	-2.036685	-0.059532
C	6.409475	-1.884859	-1.414041
C	5.852692	-0.827817	-2.134243
C	5.002233	0.107207	-1.513733
C	1.400128	3.546834	0.670411
C	0.868256	3.850321	-0.616479
C	-0.285298	4.651582	-0.681770
C	-0.869300	5.181781	0.475931
C	-0.240696	4.999861	1.709691
C	0.920933	4.208299	1.836392
C	4.962742	-1.405752	2.089236
C	5.609261	-0.330325	2.984872
C	4.585604	1.346585	-2.302029
C	3.750389	1.012284	-3.549932
C	1.624306	3.480935	-1.895286
C	2.892677	4.362172	-2.015076
C	1.678679	4.248294	3.167033
C	0.812852	4.031016	4.423660
C	3.475721	-1.664592	2.385946
C	5.825958	2.193902	-2.661646
C	0.809862	3.646563	-3.185753
C	2.417101	5.605818	3.279156
C	-2.636143	1.099560	-0.219146
C	-2.456224	2.079210	-1.215432
C	-3.552961	2.834145	-1.687958
C	-4.841815	2.627287	-1.171987
C	-5.025230	1.669632	-0.166523
C	-3.933856	0.926354	0.307539
C	-3.381011	3.913310	-2.735232
F	-3.057474	5.117500	-2.179273
C	-6.417679	1.383333	0.360746
F	-6.395518	0.965765	1.648813

Si	-0.386196	0.705818	2.800620
C	1.121748	0.348092	3.948808
C	0.856924	-0.402373	5.120519
C	1.797726	-0.495483	6.159535
C	3.009345	0.210158	6.071795
C	3.288504	0.958215	4.915923
C	2.376202	0.982058	3.850273
F	-2.391494	3.622806	-3.623379
F	-4.509696	4.114071	-3.453430
F	-7.213684	2.482038	0.301069
F	-7.031508	0.413170	-0.364311
C	-3.943391	-0.579912	3.781052
C	-2.957938	-1.161553	-2.566732
C	-2.405386	-5.481318	1.493010
H	-1.814192	0.250541	1.935080
H	5.763223	2.063003	0.988154
H	3.976623	4.180181	1.319001
H	0.772063	-3.971570	-1.420178
H	-0.585101	-3.135047	-2.158804
H	-2.808491	-4.645613	-0.420023
H	1.098195	-2.508204	1.356221
H	-1.390348	0.423671	3.893635
H	-0.455761	2.136841	2.417652
H	-0.113443	-0.901982	5.241215
H	1.570194	-1.094632	7.051244
H	3.732186	0.176597	6.897498
H	4.229682	1.515290	4.838948
H	2.641781	1.508860	2.932455
H	2.971513	-3.372798	-0.212448
H	4.726848	-4.966348	-0.932437
H	5.527404	-4.974277	-3.314354
H	4.596121	-3.291529	-4.928475
H	2.817219	-1.726713	-4.217065
H	0.203932	1.021729	-2.494317
H	-0.994387	1.810482	-4.578199
H	-1.374849	0.222909	-6.465017
H	-0.593504	-2.154569	-6.271453
H	0.544740	-2.926795	-4.200413
H	0.925276	-3.078584	3.770636
H	-0.495925	-2.161633	3.241910
H	-0.628777	-3.915942	3.491021
H	2.175964	-4.607373	2.188584
H	0.675624	-5.524655	1.934442
H	1.585693	-4.913690	0.534246
H	-6.269081	-2.865485	-1.323222
H	-7.288308	-3.055065	0.932009
H	-5.894177	-2.575009	2.945177
H	-4.852088	-2.078017	-2.632119
H	-2.301265	-1.647303	2.874984
H	-2.743396	-2.697417	5.052636
H	-4.375008	-3.204119	4.537325
H	-2.932934	-3.925334	3.773691
H	-3.427623	-0.358498	4.733034
H	-3.837545	0.307722	3.135344
H	-5.019346	-0.710315	3.998715

H	-3.430114	-3.586216	-3.824600
H	-2.420815	-3.924893	-2.402662
H	-4.120341	-4.466801	-2.424979
H	-2.796250	-1.206674	-3.658638
H	-3.389300	-0.177890	-2.329914
H	-1.971107	-1.224695	-2.091823
H	-1.830776	-6.913432	-0.779536
H	-0.912359	-5.685572	-1.688073
H	-0.248555	-6.361090	-0.178047
H	-2.967104	-6.425126	1.339404
H	-1.581381	-5.703394	2.196500
H	-3.092834	-4.769760	1.978551
H	-4.122603	0.187823	1.085774
H	-5.690423	3.206497	-1.547886
H	-1.466625	2.248945	-1.646058
H	-0.733181	4.886458	-1.648774
H	-1.788754	5.774721	0.399006
H	-0.648869	5.490666	2.600487
H	6.100737	-0.703895	-3.194548
H	7.077927	-2.598944	-1.908636
H	6.528121	-2.871830	0.505523
H	1.927650	2.421144	-1.815244
H	2.441805	3.457259	3.161301
H	1.461018	4.005608	5.318666
H	0.083137	4.849417	4.565858
H	0.261784	3.078877	4.385611
H	3.054951	5.627018	4.183102
H	3.053447	5.810792	2.398538
H	1.690094	6.436170	3.352957
H	3.424307	4.136943	-2.956621
H	2.604518	5.430041	-2.032777
H	3.600166	4.210558	-1.185134
H	1.347582	3.184413	-4.031358
H	-0.178116	3.176383	-3.118817
H	0.650284	4.713256	-3.429464
H	3.951411	1.964329	-1.654209
H	5.496220	-2.346518	2.329933
H	3.476096	1.939786	-4.084743
H	4.305909	0.367984	-4.255466
H	2.819082	0.494455	-3.271447
H	5.523535	3.128737	-3.168168
H	6.398088	2.464205	-1.755056
H	6.505273	1.645594	-3.340585
H	5.532259	-0.624143	4.046971
H	6.679155	-0.207362	2.731108
H	5.113180	0.644585	2.877032
H	3.172452	-2.619881	1.930068
H	3.285645	-1.725605	3.471602
H	2.819921	-0.880520	1.973673

Table S112. Cartesian geometry of 3c-TS3 (74 kcal/mol) in Figure S146 in Angstrom [Å].

Atomtype	X Coordinates	Y Coordinates	Z Coordinates
C	-5.125465	-1.316799	-0.656358
C	-4.505885	-0.211908	0.025630
C	-5.303794	0.544545	0.956444

C	-6.683656	0.253340	1.058272
C	-7.294596	-0.775156	0.337363
C	-6.499287	-1.571945	-0.489632
N	-3.136463	0.137331	-0.250161
Si	-2.727555	1.415797	-1.415246
C	-1.694198	0.823275	-2.928747
C	-1.492738	1.967802	-3.947344
C	-4.834697	1.640589	1.938074
C	-3.485178	1.411417	2.624638
C	-4.336111	-2.302792	-1.513293
C	-4.179293	-3.650033	-0.773874
C	-1.604692	2.713574	-0.562926
P	0.074901	2.231291	0.203626
C	0.942883	3.866553	-0.202741
C	0.951086	4.314884	-1.544154
C	1.418474	5.592183	-1.886888
C	1.920101	6.451297	-0.895604
C	1.979831	5.998806	0.431546
C	1.509242	4.719369	0.770733
Ni	0.608755	-0.063436	-0.056204
C	2.502186	-0.573079	-0.025973
N	3.560994	0.153524	-0.598828
C	4.767282	-0.547429	-0.558606
C	4.521693	-1.724812	0.069769
N	3.164946	-1.743638	0.397819
C	3.681205	1.589554	-0.819267
C	3.626576	2.149993	-2.123238
C	3.995872	3.500699	-2.274009
C	4.413949	4.279023	-1.191094
C	4.463606	3.709401	0.081297
C	4.124870	2.359352	0.290869
C	2.705319	-2.830278	1.247952
C	2.519699	-2.574912	2.635878
C	2.188315	-3.657990	3.472152
C	2.046089	-4.954579	2.966528
C	2.297303	-5.197732	1.613790
C	2.659416	-4.158076	0.733603
C	3.223692	1.388127	-3.381958
C	1.776758	0.880872	-3.295525
C	4.355102	1.764304	1.678143
C	5.857127	1.801811	2.036623
C	2.826891	-1.213975	3.258585
C	2.041098	-0.903820	4.544834
C	3.073697	-4.532196	-0.689450
C	4.465015	-5.210052	-0.685448
Ge	-1.547265	-0.717599	0.435329
C	-2.013834	-1.746837	2.085554
C	-0.931889	-2.237413	2.842222
C	-1.148604	-3.004930	3.999966
C	-2.456924	-3.291204	4.422109
C	-3.544520	-2.819602	3.667195
C	-3.326440	-2.061611	2.504130
Si	-0.330247	-2.601922	-1.097331
C	0.624065	-2.589042	-2.782043
C	-0.070775	-3.136564	-3.889633
C	0.576072	-3.406909	-5.106199
C	1.956247	-3.177530	-5.230286
C	2.661749	-2.628972	-4.146678
C	1.992537	-2.299664	-2.956480
C	4.254991	0.314462	-3.782365

C	3.500659	2.445099	2.762888
C	4.343599	-1.137446	3.554474
C	2.067934	-5.449539	-1.417004
C	-0.293710	2.524968	2.015386
C	-0.866089	3.729782	2.482536
C	-1.149317	3.909567	3.843793
C	-0.892895	2.872741	4.758372
C	-0.346236	1.663456	4.302825
C	-0.036412	1.495587	2.942298
C	-4.356101	2.286877	-1.966327
C	-5.113723	1.532512	-3.078686
C	-4.179585	3.777570	-2.325119
C	-4.940403	3.080694	1.388246
C	-4.929927	-2.530299	-2.917936
C	-2.163028	-0.437830	-3.670251
H	-1.807575	-2.197978	-0.360484
H	5.688765	-0.119259	-0.950147
H	5.184016	-2.545229	0.336765
H	-1.417506	3.508283	-1.304341
H	-2.183893	3.202099	0.238772
H	-5.003157	2.236951	-1.073132
H	-0.712810	0.584438	-2.466274
H	-1.321178	-3.643820	-1.612784
H	0.367487	-3.284751	0.018214
H	-1.135682	-3.384639	-3.792530
H	0.006097	-3.822761	-5.947752
H	2.478779	-3.421543	-6.164654
H	3.740090	-2.453934	-4.231130
H	2.548951	-1.822634	-2.146411
H	0.567232	3.666480	-2.339783
H	1.391800	5.913831	-2.936261
H	2.273772	7.456692	-1.157837
H	2.392145	6.645920	1.217227
H	1.557683	4.402588	1.816171
H	0.391668	0.552222	2.579461
H	-0.169046	0.843523	5.006742
H	-1.132049	3.003233	5.821682
H	-1.586235	4.855475	4.189680
H	-1.082637	4.541740	1.777859
H	-1.461261	-0.690120	-4.488588
H	-2.205152	-1.317047	-3.009622
H	-3.166312	-0.311577	-4.112682
H	-0.759327	1.675536	-4.723900
H	-2.438013	2.205163	-4.469746
H	-1.130470	2.905524	-3.488890
H	-7.292180	0.851544	1.750918
H	-8.369109	-0.971882	0.444757
H	-6.951974	-2.418008	-1.021560
H	-5.584899	1.595894	2.752756
H	-3.333781	-1.878810	-1.651471
H	-4.237587	-3.145266	-3.523497
H	-5.895877	-3.067836	-2.874236
H	-5.095546	-1.579570	-3.452809
H	-3.568522	-4.351042	-1.371797
H	-3.683956	-3.516688	0.203484
H	-5.167742	-4.114976	-0.595096
H	-4.816697	3.806133	2.215171
H	-4.159050	3.298705	0.644127
H	-5.922228	3.262437	0.913629
H	-3.323303	2.179443	3.400474

H	-3.435842	0.423177	3.106717
H	-2.651548	1.484354	1.916257
H	-5.157931	4.232951	-2.581077
H	-3.753317	4.363711	-1.490089
H	-3.517248	3.919051	-3.199661
H	-6.095151	2.012045	-3.270201
H	-4.560661	1.532655	-4.036252
H	-5.311425	0.484379	-2.794565
H	-4.191094	-1.714763	1.932062
H	-4.572896	-3.045970	3.979133
H	-2.628361	-3.884840	5.329805
H	-0.292104	-3.380884	4.570963
H	0.091772	-2.029582	2.509604
H	2.052449	-3.484369	4.544093
H	1.765956	-5.779096	3.634288
H	2.230112	-6.220979	1.227018
H	4.788704	4.317199	0.933518
H	4.681856	5.331311	-1.338012
H	3.945545	3.949890	-3.273877
H	2.565594	-0.436599	2.519080
H	3.154861	-3.605664	-1.274269
H	2.334301	-5.515429	-2.487423
H	2.080656	-6.475048	-1.002500
H	1.036507	-5.069304	-1.352099
H	4.788425	-5.424174	-1.721636
H	5.240632	-4.585494	-0.207639
H	4.428838	-6.168712	-0.134342
H	4.598255	-0.173315	4.028403
H	4.630901	-1.948181	4.250177
H	4.949543	-1.239825	2.638519
H	2.193535	0.153945	4.821348
H	0.960076	-1.074504	4.418361
H	2.388781	-1.517930	5.396579
H	4.053252	0.709356	1.650856
H	3.243554	2.133682	-4.201334
H	3.686912	1.979234	3.747845
H	3.735504	3.521724	2.853188
H	2.426927	2.343099	2.539842
H	6.037489	1.317173	3.013173
H	6.460299	1.273968	1.275421
H	6.228256	2.841496	2.101972
H	4.000958	-0.102853	-4.773130
H	5.268209	0.754917	-3.839537
H	4.284639	-0.519657	-3.065640
H	1.091617	1.738925	-3.228247
H	1.499174	0.281981	-4.180328
H	1.603077	0.260918	-2.401238

Table S113. Cartesian geometry of 3d-TS3 (74.3 kcal/mol) in Figure S146 in Angstrom [Å].

Atomtype	X Coordinates	Y Coordinates	Z Coordinates
C	-1.816010	4.443386	-1.554200
C	-1.246113	3.799513	-0.433438
C	-1.356240	4.450231	0.817310
C	-1.926051	5.726757	0.930811
C	-2.431774	6.379131	-0.205473
C	-2.390602	5.720662	-1.444180
P	-0.239837	2.197469	-0.534676
C	1.362344	2.935950	0.194313
Si	2.513651	1.896150	1.319060
C	1.434993	1.518631	2.868299

C	1.112329	2.809543	3.653540
Ni	-0.650138	-0.017320	0.097176
C	-2.497162	-0.681602	0.079448
N	-3.631995	0.035601	0.496569
C	-4.778270	-0.760117	0.531727
C	-4.416029	-1.997210	0.107057
N	-3.049016	-1.954940	-0.173083
C	-3.865623	1.474092	0.476666
C	-3.913447	2.238280	1.673173
C	-4.379410	3.564505	1.587435
C	-4.792680	4.127098	0.376537
C	-4.741560	3.359112	-0.786807
C	-4.305171	2.021155	-0.759794
C	-2.461765	-3.118240	-0.818288
C	-2.228027	-3.070613	-2.222187
C	-1.750330	-4.233182	-2.855891
C	-1.513992	-5.411195	-2.139261
C	-1.821516	-5.462047	-0.777503
C	-2.329426	-4.338325	-0.094359
C	-3.523485	1.719717	3.053829
C	-2.043395	1.313565	3.107090
C	-4.430977	1.194287	-2.037159
C	-5.914507	1.063150	-2.447061
C	-2.631263	-1.864196	-3.069748
C	-1.841464	-1.704459	-4.380876
C	-2.810709	-4.529898	1.344342
C	-4.141568	-5.320604	1.360412
Ge	1.561208	-0.621817	-0.149454
C	2.171962	-1.837313	-1.612741
C	1.177496	-2.434202	-2.406772
C	1.512687	-3.322287	-3.444047
C	2.854579	-3.642212	-3.722379
C	3.851100	-3.056293	-2.911752
C	3.520109	-2.177164	-1.870817
C	3.231968	-4.582427	-4.846967
Si	0.438667	-2.297501	1.635837
C	-0.631671	-2.071873	3.233290
C	0.024249	-2.345125	4.459701
C	-0.685272	-2.459703	5.666022
C	-2.085605	-2.350024	5.666409
C	-2.753545	-2.071749	4.462488
C	-2.028790	-1.890318	3.273230
N	3.056228	0.456079	0.431177
C	4.456992	0.164095	0.269804
C	5.115160	-0.748396	1.167346
C	6.509372	-0.927112	1.100423
C	7.289451	-0.240074	0.167350
C	6.647290	0.590121	-0.753864
C	5.247936	0.793611	-0.756817
C	4.353626	-1.617628	2.164915
C	4.341515	-3.088941	1.693962
C	4.760584	1.661634	-1.937959
C	3.461406	1.224772	-2.620453
C	0.188884	2.220119	-2.356228
C	0.704559	3.369641	-2.996548
C	1.042142	3.342142	-4.356965
C	0.896019	2.153367	-5.093970
C	0.404584	1.000310	-4.463467
C	0.041480	1.037248	-3.106316
C	-4.499743	0.650913	3.583624

C	-3.575287	1.749850	-3.190061
C	-4.139828	-1.969922	-3.397873
C	-1.795055	-5.238416	2.265338
C	4.054720	2.962933	1.767443
C	4.796299	2.473778	3.028529
C	3.767126	4.477485	1.842668
C	4.881938	-1.526047	3.610641
C	4.757111	3.180544	-1.654033
C	1.928964	0.441211	3.845673
H	1.916303	-1.939550	0.862370
H	-5.746195	-0.353756	0.821192
H	-5.001651	-2.901130	-0.044130
H	1.087384	3.841378	0.761054
H	1.953083	3.302493	-0.661880
H	4.750069	2.800384	0.925352
H	0.496700	1.138938	2.410857
H	1.473609	-3.129761	2.384970
H	-0.146949	-3.212651	0.626874
H	1.110997	-2.500414	4.470550
H	-0.145597	-2.662703	6.600682
H	-2.653068	-2.477340	6.597757
H	-3.846326	-1.991600	4.451721
H	-2.561414	-1.615744	2.359820
H	-0.971848	3.964377	1.721003
H	-1.977393	6.210172	1.915202
H	-2.866591	7.383505	-0.122740
H	-2.803938	6.203461	-2.339801
H	-1.785518	3.964232	-2.536663
H	-0.342359	0.140148	-2.604253
H	0.313456	0.065520	-5.026451
H	1.177768	2.123717	-6.154459
H	1.434594	4.246513	-4.840094
H	0.833825	4.299546	-2.429657
H	1.189227	0.281830	4.653909
H	2.073425	-0.533210	3.354115
H	2.888841	0.716317	4.316436
H	0.353168	2.612608	4.435596
H	2.011418	3.196538	4.168050
H	0.726084	3.625682	3.017017
H	7.248018	1.094646	-1.523595
H	8.378575	-0.373121	0.136582
H	6.990872	-1.624567	1.797211
H	5.549666	1.521879	-2.703675
H	3.316217	-1.260743	2.176829
H	4.207444	-2.077461	4.292750
H	5.888947	-1.973342	3.709541
H	4.939451	-0.481918	3.962013
H	3.755736	-3.714355	2.392212
H	3.890430	-3.186574	0.691163
H	5.370851	-3.492976	1.646238
H	4.627830	3.737461	-2.601928
H	3.932574	3.476224	-0.987409
H	5.704686	3.507700	-1.187786
H	3.287453	1.834747	-3.523820
H	3.497258	0.167107	-2.922440
H	2.592718	1.359341	-1.965613
H	4.700565	5.039296	2.050311
H	3.348703	4.873467	0.898929
H	3.052875	4.725386	2.650135
H	5.732866	3.049732	3.173395

H	4.191846	2.601655	3.945609
H	5.076062	1.409403	2.945501
H	4.326946	-1.749787	-1.269281
H	4.907853	-3.296630	-3.095755
H	0.716473	-3.776568	-4.046135
H	0.124170	-2.217174	-2.197341
H	-1.571822	-4.220477	-3.935315
H	-1.118245	-6.297651	-2.650896
H	-1.685042	-6.400032	-0.227369
H	-5.063828	3.796968	-1.738549
H	-5.136216	5.166972	0.339347
H	-4.409341	4.170193	2.501971
H	-2.457446	-0.955032	-2.467461
H	-3.005926	-3.537588	1.773555
H	-2.138858	-5.174016	3.313566
H	-1.693506	-6.309878	2.009923
H	-0.795423	-4.779924	2.212957
H	-4.526573	-5.400464	2.394611
H	-4.923893	-4.849216	0.739296
H	-3.988620	-6.345534	0.972909
H	-4.457695	-1.125073	-4.033701
H	-4.337898	-2.907958	-3.949873
H	-4.764070	-1.967556	-2.488879
H	-2.070301	-0.722516	-4.830404
H	-0.753924	-1.760727	-4.218260
H	-2.118193	-2.477236	-5.122376
H	-4.061573	0.182679	-1.825495
H	-3.633704	2.583266	3.739247
H	-3.687263	1.118171	-4.090237
H	-3.875590	2.778005	-3.464145
H	-2.508029	1.762725	-2.917878
H	-6.018126	0.414675	-3.335809
H	-6.514482	0.622862	-1.629658
H	-6.351014	2.048481	-2.695146
H	-4.265890	0.413209	4.636760
H	-5.541281	1.020433	3.532494
H	-4.439791	-0.285026	3.008770
H	-1.415699	2.198976	2.927435
H	-1.771049	0.886180	4.087836
H	-1.783585	0.573327	2.333261
H	2.340750	-5.007869	-5.342794
H	3.832738	-4.061754	-5.618835
H	3.850350	-5.422879	-4.475892

Table S114. Cartesian geometry of 3e-TS3 (74.4 kcal/mol) in Figure S146 in Angstrom [Å].

Atomtype	X Coordinates	Y Coordinates	Z Coordinates
C	2.264106	-3.959617	-2.281158
C	1.704927	-3.575738	-1.041990
C	1.977942	-4.396140	0.077285
C	2.697898	-5.593119	-0.050172
C	3.192838	-5.990830	-1.302900
C	2.987725	-5.156520	-2.412643
P	0.520784	-2.111645	-0.828236
C	-0.928681	-3.141092	-0.134270
Si	-2.110718	-2.428132	1.194416
C	-0.977677	-2.146342	2.726763
C	-0.451481	-3.486913	3.286709
Ni	0.710048	0.031961	0.118044
C	2.464180	0.910181	0.095666
N	3.701372	0.283383	0.327018

C	4.749598	1.200169	0.422765
C	4.218573	2.432957	0.221923
N	2.847923	2.265209	0.017523
C	4.095609	-1.094764	0.062249
C	4.318722	-2.022467	1.114588
C	4.926662	-3.250765	0.790027
C	5.312871	-3.563225	-0.516269
C	5.088428	-2.637444	-1.535292
C	4.503312	-1.385349	-1.268675
C	2.086866	3.431519	-0.401537
C	1.754462	3.567230	-1.779496
C	1.101996	4.744148	-2.192797
C	0.790255	5.763244	-1.286582
C	1.192873	5.646348	0.046041
C	1.872682	4.503006	0.512853
C	3.977046	-1.775680	2.580715
C	2.469078	-1.567014	2.783544
C	4.436799	-0.363910	-2.401663
C	5.860015	0.011772	-2.869995
C	2.225849	2.561559	-2.829369
C	1.359578	2.502283	-4.100055
C	2.431667	4.530622	1.935668
C	3.662255	5.466988	2.007912
Ge	-1.568434	0.373909	0.075944
C	-2.420618	1.711155	-1.131215
C	-1.559550	2.556120	-1.861527
C	-2.058292	3.542708	-2.719027
C	-3.450843	3.713973	-2.871774
C	-4.329244	2.890348	-2.136038
C	-3.809385	1.910039	-1.271879
O	-3.842453	4.694486	-3.738889
C	-5.228653	4.920196	-3.931694
Si	-0.490094	1.885568	2.037718
C	0.710771	1.560237	3.522575
C	0.117377	1.564160	4.809786
C	0.893236	1.581940	5.980069
C	2.293486	1.645420	5.889773
C	2.901286	1.635624	4.623525
C	2.118950	1.547989	3.460389
N	-2.881965	-0.956508	0.565375
C	-4.314142	-0.818367	0.527379
C	-5.005300	-0.130800	1.586169
C	-6.412025	-0.111781	1.619746
C	-7.174377	-0.745379	0.635507
C	-6.509938	-1.351941	-0.432741
C	-5.100303	-1.383743	-0.539582
C	-4.276289	0.667894	2.663636
C	-4.450182	2.183269	2.418944
C	-4.601496	-2.004735	-1.862938
C	-3.416446	-1.310483	-2.539785
C	-0.038963	-1.912899	-2.603481
C	-0.464739	-3.008862	-3.387633
C	-0.902218	-2.820577	-4.706210
C	-0.949253	-1.525988	-5.253528
C	-0.549040	-0.427927	-4.477158
C	-0.084404	-0.621971	-3.165056
C	4.865911	-0.690989	3.220841
C	3.564740	-0.840399	-3.577542
C	3.682077	2.904450	-3.225004
C	1.410520	4.958656	3.010800

C	-3.482602	-3.732688	1.556723
C	-4.181964	-3.527698	2.916196
C	-3.018961	-5.194430	1.382970
C	-4.695030	0.309654	4.103756
C	-4.396950	-3.535506	-1.813514
C	-1.528423	-1.295794	3.881268
H	-1.974430	1.453317	1.323004
H	5.776563	0.877405	0.587059
H	4.685001	3.414721	0.183151
H	-0.510836	-4.076367	0.274469
H	-1.531948	-3.453306	-1.003230
H	-4.250892	-3.536189	0.788390
H	-0.123820	-1.587407	2.288258
H	-1.551742	2.470169	2.965900
H	-0.094855	3.010249	1.156191
H	-0.976332	1.582799	4.902431
H	0.401614	1.574774	6.962080
H	2.907558	1.699463	6.798423
H	3.992625	1.692295	4.542352
H	2.614233	1.482164	2.489059
H	1.605660	-4.111021	1.067664
H	2.874374	-6.214829	0.837366
H	3.745009	-6.933439	-1.409119
H	3.388876	-5.437223	-3.395688
H	2.109575	-3.339538	-3.168475
H	0.230807	0.231172	-2.550979
H	-0.608248	0.584670	-4.889741
H	-1.309945	-1.373318	-6.278954
H	-1.221809	-3.684512	-5.303558
H	-0.443038	-4.022096	-2.968435
H	-0.763646	-1.173539	4.672528
H	-1.807637	-0.282715	3.553841
H	-2.422568	-1.749483	4.343102
H	0.334349	-3.311649	4.047234
H	-1.260110	-4.054165	3.783875
H	-0.020019	-4.147129	2.513081
H	-7.103212	-1.808918	-1.237214
H	-8.271057	-0.740626	0.682360
H	-6.917798	0.415096	2.438544
H	-5.455171	-1.859038	-2.554760
H	-3.208563	0.434161	2.570077
H	-4.036690	0.828224	4.826181
H	-5.733809	0.622771	4.320349
H	-4.621514	-0.774269	4.295728
H	-3.887616	2.764048	3.172640
H	-4.079095	2.474422	1.420966
H	-5.517262	2.470121	2.484569
H	-4.271751	-3.928777	-2.840669
H	-3.496832	-3.816058	-1.245508
H	-5.262573	-4.045205	-1.351729
H	-3.236252	-1.756232	-3.533282
H	-3.599774	-0.233706	-2.674589
H	-2.493194	-1.424415	-1.959745
H	-3.864047	-5.892248	1.553258
H	-2.627965	-5.393707	0.368079
H	-2.223754	-5.466220	2.102222
H	-5.035277	-4.228231	3.021112
H	-3.502694	-3.713822	3.768702
H	-4.584254	-2.504509	3.013004
H	-4.519712	1.291738	-0.716291

H	-5.415489	3.000323	-2.218171
H	-1.391068	4.201720	-3.283107
H	-0.475937	2.449469	-1.738838
H	0.842540	4.871231	-3.248083
H	0.258964	6.660496	-1.628205
H	0.993883	6.467074	0.744579
H	5.388525	-2.879400	-2.561346
H	5.771155	-4.533143	-0.739741
H	5.092808	-3.981732	1.591369
H	2.205346	1.557814	-2.369381
H	2.768060	3.515997	2.189086
H	1.832881	4.775920	4.015491
H	1.170960	6.036070	2.937432
H	0.467710	4.394685	2.936959
H	4.108693	5.434695	3.019767
H	4.446380	5.195416	1.278891
H	3.367341	6.512470	1.798024
H	4.044800	2.212110	-4.004894
H	3.727043	3.932434	-3.631230
H	4.371351	2.843823	-2.366328
H	1.664093	1.635461	-4.712013
H	0.288649	2.397196	-3.866506
H	1.486764	3.406078	-4.724914
H	3.972032	0.550080	-2.009914
H	4.234263	-2.714844	3.109374
H	3.533770	-0.070161	-4.369712
H	3.959723	-1.768773	-4.029836
H	2.530113	-1.028803	-3.249915
H	5.820728	0.795835	-3.647681
H	6.466604	0.393679	-2.028488
H	6.385717	-0.862106	-3.297693
H	4.684970	-0.646936	4.309685
H	5.935146	-0.920910	3.053904
H	4.662172	0.307876	2.807607
H	1.932323	-2.484566	2.499902
H	2.226222	-1.331730	3.834458
H	2.069546	-0.754220	2.155965
H	-5.315185	5.742385	-4.662722
H	-5.741828	4.021639	-4.335446
H	-5.734013	5.219736	-2.989132

Table S115. Cartesian geometry of 3f-TS3 (75.0 kcal/mol) in Figure S146 in Angstrom [Å].

Atomtype	X Coordinates	Y Coordinates	Z Coordinates
C	2.613697	-3.381902	-2.883526
C	2.075818	-3.251205	-1.583756
C	2.489827	-4.190954	-0.611012
C	3.328743	-5.265536	-0.940903
C	3.803432	-5.415132	-2.254196
C	3.455830	-4.455565	-3.217577
P	0.755329	-1.976422	-1.110386
C	-0.542649	-3.252981	-0.537380
Si	-1.734750	-2.879758	0.916153
C	-0.567916	-2.695727	2.438048
C	0.126653	-4.034348	2.774576
Ni	0.750430	0.022572	0.151194
C	2.395017	1.084826	0.199933
N	3.702969	0.575077	0.289904
C	4.651016	1.580734	0.485618
C	3.982265	2.761647	0.491123
N	2.628180	2.472314	0.312743

C	4.228997	-0.696293	-0.192701
C	4.600593	-1.737889	0.698930
C	5.321647	-2.827542	0.173150
C	5.677153	-2.898099	-1.176550
C	5.304877	-1.864346	-2.035941
C	4.600581	-0.740525	-1.564638
C	1.732388	3.598522	0.098601
C	1.330037	3.903611	-1.232622
C	0.556225	5.060370	-1.445067
C	0.187642	5.897374	-0.386691
C	0.648648	5.620263	0.902607
C	1.451484	4.493116	1.171521
C	4.303803	-1.751141	2.195049
C	2.793323	-1.744771	2.472233
C	4.368986	0.421307	-2.527960
C	5.719091	1.026354	-2.972117
C	1.843574	3.115004	-2.436215
C	0.916263	3.140334	-3.664161
C	2.055580	4.358676	2.569183
C	3.192630	5.390611	2.764118
Ge	-1.553838	0.095630	0.207847
C	-2.592634	1.494262	-0.743058
C	-1.865074	2.548332	-1.324505
C	-2.494533	3.609931	-1.986324
C	-3.912024	3.665021	-2.102735
C	-4.649686	2.607336	-1.495668
C	-4.001117	1.560333	-0.829641
N	-4.544378	4.706304	-2.770685
C	-3.757835	5.802773	-3.304251
Si	-0.525194	1.413540	2.339393
C	0.769644	1.019353	3.728845
C	0.236000	0.773162	5.018670
C	1.055124	0.711470	6.157606
C	2.435197	0.944780	6.039883
C	2.985190	1.183536	4.769709
C	2.167069	1.174395	3.627969
N	-2.689936	-1.433432	0.532975
C	-4.128611	-1.451860	0.562107
C	-4.837017	-1.005131	1.732378
C	-6.234753	-1.146940	1.812543
C	-6.972043	-1.712756	0.769609
C	-6.298857	-2.082277	-0.397012
C	-4.900320	-1.941903	-0.551341
C	-4.144613	-0.294195	2.892095
C	-4.481341	1.213398	2.874333
C	-4.400764	-2.305991	-1.966582
C	-3.329715	-1.392468	-2.569208
C	0.092312	-1.577959	-2.815083
C	-0.248548	-2.585372	-3.745971
C	-0.768423	-2.253136	-5.004808
C	-0.985623	-0.905401	-5.342378
C	-0.671629	0.102163	-4.417886
C	-0.123170	-0.231279	-3.167579
C	5.103193	-0.681291	2.964922
C	3.504945	0.024456	-3.739047
C	3.232454	3.668000	-2.834921
C	1.035499	4.510868	3.717352
C	-2.937806	-4.371597	1.122377
C	-3.592026	-4.444114	2.517476
C	-2.327164	-5.730906	0.721307

C	-4.459304	-0.894936	4.276825
C	-4.029604	-3.794511	-2.150229
C	-1.149231	-2.089067	3.724428
C	-5.994692	4.757151	-2.825968
H	-1.987855	0.909495	1.634481
H	5.713635	1.355743	0.563529
H	4.339219	3.784941	0.583185
H	-0.007957	-4.183936	-0.284892
H	-1.146758	-3.502317	-1.425956
H	-3.757557	-4.155998	0.414719
H	0.198586	-1.986110	2.060569
H	-1.594746	1.745078	3.379808
H	-0.300583	2.694175	1.626244
H	-0.848398	0.656069	5.142815
H	0.609807	0.509314	7.140962
H	3.078521	0.938066	6.929709
H	4.059653	1.373689	4.667855
H	2.624477	1.302954	2.644415
H	2.137726	-4.099274	0.422657
H	3.614116	-5.986705	-0.163790
H	4.449136	-6.262206	-2.519248
H	3.837602	-4.540552	-4.243820
H	2.351078	-2.659241	-3.661066
H	0.124475	0.551169	-2.438952
H	-0.864817	1.151227	-4.665109
H	-1.412326	-0.643313	-6.319216
H	-1.020233	-3.048664	-5.718317
H	-0.096219	-3.640739	-3.489521
H	-0.365350	-2.004661	4.501935
H	-1.543751	-1.073994	3.564540
H	-1.971004	-2.698945	4.138319
H	0.926271	-3.882051	3.525650
H	-0.589774	-4.755908	3.209320
H	0.587453	-4.522601	1.897173
H	-6.878228	-2.481398	-1.241443
H	-8.059806	-1.835894	0.850872
H	-6.753747	-0.802251	2.715702
H	-5.296795	-2.159888	-2.602526
H	-3.064074	-0.397449	2.733247
H	-3.825067	-0.416056	5.046701
H	-5.513660	-0.728596	4.568030
H	-4.267381	-1.981075	4.307049
H	-3.943408	1.739621	3.684056
H	-4.191269	1.678333	1.916214
H	-5.567485	1.372630	3.016938
H	-3.909519	-4.019593	-3.227453
H	-3.079092	-4.048261	-1.656447
H	-4.812475	-4.460849	-1.743387
H	-3.160296	-1.659006	-3.626897
H	-3.626081	-0.333447	-2.524675
H	-2.369328	-1.495406	-2.050564
H	-3.082954	-6.537659	0.811634
H	-1.963878	-5.736845	-0.323042
H	-1.475147	-6.010585	1.368897
H	-4.358557	-5.245201	2.543592
H	-2.858024	-4.671355	3.312731
H	-4.098106	-3.497809	2.775665
H	-4.622820	0.780891	-0.380095
H	-5.743288	2.595812	-1.530484
H	-1.871351	4.401501	-2.406101

H	-0.772042	2.553540	-1.240041
H	0.248055	5.318797	-2.462922
H	-0.436177	6.780779	-0.573842
H	0.399422	6.301757	1.723922
H	5.579689	-1.916998	-3.095722
H	6.226377	-3.765933	-1.558534
H	5.603549	-3.644846	0.848865
H	1.951508	2.060335	-2.127579
H	2.495788	3.355923	2.653453
H	1.506943	4.214748	4.671931
H	0.694042	5.557986	3.821143
H	0.147603	3.876068	3.571411
H	3.672109	5.248508	3.751070
H	3.975709	5.311848	1.989190
H	2.792892	6.421546	2.724043
H	3.621872	3.135901	-3.720514
H	3.149014	4.741510	-3.089049
H	3.969343	3.563795	-2.021027
H	1.272206	2.406812	-4.408372
H	-0.122816	2.887138	-3.401078
H	0.914695	4.131281	-4.156039
H	3.821950	1.206100	-1.989863
H	4.683195	-2.723737	2.566303
H	3.352657	0.894612	-4.403515
H	3.981704	-0.773225	-4.338075
H	2.513323	-0.331170	-3.417183
H	5.558376	1.904533	-3.623304
H	6.314239	1.351321	-2.099155
H	6.321022	0.291480	-3.538333
H	4.967474	-0.816573	4.052791
H	6.181885	-0.766776	2.734380
H	4.779228	0.339070	2.712190
H	2.345017	-2.665375	2.069845
H	2.575361	-1.694598	3.553253
H	2.280529	-0.899329	1.985822
H	-3.184195	6.334647	-2.513150
H	-3.025520	5.455050	-4.063981
H	-4.421430	6.535837	-3.791372
H	-6.314477	5.634955	-3.411166
H	-6.418778	3.854918	-3.314282
H	-6.454059	4.833688	-1.816209

Table S116. Cartesian geometry of 3g-TS3 (83.1 kcal/mol) in Figure S146 in Angstrom [Å].

Atomtype	X Coordinates	Y Coordinates	Z Coordinates
C	0.292084	2.178296	-2.614764
C	0.424261	2.944641	-1.442378
C	0.947915	4.252973	-1.545052
C	1.313088	4.783319	-2.790070
C	1.186344	4.003307	-3.954051
C	0.682201	2.697309	-3.861556
P	-0.075240	2.182742	0.195735
Ni	-0.648259	-0.038325	-0.094688
Ge	1.448426	-0.730367	-0.674623
N	3.099084	-0.091242	0.155990
C	4.418434	-0.470789	-0.302510
C	4.940053	-1.782557	0.013664
C	6.189348	-2.202243	-0.477145
C	6.957543	-1.391707	-1.310964
C	6.472693	-0.123279	-1.614714
C	5.250896	0.389621	-1.112759

C	4.213584	-2.804785	0.885953
C	4.964612	-3.088777	2.205031
C	5.016307	1.833825	-1.630368
C	6.175005	2.786729	-1.224802
C	-0.954788	3.699198	0.937046
C	-1.554663	4.691253	0.129326
C	-2.045824	5.885798	0.680515
C	-1.988611	6.109742	2.064095
C	-1.457894	5.107746	2.892244
C	-0.955402	3.922336	2.334471
C	1.531174	2.385516	1.170646
Si	2.730434	0.932071	1.568904
C	4.378223	1.730144	2.161399
C	4.203508	3.048250	2.945251
C	1.759530	0.038961	2.991157
C	2.294757	-1.312730	3.487985
C	1.543595	0.946972	4.225043
C	-2.515062	-0.578657	-0.274238
N	-3.619291	0.086450	0.263361
C	-4.820460	-0.573845	-0.004793
C	-4.511827	-1.668557	-0.746331
N	-3.125512	-1.677369	-0.905679
C	-3.737140	1.479744	0.656422
C	-3.738771	1.869178	2.019199
C	-4.158649	3.181306	2.319943
C	-4.581991	4.066311	1.324987
C	-4.545518	3.668934	-0.014944
C	-4.117942	2.380259	-0.377914
C	-2.509072	-2.695898	-1.724427
C	-1.865973	-2.300156	-2.930660
C	-1.251480	-3.298941	-3.707247
C	-1.325100	-4.650252	-3.346601
C	-2.047717	-5.026547	-2.211943
C	-2.651753	-4.067823	-1.372514
C	-3.380429	0.950950	3.187022
C	-4.427054	-0.167298	3.377124
C	-4.136331	1.979445	-1.853104
C	-3.249174	2.901953	-2.711638
C	-1.994022	-0.873896	-3.463988
C	-3.412728	-0.691440	-4.054708
C	-3.439153	-4.574150	-0.160561
C	-2.643633	-5.576474	0.704066
C	-1.931784	0.430677	3.131411
C	-5.577581	1.926490	-2.402362
C	-0.936209	-0.488021	-4.506487
C	-4.769668	-5.222987	-0.609811
N	1.828200	-1.165342	-2.501084
C	1.985626	-2.547567	-2.924449
Si	0.391589	-2.901459	0.565639
C	-0.650528	-3.009797	2.191675
C	-0.049153	-3.720082	3.260460
C	-0.787066	-4.115330	4.388348
C	-2.163119	-3.843616	4.453440
C	-2.776503	-3.140950	3.402839
C	-2.020149	-2.697370	2.307020
C	2.694121	-0.279644	-3.265272
C	3.963009	-4.131262	0.137629
C	3.711440	2.570233	-1.336520
C	5.305792	0.757569	2.915860
H	1.747491	-2.401251	-0.294664

H	-5.776880	-0.177357	0.332624
H	-5.144718	-2.433943	-1.189342
H	1.245415	2.748919	2.169533
H	2.070749	3.231972	0.717980
H	4.889565	1.975770	1.212058
H	0.766850	-0.141725	2.520762
H	1.412773	-3.903957	1.054889
H	-0.322308	-3.493666	-0.584445
H	1.010268	-3.999367	3.200159
H	-0.290218	-4.657315	5.204071
H	-2.755162	-4.178066	5.315565
H	-3.852864	-2.940594	3.437931
H	-2.504481	-2.120248	1.516532
H	-0.539129	3.172270	3.015307
H	-1.424369	5.248396	3.980625
H	-2.364381	7.047227	2.493733
H	-2.482876	6.643187	0.016370
H	-1.615036	4.553552	-0.953641
H	-0.101001	1.158920	-2.535835
H	0.599483	2.073404	-4.758741
H	1.485900	4.412272	-4.927723
H	1.714266	5.803433	-2.851185
H	1.065516	4.867954	-0.644983
H	1.638588	-1.720123	4.280972
H	2.336819	-2.069022	2.691521
H	3.310994	-1.221491	3.909775
H	0.813805	0.484154	4.918368
H	2.488109	1.078337	4.786681
H	1.164084	1.955626	3.984902
H	7.063794	0.505058	-2.292175
H	7.913909	-1.741606	-1.720321
H	6.560277	-3.198720	-0.208242
H	5.065100	1.726443	-2.734436
H	3.240409	-2.374232	1.149055
H	4.364321	-3.760043	2.848238
H	5.935403	-3.583728	2.013737
H	5.162629	-2.165065	2.772301
H	3.413021	-4.839928	0.782517
H	3.368617	-3.970195	-0.777705
H	4.912985	-4.615670	-0.154631
H	6.011239	3.785431	-1.672709
H	6.204896	2.913666	-0.126402
H	7.170012	2.434838	-1.544321
H	3.582398	3.390548	-2.062389
H	2.825994	1.932075	-1.388482
H	3.731341	3.032241	-0.337876
H	5.192354	3.491322	3.182028
H	3.631101	3.805894	2.379165
H	3.681202	2.889669	3.907067
H	6.277658	1.242192	3.141367
H	4.870413	0.437458	3.881492
H	5.519939	-0.143579	2.315789
H	-0.718826	-3.020785	-4.620898
H	-0.835957	-5.411942	-3.967198
H	-2.133198	-6.088520	-1.955463
H	-4.851518	4.373008	-0.797930
H	-4.911590	5.076567	1.593250
H	-4.158080	3.506851	3.367298
H	-1.875827	-0.184874	-2.607089
H	-3.682452	-3.714120	0.483080

H	-3.192159	-5.779637	1.641746
H	-2.502943	-6.541181	0.182244
H	-1.652861	-5.181816	0.977915
H	-5.360956	-5.541611	0.269475
H	-5.392396	-4.537981	-1.213670
H	-4.574381	-6.117649	-1.230431
H	-3.548000	0.340982	-4.424202
H	-3.560924	-1.383554	-4.905339
H	-4.202480	-0.892048	-3.310608
H	-1.066517	0.573637	-4.778286
H	0.077102	-0.633743	-4.095271
H	-1.042960	-1.072835	-5.439393
H	-3.710854	0.969197	-1.937268
H	-3.450730	1.582766	4.094430
H	-3.263321	2.574564	-3.767658
H	-3.598471	3.950933	-2.684627
H	-2.203316	2.874654	-2.367365
H	-5.577924	1.574913	-3.450828
H	-6.206618	1.237717	-1.810378
H	-6.049840	2.926701	-2.378066
H	-4.232834	-0.713814	4.317504
H	-5.446443	0.259314	3.426378
H	-4.400462	-0.895954	2.552185
H	-1.218123	1.257135	3.269451
H	-1.746151	-0.317776	3.921255
H	-1.692602	-0.038238	2.162770
H	3.021543	-2.936645	-2.776720
H	1.281594	-3.216694	-2.404322
H	1.759071	-2.632980	-4.010919
H	3.774919	-0.531348	-3.172960
H	2.431557	-0.336455	-4.346722
H	2.562861	0.770244	-2.964535

Table S117. Cartesian geometry of 3a-INT2 (14.1 kcal/mol) in Figure S146 in Angstrom [Å].

Atomtype	X Coordinates	Y Coordinates	Z Coordinates
C	3.493087	2.175935	-1.298465
C	2.124901	1.848987	-1.140760
C	1.177654	2.863057	-1.396720
C	1.561646	4.162352	-1.748175
C	2.934850	4.481272	-1.865175
C	3.901108	3.468940	-1.648642
Ge	1.484974	0.017959	-0.599537
Ni	-0.863543	-0.034406	-0.288643
P	-0.999180	0.227362	1.906859
C	0.284474	-0.615075	2.974710
Si	1.868423	-1.409903	2.202754
C	2.960739	-1.620837	3.783343
C	2.246892	-2.485607	4.850219
Si	2.114933	-1.096235	-2.709753
C	2.389050	-2.968153	-2.983053
C	2.989866	-3.836749	-2.044573
C	3.168530	-5.203603	-2.315628
C	2.741890	-5.740004	-3.541561
C	2.139210	-4.897576	-4.491977
C	1.966728	-3.530970	-4.214059
N	2.625977	-0.402987	0.966723
C	3.897223	0.228681	1.174969
C	4.014448	1.419602	1.964054
C	5.289809	1.971150	2.203514
C	6.451379	1.409833	1.666028

C	6.339332	0.272734	0.858362
C	5.092654	-0.326586	0.601066
C	2.810682	2.166139	2.537917
C	2.710832	3.595839	1.962131
C	5.070222	-1.550646	-0.307285
C	5.851850	-2.749664	0.271441
C	2.843246	2.238763	4.079992
C	5.616196	-1.234730	-1.717046
C	-2.569280	-0.104773	-1.196958
N	-3.357269	-1.205831	-1.470506
C	-4.222655	-0.977935	-2.545252
C	-4.020605	0.307767	-2.941573
N	-3.033102	0.830066	-2.105372
C	-3.512915	-2.364806	-0.629164
C	-4.678219	-2.411595	0.181433
C	-4.863930	-3.546719	0.993502
C	-3.929617	-4.586803	0.992541
C	-2.803045	-4.526328	0.158740
C	-2.566127	-3.421470	-0.685594
C	-2.655024	2.217360	-2.137174
C	-3.218826	3.085744	-1.159475
C	-2.897162	4.452573	-1.252473
C	-2.072842	4.934495	-2.281639
C	-1.548937	4.058725	-3.240085
C	-1.821479	2.676464	-3.187153
C	-5.757730	-1.325356	0.143441
C	-7.034854	-1.866468	-0.537658
C	-1.356988	-3.377709	-1.631722
C	-0.425518	-4.591165	-1.481209
C	-4.192161	2.558504	-0.098315
C	-5.600192	2.358002	-0.710480
C	-1.286964	1.734072	-4.267011
C	-2.131358	1.868305	-5.556153
C	-6.082534	-0.736076	1.526076
C	-1.759273	-3.266754	-3.123256
C	-4.317408	3.438225	1.158709
C	0.203212	1.951538	-4.594322
C	-0.796843	2.030767	2.315918
C	-0.867886	2.961193	1.264705
C	-0.792101	4.340857	1.511154
C	-0.668565	4.809456	2.827613
C	-0.597926	3.889279	3.890232
C	-0.649613	2.510225	3.636529
C	-2.537201	-0.192063	2.903158
C	-2.898668	-1.553329	3.023538
C	-3.925669	-1.953754	3.890274
C	-4.640543	-0.998739	4.632080
C	-4.342989	0.362571	4.464551
C	-3.304346	0.764106	3.604972
C	1.371929	-3.102704	1.423590
C	0.092569	-3.712960	2.027784
C	2.519387	-4.133465	1.457784
C	4.420503	-2.085762	3.626318
H	-0.389533	0.042571	-1.681625
H	1.165753	-2.872535	0.357467
H	-4.903920	-1.747994	-2.902709
H	-4.493769	0.906457	-3.717613
H	-3.088261	1.831470	3.499325
H	-1.390607	0.700566	-3.889120
H	-0.243946	-1.417305	3.517759

H	0.617878	0.099534	3.749463
H	3.726500	2.803736	4.432376
H	2.884915	1.238268	4.544299
H	1.944002	2.756140	4.458059
H	2.685760	-4.512713	2.483143
H	3.475782	-3.718279	1.102388
H	2.277845	-5.005674	0.818495
H	-1.839747	6.005828	-2.336532
H	7.243209	-0.168510	0.419168
H	1.899479	1.625306	2.234215
H	3.001099	-0.586230	4.176483
H	-0.791125	-2.452678	-1.377765
H	-1.017365	2.589615	0.247720
H	7.432692	1.859312	1.865860
H	-0.600348	1.807368	4.477031
H	5.370759	2.876327	2.818403
H	4.265938	1.421158	-1.130398
H	-5.750863	-3.609245	1.634980
H	-2.375729	-2.317303	2.439001
H	-3.299184	5.154225	-0.514543
H	4.010510	-1.840735	-0.395985
H	4.967090	3.704736	-1.747920
H	-3.811877	1.571615	0.227381
H	-5.387806	-0.487433	-0.467543
H	0.111219	2.627206	-1.343256
H	-0.907835	4.450135	-4.037620
H	-4.081260	-5.462409	1.637070
H	-4.917174	1.124181	5.008235
H	-0.850012	-3.320305	-3.747982
H	-2.269326	-2.322128	-3.363570
H	-2.420926	-4.107220	-3.408617
H	-0.058381	-4.753619	1.680606
H	-0.802127	-3.145166	1.726112
H	0.123172	-3.750341	3.133125
H	-2.100320	-5.363043	0.169237
H	5.146725	-0.338208	-2.152508
H	5.428014	-2.082683	-2.401450
H	6.708351	-1.060512	-1.684975
H	-5.441453	-1.311485	5.314015
H	-4.174925	-3.019042	3.969472
H	1.821921	4.105245	2.367627
H	2.629216	3.584210	0.865356
H	3.598723	4.197529	2.232352
H	-0.492129	4.248197	4.921973
H	0.385694	2.946836	-5.040582
H	0.537161	1.193454	-5.325404
H	0.834245	1.857591	-3.697737
H	-0.613451	5.886782	3.028627
H	-0.841346	5.040129	0.668847
H	-6.441542	-1.504117	2.233837
H	-6.878101	0.026724	1.431836
H	-5.196247	-0.257717	1.969173
C	3.336874	5.817756	-2.205578
H	-0.949744	-5.524182	-1.763249
H	-0.037613	-4.708372	-0.458531
H	0.436896	-4.483998	-2.152329
H	-3.337961	3.660149	1.610212
H	-4.930885	2.910359	1.911080
H	-4.824083	4.396676	0.936810
H	-3.204480	1.681862	-5.374919

H	-1.783047	1.150275	-6.322081
H	-2.035260	2.888121	-5.974900
H	4.936328	-2.049827	4.607521
H	4.989180	-1.449342	2.929838
H	4.479540	-3.130598	3.272589
H	6.933864	-2.524824	0.322767
H	5.725994	-3.636809	-0.377897
H	5.520529	-3.011965	1.287510
H	0.803432	4.930287	-1.934344
H	2.154899	-3.538435	4.522799
H	1.232669	-2.123135	5.097548
H	2.830173	-2.492426	5.793053
H	-6.822344	-2.272770	-1.543321
H	-7.787523	-1.062186	-0.641925
H	-7.486468	-2.679346	0.061849
H	-6.010035	3.328860	-1.047716
H	-6.287340	1.938644	0.046565
H	-5.590062	1.672863	-1.573268
H	3.262275	-0.382211	-3.368244
H	0.924018	-0.788175	-3.576862
H	1.486357	-2.893211	-4.969283
H	1.797812	-5.307265	-5.451947
H	2.873383	-6.808843	-3.754860
H	3.632074	-5.850587	-1.559263
H	3.306053	-3.453624	-1.072559
N	3.654286	6.910812	-2.484788

Table S118. Cartesian geometry of 3b-INT2 (16.6 kcal/mol) in Figure S146 in Angstrom [Å].

Atomtype	X Coordinates	Y Coordinates	Z Coordinates
C	1.755610	-4.314561	-2.469649
C	1.479454	-3.167681	-3.247720
C	1.037668	-3.375954	-4.579024
C	0.885307	-4.669462	-5.106938
C	1.172028	-5.793493	-4.313152
C	1.608596	-5.610835	-2.990699
Si	1.704126	-1.363999	-2.654422
Ge	1.146762	-0.426202	-0.435652
N	1.979714	-1.416732	1.082520
C	3.345242	-1.233321	1.485786
C	3.698328	-0.242536	2.459599
C	5.037074	-0.134292	2.887018
C	6.045989	-0.948332	2.365814
C	5.716697	-1.886246	1.382062
C	4.394648	-2.045076	0.928683
C	2.692726	0.752990	3.038020
C	2.509084	0.583705	4.561524
C	4.141132	-3.074084	-0.169271
C	4.916371	-2.759000	-1.468643
Ni	-1.172731	0.023249	-0.293334
C	-2.787486	0.482825	-1.254886
N	-3.784412	-0.337006	-1.748384
C	-4.488734	0.267941	-2.794591
C	-3.969904	1.516922	-2.941938
N	-2.959029	1.640394	-1.988858
C	-4.281664	-1.539185	-1.128538
C	-5.483558	-1.422382	-0.379765
C	-5.997513	-2.590768	0.214762
C	-5.348430	-3.819178	0.056754
C	-4.184330	-3.910103	-0.720378
C	-3.620389	-2.776554	-1.341662

C	-2.291691	2.893160	-1.755994
C	-2.761737	3.713136	-0.692470
C	-2.188227	4.991818	-0.564744
C	-1.208146	5.437257	-1.463013
C	-0.765423	4.607281	-2.499054
C	-1.290954	3.311165	-2.666172
C	-6.275751	-0.114697	-0.281540
C	-6.585751	0.316214	1.161432
C	-2.367609	-2.880123	-2.222981
C	-2.626693	-2.454660	-3.689619
C	-3.898353	3.253622	0.226225
C	-3.902193	3.916528	1.615971
C	-0.841226	2.436297	-3.836721
C	0.691423	2.351221	-3.969171
P	-1.450560	-0.010927	1.900885
C	-0.841467	1.606361	2.583473
C	-0.600131	2.662440	1.685262
C	-0.219440	3.930962	2.151646
C	-0.078829	4.157628	3.529233
C	-0.311263	3.108579	4.436909
C	-0.684893	1.840571	3.967200
C	-3.121245	-0.155713	2.753022
C	-3.811606	-1.385948	2.657403
C	-4.964698	-1.628936	3.417264
C	-5.477804	-0.633798	4.265706
C	-4.845457	0.618125	4.313743
C	-3.679759	0.857060	3.563179
C	-0.514720	-1.291800	2.884748
Si	0.869466	-2.365616	2.074564
C	0.026019	-3.713371	0.983597
C	0.833982	-5.025575	0.913697
C	1.687997	-3.107501	3.664686
C	2.970620	-3.949125	3.534485
C	0.651598	-3.875241	4.521597
C	-7.575206	-0.221531	-1.111725
C	-1.755551	-4.289452	-2.240688
C	-5.268197	3.494224	-0.453095
C	-1.465577	2.944936	-5.156417
C	2.416299	1.155205	-0.621799
C	2.028934	2.510361	-0.693022
C	2.997348	3.532333	-0.822711
C	4.364504	3.225596	-0.866626
C	4.761219	1.883156	-0.807789
C	3.798647	0.869425	-0.705643
C	3.106284	2.210913	2.734717
C	4.488817	-4.517044	0.258085
C	-1.430622	-4.008418	1.387745
H	-0.590228	0.206418	-1.635394
H	0.011156	-3.278840	-0.037771
H	-5.297841	-0.246689	-3.309615
H	-4.228464	2.334085	-3.613148
H	-3.199509	1.837175	3.631130
H	-1.209920	1.410744	-3.652715
H	-1.271110	-1.989133	3.281989
H	-0.070255	-0.798143	3.767734
H	3.470171	0.719433	5.092027
H	2.123859	-0.416029	4.826690
H	1.801635	1.337056	4.949475
H	0.779001	-5.580446	1.868787
H	1.899411	-4.859342	0.691701

H	0.430443	-5.689213	0.123268
H	-0.771508	6.435825	-1.344180
H	6.506950	-2.511041	0.947342
H	1.720675	0.575580	2.548337
H	1.968399	-2.196381	4.228852
H	-1.624610	-2.165114	-1.801943
H	-0.747697	2.489076	0.612886
H	7.083461	-0.836052	2.704873
H	-0.876499	1.036372	4.687756
H	5.297065	0.623585	3.636599
H	4.150044	-0.161278	-0.684621
H	-6.919622	-2.532185	0.805057
H	-3.448588	-2.172476	1.988890
H	-2.513166	5.652633	0.245530
H	3.060023	-3.023589	-0.381186
C	6.240010	1.552971	-0.793527
H	-3.773649	2.165134	0.381611
H	-5.670127	0.693352	-0.719647
H	0.971022	2.788997	-0.655553
H	0.008337	4.968529	-3.182271
H	-5.757193	-4.721509	0.529823
H	-5.253177	1.418981	4.944426
H	-1.708101	-2.616743	-4.281323
H	-2.901852	-1.394124	-3.789148
H	-3.432528	-3.070736	-4.133089
H	-1.821068	-4.900665	0.861947
H	-2.093638	-3.169828	1.121701
H	-1.539104	-4.207477	2.470665
H	-3.711021	-4.887650	-0.840499
H	4.769786	-1.725238	-1.814704
H	4.589066	-3.436128	-2.279171
H	6.002725	-2.904058	-1.320600
H	-6.378174	-0.823863	4.863621
H	-5.470583	-2.598307	3.328261
H	2.295563	2.902966	3.020826
H	3.321662	2.354543	1.665658
H	4.012526	2.494834	3.302352
H	-0.196572	3.276931	5.515389
H	1.146038	3.338383	-4.169364
H	0.960045	1.679757	-4.804446
H	1.148802	1.945431	-3.053819
H	0.218473	5.147826	3.897298
H	-0.043157	4.736126	1.434070
H	-7.154968	-0.451517	1.714734
H	-7.195192	1.239188	1.154398
H	-5.660538	0.518953	1.720937
H	5.109946	4.021568	-0.952626
H	-2.459624	-5.016178	-2.688907
H	-1.478338	-4.648839	-1.238543
H	-0.847781	-4.292264	-2.857325
H	-2.923090	3.834546	2.114723
H	-4.658333	3.423692	2.253106
H	-4.172675	4.987714	1.555847
H	-2.568591	2.981290	-5.105120
H	-1.182717	2.284931	-5.997923
H	-1.106008	3.965823	-5.386631
H	3.359809	-4.202078	4.541761
H	3.768818	-3.414997	2.995836
H	2.777447	-4.906007	3.017503
H	5.578041	-4.624558	0.418328

H	4.198113	-5.230290	-0.536499
H	3.987547	-4.812735	1.190924
C	2.595985	4.983298	-0.966821
H	0.303887	-4.792912	4.010446
H	-0.240714	-3.274264	4.772687
H	1.108428	-4.195391	5.479694
H	-7.371223	-0.514214	-2.157721
H	-8.107836	0.748232	-1.121696
H	-8.255863	-0.979686	-0.680400
H	-5.420503	4.576231	-0.627314
H	-6.085783	3.131072	0.195378
H	-5.349990	2.977731	-1.423378
H	3.089394	-0.948332	-3.066519
H	0.782613	-0.587648	-3.555053
H	0.802892	-2.512462	-5.216852
H	0.537745	-4.800063	-6.140408
H	1.050571	-6.804927	-4.722336
H	1.826016	-6.478836	-2.354408
H	2.072940	-4.205205	-1.431018
F	6.489124	0.284297	-1.194540
F	6.944293	2.382416	-1.607822
F	6.765003	1.692401	0.451601
F	3.588231	5.831280	-0.605244
F	2.276045	5.292843	-2.257366
F	1.511968	5.299303	-0.211416

Table S119. Cartesian geometry of 3c-INT2 (14.1 kcal/mol) in Figure S146 in Angstrom [Å].

Atomtype	X Coordinates	Y Coordinates	Z Coordinates
C	3.426559	3.794671	1.186530
C	2.701936	3.276605	2.283246
C	2.317835	4.189573	3.297957
C	2.643894	5.554160	3.218497
C	3.366867	6.043504	2.116624
C	3.758895	5.157041	1.100165
Si	2.217833	1.437651	2.497654
Ge	1.506397	-0.150287	0.748112
N	2.720950	-0.272639	-0.820207
C	3.916663	-1.063620	-0.793784
C	3.913205	-2.428706	-1.229776
C	5.124407	-3.149372	-1.265749
C	6.333960	-2.583513	-0.852964
C	6.335317	-1.267244	-0.378031
C	5.157349	-0.499115	-0.337288
C	2.639765	-3.175977	-1.624025
C	2.669454	-3.653790	-3.091827
C	5.253398	0.916874	0.219845
C	5.734470	0.928148	1.687423
Ni	-0.810860	0.086661	0.294734
C	-2.510911	0.596142	1.061553
N	-3.168580	1.809166	0.990446
C	-4.078347	1.975771	2.039563
C	-4.038481	0.831456	2.774382
N	-3.101630	-0.001361	2.161314
C	-3.165365	2.707071	-0.136116
C	-4.293076	2.653489	-0.997934
C	-4.319535	3.535070	-2.095420
C	-3.272713	4.435858	-2.313746
C	-2.188730	4.490876	-1.425147
C	-2.105046	3.635922	-0.306363
C	-2.886028	-1.358677	2.585529

C	-3.522729	-2.398453	1.849984
C	-3.362822	-3.712934	2.326010
C	-2.622610	-3.976899	3.489080
C	-2.021526	-2.931937	4.200268
C	-2.132013	-1.596908	3.761386
C	-5.498055	1.747561	-0.727310
C	-5.869338	0.847080	-1.916511
C	-0.934807	3.720414	0.684641
C	-1.385371	4.064467	2.125937
C	-4.399503	-2.085270	0.631557
C	-4.604215	-3.264760	-0.336206
C	-1.507493	-0.456610	4.567278
C	-0.064308	-0.743969	5.025212
P	-0.931940	-0.746617	-1.753159
C	-0.933270	-2.604429	-1.647501
C	-1.114223	-3.196833	-0.386156
C	-1.189539	-4.591095	-0.244197
C	-1.109582	-5.413683	-1.377643
C	-0.930290	-4.833938	-2.647674
C	-0.830754	-3.440792	-2.781301
C	-2.389230	-0.464501	-2.910134
C	-2.574370	0.828314	-3.451547
C	-3.540603	1.067121	-4.439519
C	-4.366857	0.024518	-4.890848
C	-4.241952	-1.247850	-4.311879
C	-3.267602	-1.489627	-3.326521
C	0.468106	-0.376400	-2.936167
Si	2.107414	0.436896	-2.312835
C	1.776438	2.315932	-2.030165
C	3.022896	3.187124	-2.289888
C	3.253668	0.113549	-3.837068
C	4.749317	0.466305	-3.735687
C	2.658801	0.721652	-5.130263
C	-6.710172	2.595750	-0.280557
C	0.138800	4.738948	0.269069
C	-5.785906	-1.563011	1.081264
C	-2.388942	-0.123115	5.793890
C	1.923479	-1.815593	1.790277
C	0.870363	-2.626553	2.262576
C	1.117310	-3.806033	2.985579
C	2.437416	-4.197049	3.261265
C	3.501254	-3.387936	2.826079
C	3.245437	-2.209783	2.104847
C	2.389008	-4.384211	-0.695316
C	6.172552	1.837246	-0.611626
C	0.572085	2.857209	-2.823633
H	-0.363655	0.325491	1.678389
H	1.532919	2.400650	-0.950555
H	-4.670953	2.882996	2.142831
H	-4.596866	0.519989	3.655127
H	-3.188012	-2.492626	-2.897218
H	-1.471393	0.432970	3.912149
H	0.052643	0.289203	-3.711906
H	0.740943	-1.311328	-3.459036
H	3.498116	-4.367159	-3.260120
H	2.805114	-2.817923	-3.799998
H	1.727094	-4.169820	-3.346443
H	3.241652	3.260576	-3.371449
H	3.926963	2.793530	-1.798416
H	2.863123	4.217445	-1.914588

H	-2.514616	-5.010585	3.842676
H	7.276043	-0.818296	-0.034157
H	1.789725	-2.485255	-1.499140
H	3.198074	-0.988007	-3.939628
H	-0.478339	2.705117	0.719263
H	-1.224783	-2.546528	0.485171
H	7.264069	-3.165590	-0.889621
H	-0.698150	-3.004314	-3.778722
H	5.113722	-4.189742	-1.614758
H	4.096243	-1.607628	1.774121
H	-5.172851	3.511487	-2.783473
H	-1.956614	1.664247	-3.108941
H	-3.824482	-4.544654	1.784286
H	4.230191	1.325270	0.181185
H	4.538579	-3.675849	3.042255
H	-3.891262	-1.280253	0.066940
H	-5.244801	1.070432	0.103037
H	-0.164705	-2.319415	2.087775
H	-1.442632	-3.154156	5.103216
H	-3.302087	5.113932	-3.176613
H	-4.903396	-2.065411	-4.627369
H	-0.492472	4.181757	2.765395
H	-2.015573	3.284950	2.579102
H	-1.943712	5.020433	2.138183
H	0.524527	3.962464	-2.779421
H	-0.377236	2.482678	-2.407495
H	0.613664	2.585823	-3.895450
H	-1.394941	5.217592	-1.614448
H	5.157508	0.232903	2.317723
H	5.631631	1.941657	2.117812
H	6.800199	0.637493	1.752745
H	-5.119237	0.209019	-5.668190
H	-3.652451	2.079945	-4.846161
H	1.447132	-4.884353	-0.973872
H	2.316867	-4.074653	0.357923
H	3.204670	-5.127085	-0.779663
H	-0.857586	-5.471757	-3.537939
H	-0.022152	-1.578077	5.750393
H	0.351679	0.150349	5.523058
H	0.589224	-0.996088	4.176449
H	-1.172515	-6.504561	-1.275619
H	-1.318252	-5.021544	0.755244
H	-6.116643	1.429589	-2.821719
H	-6.755644	0.235622	-1.662752
H	-5.042198	0.167208	-2.168724
H	2.637123	-5.122454	3.817743
H	-0.277491	5.764429	0.259964
H	0.565541	4.530161	-0.723094
H	0.963762	4.726971	0.993531
H	-3.649517	-3.712700	-0.653394
H	-5.133011	-2.906580	-1.237880
H	-5.228895	-4.058732	0.115601
H	-3.424523	0.132944	5.509260
H	-1.965377	0.733198	6.351582
H	-2.435112	-0.989382	6.481179
H	5.281837	0.122165	-4.645852
H	5.232743	-0.011455	-2.868823
H	4.907312	1.557492	-3.666522
H	7.223872	1.497200	-0.554832
H	6.135184	2.870862	-0.218105

H	5.889398	1.859189	-1.674745
H	0.272151	-4.412439	3.334350
H	2.660080	1.827649	-5.096496
H	1.621697	0.395359	-5.327691
H	3.266705	0.426715	-6.009432
H	-6.466477	3.233043	0.588946
H	-7.558379	1.941658	-0.002832
H	-7.046814	3.261381	-1.097806
H	-6.321952	-2.347532	1.648392
H	-6.396446	-1.298020	0.199092
H	-5.711798	-0.669203	1.721073
H	3.263641	0.819074	3.382853
H	0.984573	1.509681	3.358264
H	1.745667	3.831176	4.165185
H	2.328334	6.238884	4.017063
H	3.618480	7.110075	2.049897
H	4.315859	5.525299	0.228457
H	3.721994	3.134454	0.368972

Table S120. Cartesian geometry of 3d-INT2 (14.2 kcal/mol) in Figure S146 in Angstrom [Å].

Atomtype	X Coordinates	Y Coordinates	Z Coordinates
C	3.160676	3.823048	1.749547
C	2.514379	3.069284	2.754589
C	2.116233	3.757804	3.928485
C	2.354548	5.133438	4.088270
C	3.001244	5.859776	3.073055
C	3.405355	5.198132	1.901929
Si	2.148788	1.193954	2.643074
Ge	1.494049	-0.100062	0.646229
N	2.663069	0.148682	-0.943359
C	3.907560	-0.549501	-1.083965
C	3.981302	-1.807323	-1.766991
C	5.235243	-2.424647	-1.952305
C	6.416075	-1.864151	-1.457427
C	6.344910	-0.660649	-0.747107
C	5.121298	0.004735	-0.548906
C	2.750082	-2.555252	-2.277189
C	2.776296	-2.759212	-3.807441
C	5.141011	1.298758	0.256955
C	5.660802	1.076700	1.694123
Ni	-0.847242	0.052209	0.287949
C	-2.551541	0.289543	1.169668
N	-3.290572	1.444877	1.337722
C	-4.173222	1.351603	2.418632
C	-4.032666	0.097970	2.928363
N	-3.064083	-0.539550	2.152154
C	-3.384200	2.531809	0.397142
C	-4.535689	2.558620	-0.434014
C	-4.657780	3.622796	-1.347784
C	-3.678196	4.617739	-1.420813
C	-2.568345	4.583213	-0.563670
C	-2.391424	3.546770	0.376649
C	-2.745956	-1.933040	2.311321
C	-3.339093	-2.861328	1.409132
C	-3.077873	-4.226572	1.628185
C	-2.280991	-4.647748	2.704300
C	-1.723793	-3.712265	3.583755
C	-1.936699	-2.330401	3.404460
C	-5.668011	1.534421	-0.311729
C	-6.013354	0.840311	-1.639307

C	-1.194669	3.530743	1.339261
C	-1.614604	3.565966	2.829644
C	-4.275713	-2.390626	0.289929
C	-4.432083	-3.379817	-0.879150
C	-1.359603	-1.317055	4.394298
C	0.115058	-1.580089	4.756533
P	-0.974523	-0.396995	-1.873731
C	-0.852802	-2.237834	-2.116881
C	-0.960632	-3.064527	-0.985674
C	-0.940161	-4.462781	-1.105390
C	-0.837679	-5.053800	-2.373416
C	-0.731425	-4.237987	-3.515537
C	-0.726517	-2.840721	-3.388185
C	-2.484003	-0.005881	-2.926896
C	-2.775269	1.350689	-3.198329
C	-3.784025	1.706157	-4.105309
C	-4.548980	0.713893	-4.740682
C	-4.319573	-0.634371	-4.425615
C	-3.301268	-0.991835	-3.523290
C	0.357112	0.282706	-2.996638
Si	1.962088	1.076949	-2.267794
C	1.525683	2.848157	-1.640557
C	2.706122	3.833521	-1.766251
C	3.079201	1.108897	-3.845782
C	4.552164	1.538169	-3.710735
C	2.408425	1.894709	-4.998372
C	-6.920622	2.197209	0.304396
C	-0.208124	4.684725	1.098432
C	-5.678278	-2.064299	0.858505
C	-2.216595	-1.279542	5.681435
C	2.048740	-1.894014	1.346835
C	1.069397	-2.852840	1.674421
C	1.415524	-4.130017	2.145033
C	2.764016	-4.498265	2.314168
C	3.749513	-3.530704	2.024201
C	3.400604	-2.255663	1.555388
C	2.599092	-3.925188	-1.579789
C	5.974879	2.415389	-0.407161
C	0.263522	3.442062	-2.294035
H	-0.371570	0.061617	1.682949
H	1.315327	2.720764	-0.558238
H	-4.820262	2.180247	2.700836
H	-4.538600	-0.408310	3.748294
H	-3.139999	-2.050909	-3.301451
H	-1.405648	-0.321447	3.916321
H	-0.127314	1.049968	-3.624448
H	0.671250	-0.517351	-3.691953
H	3.641721	-3.377826	-4.111169
H	2.846168	-1.803175	-4.354919
H	1.860745	-3.279242	-4.140162
H	2.882892	4.114146	-2.821280
H	3.649106	3.419935	-1.374258
H	2.495283	4.766776	-1.207172
H	-2.093247	-5.718688	2.856134
H	7.263351	-0.219243	-0.339140
H	1.860247	-1.957187	-2.021460
H	3.089845	0.041618	-4.141837
H	-0.669285	2.563094	1.171910
H	-1.091072	-2.596454	-0.006719
H	7.380436	-2.365013	-1.613751

H	-0.649387	-2.218923	-4.288395
H	5.282815	-3.380887	-2.488679
H	4.203916	-1.546853	1.335930
H	-5.531838	3.666737	-2.008224
H	-2.209623	2.146543	-2.703560
H	-3.503742	-4.974305	0.951439
H	4.092893	1.635931	0.310052
H	4.810667	-3.781853	2.160702
H	-3.842389	-1.459294	-0.122403
H	-5.344315	0.737954	0.376009
H	0.010761	-2.592105	1.588921
H	-1.098815	-4.055628	4.415224
H	-3.781079	5.438447	-2.142643
H	-4.932514	-1.420807	-4.885289
H	-0.709144	3.631693	3.458740
H	-2.169198	2.669193	3.143147
H	-2.239537	4.456694	3.034187
H	0.149960	4.515680	-2.048133
H	-0.645724	2.936051	-1.931169
H	0.284030	3.371827	-3.398079
H	-1.829095	5.384765	-0.634479
H	5.145891	0.242140	2.196083
H	5.506517	1.986648	2.303381
H	6.744057	0.850706	1.688869
H	-5.335172	0.990918	-5.454514
H	-3.978474	2.767898	-4.301478
H	1.688137	-4.432022	-1.937652
H	2.526630	-3.815243	-0.487608
H	3.461717	-4.581384	-1.802898
H	-0.641662	-4.693313	-4.510171
H	0.238560	-2.529084	5.311155
H	0.488714	-0.766027	5.403388
H	0.752562	-1.622923	3.860483
H	-0.826194	-6.146416	-2.475797
H	-1.012755	-5.078637	-0.202002
H	-6.329045	1.558222	-2.416991
H	-6.846603	0.128964	-1.486040
H	-5.149337	0.281165	-2.028016
C	3.152936	-5.888812	2.767079
H	-0.693516	5.660825	1.289911
H	0.197565	4.694820	0.075987
H	0.640384	4.599367	1.789928
H	-3.460158	-3.691129	-1.292733
H	-5.011566	-2.897976	-1.687261
H	-4.987706	-4.286910	-0.573745
H	-3.276494	-1.051004	5.472955
H	-1.831425	-0.512128	6.378884
H	-2.180332	-2.258509	6.196492
H	5.078823	1.392516	-4.676043
H	5.089349	0.951677	-2.948582
H	4.643059	2.609201	-3.455669
H	7.047314	2.144152	-0.429294
H	5.880750	3.356101	0.167984
H	5.661740	2.608676	-1.444321
H	0.619583	-4.846847	2.385965
H	2.342122	2.975042	-4.768248
H	1.387956	1.540204	-5.230702
H	3.006184	1.800775	-5.927557
H	-6.693981	2.681545	1.271681
H	-7.713439	1.443795	0.472409

H	-7.326954	2.974190	-0.370502
H	-6.140119	-2.977912	1.278619
H	-6.335168	-1.684798	0.054936
H	-5.643383	-1.302545	1.653667
H	3.251581	0.497547	3.391742
H	0.934457	1.032193	3.518647
H	1.602189	3.212183	4.732256
H	2.030104	5.641368	5.006371
H	3.184163	6.935584	3.193600
H	3.902832	5.753185	1.095625
H	3.461572	3.341268	0.817443
H	3.991683	-5.860949	3.488053
H	2.305840	-6.417164	3.241787
H	3.488041	-6.504010	1.907501

Table S121. Cartesian geometry of 3e-INT2 (14.3 kcal/mol) in Figure S146 in Angstrom [Å].

Atomtype	X Coordinates	Y Coordinates	Z Coordinates
C	2.724099	-4.017244	-2.064096
C	2.198187	-3.089694	-2.991016
C	1.763041	-3.596803	-4.241638
C	1.850585	-4.965291	-4.548814
C	2.379418	-5.866808	-3.608584
C	2.818012	-5.386692	-2.363675
Si	2.043714	-1.207912	-2.676226
Ge	1.456766	-0.071967	-0.566115
N	2.533882	-0.627648	1.012475
C	3.839473	-0.089851	1.259621
C	4.023150	1.068850	2.083252
C	5.328168	1.521689	2.365254
C	6.459465	0.894131	1.836257
C	6.285500	-0.207929	0.991665
C	5.005756	-0.709441	0.691214
C	2.861376	1.885646	2.647231
C	2.857476	1.918841	4.190812
C	4.916804	-1.899710	-0.257284
C	5.516844	-1.580332	-1.644096
Ni	-0.897565	0.004598	-0.285362
C	-2.582732	0.061497	-1.232760
N	-3.437831	-0.977458	-1.547061
C	-4.261946	-0.669547	-2.634326
C	-3.964020	0.607048	-2.998570
N	-2.962275	1.043752	-2.130990
C	-3.688569	-2.139656	-0.734266
C	-4.868984	-2.122726	0.055468
C	-5.146401	-3.257805	0.840875
C	-4.286368	-4.360102	0.832316
C	-3.143981	-4.360941	0.018192
C	-2.815371	-3.257559	-0.796934
C	-2.489053	2.401920	-2.124551
C	-3.014915	3.287851	-1.141637
C	-2.596773	4.630289	-1.197145
C	-1.713707	5.073032	-2.194516
C	-1.227220	4.182075	-3.158177
C	-1.597672	2.822068	-3.142622
C	-5.871826	-0.965537	0.019369
C	-6.185467	-0.383673	1.407523
C	-1.585890	-3.276045	-1.717367
C	-1.945238	-3.099548	-3.213602
C	-4.045373	2.807753	-0.112567
C	-4.139224	3.670392	1.158957

C	-1.096800	1.864102	-4.224769
C	0.411074	1.994716	-4.514721
P	-1.055319	0.227085	1.909486
C	-0.743325	2.004660	2.362348
C	-0.730262	2.958616	1.330413
C	-0.564464	4.324443	1.607960
C	-0.436082	4.756552	2.936324
C	-0.451208	3.812617	3.980325
C	-0.591548	2.445885	3.695718
C	-2.637337	-0.104590	2.872328
C	-3.089830	-1.440215	2.971147
C	-4.164079	-1.779845	3.806066
C	-4.834616	-0.786588	4.539035
C	-4.443422	0.553454	4.393816
C	-3.359017	0.893210	3.564651
C	0.150824	-0.719117	2.981386
Si	1.685481	-1.606288	2.207772
C	1.078249	-3.238914	1.379202
C	2.143098	-4.354663	1.411589
C	2.736098	-1.934998	3.797749
C	4.157694	-2.508367	3.647709
C	1.945358	-2.762315	4.839873
C	-7.167578	-1.405136	-0.698586
C	-0.746218	-4.556270	-1.580223
C	-5.449744	2.713559	-0.757469
C	-1.901070	2.066548	-5.530493
C	2.224572	1.712184	-1.046332
C	1.365750	2.808312	-1.286090
C	1.854257	4.079166	-1.605001
C	3.245065	4.298090	-1.702093
C	4.126232	3.214597	-1.500002
C	3.609974	1.945767	-1.182948
C	2.878981	3.330780	2.103063
C	5.595061	-3.170469	0.297977
C	-0.256085	-3.761105	1.945008
H	-0.372325	0.089664	-1.660060
H	0.911347	-2.970758	0.315196
H	-4.984778	-1.384351	-3.023578
H	-4.377930	1.251963	-3.771472
H	-3.071298	1.944997	3.475356
H	-1.271954	0.833906	-3.864423
H	-0.438768	-1.491434	3.504392
H	0.523941	-0.041923	3.771456
H	3.771968	2.404654	4.580215
H	2.808868	0.907418	4.630414
H	1.990815	2.495400	4.559218
H	2.258942	-4.767315	2.431086
H	3.135302	-4.005343	1.084087
H	1.850284	-5.192018	0.747483
H	-1.402000	6.125343	-2.218116
H	7.165480	-0.699352	0.556901
H	1.923108	1.418801	2.305928
H	2.852025	-0.914376	4.212064
H	-0.962336	-2.399993	-1.427086
H	-0.883698	2.619517	0.302656
H	7.465617	1.265701	2.070815
H	-0.608081	1.723970	4.521244
H	5.457830	2.401812	3.007877
H	4.326642	1.135088	-1.023560
H	-6.046653	-3.271554	1.466532

H	-2.601428	-2.232502	2.394871
H	-2.966411	5.343208	-0.453055
H	3.841232	-2.107787	-0.378604
H	5.210870	3.340888	-1.577121
H	-3.738427	1.791921	0.201467
H	-5.433203	-0.142913	-0.566334
H	0.282950	2.660316	-1.250552
H	-0.534444	4.541438	-3.926702
H	-4.509317	-5.236057	1.455375
H	-4.980260	1.346587	4.930681
H	-1.028424	-3.205486	-3.820219
H	-2.378003	-2.113938	-3.440043
H	-2.660688	-3.881269	-3.534276
H	-0.477303	-4.781816	1.576993
H	-1.097769	-3.122285	1.632621
H	-0.254564	-3.817435	3.049968
H	-2.500822	-5.244292	0.022266
H	5.120750	-0.640053	-2.059754
H	5.285012	-2.391638	-2.358974
H	6.617735	-1.485639	-1.583156
H	-5.672453	-1.051873	5.196355
H	-4.484340	-2.827277	3.867767
H	2.013075	3.891109	2.491379
H	2.835246	3.346694	1.004104
H	3.796248	3.863152	2.418885
H	-0.342786	4.142438	5.021560
H	0.661501	2.980097	-4.950147
H	0.716682	1.221748	-5.242681
H	1.013220	1.861055	-3.603283
H	-0.310825	5.823271	3.161720
H	-0.545202	5.040938	0.779180
H	-6.613036	-1.138830	2.090748
H	-6.923219	0.435334	1.313981
H	-5.277870	0.021450	1.879242
O	3.633012	5.573665	-2.001860
H	-1.329503	-5.442206	-1.896421
H	-0.389124	-4.726012	-0.553741
H	0.135789	-4.494682	-2.231232
H	-3.156849	3.818322	1.633944
H	-4.802046	3.170685	1.888425
H	-4.576590	4.664428	0.945867
H	-2.987281	1.939779	-5.377862
H	-1.576277	1.341119	-6.299929
H	-1.735967	3.085432	-5.929956
H	4.663440	-2.529708	4.634744
H	4.781737	-1.905626	2.968915
H	4.140304	-3.548130	3.275182
H	6.688424	-3.025242	0.385668
H	5.425524	-4.024012	-0.385613
H	5.217052	-3.442736	1.294973
H	1.174392	4.918405	-1.787284
H	1.775314	-3.798576	4.490855
H	0.959507	-2.326813	5.083892
H	2.515205	-2.832920	5.788492
H	-6.961103	-1.803890	-1.708524
H	-7.862354	-0.549926	-0.800122
H	-7.685207	-2.198051	-0.126169
H	-5.786504	3.716094	-1.083086
H	-6.180042	2.326624	-0.023802
H	-5.466004	2.046220	-1.634017

H	3.249810	-0.569088	-3.307226
H	0.894873	-0.809503	-3.564375
H	1.339312	-2.911848	-4.989474
H	1.500321	-5.330149	-5.523690
H	2.444265	-6.937094	-3.844500
H	3.224421	-6.079281	-1.614758
H	3.047264	-3.676947	-1.078579
C	5.018424	5.859832	-2.093024
H	5.103375	6.935673	-2.324173
H	5.546897	5.651271	-1.138651
H	5.509503	5.277031	-2.901060

Table S122. Cartesian geometry of 3f-INT2 (14.1 kcal/mol) in Figure S146 in Angstrom [Å].

Atomtype	X Coordinates	Y Coordinates	Z Coordinates
C	2.326033	-4.189340	-2.312395
C	1.901517	-3.151539	-3.172061
C	1.438644	-3.523622	-4.459663
C	1.401815	-4.868393	-4.865903
C	1.830295	-5.881592	-3.990526
C	2.295094	-5.536309	-2.710904
Si	1.913458	-1.291145	-2.718167
Ge	1.410031	-0.254301	-0.537209
N	2.400531	-1.022171	1.009715
C	3.746528	-0.627685	1.305392
C	4.025349	0.451086	2.207025
C	5.362656	0.757696	2.531453
C	6.438056	0.062246	1.971682
C	6.174463	-0.959961	1.053236
C	4.858016	-1.316686	0.708170
C	2.937121	1.334255	2.815966
C	2.908841	1.255244	4.357642
C	4.671875	-2.429273	-0.317306
C	5.310324	-2.072843	-1.677552
Ni	-0.932348	0.030605	-0.273691
C	-2.583934	0.320231	-1.234547
N	-3.528754	-0.605447	-1.635314
C	-4.298732	-0.141253	-2.706707
C	-3.873010	1.123453	-2.972197
N	-2.850498	1.397096	-2.062828
C	-3.904609	-1.793217	-0.912795
C	-5.093155	-1.719540	-0.138737
C	-5.492862	-2.876045	0.557662
C	-4.740247	-4.051863	0.481415
C	-3.586296	-4.103842	-0.314571
C	-3.139790	-2.982301	-1.044483
C	-2.250755	2.699160	-1.949229
C	-2.707480	3.556193	-0.907849
C	-2.164694	4.853478	-0.858297
C	-1.229022	5.283294	-1.812468
C	-0.811238	4.423278	-2.834723
C	-1.305686	3.106133	-2.923250
C	-5.978266	-0.470120	-0.099569
C	-6.257987	0.036284	1.324945
C	-1.901287	-3.054068	-1.949911
C	-2.217390	-2.741626	-3.433582
C	-3.797577	3.101974	0.070128
C	-3.829935	3.869374	1.404158
C	-0.875526	2.185818	-4.066674
C	0.643764	2.190506	-4.325079
P	-1.105916	0.100606	1.928960

C	-0.634924	1.801527	2.519865
C	-0.507100	2.823207	1.563396
C	-0.219682	4.142574	1.946111
C	-0.082058	4.461407	3.305207
C	-0.208930	3.448577	4.274321
C	-0.470942	2.126299	3.884827
C	-2.729717	-0.149017	2.846402
C	-3.309546	-1.438232	2.832674
C	-4.426062	-1.737346	3.626651
C	-5.012084	-0.744842	4.429744
C	-4.492509	0.558510	4.396309
C	-3.365615	0.856137	3.608666
C	-0.015478	-1.033452	2.940693
Si	1.447251	-1.999545	2.122272
C	0.710030	-3.509965	1.175057
C	1.671589	-4.715818	1.136427
C	2.432450	-2.535148	3.698548
C	3.800677	-3.221001	3.527046
C	1.549519	-3.360880	4.664663
C	-7.297534	-0.726189	-0.862007
C	-1.188524	-4.414804	-1.893709
C	-5.192340	3.195800	-0.595261
C	-1.629959	2.559130	-5.364388
C	2.344739	1.473330	-0.877419
C	1.601708	2.659723	-1.037849
C	2.202887	3.905950	-1.250546
C	3.618925	4.029597	-1.322613
C	4.377304	2.829037	-1.200451
C	3.749543	1.596212	-0.989174
C	3.103305	2.806500	2.380352
C	5.229880	-3.789262	0.154191
C	-0.673824	-3.950653	1.689307
H	-0.369184	0.169766	-1.629106
H	0.585120	-3.153292	0.131610
H	-5.079050	-0.752593	-3.156553
H	-4.208694	1.859505	-3.700348
H	-2.977141	1.878945	3.606829
H	-1.155389	1.153995	-3.786096
H	-0.684680	-1.784315	3.394770
H	0.402344	-0.455612	3.785603
H	3.859962	1.617686	4.791300
H	2.753038	0.224331	4.720248
H	2.096735	1.886647	4.758994
H	1.733687	-5.210002	2.123901
H	2.696036	-4.433774	0.845694
H	1.316954	-5.473653	0.409795
H	-0.821459	6.301210	-1.755262
H	7.010985	-1.502888	0.594557
H	1.964654	0.986915	2.430379
H	2.629549	-1.561759	4.188886
H	-1.203180	-2.263238	-1.592424
H	-0.664535	2.576246	0.510516
H	7.470931	0.320411	2.240029
H	-0.572725	1.349810	4.652749
H	5.564634	1.576542	3.233679
H	4.390958	0.715460	-0.891022
H	-6.402643	-2.847925	1.168890
H	-2.888760	-2.225744	2.199155
H	-2.478446	5.541262	-0.066389
H	3.583025	-2.532042	-0.455480

H	5.469539	2.848632	-1.259935
H	-3.596211	2.039819	0.306652
H	-5.451083	0.345986	-0.617408
H	0.508944	2.613962	-1.024538
H	-0.074728	4.770239	-3.567517
H	-5.057092	-4.944268	1.036796
H	-4.961104	1.355054	4.989160
H	-1.304552	-2.891582	-4.036781
H	-2.552169	-1.706170	-3.594192
H	-2.997525	-3.428171	-3.815558
H	-0.979316	-4.917347	1.243491
H	-1.451264	-3.217575	1.420289
H	-0.692782	-4.089213	2.786904
H	-3.028279	-5.042032	-0.365262
H	5.001316	-1.075952	-2.030003
H	5.015842	-2.811510	-2.446154
H	6.414663	-2.078496	-1.605927
H	-5.883189	-0.979047	5.054869
H	-4.845726	-2.750586	3.600893
H	2.286946	3.417666	2.798386
H	3.083638	2.905741	1.285052
H	4.060503	3.223725	2.747013
H	-0.092827	3.688524	5.339066
H	0.996185	3.176211	-4.682470
H	0.888616	1.446829	-5.104733
H	1.211428	1.933034	-3.417992
H	0.137608	5.491716	3.613035
H	-0.112275	4.912233	1.173529
H	-6.768270	-0.721602	1.945468
H	-6.911152	0.928298	1.285744
H	-5.323393	0.314631	1.834374
N	4.230832	5.262741	-1.507953
H	-1.846642	-5.217233	-2.278581
H	-0.867901	-4.688296	-0.877636
H	-0.292680	-4.392488	-2.528205
H	-2.846335	3.883408	1.898949
H	-4.550641	3.382288	2.085676
H	-4.165387	4.914912	1.265346
H	-2.726230	2.527492	-5.235976
H	-1.360327	1.863250	-6.180965
H	-1.360060	3.583468	-5.685314
H	4.281505	-3.359595	4.517112
H	4.489510	-2.628110	2.904453
H	3.700261	-4.225238	3.077583
H	6.331579	-3.750140	0.248937
H	4.986562	-4.578388	-0.582536
H	4.824741	-4.089347	1.132466
H	1.554137	4.777916	-1.362356
H	1.294730	-4.348683	4.236213
H	0.601714	-2.856404	4.925945
H	2.090508	-3.552311	5.613536
H	-7.112567	-1.066731	-1.897118
H	-7.904498	0.198019	-0.905482
H	-7.899420	-1.505194	-0.356806
H	-5.424255	4.248515	-0.845428
H	-5.970120	2.827380	0.098005
H	-5.256877	2.602092	-1.521181
H	3.178367	-0.720903	-3.298172
H	0.814759	-0.727251	-3.580381
H	1.091329	-2.749319	-5.158131

H	1.032626	-5.127031	-5.867440
H	1.797444	-6.933387	-4.303694
H	2.624734	-6.316559	-2.012094
H	2.666808	-3.953266	-1.302572
C	5.679880	5.358157	-1.523902
H	5.982487	6.409781	-1.657461
H	6.132650	4.990214	-0.577988
H	6.126022	4.770513	-2.354177
C	3.420050	6.461383	-1.604882
H	4.068872	7.341246	-1.746478
H	2.717126	6.419142	-2.464884
H	2.809800	6.631112	-0.690397

Table S123. Cartesian geometry of 3g-INT2 (20.5 kcal/mol) in Figure S146 in Angstrom [Å].

Atomtype	X Coordinates	Y Coordinates	Z Coordinates
C	3.746721	3.669751	0.144413
C	2.967836	3.604030	1.320507
C	2.670330	4.828449	1.971212
C	3.131180	6.057199	1.469099
C	3.905448	6.093914	0.296033
C	4.213276	4.893492	-0.364567
Si	2.259891	1.995202	2.084402
Ge	1.483332	-0.070145	0.972492
N	2.757175	-0.780028	-0.389881
C	3.889466	-1.580742	-0.024207
C	3.818719	-3.012632	-0.039270
C	4.971016	-3.771560	0.249774
C	6.187705	-3.169303	0.582949
C	6.255299	-1.772916	0.634988
C	5.139223	-0.968981	0.338607
C	2.522771	-3.778806	-0.299240
C	2.617898	-4.745809	-1.498156
C	5.323762	0.543812	0.399139
C	5.761424	1.036200	1.795737
Ni	-0.786108	0.120938	0.334554
C	-2.458842	0.979622	0.795603
N	-3.002680	2.150205	0.303808
C	-3.918722	2.719353	1.194540
C	-3.998651	1.877410	2.260864
N	-3.128205	0.819935	1.995354
C	-2.891844	2.623962	-1.051570
C	-3.993739	2.362437	-1.908406
C	-3.917479	2.832768	-3.233187
C	-2.794545	3.538091	-3.676702
C	-1.734847	3.808942	-2.798203
C	-1.753687	3.369211	-1.457902
C	-3.024081	-0.332362	2.847399
C	-3.715915	-1.515530	2.461105
C	-3.643931	-2.614343	3.336850
C	-2.933812	-2.532173	4.545946
C	-2.281834	-1.347604	4.907056
C	-2.305575	-0.219659	4.061846
C	-5.270715	1.670251	-1.422489
C	-5.665862	0.449897	-2.269954
C	-0.606638	3.694651	-0.488675
C	-1.064415	4.509835	0.746188
C	-4.550151	-1.558004	1.174801
C	-4.832821	-2.970948	0.633944
C	-1.627504	1.085794	4.480484
C	-0.178720	0.901951	4.972357

P	-0.877107	-1.311236	-1.349907
C	-1.018464	-3.044686	-0.684554
C	-1.355522	-3.212761	0.669144
C	-1.554992	-4.492658	1.211353
C	-1.438422	-5.626174	0.393211
C	-1.104686	-5.471507	-0.965375
C	-0.888748	-4.192022	-1.498688
C	-2.267181	-1.325590	-2.617407
C	-2.340330	-0.260440	-3.543991
C	-3.254320	-0.289476	-4.607190
C	-4.139814	-1.370430	-4.752327
C	-4.126785	-2.400138	-3.798669
C	-3.203722	-2.376479	-2.737475
C	0.587946	-1.405090	-2.507338
Si	2.271881	-0.558198	-2.069993
C	2.098194	1.327020	-2.451635
C	3.422559	1.964818	-2.920221
C	3.440872	-1.454300	-3.321891
C	4.958048	-1.202711	-3.238986
C	2.957119	-1.275811	-4.781180
C	-6.431527	2.687151	-1.345394
C	0.550321	4.457517	-1.154760
C	-5.897615	-0.822285	1.375419
C	-2.475382	1.796697	5.560842
N	1.666391	-1.197875	2.478936
C	2.099264	-4.562582	0.959845
C	6.337327	1.061147	-0.645160
C	0.971551	1.656991	-3.449567
H	-0.368089	0.758978	1.596576
H	1.833383	1.791199	-1.478809
H	-4.428427	3.654528	0.969262
H	-4.594950	1.917637	3.170828
H	-3.210528	-3.195944	-2.012212
H	-1.582076	1.740394	3.591672
H	0.252500	-0.977410	-3.467277
H	0.802061	-2.469020	-2.716804
H	3.374736	-5.532307	-1.318432
H	2.896337	-4.228163	-2.432537
H	1.648048	-5.249022	-1.659842
H	3.676341	1.648895	-3.949104
H	4.275325	1.698039	-2.275956
H	3.342454	3.070016	-2.924606
H	-2.893695	-3.402784	5.213355
H	7.203626	-1.288879	0.901772
H	1.729641	-3.041953	-0.504079
H	3.284590	-2.517678	-3.055730
H	-0.228525	2.717454	-0.110637
H	-1.489329	-2.325590	1.293096
H	7.071535	-3.780982	0.806330
H	-0.643776	-4.089310	-2.562737
H	4.906705	-4.867022	0.225806
H	-4.749183	2.639851	-3.921078
H	-1.676618	0.604569	-3.444518
H	-4.151163	-3.548828	3.075716
H	4.337349	0.976830	0.166235
H	-3.972805	-1.018253	0.399716
H	-5.095712	1.292174	-0.403300
H	-1.734292	-1.297187	5.855164
H	-2.743429	3.893638	-4.714090
H	-4.836023	-3.234537	-3.877500

H	-0.179440	4.777095	1.350675
H	-1.752743	3.953720	1.399415
H	-1.559277	5.447886	0.428483
H	1.016091	2.716113	-3.769808
H	-0.021532	1.504422	-2.996500
H	1.031585	1.046797	-4.370697
H	-0.878625	4.375475	-3.172264
H	5.110619	0.651212	2.596618
H	5.732021	2.140844	1.836917
H	6.796551	0.715829	2.019778
H	-4.851911	-1.396437	-5.586970
H	-3.279668	0.547970	-5.315457
H	1.121250	-5.046651	0.803161
H	2.018186	-3.887848	1.824223
H	2.837181	-5.349569	1.206001
H	-1.006017	-6.353278	-1.611426
H	-0.126846	0.260024	5.871477
H	0.248403	1.885150	5.240729
H	0.460325	0.465584	4.189174
H	-1.596465	-6.629054	0.809934
H	-1.812259	-4.590021	2.272479
H	-5.848121	0.715604	-3.326425
H	-6.597148	0.002797	-1.874183
H	-4.877613	-0.317262	-2.246791
H	0.210095	5.448370	-1.511779
H	0.987435	3.914952	-2.005977
H	1.352879	4.629109	-0.424933
H	-3.911887	-3.562720	0.515422
H	-5.318210	-2.890884	-0.355635
H	-5.523547	-3.529331	1.294208
H	-3.512656	1.973279	5.223318
H	-2.027770	2.773489	5.823951
H	-2.523822	1.183872	6.481157
H	5.492289	-1.876345	-3.939890
H	5.357949	-1.390560	-2.229688
H	5.216179	-0.168910	-3.530609
H	7.356417	0.697237	-0.414609
H	6.367245	2.167224	-0.635387
H	6.091429	0.727661	-1.664527
H	3.064301	-0.227905	-5.119744
H	1.901203	-1.566740	-4.928069
H	3.565911	-1.898416	-5.467746
H	-6.171309	3.558034	-0.716515
H	-7.334920	2.210894	-0.919397
H	-6.689459	3.067136	-2.352054
H	-6.502407	-1.338983	2.144657
H	-6.472247	-0.818208	0.431352
H	-5.763157	0.224259	1.692220
H	3.145501	1.653679	3.251341
H	0.975268	2.473966	2.707928
H	2.057616	4.825906	2.883887
H	2.880938	6.990041	1.992037
H	4.262089	7.053255	-0.101315
H	4.809182	4.905605	-1.286813
H	3.978030	2.755769	-0.404831
C	2.930597	-1.472752	3.136110
C	0.542665	-1.887713	3.072840
H	3.205028	-2.550943	3.098344
H	3.757266	-0.927147	2.656841
H	2.902529	-1.175983	4.211766

H	0.522578	-2.981437	2.861748
H	0.541930	-1.778295	4.179785
H	-0.403190	-1.455771	2.711631

Table S124. Cartesian geometry of 3a-INT3 (17.0 kcal/mol) in Figure S146 in Angstrom [Å].

Atomtype	X Coordinates	Y Coordinates	Z Coordinates
C	4.347230	-2.050524	-1.721924
C	4.052648	-1.412126	-0.466679
C	5.080079	-0.620860	0.153643
C	6.301373	-0.420492	-0.520349
C	6.556096	-0.989793	-1.771746
C	5.586131	-1.819565	-2.347958
N	2.727819	-1.488400	0.068921
Ge	1.521462	-0.314821	-0.922308
C	2.362889	1.506904	-0.774595
C	1.819997	2.533987	0.022297
C	2.443619	3.781082	0.132999
C	3.620914	4.042608	-0.607154
C	4.165105	3.031772	-1.435922
C	3.542352	1.778704	-1.505609
C	4.960885	-0.039639	1.564615
C	6.043274	-0.641038	2.494334
C	3.385462	-3.047907	-2.369807
C	3.141355	-2.798265	-3.871197
Ni	-0.699506	0.101671	-0.301501
Si	-1.048517	-1.153649	-2.177775
C	-1.423487	-3.002068	-1.843255
C	-2.506766	-3.446379	-1.047939
C	-2.820963	-4.809425	-0.931924
C	-2.061163	-5.769021	-1.624055
C	-0.982874	-5.351731	-2.421455
C	-0.662870	-3.986768	-2.518920
P	-0.172298	0.034122	1.977543
C	-0.043452	1.525571	3.098181
C	-1.160818	1.996663	3.820001
C	-1.050799	3.090381	4.692992
C	0.176796	3.754824	4.844853
C	1.291216	3.315715	4.110428
C	1.185270	2.211060	3.250195
C	-1.456239	-1.011880	2.816150
C	-2.303823	-1.779386	2.002914
C	-3.151857	-2.747543	2.561989
C	-3.174805	-2.939335	3.950737
C	-2.346628	-2.156726	4.778908
C	-1.486381	-1.202552	4.216586
C	1.346400	-0.897209	2.557518
Si	2.082342	-2.316937	1.485571
C	0.695637	-3.572372	1.074465
C	1.161802	-4.752382	0.201902
C	3.483644	-3.088929	2.556185
C	4.506223	-3.941695	1.775698
C	2.970839	-3.867012	3.787908
C	-2.284612	1.175564	-0.789057
N	-3.650814	0.941058	-0.940555
C	-4.282840	1.954769	-1.665462
C	-3.329058	2.864258	-1.980645
N	-2.128278	2.393708	-1.446938
C	-4.471922	-0.130593	-0.423361
C	-4.801568	-0.129988	0.963220
C	-5.660292	-1.145985	1.424610

C	-6.197597	-2.103239	0.554023
C	-5.924829	-2.033274	-0.813762
C	-5.083313	-1.031906	-1.341872
C	-0.985412	3.279175	-1.577886
C	-0.943278	4.404397	-0.717142
C	-0.025505	5.428597	-1.021343
C	0.835208	5.323245	-2.119740
C	0.804017	4.181027	-2.930421
C	-0.114727	3.142200	-2.690696
C	-4.294214	0.962359	1.914649
C	-4.546898	0.643145	3.398834
C	-4.997435	-0.891800	-2.865157
C	-6.338405	-0.345039	-3.413336
C	-1.866636	4.534952	0.493460
C	-1.083755	4.893360	1.769790
C	-0.192617	1.921439	-3.601376
C	1.125857	1.589004	-4.319533
C	-4.915549	2.346736	1.604511
C	-4.637120	-2.201014	-3.596004
C	-2.992453	5.561270	0.242248
C	-1.336881	2.024954	-4.632935
C	5.026194	1.498563	1.635764
C	3.892979	-4.489169	-2.139278
C	-0.041302	-4.126205	2.315274
H	-0.034896	-2.979519	0.484660
H	-5.352861	1.936146	-1.857430
H	-3.375983	3.803887	-2.528149
H	-0.831665	-0.609680	4.867130
H	-0.424112	1.079285	-2.925427
H	1.154265	-1.217277	3.598060
H	2.165666	-0.160190	2.606717
H	7.041280	-0.232631	2.247853
H	6.104250	-1.738641	2.404147
H	5.832370	-0.386564	3.550798
H	1.938713	-5.349661	0.716440
H	1.570416	-4.425403	-0.764688
H	0.314061	-5.427421	-0.015390
H	1.556323	6.123302	-2.328171
H	5.800609	-2.310322	-3.305659
H	3.974864	-0.331810	1.951536
H	4.019109	-2.205452	2.948792
H	-4.207245	-0.160494	-3.101825
H	-2.119392	1.483471	3.723892
H	7.510871	-0.809505	-2.282621
H	2.076592	1.896311	2.696368
H	7.072857	0.200259	-0.046778
H	3.990807	0.997466	-2.132291
H	-5.912731	-1.203676	2.486128
H	-2.277444	-1.619775	0.920454
H	0.024363	6.316283	-0.380072
H	2.415377	-2.949638	-1.855881
H	5.079665	3.232294	-2.006549
H	-2.337372	3.550723	0.665706
H	-3.201172	1.038473	1.752624
H	0.880473	2.366103	0.558398
H	1.505119	4.098528	-3.766765
H	-6.853407	-2.891506	0.945783
H	-2.365063	-2.299169	5.867066
H	-6.259224	-0.156049	-4.500388
H	-6.644511	0.596351	-2.922403

H	-7.152492	-1.076905	-3.253766
H	-1.005916	-4.574776	2.011403
H	-0.262373	-3.363339	3.079338
H	0.547931	-4.923126	2.801829
H	-6.388247	-2.754301	-1.496524
H	2.803822	-1.763243	-4.056652
H	2.361593	-3.486534	-4.249729
H	4.051985	-2.978316	-4.473332
H	-3.833754	-3.697560	4.392695
H	-3.789901	-3.347534	1.904644
H	4.995841	1.829409	2.692189
H	4.185120	1.966249	1.107525
H	5.962159	1.886146	1.192697
H	2.255521	3.830930	4.207199
H	1.332952	2.305399	-5.137428
H	1.055993	0.581569	-4.766673
H	1.981166	1.595861	-3.625550
H	0.264170	4.611848	5.524406
H	-1.933116	3.424066	5.254840
H	-5.630185	0.609109	3.619531
H	-4.128658	1.444399	4.033294
H	-4.093566	-0.311592	3.706644
C	4.231682	5.340031	-0.537833
H	-5.428486	-2.965069	-3.480982
H	-3.692393	-2.628153	-3.227764
H	-4.527662	-2.001841	-4.678138
H	-0.279042	4.166127	1.965232
H	-1.756757	4.887532	2.644569
H	-0.632894	5.900985	1.705737
H	-2.323237	2.140548	-4.154750
H	-1.368916	1.106522	-5.247769
H	-1.171429	2.887941	-5.306288
H	5.340543	-4.249112	2.438873
H	4.942056	-3.400701	0.918547
H	4.043744	-4.868936	1.388888
H	4.865919	-4.645301	-2.643092
H	3.173907	-5.227575	-2.543452
H	4.029957	-4.696482	-1.064341
H	2.013534	4.562088	0.767785
H	2.554219	-4.848299	3.497876
H	2.185017	-3.325443	4.347544
H	3.804646	-4.065701	4.491216
H	-4.701031	2.699494	0.585057
H	-4.510690	3.100675	2.305173
H	-6.013793	2.313495	1.735914
H	-2.569377	6.567948	0.063514
H	-3.661404	5.621975	1.121338
H	-3.606813	5.288712	-0.633496
H	1.993345	-0.648872	-2.361105
H	-2.272682	-0.629462	-2.868600
H	-0.062080	-1.237655	-3.308980
H	0.187424	-3.681834	-3.143049
H	-0.378536	-6.092998	-2.960898
H	-2.308167	-6.835522	-1.541503
H	-3.673268	-5.121532	-0.313284
H	-3.150517	-2.717621	-0.544778
N	4.705837	6.410887	-0.489908

Table S125. Cartesian geometry of 3b-INT3 (27.1 kcal/mol) in Figure S146 in Angstrom [Å].

Atomtype	X Coordinates	Y Coordinates	Z Coordinates
C	3.033676	1.577046	3.337528
C	2.054999	0.561073	3.250363
C	2.035812	-0.432751	4.255633
C	2.920479	-0.377177	5.342932
C	3.862139	0.661184	5.440940
C	3.924908	1.627024	4.424063
P	0.706500	0.588247	1.928484
C	0.670098	2.400342	1.550251
Si	-0.708550	3.370557	0.602491
C	-1.759153	4.415590	1.835735
C	-0.920285	5.288452	2.794438
Ni	1.089136	-0.275711	-0.235309
C	2.342828	-1.662042	-0.887630
N	3.687776	-1.782574	-1.233260
C	3.944879	-2.957695	-1.945211
C	2.763359	-3.610234	-2.068948
N	1.802685	-2.818481	-1.438808
C	4.815338	-0.953330	-0.873695
C	5.281991	-0.993041	0.469200
C	6.419688	-0.226076	0.782599
C	7.088351	0.516743	-0.200763
C	6.649773	0.481826	-1.526692
C	5.514114	-0.264065	-1.900559
C	0.474650	-3.375010	-1.308718
C	0.281193	-4.322988	-0.267271
C	-0.914490	-5.062955	-0.279793
C	-1.872280	-4.864502	-1.283232
C	-1.660249	-3.918661	-2.292734
C	-0.474714	-3.158787	-2.341121
C	4.615208	-1.893079	1.513533
C	4.937972	-3.384690	1.251788
C	5.162467	-0.388269	-3.386061
C	5.082239	0.963895	-4.120860
C	1.349836	-4.527247	0.810429
C	1.344103	-5.932906	1.436590
C	-0.210192	-2.171558	-3.481126
C	0.715336	-2.765223	-4.569578
Si	1.644199	1.012952	-1.991992
C	3.030692	2.161777	-1.325847
C	3.731692	1.919862	-0.124923
C	4.745411	2.784333	0.318171
C	5.090273	3.912678	-0.443764
C	4.398357	4.180053	-1.637098
C	3.370540	3.321861	-2.061374
Ge	-0.976217	0.552454	-0.934104
C	-2.360647	-0.864352	-0.522558
C	-2.200676	-1.900180	0.414313
C	-3.253915	-2.795881	0.682014
C	-4.457893	-2.711177	-0.034806
C	-4.607773	-1.711755	-1.005422
C	-3.576886	-0.784973	-1.233611
C	-0.708315	0.351724	3.125023
C	-1.186846	-0.942690	3.408729
C	-2.144536	-1.159466	4.412719
C	-2.640832	-0.076630	5.153938
C	-2.168856	1.219556	4.885929
C	-1.208228	1.431450	3.885485
N	-1.737213	2.215572	-0.261022

C	-3.106959	2.369948	-0.685036
C	-4.193041	2.015188	0.190212
C	-5.509587	1.996585	-0.311882
C	-5.799075	2.298831	-1.644116
C	-4.754492	2.687660	-2.487142
C	-3.420837	2.753999	-2.036834
C	-4.021427	1.670259	1.671579
C	-4.532569	0.261877	2.036266
C	-2.389388	3.304984	-3.025690
C	-2.768478	4.749630	-3.440044
C	0.370281	4.554308	-0.497942
C	1.642763	5.090183	0.201784
C	-0.377449	5.753506	-1.113471
C	-4.753859	2.691395	2.575758
C	-2.239569	2.452675	-4.305664
C	5.007032	-1.556532	2.957566
C	6.191978	-1.298151	-4.099226
C	1.251342	-3.447131	1.910183
C	-1.492739	-1.649825	-4.155259
C	-2.842132	5.288957	1.159252
H	0.703390	3.897299	-1.326963
H	4.950194	-3.219947	-2.265563
H	2.504548	-4.568330	-2.517207
H	1.295187	-1.239775	4.215889
H	0.316385	-1.311407	-3.028925
H	1.565686	2.520872	0.921228
H	0.884039	2.970503	2.472360
H	-5.849424	2.550723	2.510855
H	-4.540665	3.734974	2.301782
H	-4.461168	2.537808	3.631344
H	-0.500409	6.561625	-0.367982
H	-1.376305	5.497473	-1.489463
H	0.198413	6.183251	-1.957978
H	-2.804297	-5.441224	-1.263151
H	-4.978592	2.956502	-3.527003
H	-2.944535	1.697901	1.900176
H	-2.282572	3.662455	2.451734
H	4.168413	-0.860563	-3.466471
H	-0.793960	-1.801525	2.862647
H	-6.827899	2.235016	-2.019611
H	-0.842715	2.447835	3.717216
H	-6.328593	1.709931	0.358003
H	-3.737560	0.006243	-1.974218
H	6.799073	-0.210774	1.808786
H	3.099896	2.355844	2.572474
H	-1.109428	-5.798829	0.504714
H	-1.411135	3.325362	-2.515571
C	-5.924666	-1.596181	-1.744801
H	2.332903	-4.395042	0.324633
H	3.523150	-1.746701	1.416023
H	-1.248808	-2.008666	0.936585
H	-2.431988	-3.770383	-3.054106
H	7.967041	1.115795	0.070308
H	2.868947	-1.149573	6.121258
H	5.903072	-1.452990	-5.155869
H	6.286765	-2.289360	-3.621307
H	7.194654	-0.830739	-4.085145
H	2.196056	5.759900	-0.484929
H	2.351643	4.300820	0.496499
H	1.396254	5.681950	1.102722

H	7.199328	1.040092	-2.292970
H	-1.967283	1.411143	-4.070491
H	-1.450283	2.875976	-4.955855
H	-3.180827	2.438580	-4.887340
H	4.547264	0.710257	6.296661
H	4.665691	2.435873	4.475046
H	-4.455764	0.104736	3.126062
H	-3.945256	-0.523201	1.550344
H	-5.590413	0.125597	1.745984
H	-2.546362	2.074511	5.461138
H	-1.960131	-2.429612	-4.786334
H	-1.244879	-0.799075	-4.813944
H	-2.239019	-1.306924	-3.422497
H	-3.395647	-0.239662	5.933537
H	-2.510016	-2.175579	4.598090
H	6.067201	-1.804190	3.157174
H	4.394130	-2.148269	3.657985
H	4.846359	-0.493625	3.190332
H	-5.272480	-3.415636	0.163567
H	6.068399	1.462475	-4.168131
H	4.379843	1.649065	-3.625226
H	4.742645	0.804083	-5.161057
H	1.216432	-2.433458	1.472713
H	2.126771	-3.495264	2.582813
H	0.345555	-3.602952	2.517908
H	1.719407	-3.006681	-4.186616
H	0.839806	-2.036043	-5.391883
H	0.271109	-3.686212	-4.993440
H	-3.520123	5.720542	1.923308
H	-3.462023	4.721932	0.443539
H	-2.395022	6.139075	0.617985
H	-3.634201	4.744880	-4.128397
H	-1.926303	5.240742	-3.962606
H	-3.050381	5.370158	-2.571381
C	-3.205793	-3.796017	1.813200
H	-0.427115	6.118961	2.256380
H	-0.130026	4.725028	3.326266
H	-1.570450	5.746856	3.566948
H	4.616236	-3.727608	0.256508
H	4.433904	-4.018826	2.004566
H	6.027701	-3.556939	1.337572
H	0.445753	-6.097679	2.060108
H	2.224608	-6.052272	2.094784
H	1.377550	-6.724629	0.665752
H	-1.319216	0.677369	-2.440918
H	2.206758	0.189869	-3.121004
H	0.769610	1.995704	-2.729152
H	2.822647	3.562256	-2.983233
H	4.647668	5.069745	-2.230332
H	5.887654	4.586875	-0.104804
H	5.274610	2.562678	1.252710
H	3.485694	1.035612	0.473530
F	-5.798199	-0.933384	-2.917133
F	-6.448152	-2.820158	-2.023009
F	-6.856368	-0.935450	-1.006409
F	-3.603367	-5.038241	1.427253
F	-1.970136	-3.926861	2.363765
F	-4.041639	-3.427353	2.823761

Table S126. Cartesian geometry of 3c-INT3 (17.5 kcal/mol) in Figure S146 in Angstrom [Å].

Atomtype	X Coordinates	Y Coordinates	Z Coordinates
C	-1.878811	-1.920078	2.105727
C	-1.172278	-0.976174	2.866133
C	-1.155019	-1.100772	4.274248
C	-1.828467	-2.162026	4.896312
C	-2.513186	-3.118025	4.120873
C	-2.536739	-2.994009	2.724509
P	-0.103690	0.240406	1.955730
C	1.560574	-0.374397	2.556827
Si	2.517924	-1.701864	1.542203
C	4.032255	-2.184954	2.629812
C	3.663852	-2.974378	3.905001
Ni	-0.640194	0.098335	-0.309709
C	-2.401319	0.831016	-0.824726
N	-3.701269	0.341927	-0.951761
C	-4.508851	1.183091	-1.722008
C	-3.738629	2.235236	-2.090996
N	-2.472534	2.022025	-1.543936
C	-4.311449	-0.831389	-0.368655
C	-4.634783	-0.814261	1.019408
C	-5.294018	-1.942152	1.544454
C	-5.645827	-3.029087	0.733350
C	-5.388881	-2.987710	-0.638692
C	-4.746542	-1.879642	-1.229765
C	-1.511876	3.094554	-1.725035
C	-1.671556	4.243239	-0.910607
C	-0.960285	5.404313	-1.269063
C	-0.103191	5.411035	-2.374765
C	0.073877	4.249316	-3.136536
C	-0.635464	3.070767	-2.841041
C	-4.332671	0.402622	1.904790
C	-5.201008	1.629252	1.530910
C	-4.693368	-1.807610	-2.759318
C	-4.101743	-3.064480	-3.428335
C	-2.597392	4.253444	0.305111
C	-3.903873	5.025004	0.018632
C	-0.485149	1.816199	-3.694602
C	-1.629762	1.653350	-4.717900
Si	-0.756012	-1.288647	-2.121992
C	-0.795502	-3.162854	-1.719363
C	-1.759119	-3.760512	-0.872390
C	-1.833707	-5.153053	-0.712657
C	-0.947335	-5.991079	-1.412267
C	0.016061	-5.421442	-2.260755
C	0.096375	-4.025323	-2.401500
Ge	1.621419	0.079272	-0.942408
C	2.132704	2.018659	-0.889026
C	1.433225	2.982705	-0.136833
C	1.862907	4.319038	-0.088677
C	2.984648	4.722145	-0.832056
C	3.670987	3.782233	-1.620103
C	3.252289	2.440371	-1.642159
C	-0.239252	1.781729	3.006691
C	-1.415646	2.073812	3.729399
C	-1.497121	3.206685	4.554313
C	-0.409101	4.088449	4.655533
C	0.757914	3.826886	3.917986
C	0.845443	2.685112	3.105619
N	3.009425	-0.846832	0.081901

C	4.301856	-0.579174	-0.469136
C	5.190197	0.391620	0.109433
C	6.362952	0.756205	-0.581344
C	6.705469	0.182098	-1.809303
C	5.880046	-0.815142	-2.343380
C	4.694077	-1.213783	-1.699451
C	4.979024	1.007149	1.494530
C	4.772431	2.533758	1.493986
C	3.903164	-2.377233	-2.299369
C	4.632917	-3.709147	-2.014357
C	1.355444	-3.185747	1.205729
C	0.747320	-3.816950	2.478495
C	1.991668	-4.298219	0.352989
C	6.152221	0.645732	2.437939
C	3.621296	-2.230874	-3.807672
C	-4.513674	0.124160	3.407516
C	-6.114726	-1.545942	-3.315318
C	-1.896633	4.824619	1.551191
C	0.871770	1.703972	-4.409410
C	5.173629	-2.897107	1.873374
H	0.524172	-2.740929	0.619230
H	-5.556942	0.957977	-1.903544
H	-3.957145	3.120452	-2.685574
H	-0.608763	-0.371056	4.884492
H	-0.553086	0.979015	-2.977629
H	1.439893	-0.663803	3.617043
H	2.237216	0.496792	2.546791
H	7.063739	1.209504	2.163816
H	6.402715	-0.427847	2.395601
H	5.903203	0.907048	3.484518
H	2.864633	-4.747024	0.864823
H	2.324672	-3.936422	-0.629757
H	1.261090	-5.107403	0.171145
H	0.454027	6.320798	-2.630754
H	6.169407	-1.305199	-3.281650
H	4.060805	0.566399	1.905972
H	4.420251	-1.208735	2.972284
H	-4.051922	-0.957476	-3.043702
H	-2.265835	1.391420	3.672787
H	7.618873	0.490843	-2.334380
H	1.770797	2.514999	2.544566
H	7.024392	1.513143	-0.140324
H	3.817138	1.712332	-2.238983
H	-5.531397	-1.985813	2.610144
H	-1.891235	-1.810852	1.016830
H	-1.072297	6.312244	-0.665021
H	2.930138	-2.412383	-1.783143
H	4.550138	4.088322	-2.202849
H	-2.864520	3.206122	0.532684
H	-3.273416	0.670121	1.722561
H	0.529773	2.693043	0.410751
H	0.776312	4.259771	-3.975594
H	-6.145870	-3.901050	1.174546
H	-1.811083	-2.251564	5.990166
H	-6.076739	-1.401091	-4.411380
H	-6.587604	-0.653509	-2.866997
H	-6.778687	-2.406415	-3.108664
H	-0.132112	-4.432316	2.210021
H	0.414060	-3.079456	3.226807
H	1.472777	-4.487371	2.971714

H	-5.711661	-3.818800	-1.275931
H	3.121554	-1.271660	-4.030700
H	2.961950	-3.050002	-4.153189
H	4.548621	-2.286378	-4.408857
H	-3.023762	-3.957695	4.609544
H	-3.064295	-3.729032	2.107602
H	4.687718	2.903510	2.534860
H	3.861359	2.819859	0.952307
H	5.624632	3.057011	1.021853
H	1.613489	4.512187	3.974239
H	0.937852	2.405523	-5.263229
H	0.992836	0.680671	-4.806950
H	1.710503	1.906827	-3.724722
H	-0.471406	4.975738	5.298201
H	-2.419226	3.399232	5.118481
H	-5.570826	-0.096410	3.646930
H	-4.246453	1.022503	3.991575
H	-3.890152	-0.712672	3.758049
H	3.320729	5.767101	-0.801069
H	-4.734564	-3.955525	-3.258220
H	-3.090125	-3.286491	-3.057570
H	-4.042344	-2.907446	-4.521303
H	-0.958070	4.288470	1.765830
H	-2.549095	4.723319	2.435667
H	-1.663138	5.899255	1.433983
H	-2.619709	1.602284	-4.235871
H	-1.488032	0.717580	-5.289609
H	-1.631534	2.499510	-5.431951
H	6.052718	-3.033225	2.536262
H	5.507969	-2.333889	0.985422
H	4.864871	-3.904579	1.536710
H	5.616985	-3.730832	-2.520510
H	4.038368	-4.568435	-2.380334
H	4.804252	-3.846563	-0.933073
H	1.308137	5.045778	0.516856
H	3.414799	-4.024675	3.669674
H	2.802116	-2.543213	4.448647
H	4.523457	-2.995985	4.605198
H	-5.059138	1.958893	0.491174
H	-4.940184	2.482187	2.184894
H	-6.273608	1.400707	1.679093
H	-3.685899	6.083406	-0.219972
H	-4.566991	5.002914	0.903979
H	-4.458394	4.590460	-0.831134
H	2.131350	-0.249384	-2.369297
H	-2.059493	-1.029451	-2.820801
H	0.219710	-1.244326	-3.264858
H	0.860577	-3.599611	-3.064975
H	0.717826	-6.065623	-2.807045
H	-1.007150	-7.081094	-1.295476
H	-2.598660	-5.585784	-0.053711
H	-2.500603	-3.136708	-0.362954

Table S127. Cartesian geometry of 3d-INT3 (17.8 kcal/mol) in Figure S146 in Angstrom [Å].

Atomtype	X Coordinates	Y Coordinates	Z Coordinates
C	4.513196	-1.714983	-1.702857
C	4.172572	-1.077378	-0.458793
C	5.143655	-0.209151	0.149292
C	6.351701	0.059124	-0.524532
C	6.649056	-0.514847	-1.764316

C	5.737164	-1.415229	-2.328970
N	2.856411	-1.235751	0.077754
Ge	1.575328	-0.147650	-0.927210
C	2.269973	1.726042	-0.813682
C	1.665220	2.728703	-0.029856
C	2.215339	4.014311	0.058239
C	3.370375	4.359390	-0.673961
C	3.956177	3.371596	-1.490010
C	3.421704	2.072813	-1.552589
C	4.979341	0.389492	1.548046
C	6.098558	-0.113277	2.492338
C	3.619544	-2.781003	-2.338380
C	3.362598	-2.566580	-3.843030
Ni	-0.676182	0.093517	-0.303031
Si	-0.922682	-1.222310	-2.153175
C	-1.162683	-3.091218	-1.798120
C	-2.197637	-3.604311	-0.980198
C	-2.416801	-4.984862	-0.852793
C	-1.607976	-5.894620	-1.556635
C	-0.575970	-5.409316	-2.376441
C	-0.350768	-4.026227	-2.484610
P	-0.142401	0.107083	1.970437
C	-0.122108	1.622465	3.066613
C	-1.267317	2.018359	3.789981
C	-1.236084	3.131334	4.644933
C	-0.061817	3.889742	4.777078
C	1.077662	3.525203	4.040229
C	1.051271	2.402664	3.197379
C	-1.342579	-1.015169	2.836352
C	-2.138674	-1.854294	2.042284
C	-2.912521	-2.870679	2.622951
C	-2.913960	-3.040169	4.014763
C	-2.138477	-2.187005	4.824069
C	-1.351075	-1.184267	4.239880
C	1.442148	-0.698637	2.560946
Si	2.270944	-2.081095	1.507771
C	0.971327	-3.432967	1.119265
C	1.509565	-4.584548	0.250894
C	3.720988	-2.741027	2.589226
C	4.797701	-3.534215	1.819364
C	3.263767	-3.532151	3.834584
C	-2.342808	1.027626	-0.804584
N	-3.687138	0.684538	-0.949238
C	-4.395745	1.629095	-1.696700
C	-3.515319	2.602662	-2.033243
N	-2.282215	2.239386	-1.489713
C	-4.422925	-0.434071	-0.404784
C	-4.751948	-0.425201	0.981948
C	-5.533271	-1.490650	1.468656
C	-5.995395	-2.507155	0.622376
C	-5.725522	-2.451635	-0.746673
C	-4.962396	-1.402257	-1.299971
C	-1.212281	3.208366	-1.638768
C	-1.254563	4.345374	-0.794078
C	-0.425288	5.435466	-1.120645
C	0.433858	5.381143	-2.223522
C	0.494817	4.226289	-3.013560
C	-0.336404	3.121598	-2.751967
C	-4.325316	0.723172	1.906727
C	-4.549404	0.419472	3.398758

C	-4.887380	-1.291816	-2.826301
C	-6.266368	-0.861979	-3.384254
C	-2.176568	4.417078	0.422542
C	-1.421037	4.873824	1.683712
C	-0.315282	1.881689	-3.639332
C	1.025661	1.646749	-4.354296
C	-5.049022	2.049721	1.567887
C	-4.428517	-2.585917	-3.528024
C	-3.394559	5.329287	0.161077
C	-1.465744	1.869225	-4.669091
C	4.930834	1.929205	1.585993
C	4.217514	-4.183830	-2.089085
C	0.281293	-4.027272	2.367857
H	0.199135	-2.894618	0.530405
H	-5.460871	1.522909	-1.887346
H	-3.634439	3.522549	-2.602899
H	-0.736038	-0.535753	4.875901
H	-0.475306	1.036636	-2.946521
H	1.280489	-1.010999	3.609078
H	2.204780	0.097910	2.587855
H	7.066031	0.360054	2.239597
H	6.238102	-1.205527	2.425482
H	5.866312	0.147230	3.543007
H	2.327742	-5.125771	0.763846
H	1.889708	-4.236840	-0.719867
H	0.707144	-5.315565	0.042743
H	1.080742	6.236056	-2.457018
H	5.986354	-1.907021	-3.277840
H	4.015925	0.034338	1.938796
H	4.195448	-1.817002	2.966709
H	-4.154927	-0.508398	-3.081003
H	-2.184105	1.431289	3.709870
H	7.592311	-0.281683	-2.275457
H	1.958370	2.151175	2.637118
H	7.078028	0.738687	-0.060126
H	3.923747	1.319266	-2.173430
H	-5.781538	-1.541180	2.531563
H	-2.129882	-1.709930	0.957351
H	-0.445254	6.334112	-0.493074
H	2.644426	-2.737196	-1.826860
H	4.858446	3.613901	-2.068814
H	-2.551941	3.397056	0.619952
H	-3.241327	0.877154	1.738801
H	0.739135	2.509265	0.512240
H	1.199487	4.184667	-3.849766
H	-6.590291	-3.332791	1.033992
H	-2.140390	-2.312000	5.914584
H	-6.201847	-0.691762	-4.475414
H	-6.644066	0.063814	-2.913876
H	-7.021807	-1.650571	-3.207109
H	-0.651472	-4.543932	2.072498
H	0.012072	-3.276227	3.128277
H	0.926881	-4.778453	2.856006
H	-6.131343	-3.222844	-1.411131
H	2.959304	-1.557778	-4.040948
H	2.629326	-3.308326	-4.213612
H	4.284187	-2.694873	-4.441967
H	-3.515075	-3.835537	4.473677
H	-3.510622	-3.525092	1.980165
H	4.864125	2.278361	2.635319

H	4.065782	2.322894	1.036308
H	5.842692	2.372661	1.144262
H	2.000436	4.114186	4.120501
H	1.168578	2.359439	-5.189294
H	1.041216	0.627056	-4.778300
H	1.877210	1.743456	-3.662421
H	-0.035825	4.761426	5.443180
H	-2.137793	3.405448	5.208349
H	-5.626528	0.309196	3.624996
H	-4.190517	1.264083	4.013049
H	-4.024715	-0.491481	3.725738
C	3.940328	5.757647	-0.581785
H	-5.157622	-3.406384	-3.391537
H	-3.452280	-2.929102	-3.154603
H	-4.338015	-2.404237	-4.615027
H	-0.545556	4.234286	1.880935
H	-2.081895	4.816692	2.565872
H	-1.073750	5.920285	1.597480
H	-2.457913	1.909220	-4.190569
H	-1.420426	0.940054	-5.266735
H	-1.374985	2.730414	-5.359024
H	5.650680	-3.778263	2.485425
H	5.196499	-2.975895	0.955304
H	4.396517	-4.494209	1.443991
H	5.198555	-4.284496	-2.591569
H	3.546846	-4.971970	-2.482649
H	4.367596	-4.367897	-1.011633
H	1.728465	4.768208	0.689978
H	2.917973	-4.545781	3.562738
H	2.440754	-3.037986	4.384668
H	4.108902	-3.658825	4.541273
H	-4.861433	2.394892	0.540546
H	-4.701661	2.847085	2.251107
H	-6.141710	1.936989	1.702048
H	-3.066793	6.365694	-0.046663
H	-4.057688	5.350646	1.046425
H	-3.990259	4.980290	-0.700233
H	2.059428	-0.488969	-2.360166
H	-2.185420	-0.807745	-2.851984
H	0.060269	-1.252827	-3.290382
H	0.465698	-3.667612	-3.125194
H	0.066586	-6.110710	-2.925105
H	-1.780815	-6.974970	-1.465289
H	-3.233722	-5.350492	-0.215932
H	-2.880249	-2.918458	-0.468099
H	4.841574	5.877633	-1.209317
H	3.195564	6.511663	-0.905926
H	4.215696	6.010122	0.460956

Table S128. Cartesian geometry of 3e-INT3 (18 kcal/mol) in Figure S146 in Angstrom [Å].

Atomtype	X Coordinates	Y Coordinates	Z Coordinates
C	4.208889	-2.318538	-1.679650
C	3.929890	-1.682395	-0.419322
C	4.993864	-0.966914	0.231292
C	6.238792	-0.836366	-0.415601
C	6.482697	-1.404034	-1.669918
C	5.472739	-2.160098	-2.277760
N	2.594805	-1.685335	0.091566
Ge	1.490213	-0.399498	-0.893040
C	2.422053	1.356084	-0.701911

C	1.939650	2.407217	0.107060
C	2.637455	3.610029	0.240214
C	3.829375	3.817995	-0.486364
C	4.319744	2.794204	-1.323214
C	3.618558	1.577184	-1.413867
C	4.882703	-0.397653	1.647419
C	5.907022	-1.074981	2.590360
C	3.197503	-3.239769	-2.363314
C	3.000167	-2.948736	-3.864009
Ni	-0.719961	0.131881	-0.296546
Si	-1.105488	-1.064777	-2.201543
C	-1.587251	-2.900028	-1.928247
C	-2.699125	-3.305842	-1.152051
C	-3.094239	-4.650971	-1.082383
C	-2.389152	-5.630722	-1.803638
C	-1.283666	-5.251867	-2.582810
C	-0.883176	-3.905658	-2.633540
P	-0.238460	-0.013146	1.982050
C	-0.042102	1.445264	3.137242
C	-1.138804	1.960121	3.860865
C	-0.977721	3.025584	4.760552
C	0.283021	3.617534	4.938495
C	1.378261	3.135521	4.202092
C	1.220538	2.059225	3.314495
C	-1.594792	-0.998210	2.781893
C	-2.475202	-1.695713	1.940948
C	-3.388090	-2.622624	2.466792
C	-3.444106	-2.841554	3.850540
C	-2.582723	-2.128170	4.706834
C	-1.657690	-1.216794	4.177193
C	1.212505	-1.042010	2.569872
Si	1.874201	-2.487751	1.483241
C	0.415028	-3.640409	1.026787
C	0.812914	-4.822446	0.124202
C	3.202226	-3.366088	2.565637
C	4.182023	-4.269229	1.787179
C	2.621888	-4.128818	3.776750
C	-2.235238	1.303851	-0.782597
N	-3.610244	1.147935	-0.960757
C	-4.173706	2.208213	-1.675701
C	-3.166587	3.070185	-1.956472
N	-2.002638	2.524879	-1.412601
C	-4.498319	0.116349	-0.474492
C	-4.852493	0.115262	0.906164
C	-5.776790	-0.855623	1.336852
C	-6.351462	-1.768394	0.442707
C	-6.049340	-1.695260	-0.918741
C	-5.143501	-0.735491	-1.416861
C	-0.811860	3.349402	-1.506646
C	-0.723393	4.451621	-0.620356
C	0.252707	5.430533	-0.887289
C	1.126519	5.299607	-1.971943
C	1.048031	4.178488	-2.807968
C	0.069263	3.187615	-2.607191
C	-4.299647	1.161526	1.883664
C	-4.592559	0.832739	3.358346
C	-5.026660	-0.574774	-2.936114
C	-6.326569	0.056110	-3.492860
C	-1.654338	4.601641	0.582138
C	-0.870872	4.897387	1.874066

C	-0.058624	1.990839	-3.543592
C	1.251605	1.603009	-4.248368
C	-4.837975	2.583300	1.588280
C	-4.729652	-1.889090	-3.685708
C	-2.728393	5.684087	0.340288
C	-1.179753	2.173686	-4.589418
C	5.037378	1.132664	1.738361
C	3.603738	-4.715234	-2.149699
C	-0.371036	-4.180509	2.242640
H	-0.267169	-2.983386	0.447014
H	-5.239669	2.251895	-1.885368
H	-3.152710	4.021119	-2.486011
H	-0.977686	-0.679367	4.849543
H	-0.343950	1.149378	-2.887650
H	0.987580	-1.364918	3.603033
H	2.072786	-0.355036	2.640053
H	6.933324	-0.726354	2.368558
H	5.902350	-2.173337	2.487981
H	5.691035	-0.819967	3.645817
H	1.543453	-5.484559	0.627784
H	1.253019	-4.494203	-0.827825
H	-0.074793	-5.433345	-0.121763
H	1.899341	6.058556	-2.144909
H	5.674106	-2.649822	-3.238951
H	3.873067	-0.634137	2.010355
H	3.785476	-2.524013	2.980629
H	-4.192968	0.114517	-3.148193
H	-2.122950	1.502310	3.745018
H	7.457769	-1.279666	-2.159061
H	2.097066	1.712633	2.756307
H	7.038235	-0.272603	0.082612
H	4.032999	0.783752	-2.049549
H	-6.051040	-0.914126	2.392903
H	-2.423206	-1.514232	0.862821
H	0.342238	6.297676	-0.222750
H	2.226426	-3.085387	-1.865833
H	5.247078	2.919322	-1.891575
H	-2.171968	3.636921	0.728823
H	-3.201724	1.179407	1.739016
H	0.986669	2.293427	0.634599
H	1.761074	4.071609	-3.631274
H	-7.057933	-2.523568	0.810990
H	-2.625897	-2.292128	5.791320
H	-6.220748	0.259125	-4.575171
H	-6.586697	1.004706	-2.989382
H	-7.182762	-0.631387	-3.357843
H	-1.357180	-4.560704	1.915200
H	-0.554983	-3.426156	3.024901
H	0.161438	-5.025504	2.713603
H	-6.540414	-2.378848	-1.620512
H	2.730696	-1.891524	-4.034944
H	2.187512	-3.580528	-4.270935
H	3.910187	-3.173918	-4.451969
H	-4.154168	-3.567662	4.266650
H	-4.050544	-3.169240	1.787376
H	4.996696	1.453971	2.797722
H	4.241909	1.654237	1.190168
H	6.008376	1.465676	1.326321
H	2.368159	3.595600	4.316772
H	1.508942	2.323571	-5.048560

H	1.135347	0.609712	-4.717130
H	2.093055	1.551298	-3.539632
H	0.410581	4.452335	5.639223
H	-1.845930	3.394057	5.322809
H	-5.679317	0.856766	3.563110
H	-4.138944	1.598388	4.012192
H	-4.198787	-0.151317	3.655873
O	4.416464	5.043872	-0.327672
H	-5.562471	-2.610975	-3.592370
H	-3.813530	-2.372461	-3.314996
H	-4.596991	-1.677875	-4.763004
H	-0.093907	4.137219	2.055551
H	-1.551798	4.893525	2.742755
H	-0.382998	5.889249	1.839774
H	-2.166266	2.332322	-4.124056
H	-1.250264	1.269498	-5.221973
H	-0.958844	3.038950	-5.243986
H	4.983987	-4.640742	2.457730
H	4.665383	-3.742204	0.946913
H	3.667901	-5.158294	1.376192
H	4.573451	-4.927866	-2.639135
H	2.844583	-5.397282	-2.579034
H	3.706648	-4.949056	-1.076362
H	2.262069	4.419533	0.874354
H	2.151708	-5.079120	3.465812
H	1.860428	-3.548172	4.330799
H	3.429520	-4.387579	4.491258
H	-4.586413	2.940343	0.578849
H	-4.403916	3.301564	2.308648
H	-5.938566	2.609043	1.701132
H	-2.256116	6.672436	0.182841
H	-3.401707	5.761582	1.214806
H	-3.347221	5.456277	-0.545040
H	1.952675	-0.759913	-2.328895
H	-2.296409	-0.464560	-2.890611
H	-0.118592	-1.171977	-3.330856
H	-0.013058	-3.630720	-3.244019
H	-0.721236	-6.009437	-3.144649
H	-2.699253	-6.682894	-1.757239
H	-3.966570	-4.932148	-0.476856
H	-3.301660	-2.557027	-0.627514
C	5.630417	5.309414	-1.009049
H	5.930964	6.334195	-0.730553
H	6.434276	4.602876	-0.712070
H	5.506805	5.256293	-2.111843

Table S129. Cartesian geometry of 3f-INT3 (19.1 kcal/mol) in Figure S146 in Angstrom [Å].

Atomtype	X Coordinates	Y Coordinates	Z Coordinates
C	3.754914	-2.979120	-1.686604
C	3.577163	-2.301619	-0.429556
C	4.736016	-1.741122	0.210768
C	5.982508	-1.792027	-0.444155
C	6.135278	-2.396396	-1.695767
C	5.024528	-3.005785	-2.292607
N	2.258237	-2.110448	0.086745
Ge	1.348461	-0.681948	-0.905165
C	2.514729	0.922551	-0.730674
C	2.193216	2.037412	0.069755
C	3.064384	3.117972	0.224129
C	4.303861	3.164449	-0.470434

C	4.607086	2.068640	-1.324083
C	3.737282	0.972723	-1.432322
C	4.716170	-1.156353	1.624758
C	5.642613	-1.967099	2.563567
C	2.617295	-3.750181	-2.357687
C	2.454701	-3.444971	-3.859769
Ni	-0.760584	0.161399	-0.297898
Si	-1.326364	-0.996454	-2.182230
C	-2.076828	-2.735279	-1.882596
C	-3.230810	-2.960708	-1.094605
C	-3.823342	-4.230096	-1.006070
C	-3.279337	-5.312833	-1.719531
C	-2.135570	-5.113298	-2.509928
C	-1.537582	-3.843318	-2.579700
P	-0.283934	-0.027101	1.978136
C	0.133904	1.399329	3.114026
C	-0.869846	2.081755	3.834020
C	-0.547388	3.124977	4.716325
C	0.788258	3.526135	4.880218
C	1.795359	2.875898	4.147215
C	1.474648	1.821937	3.276888
C	-1.763773	-0.792478	2.799987
C	-2.744304	-1.362950	1.974298
C	-3.779105	-2.138920	2.518499
C	-3.854829	-2.330420	3.905399
C	-2.890609	-1.741186	4.746383
C	-1.846150	-0.982455	4.198446
C	1.004052	-1.252777	2.568250
Si	1.440033	-2.787541	1.489783
C	-0.174317	-3.721767	1.054699
C	0.042860	-4.958892	0.164252
C	2.633446	-3.844140	2.571628
C	3.467439	-4.883117	1.793005
C	1.952276	-4.509810	3.787195
C	-2.090406	1.535504	-0.792369
N	-3.474647	1.581136	-0.962107
C	-3.881333	2.702752	-1.689782
C	-2.760620	3.403611	-1.987921
N	-1.685540	2.700638	-1.441458
C	-4.500518	0.697095	-0.457109
C	-4.839417	0.763856	0.925724
C	-5.892840	-0.055510	1.374617
C	-6.602387	-0.884378	0.495794
C	-6.303495	-0.872920	-0.868261
C	-5.270678	-0.062595	-1.384298
C	-0.387950	3.340104	-1.555410
C	-0.132888	4.431159	-0.687921
C	0.967008	5.259281	-0.982656
C	1.799181	4.988757	-2.073990
C	1.558685	3.872482	-2.884579
C	0.452164	3.033607	-2.657303
C	-4.129174	1.727337	1.886411
C	-4.456493	1.462904	3.366770
C	-5.141680	0.059138	-2.906152
C	-6.338638	0.866445	-3.465805
C	-1.017381	4.728343	0.522117
C	-0.182851	4.919865	1.801712
C	0.150539	1.848915	-3.568444
C	1.387754	1.273177	-4.276739
C	-4.451125	3.209970	1.575978

C	-5.045570	-1.294360	-3.638521
C	-1.926582	5.951889	0.278011
C	-0.947899	2.163163	-4.607194
C	5.080981	0.338142	1.708334
C	2.807758	-5.267143	-2.133837
C	-1.025047	-4.128779	2.279106
H	-0.757498	-2.979636	0.470007
H	-4.930694	2.899404	-1.895487
H	-2.610772	4.333819	-2.533052
H	-1.088702	-0.542712	4.858953
H	-0.239298	1.066998	-2.892760
H	0.740833	-1.531132	3.605368
H	1.956426	-0.699268	2.627097
H	6.705998	-1.766210	2.333541
H	5.483414	-3.054362	2.465727
H	5.471956	-1.681065	3.619427
H	0.670944	-5.714920	0.673661
H	0.522406	-4.709344	-0.792722
H	-0.925440	-5.436475	-0.072541
H	2.659215	5.636963	-2.280664
H	5.148140	-3.525052	-3.251475
H	3.686452	-1.248949	1.995964
H	3.335129	-3.095006	2.981542
H	-4.217298	0.615616	-3.131675
H	-1.911689	1.773494	3.728756
H	7.114808	-2.413982	-2.191472
H	2.286509	1.341995	2.719858
H	6.856940	-1.343657	0.045316
H	4.033699	0.135077	-2.077608
H	-6.164277	-0.060747	2.433017
H	-2.674664	-1.203861	0.893578
H	1.179507	6.121382	-0.339204
H	1.681773	-3.452984	-1.856783
H	5.535570	2.054164	-1.903380
H	-1.666837	3.850296	0.687335
H	-3.042054	1.578864	1.734817
H	1.232049	2.068270	0.594103
H	2.241924	3.652066	-3.710413
H	-7.409102	-1.523167	0.878133
H	-2.948208	-1.883786	5.833232
H	-6.211094	1.037824	-4.551245
H	-6.453841	1.849262	-2.974062
H	-7.285443	0.313623	-3.317664
H	-2.058347	-4.361852	1.959288
H	-1.091424	-3.348776	3.055012
H	-0.620820	-5.038914	2.756112
H	-6.894734	-1.485968	-1.557851
H	2.342457	-2.360917	-4.038096
H	1.554977	-3.953274	-4.256511
H	3.317838	-3.805917	-4.450822
H	-4.660376	-2.939053	4.335806
H	-4.520542	-2.590592	1.851090
H	5.099729	0.663344	2.767232
H	4.357431	0.963123	1.168768
H	6.082326	0.534565	1.281409
H	2.843508	3.184844	4.251356
H	1.729163	1.935382	-5.095717
H	1.132848	0.294876	-4.721382
H	2.223407	1.123644	-3.574946
H	1.042314	4.342719	5.567932

H	-1.348056	3.626163	5.276212
H	-5.526314	1.649436	3.577184
H	-3.890065	2.160782	4.008376
H	-4.209867	0.435065	3.674540
N	5.203656	4.223102	-0.287311
H	-5.975414	-1.884004	-3.532806
H	-4.209065	-1.902934	-3.264045
H	-4.888567	-1.119185	-4.719016
H	0.467526	4.050444	1.990264
H	-0.845937	5.035783	2.676444
H	0.452560	5.823572	1.745941
H	-1.898795	2.461198	-4.136101
H	-1.146761	1.265382	-5.221145
H	-0.620039	2.978678	-5.280579
H	4.209738	-5.365487	2.461733
H	4.018442	-4.435177	0.948503
H	2.827132	-5.688837	1.387563
H	3.733900	-5.620818	-2.626129
H	1.955819	-5.835956	-2.554052
H	2.882330	-5.505507	-1.059137
H	2.772672	3.929570	0.897341
H	1.346149	-5.381132	3.480299
H	1.286438	-3.822056	4.341933
H	2.715655	-4.882951	4.499824
H	-4.157505	3.512792	0.560127
H	-3.908421	3.863813	2.283991
H	-5.534465	3.401672	1.695728
H	-1.319016	6.860513	0.104500
H	-2.572410	6.134558	1.157553
H	-2.581107	5.805959	-0.598952
H	1.742674	-1.121289	-2.338993
H	-2.417648	-0.234259	-2.877060
H	-0.375265	-1.267031	-3.314892
H	-0.640379	-3.709721	-3.198369
H	-1.697768	-5.953150	-3.065710
H	-3.743743	-6.305807	-1.658384
H	-4.723386	-4.369823	-0.391976
H	-3.709791	-2.123946	-0.575820
C	6.534518	4.138791	-0.857118
C	4.745348	5.582863	-0.054022
H	7.172700	4.921114	-0.406258
H	6.993990	3.158749	-0.634476
H	6.551710	4.284615	-1.963460
H	5.389092	6.091650	0.692054
H	4.769513	6.193178	-0.987569
H	3.711554	5.600180	0.319860

Table S130. Cartesian geometry of 3g-INT3 (33.2 kcal/mol) in Figure S146 in Angstrom [Å].

Atomtype	X Coordinates	Y Coordinates	Z Coordinates
C	-1.772009	-2.988395	2.228305
C	-1.073116	-1.912440	2.818796
C	-1.086405	-1.812028	4.229477
C	-1.755547	-2.766306	5.015132
C	-2.434396	-3.837949	4.412993
C	-2.443591	-3.939063	3.012064
P	-0.003249	-0.801407	1.711717
C	1.022183	-2.181444	1.005600
Si	2.717522	-2.077270	0.082345
C	4.127244	-2.712138	1.238816
C	3.837908	-4.086585	1.879836

Ni	-0.730165	0.314749	-0.205977
C	-2.502551	1.106993	-0.487056
N	-3.775846	0.598504	-0.722386
C	-4.653506	1.571308	-1.206516
C	-3.955692	2.732745	-1.263889
N	-2.659561	2.451606	-0.827049
C	-4.290362	-0.710160	-0.395253
C	-4.461051	-1.048603	0.978673
C	-4.990871	-2.321653	1.266432
C	-5.383181	-3.197213	0.244311
C	-5.281871	-2.805293	-1.093245
C	-4.743531	-1.552445	-1.447603
C	-1.780420	3.593532	-0.657911
C	-1.972566	4.388822	0.501105
C	-1.358016	5.655918	0.543602
C	-0.546255	6.094826	-0.508437
C	-0.320319	5.264236	-1.615438
C	-0.943179	4.004974	-1.726112
C	-4.169404	-0.043689	2.101784
C	-5.155107	1.150597	2.062783
C	-4.756780	-1.128282	-2.918840
C	-4.192029	-2.191177	-3.883910
C	-2.812312	3.908442	1.682765
C	-4.184697	4.613716	1.723177
C	-0.753597	3.134490	-2.965329
C	-1.923328	3.278171	-3.963640
Si	-0.727848	-0.393095	-2.354328
C	-1.004242	-2.284156	-2.324767
C	-1.816729	-2.889005	-1.343587
C	-2.147374	-4.252866	-1.399258
C	-1.665549	-5.044792	-2.453878
C	-0.839371	-4.467062	-3.434768
C	-0.507033	-3.103952	-3.366037
Ge	1.502648	0.785239	-0.812560
N	1.834832	2.434325	0.033909
C	0.972806	-0.028539	3.079308
C	0.463834	1.142781	3.676106
C	1.039906	1.662559	4.846121
C	2.132990	1.009872	5.439884
C	2.639610	-0.166188	4.859674
C	2.060899	-0.684159	3.690854
N	3.005613	-0.412533	-0.408768
C	4.248504	0.256577	-0.676031
C	4.967372	0.911746	0.383763
C	6.088531	1.706953	0.078374
C	6.528006	1.889206	-1.236209
C	5.858332	1.224176	-2.267751
C	4.743397	0.398162	-2.020659
C	4.589614	0.780513	1.858724
C	4.205762	2.126950	2.498972
C	4.165704	-0.358182	-3.220614
C	5.247184	-1.270595	-3.852922
C	2.440906	-3.335326	-1.370408
C	1.573713	-4.563244	-1.008579
C	3.729222	-3.836700	-2.051816
C	5.723586	0.137309	2.689450
C	3.599483	0.566154	-4.321660
C	-4.232535	-0.647824	3.514150
C	-6.199530	-0.766620	-3.348960
C	-2.073050	4.086161	3.027047

C	0.581235	3.366944	-3.693573
C	5.529809	-2.725766	0.586339
H	1.869222	-2.737351	-2.110634
H	-5.694734	1.345710	-1.422473
H	-4.248291	3.738793	-1.560017
H	-0.562650	-0.995449	4.735464
H	-0.755416	2.094220	-2.595857
H	0.322363	-2.569973	0.245126
H	1.104302	-2.985317	1.760172
H	6.578181	0.832755	2.792699
H	6.106979	-0.789799	2.236598
H	5.362602	-0.093923	3.709427
H	4.208114	-4.627704	-1.444203
H	4.474325	-3.045437	-2.209254
H	3.502522	-4.285131	-3.040422
H	-0.067520	7.081103	-0.458712
H	6.216640	1.338683	-3.298796
H	3.698809	0.136501	1.910215
H	4.162159	-1.968410	2.056316
H	-4.125729	-0.229054	-3.022186
H	-0.414067	1.632602	3.241253
H	7.392132	2.530694	-1.453506
H	2.444488	-1.619624	3.272391
H	6.623278	2.206450	0.896357
H	-5.110671	-2.636688	2.306234
H	-1.777120	-3.111056	1.144515
H	-1.504380	6.296178	1.421427
H	3.340023	-0.989478	-2.850321
H	-2.987803	2.827984	1.547158
H	-3.142498	0.338146	1.932260
H	0.339183	5.609733	-2.417782
H	-5.791112	-4.184285	0.498381
H	-1.742057	-2.666941	6.108346
H	-6.208192	-0.382812	-4.386486
H	-6.661622	-0.003914	-2.697118
H	-6.847469	-1.662806	-3.311971
H	1.495923	-5.242568	-1.879728
H	0.541401	-4.301538	-0.728922
H	2.014650	-5.147001	-0.178779
H	-5.621304	-3.483853	-1.883753
H	2.805007	1.222635	-3.931670
H	3.173670	-0.037302	-5.146329
H	4.394667	1.205270	-4.750911
H	-2.952189	-4.584565	5.028533
H	-2.975129	-4.762824	2.518433
H	3.922766	1.980404	3.556607
H	3.352046	2.568103	1.972798
H	5.048365	2.843567	2.467757
H	3.486683	-0.687729	5.322458
H	0.583923	4.330248	-4.238927
H	0.737536	2.565636	-4.437111
H	1.434527	3.353471	-2.997975
H	2.588233	1.414143	6.352896
H	0.629813	2.576700	5.294463
H	-5.276940	-0.878992	3.799617
H	-3.843348	0.081675	4.247109
H	-3.639630	-1.564941	3.609788
H	-4.830831	-3.093324	-3.916892
H	-3.175123	-2.500082	-3.603417
H	-4.157606	-1.777113	-4.908741

H	-1.038408	3.707754	2.970525
H	-2.604762	3.539975	3.828685
H	-2.024402	5.149205	3.328245
H	-2.890005	2.989558	-3.519702
H	-1.750463	2.625266	-4.839132
H	-1.999860	4.323286	-4.321023
H	6.308869	-2.908932	1.354302
H	5.774072	-1.774812	0.081889
H	5.622684	-3.535376	-0.156677
H	5.984395	-0.670632	-4.418900
H	4.789441	-1.990709	-4.557016
H	5.809485	-1.837792	-3.090573
H	3.831341	-4.892145	1.122093
H	2.868472	-4.123432	2.412070
H	4.625437	-4.344452	2.617062
H	-5.090829	1.745925	1.141047
H	-4.949848	1.829552	2.910500
H	-6.194898	0.786256	2.166392
H	-4.054768	5.708599	1.816366
H	-4.778227	4.263736	2.587775
H	-4.768176	4.413622	0.807695
H	1.944743	1.194093	-2.235940
H	-1.925760	0.155278	-3.093047
H	0.374309	-0.228337	-3.368131
H	0.137235	-2.668072	-4.141903
H	-0.448839	-5.084116	-4.254812
H	-1.923367	-6.110423	-2.508497
H	-2.795305	-4.687255	-0.627711
H	-2.223383	-2.261887	-0.544055
C	2.756448	3.390055	-0.552714
C	0.978333	2.991483	1.056320
H	1.478300	3.112504	2.045292
H	0.603730	3.989046	0.762741
H	0.093960	2.348704	1.212924
H	3.488035	3.773675	0.192496
H	3.349803	2.923523	-1.358615
H	2.225299	4.277502	-0.973136

Table S131. Cartesian geometry of $[Ph(H)_2Si]_2$ in Figure S146 in Angstrom [Å].

Atomtype	X Coordinates	Y Coordinates	Z Coordinates
C	-3.251028	-0.934163	-0.989966
C	-2.704134	0.325751	-0.694298
C	-1.850908	0.506278	0.420806
C	-1.562428	-0.617955	1.230043
C	-2.109274	-1.878543	0.938127
C	-2.952481	-2.040010	-0.174347
Si	-0.969971	2.159008	0.715787
Si	0.966051	2.109325	-0.648598
H	1.935445	3.206467	-0.321432
H	-1.873961	3.301878	0.357568
H	-0.584797	2.265571	2.161375
H	-0.893063	-0.514649	2.094945
H	-1.871551	-2.738380	1.577986
H	-3.378191	-3.025226	-0.405246
H	-3.911923	-1.053940	-1.858539
H	-2.945424	1.177615	-1.346203
C	1.808184	0.430484	-0.387853
H	0.534428	2.255481	-2.079830
C	1.169563	-0.763739	-0.804546

C	1.757685	-2.016552	-0.567695
C	2.995380	-2.101159	0.093594
C	3.642009	-0.927409	0.516672
C	3.052170	0.326057	0.278737
H	0.195006	-0.721655	-1.309062
H	1.244121	-2.929606	-0.896098
H	3.455026	-3.080732	0.279534
H	4.609252	-0.987516	1.032879
H	3.572102	1.233793	0.615835

References

1. P. M. Keil, T. Szilvási, T. J. Hadlington, *Chem. Sci.* **2021**,*12*, 5582-5590.
2. J. C. DeMott, N. Bhuvanesh, O. V. Ozerov, *Chem. Sci.* **2013**,*4*, 642-649.
3. S. Harder, J. Boersma, L. Brandsma, J. A. Kanters, A. J. M. Duisenberg, J. H. Van Lenthe, *Organometallics* **1990**, *9*, 511–516.
4. S. Hunig, H. Schweeberg, H. Schwartz, *Liebigs Ann.* **1954**, *587*, 132-145.
5. Schulz, T. L. Kalkuhl, P. M. Keil, T. J. Hadlington, *Angew. Chem. Int. Ed.* **2023,** *62*, e202305996.
6. J. Sicard, R. T. Baker, *Org. Proc. Res. Dev.* **2020**, *24*, 2950-2952.
7. B.-C. Liu, N. Ge, Y.-Q. Zhai, T. Zhang, Y.-S. Ding, Y.-Z. Zheng, *Chem. Commun.* **2019**, *55*, 9355-9358.
8. M. Muhr, P. Heiß, M. Schütz, R. Bühler, C. Gemel, M. H. Linden, H. B. Linden, R. A. Fischer, *Dalton Trans.*, **2021**, *50*, 9031−9036.
9. Hansch, A. Leo, R. W. Taft, *Chem. Rev.* **1991**, *91*, 165-195.
10. Grimme, S., Semiempirical GGA-type density functional constructed with a long-range dispersion correction. J Comput Chem 2006, 27 (15), 1787-99.
11. Aleksandr V. Marenich, C. J. C., * and Donald G. Truhlar*, Universal Solvation Model Based on Solute Electron Density and on a Continuum Model ofthe Solvent Defined by the Bulk Dielectric Constant and Atomic Surface Tensions. The Journal of Physical Chemistry B 2009, 113 (18), 6378–6396.
12. Weigend, F.; Ahlrichs, R., Balanced basis sets of split valence, triple zeta valence and quadruple zeta valence quality for H to Rn: Design and assessment of accuracy. Phys Chem Chem Phys 2005, 7 (18), 3297-305.
13. Frisch, M. J.; Trucks, G. W.; Schlegel, H. B.; Scuseria, G. E.; Robb, M. A.; Cheeseman, J. R.; Scalmani, G.; Barone, V.; Petersson, G. A.; Nakatsuji, H.; Li, X.; Caricato, M.; Marenich, A. V.; Bloino, J.; Janesko, B. G.; Gomperts, R.; Mennucci, B.; Hratchian, H. P.; Ortiz, J. V.; Izmaylov, A. F.; Sonnenberg, J. L.; Williams; Ding, F.; Lipparini, F.; Egidi, F.; Goings, J.; Peng, B.; Petrone, A.; Henderson, T.; Ranasinghe, D.; Zakrzewski, V. G.; Gao, J.; Rega, N.; Zheng, G.; Liang, W.; Hada, M.; Ehara, M.; Toyota, K.; Fukuda, R.; Hasegawa, J.; Ishida, M.; Nakajima, T.; Honda, Y.; Kitao, O.; Nakai, H.; Vreven, T.; Throssell, K.; Montgomery Jr., J. A.; Peralta, J. E.; Ogliaro, F.; Bearpark, M. J.; Heyd, J. J.; Brothers, E. N.; Kudin, K. N.; Staroverov, V. N.; Keith, T. A.; Kobayashi, R.; Normand, J.; Raghavachari, K.; Rendell, A. P.; Burant, J. C.; Iyengar, S. S.; Tomasi, J.; Cossi, M.; Millam, J. M.; Klene, M.; Adamo, C.; Cammi, R.; Ochterski, J. W.; Martin, R. L.; Morokuma, K.; Farkas, O.; Foresman, J. B.; Fox, D. J. Gaussian 16 Rev. C.01, Wallingford, CT, 2016.
14. Harvey, J. N.; Himo, F.; Maseras, F.; Perrin, L., Scope and Challenge of Computational Methods for Studying Mechanism and Reactivity in Homogeneous Catalysis. ACS Catalysis 2019, 9 (8), 6803-6813.
15. Glendening, E. D. R., A.E.; Carpenter, J.E., Weinhold, F. NBO Version 3.1.
16. Avogadro: an open-source molecular builder and visualization tool. Version 1.20.
17. Hanwell, M. D.; Curtis, D. E.; Lonie, D. C.; Vandermeersch, T.; Zurek, E.; Hutchison, G. R., Avogadro: an advanced semantic chemical editor, visualization, and analysis platform. Journal of Cheminformatics 2012, 4 (1), 17.
